# Single-carbon insertion enables conversion of cyclopropenes into cyclobutenes: access to oxaspiro[2.3]hexenes

**DOI:** 10.1039/d6sc02968j

**Published:** 2026-07-01

**Authors:** Maria Chiara Cabua, Iktedar Mahdi, Ernesto Mesto, Emanuela Schingaro, Francesco Secci, Sehrish Sarfaraz, Marco Colella, Philipp Natho, Nadeem S. Sheikh, Renzo Luisi

**Affiliations:** a Department of Pharmacy-Drug Sciences, University of Bari Aldo Moro Via E. Orabona 4 70125 Bari Italy Renzo.Luisi@uniba.it; b Dipartimento di Scienze Chimiche e Geologiche, Università degli Studi di Cagliari S.S. 554, bivio per Sestu Monserrato (Ca) Italy; c Department of Earth and Geoenvironmental Sciences, University of Bari Aldo Moro Via E. Orabona 4 70125 Bari Italy; d Department of Chemistry GGDC Chitti Dheri Mansehra, Higher Education Department KP 21300 Pakistan; e Chemical Sciences, Faculty of Science, Universiti Brunei Darussalam Jalan Tungku Link Gadong BE1410 Brunei Darussalam

## Abstract

We report 1-oxaspiro[2.3]hex-4-enes as a novel strained spiro heterocycle. This motif was accessed *via* a formal C1 insertion into cyclopropenes. The method employs dihalocarbenes to trigger a skeletal editing process involving *in situ* bicyclo[1.1.0]butane formation and strain-release-driven ring expansion, delivering halogenated cyclobutenes under simple conditions. A broad substrate scope is demonstrated, enabling access to chloro-, bromo-, and fluoro-substituted spirocycles, including derivatives of bioactive molecules. Mechanistic studies supported by *in silico* calculations demonstrate an asynchronous, concerted carbene addition followed by intramolecular epoxidation and ring expansion as the rate-determining step.

## Abstract 2

A skeletal editing strategy converts cyclopropene carbinols into halogenated 1-oxaspiro[2.3]hex-4-enes *via* dihalocarbene addition & strain-release ring expansion, providing access to a rare strained spirocyclic scaffold with broad substrate scope.

## Graphical abstract

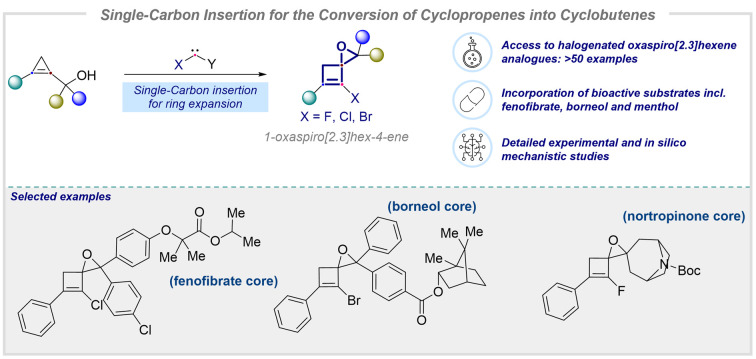

## Introduction

**Scheme 1:**
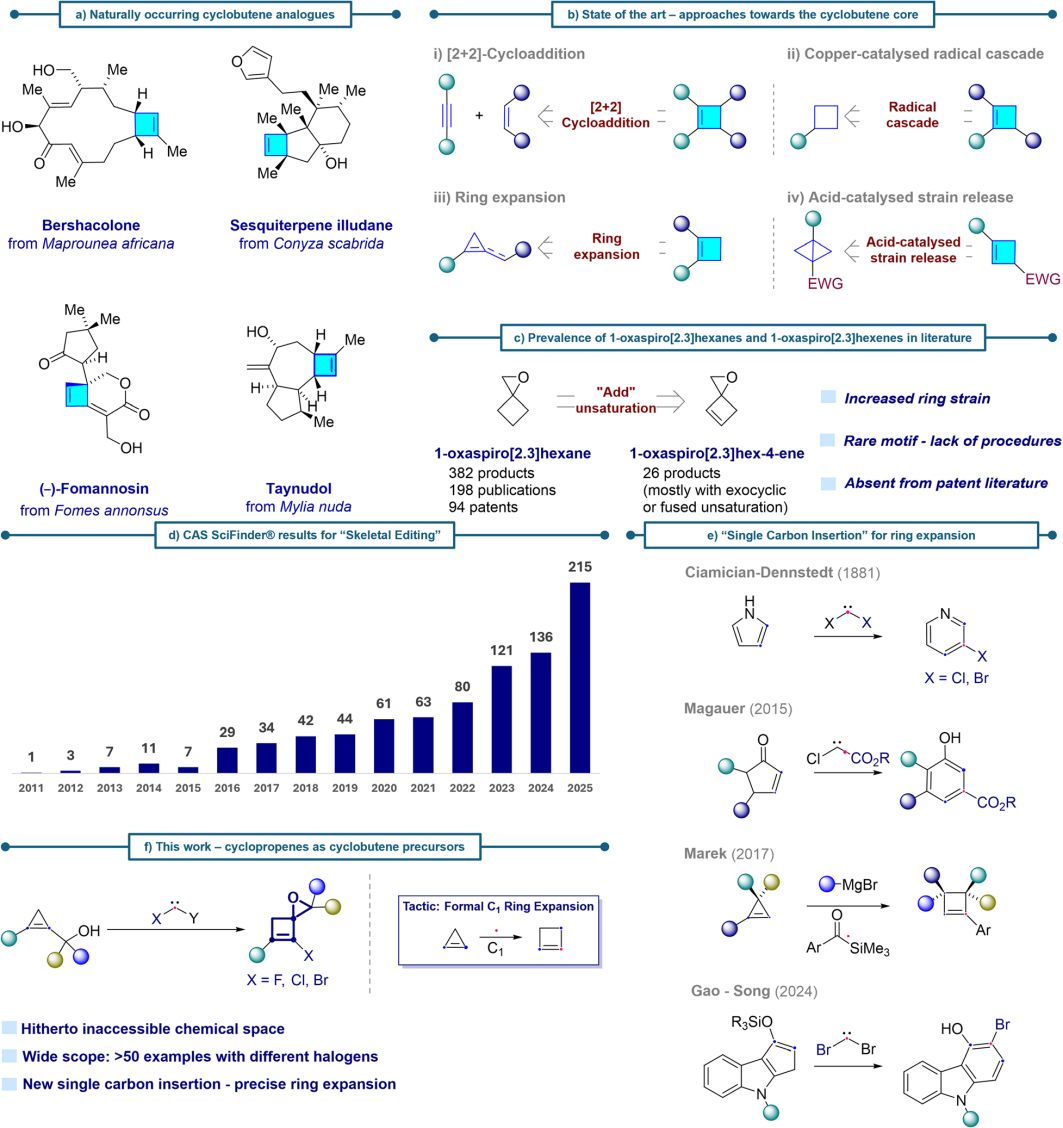
(a) Naturally occurring cyclobutene analogues; (b) state-of-the-art approaches to the cyclobutene core; (c) prevalence of spiro[2.3]hexanes and spiro[2.3]hexenes in literature. Data obtained from CAS SciFinder® accessed on 27th November 2025; (d) CAS SciFinder® results for “Skeletal Editing”. Keyword search accessed on 27th November 2025; (e) “Single Carbon Insertion” for ring expansion – selected examples; (f) this work – single carbon insertion to convert cyclopropenes into cyclobutenes.

Cyclobutenes – strained four-membered carbocycles bearing a double bond – are valuable synthetic building blocks in pharmaceuticals and materials science and are frequently encountered in natural products (Scheme 1a). Their synthetic utility stems from their rich and distinctive reactivity, particularly in ring-opening and cycloaddition transformations.^1–3^ Classical approaches to these carbocyclic motifs rely on [2 + 2]-photocycloadditions between olefins and alkynes, which provide good regio- and stereocontrol but often suffer from limited substrate scope and poor scalability. More recently, catalytic enantioselective [2 + 2]-cycloadditions between alkynes and alkenes have significantly expanded the synthetic toolbox, enabling the efficient preparation of chiral cyclobutenes from simple precursors under mild conditions (Scheme 1b).^4–6^ Beyond cycloadditions, innovative strategies have further diversified access to functionalized cyclobutenes. For example, copper-catalyzed radical cascade processes starting from cyclobutanes allow selective cleavage of multiple C–H bonds and the direct installation of amino, sulfonyl, or bromo substituents, affording densely functionalized cyclobutenes in good yields.^7^ Transition metal-catalyzed ring expansion of alkylydenecyclopropanes represents an additional route towards mono- and di-substituted cyclobutenes (Scheme 1b).^8–10^ Brønsted acid catalysis has also emerged as a powerful platform, promoting strain-release cascades from bicyclo[1.1.0]butanes to deliver enantioenriched cyclobutenes (Scheme 1b).^11^ In parallel, within the broader context of strained heterocycles,^12^ saturated spiro-heterocycles containing six total ring atoms (*i.e.*, spiro[2.3]hexanes) have attracted increasing interest as potential bioisosteres in early drug-discovery programs,^13–18^ owing to their high sp^3^-carbon content and reduced conformational flexibility.^19–22^

Whereas saturated derivatives such as 1-oxaspiro[2.3]hexanes are well represented in the literature, the corresponding unsaturated cyclobutene analogues (1-oxaspiro[2.3]hexenes) remain comparatively rare (Scheme 1c). Specifically, over 400 1-oxaspiro[2.3]hexane derivatives are reported in over 200 publications and 94 patents demonstrating growing accessibility of this motif. In stark contrast, reports of the unsaturated 1-oxaspiro[2.3]hex-4-ene analogue are rather diffuse, and only 26 derivatives are reported – notably, however, the majority of those contain the cyclobutane system fused to an aromatic ring, placing the alkene outside the four-membered ring.^23^ Consequently, the development of practical and broadly applicable strategies to access oxaspiro[2.3]hexenes represents a timely and important challenge in synthetic methodology.

Among the most innovative approach in synthetic development, “skeletal editing” has recently impacted organic synthesis by enabling the precise insertion, deletion, or exchange of atoms within core molecular scaffolds. A keyword search for “skeletal editing” on CAS SciFinder® powerfully demonstrates the growing interest in this tactic across synthetic chemistry (Scheme 1d).^23^ This strategy often exploits the reactivity of strained intermediates to re-configure ring systems with remarkable step economy. This “build–edit–decorate” strategy, where simple precursors are assembled, selectively reshaped, and subsequently diversified, mirrors the flexibility of biosynthetic pathways while circumventing the constraints of traditional *de novo* synthesis approaches.^24–29^ In the context of “skeletal editing”, the possibility of transforming a carbocycle into its homologated analogue through single-carbon insertion using a simple procedure on a preformed precursor is particularly appealing. Moreover, whereas C- or N-insertions into heterocycles such as pyrroles and indoles are now well-established through methods relying on Ciamician–Dennstedt-type rearrangements,^30–32^ or nitrene-based chemistry,^33–35^ analogous one-carbon insertions into carbocycles remain underdeveloped. The difficulty arises from challenges in achieving selective C–C bond activation while managing ring strain. Nonetheless, such carbocyclic skeletal edits hold significant promise, offering streamlined routes to bioactive motifs prevalent in pharmaceuticals and natural products.^36–39^ Several examples based on one-carbon introduction have been reported and some representative examples are shown in Scheme 1e. For example, Magauer described the conversion of α,β-unsaturated cyclopentenones into the corresponding phenols using a chlorocarbene precursor.^40^ Similarly, Gao and Song reported the synthesis of carbazoles *via* carbon insertion into 3,4-dihydrocyclopenta[*b*]indoles.^41^ In contrast, Marek reported an elegant stereoselective ring expansion of cyclopropenes to the corresponding cyclobutenes through a sequence involving carbomagnesiation, reaction with acylsilanes, and a Brook rearrangement, ultimately generating a carbene intermediate responsible for cyclobutene formation.^42^

In continuation of our interest in the synthetic development of novel strained spiro heterocycles, we herein describe the successful use of cyclopropenes as a platform for the synthesis of spiro[2.3]hexenes through a single-carbon insertion strategy (Scheme 1f). This approach aligns with the principles of skeletal editing by expanding three-membered rings into four-membered scaffolds with controlled substitution patterns. By harnessing cyclopropene ring strain to drive migratory insertion, the method addresses the longstanding scarcity of C1-insertion processes in strained carbocycles and provides a versatile platform for diversity-oriented synthesis of cyclobutene derivatives.^43–45^

## Design plan and optimization

**Scheme 2:**
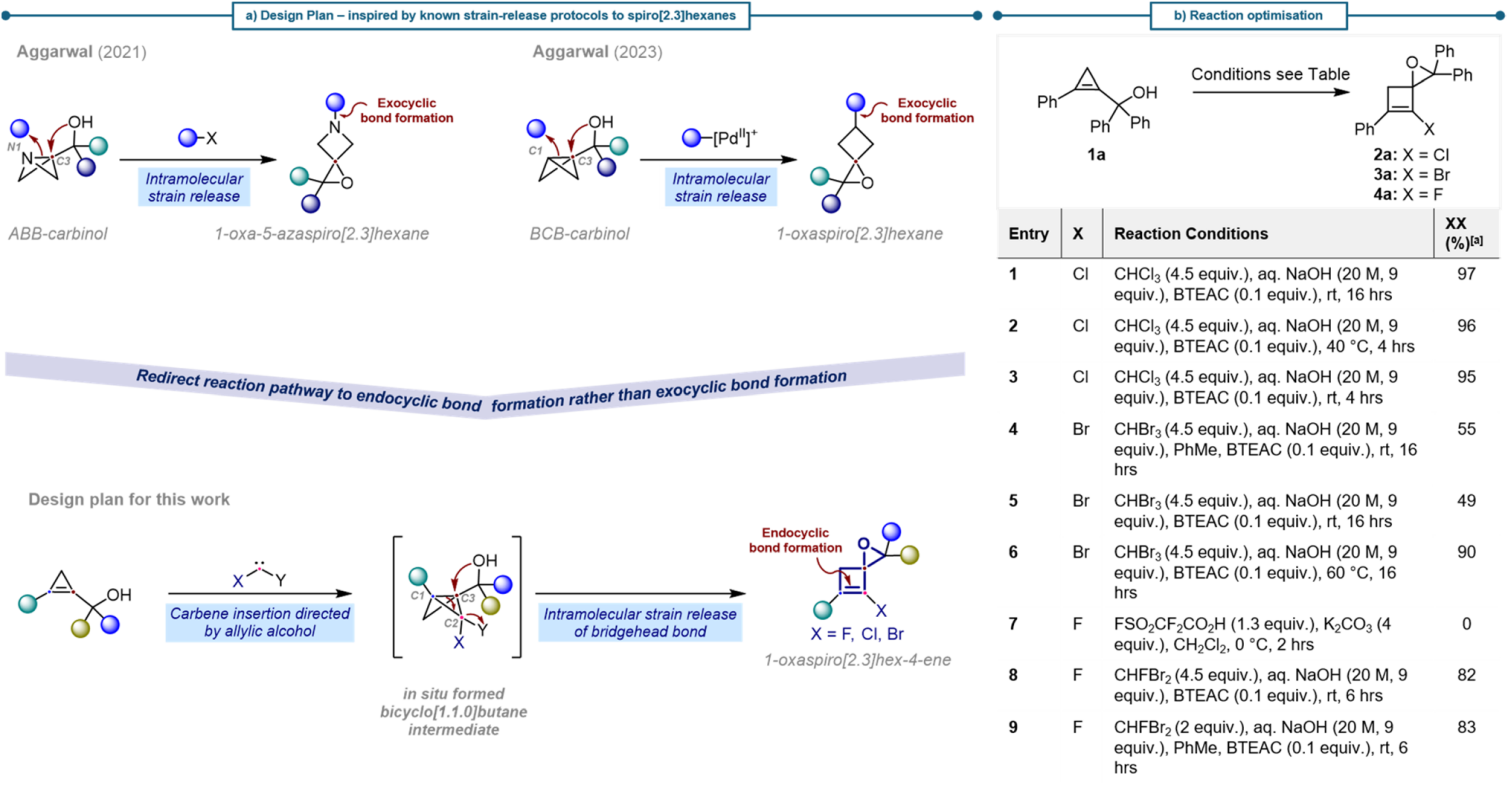
(a) Design plan – inspired by known strain-release protocols towards 1-oxa-5-azaspiro[2.3]hexanes and 1-oxaspiro[2.3]hexanes; (b) reaction optimization ^*a*^Quantitative ^1^H-NMR yields using 1,3,5-trimethoxybenzene as internal standard.

To pursue our objectives, we found inspiration from reported strategies for the synthesis of 1-oxaspiro[2.3]hexanes and 1-oxa-5-azaspiro[2.3]hexanes, which rely on the intramolecular epoxidation by strain-release of a “springloaded” bridgehead bond on azabicyclobutane- and bicyclobutane (BCB)-substituted carbinols, respectively (Scheme 2a).^46–50^ These intramolecular epoxidations are typically induced by activation of the N1 or C1 on the bridgehead, respectively, promoting formation of a partially positive charge on C3, which is intramolecularly quenched by formation of a carbon–oxygen bond from the pendant carbinol motif. Additional bond-formation on C1 or the N1 atoms with a coupling partner or electrophile would occur in an exocyclic fashion. Thus, we questioned if inclusion of a suitable leaving group on C2 of a putative BCB could divert the reaction pathway upon strain release by epoxidation towards endocyclic double bond formation (Scheme 2a). We hypothesized that reaction between cyclopropene-substituted carbinols^51^ with dihalocarbenes could form *in situ* a BCB-substituted carbinol, which under the basic conditions required for halocarbene formation, could spontaneously undergo strain-release of the bridgehead bond and intramolecular epoxidation. In the absence of an external electrophile, electrons of the C1–C3 σ-bond would be forced towards olefin formation by departure of one of the installed halides on C2 – thus forming the desired spiro[2.3]hex-4-ene with a halogen functional handle for further derivatization. In line with this design plan, we began our study with a proof of concept using carbinol 1a as a model substrate (Scheme 2b). Our objective was to affect the transformation ideally with varying halogenation of the alkene, so that various halocarbene precursors were studied. We began our study towards the formation of chlorinated product 2a. Upon optimization of the reaction conditions, we found that treatment of carbinol 1a in chloroform with a 20 M aqueous solution of sodium hydroxide and 0.1 equivalents of benzyltriethylammonium chloride (BTEAC) as the phase transfer catalyst at room temperature for 16 hours delivered the best results. Increase of temperature, or reduction of the reaction duration, though, had negligible effects on the reaction outcome, so that reaction conditions displayed in Table 1, Entry 3 were considered optimal. Pleasingly, a scale-up of the reaction to 4 mmol afforded the *ca.* 1.2 g of the desired product with minimal variation in yield (86%), demonstrating the scalability of the approach. For the synthesis of brominated product 3a, we tested various conditions with bromoform as the carbene precursor. Under analogous conditions used with chloroform, lower yields are observed. Similarly, only modest yields are observed when the reaction is not performed under neat conditions. Optimal performance was found, when the ring expansion was performed neat at elevated temperatures with 0.1 equiv. of BTEAC, pleasingly forming the brominated product 3a in 90% yield. Last, we sought to extend the reaction to the synthesis of fluorinated product 4a. As fluoroform is a gas under ambient conditions, we studied alternative carbene precursors (see SI).

For example, treatment of carbinol 1a with trifluoromethyltrimethylsilane in THF did not afford the desired fluorinated spiro[2.3]hex-4-ene at room temperature or elevated temperatures. Similarly, leveraging 2,2-difluoro-2-(fluorosulfonyl)acetic acid as a potential carbene precursor had no success with the desired product formed not even in trace quantities despite the investigation of various bases and solvents. Last, we turned our attention to dibromofluoromethane as a mixed halomethane. Upon treatment of carbinol 1a with dibromofluoromethane (neat) and nine equivalents of aqueous sodium hydroxide solution and 0.1 equiv. of BTEAC at room temperature, the desired product 4a was formed in 82% yield. Pleasingly, the competing brominated alkene was detected only in trace quantities, and the reaction performed equally well in toluene solution (Table 1, entry 9). Control experiments in all cases confirmed total cessation of reaction in the absence of base.

## Results and discussion

**Scheme 3:**
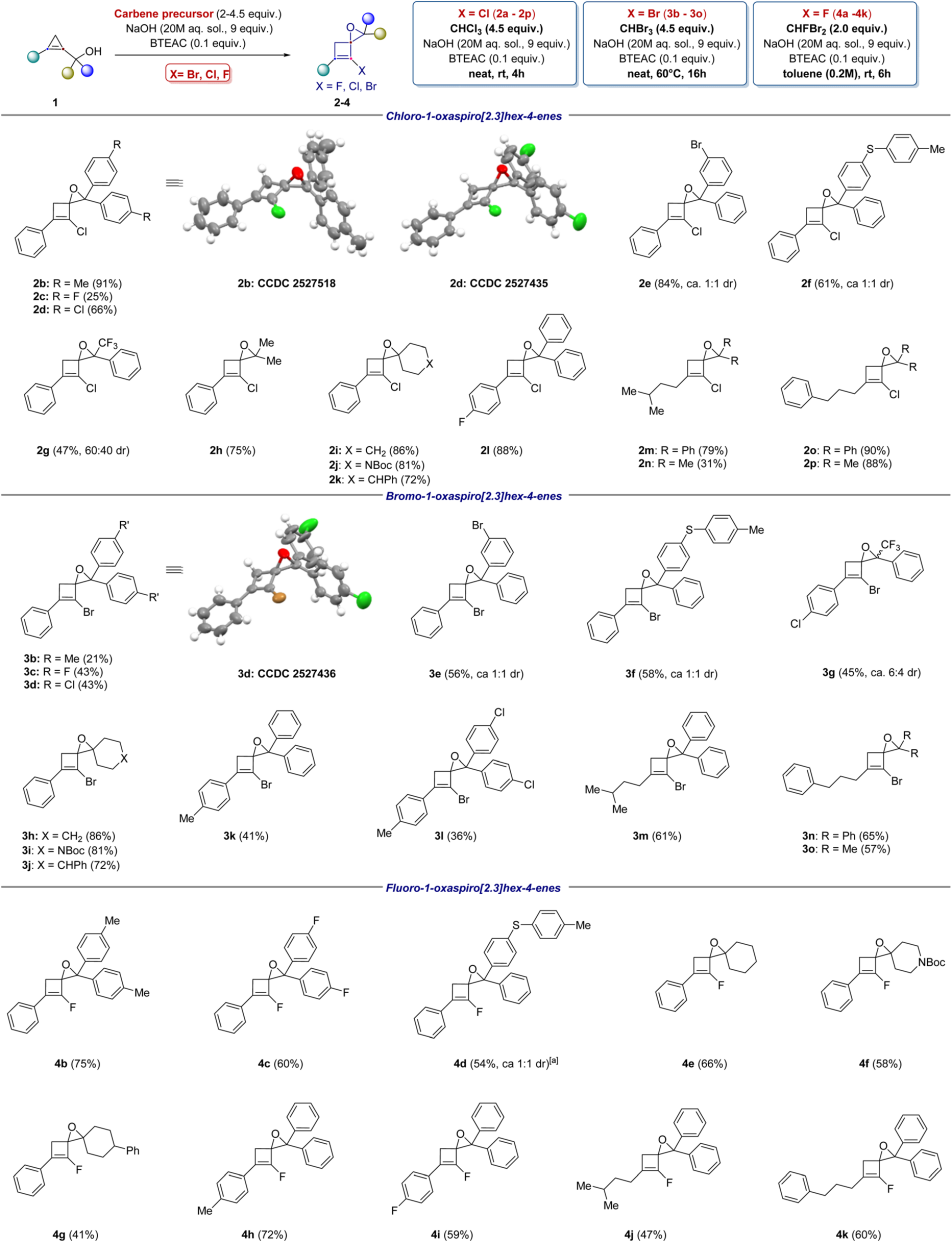
Scope exploration for the synthesis of halogenated spiro[2.3]hexenes from cyclopropenes. For further information see SI. X-ray crystallography structures for 2b, 2d and 3c exhibit positional disorder – only one of two disordered positions shown for clarity. ^*a*^Isolated as cyclopentenone 5d due to Meinwald rearrangement observed during flash column chromatography (see SI).

With a reliable protocol for the ring-expansion/halogenation for various halogens in hand, we evaluated the scope of cyclopropene-substituted carbinols that could be employed to grant divergent access to various spiro[2.3]hex-4-enes (Scheme 3). Following our optimization studies, we continued our exploration with the synthesis of chlorinated analogues. First, we studied variation of substituents on the carbinol carbon. In addition to phenyl groups, also substituted aromatic rings are tolerated with various electronic and steric properties. For example, symmetrically substituted spiro[2.3]hex-4-enes 2b–2d bearing *para*-methyl-, chloro- or fluoro-substituents are obtained in up to 91% yield. The structures of spiro[2.3]hex-4-enes 2b and 2d bearing *para*-methyl and *para*-chloro substitution on the aromatic ring were confirmed by single crystal X-ray crystallography. Sterically more challenging and asymmetric substrates bearing *meta*-bromo or *para*-thiotolyl substituents provide the desired products 2e and 2f under our standard conditions in 84% and 61% yield respectively, as a 1 : 1 mixture of diastereomers. Having studied a variety of aromatic substituents on the carbinolic carbon, we sought to accommodate aliphatic substituents. For example, trifluoromethyl-substituted derivative 2g is obtained in 47% yield after ring expansion as a mixture of diastereomers (*ca.* 6 : 4 dr). In addition, a range of other aliphatic substituted derivatives are suitable substrates bearing either acyclic or cyclic side chains. For example, dimethyl-substituted epoxide 2h is obtained in 75% yield. Cyclic derivatives affording spiro[2.3]hex-4-enes 2i–2k with an additional spiro-center are afforded in 72–86% yield, indicating that induction of additional strain on the product has little effect on the outcome of the reaction. Last, we tested the effect of variation of the substituent on the cyclopropenylic olefin on the outcome of the reaction. Replacement of the phenyl group with a *para*-fluorophenyl group (2l) had little effect. Pleasingly, replacement of the aryl-substituent with aliphatic side-chains (2m–2p) equally did not hamper the productive ring-expansion to afford the spirocyclic products in up to 90% yield. Notably, several derivatives were obtained in high purity directly from the reaction without the requirement for purification steps.

After assessing the scope for the ring expansion/chlorination series, we explored the substrate scope of the reaction for the ring expansion/bromination sequence. Analogous to our exploration under chlorinative ring expansion conditions, we began our study with testing a series of cyclopropenes bearing aromatic groups on the carbinolic carbon. These substrates smoothly underwent the desired transformation, under our standard conditions, providing brominated spiro[2.3]hex-4-enes 3b–3f in synthetically useful yields, although in most cases with marginally lower yields than their chlorinated analogues. Moreover, the structure of 3d bearing two *para*-chlorophenyl substituents was confirmed by single crystal X-ray crystallography. Similarly to our findings for the dichlorocarbene-induced ring expansion, also substrates bearing aliphatic substituents on the carbinolic centre displayed good reactivity to our standard conditions providing the brominated spiro[2.3]hex-4-enes 3g–3j in up to 86% yield. To conclude our exploration of the brominative ring enlargement, we tested substrates with varying substitution on the cyclopropenyl alkene (3k–3p). To our delight, substrates bearing substituted aromatic rings (3k–3l) or aliphatic sidechains (3m–3o) were readily transformed into the spirocycles in up to 65% yield.

To complete our objective of affecting the ring enlargement also with concomitant fluorination, we last turned our attention to study the scope for this transformation. Using the optimized conditions reported in Table 1, entry 9, various cyclopropenyl carbinols substituted with a range of aliphatic and aromatic substituents could be converted to the desired spirocycles 4b–4k in 41–75% yield. During our scope investigation we discovered that only in few cases (*e.g.*, 4c or 4g) some undesired brominated spirocycle was formed, although the extent never exceeded 20% based on the starting material. It is worth noting that fluorinated spiro[2.3]hex-4-enes displayed notable sensitivity towards acidic media, causing facile acid-mediated rearrangement to cyclopentenones during flash column chromatography. With the exception for derivative 4d, the undesired Meinwald-type rearrangement could, however, be suppressed by pre-treatment of the silica gel with triethylamine.

**Scheme 4:**
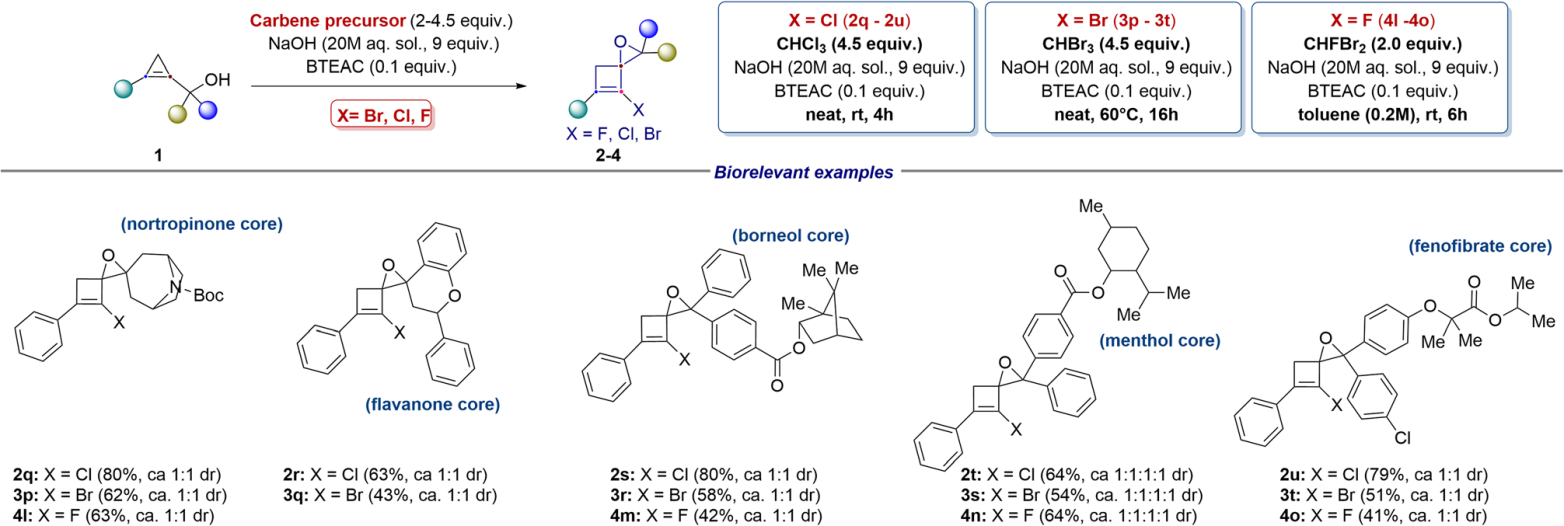
Scope exploration for the synthesis of halogenated spiro[2.3]hexenes from cyclopropenes incorporating bioactive molecules. For further information see SI.

To further highlight the versatility of this method, we extended the protocol beyond structurally simple substrates to derivatives bearing biologically relevant natural products and drug molecules (Scheme 4). For example, derivatives bearing *N*-Boc nortopinone and flavanone are converted to the corresponding spiro[2.3]hex-4-enes (2q, 3p, 4l, 2r, 3q) in 43–80% yield, with best yields typically observed for the chlorinated analogues. Next, as esters offered a useful anchor point, naturally occurring and biologically active terpenes could be introduced on the aromatic ring, including borneol (2s, 3r, 4m) and menthol (2t, 3s, 4n), affording the desired spirocycles in 42–80% yield. Notably, the base-sensitive ester moiety remained unaffected under our standard conditions. Last, substrates bearing fenofibrate, a drug for the treatment of abnormal blood lipid levels, were converted to the halogenated spiro[2.3]hex-4-enes 2u, 3t, and 4o in 41–79% yield, again with best results obtained under chlorinative conditions.

**Scheme 5:**
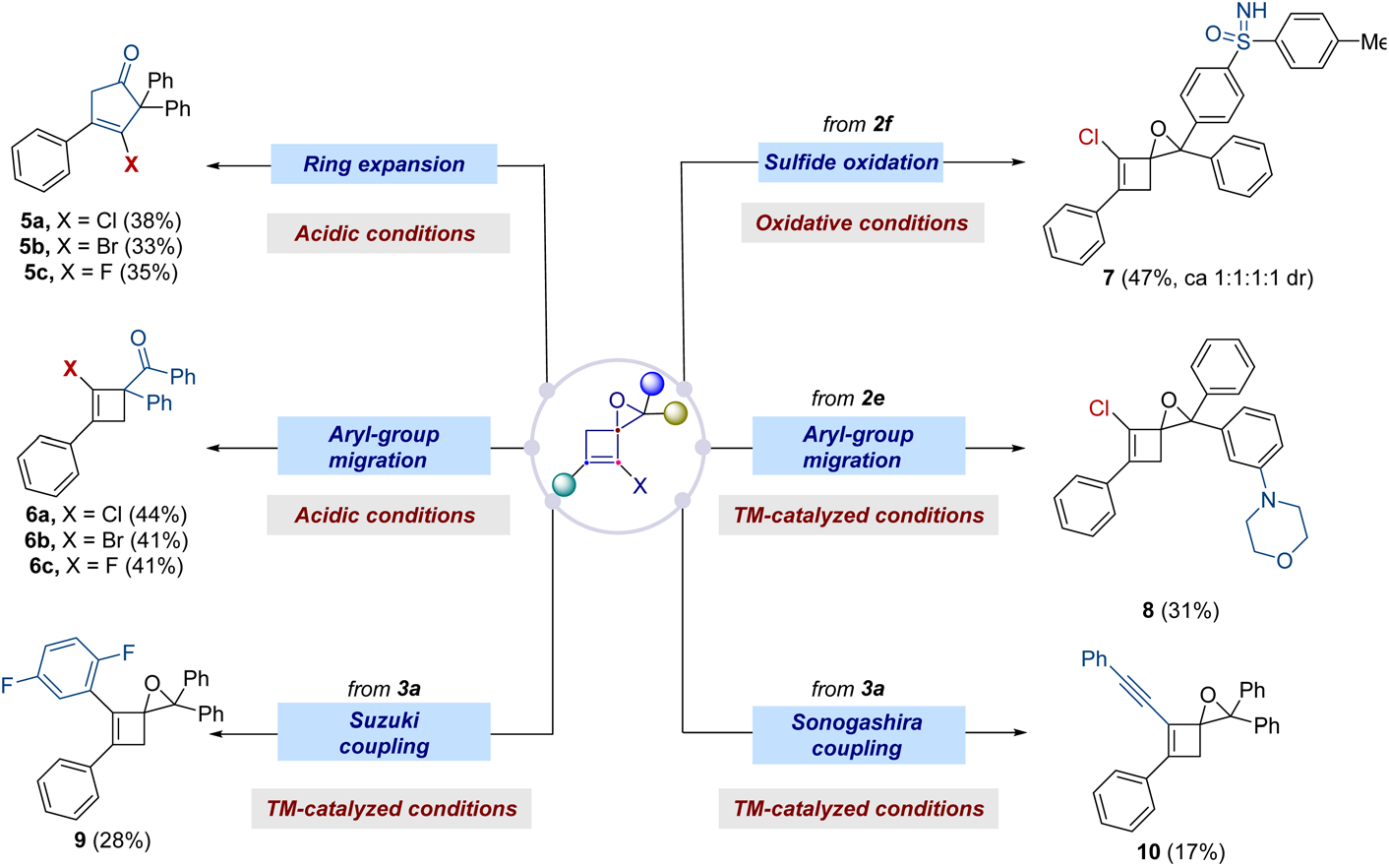
Further derivatization of halogenated spiro[2.3]hexenes. Ring expansion/aryl group migration: TFA (0.4 mL mmol^−1^), THF, 0 °C to rt, 30 min. Sulfide oxidation: [NH_4_][H_2_NCO_2_] (2 equiv.), PIDA (1.25 equiv.), MeOH, rt. Buchwald–Hartwig coupling: Pd_2_(dba)_3_ (3 mol%), XPhos (12 mol%), NaO^*t*^Bu, PhMe, 100 °C. Suzuki coupling: (2,5-difluorophenyl)boronic acid (1.2 equiv.), Pd(OAc)_2_ (5 mol%), XPhos (10 mol%), aq. K_3_PO_4_ (2M, 3 equiv.), THF, 70 °C. Sonogashira coupling: phenyl acetylene (1.2 equiv.), Pd(PPh_3_)_4_ (5 mol%), CuI (10 mol%), Et_3_N (1.2 equiv.), THF, 70 °C.

After assessing the scope, we tested the robustness of this new motif under different conditions (Scheme 5). Analogous to its saturated equivalent – 1-oxaspiro[2.3]hexane – our findings suggest that this strained spiro heterocycle is stable under oxidative conditions, in the presence of transition metals and basic conditions. However, notable sensitivity under acidic conditions is observed, which, however, can be synthetically exploited. Specifically, when 2a–4a are treated with sub-stoichiometric quantities of trifluoroacetic acid, a mixture of cyclobutenes 6a–6c, resulting from acid-catalyzed 1,2-migration of epoxide, and cyclopentenones 5a–5c, resulting from acid-catalyzed ring expansion, are obtained. Under the conditions employed, cyclobutenes 6a–6c are formed preferentially, although preliminary results indicate that reduction of the excess of acid catalyst can promote preferential formation of the cyclopentenones 5a–5c (see SI). Under oxidative conditions, sulfide-bearing spirocycle 2f could be transformed into sulfoximine 7 in the presence of PIDA and ammonium carbamate in methanol in 47% yield. In addition, the aryl–bromine bond of 1-oxaspiro[2.3]hex-4-ene 2e could be engaged in a Buchwald–Hartwig amination with morpholine, affording spirocycle 8 in 31%. In addition to aryl-bromine bonds, also the installed vinylic halides on the cyclobutene motif were suitable precursors for transition metal-catalyzed cross-coupling reactions.^52^ To this end alkenyl bromide 3a was successfully engaged in a Suzuki coupling to afford compound 9 in 28% yield, as well as in a Sonogashira coupling to afford alkyne 10 in 17% yield. This showcases the versatility of the installed functional handles for further derivatization.

**Scheme 6:**
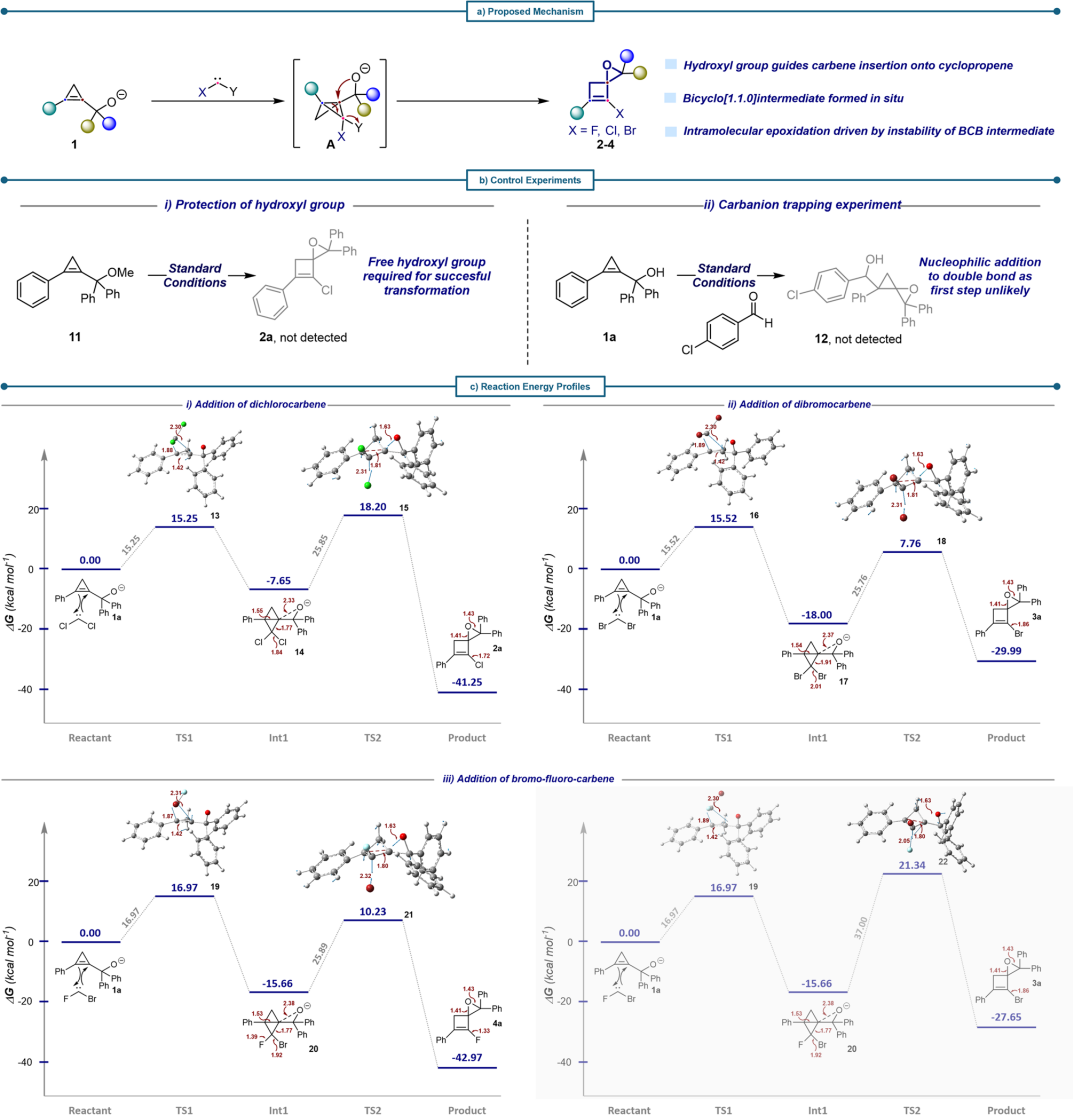
(a) Mechanistic proposal; (b) control experiments; (c) reaction energy profiles for the addition of dihalocarbene (addition of (i) dichlorocarbene (ii) dibromocarbene (iii) flourobromocarbene). All the reported energy values are presented in kcal mol^−1^ with reference initial reactant at 0.00 kcal mol^−1^. Measured bond lengths are presented in Angstroms (Å).

To conclude our study, we sought to investigate the mechanism for the observed chemical reactivity. In line with our design plan, we propose a mechanism based on the reaction between cyclopropene 1 and dihalocarbene to form initially a bicyclo[1.1.0]butane derivative A, which would spontaneously undergo intramolecular epoxidation driven by strain release (Scheme 6a). To validate this hypothesis, several experimental probes and *in silico* investigations were considered.

First, we tested the role of the carbinol moiety, which we presumed would serve a dual role as (a) pendant nucleophile for desired intramolecular epoxidation, and (b) in line with literature precedent, promoter for initial carbene addition across the unactivated alkene, which is readily observed for allylic alcohols (Scheme 6b(i)).^53^ Thus, protection of the hydroxyl moiety as the methyl ether (11), and thus precluding intramolecular epoxidation, expectedly led to complete cessation of product formation 2a. Interestingly, in this case also formation of the putative bicyclo[1.1.0]butane intermediate was not observed, which supports our hypothesis that the hydroxyl group facilitates carbene insertion into the cyclopropene. Last, we aimed to rationalize the mechanism of strain-release/halide elimination and hypothesized two potential pathways: (i) carbene insertion, followed by strain release as initially proposed; or (ii) initial nucleophilic addition across the double bond with localized carbanion formation, followed by addition to carbene. We proposed that in the case of formal carbanion formation on C1, this intermediate might be trapped with a reactive electrophile. Thus, in addition to our standard conditions, *para*-chlorobenzaldehyde was included as a suitable “anion trap” (Scheme 6b(ii)). However, the expected product 12 was not detected, which indicated to us that epoxidation as first step is unlikely.

To seek further evidence for the underlying mechanism, we turned our attention to *in silico* studies (Scheme 6c). Based on the preliminary mechanistic hypothesis and experimental evidence, a DFT study was carried out to construct a plausible reaction pathway for each ring-expansion/halogenation by identification of the reaction intermediates and all related transition states. Optimization of geometry was performed at M06-2X/6-31+G(d,p) level.^54^ The reaction between cyclopropene 1a and dichlorocarbene proceeds through a concerted, but asynchronous [2 + 1]-cycloaddition (Scheme 6c(i)). That such electrophilic attack is feasible was confirmed through determination of the electrostatic potential and other chemical features of the carbene structures (see SI). Completion of the cycloaddition culminates in the formation of bicyclic intermediate 14, which is thermodynamically more stable with respect to the reactants (−7.65 kcal mol^−1^), indicating an exothermic process. The energy profile shows that the transition state (13) for this step is easily achievable with an activation barrier of 15.25 kcal mol^−1^. Insertion of the carbene into the cyclopropene π-bond is supported by the increase of the carbon–carbon double bond length from 1.30 Å in the starting material to 1.42 Å in 13, and eventually 1.55 Å in 14. In line with our hypothesis, bicyclo[1.1.0]butane intermediate 14 is prone to an intramolecular strain release reaction by nucleophilic attack from the alkoxide anion onto the strained bridgehead carbon (carbon–oxygen interaction is 2.33 Å), proceeding through 15. This step is rate-determining with an activation energy of 25.85 kcal mol^−1^. Nucleophilic attack of the alkoxide thus triggers a highly coordinated electronic reorganization, leading to the cleavage of the bridgehead bond, and the carbon–chlorine bond, as well as the formation of the endocyclic double bond. Product formation to 2a is overall exergonic (Δ*G* = −41.52 kcal mol^−1^) with respect to the reactants.

We next investigated the reaction pathway for the reaction between dibromocarbene and cyclopropene 1a to from cyclobutene 3a (Scheme 6c(ii)). Overall, this process was found to be analogous to that with dichlorocarbene, beginning with a concerted, asynchronous [2 + 1]-cycloaddition of the dibromocarbene to the cyclopropene 1a. Whereas the energetic barrier to 16 was comparable to that for dichlorocarbene addition (15.52 kcal mol^−1^*vs.* 15.25 kcal mol^−1^), formation of 17 is significantly more exothermic in this case (−18.00 kcal mol^−1^*vs.* −7.65 kcal mol^−1^). Subsequent ring expansion by intramolecular epoxidation of the alkoxide onto the bridgehead bond and elimination of bromide forms brominated cyclobutene 3a. This step proceeds through 18, which also in this case is the rate-determining step with a comparable activation energy of 25.76 kcal mol^−1^. Notable, however, is that while product formation to 3a is still highly exergonic (−29.99 kcal mol^−1^), the energetic benefit is approx. 11 kcal mol^−1^ smaller than for the chlorinated derivative 2a.

Last, we sought to rationalize the preferential formation of fluorinated cyclobutene derivatives 4a over the brominated analogue 3a from the reaction between cyclopropene 1a and bromo-fluoro-carbene (Scheme 6c(iii)). To this end, reaction energy profiles for the formation of both products were calculated. In both cases, the reaction begins with the addition of bromo-fluoro-carbene onto the cyclopropene forming 20*via*19 with an activation energy of 16.97 kcal mol^−1^. Also in this case formation of 20 is exergonic (−15.66 kcal mol^−1^). At this point the energetic profile for the formation of the two possible products (3a*vs.*4a) significantly diverges. Intramolecular epoxidation and ring expansion in both cases was found to be the rate-determining step, yet the energetic barrier to reach 22 is significantly higher for the formation of brominated analogue 3a (37.00 kcal mol^−1^) than for the formation of the fluorinated analogue 4a (21: 25.89 kcal mol^−1^), indicating a significant difference in reaction rates at room temperature in favour of the formation of fluorinated spiro[2.3]hexene 4a due to the better leaving group ability of the bromide anion. Product formation is exergonic in both cases, although the fluorinated derivative 4a is significantly more stable (−42.97 kcal mol^−1^) with respect to the brominated analogue 3a (−27.65 kcal mol^−1^). Overall these results indicate that the formation of the fluorinated spiro[2.3]hexene 4a is preferred both kinetically (lower activation energy barrier in the rate-determining step) and thermodynamically (formation of more stable product). This is in line with our experimental observations, in which predominantly the fluorinated products were formed under our reaction conditions, with the brominated analogues being formed only in minor quantities.

## Conclusion

We have developed a general and efficient strategy for the synthesis of 1-oxaspiro[2.3]hex-4-enes *via* a formal single carbon insertion into cyclopropene carbinols, enabled by a skeletal editing approach. This transformation proceeds through dihalocarbene addition and strain-release-driven ring expansion, providing rapid access to halogenated cyclobutene frameworks under operationally simple conditions. The method exhibits broad substrate scope, tolerating a wide range of aryl and alkyl substituents, and can be successfully applied to complex, biologically relevant molecules.

Mechanistic investigations, supported by control experiments and DFT studies, suggest a concerted carbene addition followed by intramolecular epoxidation and halide elimination. The resulting spirocycles combine structural novelty with useful reactivity, offering opportunities for further functionalization and derivatization. Overall, this work expands the toolbox for the construction of strained spirocyclic architectures and opens new avenues for their exploration.

## Author contributions

M. C. C., I. M., E. M., S. S.: investigation, data curation, formal analysis. E. S.: supervision. F. S.: funding acquisition. M. C.: supervision. P. N.: investigation, writing – original draft. N. S. S.: investigation, supervision. R. L.: conceptualization, funding acquisition, project administration, resources, writing – review and editing.

## Conflicts of interest

There are no conflicts to declare.

## Supplementary Material

SC-OLF-D6SC02968J-s001

SC-OLF-D6SC02968J-s002

## Data Availability

CCDC 2527518 (for 2b), 2527435 (for 2d) and 2527436 (for 3d) contain the supplementary crystallographic data for this paper.^55*a*–*c*^ The data supporting this article have been included as part of the supplementary information (SI). Supplementary information is available. See DOI: https://doi.org/10.1039/d6sc02968j.
