# Supplementary material for: Single-carbon insertion enables conversion of cyclopropenes into cyclobutenes: access to oxaspiro[2.3]hexenes
Source: Chem Sci. 2026 Jul 1;17(33):15928–37. doi: 10.1039/d6sc02968j (PMC13348279; doi:10.1039/d6sc02968j)
Supplement: Supplementary file 1 [file SC-017-D6SC02968J-s001.pdf]

Supplementary Information (SI) for Chemical Science.
This journal is © The Royal Society of Chemistry 2026

Single-Carbon Insertion Enables Conversion of Cyclopropenes into Cyclobutenes: Access to Oxaspiro[2.3]hexenes

Supplementary Information

Maria Chiara Cabua,†,[a,b] Iktedar Mahdi,†,[a] Ernesto Mesto,[c] Emanuela Schingaro,[c] Francesco Secci,[b] Sehrish Sarfaraz,[d] Marco Colella,[a] Philipp Natho,[a] Nadeem S. Sheikh,[e] Renzo Luisi*,[a]

a. Department of Pharmacy-Drug Sciences.
University of Bari "A. Moro", Via E. Orabona 4, 70125 Bari, Italy
Renzo.Luisi@uniba.it

b. Dipartimento di Scienze Chimiche e Geologiche
Università degli Studi di Cagliari, S.S. 554, bivio per Sestu, Monserrato (Ca), Italy

c. Department of Earth and Geoenvironmental Sciences
University of Bari "A. Moro", Via E. Orabona 4, 70125 Bari, Italy

d. Department of Chemistry
GGDC Chitti Dheri Mansehra, Higher Education Department KP, 21300, Pakistan

e. Chemical Sciences, Faculty of Science, Universiti Brunei Darussalam
Jalan Tungku Link, Gadong BE1410, Brunei Darussalam

† These authors contributed equally

Table of Contents

1. General Information S3

2. Optimization Studies S5

3. General Procedures S6

3.1. General Procedure SM (GP-SM): Synthesis of tribromocyclopropanes. S6

3.2. General Procedures 1 (GP1): Synthesis of cyclopropenyl carbinols. S7

3.2-A General Procedure 1A (GP1-A): Synthesis of cyclopropenyl carbinols in flow. S7

3.2-B General Procedure 1B (GP1-B): Synthesis of cyclopropenyl carbinols in batch. S8

3.3 General Procedures 2, 3, and 4: Synthesis of halo-oxaspiro[2.3]hexenes. S9

3.3-A General Procedure 2 (GP2): Synthesis of chloro-oxaspiro[2.3]hexenes. S9

3.3-B General Procedure 3 (GP3): Synthesis of bromo-oxaspiro[2.3]hexenes. S10

3.3-C General Procedure 4 (GP4): Synthesis of fluoro-oxaspiro[2.3]hexenes. S11

3.4 General Procedure 5 (GP5): Acid-catalyzed 1,2-migration of epoxide / ring expansion of oxaspiro[2.3]hexenes. S12

4. Synthesis and characterization of compounds S13

5. X-Ray Crystallography S108

6. Computational Calculations S111

7. References S133

8. NMR Spectra S135

1. General Information

Solvents, Reagents & Reactions

Chemical symbols are used with their standard meanings. SI units and corresponding standard abbreviations are applied. Solvent evaporation was carried out using a Büchi R-300 rotary evaporator under reduced pressure (0– 1000 mbar) with a bath temperature maintained between 35 and 40 °C. Reagents were purchased from commercial sources (Merck, BLD, ThermoFisher Scientific, Fluorochem) and used as received, unless otherwise stated. n-Butyllithium solution (2.5 M in hexane) was purchased from Merck and titrated before use. Dry THF was purchased from Merck and used as received.

Chromatography

Thin-layer chromatography (TLC) was performed on aluminum-backed silica gel plates (0.25 mm, pre-coated with fluorescent indicator 60 F254, Merck) and visualized under UV light (λ = 254 nm). Flash column chromatography was carried out using silica gel (40–63 µm, Geduran, Merck) with pressure applied via head bellows. All chromatography solvents were obtained from commercial suppliers and used as received.

Analysis, Spectroscopy and Spectrometry of compounds

Nuclear magnetic resonance (NMR) spectra were acquired on a Bruker Ascend400 spectrometer operating at 400 MHz for ^{1}H NMR, 101 MHz for ^{13}C{1H} NMR, and 377 MHz for ^{19}F NMR. Measurements were conducted at room temperature, with chemical shifts referenced to the residual non-deuterated solvent peak. Chemical shifts (δ) are given in parts per million (ppm), reported to two decimal places for ^{1}H and ^{19}F signals, and to one decimal place for ^{13}C signals. Signal multiplicities are denoted as singlet (s), doublet (d), triplet (t), quartet (q), pentet (p), multiplet (m), broad (br), or appropriate combinations. Coupling constants (*J*) are provided in hertz (Hz), rounded to the nearest 0.1 Hz. Infrared (IR) spectra were obtained using a ThermoScientific Nicolet Summit Pro FTIR spectrometer. Samples were analyzed neat, and characteristic absorption bands (νmax) are reported in wavenumbers (cm^{-1}). High-resolution mass spectrometry (HRMS) analyses were conducted via electrospray ionization (ESI).

Naming of compounds

Compound names were generated using ChemDraw Professional 20.0 (PerkinElmer), in accordance with IUPAC nomenclature rules.

2. Optimization Studies

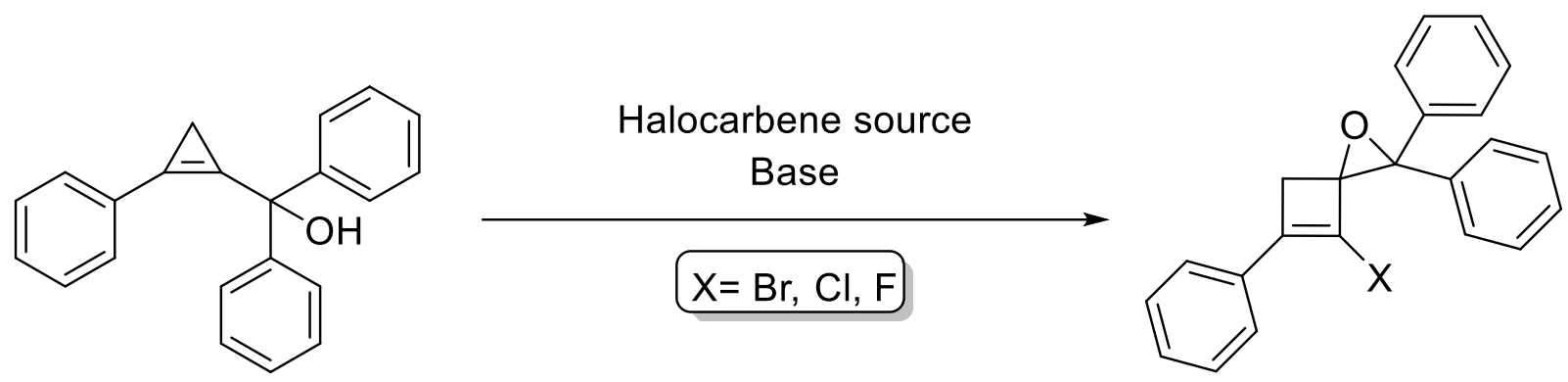

Entry	Carbene source	Equiv. carbene source	Solvent	Base	Equiv. base	Temperature [°C]	Time [hrs]	Quant ^{1}H-NMR yield [%]
1	$CHCl_3$[a]	4.5	Neat	NaOH (aq. 20 M solution)	9	rt	16	97[b]
2	$CHCl_3$[a]	4.5	Neat	NaOH (aq. 20 M solution)	9	40	4	96[b]
3	$CHCl_3$[a]	4.5	Neat	NaOH (aq. 20 M solution)	9	rt	4	95[b]
4	$CHCl_3$[a]	4.5	Neat	NaOH (aq. 10 M solution)	9	rt	4	86[b]
5	$CHCl_3$[a]	4.5	Neat	NaOH (aq. 20 M solution)	4.5	rt	4	87[b]
6	$CHBr_3$[a]	4.5	Toluene	NaOH (aq. 20 M solution)	9	rt	16	55[c]
7	$CHBr_3$[a]	4.5	Neat	NaOH (aq. 20 M solution)	9	rt	16	49[c]
8	$CHBr_3$[a]	4.5	Neat	NaOH (aq. 20 M solution)	9	60	16	90[c]
9	$FSO_2CF_2CO_2H$	1.3	DCM	K_2CO_3	4	0	4	0[b]
10	$FSO_2CF_2CO_2H$	1.3	DCM	NaOH (aq. 20 M solution)	4	0	4	0[b]
11	$FSO_2CF_2CO_2H$	1.3	Toluene	NaOH (aq. 20 M solution)	4	0	4	0[b]
12	$FSO_2CF_2CO_2H$	1.3	Toluene	NaOH (aq. 20 M solution)	4	0 to rt	16	0[b]
13	$(CH_3)_3SiCF_3$[d]	2.5	THF			80	16	0[b]
14	$(CH_3)_3SiCF_3$[d]	5	THF			80	16	0[b]
15	$CHFBr_2$[a]	4.5	Neat	NaOH (aq. 20 M solution)	9	rt	6	82[b]
16	$CHFBr_2$[a]	4.5	Toluene	NaOH (aq. 20 M solution)	9	rt	6	83[b]
17	$CHFBr_2$[a]	2	Toluene	NaOH (aq. 20 M solution)	9	rt	6	83[b]

Table S1. Optimization of reaction conditions

[a]*Benzyltriethylammonium chloride* (0.1 equiv.) has been used as a phase transfer catalyst. [b]Determined by quantitative ^{1}H-NMR using *1,3,5-trimethoxybenzene* as an internal standard. [c]Determined by quantitative ^{1}H-NMR using *dibromomethane* as an internal standard. [d]*Sodium iodide* (0.5 equiv.) has been used as initiator.

3. General Procedures

3.1. General Procedure SM (GP-SM): Synthesis of tribromocyclopropanes.

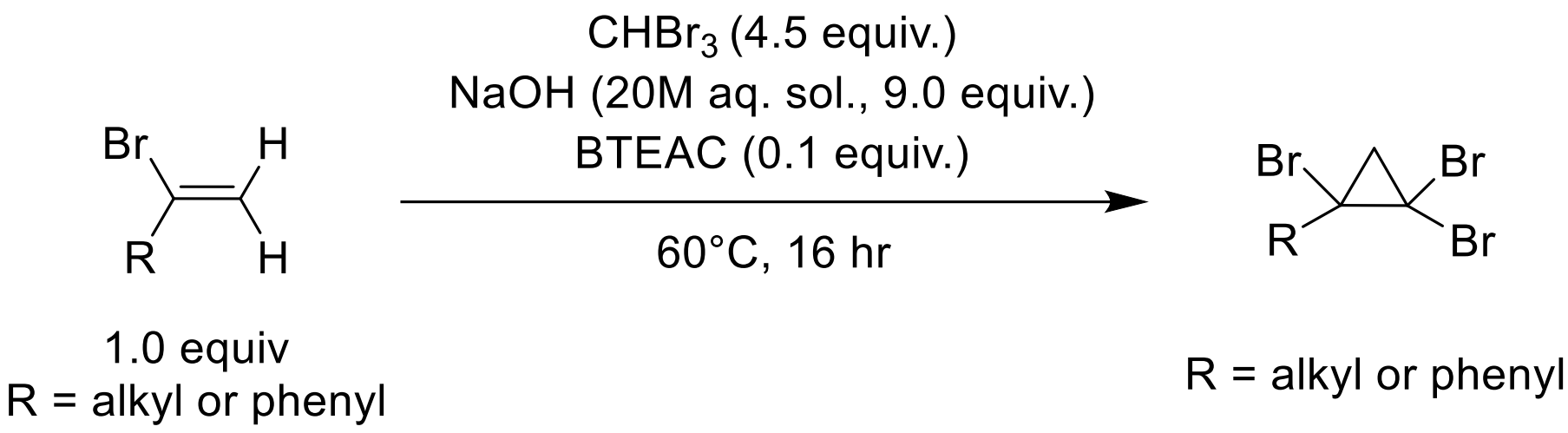

Tribromocyclopropanes were prepared adapting a previously reported procedure.[1]

To a vigorously stirred solution of *bromoalkene* (1.0 equiv.), *benzyltriethylammonium chloride* (0.1 equiv.) and *bromoform* (4.5 equiv.), an *aqueous 20 M NaOH solution* (9.0 equiv.) was added dropwise. The resulting mixture was stirred and heated to 60 °C overnight, before it was cooled to room temperature. It was then diluted with DCM and washed with water twice; the organic phase were dried over anhydrous sodium sulfate and the solvent removed under reduced pressure to afford the crude material, which was purified by column chromatography (hexane) to afford the desired tribromocyclopropanes **SM1-SM5**.

3.2. General Procedures 1 (GP1): Synthesis of cyclopropenyl carbinols.

3.2-A General Procedure 1A (GP1-A): Synthesis of cyclopropenyl carbinols in flow.

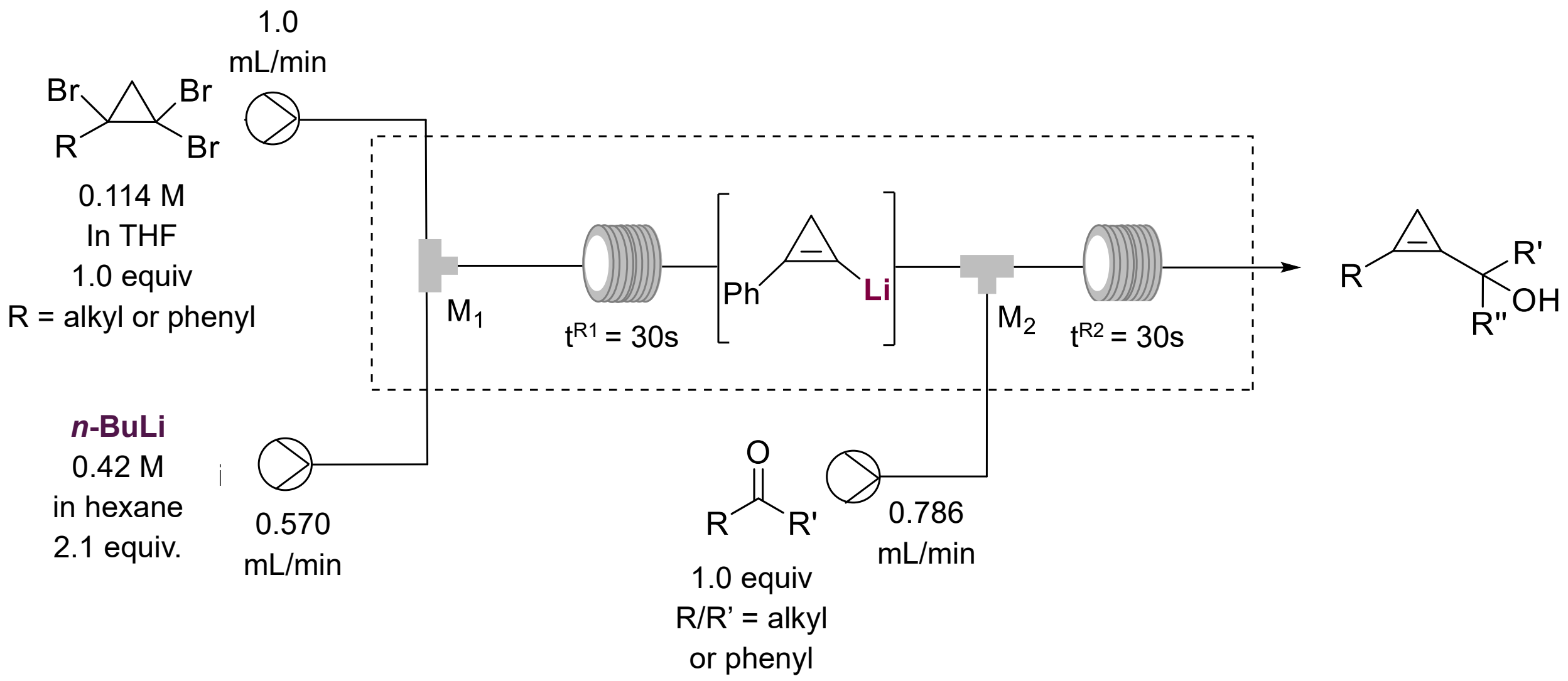

Cyclopropenyl carbinols were prepared in flow following a previously reported procedure.[2]

The reactor was completely immersed into an ice bath (0°C). A solution of the *tribromocyclopropane* (0.114 M in THF) (flow rate: 1 mL/min by Harvard Apparatus PHD ULTRA syringe pump) and a freshly prepared solution of *n-butyllithium* (0.42 M in n-Hexane) (flow rate: 0.57 mL/min by Harvard Apparatus PHD ULTRA syringe pump), were introduced to M1 (inner diameter = 0.5 mm) by syringe pumps. The resulting solution was passed through R1 [$\phi 1$ = 1 mm, L1 = 100 cm (t^{R1} = 30 s)] and mixed in M2 (inner diameter = 0.5 mm) with a solution of *ketone* (0.145 M in THF) (flow rate: 0.786 mL/min by Harvard Apparatus PHD ULTRA syringe pump). The resulting solution was passed through R2 ($\phi 2$ = 1.0 mm, L2 = 150 cm (t^{R2} = 30 s). After reaching the steady state (collection for one minute), the solution was collected for 5 minutes in a separate vial containing an excess of water as the quench. The reaction mixture was extracted with Et_2O (3 x 5 mL). The combined organic phases were dried over sodium sulfate and the solvent filtrated and evaporated under reduced pressure. The residue was purified as described per example to yield the desired products **1a-d**, **1g-i**, **1l**, **1n**, and **1w**.

3.2-B General Procedure 1B (GP1-B): Synthesis of cyclopropenyl carbinols in batch.

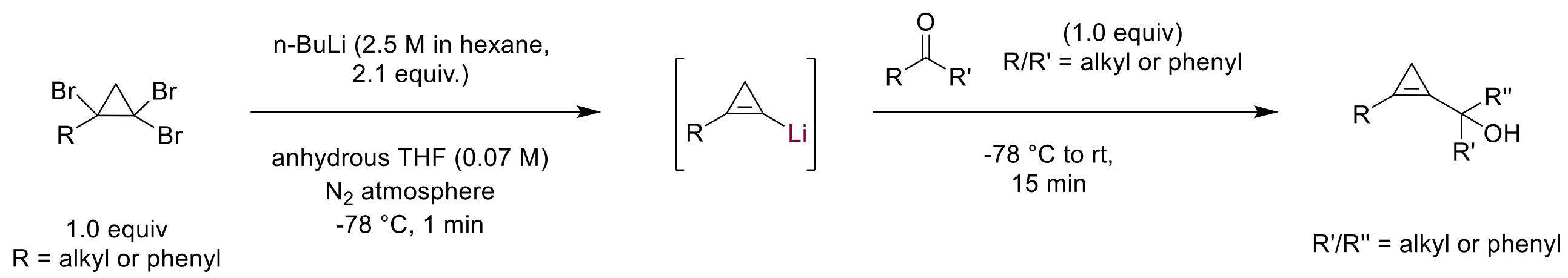

Cyclopropenyl carbinols were prepared in batch adapting a previously reported procedure.[1]

A solution of the *tribromocyclopropane* (0.07 M in anhydrous THF) was cooled down to -78 °C and purged with nitrogen gas (N_2). A *N-butyllithium* solution (2.1 equiv., 2.5 M in hexane) was added and the mixture was allowed to stir for one minute. Then, the so generated cyclopropenyllithium was quenched by adding a *ketone* (1.0 equiv.) and the solution was stirred for 15 minutes, while warming to room temperature. The reaction mixture was quenched with water, extracted with Et_2O three times, and the combined organic phases were dried over sodium sulfate. The solvent was filtrated and evaporated under reduced pressure, and the residue was purified, when needed, as described per example to yield the desired products **1e-f**, **1j-k**, **1m**, and **1o-v**.

3.3 General Procedures 2, 3, and 4: Synthesis of halo-oxaspiro[2.3]hexenes.

3.3-A General Procedure 2 (GP2): Synthesis of chloro-oxaspiro[2.3]hexenes.

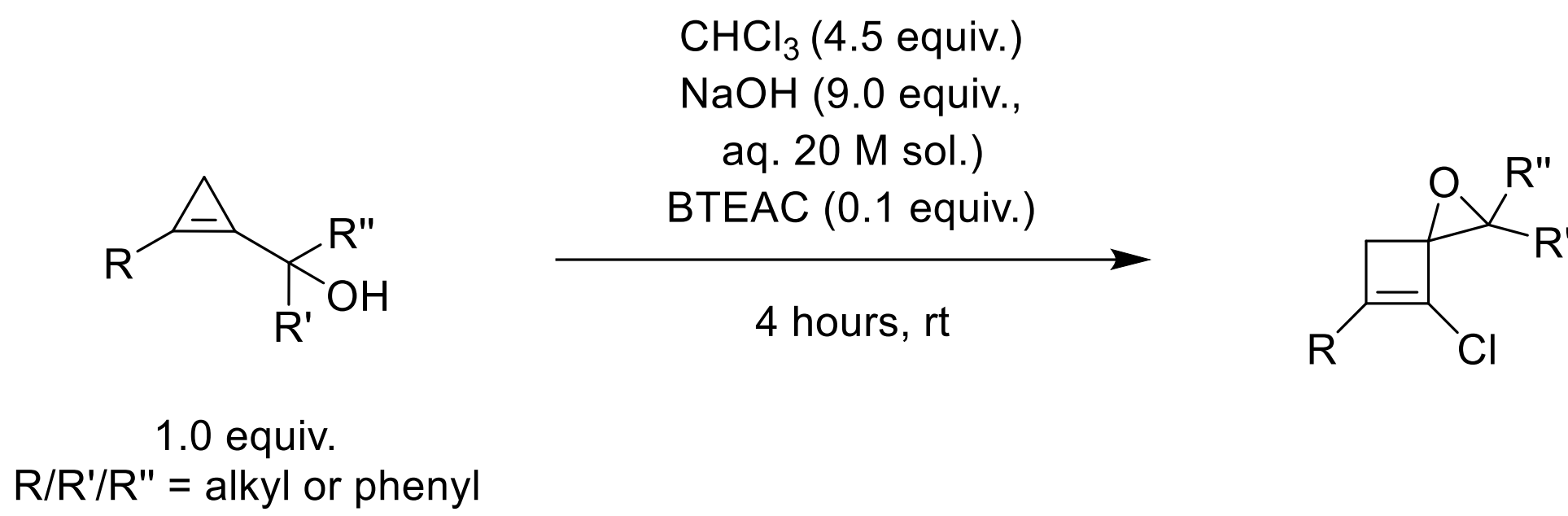

To a vigorously stirred solution of *cyclopropenyl carbinol* (1.0 equiv.), *benzyltriethylammonium chloride* (0.1 equiv.) and *chloroform* (4.5 equiv.), an *aqueous 20 M NaOH solution* (9.0 equiv.) was added dropwise. The resulting mixture was stirred at room temperature for 4 hours. It was then diluted with DCM and washed with water twice; the organic phase was dried over anhydrous sodium sulfate, filtered and the solvent removed under reduced pressure to afford the crude material, which was purified, when necessary, by flash column chromatography, as indicated in each specific case, to yield the desired *chloro-oxaspiro[2.3]hexenes* **2a-2u**.

3.3-B General Procedure 3 (GP3): Synthesis of bromo-oxaspiro[2.3]hexenes.

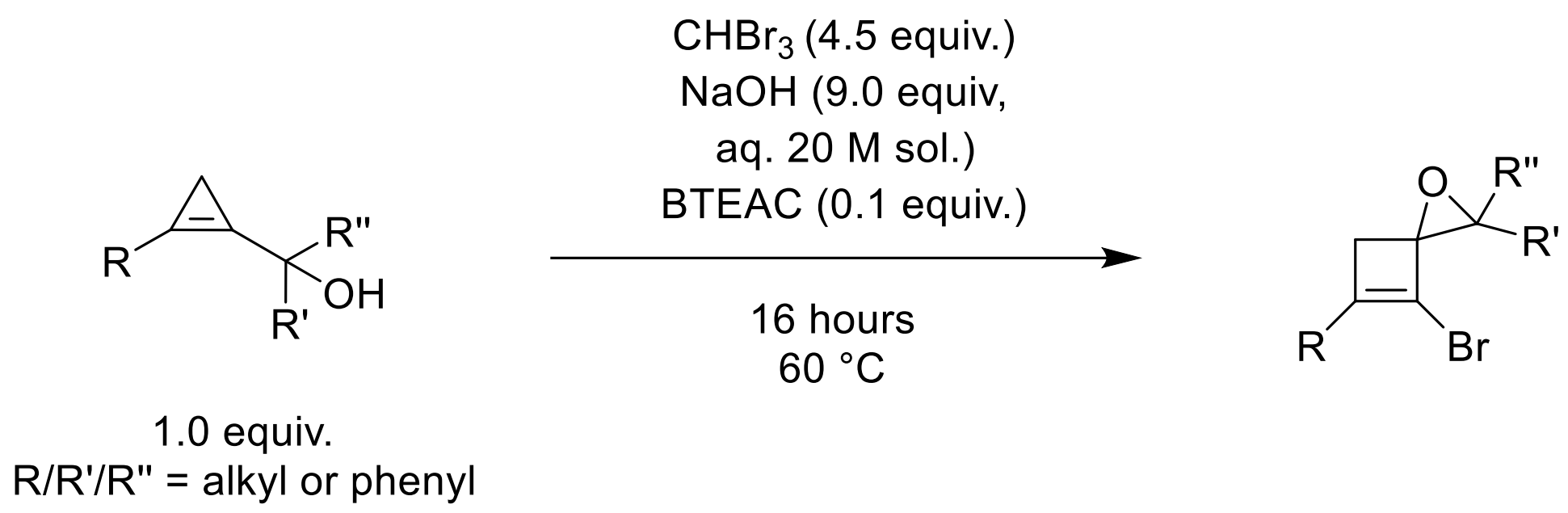

To a vigorously stirred solution of *cyclopropenyl carbinol* (1.0 equiv.), *benzyltriethylammonium chloride* (0.1 equiv.) and *bromoform* (4.5 equiv.), an *aqueous 20 M NaOH solution* (9.0 equiv.) was added dropwise. The resulting mixture was stirred and heated to 60 °C overnight, before it was cooled to room temperature. It was then diluted with DCM and washed with water twice; the organic phase was dried over anhydrous sodium sulfate, filtered and the solvent removed under reduced pressure to afford the crude material, which was purified by flash column chromatography, as indicated in each specific case, to yield the desired *bromo-oxaspiro[2.3]hexenes* **3a-3u**.

3.3-C General Procedure 4 (GP4): Synthesis of fluoro-oxaspiro[2.3]hexenes.

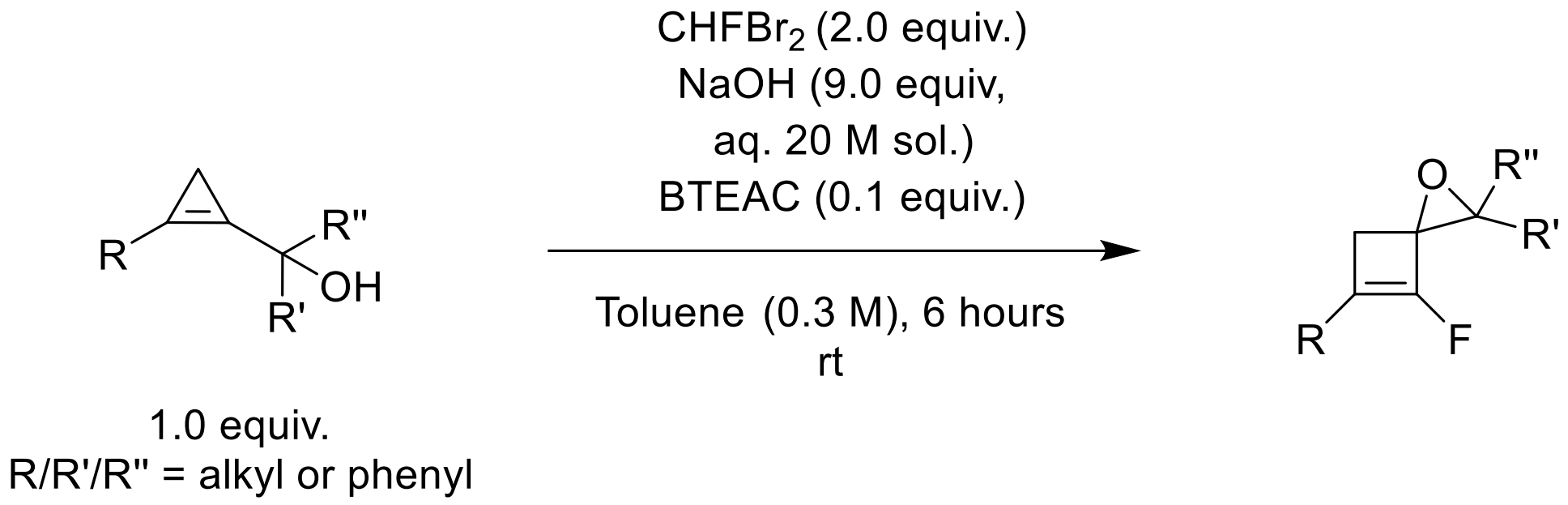

To a vigorously stirred solution of *cyclopropenyl carbinol* (1.0 equiv.), *benzyltriethylammonium chloride* (0.1 equiv.) and *dibromofluoromethane* (2.0 equiv.) in toluene (0.3 M), an *aqueous 20 M NaOH solution* (9.0 equiv.) was added dropwise. The resulting mixture was stirred at room temperature for 6 hours, before it was cooled to room temperature. It was then diluted with DCM and washed with water twice; the organic phase was dried over anhydrous sodium sulfate, filtered and the solvent removed under reduced pressure to afford the crude material, which was purified by flash column chromatography or by precipitation in hexane, as indicated in each specific case, to yield the desired *fluoro-oxaspiro[2.3]hexenes* **4a-4o**.

3.4 General Procedure 5 (GP5): Acid-catalyzed 1,2-migration of epoxide / ring expansion of oxaspiro[2.3]hexenes.

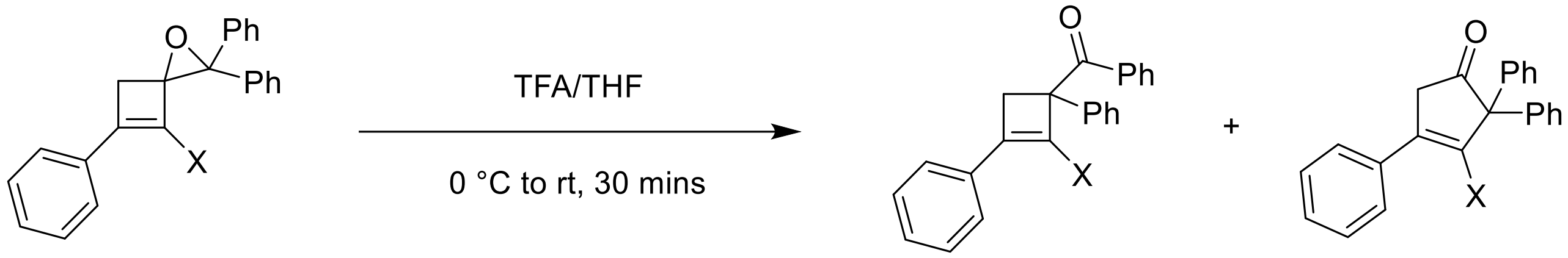

*Products **5** and **6** were prepared following a previously reported procedure.[3]*

To a solution of *halo-oxaspiro[2.3]hexene* in THF (0.4 M) cooled to 0 °C was added *trifluoroacetic acid* (0.4 mL/mmol of *oxaspiro[2.3]hexene*) dropwise. The solution was stirred for thirty minutes while warming to room temperature. Then, it was diluted with Et_2O and quenched by the addition of saturated aqueous sodium bicarbonate solution. The organic phase was separated, washed with saturated aqueous sodium chloride solution, dried over sodium sulfate, filtered, and concentrated under reduced pressure. The acidic treatment of oxaspiro[2.3]hexenes thus performed afforded a 30 : 70 mixture of ring expansion products **5a-c** and 1,2-migration of epoxide derivatives **6a-c** and**;** they could be successfully separated and purified by flash column chromatography, as indicated in each specific case.

*Note: Decreasing the excess of acid catalyst promote the preferential formation of cyclopentenones derivatives **5**; when 1.0 equiv. of TFA were used, a 1:1 ratio was achieved between the two possible products, favouring the formation of expansion derivatives compared to standard conditions.*

4. Synthesis and characterization of compounds

(1,2,2-Tribromocyclopropyl)benzene, SM1

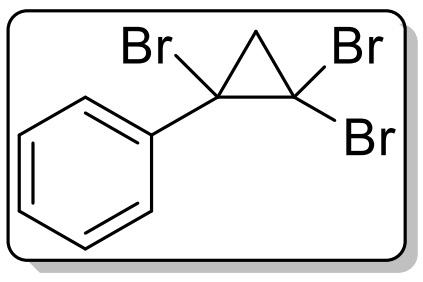

Prepared following **General Procedure SM** using *(1-bromovinyl)benzene* (5.10 g, 28.0 mmol). After flash column chromatography (hexane), the title compound **S1** was obtained as a white solid (8.51 g, 24.0 mmol, 86%).

^{1}H-NMR (400 MHz, $CDCl_3$): δ_H 7.51 – 7.45 (2H, m), 7.41 – 7.31 (3H, m), 2.51 (1H, d, *J* = 9.3 Hz), 2.25 (1H, d, *J* = 9.3 Hz)

The spectroscopic data are in agreement with those previously reported.[1]

1-Methyl-4-(1,2,2-tribromocyclopropyl)benzene, SM2

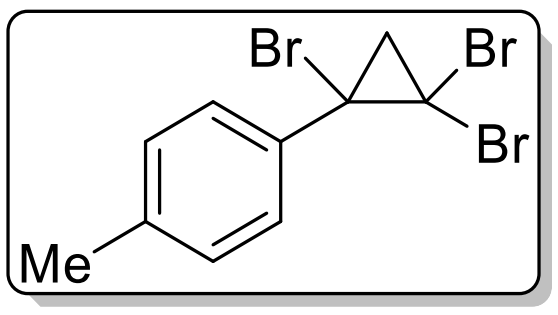

Prepared following **General Procedure SM** using *1-(1-bromovinyl)-4-methylbenzene* (1.44 g, 7.28 mmol). After flash column chromatography (hexane), the title compound **S2** was obtained as a yellow oil (1.40 g, 3.80 mmol, 52%).

^{1}H-NMR (400 MHz, $CDCl_3$): δ_H 7.51 – 7.45 (2H, m), 7.42 – 7.31 (3H, m), 2.49 (d, *J* = 9.3 Hz, 1H), 2.36 (3H, s), 2.25 (d, *J* = 9.3 Hz, 1H).

The spectroscopic data are in agreement with those previously reported.[4]

1-Fluoro-4-(1,2,2-tribromocyclopropyl)benzene, SM3

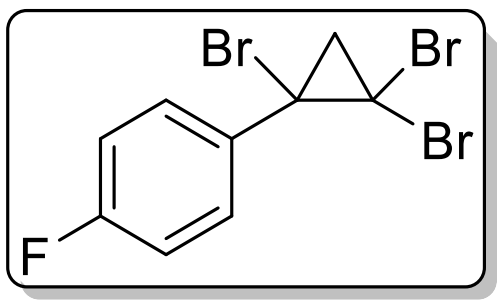

Prepared following **General Procedure SM** using *1-(1-bromovinyl)-4-fluorobenzene* (8.81 g, 43.8 mmol). After flash column chromatography (hexane), the title compound **SM3** was obtained as a white solid (4.70 g, 12.5 mmol, 29%).

^{1}H-NMR (400 MHz, $CDCl_3$): δ_H 7.47 – 7.43 (2H, m), 7.12 – 7.04 (2H, m), 2.47 (1H, d, *J* = 9.4 Hz), 2.25 (1H, *J* = 9.4 Hz)

^{19}F-NMR (377 MHz, $CDCl_3$): δ_F -111.55 (m, *J* = 8.7, 5.1 Hz)

The spectroscopic data are in agreement with those previously reported.[2]

1,1,2-Tribromo-2-isopentylcyclopropane, SM4

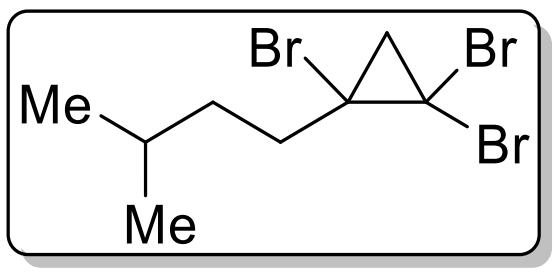

Prepared following **General Procedure SM** using *2-bromo-5-methylhex-1-ene* (2.00 g, 11.3 mmol). After flash column chromatography (hexane), the title compound **SM4** was obtained as a colourless oil (2.70 g, 7.91 mmol, 70%).

^{1}H-NMR (400 MHz, $CDCl_3$): δ_H 2.14 – 2.06 (m, 1H), 2.01 – 1.98 (m, 1H), 1.95 (d, *J* = 9.2 Hz, 1H), 1.83 (d, *J* = 9.2 Hz, 1H), 1.68 – 1.54 (m, 3H), 0.94 (s, 3H), 0.93 (s, 3H).

^{13}C{^{1}H}-NMR (101 MHz, $CDCl_3$): 46.1, 40.0, 38.2, 36.6, 33.3, 28.0, 22.8, 22.6.

IR (neat, ν cm^{-1}): 3445, 3025, 2952, 2866, 1840, 1655, 1599, 1490, 1446, 1319, 1164, 1018, 900

HRMS *mass not found in ESI+ or ESI- mode*

(3-(1,2,2-Tribromocyclopropyl)propyl)benzene, SM5

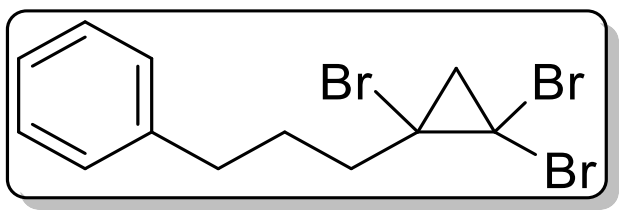

Prepared following **General Procedure SM** using *(4-bromopent-4-en-1-yl)benzene* (2.00 g, 8.88 mmol). After flash column chromatography (hexane), the title compound **SM5** was obtained as a yellow oil (2.30 g, 5.80 mmol, 66%).

^{1}H-NMR (400 MHz, $CDCl_3$): δ_H 7.34 – 7.27 (m, 2H), 7.25 – 7.20 (m, 3H), 2.75 – 2.63 (m, 2H), 2.19 – 2.10 (m, 2H), 2.08 – 2.00 (m, 2H), 1.96 (d, *J* = 9.2 Hz, 1H), 1.82 (d, *J* = 9.2 Hz, 1H)

^{13}C{^{1}H}-NMR (101 MHz, $CDCl_3$): 141.7, 128.5, 126.1, 45.6, 41.4, 38.2, 35.2, 29.4

IR (neat, ν cm^{-1}): 3060, 3025, 2588, 1495, 1452, 1170, 1013, 746, 696.

HRMS *mass not found in ESI+ or ESI- mode*

1,7,7-Trimethylbicyclo[2.2.1]heptan-2-yl 4-benzoylbenzoate, A

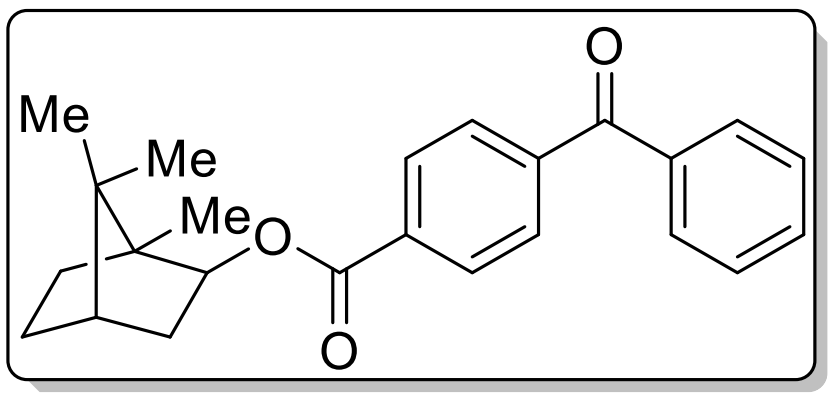

Prepared following a previously reported procedure.[3]

4-Benzoylbenzoic acid (1.00 g, 4.40 mmol, 1.0 equiv.) and (-)-*borneol* (0.682 g, 4.40 mmol, 1.0 equiv.) were dissolved in dichloromethane (0.1 M) and *4-dimethylaminopyridine* (0.433 g, 3.52 mmol, 0.8 equiv.) and *N,N'-dicyclohexylcarbodiimide* (1.00 g, 4.84 mmol, 1.1 equiv.) were added. The resulting solution was allowed to stir overnight at room temperature; it was then diluted with dichloromethane, washed with saturated aqueous ammonium chloride solution twice, saturated aqueous sodium bicarbonate solution twice, and saturated aqueous sodium chloride solution twice. The organic phase was dried over sodium sulfate, filtered, and concentrated under reduced pressure. The crude material was purified by flash column chromatography (hexane to 10% EtOAc/hexane) to yield the compound **B** as a yellowish oil (1.00 g, 2.70 mmol, 62%).

^{1}H-NMR (400 MHz, $CDCl_3$): δ_H 8.17 – 8.13 (2H, m), 7.89 – 7.78 (4H, m), 7.66 – 7.58 (m, 1H), 7.52 – 7.47 (2H, m), 5.16 (1H, ddd, *J* = 9.9, 3.5, 2.1 Hz, 1H), 2.55 – 2.46 (m, 1H), 2.17 – 2.10 (1H, m), 1.87 – 1.80 (1H, m), 1.76 (1H, t, *J* = 4.5 Hz, 1H), 1.48 – 1.39 (1H, m), 1.36 – 1.29 (1H, m), 1.15 (1H, dd, *J* = 13.8, 3.5 Hz, 1H), 0.99 (s, 3H), 0.93 (6H, app. d, *J* = 1.5 Hz).

The spectroscopic data are in agreement with those previously reported.[3]

2-Isopropyl-5-methylcyclohexyl 4-benzoylbenzoate, B

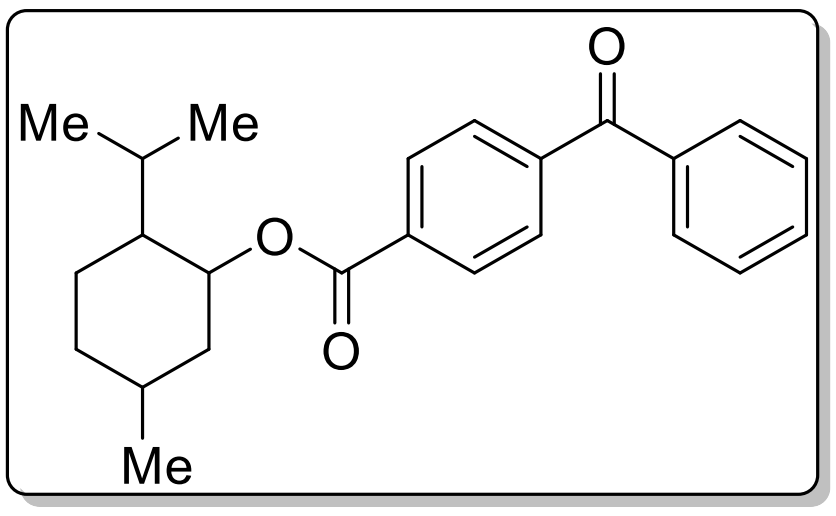

Prepared following a previously reported procedure.[3]

4-Benzoylbenzoic acid (2.00 g, 8.80 mmol, 1.0 equiv.) and *(+/-)-menthol* (1.40 g, 8.80 mmol, 1.0 equiv.) were dissolved in dichloromethane (0.1 M) and *4-dimethylaminopyridine* (0.860 g, 7.04 mmol, 0.8 equiv.) and *N,N'-dicyclohexylcarbodiimide* (2.00 g, 9.68 mmol, 1.1 equiv.) were added. The resulting solution was allowed to stir overnight at room temperature; it was then diluted with dichloromethane, washed with saturated aqueous ammonium chloride solution twice, saturated aqueous sodium bicarbonate solution twice, and saturated aqueous sodium chloride solution twice. The organic phase was dried over sodium sulfate, filtered, and concentrated under reduced pressure. The crude material was purified by flash column chromatography (hexane to 10% EtOAc/hexane) to yield the compound **A** as a colourless oil (1.30 g, 3.60 mmol, 41%).

^{1}H-NMR (400 MHz, $CDCl_3$): δ_H 8.19 – 8.11 (2H, m), 7.85 – 7.80 (4H, m), 7.66 – 7.57 (1H, m), 7.55 – 7.45 (2H, m), 4.98 (1H, td, *J* = 10.9, 4.4 Hz), 2.18 – 2.12 (1H, m), 2.01 – 1.92 (1H, m), 1.80 – 1.70 (2H, m), 1.63 – 1.57 (2H, m), 1.19 – 1.08 (2H, m), 0.95 (3H, d, *J* = 4.2 Hz), 0.93 (3H, d, *J* = 4.7 Hz), 0.82 (3H, d, *J* = 6.9 Hz).

The spectroscopic data are in agreement with those previously reported.[3]

Diphenyl(2-phenylcycloprop-1-en-1-yl)methanol, 1a

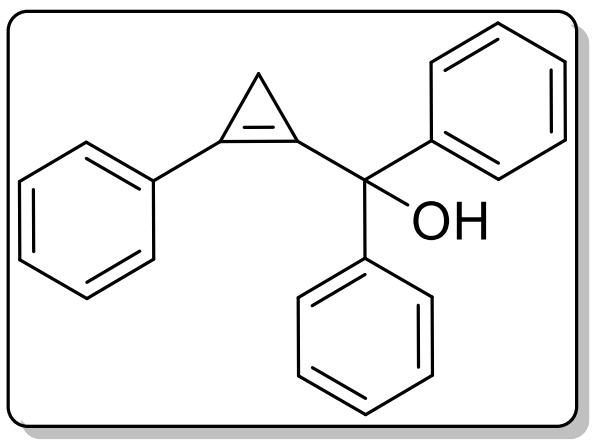

Prepared following **General Procedure 1A** using *(1,2,2-tribromocyclopropyl)benzene* **SM1** (200 mg, 0.570 mmol) and *benzophenone* (103 mg, 0.570 mmol). Quantitative ^{1}H NMR analysis indicated an 80% yield for compound **1a** (136 mg, 0.456 mmol), with 1,3,5-trimethoxybenzene used as an internal standard. Purification by flash column chromatography (hexane to 95:5 hexane:DCM) yielded the title product as a yellow oil.

^{1}H-NMR (400 MHz, $CDCl_3$): δ_H 7.51 – 7.46 (4H, m), 7.40 – 7.25 (11H, m), 2.81 (1H, s), 1.57 (2H, s)

The spectroscopic data are in agreement with those previously reported.[1]

(2-Phenylcycloprop-1-en-1-yl)di-*p*-tolylmethanol, 1b

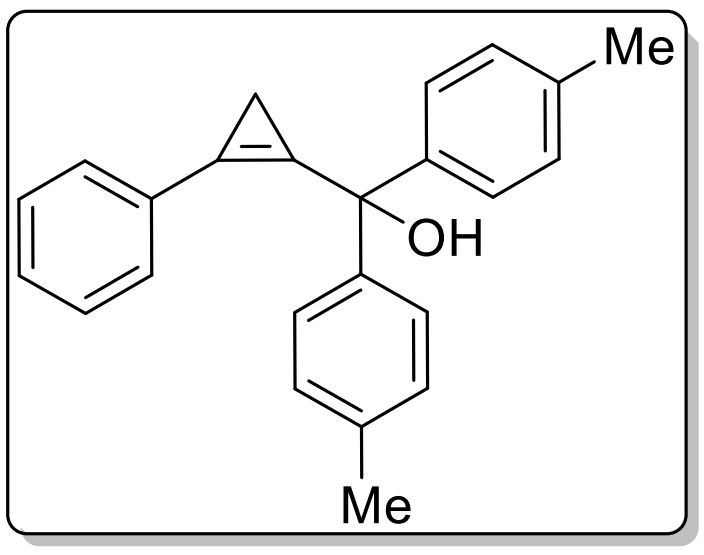

Prepared following **General Procedure 1A** using *(1,2,2-tribromocyclopropyl)benzene* **SM1** (200 mg, 0.570 mmol) and *di-p-tolylmethanone* (120 mg, 0.570 mmol). Quantitative ^{1}H NMR analysis indicated an 68% yield for compound **1b** (126 mg, 0.387 mmol), with 1,3,5-trimethoxybenzene used as an internal standard. Purification by flash column chromatography (hexane to 10:1 hexane:EtOAc) yielded the title product as a pale yellow oil.

^{1}H-NMR (400 MHz, $CDCl_3$): δ_H 7.47 – 7.30 (9H, m), 7.25 – 7.18 (4H, m), 2.86 (1H, s), 2.42 (6H, s), 1.61 (2H, s)

The spectroscopic data are in agreement with those previously reported.[2]

Bis(4-fluorophenyl)(2-phenylcycloprop-1-en-1-yl)methanol, 1c

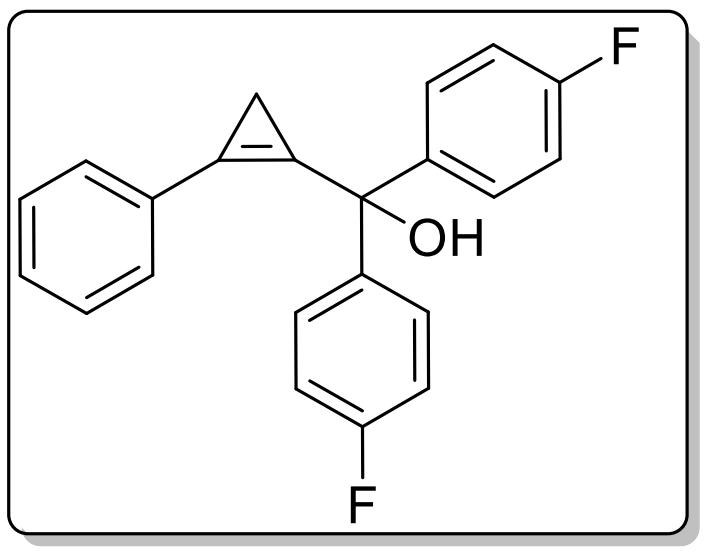

Prepared following **General Procedure 1A** using *(1,2,2-tribromocyclopropyl)benzene* **SM1** (200 mg, 0.570 mmol) and *bis(4-fluorophenyl)methanone* (124 mg, 0.570 mmol). Quantitative ^{1}H NMR analysis indicated an 71% yield for compound **1c** (135 mg, 0.405 mmol), with 1,3,5-trimethoxybenzene used as an internal standard. Purification by flash column chromatography (hexane to 10:1 hexane:EtOAc) yielded the title product as a pale yellow oil.

^{1}H-NMR (400 MHz, $CDCl_3$): δ_H 7.50 – 7.43 (4H, m), 7.41 – 7.30 (5H, m), 7.12 – 7.04 (4H, m), 3.01 (1H, s), 1.60 (2H, s)

^{19}F-NMR (377 MHz, $CDCl_3$): δ_F -114.40 (m)

The spectroscopic data are in agreement with those previously reported.[2]

Bis(4-chlorophenyl)(2-phenylcycloprop-1-en-1-yl)methanol, 1d

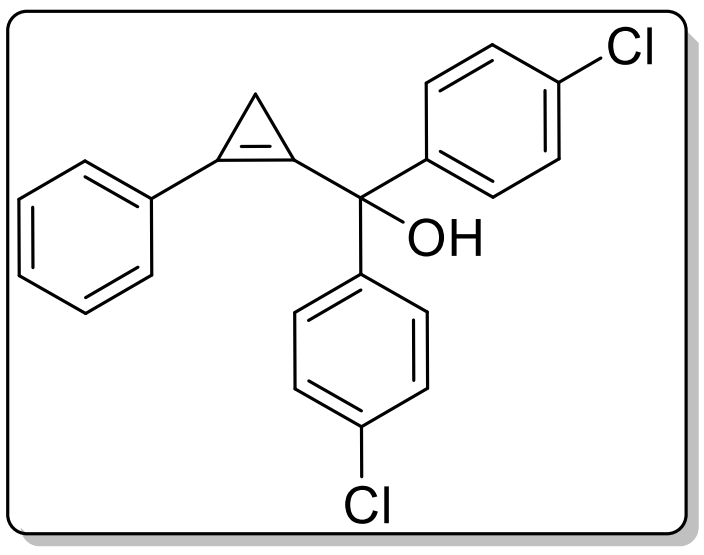

Prepared following **General Procedure 1A** using *(1,2,2-tribromocyclopropyl)benzene* **SM1** (200 mg, 0.570 mmol) and *bis(4-chlorophenyl)methanone* (143 mg, 0.570 mmol). Quantitative ^{1}H NMR analysis indicated an 62% yield for compound **1d** (130 mg, 0.353 mmol), with 1,3,5-trimethoxybenzene used as an internal standard. Purification by flash column chromatography (hexane to 20:1 hexane:EtOAc) yielded the title product as a pale yellow oil.

^{1}H-NMR (400 MHz, $CDCl_3$): δ_H 7.42 – 7.39 (2H, m), 7.39 – 7.37 (3H, m), 7.36 – 7.28 (8H, m), 2.82 (1H, s), 1.54 (2H, s)

The spectroscopic data are in agreement with those previously reported.[2]

(3-Bromophenyl)(phenyl)(2-phenylcycloprop-1-en-1-yl)methanol, 1e

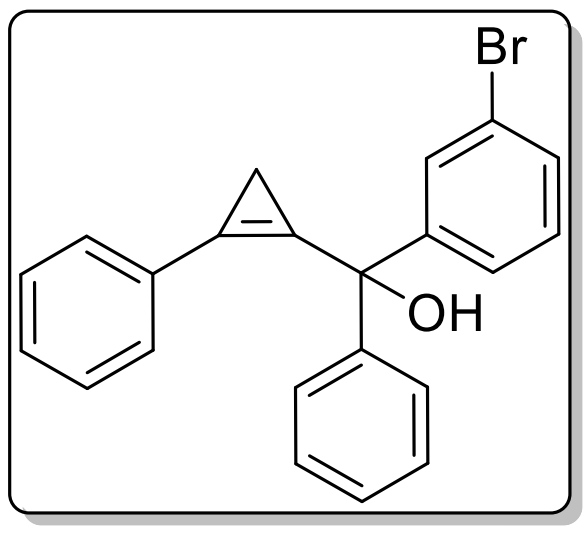

Prepared following **General Procedure 1B** using *(1,2,2-tribromocyclopropyl)benzene* **SM1** (500 mg, 1.41 mmol) and *3-bromobenzophenone* (368 mg, 1.41 mmol). The title compound **1e** was obtained as a yellow oil after work-up without further purification (436 mg, 1.15 mmol, 82%).

^{1}H-NMR (400 MHz, $CDCl_3$): δ_H 7.72 (1H, t, *J* = 1.8 Hz), 7.48 – 7.40 (5H, m), 7.39 – 7.28 (7H, m), 7.23 (1H, t, *J* = 7.9 Hz), 2.87 (s, 1H), 1.60 (1H, d, *J* = 7.2 Hz), 1.57 (1H, d, *J* = 7.2 Hz)

^{13}C{^{1}H}-NMR (101 MHz, $CDCl_3$): δ_C 146.9, 144.1, 130.9, 130.4, 130.0, 129.8, 128.9, 128.7, 128.6, 128.5, 128.1, 126.7, 125.5, 122.7, 116.7, 111.9, 77.8, 9.8

IR (neat, ν cm^{-1}): 3544, 3059, 2958 2870, 1591, 1498, 1446, 1166, 760, 692.

HRMS (ESI+): *m/z* calcd for $C_{22}H_{17}{}^{79}BrONa$ 399.0355 $[M+Na]^+$*;* found 399.0351

Phenyl(2-phenylcycloprop-1-en-1-yl)(4-(*p*-tolylthio)phenyl)methanol, 1f

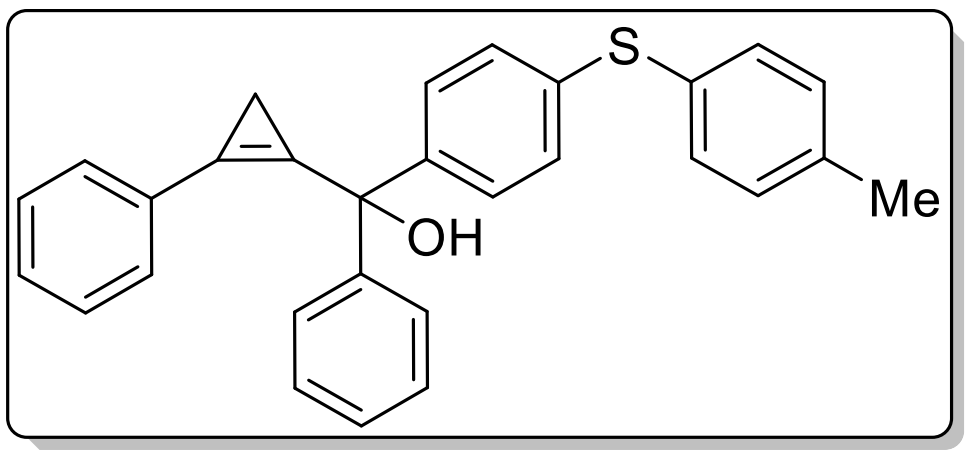

Prepared following **General Procedure 1B** using *(1,2,2-tribromocyclopropyl)benzene* **SM1** (400 mg, 1.13 mmol) and *phenyl(3-(p-tolylthio)phenyl)methanone* (343 mg, 1.13 mmol). The title compound **1f** was obtained as a yellow oil after work-up without further purification (446 mg, 1.06 mmol, 94%).

^{1}H-NMR (400 MHz, $CDCl_3$): δ_H 7.50 – 7.42 (2H, m), 7.41 – 7.25 (12H, m), 7.24 – 7.21 (2H, m), 7.17 – 7.11 (2H, m), 2.79 (1H, s), 2.34 (3H, s), 1.54 (2H, s)

^{13}C{^{1}H}-NMR (101 MHz, $CDCl_3$): δ_C 144.4, 142.9, 138.0, 137.0, 132.7, 131.0, 130.3, 130.3, 129.4, 128.7, 128.6, 128.5, 127.9, 127.6, 126.8, 126.1,117.3, 111.5, 78.0, 21.3, 9.8

IR (neat, v cm^{-1}): 3550, 3449, 3025, 2955, 2688, 1596, 1488, 1396, 1018, 809, 633

HRMS (ESI+): *m/z* calcd for $C_{29}H_{25}OS$ 421.1621 $[M+H]^+$*;* found 421.1633

2,2,2-Trifluoro-1-phenyl-1-(2-phenylcycloprop-1-en-1-yl)ethan-1-ol, 1g

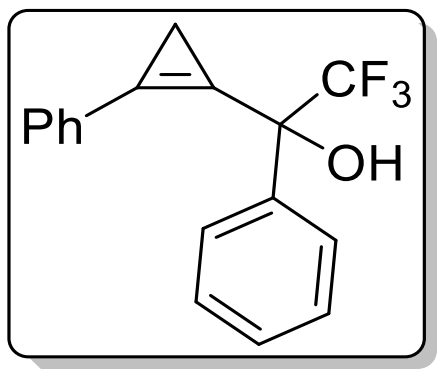

Prepared following **General Procedure 1A** using *(1,2,2-tribromocyclopropyl)benzene* **SM1** (200 mg, 0.570 mmol) and *2,2,2-trifluoro-1-phenylethan-1-one* (100 mg, 0.570 mmol). Quantitative ^{1}H NMR analysis indicated an 76% yield for compound **1g** (125 mg, 0.433 mmol), with 1,3,5-trimethoxybenzene used as an internal standard. Purification by flash column chromatography (hexane to 98:2 hexane:EtOAc) yielded the title product as a yellow oil.

^{1}H-NMR (400 MHz, $CDCl_3$): δ_H 7.68 - 7.61 (2H, m), 7.61- 7.56 (2H, m), 7.47- 7.36 (6H, m), 2.96 (1H, s), 1.56 (1H, d, *J*= 7.5 Hz), 1.51 (1H, d, *J*= 7.5 Hz)

^{19}F-NMR (377 MHz, $CDCl_3$): δ_F -78.53 (s)

The spectroscopic data are in agreement with those previously reported.[2]

2-(2-Phenylcycloprop-1-en-1-yl)propan-2-ol, 1h

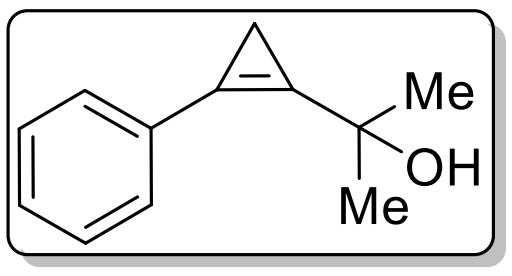

Prepared following **General Procedure 1A** using *(1,2,2-tribromocyclopropyl)benzene* **SM1** (200 mg, 0.570 mmol) and *acetone* (33.1 mg, 0.570 mmol). Quantitative ^{1}H NMR analysis indicated an 80% yield for compound **1h** (79.3 mg, 0.450 mmol), with 1,3,5-trimethoxybenzene used as an internal standard. Purification by flash column chromatography (hexane to 20:1 hexane:EtOAc) yielded the title product as a yellow oil.

^{1}H-NMR (400 MHz, $CDCl_3$): δ_H 7.60 – 7.53 (2H, m), 7.43 – 7.37 (2H, m), 7.34 – 7.27 (1H, m), 1.61 (6H, s), 1.34 (2H, s)

The spectroscopic data are in agreement with those previously reported.[2]

1-(2-Phenylcycloprop-1-en-1-yl)cyclohexan-1-ol, 1i

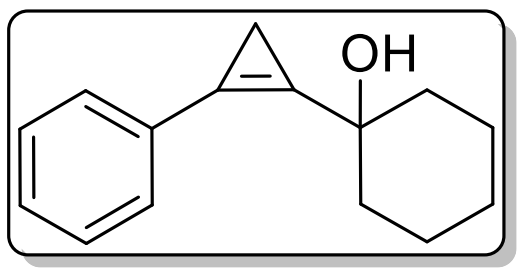

Prepared following **General Procedure 1A** using *(1,2,2-tribromocyclopropyl)benzene* **SM1** (200 mg, 0.570 mmol) and *cyclohexanone* (56.0 mg, 0.570 mmol). Quantitative ^{1}H NMR analysis indicated an 74% yield for compound **1i** (90.3 mg, 0.420 mmol) with 1,3,5-trimethoxybenzene used as an internal standard. Purification by flash column chromatography (hexane to 5:1 hexane:Et_2O) yielded the title product as a yellow oil.

^{1}H-NMR (400 MHz, $CDCl_3$): δ_H 7.62 – 7.56 (2H, m), 7.45 – 7.39 (2H, m), 7.35 – 7.28 (1H, m), 2.08 – 2.00 (2H, m), 1.97 (1H, br s), 1.85 – 1.73 (4H, m), 1.52 – 1.44 (4H, m), 1.32 (2H, s)

The spectroscopic data are in agreement with those previously reported.[2]

tert-Butyl 4-hydroxy-4-(2-phenylcycloprop-1-en-1-yl)piperidine-1-carboxylate, 1j

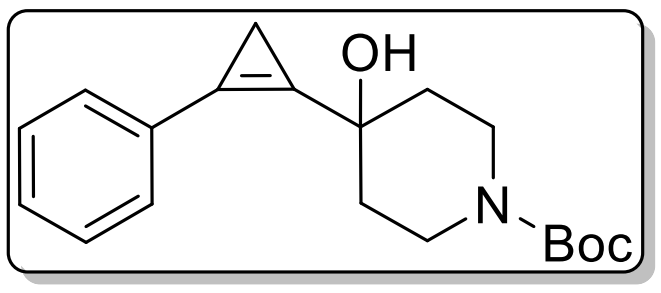

Prepared following **General Procedure 1B** using *(1,2,2-tribromocyclopropyl)benzene* **SM1** (400 mg, 1.13 mmol) and *tert-butyl 4-oxopiperidine-1-carboxylate* (225 mg, 1.13 mmol). Purification by flash column chromatography (10:1 hexane:EtOAc to hexane to 5:1 hexane:EtOAc) yielded the title product **1j** as a yellow oil (282 mg, 0.890 mmol, 79%).

^{1}H-NMR (400 MHz, $CDCl_3$): δ_H 7.55 – 7.48 (2H, m), 7.41 – 7.36 (2H, m), 7.32 – 7.26 (m, 1H), 3.55 – 3.50 (4H, m), 2.51 (1H, bs), 1.99 (2H, m), 1.83 (2H, m), 1.44 (9H, s), 1.30 (2H, s)

^{13}C{^{1}H}-NMR (101 MHz, $CDCl_3$): δ_C 154.9, 129.9, 129.0, 128.7, 128.6, 116.8, 110.4, 79.7, 69.0, 40.1, 36.5, 28.5, 6.9

IR (neat, v cm^{-1}): 3421, 2973, 2869, 1666, 1489, 1366, 1241, 1150, 1019, 760

HRMS (ESI-) m/z calcd for $C_{19}H_{24}NO_3$ $[M-H]^-$ 314.1762; found 314.1743

4-Phenyl-1-(2-phenylcycloprop-1-en-1-yl)cyclohexan-1-ol, 1k

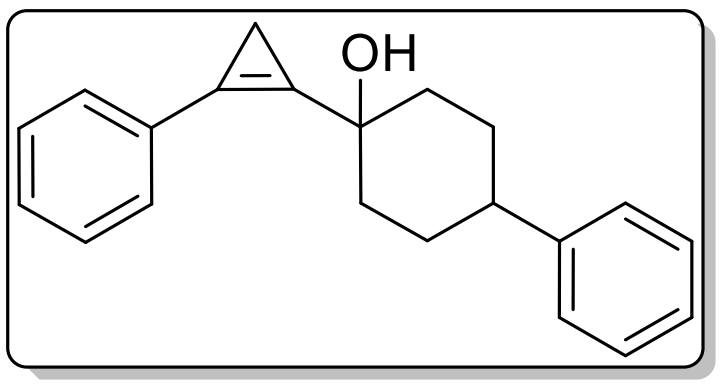

Prepared following **General Procedure 1B** using *(1,2,2-tribromocyclopropyl)benzene* **SM1** (400 mg, 1.13 mmol) and *4-phenylcyclohexan-1-one* (197 mg, 1.13 mmol). Purification by flash column chromatography (hexane to 5:1 hexane:Et_2O) yielded the title product **1k** as a yellow oil (270 mg, 0.933 mmol, 82%).

^{1}H-NMR (400 MHz, $CDCl_3$): δ_H 7.69 – 7.58 (2H, m), 7.50 – 7.42 (2H, m), 7.40 – 7.34 (1H, m), 7.30 – 7.25 (2H, m), 7.22 – 7.14 (3H, m), 2.64 (1H, tt, *J* = 12.4, 3.4 Hz), 2.45 – 2.39 (2H, m), 2.24 (1H, s), 2.02 - 1.92 (4H, m), 1.75 – 1.60 (2H, m), 1.43 (s, 2H)

^{13}C{^{1}H}-NMR (101 MHz, $CDCl_3$): δ_C 146.4, 130.1, 129.3, 128.8, 128.5, 128.4, 126.9, 126.2, 116.7, 110.7, 70.8, 43.4, 38.7, 31.5, 6.6

IR (neat, ν cm^{-1}): 3354, 3025, 2929, 2864, 1491, 1447, 1341, 1017, 906, 757

HRMS (ESI-): *m/z* calcd for $C_{21}H_{21}O$ 289.1598 $[M-H]^-$*;* found 289.1581

Diphenyl(2-(*p*-tolyl)cycloprop-1-en-1-yl)methanol, 1l

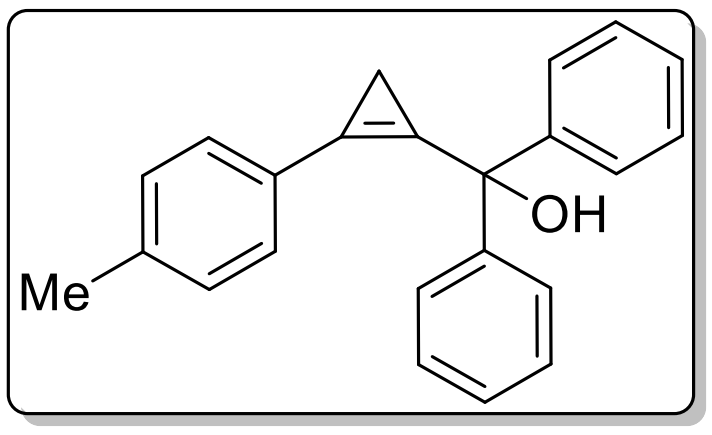

Prepared following **General Procedure 1A** using *1-methyl-4-(1,2,2-tribromocyclopropyl)benzene* **SM2** (200 mg, 0.570 mmol) and *benzophenone* (103 mg, 0.570 mmol). Quantitative ^{1}H NMR analysis indicated an 67% yield for compound **1l** (119 mg, 0.382 mmol), with 1,3,5-trimethoxybenzene used as an internal standard. Purification by flash column chromatography (hexane to 10:1 hexane:EtOAc) yielded the title product as a pale yellow oil.

^{1}H-NMR (400 MHz, $CDCl_3$): δ_H 7.50 – 7.39 (4H, m), 7.38-7.32 (6H, m), 7.18 – 7.12 (4H, m), 2.84 (1H, s), 2.36 (3H, s), 1.56 (2H, s)

The spectroscopic data are in agreement with those previously reported.[2]

Bis(4-chlorophenyl)(2-(*p*-tolyl)cycloprop-1-en-1-yl)methanol, 1m

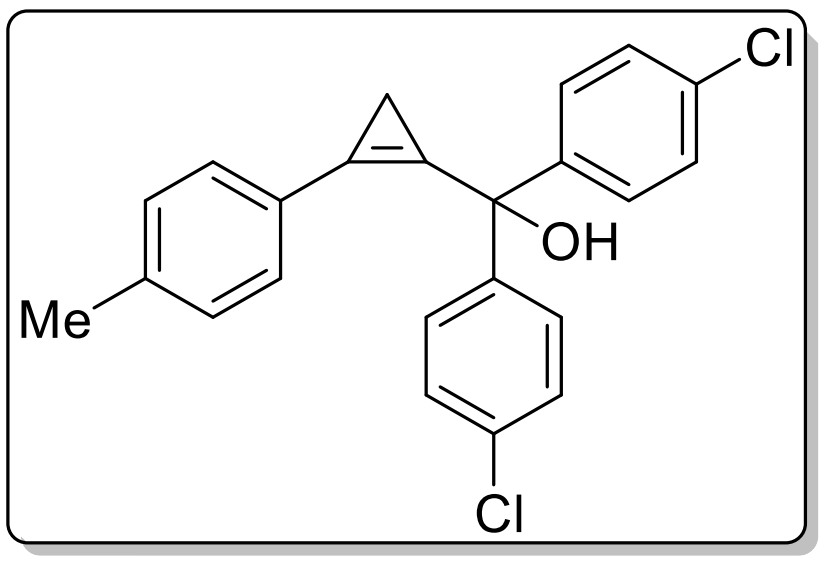

Prepared following **General Procedure 1B** using *diphenyl(2-(p-tolyl)cycloprop-1-en-1-yl)methanol* **SM2** (400 mg, 1.10 mmol) and *bis(4-chlorophenyl)methanone* (276 mg, 1.10 mmol). The title compound **1m** was obtained as a yellow oil after work-up without further purification (323 mg, 0.850 mmol, 77%).

^{1}H-NMR (400 MHz, $CDCl_3$): δ_H 7.42 – 7.35 (4H, m), 7.36 – 7.28 (4H, m), 7.19 – 7.13 (4H, m), 2.79 (1H, s), 2.36 (3H, s), 1.51 (2H, s)

^{13}C{^{1}H}-NMR (101 MHz, $CDCl_3$): δ_C 143.0, 139.3, 133.9, 130.3, 129.5, 128.7, 128.2, 125.6, 114.9, 112.1, 77.4, 21.6, 9.6

IR (neat, ν cm^{-1}): 3444, 2952, 2868, 1654, 1591, 1508., 1488, 1399, 1092, 818

HRMS (ESI+): *m/z* calcd for $C_{23}H_{18}{}^{35}Cl_2ONa$ 403.0633 $[M+Na]^+$*;* found 403.0646

(2-(4-Fluorophenyl)cycloprop-1-en-1-yl)diphenylmethanol, 1n

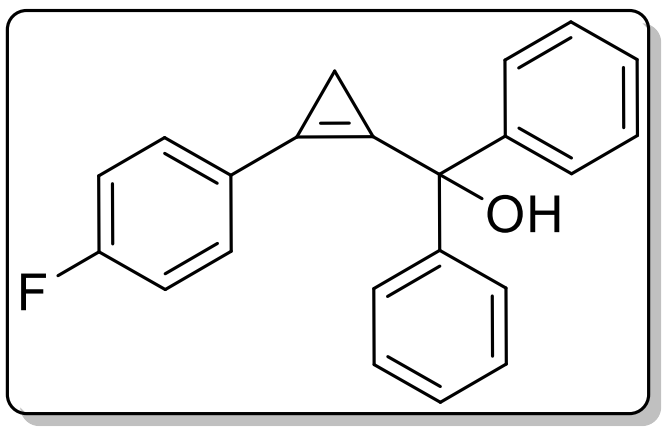

Prepared following **General Procedure 1A** using *1-fluoro-4-(1,2,2-tribromocyclopropyl)benzene* **SM3** (200 mg, 0.570 mmol) and *benzophenone* (103 mg, 0.570 mmol). Quantitative ^{1}H NMR analysis indicated an 72% yield for compound **1n** (130 mg, 0.410 mmol), with 1,3,5-trimethoxybenzene used as an internal standard. Purification by flash column chromatography (hexane to 95:5 hexane:EtOAc) yielded the title product as a yellow oil.

^{1}H-NMR (400 MHz, $CDCl_3$): δ_H 7.49-7.43 (4H, m), 7.39-7.28 (6H, m), 7.23-7.18 (2H, m), 7.02-6.95 (2H, m), 2.83 (1H, s), 1.57 (2H, s)

^{19}F-NMR (377 MHz, $CDCl_3$): δ_F -111.94 (m)

The spectroscopic data are in agreement with those previously reported.[2]

(2-Isopentylcycloprop-1-en-1-yl)diphenylmethanol, 1o

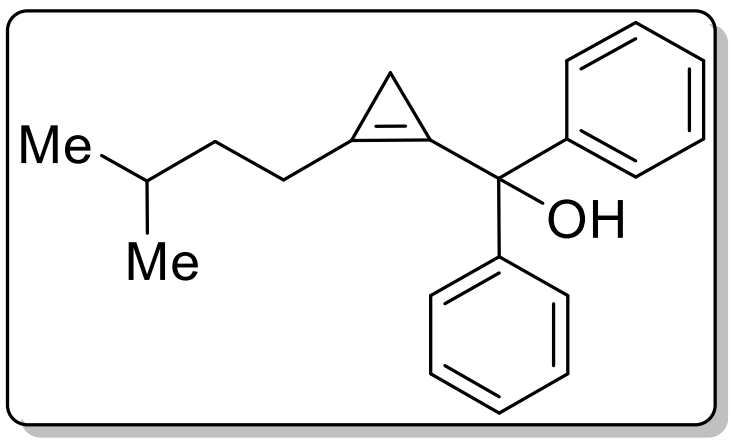

Prepared following **General Procedure 1B** using *1,1,2-tribromo-2-isopentylcyclopropane* **SM4** (400 mg, 1.15 mmol) and *benzophenone* (209 mg, 1.15 mmol). Purification of the crude material by flash column chromatography (hexane to 5:1 hexane:Et_2O) yielded the title product **1o** as a yellow oil (258 mg, 0.884 mmol, 76%).

^{1}H-NMR (400 MHz, $CDCl_3$): δ_H 7.44 – 7.39 (4H, m), 7.35 – 7.29 (4H, m), 7.28 – 7.21 (2H, m), 2.62 (1H, s), 2.46 – 2.40 (2H, m), 1.53 – 1.46 (1H, m), 1.39 – 1.33 (2H, m), 1.17 (2H, s), 0.85 (3H, s), 0.84 (3H, s)

^{13}C{^{1}H}-NMR (101 MHz, $CDCl_3$): δ_C 145.1, 128.3, 127.5, 126.5, 126.2, 114.1, 113.3, 36.1, 27.8, 23.4, 22.4, 9.5

IR (neat, ν cm^{-1}): 3564, 3455, 2954, 2930, 2868, 1621, 1447, 1016, 753, 698

HRMS (ESI+): *m/z* calcd for $C_{21}H_{24}ONa$ 315.1719 $[M+Na]^+$*;* found 315.1722

2-(2-Isopentylcycloprop-1-en-1-yl)propan-2-ol, 1p

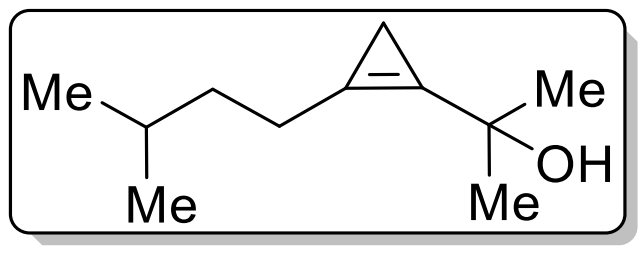

Prepared following **General Procedure 1B** using *1,1,2-tribromo-2-isopentylcyclopropane* **SM4** (346 mg, 1.00 mmol) and *acetone* (58 mg, 1.00 mmol). Purification by flash column chromatography (hexane to 5:1 hexane:Et_2O) yielded the title product **1p** as a colourless oil (106 mg, 0.631 mmol, 63%).

^{1}H-NMR (400 MHz, $CDCl_3$): δ_H 2.47 – 2.42 (2H, m), 1.75 (1H, s), 1.62 – 1.46 (3H, m), 1.44 (6H, s), 0.94 (2H, s), 0.91 (3H, s), 0.90 (3H, s)

^{13}C{^{1}H}-NMR (101 MHz, $CDCl_3$): δ_C 114.0, 110.5, 68.9, 36.6, 28.6, 27.8, 23.7, 22.5, 7.6

IR (neat, ν cm^{-1}): 3366, 2956, 2929, 2868, 1870, 1467, 1366, 1166, 947

HRMS *mass not found in ESI+ or ESI- mode*

Diphenyl(2-(3-phenylpropyl)cycloprop-1-en-1-yl)methanol, 1q

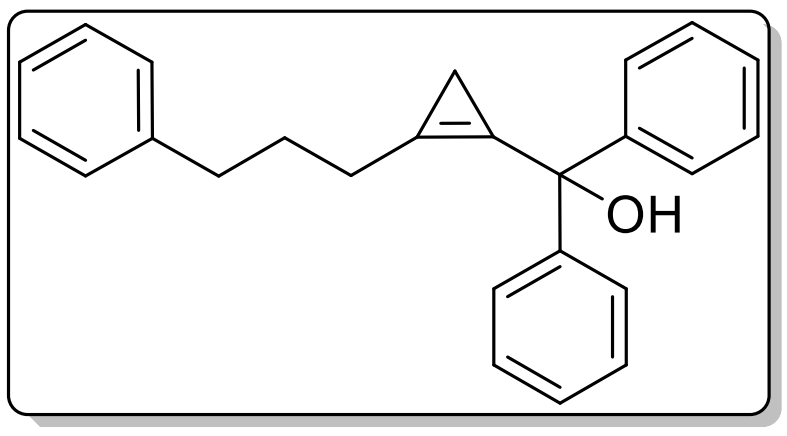

Prepared following **General Procedure 1B** using *(4-bromopent-4-en-1-yl)benzene* **SM5** (400 mg, 1.00 mmol) and *benzophenone* (182 mg, 1.00 mmol). Purification by flash column chromatography (hexane to 5:1 hexane:Et_2O) yielded the title product **1q** as a colourless oil (298 mg, 0.878 mmol, 87%).

^{1}H-NMR (400 MHz, $CDCl_3$): δ_H 7.52 – 7.42 (4H, m), 7.41 – 7.33 (4H, m), 7.33 – 7.26 (4H, m), 7.25 – 7.19 (1H, m), 7.17 – 7.12 (2H, m), 2.67 (1H, s), 2.64 – 2.59 (2H, m), 2.52 (2H, t, *J* = 7.2 Hz), 1.86 (2H, p, *J* = 7.4 Hz), 1.25 (2H, s)

^{13}C{^{1}H}-NMR (101 MHz, $CDCl_3$): δ_C 145.0, 141.9, 128.6, 128.4, 128.3, 127.5, 126.5, 125.9, 113.9, 113.4, 35.5, 28.8, 24.9, 9.7

IR (neat, ν cm^{-1}): 3546, 3453, 3059, 2937, 2863, 1600, 1491, 1447, 1331, 1012, 695

HRMS (ESI+): *m/z* calcd for $C_{25}H_{24}ONa$ 363.1719 $[M+Na]^+$*;* found 363.1726

2-(2-(3-Phenylpropyl)cycloprop-1-en-1-yl)propan-2-ol, 1r

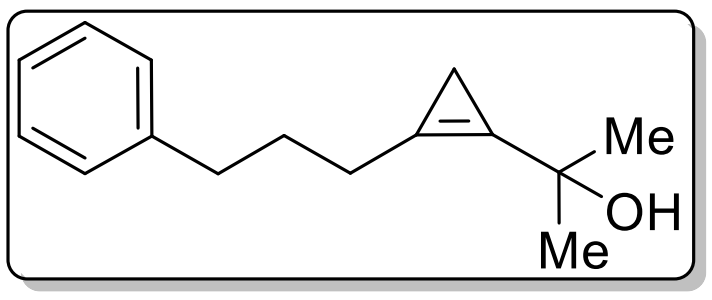

Prepared following **General Procedure 1B** using *(4-bromopent-4-en-1-yl)benzene* **SM5** (400 mg, 1.00 mmol) and *acetone* (58.1 mg, 1.00 mmol). Purification by flash column chromatography (hexane to 5:1 hexane:Et_2O) yielded the title product **1r** as a yellow oil (132 mg, 0.610 mmol, 61%).

^{1}H-NMR (400 MHz, $CDCl_3$): δ_H 7.31 – 7.27 (2H, m), 7.22 – 7.16 (3H, m), 2.69 – 2.64 (2H, m), 2.48 (2H, t, *J* = 7.2 Hz), 1.92 (2H, p, *J* = 7.3 Hz), 1.46 (6H, s), 1.34 (1H, s), 0.97 (2H, s)

^{13}C{^{1}H}-NMR (101 MHz, $CDCl_3$): δ_C 142.1, 128.6, 128.5, 126.0, 114.7, 110.0, 69.0, 35.6, 29.3, 28.5, 25.2, 7.7

IR (neat, v cm^{-1}): 3363, 2973, 2932, 2862, 1679, 1496, 1365, 1155, 744

HRMS *mass not found in ESI+ or ESI- mode*

tert-Butyl 3-hydroxy-3-(2-phenylcycloprop-1-en-1-yl)-8-azabicyclo[3.2.1]octane-8-carboxylate, 1s

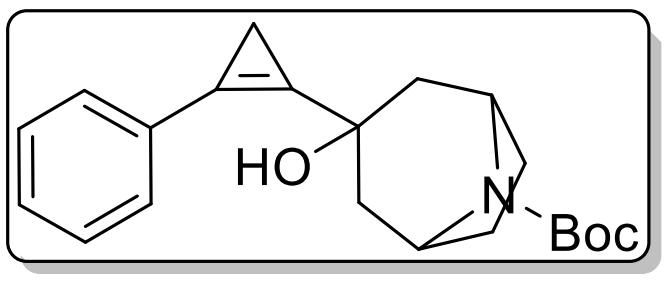

Prepared following **General Procedure 1B** using *(1,2,2-tribromocyclopropyl)benzene* **SM1** (400 mg, 1.13 mmol) and *N-Boc-Nortropinone* (255 mg, 1.13 mmol). The title compound **1s** was obtained as a yellow oil after work-up without further purification (320 mg, 0.941 mmol, 83%).

^{1}H-NMR (400 MHz, $CDCl_3$): δ_H 7.56 – 7.49 (2H, m), 7.44 – 7.34 (2H, m), 7.32 – 7.27 (1H, m), 4.35 – 4.26 (2H, m), 2.48 – 2.24 (1H, m), 2.38 – 2.22 (3H, m), 2.02 – 1.94 (2H, m), 1.91 (1H, s), 1.89 – 1.81 (2H, m), 1.47 (9H, s), 1.30 (2H, s)

^{13}C{^{1}H}-NMR (101 MHz, $CDCl_3$): δ_C 153.8, 129.9, 129.0, 128.7, 128.5, 119.4, 108.9, 79.5, 71.1, 53.3, 40.8, 28.7, 25.7, 7.9

IR (neat, v cm^{-1}): 3248, 2973, 2867, 1693, 1670, 1391, 1167, 1093, 759

HRMS (ESI-): *m/z* calcd for $C_{21}H_{26}NO_3$ $[M-H]^-$ 340.1918; found 340.1903

*Note: Due to the low intensity of some signals resulting from long relaxation times, a copy of the HSQC spectrum has been included in **Section 8** to support their chemical shift.*

2-Phenyl-4-(2-phenylcycloprop-1-en-1-yl)chroman-4-ol, 1t

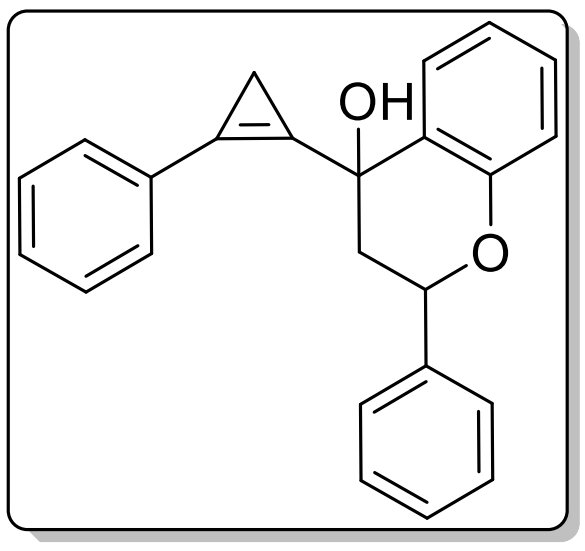

Prepared following **General Procedure 1B** using *(1,2,2-tribromocyclopropyl)benzene* **SM1** (400 mg, 1.13 mmol) and *2-phenylchroman-4-one* (253 mg, 1.13 mmol). Purification by flash column chromatography (5:1 hexane:EtOAc) yielded the title product **1t** as a colourless oil (305 mg, 0.892 mmol, 79%).

^{1}H-NMR (400 MHz, $CDCl_3$): δ_H 7.65 (1H, dd, *J* = 7.8, 1.7 Hz), 7.50 – 7.38 (5H, m), 7.38 – 7.27 (6H, m), 7.09 – 6.95 (2H, m), 5.19 (1H, dd, *J* = 12.0, 2.1 Hz), 2.74 – 2.65 (2H, m), 2.58 (1H, dd, *J* = 13.3, 12.0 Hz), 1.54 (d, *J* = 7.3 Hz, 1H) and 1.51 (d, *J* = 7.3 Hz, 1H) (*AB syst.*)

^{13}C{^{1}H}-NMR (101 MHz, $CDCl_3$): δ_C 154.2, 140.4, 130.1, 129.7, 128.8, 128.8, 128.7, 128.6, 128.4, 127.3, 126.4, 126.4, 121.4, 117.2, 116.7, 111.6, 76.8, 69.3, 43.1, 8.0

IR (neat, ν cm^{-1}): 3347, 3061, 2959, 2870, 1608, 1581, 1483, 1233, 1018, 693

HRMS (ESI-): *m/z* calcd for $C_{24}H_{19}O_2$ $[M-H]^-$ 339.1391; found 339.1373

1,7,7-Trimethylbicyclo[2.2.1]heptan-2-yl 4-(hydroxy(phenyl)(2-phenylcycloprop-1-en-1-yl)methyl)benzoate, 1u

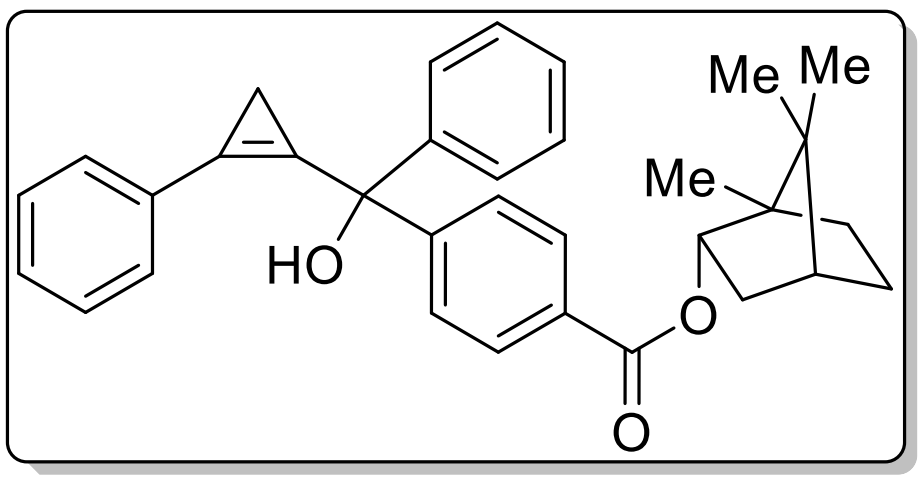

Prepared following **General Procedure 1B** using *(1,2,2-tribromocyclopropyl)benzene* **SM1** (400 mg, 1.13 mmol) and *1,7,7-trimethylbicyclo[2.2.1]heptan-2-yl 4-benzoylbenzoate* **A** (410 mg, 1.13 mmol). Purification by flash column chromatography (hexane to 10:1 hexane:Et_2O) yielded the title product **1v** as a white solid (240 mg, 0.503 mmol, dr > 95:5, 45%).

^{1}H-NMR (400 MHz, $CDCl_3$): δ_H 8.09 – 8.01 (2H, m), 7.64 – 7.56 (2H, m), 7.49 – 7.42 (2H, m), 7.39 – 7.27 (8H, m), 5.14 – 5.09 (1H, m), 2.86 (1H, s), 2.53 – 2.41 (1H, m), 2.11 (1H, ddd, *J* = 13.3, 9.4, 4.4 Hz), 1.83 – 1.75 (1H, m), 1.73 (1H, t, *J* = 4.5 Hz), 1.59 (1H, d, *J* = 7.2 Hz) and 1.56 (1H, d, *J* = 7.7 Hz) (*AB syst, overlapping with the residual water peak*), 1.43 – 1.35 (1H, m), 1.33 – 1.26 (1H, m), 1.11 (1H dd, *J* = 13.8, 3.5 Hz), 0.97 (3H, s), 0.91 (3H, s), 0.90 (3H, d, *J* = 1.3 Hz)

^{13}C{^{1}H}-NMR (101 MHz, $CDCl_3$): δ_C 166.7, 149.3, 144.3, 130.4, 130.3, 129.7, 128.9, 128.7, 128.7, 128.5, 128.1, 126.8, 116.8, 112.0, 80.7, 78.2, 49.3, 48.0, 45.2, 37.1, 28.2, 27.5, 19.9, 19.1, 13.7, 9.8

IR (neat, ν cm^{-1}): 3455, 2953, 2871, 1697, 1447, 1282, 1118, 1019, 759

HRMS (ESI-): *m/z* calcd for $C_{33}H_{33}O_3$ $[M-H]^-$ 477.2435; found 477.2425

2-Isopropyl-5-methylcyclohexyl 4-(hydroxy(phenyl)(2-phenylcycloprop-1-en-1-yl)methyl)benzoate, 1v

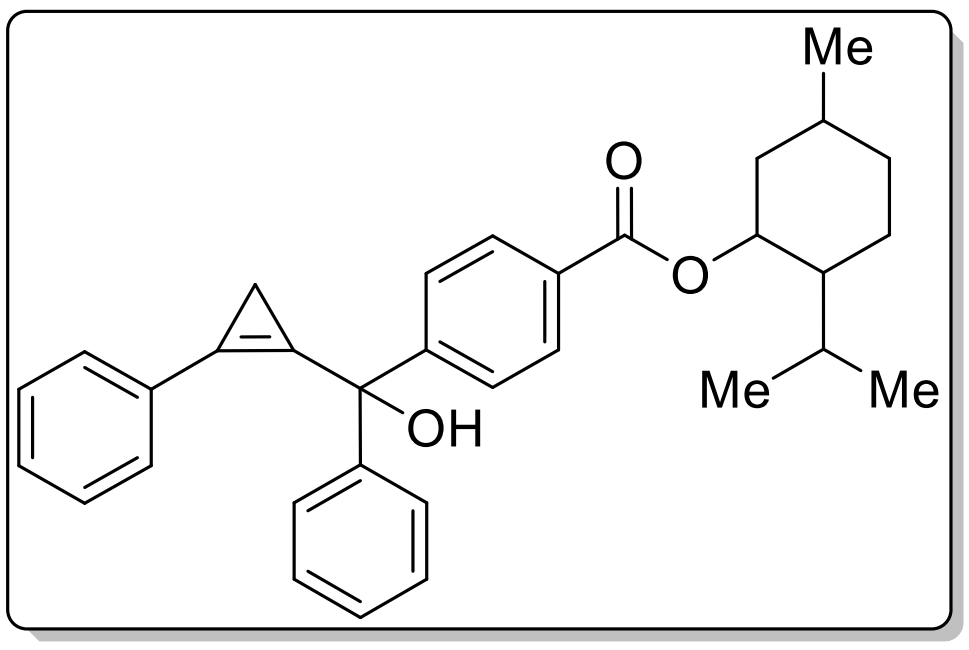

Prepared following **General Procedure 1B** using *(1,2,2-tribromocyclopropyl)benzene* **SM1** (400 mg, 1.13 mmol) and *2-isopropyl-5-methylcyclohexyl 4-benzoylbenzoate* **B** (412 mg, 1.13 mmol). The crude material was obtained as 1:1 mixture of diastereomers. Purification by flash column chromatography (10:1 hexane:Et_2O) yielded the title product **1w** as a colourless oil (375 mg, 0.784 mmol, ca. 1:1 dr, 69%).

Data reported for mixture of diasteromers ($d_1 + d_2$)

^{1}H-NMR (400 MHz, $CDCl_3$): δ_H 8.07 – 8.01 (2x2H, m, $d_1 + d_2$), 7.62 – 7.57 (2x2H, m, $d_1 + d_2$), 7.49 – 7.44 (2x2H, m, $d_1 + d_2$), 7.41 – 7.29 (2x7H, m, $d_1 + d_2$), 4.94 (2x1H, td, J = 10.9, 4.4 Hz, $d_1 + d_2$), 3.04 (2x1H, s, $d_1 + d_2$), 2.19 – 2.09 (2x1H, m, $d_1 + d_2$), 2.02 – 1.93 (2x1H, m, $d_1 + d_2$), 1.80 – 1.69 (2x2H, m, $d_1 + d_2$), 1.62 – 1.50 (2x4H, m, $d_1 + d_2$), 1.34 – 1.26 (2x2H, m, $d_1 + d_2$), 1.22 – 1.04 (2x2H, m, $d_1 + d_2$), 0.97 – 0.93 (6H, m, d_2), 0.93 – 0.90 (6H, m, d_1), 0.82 (3H, d, J = 1.5 Hz, d_2), 0.80 (3H, d, J = 1.6 Hz, d_1)

^{13}C{^{1}H}-NMR (101 MHz, $CDCl_3$): δ_C 166.0, 149.3, 149.3, 144.3, 130.3, 130.2, 129.8, 128.8, 128.6, 128.6, 128.5, 128.1, 126.8, 126.7, 126.7, 116.9, 111.9, 78.2, 78.1, 75.0, 47.4, 41.1, 34.5, 31.7, 31.6, 26.7, 26.6, 23.8, 22.8, 22.2, 20.9, 16.7, 16.7, 14.3, 9.8

Note: Some signals are doubled due to the presence of diastereomers.

IR (neat, ν cm^{-1}): 3459, 2954, 2927, 2868, 1712, 1683, 1446, 1273, 1112, 1019, 693

HRMS (ESI-): *m/z* calcd for $C_{33}H_{35}O_3$ $[M-H]^-$ 479.2592; found 479.2572

Isopropyl 2-(4-((4-chlorophenyl)(hydroxy)(2-phenylcycloprop-1-en-1-yl)methyl)phenoxy)-2-methylpropanoate, 1w

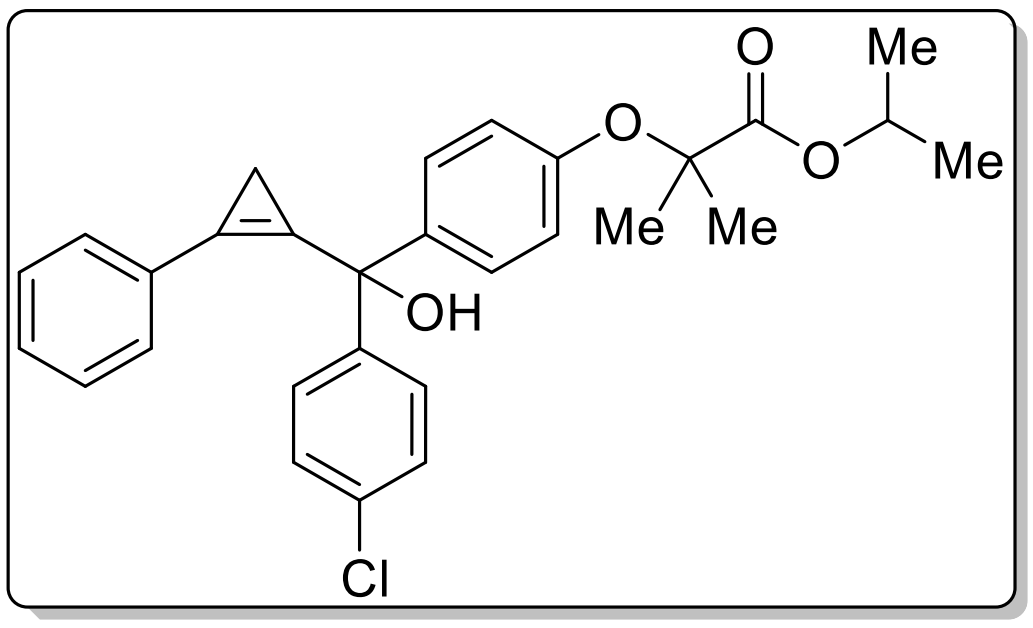

Prepared following **General Procedure 1A** using *(1,2,2-tribromocyclopropyl)benzene* **SM1** (200 mg, 0.570 mmol) and *fenofibrate* (205 mg, 0.570 mmol). Quantitative ^1H NMR analysis indicated an 58% yield for compound **1x** (158 mg, 0.331 mmol) with 1,3,5-trimethoxybenzene used as an internal standard. Purification by flash column chromatography (hexane to 5:1 hexane:Et_2O) yielded the title product as a colourless oil.

^{1}H-NMR (400 MHz, $CDCl_3$): δ_H 7.42 – 7.37 (2H, m), 7.34 – 7.30 (5H, m), 7.29 – 7.25 (4H, m), 6.85 – 6.80 (2H, m), 5.07 (1H, hept, *J* = 6.3 Hz), 2.79 (1H, s), 1.59 (6H, s), 1.52 (2H, d, *J* = 1.1 Hz), 1.21 (6H, d, *J* = 6.3 Hz)

The spectroscopic data are in agreement with those previously reported.[2]

4-Chloro-2,2,5-triphenyl-1-oxaspiro[2.3]hex-4-ene, 2a

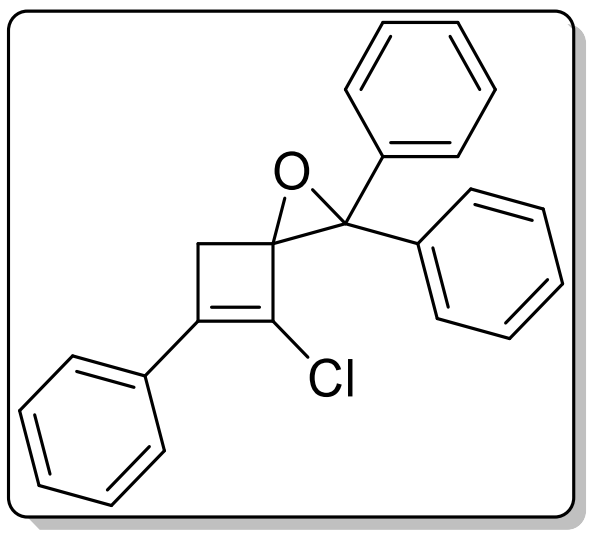

Prepared following **General Procedure 2** using *diphenyl(2-phenylcycloprop-1-en-1-yl)methanol* **1a** (60 mg, 0.20 mmol). The title compound **2a** was obtained as a colourless oil after work-up without further purification (65 mg, 0.19 mmol, 95%).

Representative example at gram-scale

To a vigorously stirred solution of *diphenyl(2-phenylcycloprop-1-en-1-yl)methanol* **1a** (1.2 g, 4.0 mmol), *benzyltriethylammonium chloride* (91 mg, 0.4 mmol) and *chloroform* (2.14 g, 1.44 mL, 18 mmol), an *aqueous 20 M NaOH solution* (1.8 mL, 36 mmol) was added dropwise. The resulting mixture was stirred at room temperature for 4 hours. It was then diluted with DCM and washed with water twice; the organic phase was dried over anhydrous sodium sulfate, filtered and the solvent removed under reduced pressure to afford the crude material, which was purified, when necessary, by flash column chromatography, as indicated in each specific case, to yield 4-chloro-2,2,5-triphenyl-1-oxaspiro[2.3]hex-4-ene (1.18 g, 3.46 mmol, 86%).

^{1}H-NMR (400 MHz, $CDCl_3$): δ_H 7.62 (dd, *J* = 8.0, 1.6 Hz, 2H), 7.57 (dd, *J* = 8.0, 1.5 Hz, 2H), 7.45 – 7.27 (m, 11H), 3.06 (d, *J* = 11.6 Hz, 1H) and 2.92 (d, *J* = 11.6 Hz, 1H) (*AB* syst.)

^{13}C{^{1}H}-NMR (101 MHz, $CDCl_3$): δ_C144.8, 139.6, 136.9, 131.7, 129.6, 128.7, 128.6, 128.3, 128.2, 128.2, 127.9, 127.1, 126.9, 118.9, 74.9, 68.4, 36.4

IR (neat, ν cm^{-1}): 3060, 3028, 2926, 1659, 1600, 1493, 1264, 1205, 1048, 905

HRMS (ESI+): *m/z* calcd for $C_{23}H_{17}{}^{35}ClONa$ $[M+Na]^+$ 367.0864; found 367.0866

4-Chloro-5-phenyl-2,2-di-*p*-tolyl-1-oxaspiro[2.3]hex-4-ene, 2b

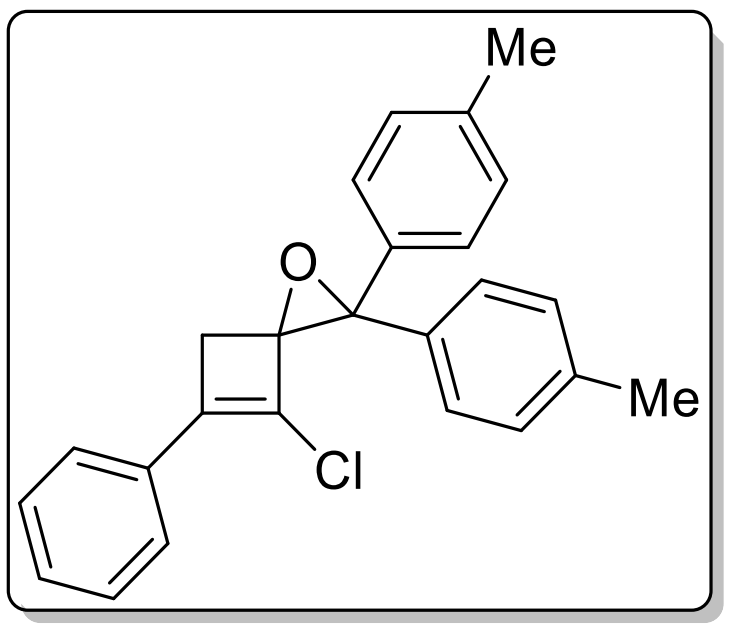

Prepared following **General Procedure 2** using *(2-phenylcycloprop-1-en-1-yl)di-p-tolylmethanol* **1b** (60 mg, 0.18 mmol). Purification by flash column chromatography (99:1 hexane:EtOAc) yielded the title product **2b** as a white crystalline solid (61 mg, 0.16 mmol, 91%).

^{1}H-NMR (400 MHz, $CDCl_3$): δ_H 7.65 – 7.58 (2H, m), 7.46 – 7.31 (5H, m), 7.25 – 7.18 (4H, m), 7.14 (2H, app. d, *J* = 8.1 Hz), 3.03 (1H, d, *J* = 11.5 Hz) and 2.90 (1H, d, *J* = 11.5 Hz) (*AB syst.*), 2.39 (3H, s), 2.34 (3H, s).

^{13}C{^{1}H}-NMR (101 MHz, $CDCl_3$): δ_C 144.5, 137.8, 137.5, 137.0, 134.1, 131.8, 129.5, 129.0, 128.8, 128.7, 128.5, 127.1, 126.8, 119.1, 74.9, 68.4, 36.5, 21.5, 21.2

IR (neat, ν cm^{-1}): 3025, 2922, 1716, 1511, 1446, 1295, 1049, 918, 812

HRMS (ESI+): *m/z* calcd for $C_{25}H_{21}{}^{35}ClONa$ $[M+Na]^+$ 395.1173; found 395.1214

Melting point 122.3 – 148.8 °C

4-Chloro-2,2-bis(4-fluorophenyl)-5-phenyl-1-oxaspiro[2.3]hex-4-ene, 2c

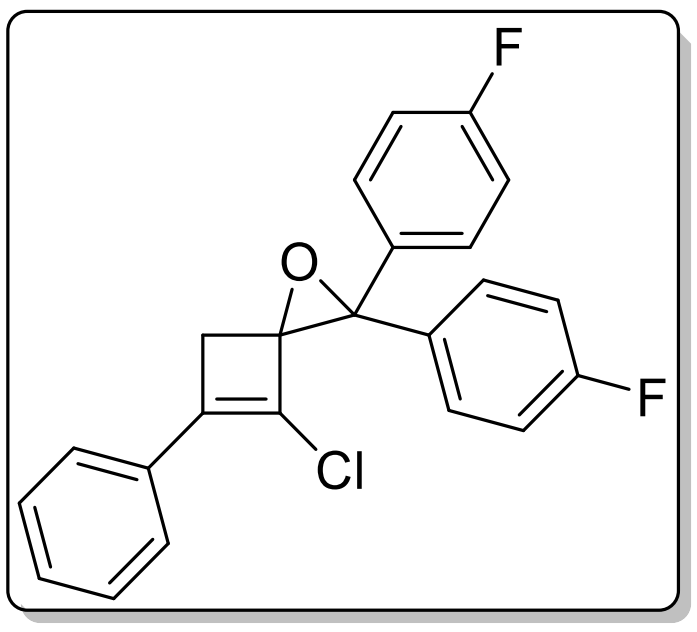

Prepared following **General Procedure 2** using *bis(4-fluorophenyl)(2-phenylcycloprop-1-en-1-yl)methanol* **1c** (60 mg, 0.18 mmol). Purification by flash column chromatography (99:1 hexane:EtOAc) yielded the title product **2c** as a white solid (17 mg, 0.045 mmol, 25%).

^{1}H-NMR (400 MHz, $CDCl_3$): δ_H 7.60 (2H, dd, *J* = 7.8, 1.7 Hz), 7.56 – 7.46 (2H, m), 7.44 – 7.34 (3H, m), 7.35 – 7.25 (2H, m), 7.15 – 6.98 (4H, m), 3.04 (1H, d, *J* = 11.6 Hz) and 2.88 (1H, d, *J* = 11.6 Hz) (*AB syst.*)

^{19}F-NMR (377 MHz, $CDCl_3$): δ_F -113.28 (m), -114.28 (m)

^{13}C{^{1}H}-NMR (101 MHz, $CDCl_3$): δ_C 162.8 (d, *J* = 247.1 Hz), 162.6 (d, *J* = 247.3 Hz), 145.0, 135.2 (d, *J* = 3.3 Hz), 132.6 (d, *J* = 3.3 Hz), 131.6, 130.4 (d, *J* = 8.3 Hz), 129.8, 128.8, 128.6 (d, *J* = 8.3 Hz), 127.1, 118.4, 115.3 (d, *J* = 21.6 Hz), 115.3 (d, *J* = 21.6 Hz), 74.9, 67.4, 36.3

IR (neat, ν cm^{-1}): 3063, 2925, 2852, 1605, 1507, 1447, 1223, 1155, 919, 830

HRMS (ESI+): *m/z* calcd for $C_{23}H_{15}{}^{35}ClF_2ONa$ $[M+Na]^+$ 403.0672; found 403.0678

4-Chloro-2,2-bis(4-chlorophenyl)-5-phenyl-1-oxaspiro[2.3]hex-4-ene, 2d

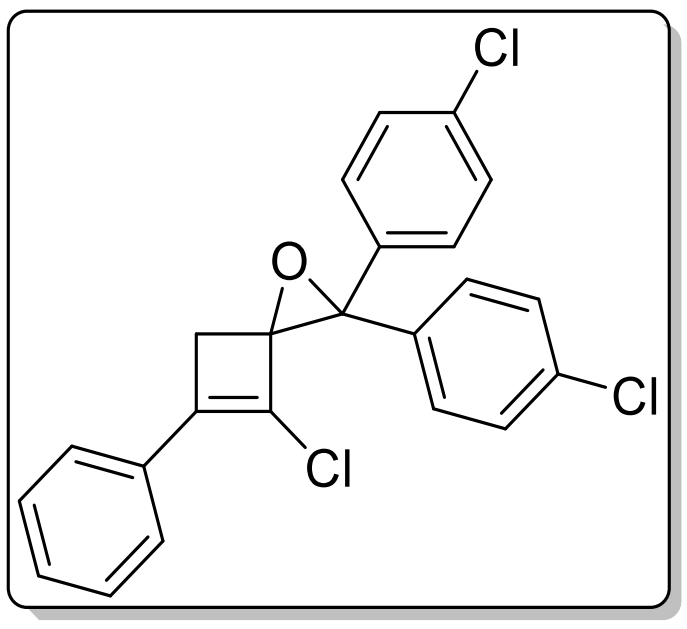

Prepared following **General Procedure 2** using *bis(4-chlorophenyl)(2-phenylcycloprop-1-en-1-yl)methanol* **1d** (65 mg, 0.18 mmol). Purification by flash column chromatography (hexane) yielded the title product **2d** as a white crystallin solid (49 mg, 0.12 mmol, 66%).

^{1}H-NMR (400 MHz, $CDCl_3$): δ_H 7.61 (2H, dd, *J* = 7.7, 1.9 Hz), 7.48 (2H, app. d, *J* = 8.4 Hz), 7.43 – 7.36 (5H, m), 7.36 – 7.25 (4H, m), 3.04 (1H, d, *J* = 11.6 Hz) and 2.88 (1H, d, *J* = 11.6 Hz) (*AB syst.*)

^{13}C{^{1}H}-NMR (101 MHz, $CDCl_3$): δ_C 145.1, 137.6, 134.9, 134.4, 134.2, 131.5, 130.0, 129.8, 128.8, 128.7, 128.6, 128.2, 127.1, 118.2, 74.9, 67.3, 36.2

IR (neat, ν cm^{-1}): 3062, 2923, 1634, 1489, 1392, 1267, 1090, 1014, 906, 815

HRMS (ESI-): *m/z* calcd for $C_{23}H_{14}{}^{35}Cl_3O$ [M-H]$^-$ 411.0116; found 411.0109

Melting point 155.1 – 166.4 °C

2-(3-Bromophenyl)-4-chloro-2,5-diphenyl-1-oxaspiro[2.3]hex-4-ene, 2e

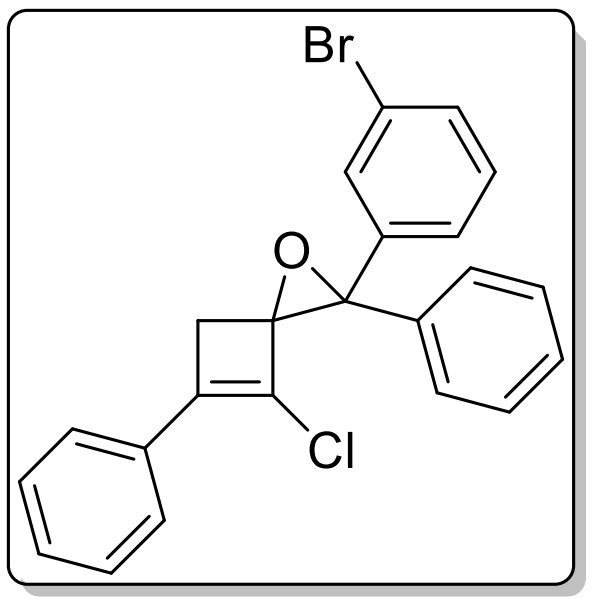

Prepared following **General Procedure 2** using *(3-bromophenyl)(phenyl)(2-phenylcycloprop-1-en-1-yl)methanol* **1e** (72 mg, 0.19 mmol). The crude material was obtained as 1:1 mixture of diastereomers. Purification by flash column chromatography (hexane to 98:2 hexane:Et_2O) yielded the title product **2e** as a yellow oil (67 mg, 0.16 mmol, ca. 1:1 dr, 84%).

Data reported for mixture of diastereomers (d_1 + d_2)

^{1}H-NMR (400 MHz, $CDCl_3$): δ_H 7.75 (1H, t, *J* = 1.8 Hz, d_1), 7.64 – 7.60 (4H, m, $d_1 + d_2$), 7.58 – 7.54 (4H, m, $d_1 + d_2$), 7.54 – 7.50 (2H, m, $d_1 + d_2$), 7.45 (1H, t, *J* = 1.6 Hz, d_2), 7.44 – 7.30 (16H, m, $d_1 + d_2$), 7.30 – 7.26 (1H, app. t, *J* = 7.9 Hz, d_1), 7.21 (1H, app. t, *J* = 7.9 Hz, d_2), 3.06 (1H, d, J = 11.5 Hz, d_2) and 2.92 (1H, d, *J* = 11.5 Hz, 1H, d_2) (*AB syst.*), 3.05 (1H, d, *J* = 11.6 Hz, d_1) and 2.90 (1H, d, *J* = 11.6 Hz, d_1) (*AB syst.*)

^{13}C{^{1}H}-NMR (101 MHz, $CDCl_3$): δ_C 145.1, 145.0, 142.0, 139.1, 138.8, 136.1, 131.6, 131.6, 131.4, 131.0, 129.9, 129.7, 129.7, 129.7, 128.7, 128.6, 128.5, 128.5, 128.3, 128.1, 127.3, 127.1, 126.8, 125.6, 122.7, 122.3, 118.5, 118.3, 74.9, 67.7, 67.7, 36.4, 36.2

Note: Signals are doubled due to the presence of diastereomers.

IR (neat, ν cm^{-1}): 3061, 3028, 2924, 1593, 1565, 1446, 1265, 1049, 906, 762

HRMS (ESI+): *m/z* calcd for $C_{23}H_{17}{}^{79}Br^{35}ClO$ $[M+H]^+$ 423.0146; found 423.0144

4-Chloro-2,5-diphenyl-2-(4-(*p*-tolylthio)phenyl)-1-oxaspiro[2.3]hex-4-ene, 2f

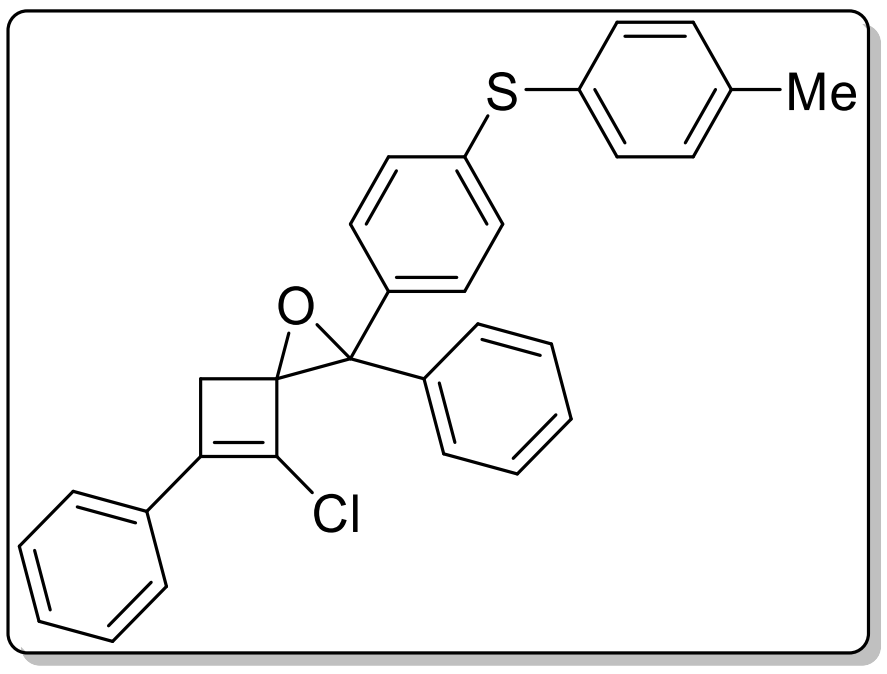

Prepared following **General Procedure 2** using *phenyl(2-phenylcycloprop-1-en-1-yl)(4-(p-tolylthio)phenyl)methanol* **1f** (81.5 mg, 0.19 mmol). The crude material was obtained as 1:1 mixture of diastereomers. The title compound **2f** was obtained as a colourless oil after work-up without further purification (52 mg, 0.12 mmol, ca. 1:1 dr, 61%).

Data reported for mixture of diastereomers (d_1 + d_2)

^{1}H-NMR (400 MHz, $CDCl_3$): δ_H 7.64 – 7.59 (4H, m, $d_1 + d_2$), 7.55 – 7.52 (2H, m, $d_1 + d_2$), 7.49 – 7.45 (2H, m, $d_1 + d_2$), 7.44 – 7.36 (10H, m, $d_1 + d_2$), 7.36 – 7.28 (10H, m, $d_1 + d_2$), 7.27 – 7.14 (8H, m, $d_1 + d_2$), 3.05 (1H, d, J = 11.5 Hz, d_2) and 2.93 (1H, d, J = 11.5 Hz, d_2) (*AB syst.*), 3.04 (1H, d, J = 11.6 Hz, d_1) and 2.91 (1H, d, J = 11.6 Hz, d_1) (*AB syst.*), 2.37 (s, 3H, d_1), 2.36 (s, 3H, d_2)

^{13}C{^{1}H}-NMR (101 MHz, $CDCl_3$): δ_C 144.8, 144.7, 139.3, 138.2, 137.9, 137.6, 137.5, 137.3, 136.6, 135.1, 133.1, 132.5, 131.6, 131.0, 130.3, 130.3, 129.6, 129.3, 129.2, 129.2, 129.0, 128.9, 128.8, 128.7, 128.6, 128.6, 128.4, 128.3, 128.3, 128.2, 127.9, 127.5, 127.1, 126.8, 118.8, 118.6, 74.9, 68.1, 68.0, 36.4, 36.4, 21.3

Note: Apart from signals at 74.9 and 21.3 ppm, signals are doubled due to the presence of diastereomers.

IR (neat, ν cm^{-1}): 3058, 3023, 2922, 2865, 1596, 1489, 1446, 1015, 906, 809

HRMS (ESI+): *m/z* calcd for $C_{30}H_{23}{}^{35}ClOSNa$ 489.1050 $[M+Na]^+$*;* found 489.1053

4-Chloro-2,5-diphenyl-2-(trifluoromethyl)-1-oxaspiro[2.3]hex-4-ene, 2g

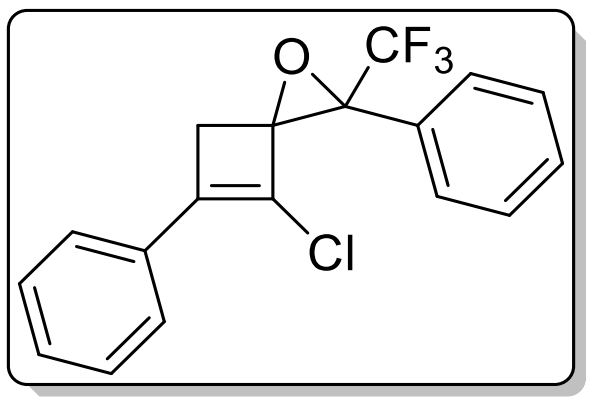

Prepared following **General Procedure 2** using *2,2,2-trifluoro-1-phenyl-1-(2-phenylcycloprop-1-en-1-yl)ethan-1-ol* **1g** (55 mg, 0.19 mmol). The crude material was obtained as 1:1 mixture of diastereomers. Purification by flash column chromatography (99:1 hexane:EtOAc) yielded the title product **2g** as a yellowish oil (23 mg, 0.70 mmol, ca. 60:40 dr, 37%).

Data reported for mixture of diastereomers (major + minor)

^{1}H-NMR (400 MHz, $CDCl_3$): δ_H 7.67 – 7.59 (4H, m, *major*), 7.59 – 7.56 (2H, m, *minor*), 7.55 – 7.51 (2H, m, *minor*), 7.47 – 7.38 (12H, m, *major + minor*), 3.47 – 3.39 (1H, m, *minor*), 3.29 (1H, d, *J* = 12.2 Hz, *minor*), 2.73 (1H, d, *J* = 11.9 Hz, *major*), 2.61 (1H, d, *J* = 11.9 Hz, *major*)

^{19}F-NMR (377 MHz, $CDCl_3$): δ_F -67.23 (s, *major*), -72.22 (s, *minor*)

^{13}C{^{1}H}-NMR (101 MHz, $CDCl_3$): δ_C 145.7, 140.7, 131.6, 131.2, 131.1, 130.2, 130.2, 129.7, 129.4, 129.2, 128.8, 128.8, 128.8, 128.4, 128.2, 127.4, 127.4, 126.7, 123.6 (q, *J* = 279.1 Hz), 123.4 (q, *J* = 279.1 Hz), 116.1, 115.9, 73.2, 71.1, 36.8, 36.1

Note: Signals are doubled due to the presence of diastereomers.

IR (neat, ν cm^{-1}): 3065, 2926, 2851, 1605, 1490, 1448, 1326, 1267, 1058, 949, 762

HRMS (ESI+): *m/z* calcd for $C_{18}H_{12}{}^{35}ClF_3ONa$ $[M+Na]^+$ 359.0421; found 359.0422

4-Chloro-2,2-dimethyl-5-phenyl-1-oxaspiro[2.3]hex-4-ene, 2h

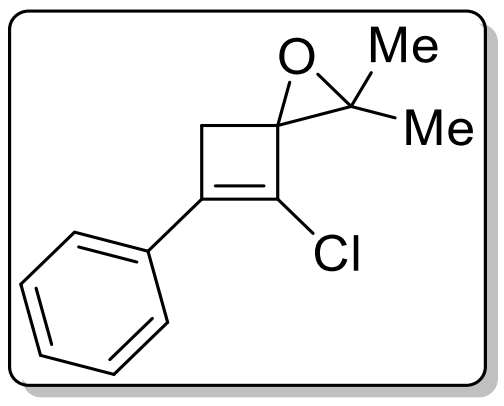

Prepared following **General Procedure 2** using *2-(2-phenylcycloprop-1-en-1-yl)propan-2-ol* **1h** (35 mg, 0.20 mmol). The title compound **2h** was obtained as a colourless oil after work-up without further purification (33 mg, 0.15 mmol, 75%).

^{1}H-NMR (400 MHz, $CDCl_3$): δ_H 7.68 – 7.62 (2H, m), 7.44 – 7.34 (3H, m), 3.06 (1H, d, J = 11.2 Hz) and 2.90 (1H, d, J = 11.2 Hz) (*AB syst.*), 1.61 (3H, s), 1.42 (3H, s)

^{13}C{^{1}H}-NMR (101 MHz, $CDCl_3$): δ_C 143.4, 131.8, 129.3, 128.7, 127.0, 118.8, 72.3, 62.5, 35.7, 23.0, 20.4

IR (neat, ν cm^{-1}): 3021, 2960, 2922, 1632, 1489, 1375, 1264, 1208, 1007, 839

HRMS (ESI+): *m/z* calcd for $C_{13}H_{13}{}^{35}ClONa$ $[M+Na]^+$ 243.0547; found 243.0607

1-Chloro-2-phenyl-11-oxadispiro[3.0.5^5.1^4]undec-1-ene, 2i

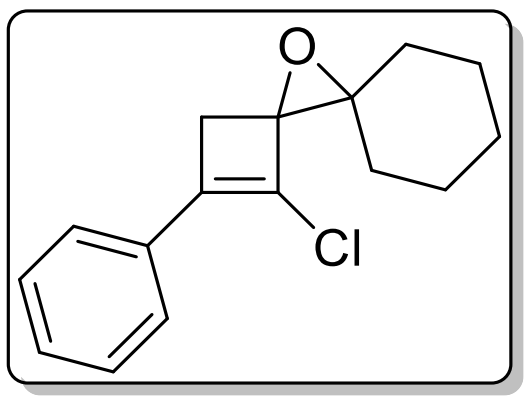

Prepared following **General Procedure 2** using *1-(2-phenylcycloprop-1-en-1-yl)cyclohexan-1-ol* **1i** (41 mg, 0.19 mmol). Purification by flash column chromatography (99:1 hexane:EtOAc) yielded the title product **2i** as a yellow oil (43 mg, 0.163 mmol, 86%).

^{1}H-NMR (400 MHz, $CDCl_3$): δ_H 7.69 – 7.62 (2H, m), 7.43 – 7.34 (3H, m), 3.05 (1H, d, *J* = 11.3 Hz) and 2.90 (1H, d, *J* = 11.3 Hz) (*AB syst.*), 2.02 – 1.87 (2H, m), 1.85 – 1.76 (2H, m), 1.68 – 1.59 (2H, m), 1.61 – 1.49 (4H, m)

^{13}C{^{1}H}-NMR (101 MHz, $CDCl_3$): δ_C 143.3, 131.9, 129.4, 128.7, 127.1, 118.8, 72.9, 67.0, 35.5, 33.5, 30.8, 25.6, 25.1, 24.9

IR (neat, ν cm^{-1}): 3059, 2930, 2855, 1632, 1488, 1447, 1260, 1185, 971, 840

HRMS (ESI+): *m/z* calcd for $C_{16}H_{17}{}^{35}ClONa$ $[M+Na]^+$ 283.0860; found 283.0903

tert-Butyl 1-chloro-2-phenyl-11-oxa-8-azadispiro[3.0.5^5.1^4]undec-1-ene-8-carboxylate, 2j

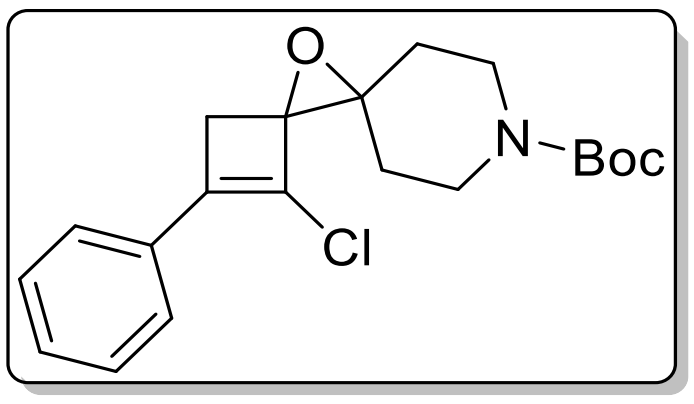

Prepared following **General Procedure 2** using *tert-butyl 4-hydroxy-4-(2-phenylcycloprop-1-en-1-yl)piperidine-1-carboxylate* **1j** (55 mg, 0.19 mmol). The title compound **2j** was obtained as a yellow oil after work-up without further purification (59 mg, 0.16 mmol, 81%).

^{1}H-NMR (400 MHz, $CDCl_3$): δ_H 7.66 – 7.61 (2H, m, 7.45 – 7.34 (3H, m), 3.78 – 3.68 (2H, m), 3.49 (2H, ddd, *J* = 13.2, 9.3, 3.7 Hz), 3.04 (1H, d, *J* = 11.3 Hz) and 2.93 (1H, d, *J* = 11.3 Hz) (*AB syst.*), 2.09 – 2.03 (1H, m), 2.00 – 1.93 (1H, m), 1.87 – 1.80 (1H, m), 1.59 – 1.51 (1H, m), 1.47 (9H, s)

^{13}C{^{1}H}-NMR (101 MHz, $CDCl_3$): δ_C 154.9, 143.8, 131.6, 129.6, 128.8, 127.1, 80.0, 72.6, 64.8, 42.4, 42.1, 35.4, 32.6, 30.4, 28.6

IR (neat, ν cm^{-1}): 2974, 2927, 2875, 1692, 1420, 1231, 1167, 1125, 763

HRMS (ESI+): *m/z* calcd for $C_{20}H_{25}{}^{35}ClNO_3$ $[M+H]^+$ 362.1517; found 362.1517

1-Chloro-2,8-diphenyl-11-oxadispiro[3.0.5^{5}.1^{4}]undec-1-ene, 2k

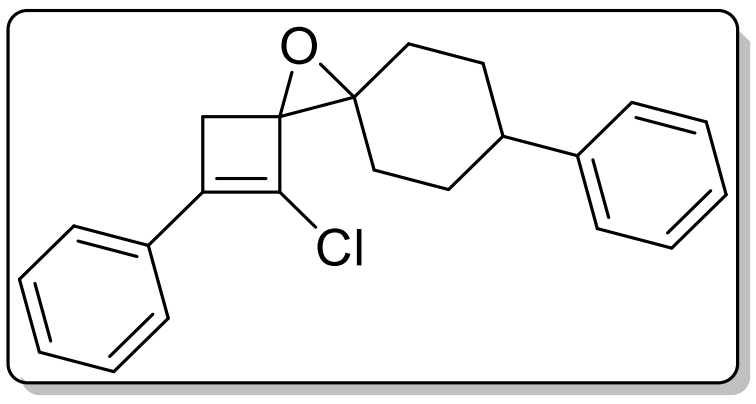

Prepared following **General Procedure 2** using *4-phenyl-1-(2-phenylcycloprop-1-en-1-yl)cyclohexan-1-ol* **1k** (63 mg, 0.2 mmol). The crude material was obtained as a single diastereomer. Purification by flash column chromatography (10:1 hexane:Et_2O to 5:1 hexane:Et_2O) yielded the title product **2k** as a yellow oil (52 mg, 0.14 mmol, 72%).

^{1}H-NMR (400 MHz, $CDCl_3$): δ_H 7.71 – 7.67 (2H, m), 7.47 – 7.37 (3H, m), 7.32 – 7.26 (2H, m), 7.25 – 7.17 (3H, m), 3.13 (1H, d, *J* = 11.4 Hz) and 2.96 (1H, d, *J* = 11.3 Hz) (*AB syst.*), 2.67 (1H, tt, *J* = 12.0, 3.3 Hz), 2.30 – 2.21 (1H, m), 2.16 – 2.05 (3H, m), 1.76 – 1.53 (4H, m)

^{13}C{^{1}H}-NMR (101 MHz, $CDCl_3$): δ_C 145.9, 143.5, 131.8, 129.5, 128.8, 128.6, 127.2, 126.9, 126.4, 118.5, 72.9, 66.8, 43.4, 35.6, 33.7, 33.4, 33.3, 30.9

IR (neat, ν cm^{-1}): 3026, 2933, 1604, 1488, 1230, 1178, 1025, 736

HRMS (ESI+): *m/z* calcd for $C_{22}H_{22}{}^{35}ClO$ $[M+H]^+$ 337.1354; found 337.1348

4-Chloro-5-(4-fluorophenyl)-2,2-diphenyl-1-oxaspiro[2.3]hex-4-ene, 2l

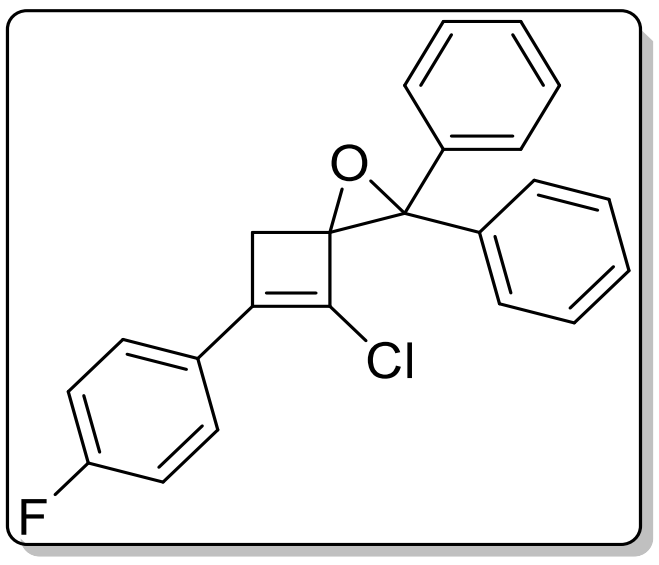

Prepared following **General Procedure 2** using *(2-(4-fluorophenyl)cycloprop-1-en-1-yl)diphenylmethanol* **1n** (70 mg, 0.20 mmol). Purification by flash column chromatography (99:1 hexane:EtOAc) yielded the title product **2l** as a white solid (63 mg, 0.17 mmol, 88%).

^{1}H-NMR (400 MHz, $CDCl_3$): δ_H 7.63 – 7.53 (4H, m), 7.43 – 7.36 (4H, m), 7.35 – 7.27 (4H, m), 7.13 – 7.05 (2H, m), 3.03 (1H, d, *J* = 11.6 Hz) and 2.89 (1H, d, *J* = 11.6 Hz) (*AB syst.*)

^{19}F-NMR (377 MHz, $CDCl_3$): δ_F -109.83 (m)

^{13}C{^{1}H}-NMR (101 MHz, $CDCl_3$): δ_C 163.2 (d, *J* = 250.8 Hz), 143.6, 139.5, 136.8, 129.1 (d, *J* = 8.4 Hz), 128.6, 128.4, 128.3, 128.2, 127.9, 126.8, 118.3 (d, *J* = 2.7 Hz), 115.9 (d, *J* = 21.9 Hz), 74.9, 68.4, 36.5

IR (neat, ν cm^{-1}): 3061, 2925, 1600, 1504, 1448, 1234, 1157, 1053, 835

HRMS (ESI-): *m/z* calcd for $C_{23}H_{15}{}^{35}ClFO$ $[M-H]^-$ 361.0801; found 361.0836

4-Chloro-5-isopentyl-2,2-diphenyl-1-oxaspiro[2.3]hex-4-ene, 2m

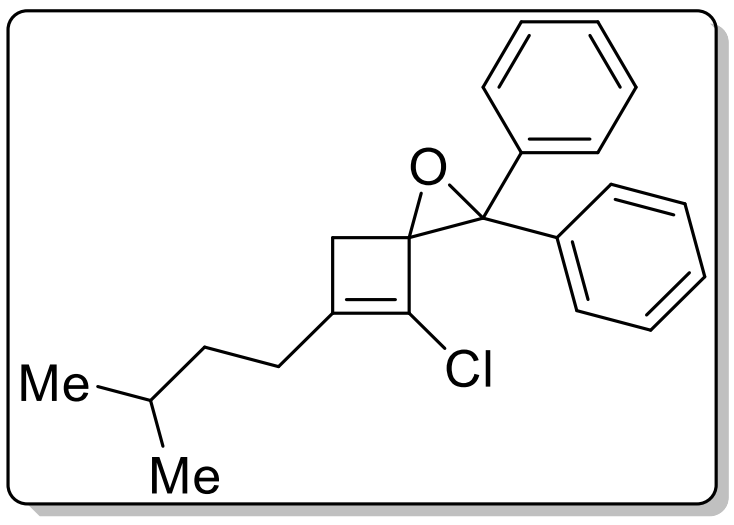

Prepared following **General Procedure 2** using *(2-isopentylcycloprop-1-en-1-yl)diphenylmethanol* **1o** (55 mg, 0.19 mmol). Purification of the crude material by flash column chromatography led to partial Meinwald rearrangement in all conditions, due to the instability of the target compound on SiO_2. Therefore, the title product **2m** was characterised after workup without further purification, yielding a yellow oil (51 mg, 0.15 mmol, 79%).

^{1}H-NMR (400 MHz, $CDCl_3$): δ_H 7.53 – 7.46 (2H, m), 7.44 – 7.38 (2H, m), 7.37 – 7.27 (6H, m), 2.69 (1H, d, *J* = 11.9 Hz) and 2.52 (1H, d, *J* = 11.9 Hz) (*AB syst.*), 2.34 – 2.23 (2H, m), 1.61 – 1.52 (1H, m), 1.43 – 1.31 (2H, m), 0.91 (3H, d, *J* = 2.2 Hz), 0.90 (3H, d, *J* = 2.4 Hz)

^{13}C{^{1}H}-NMR (101 MHz, $CDCl_3$): δ_C 147.3, 139.8, 137.1, 128.6, 128.2, 128.2, 128.1, 126.9, 126.2, 119.1, 75.3, 67.8, 38.0, 35.3, 27.9, 25.8, 22.5, 22.4

IR (neat, ν cm^{-1}): 3026, 2095, 2025, 1601, 1494, 1385, 1032, 1002, 736

HRMS (ESI+): *m/z* calcd for $C_{22}H_{23}{}^{35}ClONa$ $[M+Na]^+$ 361.1330; found 361.1352

4-Chloro-5-isopentyl-2,2-dimethyl-1-oxaspiro[2.3]hex-4-ene, 2n

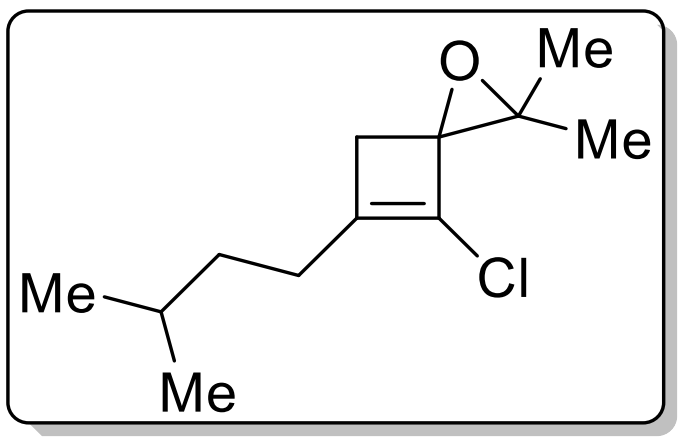

Prepared following **General Procedure 2** using *2-(2-isopentylcycloprop-1-en-1-yl)propan-2-ol* **1p** (34 mg, 0.20 mmol). Purification by flash column chromatography (hexane to 98:2 hexane:Et_2O) yielded the title product **2n** as a yellow oil (13 mg, 0.064 mmol, 31%).

^{1}H-NMR (400 MHz, $CDCl_3$): δ_H 2.65 (1H, dt, *J* = 11.5, 1.1 Hz), 2.52 (1H, dt, *J* = 11.5, 1.2 Hz), 2.36 – 2.14 (2H, m), 1.68 – 1.51 (1H, m), 1.51 (3H, s), 1.49 – 1.34 (2H, m), 1.33 (3H, s), 0.93 (s, 3H), 0.91 (s, 3H).

^{13}C{^{1}H}-NMR (101 MHz, $CDCl_3$): δ_C 149.8, 118.8, 73.4, 61.9, 37.4, 35.3, 27.9, 25.7, 23.0, 22.5, 22.4, 20.5

IR (neat, ν cm^{-1}): 2956, 2925, 2870, 1738, 1468, 1375, 1206, 1014, 843

HRMS (ESI+): m/z calcd for $C_{12}H_{20}{}^{35}ClO$ $[M+H]^+$ 215.1197; found 215.1192

4-Chloro-2,2-diphenyl-5-(3-phenylpropyl)-1-oxaspiro[2.3]hex-4-ene, 2o

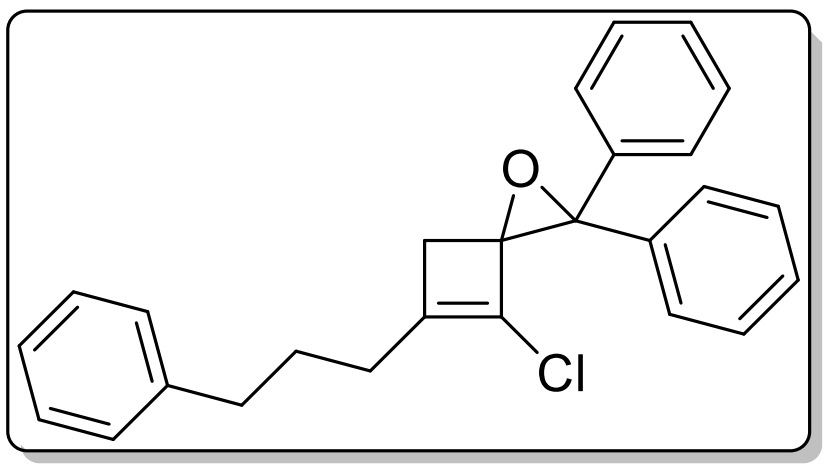

Prepared following **General Procedure 2** using *diphenyl(2-(3-phenylpropyl)cycloprop-1-en-1-yl)methanol* **1q** (62 mg, 0.182 mmol). The title compound **2o** was obtained as a colourless oil after work-up without further purification (64 mg, 0.164 mmol, 90%).

^{1}H-NMR (400 MHz, $CDCl_3$): δ_H 7.55 – 7.51 (2H, m), 7.45 – 7.35 (4H, m), 7.34 – 7.27 (6H, m), 7.23 – 7.17 (3H, m), 2.72 (1H, d, *J* = 11.9 Hz) and 2.54 (1H, d, *J* = 11.9 Hz) (*AB syst.*), 2.68 – 2.63 (2H, m), 2.39 – 2.25 (2H, m), 1.86 (2H, p, *J* = 7.7 Hz)

^{13}C{^{1}H}-NMR (101 MHz, $CDCl_3$): δ_C 150.7, 141.7, 139.7, 137.0, 128.6, 128.5, 128.5, 128.2, 128.1, 127.7, 126.8, 126.2, 126.1, 119.8, 75.2, 67.8, 38.1, 35.7, 28.1, 27.4

IR (neat, ν cm^{-1}): 3026, 2930, 2859, 1602, 1494, 1448, 1075, 1031, 750

HRMS (ESI+): m/z calcd for $C_{26}H_{23}{}^{35}ClONa$ $[M+Na]^+$ 409.1330; found 409.1341

4-Chloro-2,2-dimethyl-5-(3-phenylpropyl)-1-oxaspiro[2.3]hex-4-ene, 2p

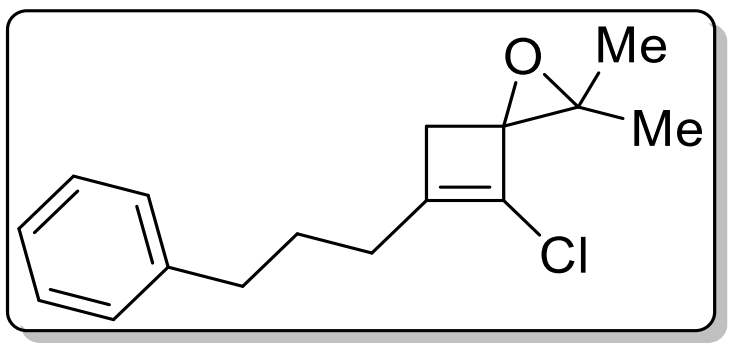

Prepared following **General Procedure 2** using *2-(2-(3-phenylpropyl)cycloprop-1-en-1-yl)propan-2-ol* **1r** (43 mg, 0.20 mmol). Purification by flash column chromatography (hexane to 98:2 hexane:Et_2O) yielded the title product **2p** as a colourless oil (46 mg, 0.18 mmol, 88%).

^{1}H-NMR (400 MHz, $CDCl_3$): δ_H 7.33 – 7.25 (2H, m), 7.24 – 7.16 (3H, m), 2.72 – 2.60 (3H, m), 2.53 (1H, dt, *J* = 11.5, 1.2 Hz), 2.37 – 2.23 (2H, m), 1.88 (2H, p, *J* = 7.8 Hz), 1.52 (3H, s), 1.33 (s, 1H).

^{13}C{^{1}H}-NMR (101 MHz, $CDCl_3$): δ_C 149.1, 141.8, 128.6, 128.5, 126.1, 119.5, 73.3, 62.0, 37.4, 35.8, 28.1, 27.4, 23.0, 20.5

IR (neat, ν cm^{-1}): 3025, 2969, 2934, 2875, 1605, 1427, 1302, 1206, 1067, 871

HRMS (ESI+): m/z calcd for $C_{16}H_{20}{}^{35}ClO$ $[M+H]^+$ 263.1197; found 263.1207

tert-Butyl 2''-chloro-3''-phenyl-8-azadispiro[bicyclo[3.2.1]octane-3,2'-oxirane-3',1''-cyclobutan]-2''-ene-8-carboxylate, 2q

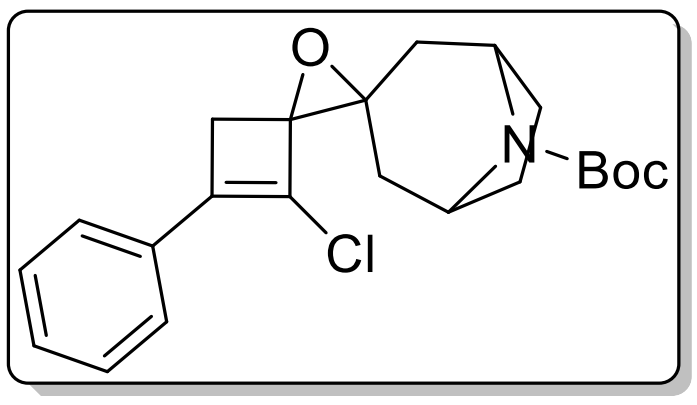

Prepared following **General Procedure 2** using *tert-butyl 3-hydroxy-3-(2-phenylcycloprop-1-en-1-yl)-8-azabicyclo[3.2.1]octane-8-carboxylate* **1s** (70 mg, 0.21 mmol). The title compound **2q** was obtained as a yellow oil after work-up without further purification (65 mg, 0.17 mmol, 80%).

^{1}H-NMR (400 MHz, $CDCl_3$): δ_H 7.65 – 7.57 (2H, m), 7.45 – 7.31 (3H, m), 4.40 – 4.13 (2H, m), 2.94 (1H, d, *J* = 11.2 Hz) and 2.85 (1H, d, *J* = 11.3 Hz) (*AB syst.*), 2.44 – 2.13 (4H, m), 2.06 – 1.82 (4H, m), 1.47 (9H, s)

^{13}C{^{1}H}-NMR (101 MHz, $CDCl_3$): δ_C 153.4, 143.7, 131.7, 129.4, 128.7, 127.0, 118.1, 79.6, 68.7, 62.7, 53.5, 52.9, 38.1, 37.1, 35.0, 28.6, 28.2, 27.6

IR (neat, ν cm^{-1}): 2976, 2930, 1685, 1390, 1160, 1097, 1010, 908, 728

HRMS (ESI-): m/z calcd for $C_{22}H_{27}{}^{37}ClNO_3$ $[M-H]^-$ 388.1674; found 388.1667

2''-Chloro-2,3''-diphenyldispiro[chromane-4,2'-oxirane-3',1''-cyclobutan]-2''-ene, 2r

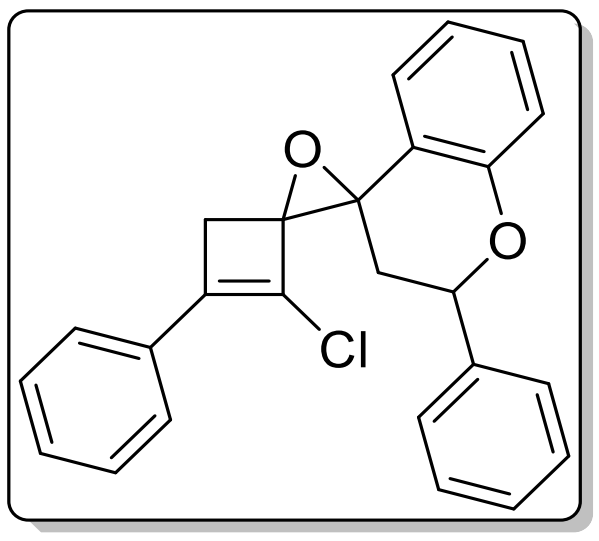

Prepared following **General Procedure 2** using *2-phenyl-4-(2-phenylcycloprop-1-en-1-yl)chroman-4-ol* **1t** (65 mg, 0.19 mmol). The crude material was obtained as 1:1 mixture of diastereomers. Purification by flash column chromatography (hexane to 98:2 hexane:Et_2O) yielded the title product **2r** as a colourless oil (47 mg, 0.12 mmol, ca. 1:1 dr, 63%).

Data reported for mixture of diastereomers (d_1 + d_2)

^{1}H-NMR (400 MHz, $CDCl_3$): δ_H 7.73 – 7.65 (4H, m, $d_1 + d_2$), 7.53 – 7.36 (17H, m, $d_1 + d_2$), 7.30 – 7.24 (2x1H, m, $d_1 + d_2$), 7.14 (1H, dd, J = 7.7, 1.7 Hz, d_2), 7.03 – 6.93 (2x2H, m, $d_1 + d_2$), 5.41 – 5.35 (1x2H, m, $d_1 + d_2$), 3.18 (1H, d, J = 11.3 Hz, d_1) and 3.04 (1H, d, J = 11.4 Hz, d_1) (*AB syst., d_1*), 3.17 (1H, d, J = 11.5 Hz, d_2) and 3.00 (1H, d, J = 11.5 Hz, d_2) (*AB syst., d_2*), 2.79 – 2.66 (1H – d_2 + 2x1H - $d_1 + d_2$, m), 1.97 (1H, dd, J = 13.4, 2.0 Hz, d_1)

^{13}C{^{1}H}-NMR (101 MHz, $CDCl_3$): δ_C 157.0, 156.8, 145.2, 144.4, 140.3, 140.1, 131.5, 131.5, 130.0, 129.9, 129.8, 129.8, 128.9, 128.9, 128.8, 128.8, 128.8, 128.5, 127.2, 127.2, 127.1, 126.3, 126.1, 125.1, 121.4, 120.8, 120.0, 119.1, 118.2, 117.0, 116.9, 116.3, 78.3, 77.7, 75.3, 74.9, 63.1, 62.5, 37.4, 36.4, 35.9, 35.0

Note: Signals are doubled due to the presence of diastereomers.

IR (neat, ν cm^{-1}): 3032, 2963, 2912, 1610, 1580, 1248, 1113, 1051, 757

HRMS (ESI+): m/z calcd for $C_{25}H_{20}{}^{35}ClO_2$ $[M+H]^+$ 387.1146; found 387.1140

1,7,7-Trimethylbicyclo[2.2.1]heptan-2-yl 4-(4-chloro-2,5-diphenyl-1-oxaspiro[2.3]hex-4-en-2-yl)benzoate, 2s

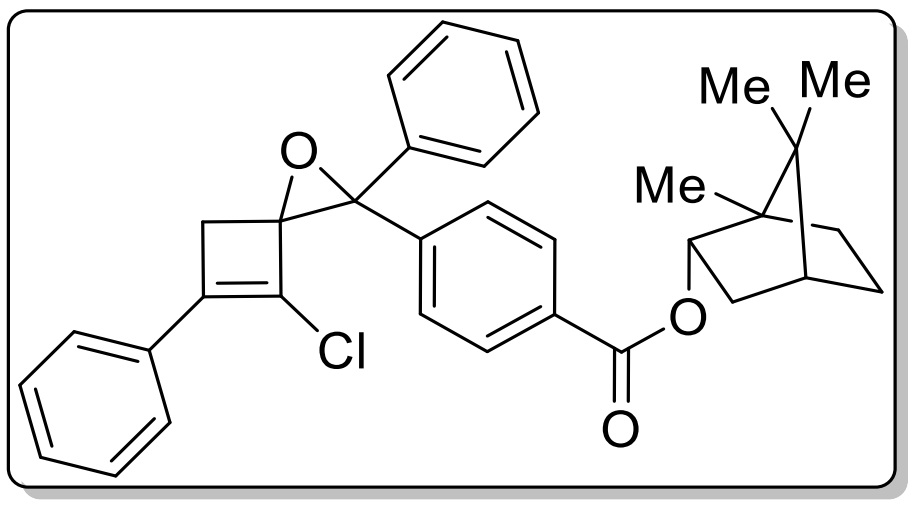

Prepared following **General Procedure 2** using *1,7,7-trimethylbicyclo[2.2.1]heptan-2-yl 4-(hydroxy(phenyl)(2-phenylcycloprop-1-en-1-yl)methyl)benzoate* **1u** (90 mg, 0.19 mmol). The crude material was obtained as 1:1 mixture of diastereomers. The title compound **2s** was obtained as a yellow oil after work-up without further purification (82 mg, 0.16 mmol, ca. 1:1 dr, 82%).

Data reported for mixture of diastereomers ($d_1 + d_2$)

^{1}H-NMR (400 MHz, $CDCl_3$): δ_H 8.14 – 8.08 (2H, m, d_1), 8.07 – 8.01 (2H, m, d_2), 7.70 – 7.63 (2H, d_1), 7.64 – 7.57 (2x2H, m, $d_1 + d_2$), 7.59 – 7.53 (2H, m, d_2), 7.50 – 7.39 (8H, m. $d_1 + d_2$), 7.38 – 7.30 (8H, m, $d_1 + d_2$), 5.19 – 5.05 (2x1H, m, $d_1 + d_2$), 3.06 (1H, d, J = 11.7 Hz) and 2.95 (1H, d, J = 11.6 Hz) (AB syst., d_1), 3.05 (1H, d, J = 11.6 Hz) and 2.86 (1H, d, J = 11.6 Hz) (AB syst., d_2), 2.54 – 2.43 (2x1H, m, $d_1 + d_2$), 2.17 – 2.06 (2x1H, m, $d_1 + d_2$), 1.85 – 1.79 (2x1H, m, $d_1 + d_2$), 1.75 (2x1H, q, J = 4.7 Hz, $d_1 + d_2$), 1.47 – 1.39 (2x1H, m, $d_1 + d_2$), 1.35 – 1.25 (2x1H, m, $d_1 + d_2$), 1.17 – 1.08 (2x1H, m), 0.98 (3H, s, d_1), 0.97 (3H, s, d_2), 0.94 – 0.92 (2x3H, m, $d_1 + d_2$), 0.92 – 0.90 (2x3H, m, $d_1 + d_2$)

^{13}C{^{1}H}-NMR (101 MHz, $CDCl_3$): δ_C ^{13}C NMR (101 MHz, $CDCl_3$) δ 166.7, 166.6, 145.1, 145.0, 144.4, 141.7, 138.7, 136.1, 132.6, 131.6, 130.7, 130.4, 129.7, 129.7, 129.6, 129.6, 129.4, 128.7, 128.6, 128.5, 128.5, 128.3, 128.1, 127.1, 127.1, 126.8, 126.8, 118.6, 118.4, 80.8, 80.8, 80.7, 74.9, 74.9, 68.1, 68.0, 49.2, 48.0, 48.0, 45.1, 45.1, 37.1, 37.0, 36.4, 36.3, 28.2, 27.5, 27.5, 19.9, 19.8, 19.1, 19.0, 13.8, 13.7

Note: Signals are doubled due to the presence of diastereomers.

IR (neat, ν cm^{-1}): 3023, 2954, 2878,1712, 1453, 1300, 1270, 1116, 906

HRMS (ESI+): m/z calcd for $C_{34}H_{34}{}^{35}ClO_3$ $[M+H]^+$ 521.2191; found 525.2191

2-Isopropyl-5-methylcyclohexyl 4-(4-chloro-2,5-diphenyl-1-oxaspiro[2.3]hex-4-en-2-yl)benzoate, 2t

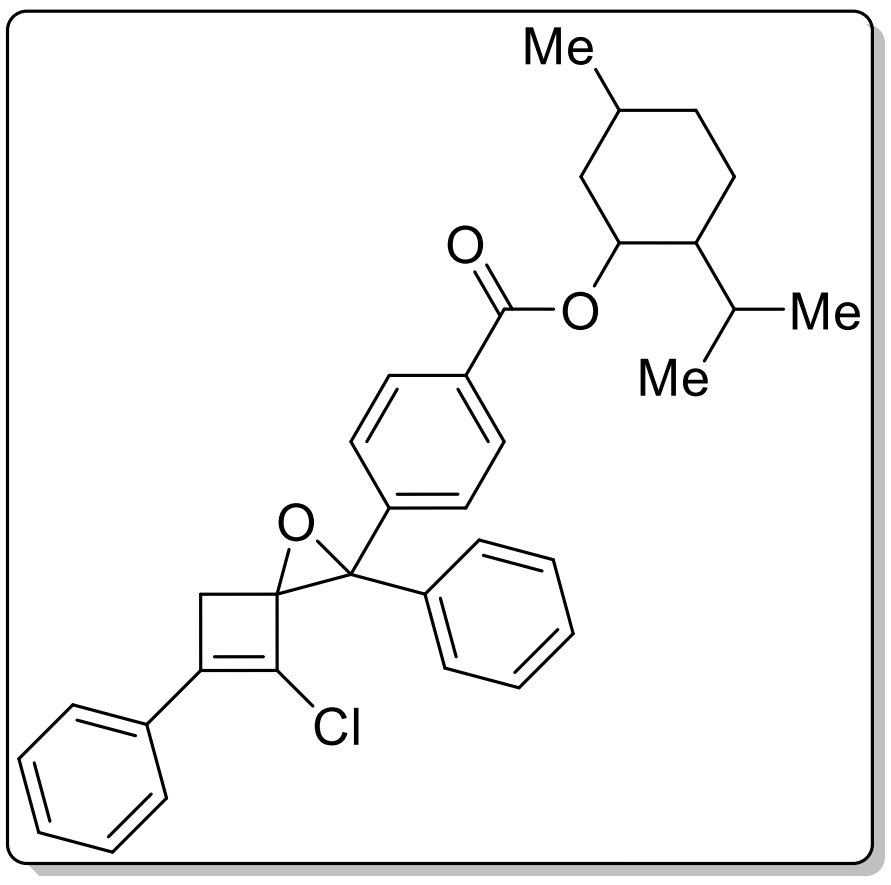

Prepared following **General Procedure 2** using *2-isopropyl-5-methylcyclohexyl 4-(hydroxy(phenyl)(2-phenylcycloprop-1-en-1-yl)methyl)benzoate* **1v** (100 mg, 0.20 mmol). The crude material was obtained as 1:1:1:1 mixture of diastereomers. The title compound **2t** was obtained as a yellow oil after work-up without further purification (72 mg, 0.13 mmol, ca. 1:1:1:1 dr, 64%).

Data reported for mixture of diasteromers ($d_1 + d_2 + d_3 + d_4$)

^{1}H-NMR (400 MHz, $CDCl_3$): δ_H 8.12 – 8.05 (2x2H, m), 8.01 (2x2H, app. d, *J* = 8.1 Hz), 7.65 (2x2H, d, *J* = 8.2 Hz), 7.62 – 7.58 (6H, m), 7.56 – 7.51 (4H, m), 7.45 (2x2H, d, *J* = 8.2 Hz), 7.42 – 7.27 (26H, m), 4.94 (4x1H, dtd, *J* = 12.4, 10.9, 4.4 Hz, $d_1 + d_2 + d_3 + d_4$), 3.09 – 3.00 (4x1H, m), 2.94 (2x1H, d, *J* = 11.6 Hz), 2.86 (2x1H, d, *J* = 11.6 Hz), 2.20 – 2.07 (4x1H, m), 2.00 – 1.91 (4x1H, m), 1.77 – 1.69 (4x2H, m), 1.59 – 1.49 (4x2H, m), 1.18 – 1.06 (4x2H, m), 0.96 – 0.93 (2x6H, m), 0.91 (2x6H, d, *J* = 7.1 Hz), 0.81 (2x3H, dd, *J* = 6.9, 2.3 Hz), 0.79 (2x3H, d, *J* = 6.9 Hz)

^{13}C{^{1}H}-NMR (101 MHz, $CDCl_3$): δ_C 166.0, 165.9, 145.0, 145.0, 144.3, 141.6, 138.8, 138.8, 136.2, 136.2 131.6, 130.7, 130.4, 130.4, 129.7, 129.7, 129.7, 129.6, 129.5, 128.8, 128.6, 128.5, 128.5, 128.5, 128.3, 128.1, 127.2, 126.8, 118.6, 118.4, 75.1, 75.1, 74.9, 74.9, 47.4, 41.1, 41.1, 41.1, 36.5, 36.2, 34.5, 34.5, 31.6, 31.6, 26.7, 26.7, 26.6, 26.6, 23.8, 23.8, 23.7, 22.2, 21.0, 20.9, 20.9, 20.9, 16.7, 16.7, 16.7

Note: Signals are doubled due to the presence of diastereomers.

IR (neat, ν cm^{-1}): 3060, 3029, 2926, 2882, 1710, 1447, 1270, 1111, 732

HRMS (ESI+) m/z calcd for $C_{34}H_{36}{}^{35}ClO_3$ $[M+H]^+$ 527.2347; found 527.2331

Isopropyl 2-(4-(4-chloro-2-(4-chlorophenyl)-5-phenyl-1-oxaspiro[2.3]hex-4-en-2-yl)phenoxy)-2-methylpropanoate, 2u

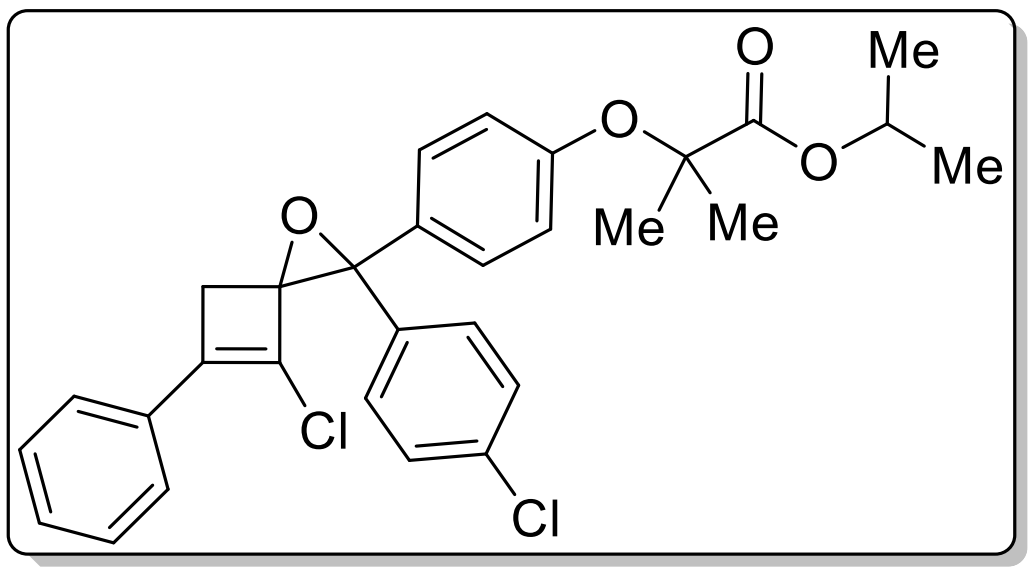

Prepared following **General Procedure 2** using *isopropyl 2-(4-((4-chlorophenyl)(hydroxy)(2-phenylcycloprop-1-en-1-yl)methyl)phenoxy)-2-methylpropanoate* **1w** (90 mg, 0.19 mmol). The crude material was obtained as 1:1 mixture of diastereomers. Purification by flash column chromatography (95:5 hexane:Et_2O) yielded the title product **2u** as a white solid (78 mg, 0.15 mmol, ca. 1:1 dr, 79%).

Data reported for mixture of diastereomers (d_1 + d_2)

^{1}H-NMR (400 MHz, $CDCl_3$): δ_H 7.63 – 7.56 (2x2H, m, *d_1 + d_2*), 7.51 – 7.44 (2H, m, *d_2*), 7.42 – 7.34 (2x5H, m, *d_1 + d_2*), 7.32 – 7.23 (4H, m, *d_1*), 7.21 – 7.14 (2H, m, *d_2*), 6.92 – 6.85 (2H, m, *d_1*), 6.85 – 6.77 (2H, m, *d_2*), 5.09 (2x1H, m, *d_1 + d_2*), 3.04 (1H, d, *J* = 11.6 Hz) and 2.91 (1H, d, *J* = 11.6 Hz) (*AB syst., d_2*), 3.01 (1H, d, *J* = 11.5 Hz) and 2.84 (1H, d, *J* = 11.5 Hz) (*AB syst., d_1*), 1.62 (6H, app. d, *J* = 2.5 Hz, *d_1*), 1.59 (6H, s, *d_2*), 1.22 (2x6H, m, *d_1 + d_2*).

^{13}C{^{1}H}-NMR (101 MHz, $CDCl_3$): δ_C 173.8, 173.6, 155.7, 155.6, 144.9, 144.8, 138.4, 135.6, 134.1, 133.8, 132.2, 131.6, 123.0, 129.6, 129.5, 128.7, 128.5, 128.4, 128.2, 127.7, 127.1, 118.7, 118.6, 118.5, 118.5, 79.3, 74.9, 69.1, 69.1, 67.6, 67.5, 36.4, 36.2, 25.5, 25.5, 25.5, 25.5, 21.7

Note: Some signals are doubled due to the presence of diastereomers.

IR (neat, ν cm^{-1}): 2928, 2932, 1727, 1610, 1489, 1383, 1177, 1101, 1014, 908

HRMS (ESI+): m/z calcd for $C_{30}H_{28}{}^{35}Cl_2O_4Na$ $[M+Na]^+$ 545.1257; found 545.1277

4-Bromo-2,2,5-triphenyl-1-oxaspiro[2.3]hex-4-ene, 3a

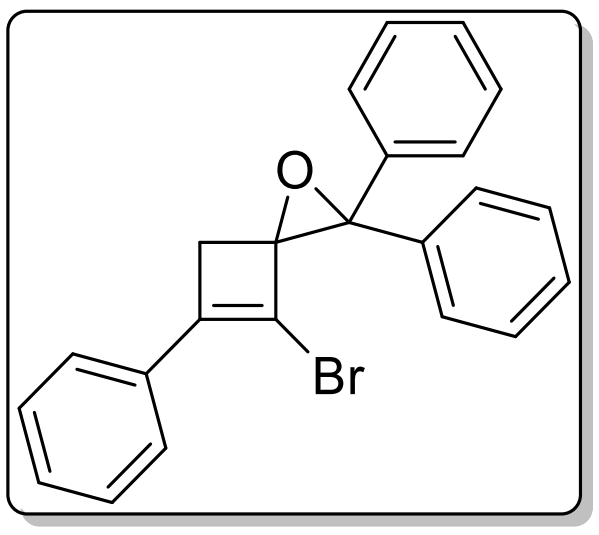

Prepared following **General Procedure 3** using *diphenyl(2-phenylcycloprop-1-en-1-yl)methanol* **1a** (59 mg, 0.20 mmol). Purification by flash column chromatography (98:2 hexane:EtOAc) yielded the title product **3a** as a white solid (43 mg, 0.11 mmol, 55%).

^{1}H-NMR (400 MHz, $CDCl_3$): δ_H 7.72 – 7.65 (2H, m), 7.60 – 7.53 (2H, m), 7.43 – 7.28 (11H, m), 3.15 (1H, d, *J* = 11.5 Hz) and 3.04 (1H, d, *J* = 11.4 Hz) (*AB syst.*)

^{13}C{^{1}H}-NMR (101 MHz, $CDCl_3$): δ_C 148.6, 139.6, 136.6, 132.0, 129.8, 128.8, 128.7, 128.4, 128.2, 128.2, 127.9, 126.8, 126.8, 107.4, 74.6, 68.5, 38.1

IR (neat, ν cm^{-1}): 3059, 3028, 2924, 1599, 1491, 1447, 1202, 1077, 900, 761

HRMS (ESI+): m/z calcd for $C_{23}H_{17}{}^{79}BrONa$ $[M+Na]^+$ 411.0355; found 411.0351

4-Bromo-5-phenyl-2,2-di-*p*-tolyl-1-oxaspiro[2.3]hex-4-ene, 3b

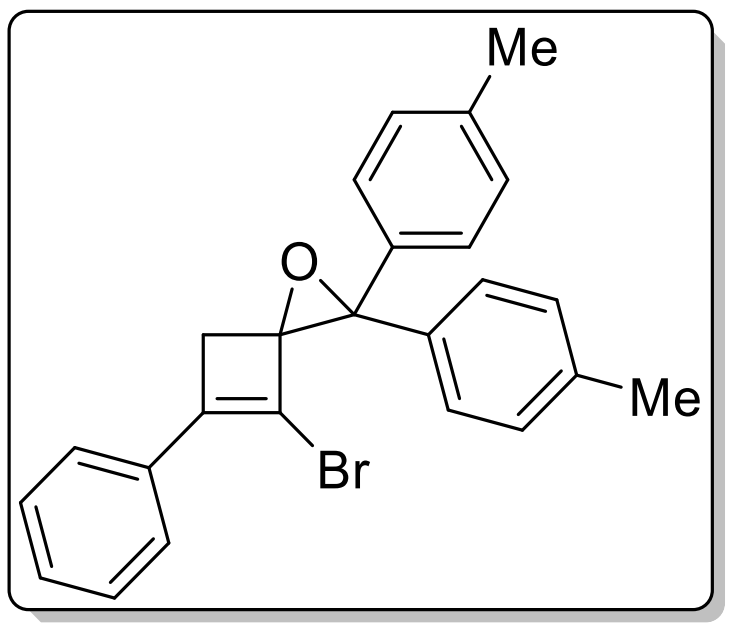

Prepared following **General Procedure 3** using *(2-phenylcycloprop-1-en-1-yl)di-p-tolylmethanol* **1b** (60 mg, 0.19 mmol). Purification by flash column chromatography (98:2 hexane:EtOAc) yielded the title product **3b** as a colourless oil (17 mg, 0.042 mmol, 21%).

^{1}H-NMR (400 MHz, $CDCl_3$): δ_H 7.71 – 7.66 (2H, m), 7.45 – 7.36 (5H, m), 7.22 (4H, dd, *J* = 12.8, 8.0 Hz), 7.13 (2H, d, *J* = 8.0 Hz), 3.11 (1H, d, *J* = 11.4 Hz) and 3.01 (1H, d, *J* = 11.4 Hz) (*AB syst.*), 2.39 (3H, s), 2.33 (3H, s)

^{13}C{^{1}H}-NMR (101 MHz, $CDCl_3$): δ_C 148.4, 137.8, 137.5, 137.0, 133.8, 132.1, 129.7, 129.0, 128.8, 128.7, 128.6, 126.8, 126.8, 107.7, 74.6, 68.5, 38.1, 21.5, 21.2

IR (neat, ν cm^{-1}): 3025, 2921, 1715, 1609, 1510, 1445, 1294, 1018, 910

HRMS (ESI-): m/z calcd for $C_{25}H_{20}{}^{79}BrO$ $[M-H]^-$ 415.0703; found 415.0707

4-Bromo-2,2-bis(4-fluorophenyl)-5-phenyl-1-oxaspiro[2.3]hex-4-ene, 3c

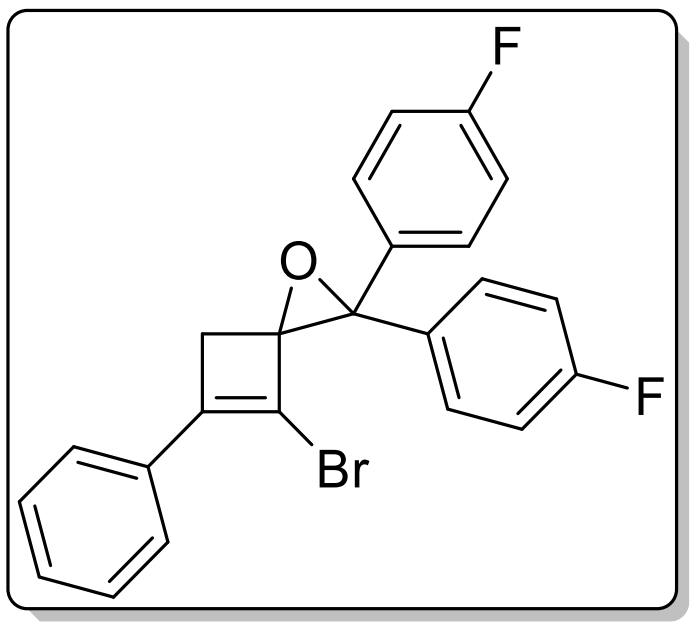

Prepared following **General Procedure 3** using *bis(4-fluorophenyl)(2-phenylcycloprop-1-en-1-yl)methanol* **1c** (63 mg, 0.19 mmol). Purification by flash column chromatography (hexane) yielded the title product **3c** as a yellow oil (35 mg, 0.082 mmol, 43%).

^{1}H-NMR (400 MHz, $CDCl_3$): δ_H 7.59 (2H, dd, *J* = 6.6, 2.9 Hz), 7.47 – 7.39 (2H, m), 7.32 – 7.27 (3H, m), 7.24 – 7.20 (2H, m), 7.01 (2H, t, *J* = 8.7 Hz), 6.94 (2H, t, *J* = 8.7 Hz), 3.03 (d, *J* = 11.4 Hz, 1H) and 2.90 (d, *J* = 11.4 Hz, 1H) (*AB syst.*)

^{19}F-NMR (377 MHz, $CDCl_3$): -113.26 (m), -114.26 (m)

^{13}C{^{1}H}-NMR (101 MHz, $CDCl_3$): δ_C 162.8 (d, *J* = 247.0 Hz), 162.6 (d, *J* = 247.3 Hz), 148.8, 135.3 (d, *J* = 3.0 Hz), 132.3 (d, *J* = 3.3 Hz), 131.8, 130.6 (d, *J* = 8.3 Hz), 129.9, 128.7, 128.6 (d, *J* = 8.2 Hz), 126.8, 115.4 (d, *J* = 21.6 Hz), 115.3 (d, *J* = 21.7 Hz), 106.9, 74.6, 67.5, 37.9

IR (neat, ν cm^{-1}): 3063, 2924, 1604, 1507, 1297, 1221, 1155, 1093, 909, 828, 762

HRMS (ESI-): m/z calcd for $C_{23}H_{14}{}^{79}BrF_2O$ $[M-H]^-$ 423.0202; found 423.0202

4-Bromo-2,2-bis(4-chlorophenyl)-5-phenyl-1-oxaspiro[2.3]hex-4-ene, 3d

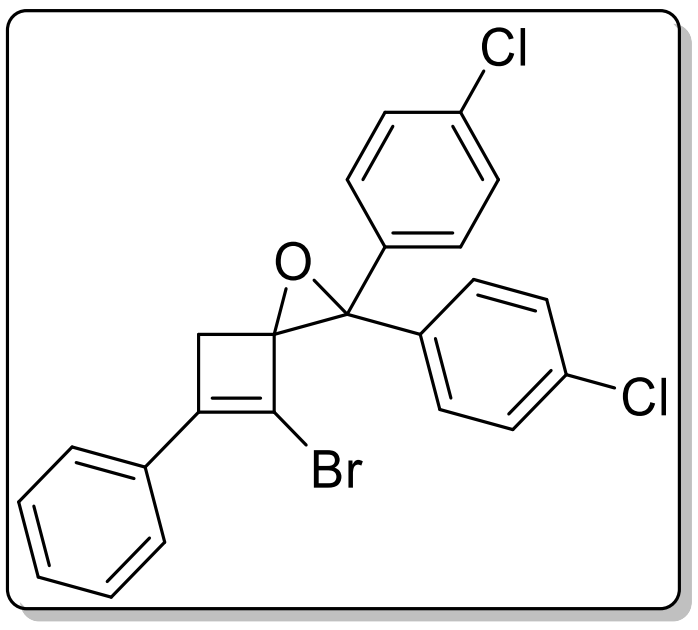

Prepared following **General Procedure 3** using *bis(4-chlorophenyl)(2-phenylcycloprop-1-en-1-yl)methanol* **1d** (73 mg, 0.20 mmol). Purification by flash column chromatography (hexane) yielded the title product **3d** as a white crystallin solid (39 mg, 0.086 mmol, 43%).

^{1}H-NMR (400 MHz, $CDCl_3$): δ_H 7.70 – 7.65 (m, 2H), 7.52 – 7.46 (2H, m), 7.42 – 7.37 (5H, m), 7.34 – 7.25 (4H, m), 3.12 (1H, d, *J* = 11.5 Hz) and 2.99 (1H, d, *J* = 11.5 Hz) (*AB syst.*)

^{13}C{^{1}H}-NMR (101 MHz, $CDCl_3$): δ_C 149.0, 137.7, 134.7, 134.4, 134.1, 131.7, 130.2, 130.0, 128.7, 128.7, 128.5, 128.1, 126.8, 106.7, 74.6, 67.4, 37.9

IR (neat, ν cm^{-1}): 3062, 2924, 1597, 1488, 1398, 1256, 1128, 1090, 906, 816

HRMS (ESI+): m/z calcd for $C_{23}H_{15}{}^{79}Br^{35}Cl_2ONa$ $[M+Na]^+$ 478.9581; found 478.9572

Melting point 128.7 – 145.5 °C

4-Bromo-2-(3-bromophenyl)-2,5-diphenyl-1-oxaspiro[2.3]hex-4-ene, 3e

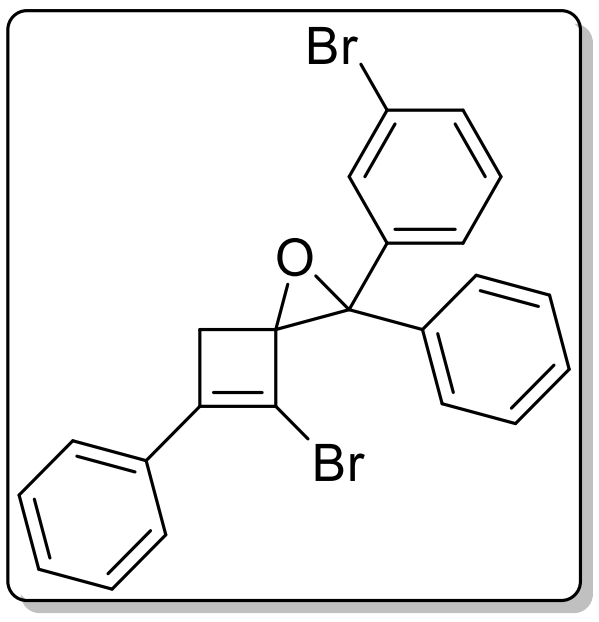

Prepared following **General Procedure 3** using *(3-bromophenyl)(phenyl)(2-phenylcycloprop-1-en-1-yl)methanol* **1e** (75 mg, 0.20 mmol). Purification by flash column chromatography (hexane to 98:2 hexane:Et_2O) yielded the title product **3e** as a colourless oil (52 mg, 0.11 mmol, ca. 1:1 dr, 56%).

Data reported for mixture of diastereomers (d_1 + d_2)

^{1}H-NMR (400 MHz, $CDCl_3$): δ_H 7.74 (1H, t, *J* = 1.9 Hz, d_1), 7.71 – 7.67 (4H, m, $d_1 + d_2$), 7.55 – 7.52 (4H, m, $d_1 + d_2$), 7.50 (1H, t, *J* = 1.9 Hz, d_2), 7.45 – 7.38 (10H, m, $d_1 + d_2$), 7.38 – 7.30 (6H, m, $d_1 + d_2$), 7.32 – 7.24 (1H, m, d_1), 7.20 (1H, t, *J* = 7.9 Hz, d_2), 3.13 (1H, d, *J* = 11.5 Hz, d_2) and 3.02 (1H, d, *J* = 11.5 Hz, d_2) (*AB syst.*), 3.12 (1H, d, *J* = 11.5 Hz, d_1) and 3.00 (1H, d, *J* = 11.4 Hz, d_1) (*AB syst.*)

^{13}C{^{1}H}-NMR (101 MHz, $CDCl_3$): δ_C 148.9, 148.9, 142.0, 138.9, 138.8, 135.9, 131.8, 131.4, 131.0, 129.9, 129.9, 129.8, 129.7, 128.8, 128.7, 128.5, 128.3, 128.1, 127.5, 126.8, 126.7, 125.5, 122.7, 122.3, 107.0, 106.8, 74.5, 67.8, 38.0, 37.9

Note: Some signals are doubled due to the presence of diastereomers.

IR (neat, ν cm^{-1}): 3061, 3028, 2924, 1565, 1446, 1265, 1203, 936

HRMS (ESI+): m/z calcd for $C_{23}H_{17}{}^{79}Br_2O$ $[M+H]^+$ 466.9641; found 466.9641

4-Bromo-2,5-diphenyl-2-(4-(*p*-tolylthio)phenyl)-1-oxaspiro[2.3]hex-4-ene, 3f

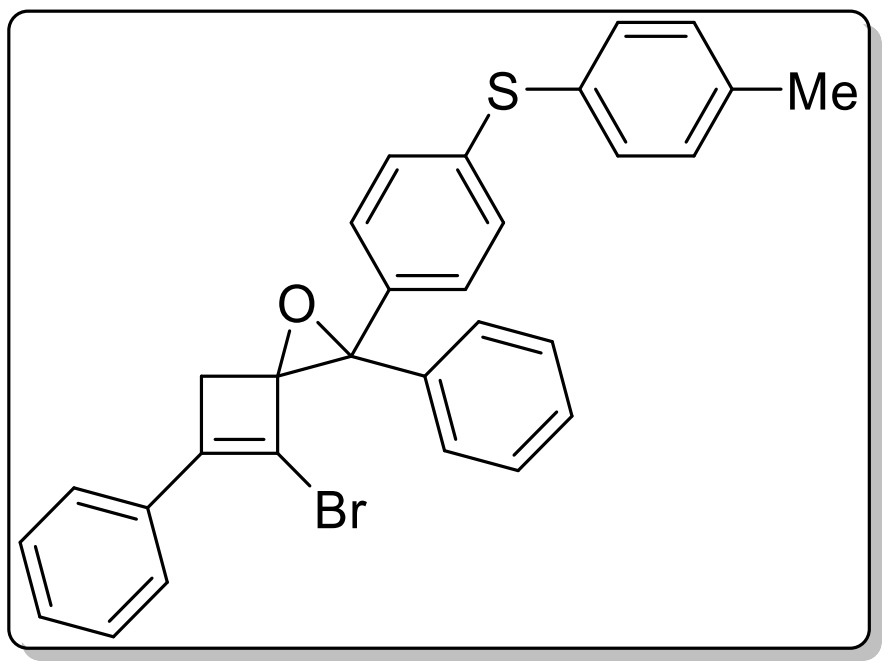

Prepared following **General Procedure 3** using *phenyl(2-phenylcycloprop-1-en-1-yl)(4-(p-tolylthio)phenyl)methanol* **1f** (80 mg, 0.19 mmol). Purification by flash column chromatography (hexane to 98:2 hexane:Et_2O) yielded the title product **3f** as a yellow solid (56 mg, 0.11 mmol, ca. 1:1 dr, 58%).

Data reported for mixture of diastereomers (d_1 + d_2)

^{1}H-NMR (400 MHz, $CDCl_3$): δ_H 7.73 – 7.64 (4H, m, $d_1 + d_2$), 7.57 – 7.50 (2H, m, $d_1 + d_2$), 7.52 – 7.44 (2H, m, $d_1 + d_2$), 7.42 – 7.36 (10H, m, $d_1 + d_2$), 7.36 – 7.28 (10H, m, $d_1 + d_2$), 7.25 – 7.12 (8H, m, $d_1 + d_2$), 3.12 (1H, d, *J* = 11.5 Hz, d_2) and 3.03 (1H, d, *J* = 11.4 Hz, d_2) (*AB syst.*), 3.12 (1H, d, *J* = 11.5 Hz, d_1) and 3.02 (1H, d, *J* = 11.5 Hz, d_1) (*AB syst.*), 2.36 (2x3H, s, $d_1 + d_2$)

^{13}C{^{1}H}-NMR (101 MHz, $CDCl_3$): δ_C 148.7, 148.6, 139.3, 138.2, 137.8, 137.6, 137.5, 137.2, 136.3, 134.9, 133.1, 132.4, 131.9, 131.9, 131.2, 130.3, 130.2, 129.8, 129.5, 129.4, 129.2, 128.9, 128.8, 128.7, 128.4, 128.4, 128.3, 128.2, 127.9, 127.5, 126.8, 107.3, 107.1, 74.6, 68.2, 68.2, 38.1, 38.0, 21.3, 21.3

Note: Some signals are doubled due to the presence of diastereomers.

IR (neat, ν cm^{-1}): 3058, 3025, 3922, 2865, 1596, 1489, 1446, 1211, 921

HRMS (ESI+): m/z calcd for $C_{30}H_{23}{}^{79}BrOSNa$ $[M+Na]^+$ 533.0545; found 533.0550

4-Bromo-5-(4-chlorophenyl)-2-phenyl-2-(trifluoromethyl)-1-oxaspiro[2.3]hex-4-ene, 3g

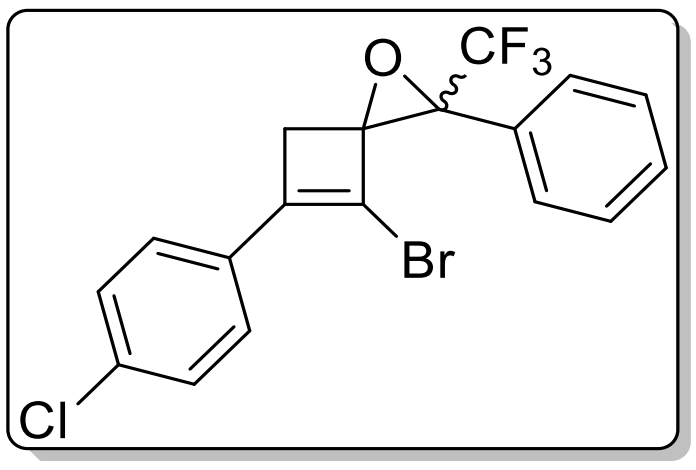

Prepared following **General Procedure 3** using 1-(2-(4-chlorophenyl)cycloprop-1-en-1-yl)-2,2,2-trifluoro-1-phenylethan-1-ol (63 mg, 0.19 mmol). The crude compound was obtained as a 60:40 mixture of diastereomers. Purification by flash column chromatography (hexane) yielded the title product **3g** as a white solid (29 mg, 0.072 mmol, 37%, > 99:1 dr, major diastereomer).

Data reported for major diastereomer

^{1}H-NMR (400 MHz, $CDCl_3$): δ_H 7.68 – 7.60 (2H, m), 7.54 – 7.50 (2H, m), 7.45 – 7.41 (3H, m), 7.40 – 7.36 (2H, m), 2.79 (1H, d, *J* = 11.8 Hz) and 2.67 (1H, d, *J* = 11.8 Hz) (*AB syst.*)

^{19}F-NMR (377 MHz, $CDCl_3$): -66.09 (s)

^{13}C{^{1}H}-NMR (101 MHz, $CDCl_3$): δ_C 149.8, 136.3, 131.6, 130.0, 129.5, 129.1, 128.8, 128.3, 126.7, 123.7 (q, *J* = 279.2 Hz), 104.8, 73.1, 64.4 (q, *J* = 38.3 Hz), 38.0

IR (neat, ν cm^{-1}): 3066, 2926, 2854, 1587, 1487, 1453, 1182, 1156, 1092, 937

HRMS (ESI+): *m/z* calcd for $C_{18}H_{11}{}^{79}Br^{35}ClF_3ONa$ 436.9526 $[M+Na]^+$; found 436.9520

1-Bromo-2-phenyl-11-oxadispiro[3.0.5^5.1^4]undec-1-ene, 3h

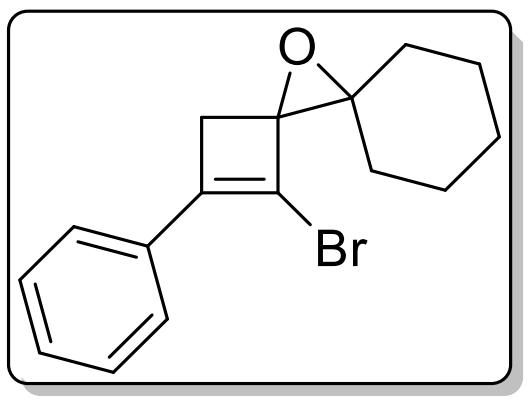

Prepared following **General Procedure 3** using *(1-(2-phenylcycloprop-1-en-1-yl)cyclohexan-1-ol* **1i** (46 mg, 0.20 mmol). Purification by flash column chromatography (hexane) yielded the title product **3h** as a colourless oil (33 mg, 0.11 mmol, 58%).

^{1}H-NMR (400 MHz, $CDCl_3$): δ_H 7.75 – 7.71 (2H, m), 7.48 – 7.32 (3H, m), 3.16 (1H, d, J = 11.1 Hz) and 2.98 (1H, d, J = 11.1 Hz) (*AB syst.*), 2.10 – 1.99 (1H, m), 1.98 – 1.91 (1H, m), 1.88 – 1.73 (2H, m), 1.67 – 1.58 (3H, m), 1.56 – 1.50 (3H, m).

^{13}C{^{1}H}-NMR (101 MHz, $CDCl_3$): δ_C 147.2 132.1, 129.6, 128.7, 126.7, 106.9, 72.8, 67.2, 37.3, 33.6, 30.5, 25.5, 25.0, 24.9

IR (neat, ν cm^{-1}): 2929, 2854, 1532, 1487, 1466, 1247, 1182, 917

HRMS (ESI+): m/z calcd for $C_{16}H_{18}{}^{79}BrO$ $[M+H]^+$ 305.0536; found 305.0531

***tert*-Butyl 1-bromo-2-phenyl-11-oxa-8-azadispiro[3.0.5^5.1^4]undec-1-ene-8-carboxylate, 3i**

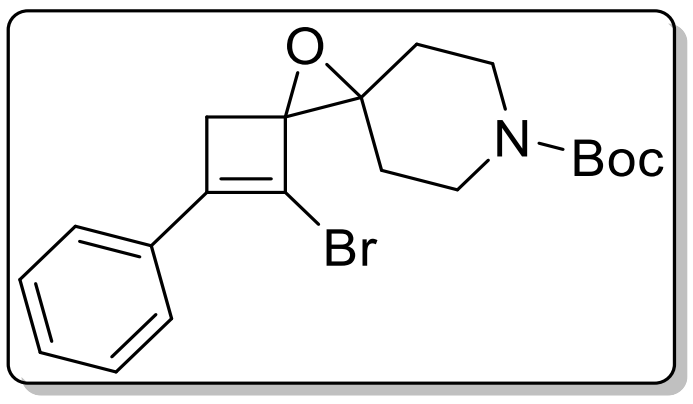

Prepared following **General Procedure 3** using *tert-butyl 4-hydroxy-4-(2-phenylcycloprop-1-en-1-yl)piperidine-1-carboxylate* **1j** (65 mg, 0.20 mmol). Purification by flash column chromatography (hexane to 98:2 hexane:Et_2O) yielded the title product **3i** as a yellow oil (53 mg, 0.13 mmol, 61%).

^{1}H-NMR (400 MHz, $CDCl_3$): δ_H 7.77 – 7.66 (2H, m), 7.48 – 7.34 (3H, m), 3.77 – 3.68 (2H, m), 3.49 (2H, ddd, *J* = 13.2, 9.3, 3.8 Hz), 3.15 (1H, d, *J* = 11.2 Hz) and 3.02 (1H, d, *J* = 11.2 Hz) (*AB syst.*), 2.11 – 2.00 (2H, m), 1.84 (1H, ddd, *J* = 13.6, 9.2, 4.5 Hz), 1.58 – 1.52 (1H, m), 1.47 (9H, s)

^{13}C{^{1}H}-NMR (101 MHz, $CDCl_3$): δ_C 154.9, 147.7, 131.9, 129.8, 128.7, 126.8, 106.1, 80.0, 72.5, 65.0, 42.5, 42.2, 37.3, 32.7, 30.4, 28.6

IR (neat, ν cm^{-1}): 2974, 2929, 2866, 1693, 1447, 1421, 1231, 1169, 1058, 993

HRMS (ESI+): m/z calcd for $C_{20}H_{25}{}^{79}BrNO_3$ $[M+H]^+$ 406.1012; found 406.1011

1-Bromo-2,8-diphenyl-11-oxadispiro[3.0.5^5.1^4]undec-1-ene, 3j

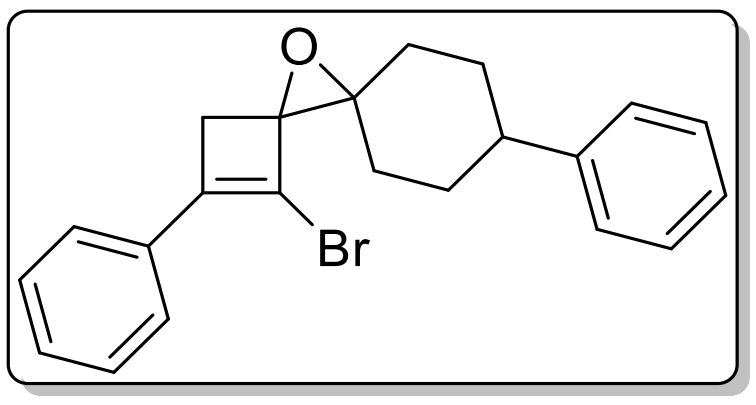

Prepared following **General Procedure 3** using *4-phenyl-1-(2-phenylcycloprop-1-en-1-yl)cyclohexan-1-ol* **1k** (56 mg, 0.19 mmol). The crude material was obtained as a single diastereomer. Purification by flash column chromatography (hexane to 98:2 hexane:Et_2O) yielded the title product **3j** as a colourless oil (31 mg, 0.083 mmol, 42%).

^{1}H-NMR (400 MHz, $CDCl_3$): δ_H 7.83 – 7.73 (2H, m), 7.50 – 7.37 (3H, m), 7.33 – 7.25 (2H, m), 7.26 – 7.15 (3H, m), 3.25 (1H, d, *J* = 11.2 Hz) and 3.05 (1H, d, *J* = 11.3 Hz) (AB syst.), 2.68 (1H, tt, *J* = 12.0, 3.3 Hz), 2.43 – 2.33 (1H, m), 2.16 – 1.98 (4H, m), 1.75 – 1.58 (3H, m)

^{13}C{^{1}H}-NMR (101 MHz, $CDCl_3$): δ_C 147.4, 145.9, 132.1, 129.7, 128.7, 128.6, 126.9, 126.8, 126.4, 106.7, 72.7, 67.0, 43.4, 37.3, 33.9, 33.4, 33.3, 30.5

IR (neat, ν cm^{-1}): 3028, 2925, 2855, 1527, 1492, 1234, 1051, 967

HRMS (ESI+): *m/z* calcd for $C_{22}H_{22}{}^{79}BrO$ $[M+H]^+$ 381.0849; found 381.0849

4-Bromo-2,2-bis(4-chlorophenyl)-5-phenyl-1-oxaspiro[2.3]hex-4-ene, 3k

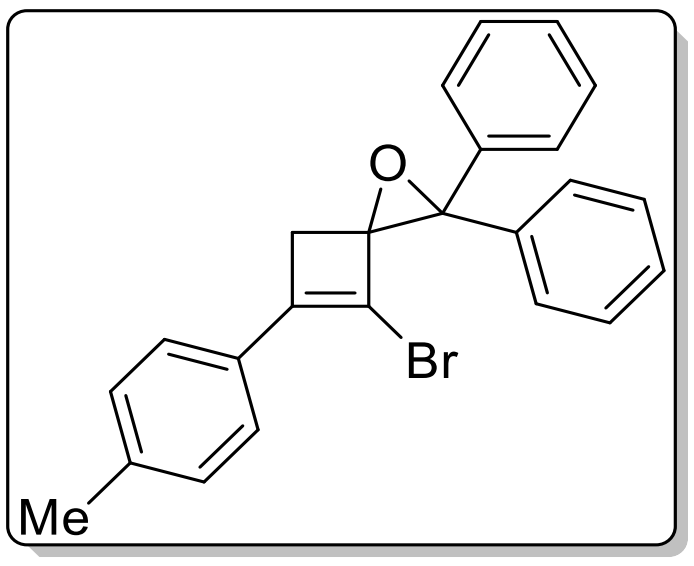

Prepared following **General Procedure 3** *(2-phenylcycloprop-1-en-1-yl)di-p-tolylmethanol* **1l** (63 mg, 0.20 mmol). Purification by flash column chromatography (98:2 hexane:EtOAc) yielded the title product **3k** as a white solid (38 mg, 0.081 mmol, 41%).

^{1}H-NMR (400 MHz, $CDCl_3$): δ_H 7.64 – 7.53 (4H, m), 7.46 – 7.26 (8H, m), 7.19 (2H, d, *J* = 7.9 Hz), 3.11 (1H, d, *J* = 11.5 Hz) and 3.00 (1H, d, *J* = 11.4 Hz) (*AB syst.*), 2.36 (s, 3H)

^{13}C{^{1}H}-NMR (101 MHz, $CDCl_3$): δ_C 148.5, 139.9, 139.5, 136.5, 129.2, 128.7, 128.2, 128.0, 128.0, 127.7, 126.7, 126.6, 105.8, 74.5, 68.3, 37.9, 21.6

IR (neat, ν cm^{-1}): 3029, 2971, 2853, 1642, 1446, 1374, 1306, 1114, 962

HRMS (ESI+): m/z calcd for $C_{24}H_{19}{}^{79}BrONa$ $[M+Na]^+$ 425.0517; found 425.0571

4-Bromo-2,2-bis(4-chlorophenyl)-5-(*p*-tolyl)-1-oxaspiro[2.3]hex-4-ene, 3l

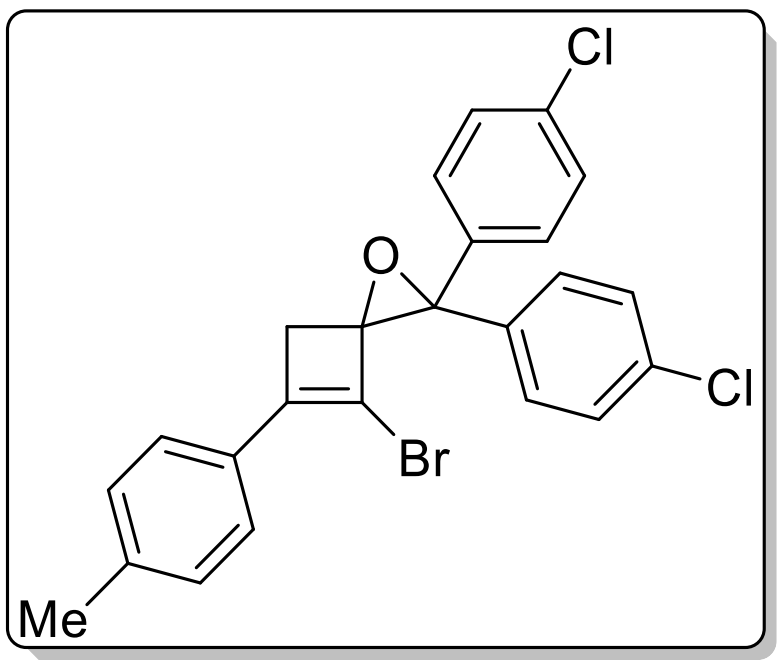

Prepared following **General Procedure 3** using *bis(4-chlorophenyl)(2-(p-tolyl)cycloprop-1-en-1-yl)methanol* **1m** (76 mg, 0.20 mmol). Purification by flash column chromatography (hexane) yielded the title product **3l** as a white solid (34 mg, 0.073 mmol, 36%).

^{1}H-NMR (400 MHz, $CDCl_3$): δ_H 7.56 (2H, d, *J* = 8.1 Hz), 7.50 – 7.45 (2H, m), 7.41 – 7.36 (2H, m), 7.32 – 7.29 (2H, m), 7.27 – 7.24 (2H, m), 7.20 (2H, d, *J* = 7.9 Hz), 3.08 (1H, d, *J* = 11.5 Hz) and 2.95 (1H, d, *J* = 11.5 Hz) (*AB syst.*), 2.36 (3H, s)

^{13}C{^{1}H}-NMR (101 MHz, $CDCl_3$): δ_C 149.0, 140.4, 137.8, 134.8, 134.4, 134.1, 130.2, 129.4, 129.1, 128.7, 128.5, 128.1, 126.8, 105.3, 74.7, 67.4, 37.9, 21.8

IR (neat, v cm^{-1}): 3027, 2922, 2855, 1598, 1490, 1399, 1281, 1091, 1015, 816

HRMS (ESI+): m/z calcd for $C_{24}H_{17}{}^{79}Br^{35}Cl_2ONa$ $[M+Na]^+$ 492.9738; found 492.9822

4-Bromo-5-isopentyl-2,2-diphenyl-1-oxaspiro[2.3]hex-4-ene, 3m

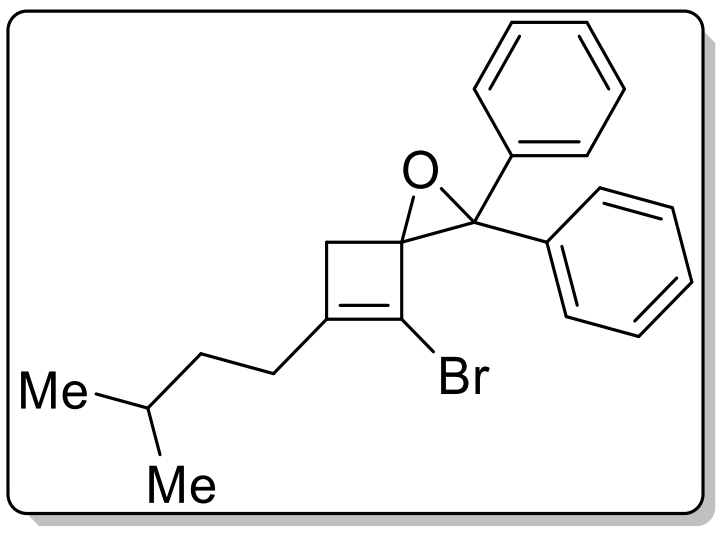

Prepared following **General Procedure 3** using *(2-isopentylcycloprop-1-en-1-yl)diphenylmethanol* **1o** (58 mg, 0.20 mmol). Purification by flash column chromatography (hexane to 98:2 hexane:Et_2O) yielded the title product **3m** as a yellow oil (47 mg, 0.12 mmol, 61%).

^{1}H-NMR (400 MHz, $CDCl_3$): δ_H 7.55 – 7.47 (2H, m), 7.42 – 7.32 (4H, m), 7.33 – 7.24 (4H, m), 2.78 (1H, d, *J* = 11.8 Hz) and 2.64 (1H, d, *J* = 11.8 Hz) (*AB syst.*), 2.33 – 2.14 (2H, m), 1.62 – 1.51 (1H, m – *overlapping with residual water peak*), 1.44 – 1.34 (2H, m), 0.91 (3H, d, *J* = 2.1 Hz), 0.90 (3H, d, *J* = 2.1 Hz)

^{13}C{^{1}H}-NMR (101 MHz, $CDCl_3$): δ_C 156.1, 139.8, 136.8, 128.8, 128.2, 128.1, 127.7, 126.8, 108.0, 75.1, 67.9, 39.6, 35.2, 27.9, 26.6, 22.5, 22.4

IR (neat, ν cm^{-1}): 3029, 2955, 2925, 2869, 1449, 1395, 1194, 961

HRMS (ESI+): m/z calcd for $C_{22}H_{24}{}^{79}BrO$ $[M+H]^+$ 383.1005; found 383.1004

4-Bromo-2,2-diphenyl-5-(3-phenylpropyl)-1-oxaspiro[2.3]hex-4-ene, 3n

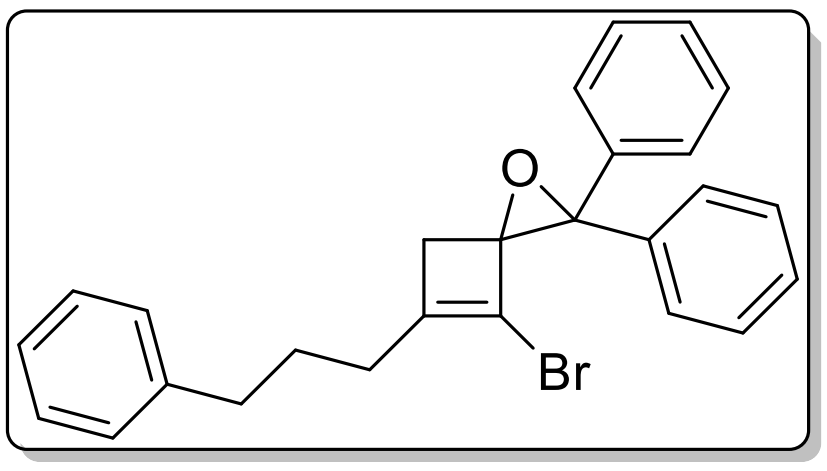

Prepared following **General Procedure 3** using *diphenyl(2-(3-phenylpropyl)cycloprop-1-en-1-yl)methanol* **1q** (68 mg, 0.20 mmol). Purification by flash column chromatography (hexane) yielded the title product **3n** as a white solid (56 mg, 0.13 mmol, 65%).

^{1}H-NMR (400 MHz, $CDCl_3$): δ_H 7.85 – 7.78 (1H, m), 7.63 – 7.57 (1H, m), 7.55 – 7.45 (3H, m), 7.42 – 7.34 (3H, m), 7.34 – 7.25 (5H, m), 7.22 – 7.15 (2H, m), 2.79 (d, *J* = 11.8 Hz, 1H), 2.65 (td, *J* = 8.1, 7.6, 2.3 Hz, 3H), 2.34 – 2.19 (2H, m), 1.84 (2H, p, *J* = 7.5 Hz)

^{13}C{^{1}H}-NMR (101 MHz, $CDCl_3$): δ_C 155.3, 141.7, 139.9, 136.8, 130.2, 132.6, 128.7, 128.6, 128.5, 128.4, 128.3, 128.1, 127.7, 126.8, 126.1, 108.7, 75.0, 67.9, 39.6, 35.7, 28.2, 28.0

IR (neat, v cm^{-1}): 3026, 2929, 2857, 1527, 1494, 1322, 1277, 1067, 988

HRMS (ESI+): m/z calcd for $C_{26}H_{24}{}^{79}BrO$ $[M+H]^+$ 431.1005; found 431.1004

4-Bromo-2,2-dimethyl-5-(3-phenylpropyl)-1-oxaspiro[2.3]hex-4-ene, 3o

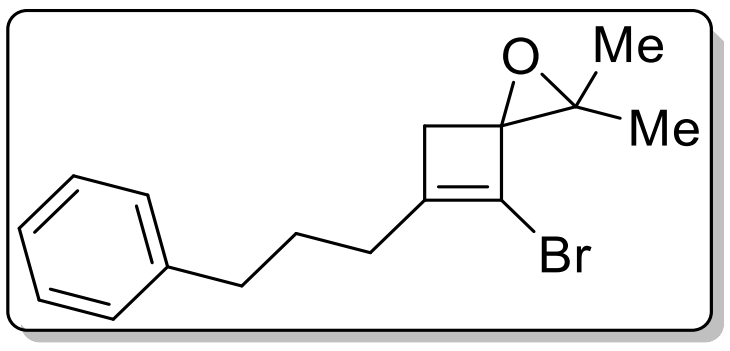

Prepared following **General Procedure 3** using *2-(2-(3-phenylpropyl)cycloprop-1-en-1-yl)propan-2-ol* **1r** (43 mg, 0.20 mmol). Purification by flash column chromatography (hexane) yielded the title product **3o** as a white solid (35 mg, 0.11 mmol, 57%).

^{1}H-NMR (400 MHz, $CDCl_3$): δ_H 7.32 – 7.27 (2H, m), 7.23 – 7.17 (3H, m), 2.76 (1H, d, *J* = 11.5 Hz), 2.70 – 2.64 (2H, m), 2.62 (1H, d, *J* = 11.4 Hz), 2.34 – 2.22 (2H, m), 1.87 (2H, p, *J* = 7.7 Hz), 1.54 (3H, s), 1.32 (3H, s)

^{13}C{^{1}H}-NMR (101 MHz, $CDCl_3$): δ_C 153.7, 141.8, 128.6, 128.5, 126.1, 108.2, 73.4, 62.2, 39.0, 35.7, 28.1, 28.0, 23.1, 20.3

IR (neat, ν cm^{-1}): 3026, 2925, 2857, 1736, 1495, 1453, 1375, 1197, 1030, 995, 916

HRMS (ESI+): m/z calcd for $C_{16}H_{20}{}^{79}BrO$ $[M+H]^+$ 307.0692; found 307.0654

tert-Butyl 2''-bromo-3''-phenyl-8-azadispiro[bicyclo[3.2.1]octane-3,2'-oxirane-3',1''-cyclobutan]-2''-ene-8-carboxylate, 3p

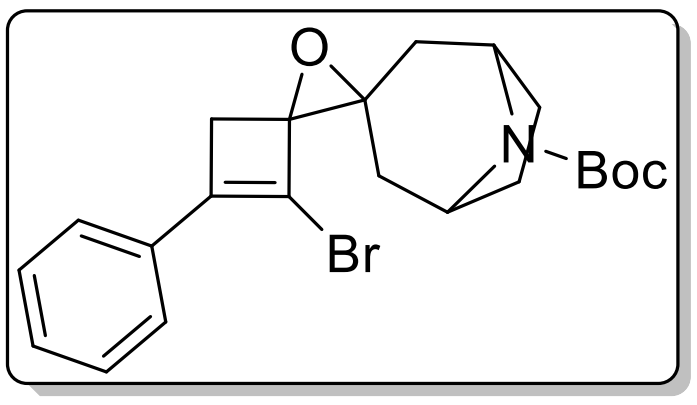

Prepared following **General Procedure 3** using *2-phenyl-4-(2-phenylcycloprop-1-en-1-yl)chroman-4-ol* **1s** (67 mg, 0.20 mmol). Purification by flash column chromatography (hexane to 9:1 hexane:EtOAc) yielded the title product **3p** as a white solid (54 mg, 0.12 mmol, 62%).

^{1}H-NMR (400 MHz, $CDCl_3$): δ_H 7.75 – 7.66 (2H, m), 7.46 – 7.33 (3H, m), 4.40 – 4.16 (2H, m), 3.06 (1H, d, *J* = 11.1 Hz) and 2.95 (1H, d, *J* = 11.1 Hz) (*AB syst.*), 2.46 – 2.21 (3H, m), 2.19 – 2.00 (5H, m), 1.48 (9H, s)

^{13}C{^{1}H}-NMR (101 MHz, $CDCl_3$): δ_C 153.4, 147.7, 132.0, 129.7, 128.7, 126.7, 106.2, 79.6, 68.8, 62.9, 53.6, 53.0, 38.3, 36.9, 34.3, 28.7, 28.3, 27.6

IR (neat, ν cm^{-1}): 2975, 2923, 1691, 1391, 1245, 1144, 1065, 955

HRMS (ESI+): m/z calcd for $C_{22}H_{27}{}^{79}BrNO_3$ $[M+H]^+$ 432.1169; found 432.1165

*Note: Due to the low intensity of the signals from the vinyl carbons of the cyclobutene ring, a copy of the HMBC spectrum has been included in **Section 8** to support their chemical shift.*

2''-Bromo-2,3''-diphenyldispiro[chromane-4,2'-oxirane-3',1''-cyclobutan]-2''-ene, 3q

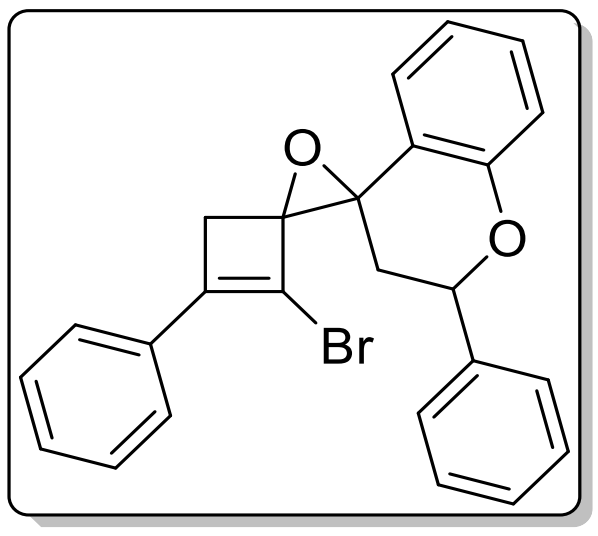

Prepared following **General Procedure 3** using *2-phenyl-4-(2-phenylcycloprop-1-en-1-yl)chroman-4-ol* **1t** (65 mg, 0.19 mmol). The crude material was obtained as 1:1 mixture of diastereomers. Purification by flash column chromatography (hexane to 95:5 hexane:EtOAc) yielded the title product **3q** as a yellow oil (35 mg, 0.084 mmol, ca. 1:1 dr, 43%).

Data reported for mixture of diastereomers ($d_1 + d_2$)

^{1}H-NMR (400 MHz, $CDCl_3$): δ_H 7.82 – 7.72 (2x2H, m, $d_1 + d_2$), 7.55 – 7.35 (17H, m, $d_1 + d_2$), 7.32 – 7.26 (2x1H, m, $d_1 + d_2$), 7.14 (1H, dd, J = 7.7, 1.8 Hz, d_2), 7.07 – 6.90 (2x2H, m, $d_1 + d_2$), 5.43 – 5.34 (2x1H, m, $d_1 + d_2$), 3.30 (1H, d, J = 11.2 Hz, d_1) and 3.13 (1H, d, J = 11.2 Hz, d_1) (*AB syst., d_1*), 3.25 (1H, d, J = 11.4 Hz, d_2) and 3.10 (1H, d, J = 11.5 Hz, d_2) (*AB syst., d_2*), 2.89 (1H, dd, J = 13.6, 2.1 Hz, d_2), 2.74 – 2.64 (2H, m, $d_1 + d_2$), 1.97 (1H, dd, J = 13.3, 2.1 Hz, d_1)

^{13}C{^{1}H}-NMR (101 MHz, $CDCl_3$): δ_C 157.0, 156.7, 149.1, 148.3, 140.4, 140.1, 131.8, 131.8, 130.1, 130.0, 129.9, 129.9, 128.9, 128.8, 128.7, 128.7, 128.4, 127.8, 127.0, 126.9, 126.3, 126.0, 125.1, 121.5, 120.8, 119.8, 118.8, 116.8, 116.2, 106.1, 105.1, 78.3, 77.8, 77.5, 74.9, 74.5, 63.3, 62.7, 38.1, 37.6, 37.5, 34.7

Note: Signals are doubled due to the presence of diastereomers.

IR (neat, ν cm^{-1}): 3031, 2957, 2922, 2853, 1608, 1487, 1226, 1226, 1050, 977

HRMS (ESI+): m/z calcd for $C_{25}H_{20}{}^{79}BrO_2$ $[M+H]^+$ 431.0641; found 431.0636

1,7,7-Trimethylbicyclo[2.2.1]heptan-2-yl 4-(6-bromo-2,5-diphenyl-1-oxaspiro[2.3]hex-4-en-2-yl)benzoate, 3r

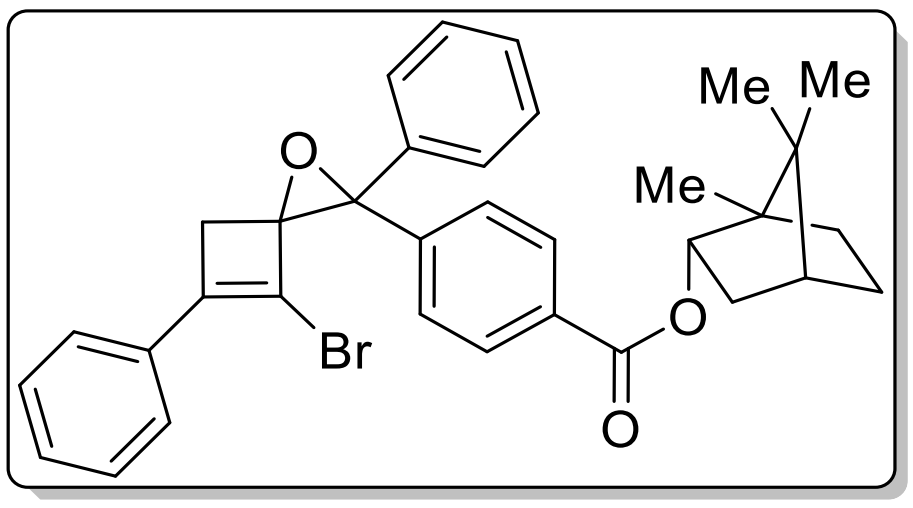

Prepared following **General Procedure 3** using *1,7,7-trimethylbicyclo[2.2.1]heptan-2-yl 4-(hydroxy(phenyl)(2-phenylcycloprop-1-en-1-yl)methyl)benzoate* **1u** (90 mg, 0.19 mmol). The crude material was obtained as 1:1 mixture of diastereomers. Purification by flash column chromatography (hexane to 95:5 hexane:Et_2O) yielded the title product **3r** as a white solid (63 mg, 0.11 mmol, ca. 1:1 dr, 58%).

Data reported for mixture of diastereomers ($d_1 + d_2$)

^{1}H-NMR (400 MHz, $CDCl_3$): δ_H 8.13 – 8.08 (2H, m, d_1), 8.04 – 8.00 (2H, m, d_2), 7.70 – 7.64 (6H, m, $d_1 + d_2$), 7.58 – 7.54 (2H, m, d_1), 7.45 -7.49 (2H, m, d_2), 7.44 – 7.29 (14H, m, $d_1 + d_2$), 5.16 – 5.08 (2x1H, m, $d_1 + d_2$), 3.15 (1H, d, *J* = 11.5 Hz) and 3.06 (1H, d, *J* = 11.5 Hz) (*AB syst., d_1*), 3.13 (1H, *J* = 11.4 Hz) and 2.97 (1H, d, *J* = 11.4 Hz) (*AB syst., d_2*), 2.52 – 2.43 (2x1H, m, $d_1 + d_2$), 2.19 – 2.07 (2x1H, m, $d_1 + d_2$), 1.87 – 1.78 (2x1H, m. $d_1 + d_2$), 1.77 – 1.73 (2x1H, m, $d_1 + d_2$), 1.47 – 1.38 (2x1H, m, $d_1 + d_2$), 1.36 – 1.30 (2x1H, m, $d_1 + d_2$), 1.17 – 1.07 (2x1H, m, $d_1 + d_2$), 0.98 (3H, s, d_1), 0.96 (3H, s, d_2), 0.94 – 0.91 (2x3H, m, $d_1 + d_2$), 0.91 – 0.87 (2x3H, m, $d_1 + d_2$)

^{13}C{^{1}H}-NMR (101 MHz, $CDCl_3$): δ_C 166.7, 166.6, 148.9, 148.8, 144.4, 141.5, 138.8, 135.9, 131.9, 130.8, 130.4, 129.9, 129.9, 129.6, 129.4, 128.8, 128.8, 128.7, 128.5, 128.3, 128.1, 126.8, 126.8, 126.8, 107.1, 106.9, 80.8, 80.8, 80.8, 74.6, 74.6, 68.3, 68.2, 49.3, 48.1, 45.2, 45.2, 38.2, 38.0, 37.1, 28.2, 27.6, 27.5, 19.9, 19.9, 19.1, 19.1, 13.8, 13.7

Note: Signals are doubled due to the presence of diastereomers.

IR (neat, ν cm^{-1}): 2954, 2879, 1713, 1611, 1447, 1300, 1271, 1116, 907

HRMS (ESI+): *m/z* calcd for $C_{34}H_{34}{}^{79}BrO_3$ $[M+H]^+$ 569.1686; found 569.1675

2-Isopropyl-5-methylcyclohexyl 4-(4-bromo-2,5-diphenyl-1-oxaspiro[2.3]hex-4-en-2-yl)benzoate, 3s

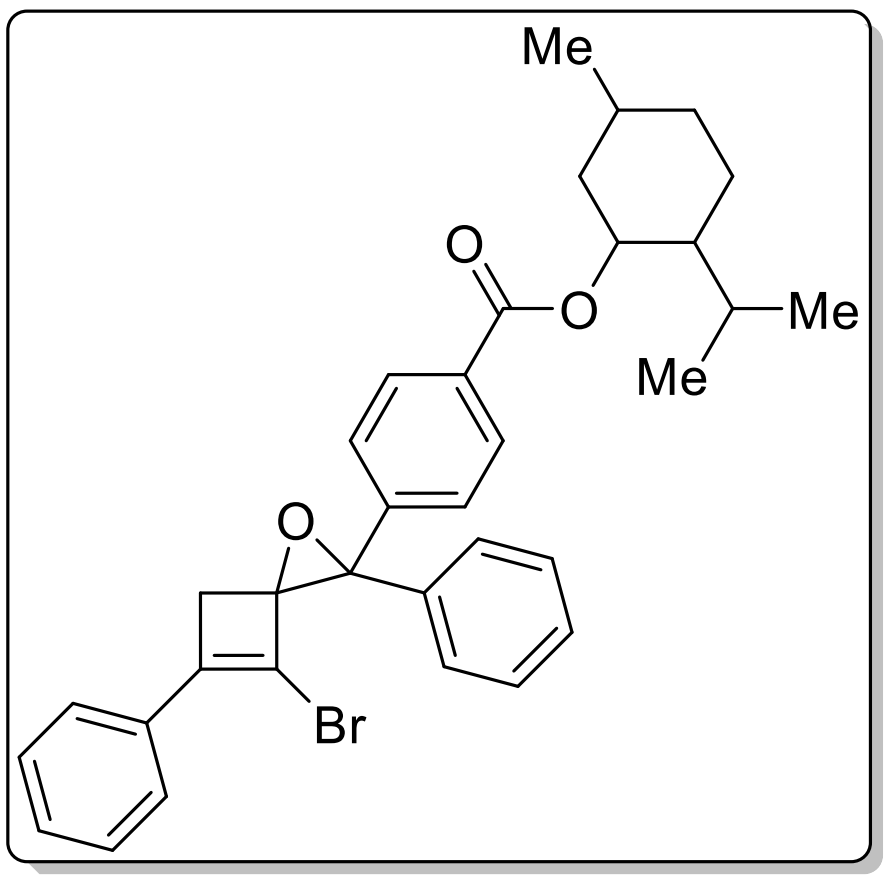

Prepared following **General Procedure 3** using *2-isopropyl-5-methylcyclohexyl 4-(hydroxy(phenyl)(2-phenylcycloprop-1-en-1-yl)methyl)benzoate* **1v** (100 mg, 0.20 mmol). The crude material was obtained as 1:1:1:1 mixture of diastereomers. Purification by flash column chromatography (hexane to 10:1 hexane:Et_2O) yielded the title product **3s** as a white solid (62 mg, 0.11 mmol, ca. 1:1:1:1 dr, 54%).

Data reported for mixture of diasteromers ($d_1 + d_2 + d_3 + d_4$)

^{1}H-NMR (400 MHz, $CDCl_3$): δ_H 8.19 – 8.14 (2x1H, m), 8.12 – 8.06 (2x2H, m), 8.04 – 7.99 (2x2H, m), 7.87 – 7.78 (2x2H, m), 7.71 – 7.63 (10H, m), 7.58 – 7.53 (2x2H, m), 7.48 – 7.34 (26H, m), 4.94 (1x4H, dtd, *J* = 12.8, 10.9, 4.4 Hz, $d_1 + d_2 + d_3 + d_4$), *[*3.17 – 3.09 (2x2H, m), 3.05 (2H, dd, *J* = 11.5, 1.2 Hz), 2.97 (2H, dd, *J* = 11.5, 0.8 Hz)*, $d_1 + d_2 + d_3 + d_4$]*, 2.18 – 2.09 (4X1H, m), 2.01 – 1.91 (4x1H, m), 1.79 – 1.69 (4x2H, m), 1.59 – 1.50 (4x2H, m), 1.19 – 1.05 (4x2H, m), 0.96 – 0.93 (2x6H, m), 0.93 – 0.89 (2x6H, m), 0.84 – 0.81 (2x3H, m), 0.81 – 0.77 (2X3H, m)

^{13}C{^{1}H}-NMR (101 MHz, $CDCl_3$): δ_C 166.0, 165.9, 165.5, 148.9, 148.8, 144.4, 141.4, 138.8, 137.2, 135.9, 134.1, 133.0, 131.9, 130.7, 130.4, 130.3, 130.2, 129.9, 129.9, 129.9, 129.6, 129.6, 129.5, 128.8, 128.8, 128.7, 128.6, 128.5, 128.3, 128.1, 126.8, 126.7, 126.7, 107.1, 106.9, 75.6, 75.1, 75.1, 74.6, 68.3, 68.2, 47.4, 41.1, 41.1, 38.1, 38.0, 34.5, 34.4, 31.6, 31.6, 26.7, 26.7, 26.6, 26.6, 23.8, 23.8, 23.7, 22.2, 21.0, 20.9, 20.9, 16.7, 16.6

Note: Signals are doubled due to the presence of diastereomers.

IR (neat, ν cm^{-1}): 2655, 2928, 2968, 1711, 1447, 1269, 1177, 1185, 978

HRMS (ESI+): m/z calcd for $C_{34}H_{36}{}^{79}BrO_3$ $[M+H]^+$ 571.1842; found 571.1832

Isopropyl 2-(4-(4-bromo-2-(4-chlorophenyl)-5-phenyl-1-oxaspiro[2.3]hex-4-en-2-yl)phenoxy)-2-methylpropanoate, 3t

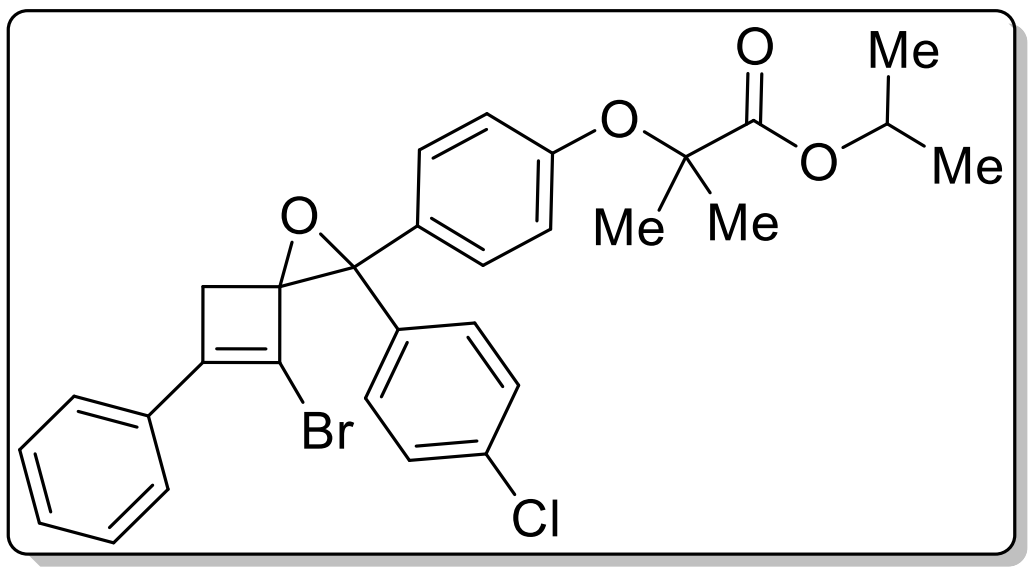

Prepared following **General Procedure 3** using *isopropyl 2-(4-((4-chlorophenyl)(hydroxy)(2-phenylcycloprop-1-en-1-yl)methyl)phenoxy)-2-methylpropanoate* **1w** (90 mg, 0.19 mmol). The crude material was obtained as 1:1 mixture of diastereomers. Purification by flash column chromatography (hexane to 10:1 hexane:$E_{t2}O$) yielded the title product **3t** as a colourless oil (55 mg, 0.10 mmol, ca 55:45 dr, 51%).

Data reported for mixture of diastereomers ($d_1 + d_2$)

^{1}H-NMR (400 MHz, $CDCl_3$): δ_H 7.69 – 7.64 (2x2H, m, $d_1 + d_2$), 7.51 – 7.44 (2H, m, d_1), 7.43 – 7.35 (10H, m, $d_1 + d_2$), 7.33 – 7.25 (2x2H, m, $d_1 + d_2$), 7.21 – 7.12 (2H, m, d_2), 6.92 – 6.85 (2H, m, d_1), 6.84 – 6.76 (2H, m, d_2), 5.12 – 2.03 (2H, m, $d_1 + d_2$), 3.12 (1H, d, J = 11.4 Hz, d_2) and 3.02 (1H, d, J = 11.4 Hz, d_2) (*AB syst., d_2*), 3.09 (1H, d, J = 11.6 Hz, d_1) and 2.95 (1H, d, J = 11.6 Hz, d_1) (*AB syst., d_1*), 1.61 (6H, d, J = 2.1 Hz, d_2), 1.59 – 1.56 (6H, m, d_1), 1.24 – 1.22 (6H, m, d_2), 1.22 – 1.19 (6H, m, d_1)

^{13}C{^{1}H}-NMR (101 MHz, $CDCl_3$): δ_C 173.8, 173.7, 155.7, 155.6, 148.7, 148.6, 138.4, 135.3, 134.1, 133.9, 132.3, 131.9, 130.2, 129.9, 129.7, 129.5, 128.7, 128.5, 128.4, 128.2, 127.7, 126.8, 118.7, 118.6, 107.2, 107.1, 79.4, 79.3, 74.6, 69.2, 69.1, 67.7, 38.1, 37.9, 25.6, 25.5, 25.5, 25.5, 21.8, 21.7, 21.7, 21.7

Note: Signals are doubled due to the presence of diastereomers.

IR (neat, ν cm^{-1}): 2981, 2934, 2875, 1726, 1508, 1383, 1278, 1095, 998

HRMS (ESI+): m/z calcd for $C_{30}H_{29}{}^{79}Br^{35}ClO_4$ $[M+H]^+$ 567.0932; found 567.0930

4-Fluoro-2,2,5-triphenyl-1-oxaspiro[2.3]hex-4-ene, 4a

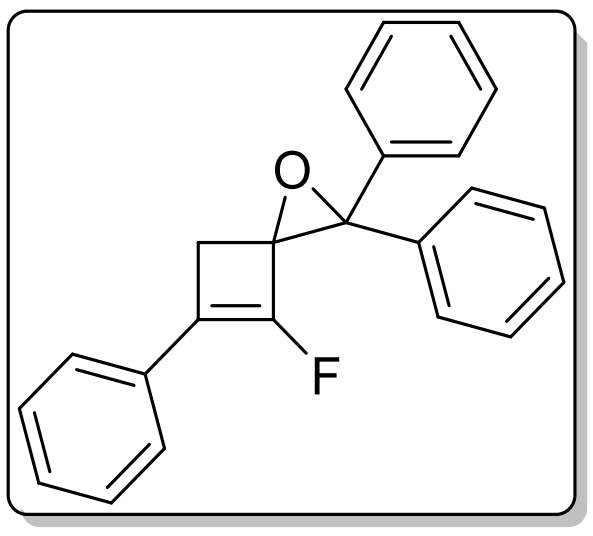

Prepared following **General Procedure 4** using *diphenyl(2-phenylcycloprop-1-en-1-yl)methanol* **1a** (60 mg, 0.20 mmol). Purification by flash column chromatography (hexane to 95:5 hexane:Et_2O, after neutralization of silica gel with 1% Et_3N) yielded the title product **4a** as a colourless oil (40 mg, 0.12 mmol, 61%).

^{1}H-NMR (400 MHz, $CDCl_3$): δ_H 7.56 – 7.52 (2H, m), 7.46 – 7.36 (8H, m), 7.36 – 7.29 (5H, m), 2.68 (1H, dd, *J* = 11.2, 10.2 Hz, 1H) and 2.56 (1H, dd, *J* = 14.8, 10.2 Hz) (*AB syst.*)

^{19}F-NMR (377 MHz, $CDCl_3$): δ_F -98.94 (dd, *J* = 14.8, 11.2 Hz)

^{13}C{^{1}H}-NMR (101 MHz, $CDCl_3$): δ_C 141.0 (d, *J* = 348.2 Hz), 139.2 (*overlapping with the doublet*), 137.5, 132.4, 130.6 (d, *J* = 5.5 Hz), 130.1, 128.8, 128.4, 128.4, 128.2, 128.0, 127.51 (d, *J* = 4.9 Hz), 127.0, 123.62 (d, *J* = 5.6 Hz), 74.5 (d, *J* = 20.5 Hz), 68.4 (d, *J* = 3.3 Hz), 30.1 (d, *J* = 22.1 Hz)

IR (neat, ν cm^{-1}): 3029, 2917, 2848, 1689, 1493, 1446, 1326, 1116, 907

HRMS (ESI+): *m/z* calcd for $C_{23}H_{18}FO$ $[M+H]^+$ 329.1336; found 329.1329

4-Fluoro-5-phenyl-2,2-di-*p*-tolyl-1-oxaspiro[2.3]hex-4-ene, 4b

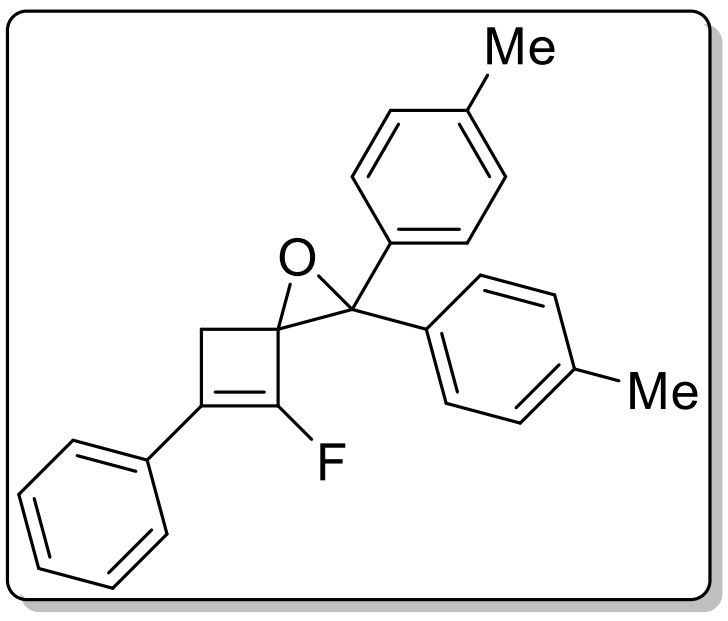

Prepared following **General Procedure 4** using *(2-phenylcycloprop-1-en-1-yl)di-p-tolylmethanol* **1a** (65 mg, 0.20 mmol). After work up, addition of hexane to the crude reaction mixture led to precipitation of product **4a**, which was isolated by filtration as a white solid (53 mg, 0.15 mmol, 75%).

^{1}H-NMR (400 MHz, $CDCl_3$): δ_H 7.51 – 7.27 (8H, m), 7.25 – 7.18 (3H, m), 7.15 (2H, app. d, *J* = 7.9 Hz), 2.65 (1H, app. t, *J* = 10.6 Hz) and 2.54 (1H, dd, *J* = 14.8, 10.2 Hz) (*AB syst.*), 2.39 (3H, s), 2.35 (3H, s)

^{19}F-NMR (377 MHz, $CDCl_3$): δ_F -98.70 (dd, *J* = 15.0, 11.2 Hz)

^{13}C{^{1}H}-NMR (101 MHz, $CDCl_3$): δ_C 141.3 (d, *J* = 349.4 Hz), 138.1, 137.6, 136.7, 134.9, 132.6, 130.8 (d, *J* = 5.5 Hz), 129.0, 128.7, 128.7 (d, *J* = 1.9 Hz), 128.1, 127.5 (d, *J* = 4.9 Hz), 126.9, 123.5 (d, *J* = 5.5 Hz), 74.4 (d, *J* = 20.6 Hz), 68.34 (d, *J* = 2.8 Hz), 30.1 (d, *J* = 22.2 Hz), 21.4, 21.2

IR (neat, v cm^{-1}): 3020, 2922, 2866, 1688, 1512, 1448, 1327, 1087, 923

HRMS (ESI+) *m/z* calcd for $C_{25}H_{22}FO$ $[M+H]^+$ 357.1649; found 357.1636

4-Fluoro-2,2-bis(4-fluorophenyl)-5-phenyl-1-oxaspiro[2.3]hex-4-ene, 4c

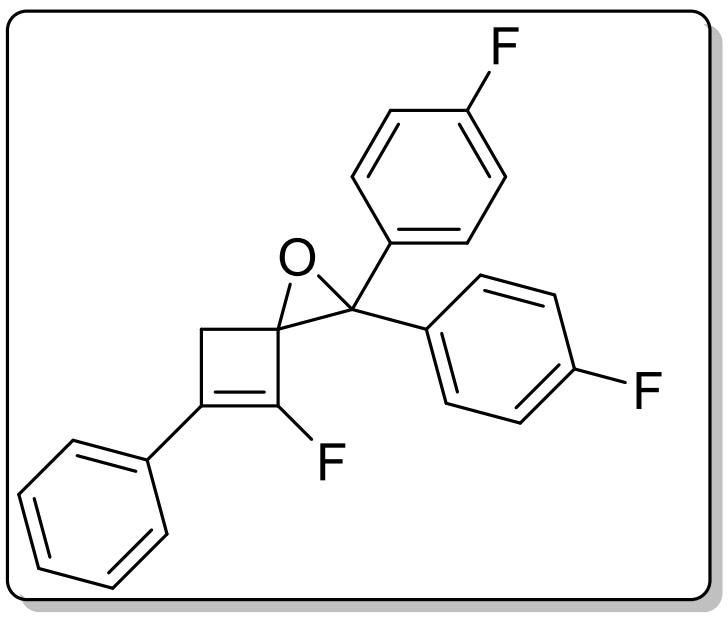

Prepared following **General Procedure 4** using *bis(4-fluorophenyl)(2-phenylcycloprop-1-en-1-yl)methanol* **1c** (67 mg, 0.20 mmol). Purification by flash column chromatography (hexane to 98:2 hexane:Et_2O, after neutralization of silica gel with 1% Et_3N) yielded the title product **4c** as a colourless oil (43 mg, 0.12 mmol, 60%).

^{1}H-NMR (400 MHz, $CDCl_3$): δ_H 7.50 – 7.45 (2H, m), 7.43 – 7.34 (4H, m), 7.34 – 7.27 (3H, m), 7.13 – 7.01 (4H, m), 2.66 (1H, dd, *J* = 11.2, 10.2 Hz) and 2.52 (1H, dd, *J* = 14.8, 10.2 Hz) (*AB syst.*)

^{19}F-NMR (377 MHz, $CDCl_3$): δ_F -99.37 (dd, *J* = 14.9, 11.2 Hz), -113.03 (m), -114.04 (m)

^{13}C{^{1}H}-NMR (101 MHz, $CDCl_3$): δ_C 162.8 (d, *J* = 247.7 Hz), 162.6 (d, *J* = 247.3 Hz), 140.6 (d, *J* = 348.6 Hz), 135.0 (d, *J* = 3.3 Hz), 133.4 (d, *J* = 3.5 Hz), 130.5 (d, *J* = 5.3 Hz), 130.0 (d, *J* = 8.3 Hz), 129.0 (d, *J* = 2.1 Hz), 129.0, 128.7 (d, *J* = 8.2 Hz), 127.5 (d, *J* = 4.8 Hz), 124.1 (d, *J* = 5.7 Hz), 115.5 (d, *J* = 21.6 Hz), 115.4 (d, *J* = 21.7 Hz), 74.5 (d, *J* = 20.8 Hz), 67.4 (d, *J* = 2.9 Hz), 29.9 (d, *J* = 21.8 Hz)

*Note: As stated in the respective spectrum in **Section 8**, the compound isolated after chromatography still contains 9% of the brominated analogue **3c**, formed during the reaction, which proved to be inseparable from the target product in all cases.*

IR (neat, ν cm^{-1}): 3031, 2932, 1690, 1604, 1508, 1456, 1222, 1155, 912

HRMS (ESI-) *m/z* calcd for $C_{23}H_{14}F_3O$ $[M-H]^-$ 363.1002; found 363.0992

4-Fluoro-2,5-diphenyl-2-(4-(*p*-tolylthio)phenyl)-1-oxaspiro[2.3]hex-4-ene, 4d

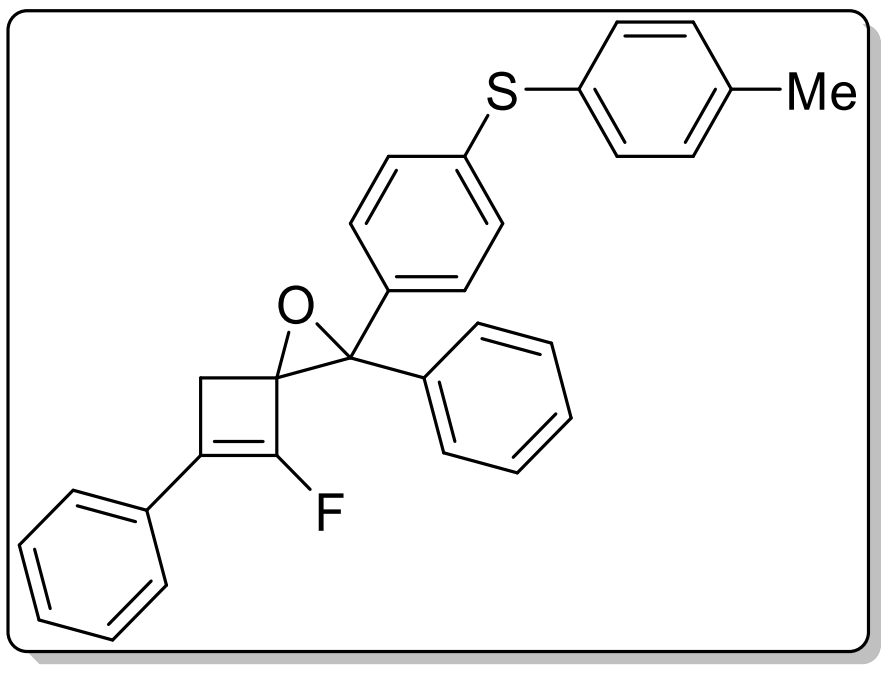

Prepared following **General Procedure 4** using *phenyl(2-phenylcycloprop-1-en-1-yl)(4-(p-tolylthio)phenyl)methanol* **1f** (84 mg, 0.20 mmol). The crude material was obtained as 1:1 mixture of diastereomers. Its purification by flash column chromatography led to partial conversion into the Meinwald rearrangement derivative **5d** in all conditions, due to the instability of the target compound on SiO_2. Flash column chromatography (hexane to 98:2 hexane:Et_2O, after neutralization of silica gel with 1% Et_3N) affords a mixture (42 mg) consisting of title compound **4d** (ca. 80%, ca. 1:1 dr) and ring-expanded product **5d** (ca. 20%), as highlighted in ^{19}F NMR spectrum in **Section 8.**

Data reported for mixture of diastereomers (d_1 + d_2)

^{1}H-NMR (400 MHz, $CDCl_3$): δ_H 7.53 – 7.50 (2H, m), 7.43 – 7.39 (8H, m, $d_1 + d_2$), 7.37 – 7.31 (12H, m, $d_1 + d_2$), 7.29 – 7.27 (2H, m), 7.22 – 7.17 (4H, m, $d_1 + d_2$), 2.70 – 2.61 (2H, m, $d_1 + d_2$), 2.59 – 2.49 (2H, m, $d_1 + d_2$), 2.38 (3H, s, d_1), 2.37 (3H, s, d_1)

^{19}F-NMR (377 MHz, $CDCl_3$): δ_F -98.74 (dd, J = 14.8, 11.3 Hz, d_1), -99.09 (dd, J = 14.8, 11.2 Hz, d_2)

^{13}C{^{1}H}-NMR (101 MHz, $CDCl_3$): δ_C 74.5 (d, J = 2.5 Hz), 74.3 (d, J = 3.0 Hz), 68.09, 30.1 (d, J = 21.8 Hz), 30.0 (d, J = 21.7 Hz), 21.3

Note: The aliphatic carbon signals have been assigned and highlighted in the corresponding ^{13}C spectrum. Signals relating to the aromatic region could not be assigned due to overlap with signals from **5d**. *Compound* **4d** *has been then fully converted to* **5d***; the ring-expanded product has been then fully characterized, and all signals have been properly assigned*.

IR (neat, ν cm^{-1}): 3023, 2923, 2859, 1644, 1603, 1495, 1361, 1015, 908

HRMS (ESI+): *m/z* calcd for $C_{30}H_{23}FOSNa$ 474.1379 $[M+Na]^+$*;* found 474.1370

1-Fluoro-2-phenyl-11-oxadispiro[3.0.5^5.1^4]undec-1-ene, 4e

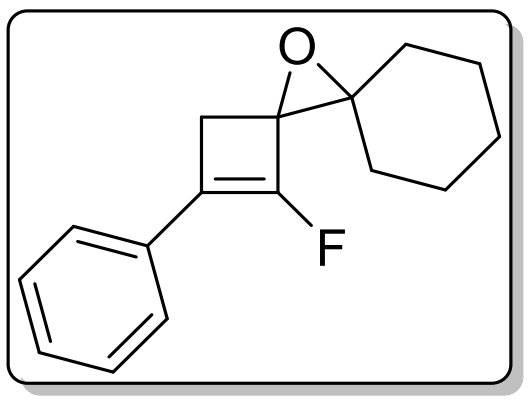

Prepared following **General Procedure 4** using *1-(2-phenylcycloprop-1-en-1-yl)cyclohexan-1-ol* **1i** (46 mg, 0.20 mmol). Purification by flash column chromatography (hexane to 98:2 hexane:Et_2O, after neutralization of silica gel with 1% Et_3N) yielded the title product **4e** as a colourless oil (33 mg, 0.13 mmol, 66%).

^{1}H-NMR (400 MHz, $CDCl_3$): δ_H 7.47 – 7.42 (2H, m), 7.41 – 7.35 (2H, m), 7.34 – 7.28 (1H, m), 2.69 (1H, dd, *J* = 15.0, 9.9 Hz) and 2.55 (1H, dd, *J* = 11.5, 9.9 Hz) (*AB syst.*), 1.86 – 1.77 (4H, m), 1.70 – 1.64 (2H, m), 1.61 – 1.50 (4H, m)

^{19}F-NMR (377 MHz, $CDCl_3$): δ_F -99.79 (dd, *J* = 15.0, 11.5 Hz)

^{13}C{^{1}H}-NMR (101 MHz, $CDCl_3$): δ_C 142.1 (d, *J* = 349.4 Hz), 130.9 (d, *J* = 5.5 Hz), 128.8 (d, *J* = 1.5 Hz), 128.5 (d, *J* = 2.0 Hz), 127.4 (d, *J* = 4.7 Hz), 121.9 (d, *J* = 5.6 Hz), 72.4 (d, *J* = 20.7 Hz), 66.4 (d, *J* = 3.1 Hz), 33.1, 32.1 (d, *J* = 1.6 Hz), 28.8 (d, *J* = 24.0 Hz), 25.5, 25.1, 24.8

IR (neat, ν cm^{-1}): 3028, 2931, 2855, 1688, 1492, 1447, 1324, 1031, 988

HRMS (ESI+) *m/z* calcd for $C_{16}H_{18}FO$ $[M+H]^+$ 245.1336; found 245.1332

tert-Butyl 1-fluoro-2-phenyl-11-oxa-8-azadispiro[3.0.5⁵.1⁴]undec-1-ene-8-carboxylate, 4f

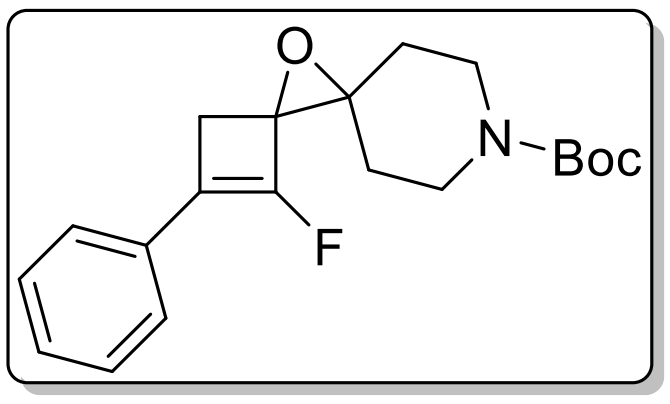

Prepared following **General Procedure 4** using *tert-butyl 4-hydroxy-4-(2-phenylcycloprop-1-en-1-yl)piperidine-1-carboxylate* **1j** (63 mg, 0.20 mmol). Purification by flash column chromatography (hexane to 90:10 hexane:Et_2O, after neutralization of silica gel with 1% Et_3N) yielded the title product **4f** as a colourless oil (41 mg, 0.12 mmol, 58%).

^{1}H-NMR (400 MHz, $CDCl_3$): δ_H 7.48 – 7.35 (4H, m), 7.36 – 7.28 (1H, m), 3.75 – 3.66 (2H, m), 3.55 – 3.48 (2H, m), 2.69 (1H, dd, *J* = 14.8, 9.9 Hz) and 2.59 (1H, dd, *J* = 11.5, 9.9 Hz) (*AB syst.*), 2.00 – 1.91 (1H, m), 1.90 – 1.72 (3H, m), 1.48 (9H, s)

^{19}F-NMR (377 MHz, $CDCl_3$): δ_F -100.48 (s)

^{13}C{^{1}H}-NMR (101 MHz, $CDCl_3$): δ_C 154.9, 141.3 (d, *J* = 348.2 Hz), 130.6 (d, *J* = 5.9 Hz), 128.8, 128.8 (d, *J* = 2.4 Hz), 127.5 (d, *J* = 4.7 Hz), 122.5 (d, J = 6.1 Hz), 80.0, 72.0 (d, *J* = 21.1 Hz), 64.3 (d, *J* = 3.3 Hz), 42.4, 41.5, 32.3, 31.6, 28.8 (d, *J* = 23.4 Hz), 28.6

IR (neat, ν cm^{-1}): 2973, 2926, 2844, 1691, 1420, 1365, 1233, 1167, 963

HRMS (ESI+) *m/z* calcd for $C_{20}H_{25}FNO_3$ $[M+H]^+$ 346.1813; found 346.1804

1-Fluoro-2,8-diphenyl-11-oxadispiro[3.0.5⁵.1⁴]undec-1-ene, 4g

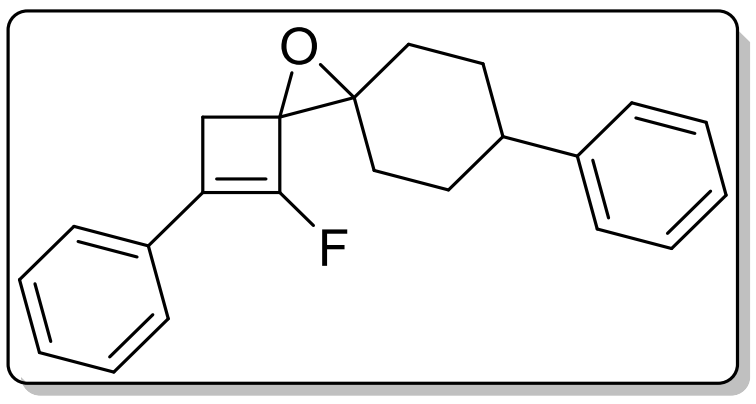

Prepared following **General Procedure 4** using *4-phenyl-1-(2-phenylcycloprop-1-en-1-yl)cyclohexan-1-ol* **1k** (58 mg, 0.20 mmol). The crude material was obtained as a single diastereomer. Purification by flash column chromatography (hexane to 98:2 hexane:Et_2O, after neutralization of silica gel with 1% Et_3N) yielded the title product **4g** as a colourless oil (26 mg, 0.083 mmol, 41%).

^{1}H-NMR (400 MHz, $CDCl_3$): δ_H 7.50 – 7.38 (4H, m), 7.37 – 7.25 (4H, m), 7.23 – 7.15 (2H, m), 2.76 (1H, dd, *J* = 14.9, 10.0 Hz), 2.69 – 2.58 (2x1H, m), 2.17 – 2.05 (4H, m), 1.89 (1H, app. d, *J* = 12.6 Hz), 1.75 – 1.58 (3H, m)

^{19}F-NMR (377 MHz, $CDCl_3$): δ_F -100.30 (dd, *J* = 14.8, 11.7 Hz)

^{13}C{^{1}H}-NMR (101 MHz, $CDCl_3$): δ_C 145.9, 141.9 (d, *J* = 349.1 Hz), 130.9 (d, *J* = 5.8 Hz), 128.8, 128.6 (d, *J* = 2.1 Hz), 128.6, 127.5 (d, *J* = 4.9 Hz), 126.9, 126.4, 122.1 (d, *J* = 5.8 Hz), 72.5 (d, *J* = 20.7 Hz), 66.2 (d, *J* = 3.0 Hz), 43.3, 33.4, 33.3, 33.3, 32.2, 29.0 (d, *J* = 23.5 Hz)

IR (neat, ν cm^{-1}): 3028, 2926, 2855, 1687, 1493, 1448, 1343, 1115, 744

HRMS (ESI+) *m/z* calcd for $C_{22}H_{22}FO$ $[M+H]^+$ 321.1649; found 321.1644

*Note: As stated in the respective spectrum in **Section 8**, the compound isolated after chromatography still contains 14% of the brominated analogue **3j**, formed during the reaction, which proved to be inseparable from the target product in all cases.*

4-Fluoro-2,2-diphenyl-5-(*p*-tolyl)-1-oxaspiro[2.3]hex-4-ene, 4h

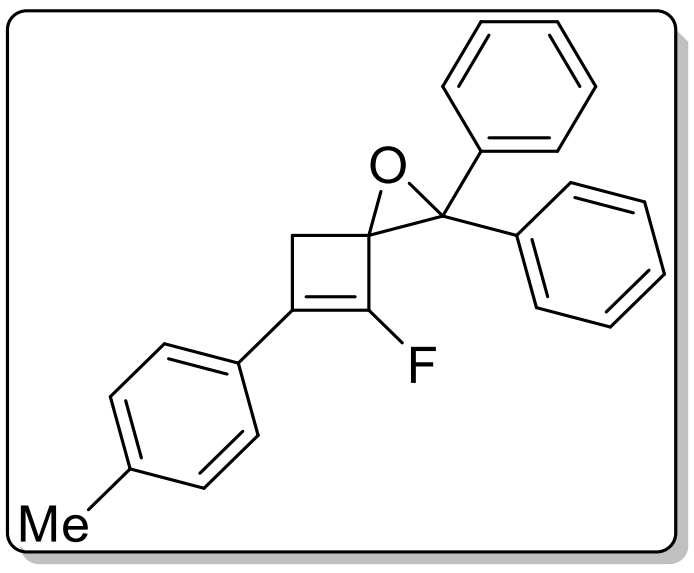

Prepared following **General Procedure 4** using *diphenyl(2-(p-tolyl)cycloprop-1-en-1-yl)methanol* **1l** (65 mg, 0.20 mmol). After work up, addition of hexane to the crude reaction mixture led to precipitation of product **4h**, which was isolated by filtration as a white solid (51 mg, 0.15 mmol, 72%).

^{1}H-NMR (400 MHz, $CDCl_3$): δ_H 7.56 – 7.45 (3H, m), 7.43 – 7.27 (9H, m), 7.20 – 7.12 (2H, m), 2.64 (1H, app. t, *J* = 10.7 Hz) and 2.52 (1H, dd, *J* = 14.8, 10.2 Hz) (*AB syst.*), 2.36 (3H, s).

^{19}F-NMR (377 MHz, $CDCl_3$): δ_F -100.34 (dd, *J* = 14.8, 11.4 Hz)

^{13}C{^{1}H}-NMR (101 MHz, $CDCl_3$): δ_C 143.4 (d, *J* = 295.6 Hz), 139.4, 138.9 (d, *J* = 2.4 Hz), 137.7, 130.3, 129.5, 128.5, 128.4, 128.3 (d, *J* = 1.8 Hz), 128.2, 127.9, 127.5 (d, *J* = 4.8 Hz), 126.8, 123.8 (d, *J* = 5.5 Hz), 74.5 (d, *J* = 20.7 Hz), 68.3 (d, *J* = 3.0 Hz), 30.1 (d, *J* = 22.1 Hz), 21.6

IR (neat, ν cm^{-1}): 3026, 2959, 2865, 1690, 1446, 1333, 1306, 1114, 958

HRMS (ESI+) *m/z* calcd for $C_{24}H_{20}FO$ $[M+H]^+$ 343.1493; found 343.1482

4-Fluoro-5-(4-fluorophenyl)-2,2-diphenyl-1-oxaspiro[2.3]hex-4-ene, 4i

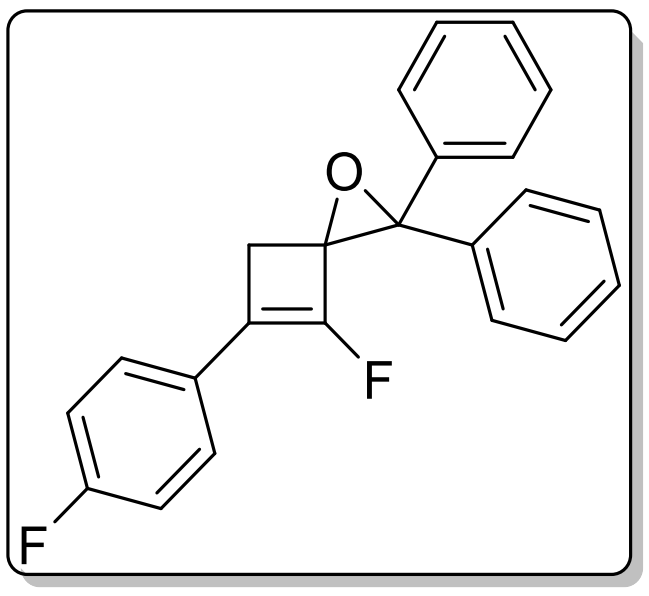

Prepared following **General Procedure 4** using *(2-(4-fluorophenyl)cycloprop-1-en-1-yl)diphenylmethanol* **1n** (65 mg, 0.20 mmol). Purification by flash column chromatography (hexane to 98:2 hexane:Et_2O, after neutralization of silica gel with 1% Et_3N) yielded the title product **4i** as a colourless oil (42 mg, 0.12 mmol, 59%).

^{1}H-NMR (400 MHz, $CDCl_3$): δ_H 7.56 – 7.51 (2H, m), 7.44 – 7.31 (10H, m), 7.11 – 7.02 (2H, m), 2.71 – 2.61 (1H, app. t., *J* = 10.4 Hz) and 2.53 (1H, dd, *J* = 14.8, 10.2 Hz) (*AB syst.*)

^{19}F-NMR (377 MHz, $CDCl_3$): δ_F -99.96 (td, *J* = 12.6, 11.9, 6.0 Hz), -111.12 (ttd, *J* = 8.6, 5.3, 2.9 Hz)

^{13}C{^{1}H}-NMR (101 MHz, $CDCl_3$): δ_C 162.7 (dd, *J* = 249.6, 2.7 Hz), 140.6 (dd, *J* = 348.3, 2.9 Hz), 139.3, 137.5, 130.2, 129.3 (dd, *J* = 8.4, 4.8 Hz), 128.4, 128.4, 128.4, 128.2, 128.0, 127.0, 122.7 (d, *J* = 5.3 Hz), 115.9 (d, *J* = 21.8 Hz), 74.4 (d, *J* = 20.7 Hz), 68.4 (d, *J* = 3.1 Hz), 30.2 (d, *J* = 22.0 Hz)

IR (neat, ν cm^{-1}): 3032, 2933, 1692, 1604, 1508, 1446, 1233, 1157, 1084, 835

HRMS (ESI+) *m/z* calcd for $C_{23}H_{17}F_2O$ $[M+H]^+$ 347.1242; found 347.1246

4-Fluoro-5-isopentyl-2,2-diphenyl-1-oxaspiro[2.3]hex-4-ene, 4j

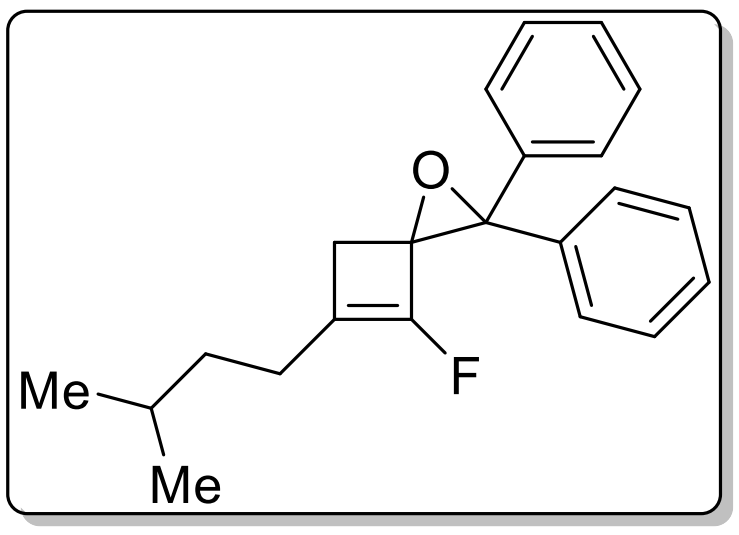

Prepared following **General Procedure 4** using *(2-isopentylcycloprop-1-en-1-yl)diphenylmethanol* **1o** (57 mg, 0.19 mmol). Purification by flash column chromatography (hexane to 98:2 hexane:Et_2O, after neutralization of silica gel with 1% Et_3N) yielded the title product **4j** as a colourless oil (29 mg, 0.091 mmol, 47%).

^{1}H-NMR (400 MHz, $CDCl_3$): δ_H 7.52 – 7.45 (2H, m), 7.41 – 7.26 (6H, m), 7.24 – 7.19 (1H, m), 2.30 (1H, t, *J* = 10.9 Hz), 2.23 – 2.10 (1H + 2H, m), 1.63 – 1.56 (1H, m – *overlapping with residual water peak*), 1.40 (2H, q, *J* = 7.7 Hz), 0.90 (3H, d, *J* = 2.1 Hz), 0.89 (3H, d, *J* = 2.1 Hz)

^{19}F-NMR (377 MHz, $CDCl_3$): δ_F -107.31 (dd, *J* = 14.9, 11.5 Hz)

^{13}C{^{1}H}-NMR (101 MHz, $CDCl_3$): δ_C 140.8 (d, *J* = 340.0 Hz), 139.7, 137.9, 132.6, 130.2, 128.3, 128.2, 127.8, 127.0, 126.7 (d, *J* = 4.4 Hz), 74.7 (d, *J* = 21.8 Hz), 67.7 (d, *J* = 2.9 Hz), 35.6, 31.8 (d, *J* = 23.7 Hz), 27.9, 24.4 (d, *J* = 3.8 Hz), 22.5, 22.4

IR (neat, v cm^{-1}): 2955, 2923, 2869, 1661, 1446, 1277, 1146, 917

HRMS (ESI+): *m/z* calcd for $C_{22}H_{24}FO$ $[M+H]^+$ 323.1806; found 323.1799

4-Fluoro-2,2-diphenyl-5-(3-phenylpropyl)-1-oxaspiro[2.3]hex-4-ene, 4k

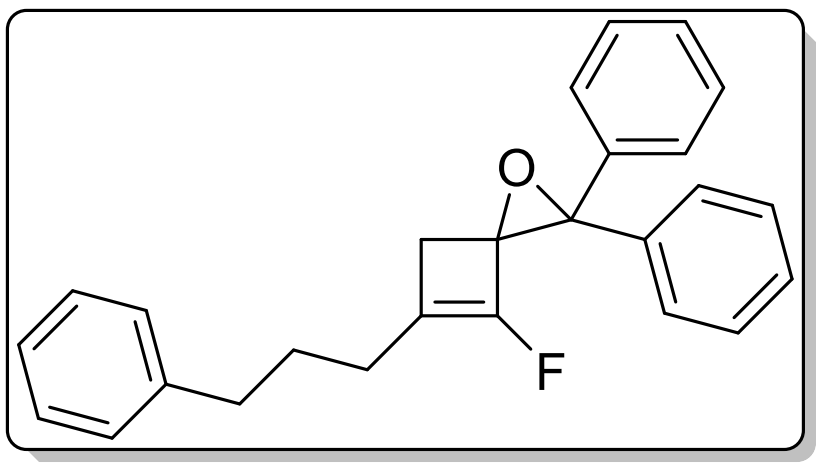

Prepared following **General Procedure 4** using *diphenyl(2-(3-phenylpropyl)cycloprop-1-en-1-yl)methanol* **1q** (70 mg, 0.20 mmol). Purification by flash column chromatography (hexane to 98:2 hexane:Et_2O, after neutralization of silica gel with 1% Et_3N) yielded the title product **4k** as a colourless oil (46 mg, 0.12 mmol, 60%).

^{1}H-NMR (400 MHz, $CDCl_3$): δ_H 7.48 (2H, app. d, *J* = 7.2 Hz), 7.42 – 7.34 (4H, m), 7.34 – 7.26 (6H, m), 7.21 – 7.15 (3H, m), 2.66 (2H, t, *J* = 7.7 Hz), 2.32 (1H, t, *J* = 10.9 Hz), 2.26 – 2.09 (3H, m), 1.86 (2H, p, *J* = 7.5 Hz)

^{19}F-NMR (377 MHz, $CDCl_3$): δ_F -106.41 (dd, *J* = 14.9, 11.5 Hz)

^{13}C{^{1}H}-NMR (101 MHz, $CDCl_3$): δ_C 141.8, 141.2 (d, *J* = 339.9 Hz), 139.6 (*overlapping with the doublet at 141.2*), 137.8, 128.6, 128.5, 128.3, 128.2, 128.2, 127.8, 127.0, 126.1, 126.1 (d, *J* = 7.4 Hz) 74.6 (d, *J* = 22.0 Hz), 67.8 (d, *J* = 2.8 Hz), 35.6, 31.7 (d, *J* = 23.5 Hz), 28.4 (d, *J* = 1.7 Hz), 26.0 (d, *J* = 3.7 Hz)

IR (neat, v cm^{-1}): 3026, 2932, 2857, 1722, 1494, 1446, 1290, 1082, 1031, 907

HRMS (ESI+) *m/z* calcd for $C_{26}H_{24}FO$ $[M+H]^+$ 371.1806; found 371.1800

*Note: Due to overlapping of the signals from the vinyl carbons of the cyclobutene ring with signals from the aromatic region, a copy of the HMBC spectrum has been included in **Section 8** to support their chemical shift.*

tert-Butyl 2''-fluoro-3''-phenyl-8-azadispiro[bicyclo[3.2.1]octane-3,2'-oxirane-3',1''-cyclobutan]-2''-ene-8-carboxylate, 4l

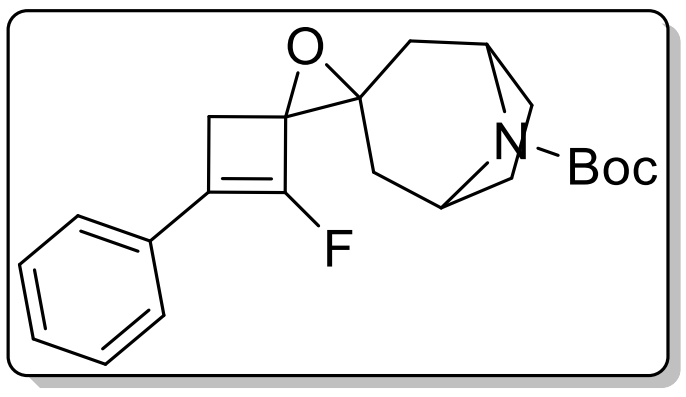

Prepared following **General Procedure 4** using *tert-butyl 3-hydroxy-3-(2-phenylcycloprop-1-en-1-yl)-8-azabicyclo[3.2.1]octane-8-carboxylate* **1s** (67 mg, 0.20 mmol). After work up, addition of hexane to the crude reaction mixture led to precipitation of product **4l**, which was isolated by filtration as a yellow solid (48 mg, 0.13 mmol, 63%).

^{1}H-NMR (400 MHz, $CDCl_3$): δ_H 7.46 – 7.39 (2H, m), 7.39 – 7.34 (2H, m), 7.33 – 7.27 (1H, m), 4.34 (2H, d, J = 28.0 Hz), 2.60 (1H, dd, J = 14.9, 9.9 Hz), 2.51 (1H, dd, J = 11.4, 9.9 Hz), 2.41 – 2.15 (2H, m), 2.18 – 2.00 (4H, m), 1.65 – 1.58 (1H, m), 1.47 (9H, s), 1.36 (1H, d, J = 12.6 Hz)

^{19}F-NMR (377 MHz, $CDCl_3$): δ_F -99.87 (d, J = 99.7 Hz)

^{13}C{^{1}H}-NMR (101 MHz, $CDCl_3$): δ_C 153.4, 141.4 (d, J = 349.0 Hz), 130.7 (d, J = 5.5 Hz), 128.7 (d, J = 1.4 Hz), 128.6 (d, J = 1.9 Hz), 127.4 (d, J = 4.8 Hz), 122.4, 79.6, 67.9 (d, J = 21.2 Hz), 62.3 (d, J = 3.2 Hz), 53.6, 53.0, 37.9, 37.5, 28.6, 28.3 (d, J = 23.8 Hz), 27.6, 27.6

IR (neat, v cm^{-1}): 2975, 2911, 1691, 1435, 1321, 1174, 1100, 1024, 762

HRMS (ESI+) *m/z* calcd for $C_{22}H_{27}FNO_3$ $[M+H]^+$ 372.1969; found 372.1964

*Note: Due to the low intensity of some signals resulting from long relaxation times, a copy of the HSQC spectrum has been included in **Section 8** to support their chemical shift.*

1,7,7-Trimethylbicyclo[2.2.1]heptan-2-yl 4-(6-fluoro-2,5-diphenyl-1-oxaspiro[2.3]hex-4-en-2-yl)benzoate, 4m

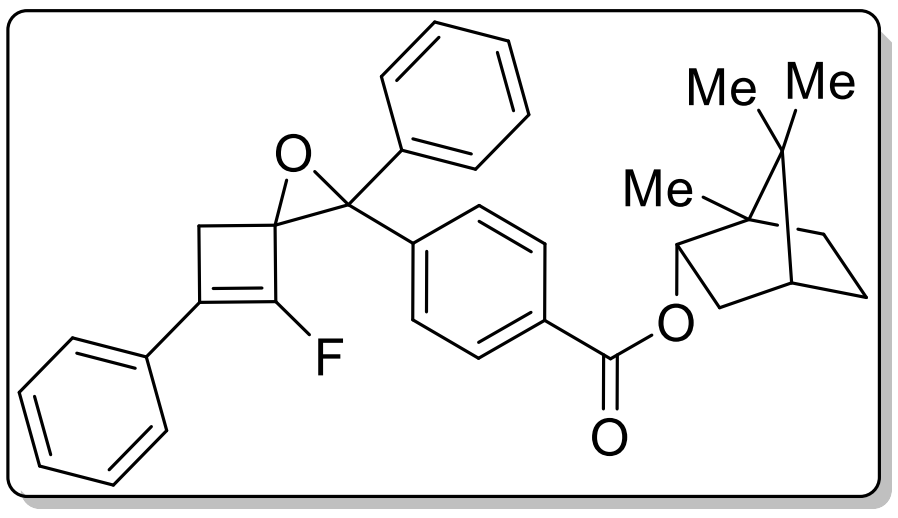

Prepared following **General Procedure 4** using *1,7,7-trimethylbicyclo[2.2.1]heptan-2-yl 4-(hydroxy(phenyl)(2-phenylcycloprop-1-en-1-yl)methyl)benzoate* **1u** (98 mg, 0.20 mmol). The crude material was obtained as 1:1 mixture of diastereomers. Purification by flash column chromatography (hexane to 90:10 hexane:Et_2O, after neutralization of silica gel with 1% Et_3N) yielded the title product **4m** as a yellow oil (44 mg, 0.086 mmol, ca. 1:1 dr, 42%).

Data reported for mixture of diastereomers (d_1 + d_2)

^{1}H-NMR (400 MHz, $CDCl_3$): δ_H 8.10 (2H, d, *J* = 8.3 Hz, d_1), 8.04 (2H, d, *J* = 8.3 Hz, d_2), 7.70 – 7.60 (3H, m, d_1), 7.58 – 7.48 (3H, m, d_2), 7.46 – 7.31 (2X9H, m, $d_1 + d_2$), 5.17 – 5.08 (2x1H, m, $d_1 + d_2$), 2.74 – 2.64 (2x1H, m, $d_1 + d_2$), 2.63 – 2.53 (2x1H, m, $d_1 + d_2$), 2.50 – 2.43 (2x1H, m, $d_1 + d_2$), 1.85 – 1.77 (2x1H, m, $d_1 + d_2$), 1.76 – 1.72 (2x1H, m, $d_1 + d_2$), 2.18 – 2.06 (2x1H, m, $d_1 + d_2$), 1.46 – 1.38 (2x1H, m, $d_1 + d_2$), 1.33 – 1.26 (2x1H, m, $d_1 + d_2$), 1.16 – 1.08 (2x1H, m, $d_1 + d_2$), 0.98 (3H, s, d_1), 0.97 (3H, s, d_2), 0.94 – 0.88 (2x6H, m, $d_1 + d_2$)

^{19}F-NMR (377 MHz, $CDCl_3$): δ_F -99.09 (dd, *J* = 14.3, 11.5 Hz), -99.49 (dd, *J* = 14.4, 11.6 Hz)

^{13}C{^{1}H}-NMR (101 MHz, $CDCl_3$): δ_C 166.6, 166.6, 144.2, 142.4, 140.7 (d, *J* = 348.5 Hz), 140.6 (d, *J* = 350.6 Hz), 136.9, 124.2 (d, *J* = 5.4 Hz), 130.9, 130.5, 129.6 (d, *J* = 2.9 Hz), 128.9, 128.8, 128.7, 128.5, 128.3, 128.1, 127.5 (d, *J* = 4.9 Hz), 127.0, 126.8, 126.7, 124.0 (d, *J* = 5.7 Hz), 80.8, 80.8, 74.5 (d, *J* = 20.5 Hz), 74.4 (d, *J* = 20.5 Hz), 68.2 (d, *J* = 3.2 Hz), 68.0 (d, *J* = 3.5 Hz), 49.3, 48.0, 45.1, 37.1, 30.1 (d, *J* = 21.6 Hz), 29.9 (d, *J* = 21.7 Hz), 28.2, 27.5, 19.9, 19.1, 13.8, 13.7

Note: Some signals are doubled due to the presence of diastereomers.

IR (neat, ν cm^{-1}): 3026, 2953, 2877, 1713, 1355, 1271, 1112, 966

HRMS (ESI+) *m/z* calcd for $C_{34}H_{34}FO_3$ $[M+H]^+$ 509.2486; found 509.2467

2-Isopropyl-5-methylcyclohexyl 4-(4-fluoro-2,5-diphenyl-1-oxaspiro[2.3]hex-4-en-2-yl)benzoate, 4n

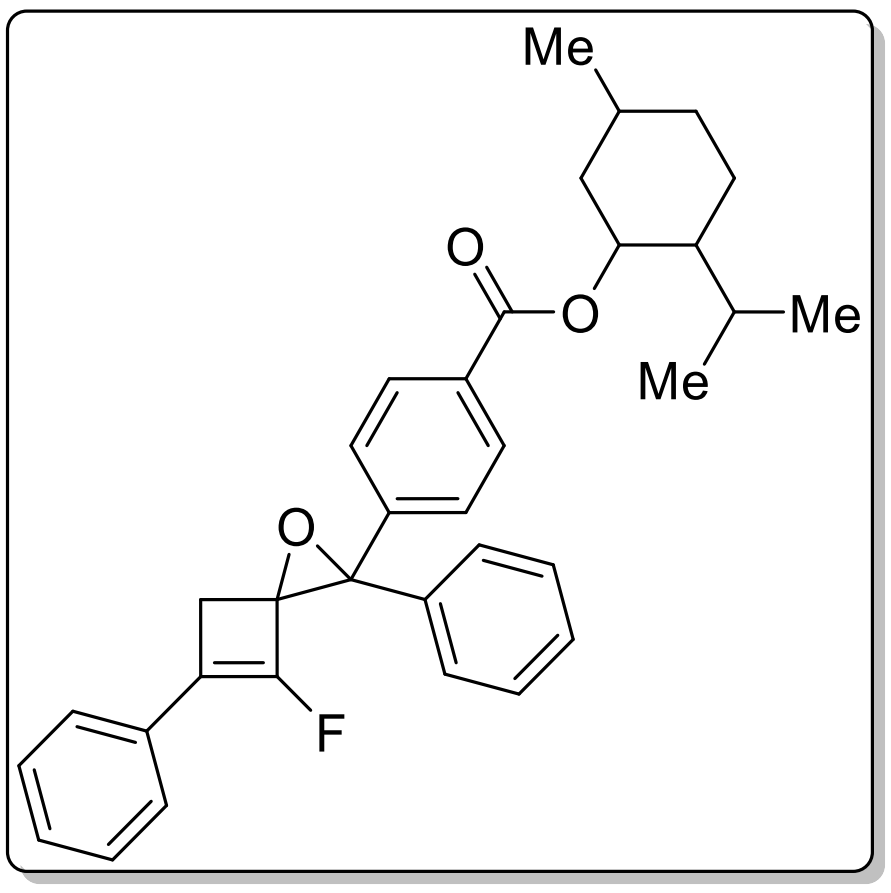

Prepared following **General Procedure 4** using *2-isopropyl-5-methylcyclohexyl 4-(hydroxy(phenyl)(2-phenylcycloprop-1-en-1-yl)methyl)benzoate* **1v** (100 mg, 0.20 mmol). The crude material was obtained as 1:1:1:1 mixture of diastereomers. Purification by flash column chromatography (hexane to 90:10 hexane:Et_2O, after neutralization of silica gel with 1% Et_3N) yielded the title product **4n** as a colourless oil (68 mg, 0.13 mmol, ca. 1:1:1:1 dr, 64%).

Data reported for mixture of diastereomers ($d_1 + d_2 + d_3 + d_4$)

^{1}H-NMR (400 MHz, $CDCl_3$): δ_H 8.10 (2x2H, d, J = 7.9 Hz, $d_1 + d_2$), 8.04 (2x2H, d, J = 8.0 Hz, 1H, $d_3 + d_4$), 7.70 – 7.60 (2x3H, m, $d_1 + d_2$), 7.59 – 7.50 (2x3H, m, $d_3 + d_4$), 7.48 – 7.30 (36H, m, $d_1 + d_2 + d_3 + d_4$), 5.00 – 4.89 (4x1H, m, $d_1 + d_2 + d_3 + d_4$), 2.74 – 2.61 (4x1H, m, $d_1 + d_2 + d_3 + d_4$), 2.61 – 2.44 (4x1H, m, $d_1 + d_2 + d_3 + d_4$), 2.18 – 2.10 (4x1H, m, $d_1 + d_2 + d_3 + d_4$), 2.04 – 1.91 (4x1H, m, $d_1 + d_2 + d_3 + d_4$), 1.79 – 1.70 (4x2H, m, $d_1 + d_2 + d_3 + d_4$), 1.62 – 1.52 (4x2H, m, $d_1 + d_2 + d_3 + d_4$), 1.22 – 1.07 (4x2H, m, $d_1 + d_2 + d_3 + d_4$), 0.97 – 0.93 (2x6H, m, $d_1 + d_2$), 0.93 – 0.90 (2x6H, m, $d_1 + d_2 + d_3 + d_4$), 0.81 (4x3H, app. t, J = 6.3 Hz, $d_1 + d_2 + d_3 + d_4$)

^{19}F-NMR (377 MHz, $CDCl_3$): δ_F -99.05 (t, J = 13.0 Hz, $d_1 + d_2$), -99.40 (2F, q, J = 13.4, 13.0 Hz, $d_3 + d_4$)

^{13}C{^{1}H}-NMR (101 MHz, $CDCl_3$): δ_C 165.9, 165.8, 144.1, 142.3, 140.7 (d, J = 348.7 Hz), 140.64 (d, J = 348.6 Hz), 138.5, 136.9, 130.9, 130.5 (d, J = 5.3 Hz), 130.5, 130.5, 129.7, 129.6, 128.9, 128.8, 128.7, 128.5, 128.3, 128.2, 128.1, 127.5 (d, J = 4.8 Hz), 126.9, 126.9, 126.8, 126.7, 124.1 (d, J = 5.8 Hz), 124.0 (d, J = 5.3 Hz), 75.1, 74.5 (d, J = 21.1 Hz), 74.4 (d, J = 20.9 Hz), 68.2 (d, J = 3.0 Hz), 68.0 (d, J = 3.0 Hz), 77.4, 41.1, 34.5, 31.6,

30.1 (d, J = 21.7 Hz), 29.90 (d, J = 21.7 Hz), 26.7, 26.6, 26.6, 23.8, 23.7, 22.2, 20.9, 20.9, 20.9, 16.7, 16.6, 16.6

Note – A: Some signals are doubled due to the presence of diastereomers.

*Note – B: A copy of $^{19}F\{^1H\}$ NMR spectrum is given in **Section 8**, highlighting the presence of four diastereomers in the sample.*

IR (neat, ν cm^{-1}): 3026, 2954, 2927, 2877, 1712, 1447, 1272, 1177, 1092, 913

HRMS (ESI+) *m/z* calcd for $C_{34}H_{36}FO_3$ $[M+H]^+$ 511.2628; found 511.2643

Isopropyl 2-(4-(2-(4-chlorophenyl)-4-fluoro-5-phenyl-1-oxaspiro[2.3]hex-4-en-2-yl)phenoxy)-2-methylpropanoate, 4o

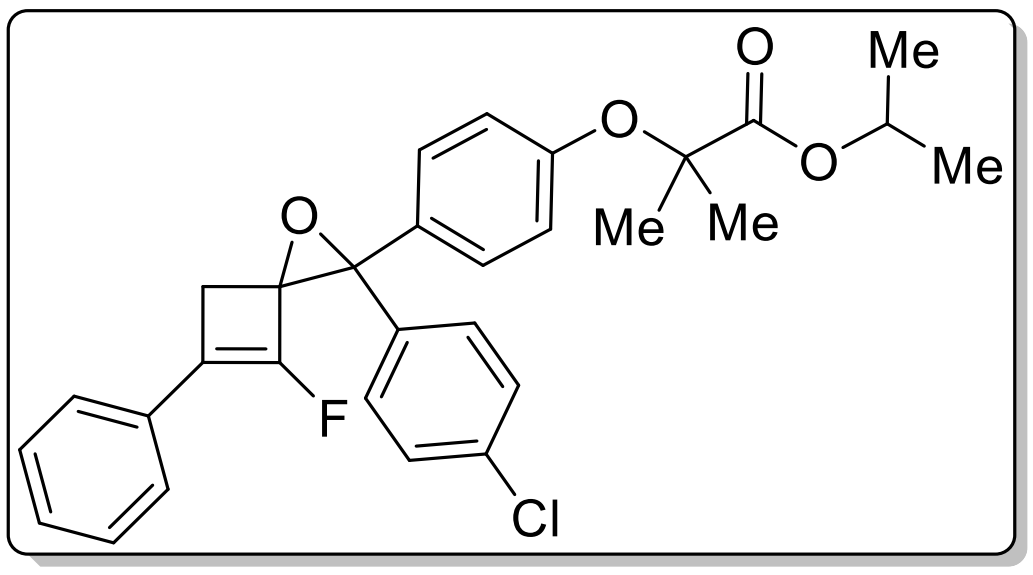

Prepared following **General Procedure 4** using *isopropyl 2-(4-((4-chlorophenyl)(hydroxy)(2-phenylcycloprop-1-en-1-yl)methyl)phenoxy)-2-methylpropanoate* **1w** (95 mg, 0.20 mmol). The crude material was obtained as 1:1 mixture of diastereomers. Purification by flash column chromatography (hexane to 95:5 hexane:Et_2O, after neutralization of silica gel with 1% Et_3N) yielded the title product **4o** as a white solid (41 mg, 0.080 mmol, ca. 60:40 dr, 41%).

Data reported for mixture of diastereomers (d_1 + d_2)

^{1}H-NMR (400 MHz, $CDCl_3$): δ_H 7.47 – 7.27 (2x9H, m, d_1 + d_2), 7.25 – 7.22 (m, 2H, d_1), 7.19 – 7.12 (2H, m, d_2), 6.89 – 6.78 (2x2H, m, d_1 + d_2), 5.08 (2x1H, hept, *J* = 6.5 Hz, d_1 + d_2), 2.71 – 2.55 (2x1H, m, d_1 + d_2), 2.58 – 2.41 (2x1H, m, d_1 + d_2), 1.62 (6H, d, *J* = 1.8 Hz, d_1), 1.59 (6H, s, d_2), 1.22 – 1.18 (2x6H, m, d_1 + d_2)

^{19}F-NMR (377 MHz, $CDCl_3$): δ_F -98.74 (dd, *J* = 14.6, 11.4 Hz, d_1), -99.37 (dd, *J* = 14.7, 11.2 Hz, d_2)

^{13}C{^{1}H}-NMR (101 MHz, $CDCl_3$): δ_C 173.7, 173.6, 154.9, 143.3, 141.5 (*overlapping with the doublet at 139.9*), 139.9 (d, *J* = 327.8 Hz), 135.5, 135.3, 133.9, 133.7, 131.7, 131.3, 132.1, 129.3, 129.3, 129.2, 128.9, 128.8, 128.6, 128.4, 128.4 (d, *J* = 2.3 Hz), 127.9, 127.5 (d, *J* = 4.9 Hz). 123.9 (d, *J* = 5.0 Hz), 119.0, 118.4, 79.3, 79.3, 74.5 (d, *J* = 20.6 Hz), 74.4 (d, *J* = 20.9 Hz), 69.2, 69.1, 41.6, 41.6, 29.9 (d, *J* = 22.1 Hz), 25.6, 25.5, 21.7, 21.7

Note – A: Some signals are doubled due to the presence of diastereomers.

Note- B: To support the chemical shift of some signals in ^{13}C{^{1}H}-NMR spectrum, due to long relaxation times, HSQC and HMBC spectra have been included in ***Section 8****.*

IR (neat, ν cm^{-1}): 2981, 2935, 1727, 1508, 1286, 1242, 1177, 1101, 920

HRMS (ESI+): *m/z* calcd for $C_{30}H_{29}{}^{35}ClFO_4$ $[M+H]^+$ 507.1733; found 507.1721

3-Chloro-2,2,4-triphenylcyclopent-3-en-1-one, 5a

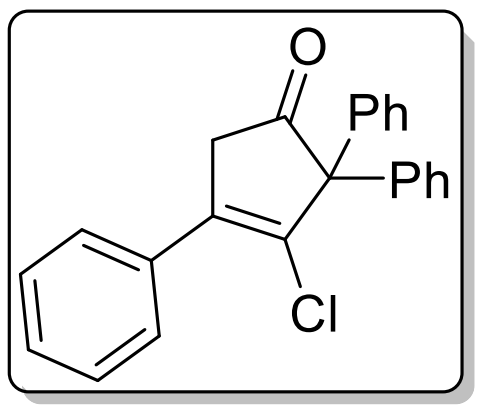

Prepared following **General Procedure 5** using *4-chloro-2,2,5-triphenyl-1-oxaspiro[2.3]hex-4-ene* **2a** (70 mg, 0.20 mmol). Purification by flash column chromatography (hexane to 90:10 hexane:Et_2O) yielded the title product **5a** as a white solid (26 mg, 0.076 mmol, 38%).

^{1}H-NMR (400 MHz, $CDCl_3$): δ_H 8.05 – 7.99 (2H, m), 7.56 – 7.49 (3H, m), 7.37 – 7.26 (10H, m), 3.86 (2H, s)

^{13}C{^{1}H}-NMR (101 MHz, $CDCl_3$): δ_C 200.5, 160.8, 142.8, 132.9, 131.6, 129.0, 128.8, 128.5, 128.3, 128.0, 127.3, 59.0, 47.2

IR (neat, v cm^{-1}): 3025, 2958, 2923, 1716, 1596, 1494, 1445, 1216, 967

HRMS (ESI+): *m/z* calcd for $C_{23}H_{18}{}^{35}ClO$ $[M+H]^+$ 345.1041; found 345.1035

3-Bromo-2,2,4-triphenylcyclopent-3-en-1-one, 5b

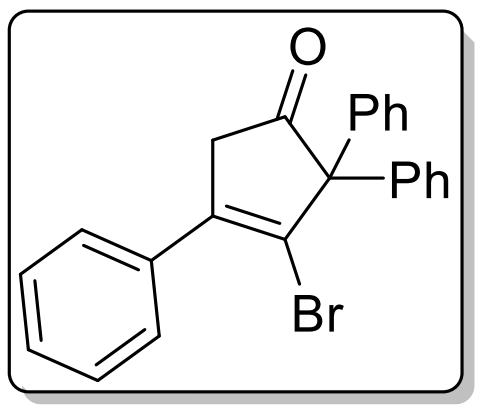

Prepared following **General Procedure 5** using *4-bromo-2,2,5-triphenyl-1-oxaspiro[2.3]hex-4-ene* **3a** (77 mg, 0.20 mmol). Purification by flash column chromatography (hexane to 90:10 hexane:Et_2O) yielded the title product **5b** as a white solid (25 mg, 0.066 mmol, 33%).

^{1}H-NMR (400 MHz, $CDCl_3$): δ_H 8.05 – 7.95 (2H, m), 7.53 (3H, dd, *J* = 5.2, 2.0 Hz), 7.37 – 7.26 (10H, m), 3.86 (2H, s)

^{13}C{^{1}H}-NMR (101 MHz, $CDCl_3$): δ_C 200.9, 164.9, 142.9, 133.6, 131.6, 128.9, 128.8, 128.2, 128.0, 127.3, 119.8, 59.2, 49.2

IR (neat, ν cm^{-1}): 3023, 2925, 2923, 1713, 1591, 1494, 1444, 1180, 870

HRMS (ESI+): *m/z* calcd for $C_{23}H_{18}{}^{79}BrO$ $[M+H]^+$ 389.0536; found 389.0526

3-Fluoro-2,2,4-triphenylcyclopent-3-en-1-one, 5c

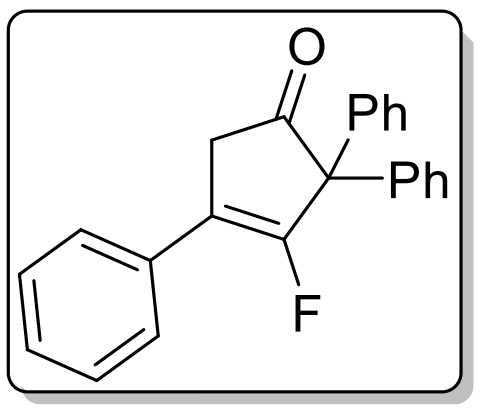

Prepared following **General Procedure 5** using *4-fluoro-2,2,5-triphenyl-1-oxaspiro[2.3]hex-4-ene* **4a** (62 mg, 0.19 mmol). Purification by flash column chromatography (hexane to 98:2 hexane:Et_2O) yielded the title product **5c** as a white solid (20 mg, 0.062 mmol, 35%).

^{1}H-NMR (400 MHz, $CDCl_3$): δ_H 7.90 – 7.83 (2H, m), 7.54 – 7.49 (3H, m), 7.37 – 7.25 (10H, m), 3.72 (2H, d, *J* = 6.3 Hz)

^{19}F-NMR (377 MHz, $CDCl_3$): δ_F -136.54 (t, *J* = 6.3 Hz)

^{13}C{^{1}H}-NMR (101 MHz, $CDCl_3$): δ_C 197.7 (d, *J* = 17.6 Hz), 152.1 (d, *J* = 284.5 Hz), 143.3, 142.8, 131.6 (d, *J* = 1.9 Hz), 131.0 (d, *J* = 5.2 Hz), 129.2, 128.8, 128.3 (d, *J* = 7.2 Hz), 128.0, 127.3, 57.4 (d, *J* = 4.5 Hz), 41.7 (d, *J* = 3.5 Hz)

IR (neat, ν cm^{-1}): 3027, 2926, 1710, 1643, 1446, 1360, 1052, 763

HRMS (ESI+): *m/z* calcd for $C_{23}H_{18}FO$ $[M+H]^+$ 329.1336; found 329.1326

3-Fluoro-2,4-diphenyl-2-(4-(*p*-tolylthio)phenyl)cyclopent-3-en-1-one, 5d

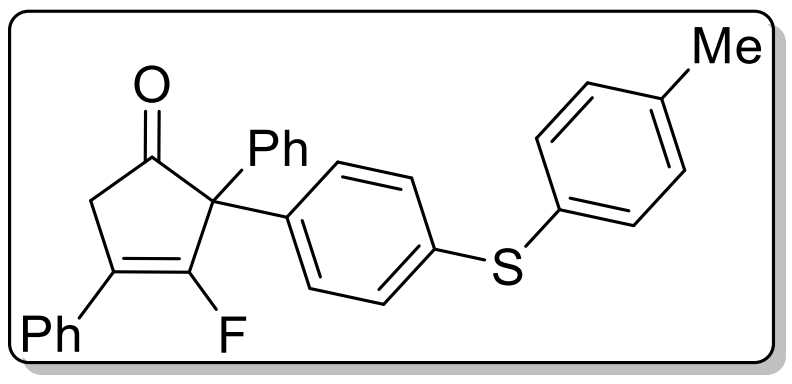

To a solution of *4-fluoro-2,5-diphenyl-2-(4-(p-tolylthio)phenyl)-1-oxaspiro[2.3]hex-4-ene* **4d** (30 mg, 0.066 mmol) in DCM (0.3 mL) was added SiO_2 in one portion; the resulting mixture was allowed to stir for 1 hour at room temperature. It was filtered through a short pad of celite and concentrated under reduced pressure to afford the title product **5d** as a yellow oil, which was characterized without any further purification (26 mg, 0.059 mmol, 88%).

^{1}H-NMR (400 MHz, $CDCl_3$): δ_H 7.88 – 7.80 (2H, m), 7.53 – 7.48 (2H, m), 7.38 – 7.26 (8H, m), 7.20 – 7.10 (6H, m), 3.67 (2H, d, *J* = 6.3 Hz), 2.35 (3H, s)

^{19}F-NMR (377 MHz, $CDCl_3$): δ_F -136.43 (t, *J* = 6.3 Hz)

^{13}C{^{1}H}-NMR (101 MHz, $CDCl_3$): δ_C 197.5 (d, *J* = 17.6 Hz), 152.0 (d, *J* = 284.6 Hz), 143.3, 142.5, 140.7, 134.6, 138.1, 136.8, 133.0, 131.6 (d, *J* = 1.9 Hz), 130.3, 130.3, 129.4, 129.2, 128.8, 128.6, 128.3 (d, *J* = 7.1 Hz), 127.9, 127.4, 57.0 (d, *J* = 4.7 Hz), 41.5 (d, *J* = 3.4 Hz), 21.3

IR (neat, v cm^{-1}): 3015, 2947, 2923, 1715, 1627, 1523, 1444, 1105, 913

HRMS (ESI+): *m/z* calcd for $C_{30}H_{23}FOSNa$ 473.1346 $[M+Na]^+$*;* found 473.1343

(2-Chloro-1,3-diphenylcyclobut-2-en-1-yl)(phenyl)methanone, 6a

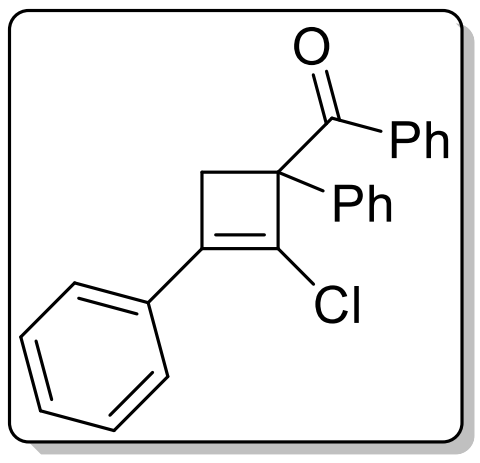

Prepared following **General Procedure 5** using *4-chloro-2,2,5-triphenyl-1-oxaspiro[2.3]hex-4-ene* **2a** (70 mg, 0.20 mmol). Purification by flash column chromatography (hexane to 90:10 hexane:Et_2O) yielded the title product **6a** as a yellowish oil (30 mg, 0.092 mmol, 44%).

^{1}H-NMR (400 MHz, $CDCl_3$): δ_H 7.87 – 7.82 (2H, m), 7.64 (2H, dd, *J* = 8.2, 1.5 Hz), 7.51 – 7.27 (11H, m), 3.76 (1H, d, *J* = 11.5 Hz, 1H) and 3.14 (1H, d, *J* = 11.5 Hz) (*AB syst.*)

^{13}C{^{1}H}-NMR (101 MHz, $CDCl_3$): δ_C 197.7, 140.8, 139.2, 135.6, 133.0, 132.0, 130.4, 129.3, 129.1, 128.6, 128.3, 127.8, 126.6, 126.3, 123.2, 65.9, 39.7

IR (neat, ν cm^{-1}): 3028, 2928, 1674, 1446, 1257, 1144, 817

HRMS (ESI-): *m/z* calcd for $C_{23}H_{16}{}^{35}ClO$ $[M-H]^-$ 343.0895; found 343.0881

(2-Bromo-1,3-diphenylcyclobut-2-en-1-yl)(phenyl)methanone, 6b

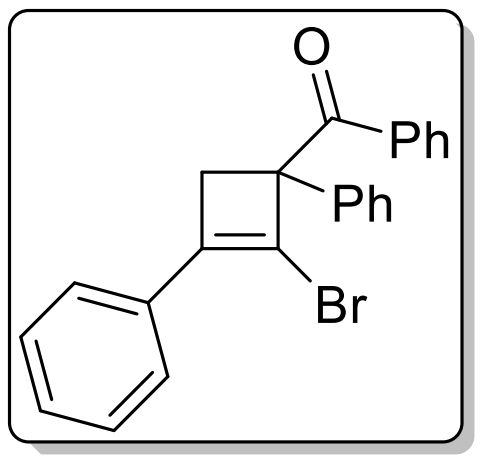

Prepared following **General Procedure 5** using *4-bromo-2,2,5-triphenyl-1-oxaspiro[2.3]hex-4-ene* **3a** (77 mg, 0.20 mmol). Purification by flash column chromatography (hexane to 90:10 hexane:Et_2O) yielded the title product **6b** as a colourless oil (31 mg, 0.081 mmol, 41%).

^{1}H-NMR (400 MHz, $CDCl_3$): δ_H 7.86 – 7.79 (2H, m), 7.71 (2H, dd, *J* = 7.9, 1.7 Hz), 7.49 – 7.29 (11H, m), 3.82 (1H, d, *J* = 11.4 Hz) and 3.30 (1H, d, *J* = 11.4 Hz) (*AB syst.*)

^{13}C{^{1}H}-NMR (101 MHz, $CDCl_3$): δ_C 197.9, 144.5, 139.3, 135.6, 133.0, 132.3, 130.4, 129.3, 129.3, 128.6, 128.3, 127.8, 126.3, 126.2, 113.3, 65.2, 41.4

IR (neat, v cm^{-1}): 3025, 2925, 1674, 1597, 1491, 1258, 1181, 1023, 936

HRMS (ESI-): *m/z* calcd for $C_{23}H_{16}{}^{79}BrO$ [M-H]$^-$ 387.0390; found 387.0385

(2-Fluoro-1,3-diphenylcyclobut-2-en-1-yl)(phenyl)methanone, 6c

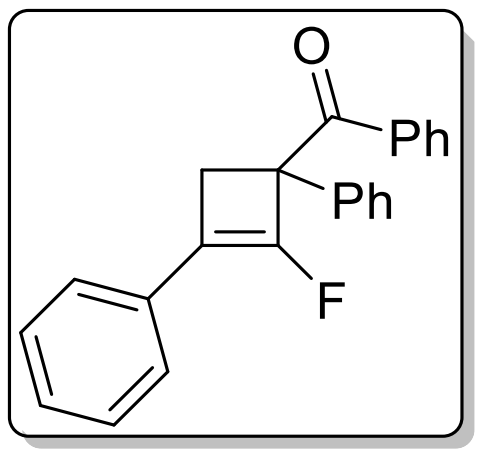

Prepared following **General Procedure 5** using *4-fluoro-2,2,5-triphenyl-1-oxaspiro[2.3]hex-4-ene* **4a** (62 mg, 0.19 mmol). Purification by flash column chromatography (hexane to 98:2 hexane:Et_2O) yielded the title product **6c** as a colourless oil (27 mg, 0.081 mmol, 41%).

^{1}H-NMR (400 MHz, $CDCl_3$): δ_H 7.92 (2H, d, *J* = 7.8 Hz), 7.52 – 7.42 (5H, m), 7.41 – 7.28 (8H, m), 3.55 (1H, dd, *J* = 14.2, 10.1 Hz) and 2.60 (1H, dd, *J* = 13.6, 10.0 Hz) (*AB syst.*)

^{19}F-NMR (377 MHz, $CDCl_3$): δ_F -90.77 (t, *J* = 13.9 Hz)

^{13}C{^{1}H}-NMR (101 MHz, $CDCl_3$): δ_C 196.6 (d, *J* = 5.4 Hz), 143.0 (d, *J* = 355.1 Hz), 139.1 (d, *J* = 5.0 Hz), 135.8, 133.1, 131.0 (d, *J* = 5.4 Hz), 130.1 (d, *J* = 2.4 Hz), 129.3, 128.7, 128.4, 127.8, 127.0 (d, *J* = 4.7 Hz), 126.2, 118.7 (d, *J* = 6.3 Hz), 67.5 (d, *J* = 20.0 Hz), 34.4 (d, *J* = 21.2 Hz)

IR (neat, v cm^{-1}): 3027, 2932, 1703, 1674, 1597, 1494, 1298, 1123, 987

HRMS (ESI+): *m/z* calcd for $C_{23}H_{18}FO$ $[M+H]^+$ 329.1336; found 329.1327

(4-(4-Chloro-2,5-diphenyl-1-oxaspiro[2.3]hex-4-en-2-yl)phenyl)(imino)(*p*-tolyl)-λ^6-sulfanone, 7

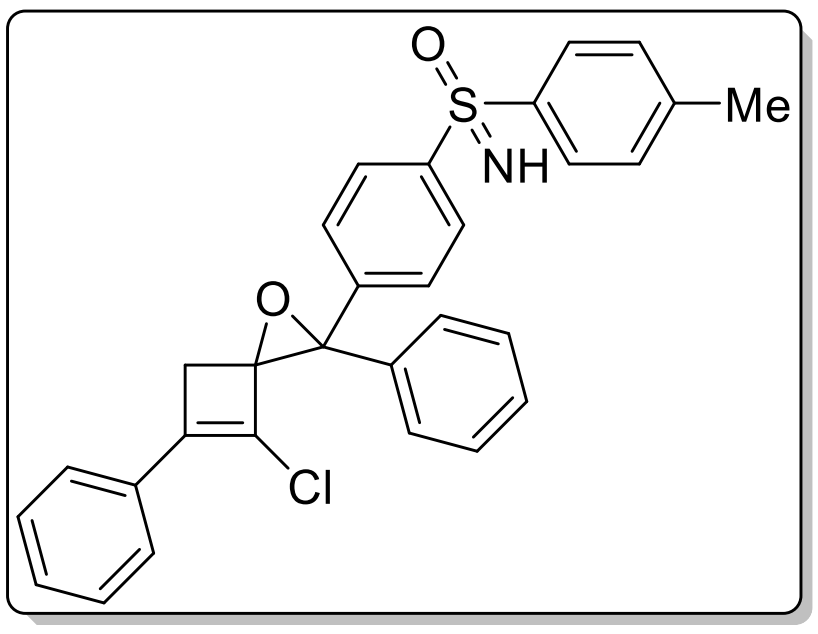

To a solution of *4-chloro-2,5-diphenyl-2-(4-(p-tolylthio)phenyl)-1-oxaspiro[2.3]hex-4-ene* **2f** (45 mg, 0.10 mmol, 1 equiv.) and *ammonium carbamate* (15 mg, 0.20 mmol, 2 equiv.) in methanol (1 mL) was added *(diacetoxyiodo)benzene* (40 mg, 0.125 mmol, 1.25 equiv.) in small portions. The resulting suspension was stirred for three hours at room temperature, before it was concentrated under reduced pressure and diluted with EtOAc (10 mL). It was then washed with saturated aqueous sodium bicarbonate solution (3x5 mL) and saturated aqueous sodium chloride solution (5 mL). The organic phase was dried over sodium sulfate, filtered, and concentrated under reduced pressure, to afford the crude material as a 1:1:1:1 mixture of diastereomers. Purification by flash column chromatography (1:1 hexane:EtOAc) afforded the title compound **7** (23 mg, 0.047 mmol, ca. 1:1:1:1 dr, 47%) as a white solid.

Data reported for mixture of diastereomers ($d_1 + d_2 + d_3 + d_4$)

^{1}H-NMR (400 MHz, $CDCl_3$): δ_H 8.11 – 7.88 (16H, m, $d_1 + d_2 + d_3 + d_4$), 7.73 – 7.66 (4H, m, $d_1 + d_2 + d_3 + d_4$), 7.61 – 7.47 (16H, m, $d_1 + d_2 + d_3 + d_4$), 7.43 – 7.27 (30H, m, $d_1 + d_2 + d_3 + d_4$), 7.24 – 7.13 (6H, m, $d_1 + d_2 + d_3 + d_4$), [3.01 (4H, dd, *J* = 11.7, 5.3 Hz), 2.96 – 2.87 (2H + 1H -*NH*, m), 2.80 (2H, d, *J* = 11.6 Hz), $d_1 + d_2 + d_3 + d_4$], 2.40 (3H, s), 2.38 (2x3H, s), 2.36 (3H, s)

^{13}C{^{1}H}-NMR (101 MHz, $CDCl_3$): δ_C 145.1, 145.0, 114.3, 143.7, 140.4, 138.3, 131.5, 130.0, 130.0, 129.8, 129.2, 129.2, 129.1, 129.0, 128.8, 128.6, 128.6, 128.6, 128.4, 128.4, 128.3, 128.2, 128.1, 128.1, 127.9, 127.8, 127.5,127.2, 126.9, 126.7, 126.7, 125.4, 75.0, 74.7, 67.8, 67.8, 36.4, 36.2, 21.6

IR (neat, ν cm^{-1}): 3321, 3059, 3028, 2924, 2856, 1595, 1490, 1229, 1024, 947

HRMS: m/z calcd for $C_{30}H_{25}{}^{35}ClNO_2S$ $[M+H]^+$ 498.1289; found 498.1293

Note: Some signals are doubled due to the presence of diastereomers.

4-(3-(4-Chloro-2,5-diphenyl-1-oxaspiro[2.3]hex-4-en-2-yl)phenyl)morpholine, 8

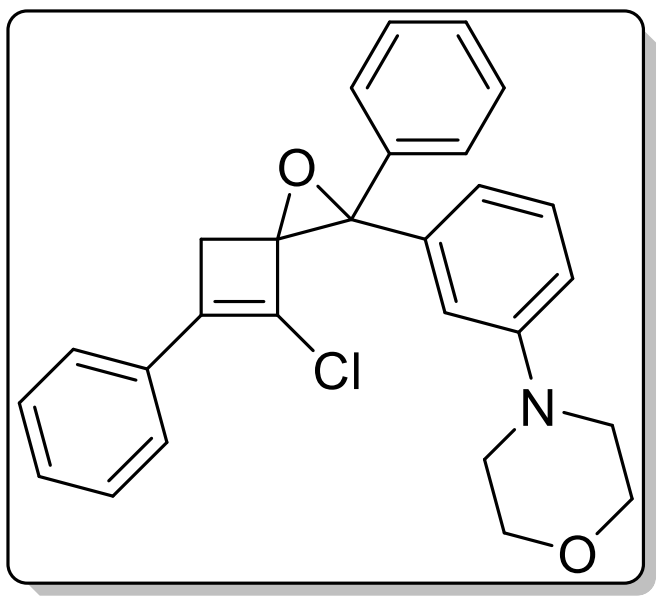

To a solution of *2-(3-bromophenyl)-4-chloro-2,5-diphenyl-1-oxaspiro[2.3]hex-4-ene* **2e** (110 mg, 0.26 mmol) and *morpholine* (22 µL, 0.26 mmol) in toluene (3 mL) at room temperature were added *tris(dibenzylideneacetone)dipalladium(0)* (7 mg, 0.080 mmol, 3 mol%), *XPhos* (15 mg, 0.031 mmol, 12 mol%) and sodium tert-butoxide (75 mg, 0.78 mmol) consecutively. The reaction mixture was allowed to stir at 100 °C for 18 h. Once the mixture has been cooled to room temperature, it was filtered through a short pad of celite and concentrated under reduced pressure to afford the crude material as a 1:1 mixture of diastereomers. Purification by flash column chromatography (10:1 hexane:EtOAc to 1:1 hexane:EtOAc) yielded the title product **8** as a white solid (35 mg, 0.082 mmol, ca. 70:30 dr, 31%).

Data reported for mixture of diastereomers

^{1}H-NMR (400 MHz, $CDCl_3$): δ_H 7.80 – 7.64 (2H, m), 7.59 – 7.32 (10H, m), 7.22 – 6.95 (2H, m), 4.07 – 4.93 (4H, m), 3.42 – 3.23 (4H, m), 3.16 (1H, dd, *J* = 11.5, 5.9 Hz), 3.04 (1H, app. t, *J* = 12.3 Hz)

^{13}C{^{1}H}-NMR (101 MHz, $CDCl_3$): δ_C 144.8, 144.7, 139.5, 137.8, 136.9, 131.7, 129.6, 129.2, 128.9, 128.7, 128.5, 128.3, 128.1, 127.8, 127.1, 126.8, 125.6, 118.9, 116.0, 74.9, 74.8, 68.6, 68.9, 49.6, 49.4, 36.5, 36.4

IR (neat, v cm^{-1}): 3028, 2961, 2920, 2855, 2825, 1600, 1581, 1446, 1232, 1000, 908

HRMS (ESI+) *m/z* calcd for $C_{27}H_{25}{}^{35}ClNO_2$ $[M+H]^+$ 430.1568; found 430.1562

Note: Some signals are doubled due to the presence of diastereomers.

4-(2,5-Difluorophenyl)-2,2,5-triphenyl-1-oxaspiro[2.3]hex-4-ene, 9

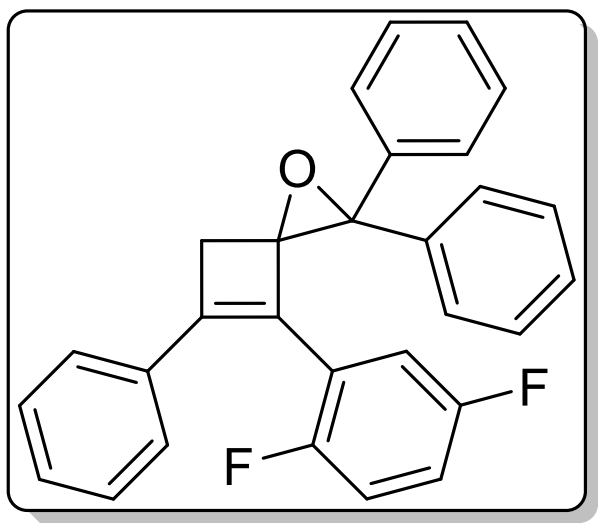

Prepared following a previously reported procedure.[5]

To a solution of *4-bromo-2,2,5-triphenyl-1-oxaspiro[2.3]hex-4-ene* **3a** (40 mg, 0.10 mmol) and *(2,5-difluorophenyl)boronic acid* (20 mg, 0.12 mmol, 1.2 equiv.) in anhydrous THF (0.1 M) at room temperature were added *palladium (II) acetate* (2 mg, 0.0050 mmol, 5 mol%), *XPhos* (5 mg, 0.010 mmol, 10 mol%) and *potassium phosphate tribasic* (2 M in H_2O, 0.15 mL, 3.0 equiv.) consecutively. The reaction mixture was allowed to stir at 70 °C for 18 h. Upon completion, the mixture was cooled to room temperature and quenched with aqueous saturated $NaHCO_3$ solution; it was then extracted with ethyl acetate (3x5 mL), dried with $MgSO_4$ and concentrated under reduced pressure to afford the crude material. Purification by flash column chromatography (2% Et_2O/hexane) yielded the title product **9** as a colourless oil (12 mg, 0.028 mmol, 28%).

^{1}H-NMR (400 MHz, $CDCl_3$): δ_H 7.42 – 7.28 (4H, m), 7.35 – 7.28 (8H, m), 7.11 – 7.01 (3H, m), 6.79 (1H, td, *J* = 8.8, 4.5 Hz), 6.73 – 6.63 (2H, m), 3.14 (2H, app. d, *J* = 2.2 Hz)

^{19}F-NMR (377 MHz, $CDCl_3$): δ_F -116.49 (m), -120.17 (m)

^{13}C{^{1}H}-NMR (101 MHz, $CDCl_3$): δ_C 157.8 (dd, *J* = 271.4, 2.4 Hz), 155.4 (dd, *J* = 273.3, 2.4 Hz), 147.6 (d, *J* = 1.6 Hz), 146.1, 139.9, 137.1, 129.4, 128.5, 128.3, 128.3, 127.6, 127.6, 127.1, 127.1, 126.8, 122.8 (dd, *J* = 19.2, 8.9 Hz), 120.5, 116.6 (dd, *J* = 24.7, 3.9 Hz), 116.1 (dd, *J* = 24.9, 8.9 Hz), 115.5 (dd, *J* = 24.3, 8.5 Hz), 73.7, 68.5, 37.9

IR (neat, v cm^{-1}): 3028, 2923, 2850, 1602, 1586, 1496, 1487, 1341, 1246, 907

HRMS (ESI+) *m/z* calcd for $C_{29}H_{20}F_2ONa$ $[M+Na]^+$ 445.1374; found 445.1383

2,2,5-Triphenyl-4-(phenylethynyl)-1-oxaspiro[2.3]hex-4-ene, 10

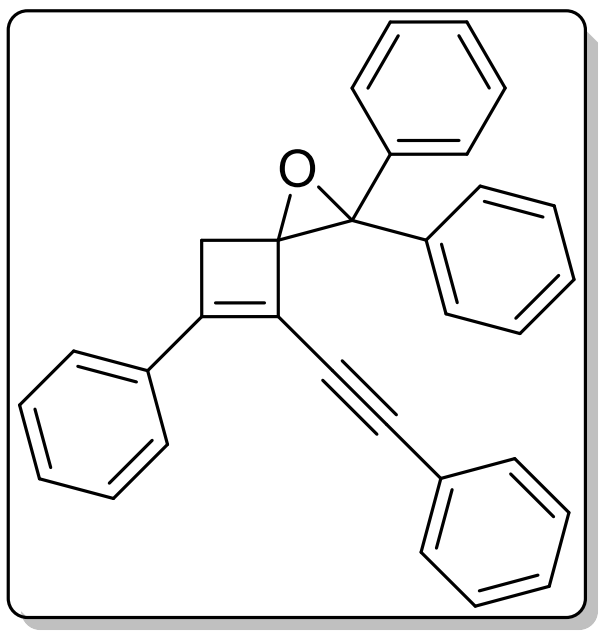

Prepared following a previously reported procedure.[5]

To a solution of *4-bromo-2,2,5-triphenyl-1-oxaspiro[2.3]hex-4-ene* **3a** (40 mg, 0.10 mmol), *Pd(PPh$_3$)$_4$* (6 mg, 0.0050 mmol, 5 mol%) and *CuI* (2 mg, 0.010 mmol, 10 mol%) in anhydrous THF (0.1 M) at room temperature were added *phenyl acetylene* (13 µL, 0.12 mmol, 1.2 equiv.) and *triethylamine* (17 µL, 0.12 mmol, 1.2 equiv.) consecutively. The reaction mixture was allowed to stir under a Nitrogen atmosphere at 70 °C for 16 h. Upon completion, the mixture has been cooled to room temperature and quenched with saturated $NaHCO_3$ solution; it was then extracted with ethyl acetate (3x5 mL), dried with $MgSO_4$ and concentrated under reduced pressure to afford the crude material. Purification by flash column chromatography (2% Et_2O/hexane) yielded the title product **10** as a yellow oil (7 mg, 0.017 mmol, 17%).

^{1}H-NMR (400 MHz, $CDCl_3$): δ_H 7.83 – 7.79 (1H, m), 7.76 – 7.71 (1H, m), 7.67 – 7.63 (2H, m), 7.43 – 7.32 (14H, m), 7.12 – 7.05 (2H, m), 3.14 (1H, d, *J* = 14.3 Hz) and 2.99 (1H, d, *J* = 14.3 Hz) (*AB syst.*)

^{13}C{^{1}H}-NMR (101 MHz, $CDCl_3$): δ_C 151.9, 139.9, 137.6, 133.5, 132.6, 131.9, 130.2, 129.7, 128.9, 128.7, 128.4, 128.3, 128.2, 128.1, 127.0, 126.9, 122.9, 119.1, 97.4, 82.4, 73.4, 68.4, 37.4

IR (neat, ν cm^{-1}): 3058, 3028, 2920, 1663, 1598, 1446, 1366, 1277, 1067, 915

HRMS (ESI+) *m/z* calcd for $C_{31}H_{22}ONa$ $[M+Na]^+$ 433.1563; found 433.1577

5. X-Ray Crystallography

The X-ray data were acquired at room temperature with a Bruker AXS X8 APEXII automated diffractometer, equipped with a CCD detector and graphite-monochromatized Mo Kα radiation (λ = 0.71073 Å) operating at 50 kV, 30 mA and 40 mm crystal-to-detector distance. Relevant crystallographic data of the investigated crystals is reported in **Table S1**. The whole Ewald sphere (±h,±k,±l) was recorded up to θ~30° with a scan ranging from 0.5 to 0.8°/frame and an exposure time ranging from 180 to 240 s/frame. The APEX program suite allowed to optimize the collection strategy;[6] the SAINT package[7] was used for the integration of the intensities of reflections and the correction of the Lorentz and polarization effects; the SADABS software[8] was employed for the empirical absorption correction; XPREP[9] was used for the subsequent analysis of the intensity data and the assignment of the space group. Structure was solved by charge flipping method[10] and by full-matrix least-square analysis using the program CRYSTALS.[11] All reflections are included in the refinement. The overall scale factor, atomic positions and anisotropic atomic displacement parameters for non-hydrogen atoms were refined. All hydrogen atoms were located and refined isotropically through ride restraints conditions.[12] All three refined molecules exhibit positional disorder localized on a four-membered carbon ring, which is present in two alternative orientations related by a 180° flip. This disorder likely arises from the low energy barrier associated with ring inversion or rotation, allowing both configurations to be populated in the crystal. The structural model accounts for this behavior by refining the disordered ring over two positions with appropriate occupancy factors. The refinement converged successfully, yielding a occupancy ratios for the two orientations are 64.5:34.5 for **2b**, 72.6:27.4 for **2d**, and 59.7:40.3 for **3d**.

Table S1. X-ray crystal data of the studied crystals.

	**2b**	**2d**	**3d**
Crystal size (mm)	0.46 × 0.23 × 0.16	0.58 × 0.21 × 0.10	0.60 × 0.45 × 0.41
Chemical formula	$C_{25}H_{21}ClO$	$C_{23}H_{15}Cl_3O$	$C_{23}H_{15}BrCl_2O$
M_r	372.89	413.73	458.18
Crystal system, space group	Monoclinic, *P2₁/c*	Orthorhombic, *Pca2₁*	Orthorhombic, *Pca2₁*
a, *b*, *c* (Å)	5.8099 (7), 22.438 (3), 15.4243 (17)	22.4849 (13), 5.6528 (3), 15.5441 (8)	22.8596 (14), 5.7044 (3), 15.4792 (9)
a, b, g (°)	90, 92.751 (6), 90	90, 90, 90	90,90, 90
V (Å^3)	2008.4 (4)	1975.70 (19)	2018.5 (2)
$R[F^2 > 2s(F^2)]$, $wR(F^2)$, S	0.072, 0.220, 0.91	0.055, 0.104, 0.92	0.103, 0.206, 1.05
$\Delta\rho_{max}$, $\Delta\rho_{min}$ (e Å^{-3})	0.47, -0.50	0.47, -0.37	0.71, -0.69

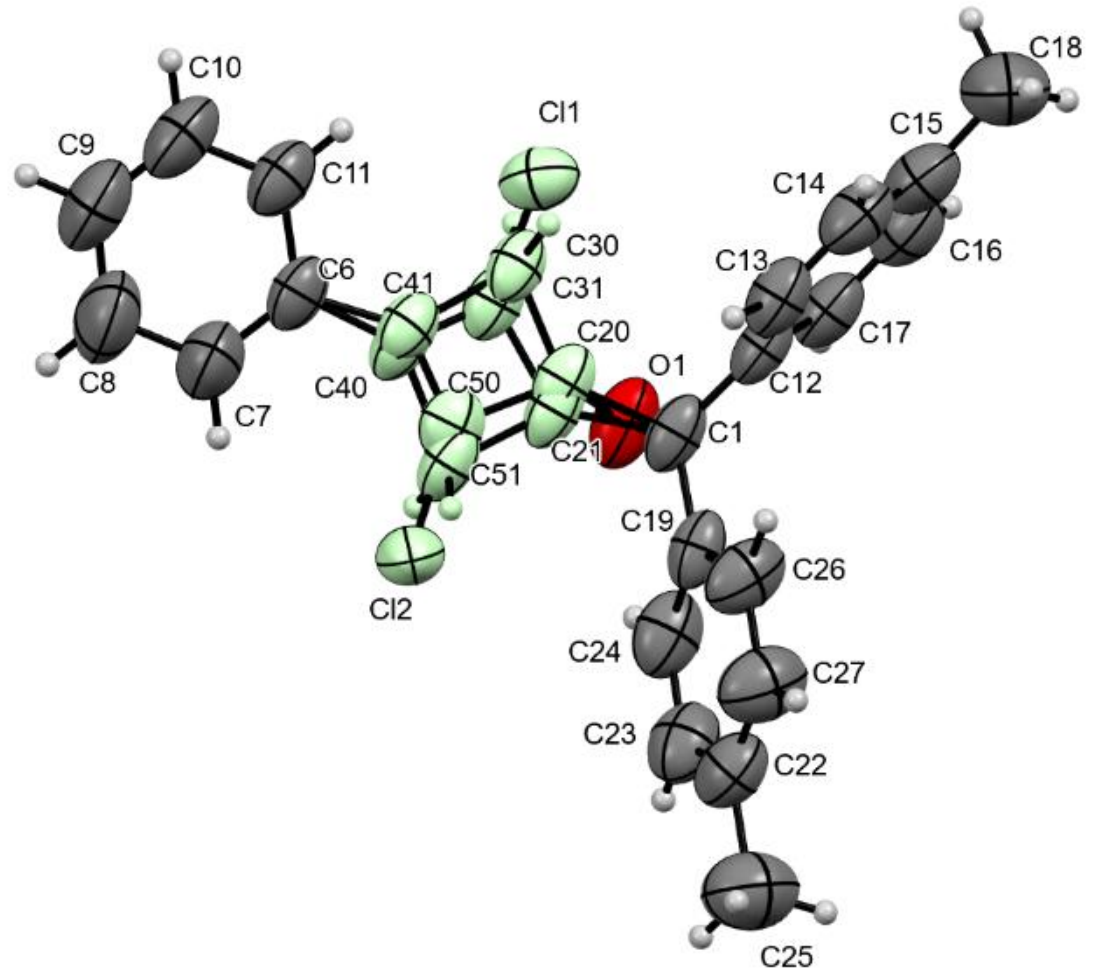

Figure S1. ORTEP drawing of compound **2b**. The light green color represents the atoms affected by positional disorder. The displacement ellipsoids are drawn at the 50% probability level. Hydrogen atoms labels are omitted for clarity.

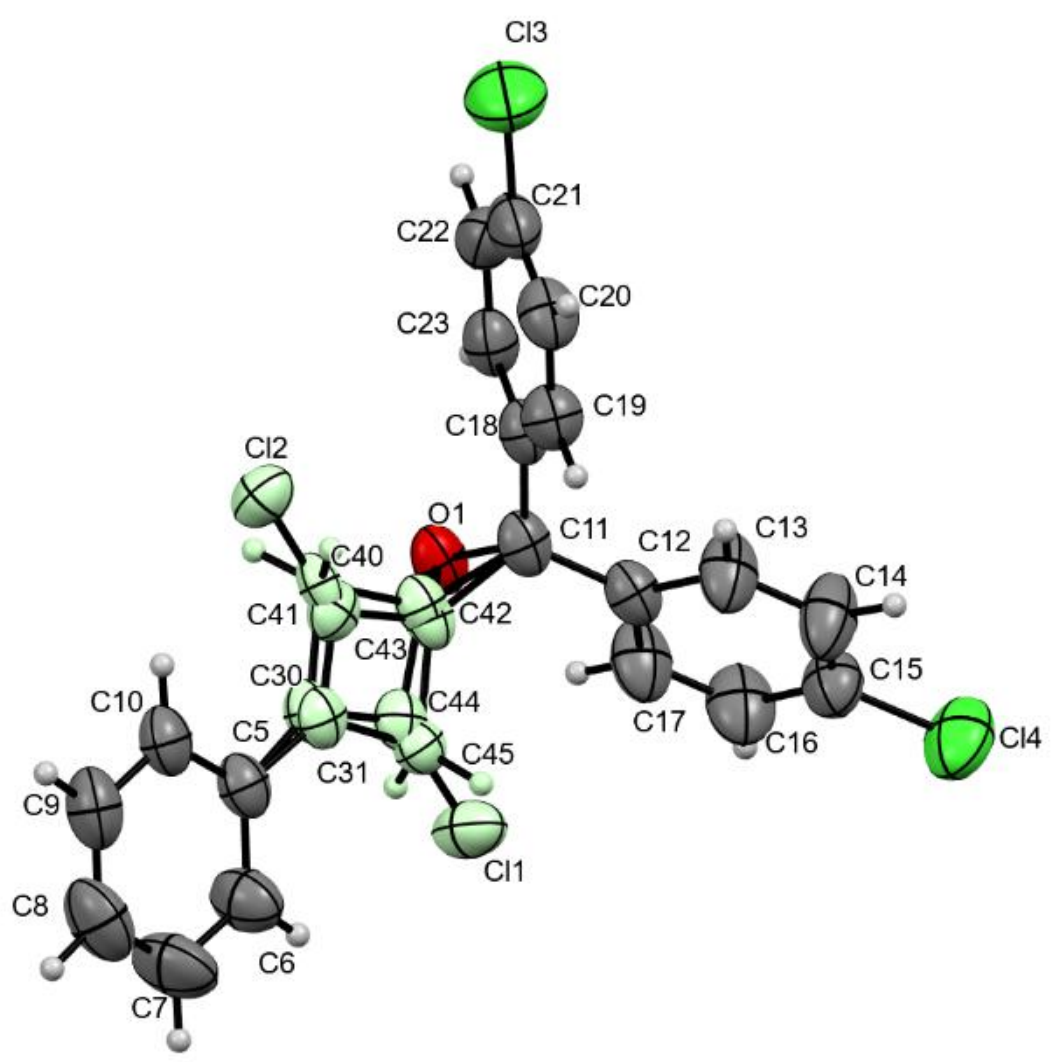

Figure S2. ORTEP drawing of compound **2d**. The light green color represents the atoms affected by positional disorder. The displacement ellipsoids are drawn at the 50% probability level. Hydrogen atoms labels are omitted for clarity.

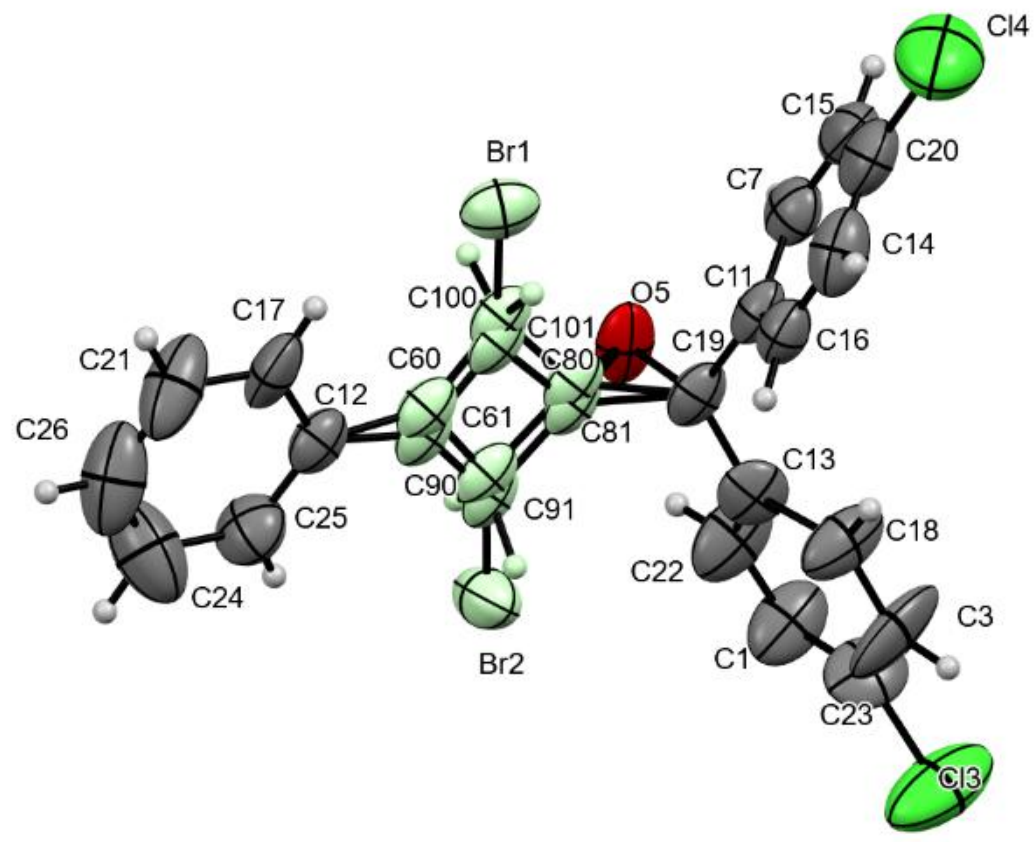

Figure S3. ORTEP drawing of compound **3d**. The light green color represents the atoms affected by positional disorder. The displacement ellipsoids are drawn at the 50% probability level. Hydrogen atoms labels are omitted for clarity.

6. Computational Calculations

1. Computational methodology

Density functional theory (DFT) calculations are performed using package Gaussian09.[13] The current work involves non-covalently bonded complexes, and M06-2X method is considered as suitable for non-covalent interactions. It has also been shown that long-range and dispersion corrected DFT methods (such as M05 and M06) perform significantly better for such systems.[14,15] Therefore, the optimization of geometry is performed at M06-2X/6-31+G(d,p) level of theory. To confirm the presence of single imaginary frequency at the transition states, frequency analyses are performed.[16] The absence of any imaginary frequencies is also used to confirm minimum or stationary points. Moreover, the transition states are also corroborated through the animation of negative frequency along the reaction axis.[17–20]

Furthermore, because of its well-documented accuracy, the B3LYP/6-31G(d,p) level of theory is also used for the assessment of electronic properties like frontier molecular orbital (FMO) analysis and chemical reactivity descriptors.[21–23] FMO analysis is performed to evaluate the electronic features of carbene structures, the energy gaps are calculated by the following equation:

$$\Delta E = E_{LUMO} - E_{HOMO} \quad (1)$$

Some important global reactivity descriptors such as chemical potential (μ), hardness (η), and electrophilicity index (ω) etc., have been evaluated. According to Koopmans theorem, HOMO and LUMO energy values can be used to predict the ionization potential and electron affinity values, respectively.[24] Moreover, chemical potential (μ), hardness (η) and softness (σ) are also by using Koopmans theorem through eq (4), (5) and (6), respectively.[25]

$$IP = -E_{HOMO} \quad (2)$$

$$EA = -E_{LUMO} \quad (3)$$

$$\mu = -\frac{IP+EA}{2} \quad (4)$$

$$\eta = \frac{IP-EA}{2} \quad (5)$$

$$\sigma = \frac{1}{2\eta} = \frac{1}{IP-EA} \quad (6)$$

Similarly, the inverse of nucleophilicity (electrophilicity index (ω)) and inverse of chemical potential (electronegativity (χ)) are also computed as:

$$\omega = \frac{\mu^2}{2\eta} \tag{7}$$

$$\chi = \frac{IP+EA}{2} \tag{8}$$

1.1 Features of carbene structures

1.1.1 Molecular electrostatic potential

Molecular electrostatic potential (MEP) is a power parameter to evaluate and validate the reactivity of a chemical compound for electrophilic/nucleophilic attack.[26] The MEP map of considered carbene structures i.e., dichlorocarbene, dibromocarbene and flourobromocarbene are presented in **Figure S1**, where the higher negative region (red-colored) favors electrophilic attack. Therefore, nucleophilic sites are likely to attack the electrophilic group and *vice versa*.

The MEP maps of dichlorocarbene and dibromocarbene reveal that halogen atom exhibit cyan blue colored region which corresponds to higher positive charge. Whereas the carbon atoms have reddish-orange color region (negative potential). Thus, the red-colored regions (carbon atoms) are more likely involved in nucleophilic attack and blue-colored regions (halogen atoms) in electrophilic attack. In the case of flourobromocarbene, higher positive charge (dark blue color region) appeared at the C—F bond as compared to C—Br bond. The MEP shows that bromine holds its electron density more loosely (more polarizable) than fluorine.

**Carbene**	**MEP**
	**$-2.510e^{-2}$** 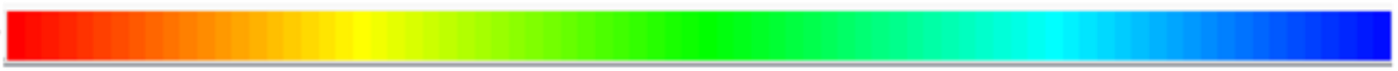 **$2.510e^{-2}$**
**CCl_2**	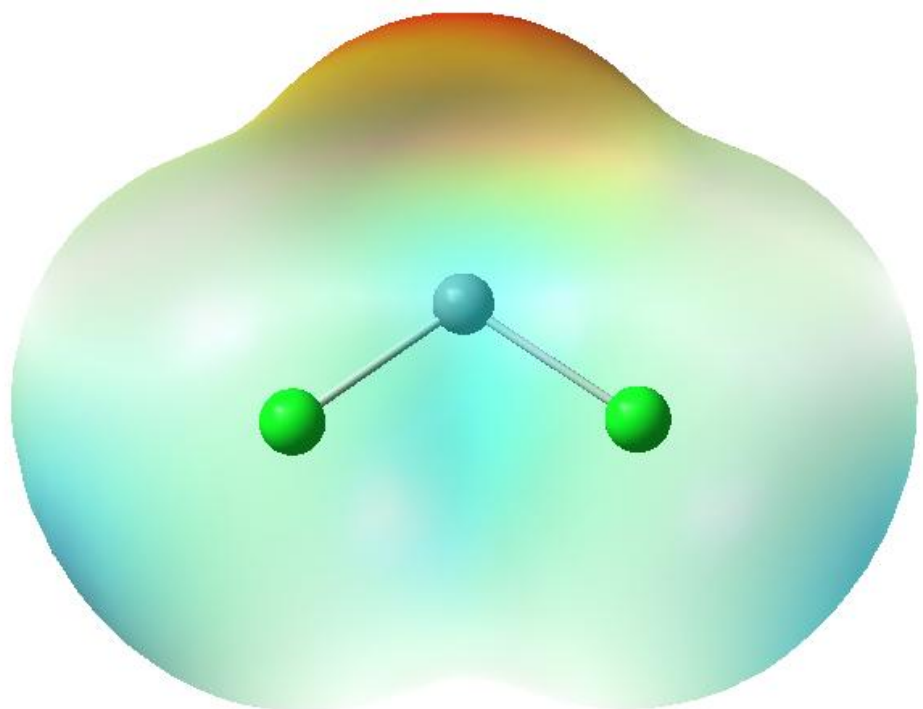

**CBr_2**	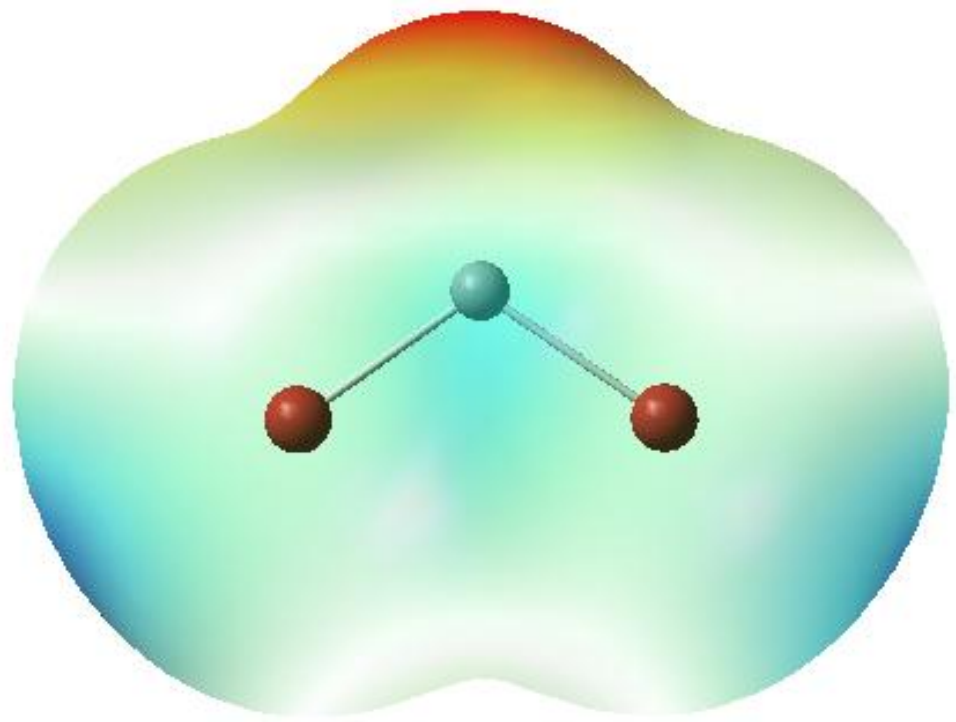
**CFBr**	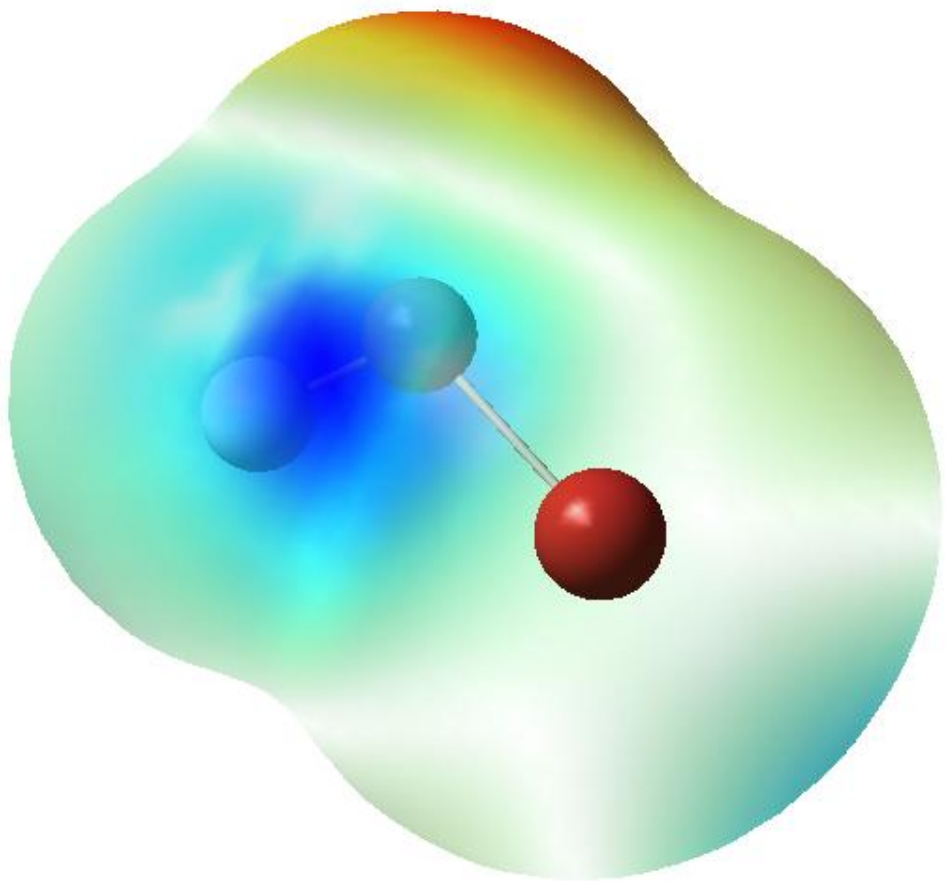

Figure S1. Molecular Electrostatics Potential (MEP) of carbene structures

1.1.2 Frontier molecular orbital analysis

Frontier molecular orbital (FMO) analysis is frequently used to study the electronic behavior of the chemical compounds. Herein, FMO analysis is performed to get insight into the electronic features of carbene structures i.e., dichlorocarbene, dibromocarbene and flourobromocarbene. The highest occupied molecular orbital i.e., HOMO can donate electrons, therefore, corresponds to ionization energy (I.P.). On the other hand, the lowest unoccupied MO can accept electrons and related with the electron affinity (E.A.).[27]

The HOMO and LUMO orbital densities of carbene structures are shown in **Figure S2**, whereas the calculated HOMO and LUMO energies values and corresponding energy gaps are reported in **Table S1**. **Figure S2** reveals that the HOMO and LUMO orbital densities are uniformly distributed over both halogen atoms in case of both dichlorocarbene and dibromocarbene. However, in the case of flourobromocarbene, the orbital densities are unevenly distributed with greater electronic density on bromine atom as compared to fluorine. The energy gaps calculated are 6.42, 5.86 and 7.19 eV observed for dichlorocarbene, dibromocarbene and flourobromocarbene, respectively. For dichlorocarbene, the values of -8.79 eV and -2.36 eV for HOMO and LUMO energy are seen, respectively. Similarly, for dibromocarbene, the HOMO and LUMO energy values are -8.53 eV and

-2.67 eV, respectively. Whereas the HOMO-LUMO values for flourobromocarbene are -9.22 eV and -2.03 eV, respectively. The lower the value of HOMO-LUMO energy gap, higher the reactivity of that compound will be. Similarly, the lower LUMO values declare good electron-acceptability power whereas a higher HOMO value is associated with the higher electron-donating ability of carbene.[28]

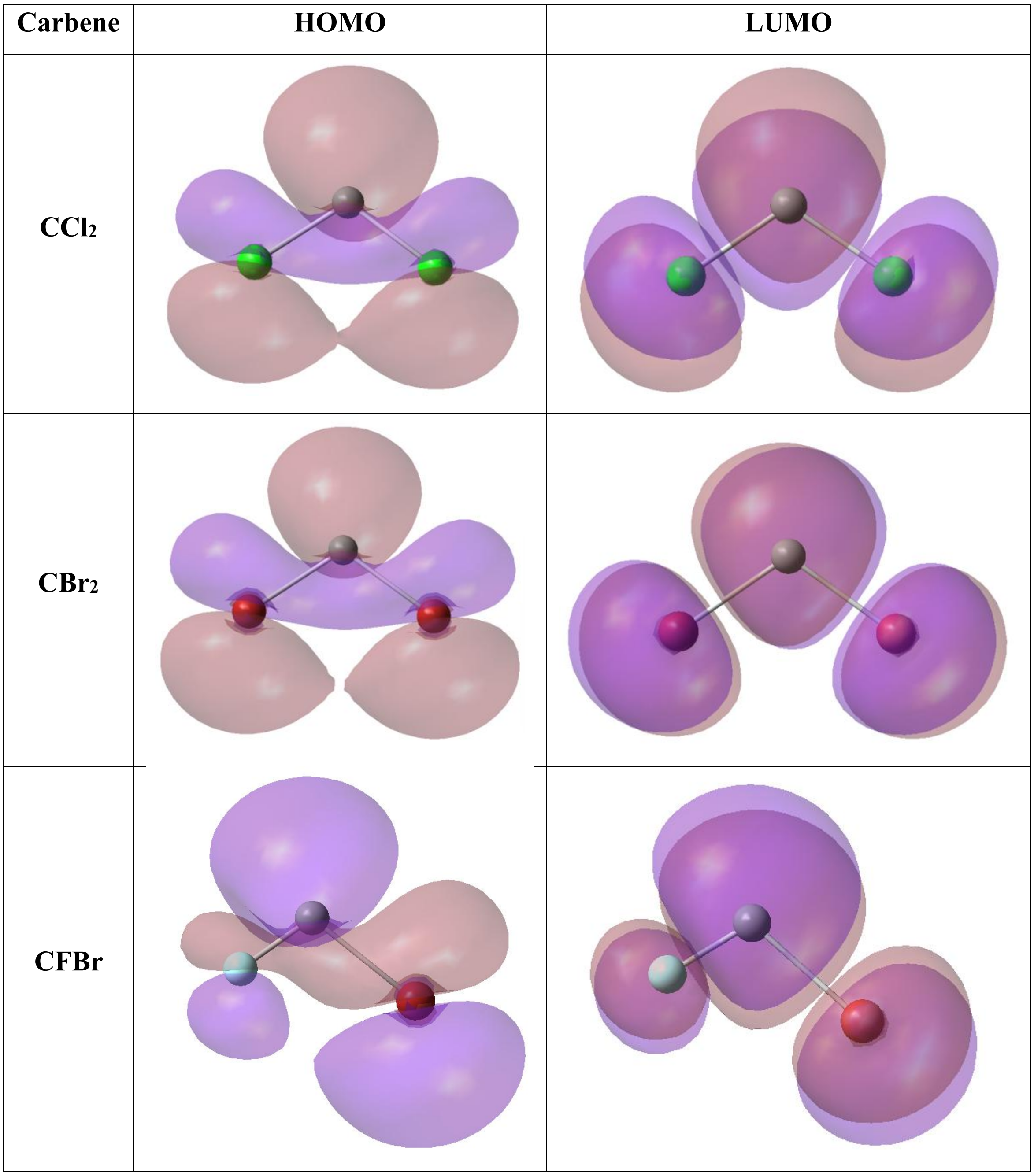

Figure S2. HOMO-LUMO orbital densities of carbene structures

1.1.3 Chemical Reactivity Descriptors

DFT simulated global reactivity descriptors are employed to understand the structural stability of the carbene structures and their reactivities by using electron affinity (EA), ionization potential (IP),

electronegativity (χ), softness (σ), hardness (η), electrophilicity index (ω) and chemical potential (μ). By employing Koopmans theorem, the HOMO energy is related to IP, and the LUMO energy is associated with EA as shown in equation 2 and 3, respectively. Moreover, the other factors of GRD are given in equation 4 to 8 given in methodology section.

The average energy of HOMO (IP) and LUMO (EA) is known as electronegativity, and it is used to gauge how much an atom or collection of atoms can draw electrons to itself. Furthermore, the idea of global electrophilicity (ω) was initially proposed by Parr *et al.,* who believed that electrophilicity was a better descriptor because it gave enough information about the complexes' hardness and chemical potential. An electrophile's capacity to retain its electronic structure is demonstrated by both its resistance to external perturbations and its capacity to gain extra electronic charge. The ability of an atom or collection of atoms to leave the equilibrium system is known as its electronic chemical potential (μ). The ability of an atom or collection of atoms to withstand electron charge exchange or to preserve the integrity of its electron cloud in the face of small disruptions during chemical reactions is known as global hardness. Conversely, the likelihood of an atom or group of atoms to receive electrons from the electrophilic site is known as softness (σ).

The values of IE, EA, η, σ, ω, μ and χ are shown in **Table S1**. The three carbene systems have different capacities for electronic excitation and charge transfer, as demonstrated by the frontier-orbital-based descriptors (E_{LUMO}, E_{HOMO}, and the energy gap E_g). With the lowest IP (8.53 eV), the least negative E_{HOMO} (−8.53 eV), and the smallest energy gap (E_g = 5.86 eV), CBr_2 has a higher overall tendency for global reactivity and a relatively simpler electron-removal process. While CFBr shows the largest Eg (7.19 eV) and higher hardness (η = 3.59 eV), indicating greater resistance to charge-transfer-type interactions, CCl_2 is intermediate in both HOMO/LUMO positions and E_g (6.42 eV), suggesting moderate reactivity. Despite having the highest E_{LUMO} (−2.03 eV), which alone suggests a favourable electron-accepting capability in orbital terms, overall global reactivity of CFBr is reduced in accordance with conceptual DFT expectations due to its larger E_g and greater hardness.

This general picture is supported by the charge-transfer descriptors: CBr_2 is the most electrophilic and most likely to accept electron-density on a global scale, according to softness/chemical hardness (σ and η) and electrophilic indices (χ and ω). In particular, CBr_2 exhibits stronger electrophilic behaviour than the other systems due to its high electronegativity and electrophilicity index (χ = 5.60 eV, ω = 5.35 eV), as well as its lowest hardness (η = 2.93 eV) and highest softness (σ = 0.17 eV). Despite having the highest χ (5.63 eV), CFBr maintains a relatively smaller ω (4.40 eV) due to its larger η and Eg limit charge transfer, whereas CCl_2 exhibits lower χ and ω (χ = 4.39 eV, ω = 3.52 eV), consistent with less electrophilic character. Consequently, the reactivity/electrophilicity order of CBr_2

> CCl_2 > CFBr is supported by the combined trend across HOMO/LUMO, E_g, and other global reactivity descriptors.

Table S1: Summarized results of chemical reactivity parameters such as HOMO, LUMO energies, energy gap (E_g), ionization potential (IP), electron affinity (EA), chemical potential (μ), chemical hardness (η), chemical softness (σ), electronegativity (χ), and electrophilicity index (ω) (all the parameters are reported in eV).

**Carbene**	**E_{LUMO}**	**E_{HOMO}**	**E_g**	**IP**	**EA**	**μ**	**η**	**σ**	**χ**	**ω**
CCl_2	-2.36	-8.79	6.42	8.79	2.36	5.57	3.21	0.16	4.39	3.52
CBr_2	-2.67	-8.53	5.86	8.53	2.67	5.60	2.93	0.17	5.60	5.35
CFBr	-2.03	-9.22	7.19	9.22	2.03	5.63	3.59	0.14	5.63	4.40

Optimized Geometries, Energies (Hartree) and Cartesian Coordiantes

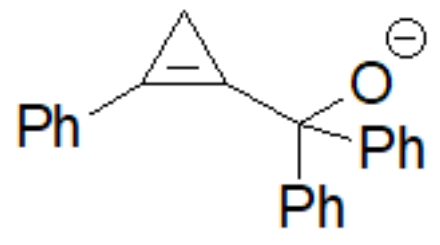

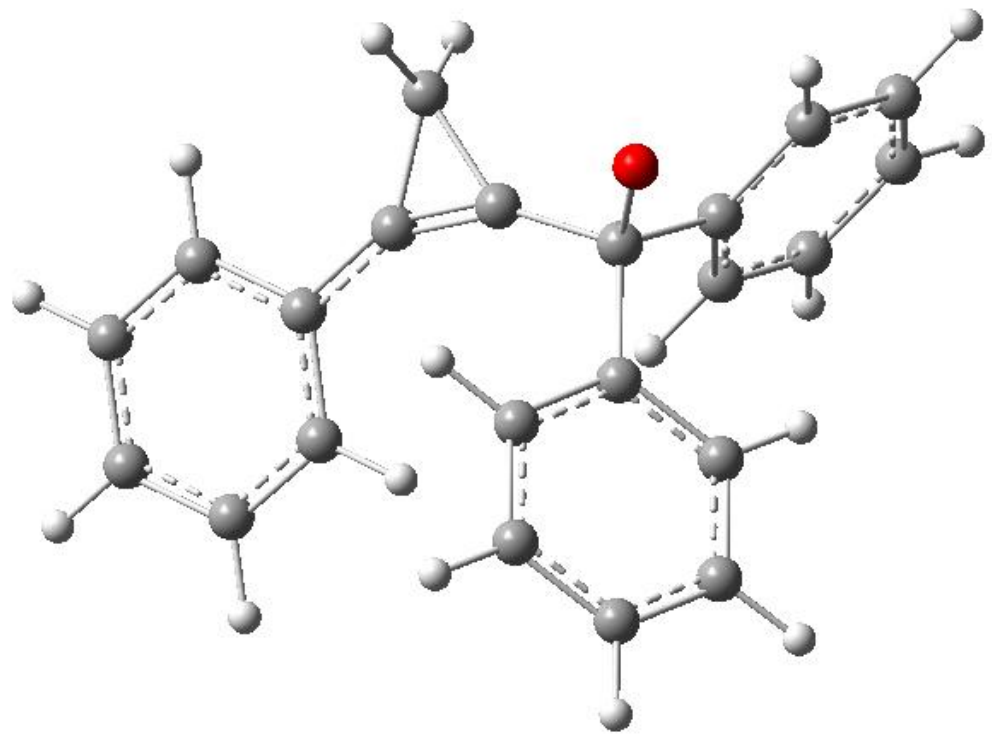

Total Electronic Energy	Sum of electronic and zero-point Energies	Sum of electronic and thermal Enthalpies	Sum of electronic and thermal Free Energies
-923.397940	-923.291231	-923.276581	-923.326762

Cartesian Coordinates

C	0.04215200	-0.69891100	1.18860200
C	1.26803800	-1.03548600	0.92691500
C	0.52977500	-1.93300700	1.88149600
H	0.18874900	-2.91549300	1.54236700
H	0.74785700	-1.89334200	2.95101500
C	2.52337800	-0.87842600	0.22499100
C	2.72367900	0.16453700	-0.68820400
C	3.55935200	-1.78738500	0.45748400
C	3.93582900	0.28947800	-1.35009900
H	1.92443300	0.87559800	-0.86743300
C	4.77070200	-1.65924500	-0.20823800
H	3.40000800	-2.59373200	1.16571000
C	4.96200200	-0.62111700	-1.11312100
H	4.08251300	1.10100900	-2.05562600
H	5.56790800	-2.37122100	-0.02053600
H	5.90888000	-0.52059300	-1.63368700
C	-1.18825800	0.11353100	0.96898600
O	-1.75581500	0.29520000	2.26819200
C	-2.11717300	-0.72184500	0.07667200
C	-3.31786800	-1.21629600	0.57270400
C	-1.74314500	-1.03749900	-1.23158500
C	-4.14672600	-1.99382700	-0.23160300
H	-3.58888300	-1.00238200	1.59923600
C	-2.56840300	-1.81233700	-2.03326600
H	-0.79711800	-0.67451300	-1.62127300
C	-3.77758000	-2.29104800	-1.53583700
H	-5.08148600	-2.37258400	0.16949700
H	-2.26588300	-2.04740100	-3.04870100
H	-4.42274600	-2.89840900	-2.16233900
C	-0.86792700	1.49737600	0.39520800
C	0.17787100	2.23156200	0.96257400
C	-1.65542600	2.10198600	-0.58275800
C	0.44451200	3.52695300	0.54365700
H	0.78285300	1.77508900	1.73826300
C	-1.39007600	3.40309000	-1.00140600
H	-2.47775000	1.55471100	-1.02835900
C	-0.33909100	4.11737500	-0.44374500
H	1.26518100	4.07878900	0.99058000
H	-2.01091000	3.85590500	-1.76778900
H	-0.13079400	5.13022600	-0.77301900

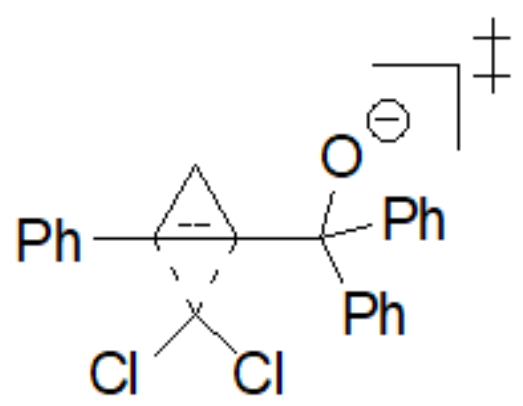

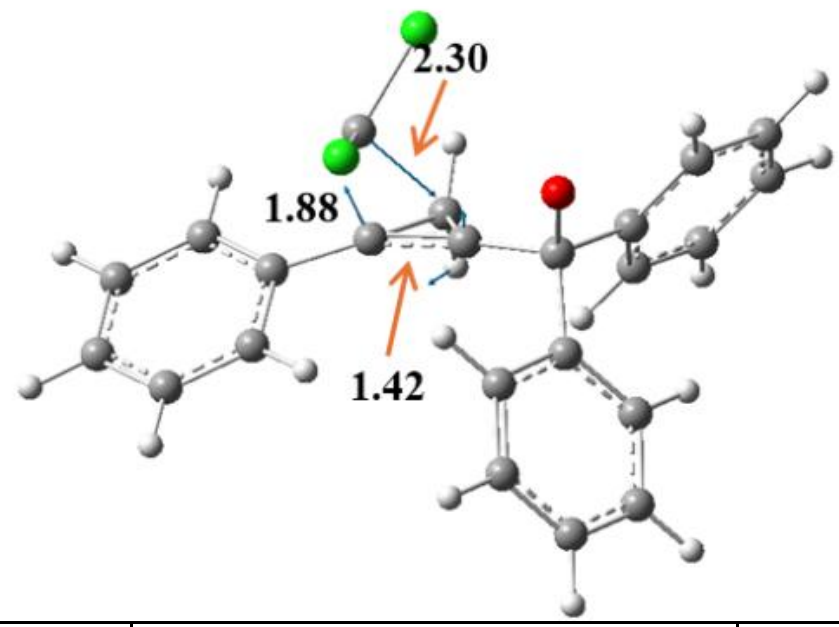

Total Electronic Energy	Sum of electronic and zero-point Energies	Sum of electronic and thermal Enthalpies	Sum of electronic and thermal Free Energies
-1881.79	-1881.456953	-1881.433477	-1881.512221

Cartesian Coordinates

C	0.29471500	1.55150800	1.04212900
C	-1.91169000	2.19905100	1.23036900
C	-0.82126400	0.82794700	0.53379600
C	0.09244300	1.56064400	-0.45106200
H	0.73820400	0.88967400	-1.05017400
H	-0.22959400	2.43701900	-1.01199500
Cl	-2.23062900	1.96978300	2.93020600
Cl	-1.83174800	3.84817000	0.67411100
C	-1.78768400	-0.31916900	0.31395800
C	-1.63387300	-1.27799800	1.34250100
C	-2.72230100	-0.52952500	-0.71487300
C	-2.40223600	-2.43597000	1.33552000
H	-0.90615100	-1.10500500	2.13150800
C	-3.49348600	-1.70065800	-0.71596500
H	-2.82750900	0.22649200	-1.49599500
C	-3.33479300	-2.65415200	0.30920500
H	-2.28344300	-3.18132900	2.12832800
H	-4.20694900	-1.86471900	-1.50927000
H	-3.92575500	-3.56824500	0.31127400
C	1.40351100	2.19082200	1.85244500
O	0.79490600	3.08308600	2.77315900
C	2.29427600	2.94395400	0.85679300
C	2.57854500	4.29336200	1.05408400
C	2.83410800	2.28838800	-0.25078300
C	3.40175300	4.97879300	0.15362500
H	2.13082400	4.80456700	1.89389000
C	3.65407400	2.96676300	-1.14750600
H	2.60262900	1.23689800	-0.42255900
C	3.94334900	4.31640900	-0.94788400
H	3.61404100	6.03240500	0.32057400
H	4.06686700	2.44564100	-2.01307000
H	4.57969200	4.85410900	-1.64032200
C	2.13272500	1.07746500	2.62314700
C	1.37870600	0.32556600	3.54327000
C	3.50016000	0.80764200	2.48168600
C	1.97895600	-0.68553500	4.27604100
H	0.31374900	0.54002200	3.64805100
C	4.09343300	-0.22798100	3.22444200
H	4.10322500	1.38197000	1.78847600
C	3.32904000	-0.96843000	4.11331500
H	1.37895200	-1.27320000	4.96997600
H	5.14678800	-0.42806000	3.10013700
H	3.80113800	-1.77433800	4.68298100

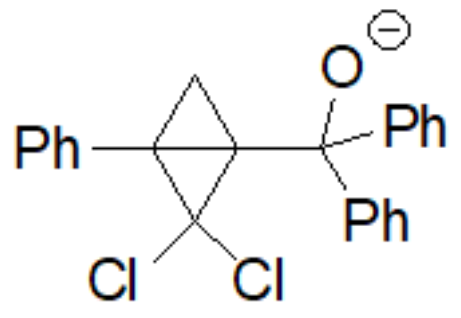

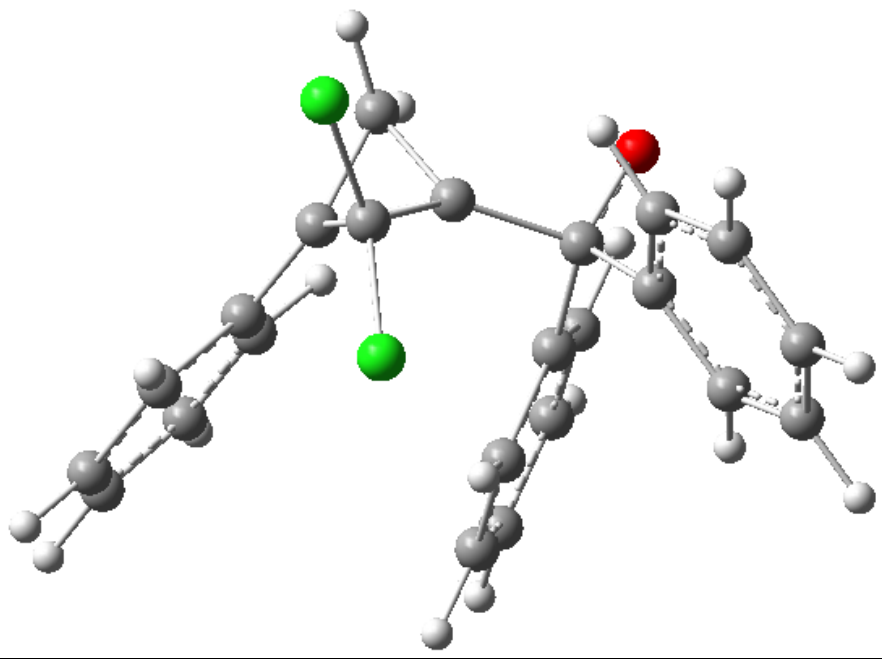

Total Electronic Energy	Sum of electronic and zero-point Energies	Sum of electronic and thermal Enthalpies	Sum of electronic and thermal Free Energies
-1881.858581	-1881.527127	-1881.504436	-1881.579074

Cartesian Coordinates

C	1.04314700	0.83333100	1.05517400
O	1.38502300	1.17540300	2.28933700
C	-0.11275600	1.70768800	0.47757500
C	-0.71823700	2.61075600	1.35056600
C	-0.59788800	1.61255800	-0.83257500
C	-1.79915000	3.39211700	0.93373800
H	-0.29694700	2.66267500	2.35171100
C	-1.66836700	2.39642000	-1.25674000
H	-0.13803700	0.91162800	-1.52499100
C	-2.27899600	3.28760200	-0.37131300
H	-2.26519400	4.08796100	1.62799700
H	-2.03891600	2.29969500	-2.27416800
H	-3.12133100	3.89204200	-0.69845800
C	2.28189800	0.84318000	0.12597400
C	3.24454800	-0.16066500	0.29216100
C	2.56817300	1.88685900	-0.75578600
C	4.43622000	-0.14344300	-0.42379900
H	3.04969600	-0.96044800	1.00329600
C	3.76790100	1.91589500	-1.47130800
H	1.84977800	2.69127500	-0.88468100
C	4.70460100	0.89753500	-1.31660600
H	5.15969900	-0.94305400	-0.28639400
H	3.96720300	2.73938000	-2.15297000
H	5.63447000	0.91425800	-1.87892400
C	-0.35954400	-1.17757400	2.18217800
H	-0.01871100	-2.04140100	2.75295200
H	-0.83728500	-0.39135900	2.76395100
C	0.46360000	-0.64899800	1.06973300
C	-0.85268800	-1.40015400	0.78321300
C	-2.15375500	-1.11507700	0.15773200
C	-2.58505400	-1.84124000	-0.95842800
C	-2.99332600	-0.12376600	0.68384600
C	-3.81835700	-1.56741500	-1.54783500
H	-1.94704200	-2.61986100	-1.36447400
C	-4.22049800	0.14973000	0.09173200
H	-2.66073000	0.46437000	1.53446500
C	-4.64043100	-0.56930500	-1.02855900
H	-4.13547700	-2.13819600	-2.41617100
H	-4.84199500	0.94277500	0.49749000
H	-5.59798700	-0.35119200	-1.49254100
C	0.42509600	-1.85053200	0.22846800
Cl	1.18009500	-3.44895800	0.76382200
Cl	0.72236300	-1.67908800	-1.51015300

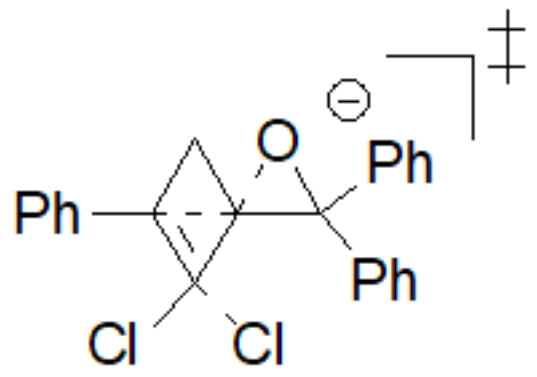	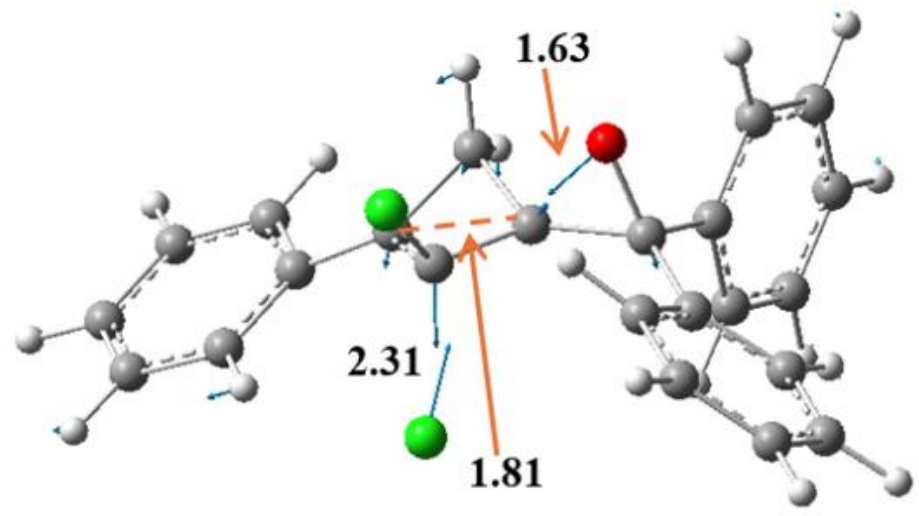		
Total Electronic Energy	Sum of electronic and zero-point Energies	Sum of electronic and thermal Enthalpies	Sum of electronic and thermal Free Energies
-1881.87	-1881.568769	-1881.546528	-1881.620667

Cartesian Coordinates

C	1.45494400	0.22565300	0.50578500
O	1.05362400	-0.00015000	1.79257800
C	1.90468400	1.64097500	0.20939700
C	2.24916800	2.50196300	1.25436200
C	1.94662700	2.10220800	-1.10935700
C	2.65027100	3.80585900	0.97720700
H	2.17878000	2.14345400	2.27740600
C	2.34651100	3.40732900	-1.38352200
H	1.65793200	1.43870000	-1.91825400
C	2.70353200	4.25876500	-0.33654200
H	2.91741200	4.47247900	1.79654600
H	2.37579500	3.75764100	-2.40879800
H	3.01552900	5.27715600	-0.55106100
C	2.32750700	-0.89257900	-0.03193900
C	2.03908200	-2.19338400	0.39681000
C	3.45073600	-0.66524500	-0.83605300
C	2.82177000	-3.26043800	-0.02033500
H	1.22219900	-2.34790500	1.09625400
C	4.22894000	-1.74500300	-1.25676900
H	3.71763300	0.33721500	-1.14203100
C	3.91610800	-3.03907900	-0.86473500
H	2.58905400	-4.26745600	0.31719600
H	5.09323200	-1.55929600	-1.88788800
H	4.52450800	-3.87238600	-1.19428600
C	-1.01866500	1.17225000	1.15161100
H	-1.05981300	1.13473600	2.24525600
H	-1.00580500	2.19778100	0.77015700
C	-0.01813500	0.17586600	0.57660900
C	-1.81081300	0.14542300	0.39278800
C	-3.15217700	0.34409600	-0.27418700
C	-3.80957400	-0.71304600	-0.95753800
C	-3.81856600	1.59177200	-0.10847900
C	-5.10532500	-0.53211500	-1.42226300
H	-3.29795000	-1.65962000	-1.10068800
C	-5.10919100	1.75943100	-0.58712900
H	-3.32868100	2.40135400	0.42329900
C	-5.75402700	0.69667000	-1.24194100
H	-5.61557900	-1.34367800	-1.93201700
H	-5.62620200	2.70564700	-0.44653600
H	-6.77180500	0.83174600	-1.60498600
C	-0.96058400	-0.99307600	0.44160500
Cl	-1.16395700	-2.28775200	1.62810600
Cl	-0.86788800	-2.00417000	-1.48520100

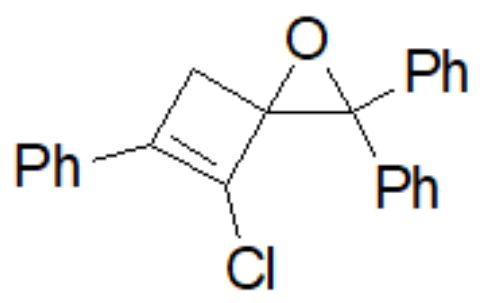

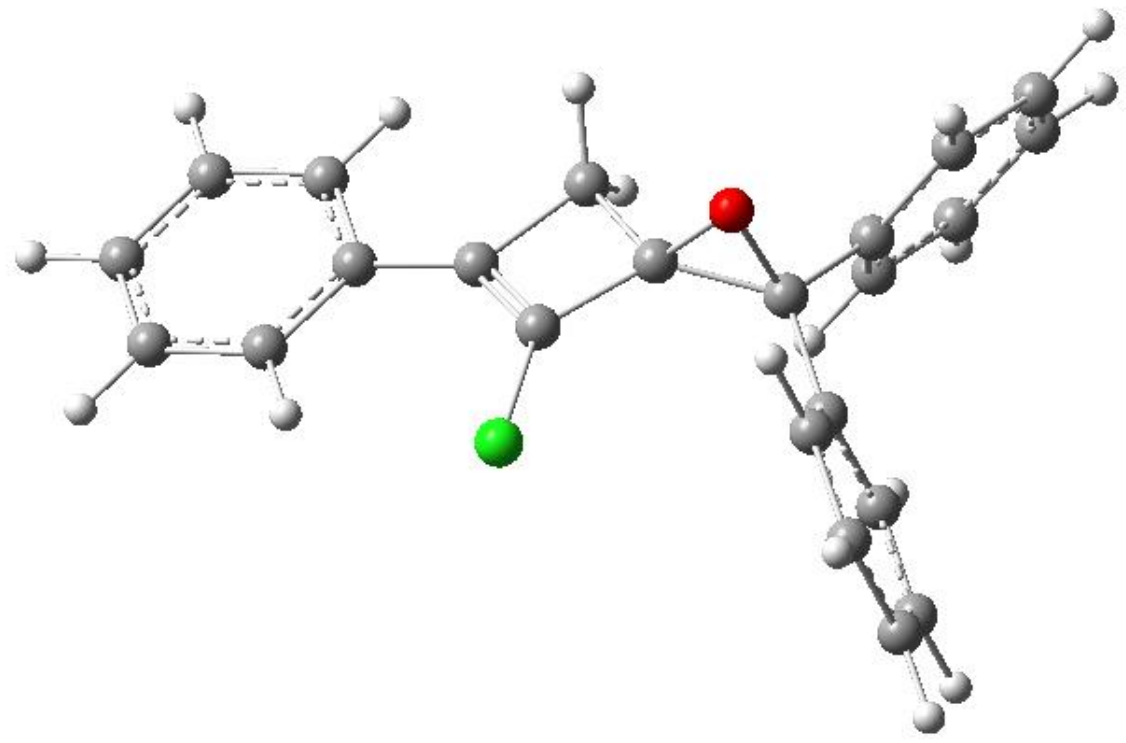

Total Electronic Energy	Sum of electronic and zero-point Energies	Sum of electronic and thermal Enthalpies	Sum of electronic and thermal Free Energies
-1421.515239	-1421.180198	-1421.159243	-1421.231738

Cartesian Coordinates

C	-1.45784800	0.12944600	-0.45809300
O	-0.95090400	0.04058500	-1.80253500
C	-2.12187100	1.44036200	-0.14078600
C	-2.96206100	2.02787800	-1.08360700
C	-1.92050600	2.07571400	1.08362400
C	-3.60227800	3.22754500	-0.80108300
H	-3.09775000	1.54140100	-2.04349900
C	-2.56307400	3.27564000	1.36741500
H	-1.25049000	1.63501600	1.81629300
C	-3.40716900	3.85246900	0.42609700
H	-4.25449500	3.67789100	-1.54240900
H	-2.39820400	3.76283300	2.32300900
H	-3.90727600	4.79011400	0.64564700
C	-2.14872500	-1.11901200	0.02588400
C	-2.28382000	-2.21009100	-0.83054900
C	-2.67795900	-1.19864100	1.31394400
C	-2.92756300	-3.36440100	-0.40254600
H	-1.87832300	-2.14099300	-1.83251300
C	-3.31374600	-2.35646800	1.74273900
H	-2.60518600	-0.35012300	1.98473000
C	-3.44114200	-3.44369900	0.88595600
H	-3.02833300	-4.20498900	-1.08161700
H	-3.71685300	-2.40555900	2.74916100
H	-3.94257500	-4.34613700	1.22034400
C	1.01644600	1.28289200	-0.64002000
H	1.16758000	1.81019800	-1.58556000
H	0.83437400	2.00351400	0.16154600
C	-0.01130500	0.14186600	-0.75621800
C	2.03314400	0.18456200	-0.34013400
C	3.44550200	0.28710100	-0.00967000
C	4.22643700	-0.84073300	0.27622500
C	4.04717700	1.54997600	0.02432100
C	5.57065200	-0.70077800	0.58627900
H	3.77476400	-1.82517400	0.25441200
C	5.39251500	1.68580100	0.33579900
H	3.45176800	2.42980400	-0.19768100
C	6.15822700	0.56039300	0.61759900
H	6.16483500	-1.58183800	0.80562900
H	5.84441400	2.67203300	0.35773000
H	7.21056500	0.66464400	0.86077400
C	1.11921500	-0.79123100	-0.48836300
Cl	1.18923100	-2.50805100	-0.40497700

6c(ii) TS1

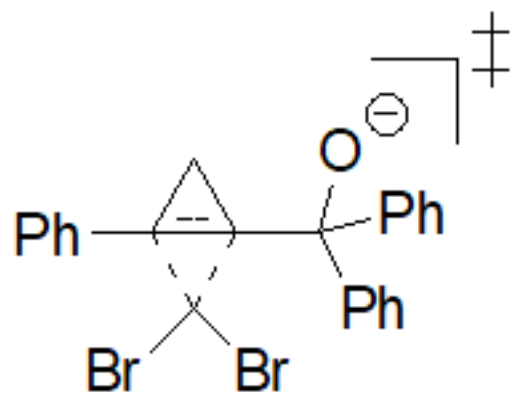

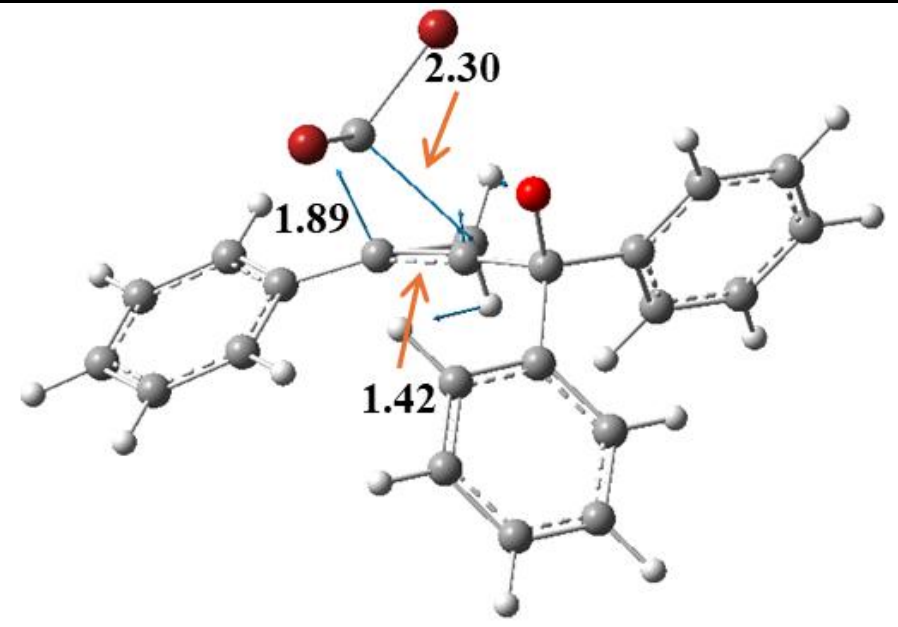

Total Electronic Energy	Sum of electronic and zero-point Energies	Sum of electronic and thermal Enthalpies	Sum of electronic and thermal Free Energies
-6105.05	-6104.729251	-6104.705208	-6104.786352

Cartesian Coordinates

C	0.26472800	0.03054800	0.39490800
C	-1.41451300	-1.33065400	-0.41147200
C	-1.05188800	-0.05659200	0.92996000
C	0.10496300	-0.67966600	1.71425900
H	0.38538200	-0.11674200	2.62564500
H	0.21901300	-1.75567100	1.83913300
C	-2.42924800	0.38099300	1.38774900
C	-2.73582900	1.64789800	0.83832000
C	-3.34676800	-0.26249400	2.23647200
C	-3.94369500	2.26461400	1.14171800
H	-2.01713400	2.13647700	0.18501600
C	-4.56315400	0.36615500	2.53893900
H	-3.09102700	-1.24238200	2.64537000
C	-4.86293500	1.62936300	1.99145000
H	-4.18263700	3.24634300	0.72061100
H	-5.26504700	-0.12553100	3.19534500
H	-5.80259600	2.12579300	2.22682300
C	1.53357700	0.30814200	-0.38466100
O	1.38253000	-0.28534800	-1.66497500
C	2.67928800	-0.34547200	0.39772500
C	3.53932200	-1.23906400	-0.23668000
C	2.87778100	-0.04970900	1.74718200
C	4.59315000	-1.82637500	0.47242300
H	3.36091900	-1.49068800	-1.27201400
C	3.92549100	-0.63109900	2.45495100
H	2.19892500	0.63498700	2.25616900
C	4.79007400	-1.52184300	1.81922700
H	5.25588800	-2.52274600	-0.03638100
H	4.06853700	-0.39659700	3.51122500
H	5.60819100	-1.98070300	2.36112800
C	1.67371500	1.83187900	-0.53921200
C	0.65144300	2.50987100	-1.22900200
C	2.77401200	2.55868300	-0.06614200
C	0.72302000	3.88158300	-1.41028300
H	-0.20346000	1.93810500	-1.59414300
C	2.82819200	3.95150800	-0.24856500
H	3.57895500	2.05603400	0.45682100
C	1.80217400	4.60297000	-0.91605300
H	-0.08521400	4.39660700	-1.92836600
H	3.67944200	4.49872400	0.12689300
H	1.85372900	5.68771200	-1.04857200
Br	-0.51420600	-3.01177400	-0.51815400
Br	-1.86006500	-0.50063700	-2.07299400

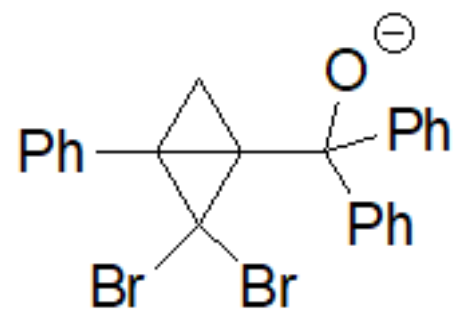

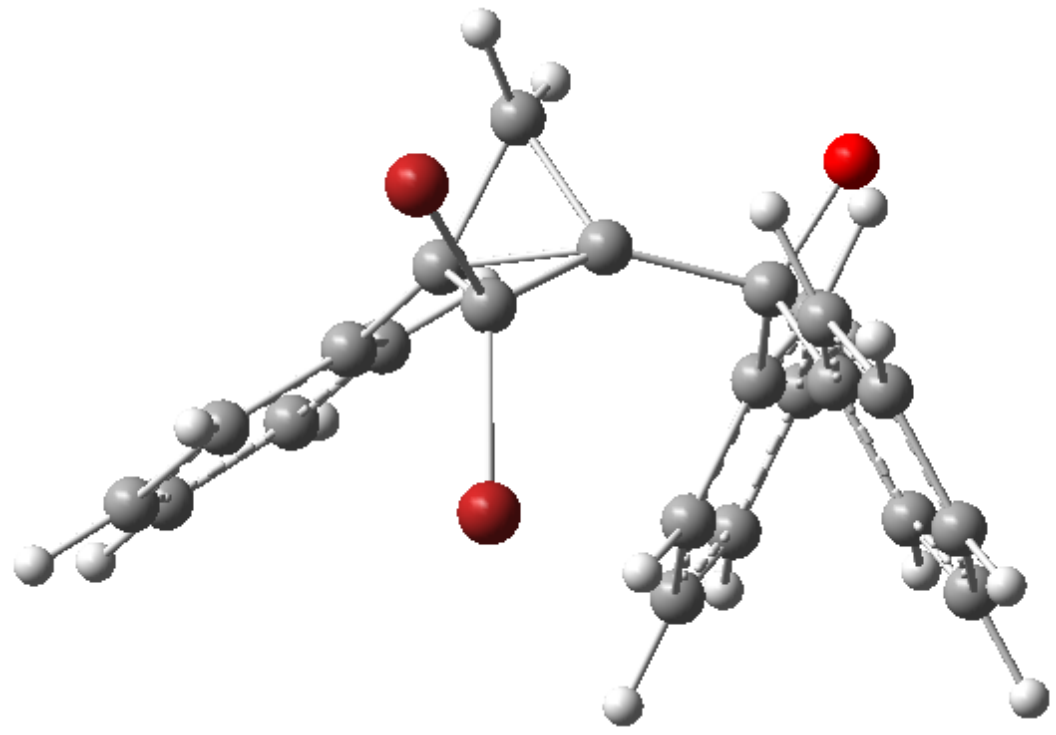

Total Electronic Energy	Sum of electronic and zero-point Energies	Sum of electronic and thermal Enthalpies	Sum of electronic and thermal Free Energies
-6105.233395	-6104.902363	-6104.879141	-6104.956122

Cartesian Coordinates

C	0.52110200	1.47935700	1.12837900
O	0.83298600	1.91307700	2.37026300
C	-0.80202000	2.08733300	0.59395600
C	-1.57783000	2.85258800	1.46485900
C	-1.28807800	1.85711900	-0.70023300
C	-2.80928800	3.37739800	1.06094400
H	-1.18957100	3.01855100	2.46443900
C	-2.50374100	2.39360500	-1.11601100
H	-0.71103700	1.25182800	-1.39286800
C	-3.27550400	3.15308000	-0.23328600
H	-3.40165900	3.96438600	1.75753900
H	-2.85882300	2.20660300	-2.12573100
H	-4.22923600	3.56283500	-0.55265700
C	1.71875100	1.72815300	0.17883600
C	2.90217000	1.02208700	0.44546300
C	1.73208800	2.66399000	-0.85511100
C	4.05107800	1.22459500	-0.31052200
H	2.91690700	0.30875900	1.26680800
C	2.88588300	2.87597500	-1.61877200
H	0.83809800	3.23638500	-1.08218100
C	4.04660000	2.15375300	-1.35658000
H	4.95198700	0.65956000	-0.08774300
H	2.87076100	3.60848500	-2.42095500
H	4.93958700	2.31269200	-1.95347300
C	-0.37918200	-0.82423800	2.25675400
H	0.18457600	-1.55914800	2.83388400
H	-1.11229400	-0.24873900	2.81955300
C	0.29582800	-0.07055600	1.16584300
C	-0.74854100	-1.16303200	0.84861100
C	-2.05937800	-1.33651100	0.20553400
C	-2.21945500	-2.35304500	-0.74168100
C	-3.15746500	-0.54667100	0.56517000
C	-3.45790300	-2.55867400	-1.34636200
H	-1.37168200	-2.98186300	-0.99835600
C	-4.39428100	-0.75900300	-0.03704900
H	-3.03447900	0.25414000	1.29092300
C	-4.54820300	-1.76222400	-0.99693800
H	-3.57252200	-3.34539900	-2.08557400
H	-5.23856800	-0.13327900	0.23614900
H	-5.51327600	-1.92227700	-1.46722900
C	0.60327000	-1.21665300	0.31330600
Br	0.84763600	-0.98113300	-1.57517900
Br	1.88527700	-2.65225800	0.91931300

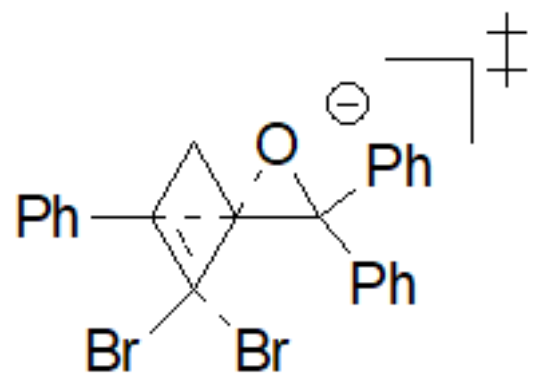

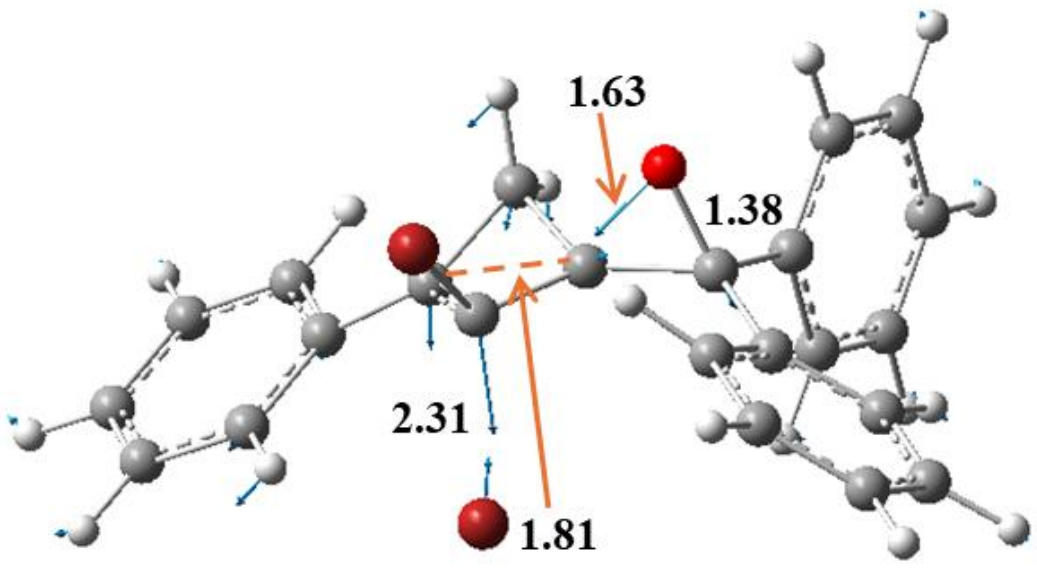

Total Electronic Energy	Sum of electronic and zero-point Energies	Sum of electronic and thermal Enthalpies	Sum of electronic and thermal Free Energies
-6105.165930	-6104.837924	-6104.814985	-6104.892884

Cartesian Coordinates

C	1.69364900	0.40078300	0.50986600
O	1.31824300	0.20460800	1.80927600
C	2.28514700	1.76172800	0.20786100
C	2.76114900	2.56681400	1.24573400
C	2.32835100	2.23172700	-1.10775300
C	3.29278700	3.82211600	0.96391700
H	2.68996300	2.20566300	2.26779300
C	2.85902400	3.48834400	-1.38652700
H	1.93832600	1.61389300	-1.91028900
C	3.34650100	4.28253500	-0.34719900
H	3.66213600	4.44528100	1.77774100
H	2.88839900	3.84588700	-2.40930100
H	3.76053200	5.26308900	-0.56531200
C	2.41704900	-0.79977100	-0.07006400
C	2.00332700	-2.06622900	0.35892100
C	3.52766100	-0.68723000	-0.91492900
C	2.64760200	-3.20753500	-0.09665100
H	1.20109200	-2.13873900	1.08800100
C	4.16595000	-1.84062600	-1.37400000
H	3.89172700	0.28356300	-1.22282400
C	3.72735600	-3.09726600	-0.98070900
H	2.31816100	-4.18699600	0.24142100
H	5.02125900	-1.74316200	-2.03637900
H	4.22754100	-3.98820200	-1.34015700
C	-0.63479800	1.60514000	1.25802100
H	-0.63895200	1.55894400	2.35210000
H	-0.52332300	2.62767000	0.88466900
C	0.22772800	0.51210100	0.63690600
C	-1.56300400	0.68084800	0.52194900
C	-2.89829600	1.03370800	-0.09129600
C	-3.69318500	0.06357100	-0.75738400
C	-3.41655800	2.34485400	0.11002100
C	-4.97757300	0.39130900	-1.17043400
H	-3.29463300	-0.93160600	-0.92776900
C	-4.69783700	2.65901000	-0.31724800
H	-2.82097100	3.08919100	0.62909400
C	-5.47980400	1.68155900	-0.95534800
H	-5.59278700	-0.35303100	-1.66673300
H	-5.10187500	3.65445400	-0.14921400
H	-6.48935800	1.93196700	-1.27786200
C	-0.84211300	-0.54460000	0.52880200
Br	-1.16652600	-1.92643700	1.80684000
Br	-0.89529900	-1.57390800	-1.54523800

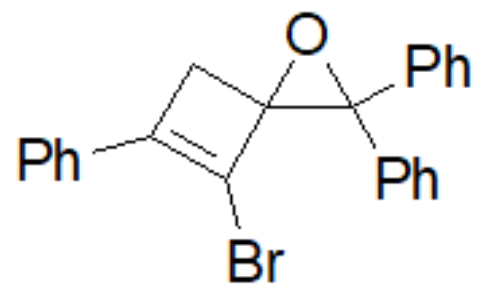

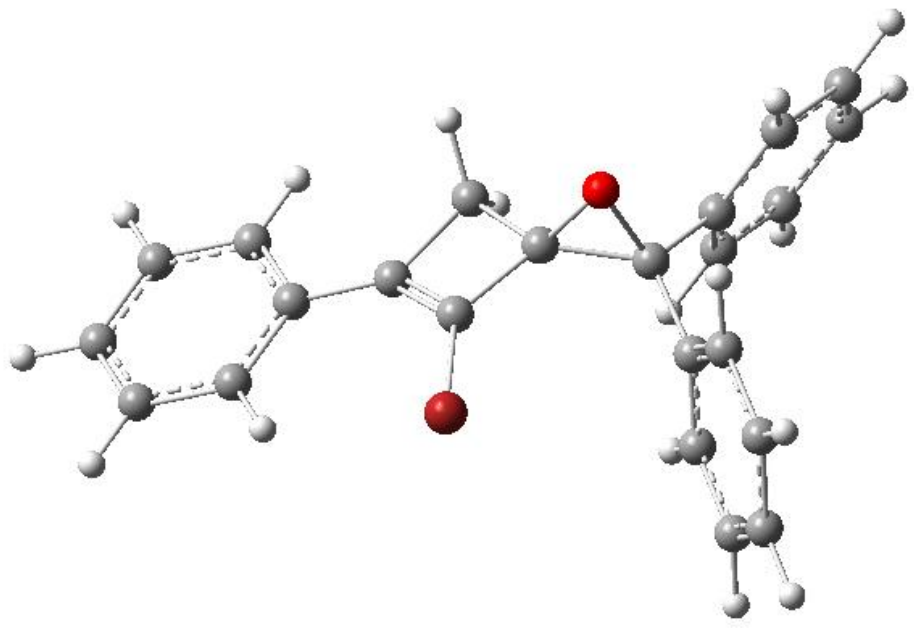

Total Electronic Energy	Sum of electronic and zero-point Energies	Sum of electronic and thermal Enthalpies	Sum of electronic and thermal Free Energies
-3533.306509	-3533.0826601	-3533.11250679	-3533.1236781

Cartesian Coordinates

C	0.04215200	-0.69891100	1.18860200
C	1.26803800	-1.03548600	0.92691500
C	0.52977500	-1.93300700	1.88149600
H	0.18874900	-2.91549300	1.54236700
H	0.74785700	-1.89334200	2.95101500
C	2.52337800	-0.87842600	0.22499100
C	2.72367900	0.16453700	-0.68820400
C	3.55935200	-1.78738500	0.45748400
C	3.93582900	0.28947800	-1.35009900
H	1.92443300	0.87559800	-0.86743300
C	4.77070200	-1.65924500	-0.20823800
H	3.40000800	-2.59373200	1.16571000
C	4.96200200	-0.62111700	-1.11312100
H	4.08251300	1.10100900	-2.05562600
H	5.56790800	-2.37122100	-0.02053600
H	5.90888000	-0.52059300	-1.63368700
C	-1.18825800	0.11353100	0.96898600
O	-1.75581500	0.29520000	2.26819200
C	-2.11717300	-0.72184500	0.07667200
C	-3.31786800	-1.21629600	0.57270400
C	-1.74314500	-1.03749900	-1.23158500
C	-4.14672600	-1.99382700	-0.23160300
H	-3.58888300	-1.00238200	1.59923600
C	-2.56840300	-1.81233700	-2.03326600
H	-0.79711800	-0.67451300	-1.62127300
C	-3.77758000	-2.29104800	-1.53583700
H	-5.08148600	-2.37258400	0.16949700
H	-2.26588300	-2.04740100	-3.04870100
H	-4.42274600	-2.89840900	-2.16233900
C	-0.86792700	1.49737600	0.39520800
C	0.17787100	2.23156200	0.96257400
C	-1.65542600	2.10198600	-0.58275800
C	0.44451200	3.52695300	0.54365700
H	0.78285300	1.77508900	1.73826300
C	-1.39007600	3.40309000	-1.00140600
H	-2.47775000	1.55471100	-1.02835900
C	-0.33909100	4.11737500	-0.44374500
H	1.26518100	4.07878900	0.99058000
H	-2.01091000	3.85590500	-1.76778900
H	-0.13079400	5.13022600	-0.77301900

6c(iii) TS1

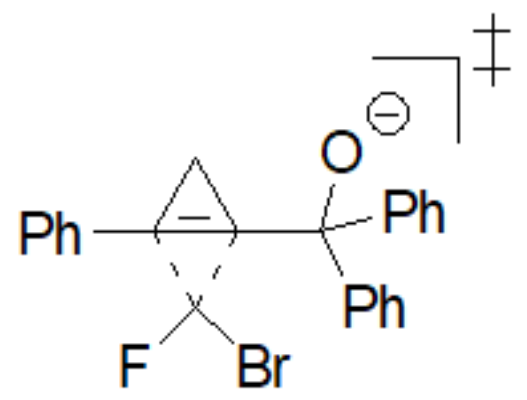

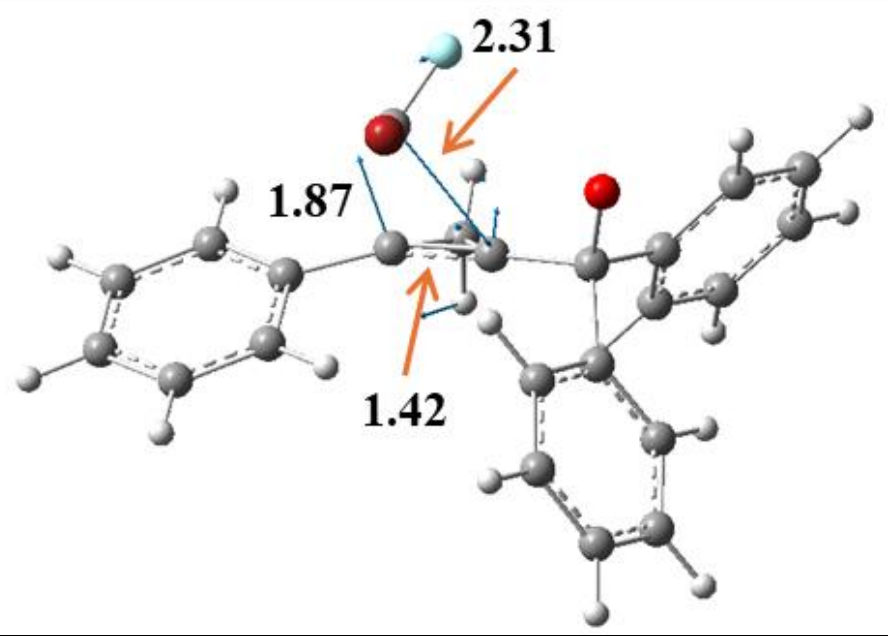

Total Electronic Energy	Sum of electronic and zero-point Energies	Sum of electronic and thermal Enthalpies	Sum of electronic and thermal Free Energies
-3633.049496	-3632.723618	-3632.700279	-3632.777403

Cartesian Coordinates

C	-0.23639500	-0.40215300	-0.26820800
C	1.35347400	-1.67165300	0.82401700
C	1.08011900	-0.67173800	-0.74265800
C	-0.10724800	-1.35863400	-1.42343000
H	-0.33609000	-0.96815000	-2.43067900
H	-0.29248200	-2.43362100	-1.33938000
C	2.48718300	-0.41119100	-1.24927000
C	2.85886800	0.92300000	-0.95473800
C	3.36960300	-1.26191800	-1.93136700
C	4.10635400	1.39497600	-1.35027800
H	2.16681300	1.57079700	-0.42851900
C	4.62631700	-0.77368400	-2.32322200
H	3.06460300	-2.28952900	-2.14215700
C	4.99596700	0.55193400	-2.03503200
H	4.39963900	2.42779900	-1.12818300
H	5.30637900	-1.42928200	-2.85220200
H	5.96351000	0.93549900	-2.34334400
C	-1.48547200	0.08582600	0.43634100
O	-1.35384700	-0.23600600	1.80916600
C	-2.66884500	-0.65697500	-0.19665000
C	-3.57621500	-1.33558700	0.61593600
C	-2.85499800	-0.66774000	-1.57678900
C	-4.66935700	-2.00366800	0.05231700
H	-3.40645500	-1.35603200	1.68411700
C	-3.94168700	-1.32998600	-2.14523300
H	-2.14601000	-0.15696000	-2.22625700
C	-4.85338700	-2.00029100	-1.33190400
H	-5.36791400	-2.52661300	0.69927300
H	-4.07705000	-1.33257400	-3.22978600
H	-5.69953100	-2.52615900	-1.75998900
C	-1.53839100	1.61388200	0.26432000
C	-0.52176600	2.36136700	0.89449300
C	-2.53799500	2.28773700	-0.45638300
C	-0.49507100	3.74262000	0.77627200
H	0.25218500	1.82542700	1.45350600
C	-2.48771100	3.68894300	-0.58257700
H	-3.33821500	1.73889400	-0.94121100
C	-1.46878300	4.40567500	0.03134200
H	0.30921900	4.30734800	1.25392700
H	-3.25941400	4.19379300	-1.15664600
H	-1.44345400	5.49048700	-0.07487500
Br	1.83917200	-0.56061800	2.29867000
F	0.63962700	-2.78065500	1.11909300

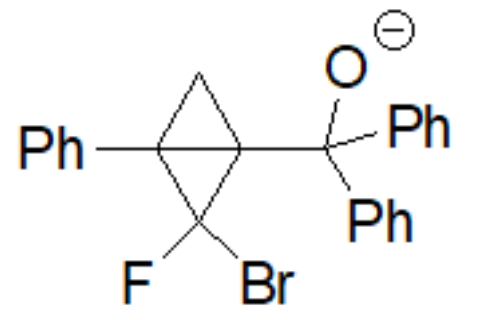

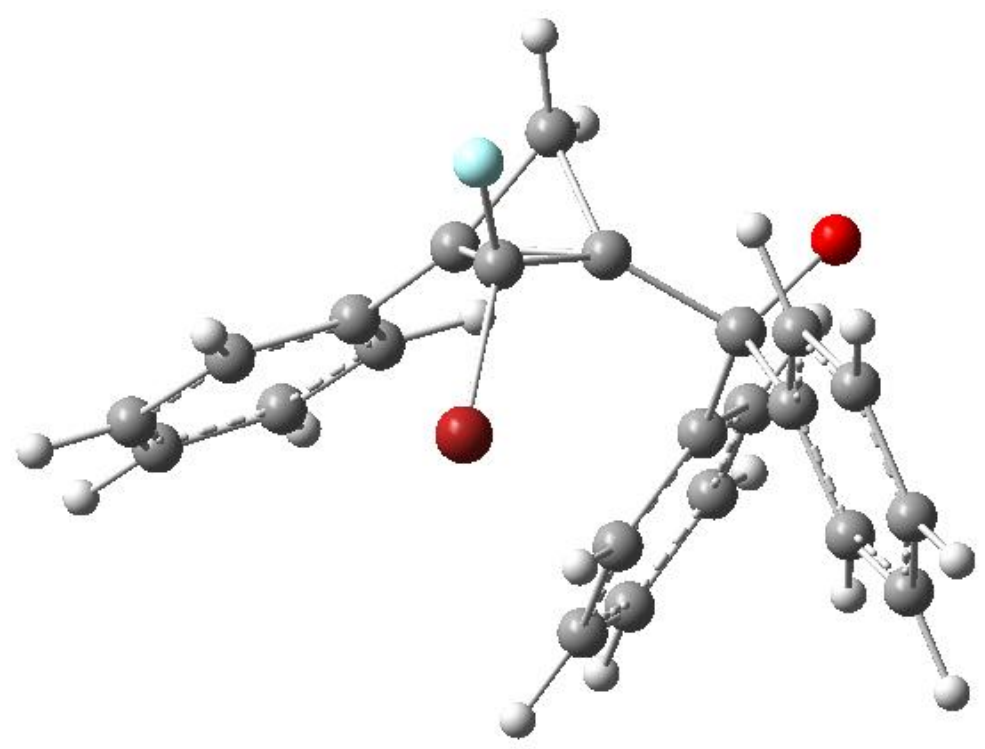

Total Electronic Energy	Sum of electronic and zero-point Energies	Sum of electronic and thermal Enthalpies	Sum of electronic and thermal Free Energies
-3633.230064	-3632.896889	-3632.874498	-3632.948686

Cartesian Coordinates

C	1.03177400	1.04207900	1.06854100
O	1.41021100	1.63296300	2.22674100
C	-0.01181700	1.87903600	0.28316900
C	-0.59071400	2.98234900	0.91017000
C	-0.43988500	1.54796500	-1.00964800
C	-1.57732100	3.73902600	0.26979800
H	-0.24914800	3.22667100	1.91106500
C	-1.40768100	2.30834400	-1.66061700
H	-0.00784400	0.68913700	-1.51556900
C	-1.98754700	3.40651000	-1.01987700
H	-2.02177300	4.59018100	0.77858800
H	-1.72127600	2.03738800	-2.66512000
H	-2.74973200	3.99418500	-1.52316700
C	2.29485100	0.74762400	0.22066400
C	3.15923500	-0.27299100	0.64451000
C	2.70103900	1.54380600	-0.85203300
C	4.37942800	-0.49752600	0.01580200
H	2.86422300	-0.90356700	1.48025600
C	3.92532000	1.32272300	-1.49196100
H	2.05900100	2.34642400	-1.20262400
C	4.76911000	0.30072700	-1.06422600
H	5.02613700	-1.29902900	0.36178000
H	4.21599400	1.95431300	-2.32679500
H	5.71854900	0.12686700	-1.56147400
C	-0.55512300	-0.60415100	2.54661600
H	-0.27798800	-1.33822500	3.30142600
H	-1.07138700	0.27518700	2.92905000
C	0.33935900	-0.32615100	1.38499800
C	-0.98991800	-1.07342100	1.18918900
C	-2.26965500	-0.92257200	0.47163600
C	-2.75644500	-1.98390400	-0.29984500
C	-3.03863300	0.24112200	0.59587600
C	-3.98810800	-1.88083200	-0.94609900
H	-2.17427400	-2.89744400	-0.37941900
C	-4.26681300	0.34264400	-0.05098200
H	-2.66175900	1.07762000	1.17911800
C	-4.74574600	-0.71643000	-0.82576100
H	-4.35535200	-2.71221000	-1.53984900
H	-4.84841700	1.25477500	0.04378500
H	-5.70436300	-0.63456700	-1.32842900
C	0.28645500	-1.67446600	0.81789300
Br	0.68382300	-1.95936900	-1.04159100
F	0.79743800	-2.77584100	1.50136200

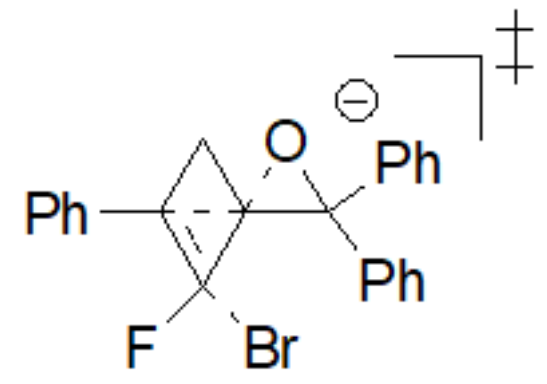

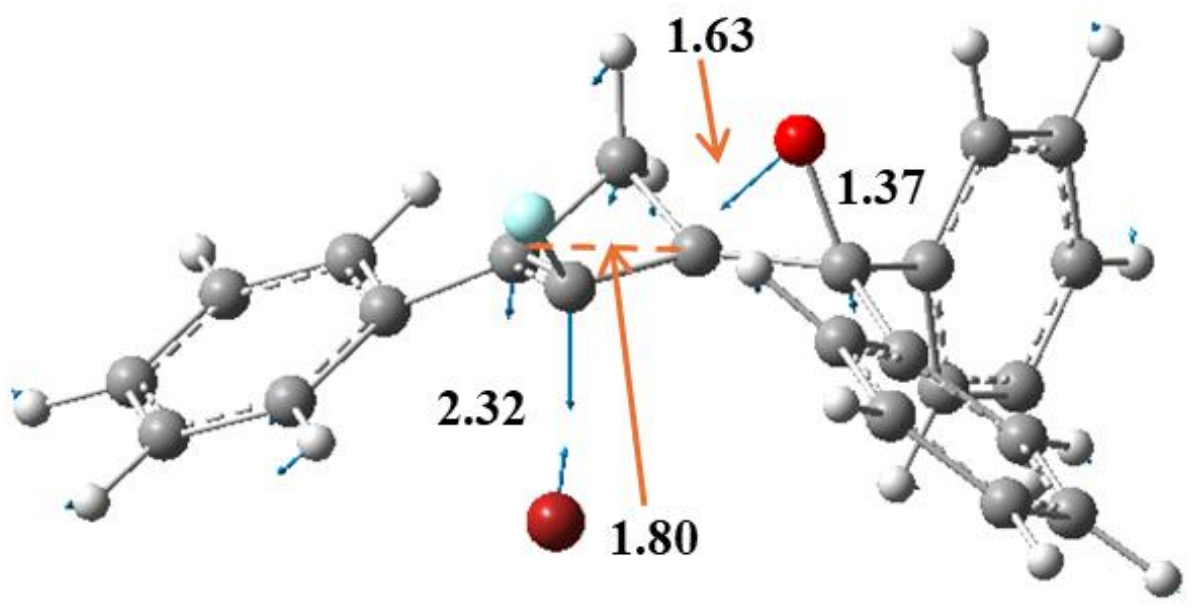

Total Electronic Energy	Sum of electronic and zero-point Energies	Sum of electronic and thermal Enthalpies	Sum of electronic and thermal Free Energies
-3633.188763	-3632.928510	-3632.906355	-3632.981458

Cartesian Coordinates

C	1.45494400	0.22565300	0.50578500
O	1.05362400	-0.00015000	1.79257800
C	1.90468400	1.64097500	0.20939700
C	2.24916800	2.50196300	1.25436200
C	1.94662700	2.10220800	-1.10935700
C	2.65027100	3.80585900	0.97720700
H	2.17878000	2.14345400	2.27740600
C	2.34651100	3.40732900	-1.38352200
H	1.65793200	1.43870000	-1.91825400
C	2.70353200	4.25876500	-0.33654200
H	2.91741200	4.47247900	1.79654600
H	2.37579500	3.75764100	-2.40879800
H	3.01552900	5.27715600	-0.55106100
C	2.32750700	-0.89257900	-0.03193900
C	2.03908200	-2.19338400	0.39681000
C	3.45073600	-0.66524500	-0.83605300
C	2.82177000	-3.26043800	-0.02033500
H	1.22219900	-2.34790500	1.09625400
C	4.22894000	-1.74500300	-1.25676900
H	3.71763300	0.33721500	-1.14203100
C	3.91610800	-3.03907900	-0.86473500
H	2.58905400	-4.26745600	0.31719600
H	5.09323200	-1.55929600	-1.88788800
H	4.52450800	-3.87238600	-1.19428600
C	-1.01866500	1.17225000	1.15161100
H	-1.05981300	1.13473600	2.24525600
H	-1.00580500	2.19778100	0.77015700
C	-0.01813500	0.17586600	0.57660900
C	-1.81081300	0.14542300	0.39278800
C	-3.15217700	0.34409600	-0.27418700
C	-3.80957400	-0.71304600	-0.95753800
C	-3.81856600	1.59177200	-0.10847900
C	-5.10532500	-0.53211500	-1.42226300
H	-3.29795000	-1.65962000	-1.10068800
C	-5.10919100	1.75943100	-0.58712900
H	-3.32868100	2.40135400	0.42329900
C	-5.75402700	0.69667000	-1.24194100
H	-5.61557900	-1.34367800	-1.93201700
H	-5.62620200	2.70564700	-0.44653600
H	-6.77180500	0.83174600	-1.60498600
C	-0.96058400	-0.99307600	0.44160500
Br	-0.82064886	-2.03873504	-1.62018121
F	-1.11588671	-1.98173573	1.34765853

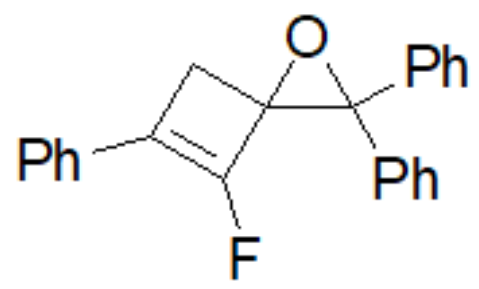

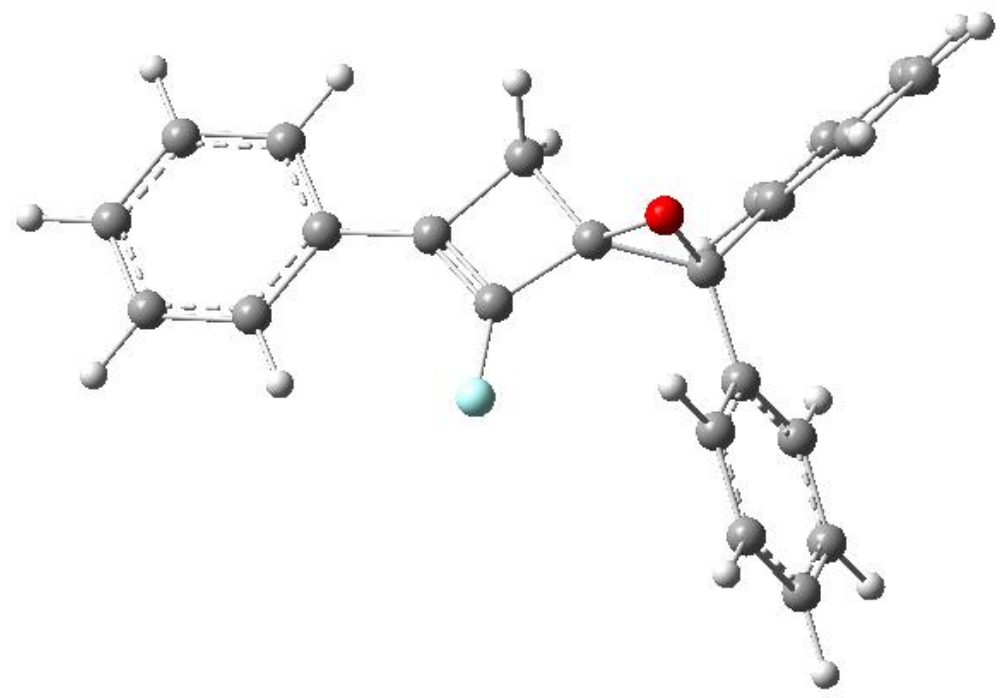

Total Electronic Energy	Sum of electronic and zero-point Energies	Sum of electronic and thermal Enthalpies	Sum of electronic and thermal Free Energies
-1061.301575	-1060.967000	-1060.946162	-1061.018146

Cartesian Coordinates

C	-1.42631900	0.04030700	-0.51478400
O	-0.92629600	-0.00787000	-1.86119800
C	-2.11848900	1.32122700	-0.15144900
C	-3.04678400	1.87821700	-1.03335200
C	-1.86077100	1.94779700	1.07035700
C	-3.71467800	3.05438400	-0.69485700
H	-3.23649300	1.39158700	-1.98596700
C	-2.53146300	3.12224100	1.41030900
H	-1.13256400	1.51790700	1.75430200
C	-3.45939000	3.67649300	0.52794000
H	-4.43276000	3.48526600	-1.38539000
H	-2.32550900	3.60567000	2.35993000
H	-3.97896500	4.59247700	0.79068600
C	-2.04687800	-1.24597300	-0.03785600
C	-1.90850700	-2.41058300	-0.80033300
C	-2.75701800	-1.29851600	1.16607200
C	-2.46333800	-3.61052800	-0.35999000
H	-1.36845700	-2.37028600	-1.74047900
C	-3.30517200	-2.50214900	1.60811000
H	-2.88810600	-0.40040100	1.76136000
C	-3.16041700	-3.66174400	0.84741200
H	-2.35241600	-4.50622800	-0.96316600
H	-3.85130300	-2.52955700	2.54572600
H	-3.59187200	-4.59690200	1.18992500
C	1.05255700	1.22417000	-0.67993500
H	1.21262200	1.76857400	-1.61423900
H	0.86933400	1.91910300	0.14382100
C	0.02078700	0.08690900	-0.81948600
C	2.07072000	0.11611700	-0.39337200
C	3.48076800	0.17604900	-0.03841100
C	4.21037400	-0.99496300	0.22497300
C	4.12419900	1.41836500	0.04245400
C	5.55745700	-0.91803100	0.56241000
H	3.71724500	-1.96060400	0.16426100
C	5.47365800	1.49086600	0.38181400
H	3.56331200	2.32609300	-0.16240800
C	6.19257000	0.32425800	0.64221000
H	6.11489800	-1.82701900	0.76475400
H	5.96340600	2.45747800	0.44236600
H	7.24377100	0.38080400	0.90615600
C	1.14682700	-0.84015500	-0.56418000
F	1.18889600	-2.17328400	-0.49537500

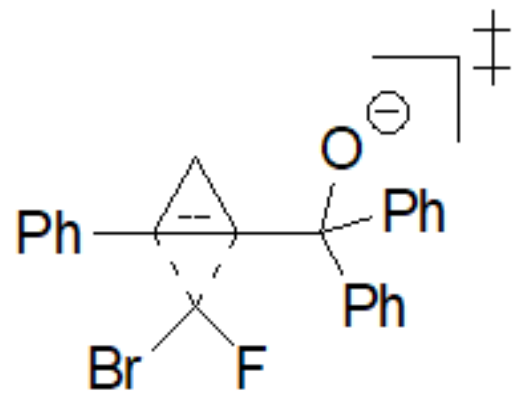

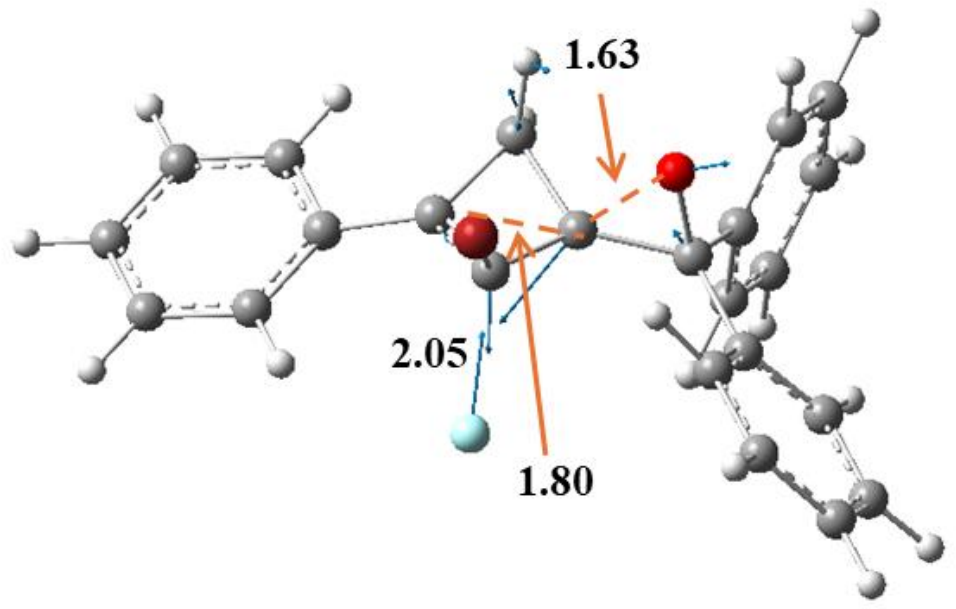

Total Electronic Energy	Sum of electronic and zero-point Energies	Sum of electronic and thermal Enthalpies	Sum of electronic and thermal Free Energies
-3633.185742	-3632.876888	-3632.854283	-3632.970908

Cartesian Coordinates

C	-1.55659400	0.24460200	-0.38053600
O	-1.15660800	-0.12166800	-1.63502400
C	-2.07805000	1.66102200	-0.25770000
C	-2.47820900	2.36355600	-1.39710400
C	-2.13139700	2.28307800	0.99282300
C	-2.94536000	3.66966500	-1.28009000
H	-2.39899200	1.88224300	-2.36775500
C	-2.59742700	3.59008300	1.10669600
H	-1.79979600	1.74333400	1.87408300
C	-3.00961500	4.28225400	-0.03329300
H	-3.25570800	4.21227700	-2.17245700
H	-2.63514100	4.06567800	2.07991400
H	-3.37322000	5.30219200	0.05614400
C	-2.36304000	-0.83982700	0.30838600
C	-2.01006900	-2.16791600	0.04248800
C	-3.48874500	-0.57048500	1.09589200
C	-2.73069100	-3.21231800	0.60386400
H	-1.19309800	-2.36763600	-0.64531000
C	-4.20417800	-1.62678700	1.66233800
H	-3.80556100	0.44760100	1.27730700
C	-3.82683000	-2.94229400	1.43160400
H	-2.44807600	-4.24068400	0.39229400
H	-5.07082600	-1.40728700	2.27921700
H	-4.38672600	-3.75728500	1.87371300
C	0.85661100	1.22779900	-1.18156100
H	0.88885000	1.05399200	-2.26226100
H	0.79298100	2.29140600	-0.93240600
C	-0.08372800	0.26220500	-0.46891900
C	1.70976500	0.34774900	-0.31246700
C	3.04520500	0.69829900	0.30178000
C	3.76466900	-0.22830000	1.10207600
C	3.64258000	1.94753300	-0.03106800
C	5.05350100	0.07666200	1.51871200
H	3.30558600	-1.17415600	1.37198900
C	4.92713800	2.24085100	0.40114000
H	3.10505800	2.65676900	-0.65256000
C	5.63407600	1.30441700	1.17404600
H	5.61124900	-0.63628100	2.11827300
H	5.39164500	3.18695400	0.13372000
H	6.64677400	1.53673100	1.50022300
C	0.92088200	-0.83002900	-0.20312900
Br	1.20200500	-2.36695200	-1.30173600
F	0.83385800	-1.51890000	1.72677300

:CCl_2	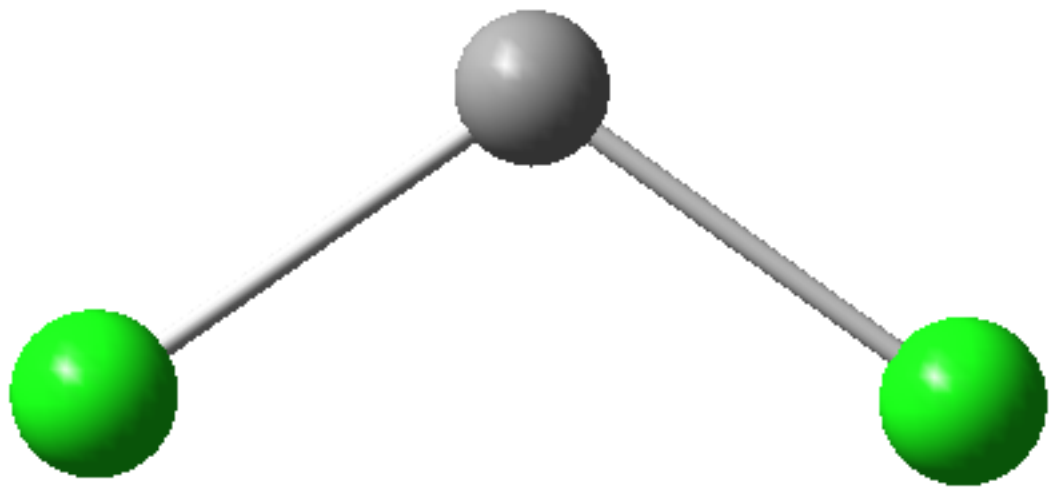		
Total Electronic Energy	Sum of electronic and zero-point Energies	Sum of electronic and thermal Enthalpies	Sum of electronic and thermal Free Energies
-958.388690	-958.384395	-958.380077	-958.410064

Cartesian Coordinates
C 0.00000000 0.00000000 0.84080300
Cl 0.00000000 1.39202100 -0.14837700
Cl 0.00000000 -1.39202100 -0.14837700

:CBr_2	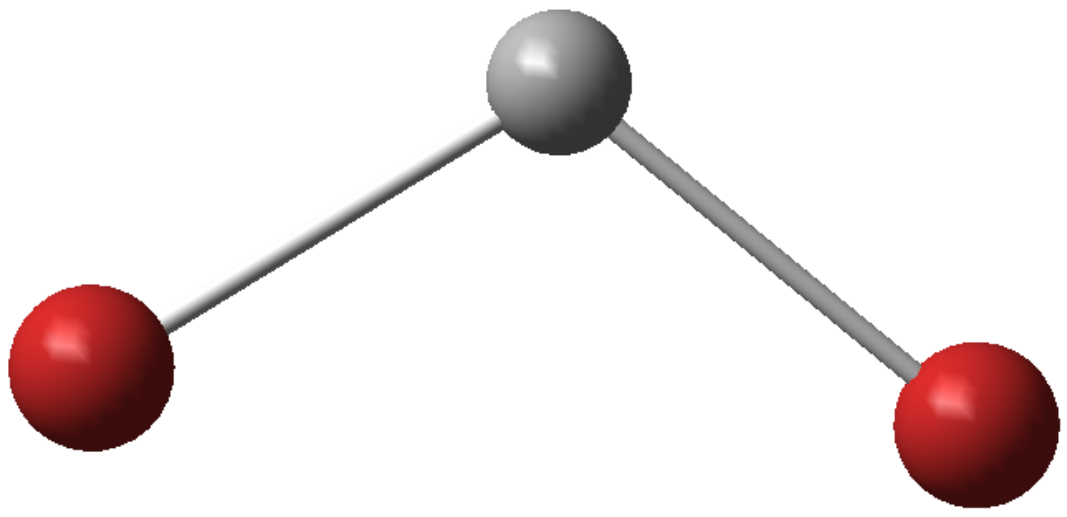		
Total Electronic Energy	Sum of electronic and zero-point Energies	Sum of electronic and thermal Enthalpies	Sum of electronic and thermal Free Energies
-5186.372081	-5186.368708	-5186.364094	-5186.396766

Cartesian Coordinates
C 0.00000000 0.00000000 0.99425100
Br 0.00000000 -1.54091600 -0.08522200
Br 0.00000000 1.54091600 -0.08522200

:CBrF	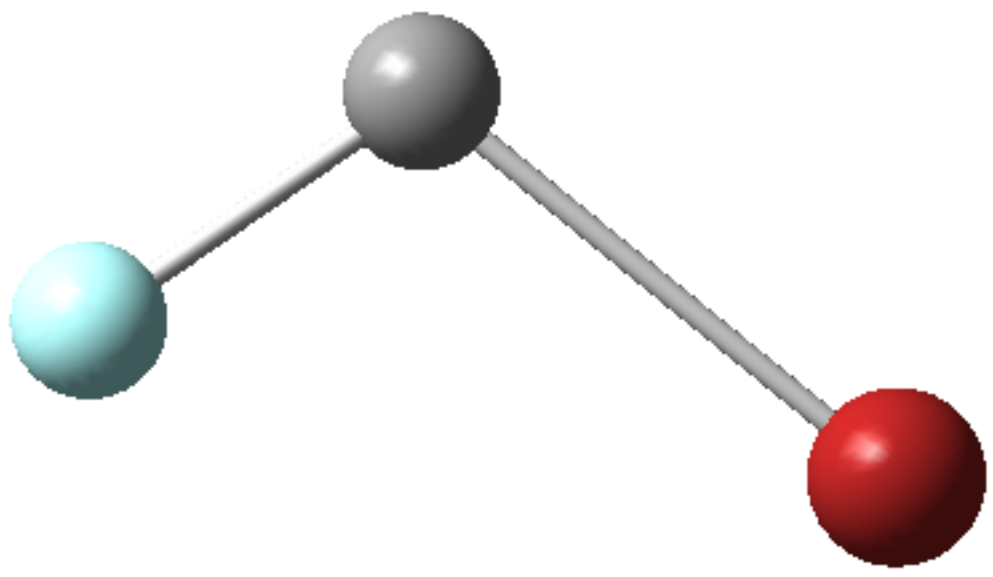		
Total Electronic Energy	Sum of electronic and zero-point Energies	Sum of electronic and thermal Enthalpies	Sum of electronic and thermal Free Energies
-2712.042058	-2712.036869	-2712.032604	-2712.063345

Cartesian Coordinates
C 0.62655600 -1.14576400 0.00000000
Br 0.00000000 0.68193500 0.00000000
F -0.41770400 -1.88812700 0.00000000

F^-	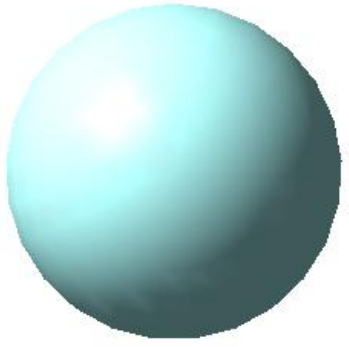		
Total Electronic Energy	Sum of electronic and zero-point Energies	Sum of electronic and thermal Enthalpies	Sum of electronic and thermal Free Energies
-99.947299	-99.947299	-99.944938	-99.961458
Cartesian Coordinates<br>F 0.00000000 0.00000000 0.00000000			

Cl^-	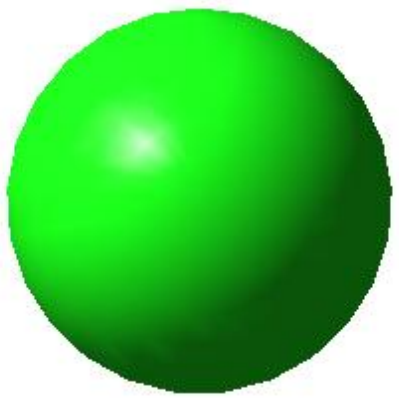		
Total Electronic Energy	Sum of electronic and zero-point Energies	Sum of electronic and thermal Enthalpies	Sum of electronic and thermal Free Energies
-460.346343	-460.346343	-460.343983	-460.361366
Cartesian Coordinates<br>Cl 0.00000000 0.00000000 0.00000000			

Br^-	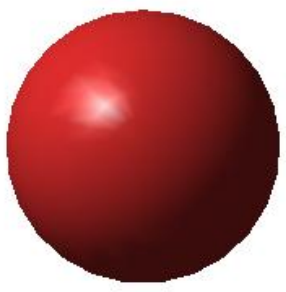		
Total Electronic Energy	Sum of electronic and zero-point Energies	Sum of electronic and thermal Enthalpies	Sum of electronic and thermal Free Energies
-2571.972013	-2571.972013	-2571.969653	-2571.988189
Cartesian Coordinates<br>Br 0.00000000 0.00000000 0.00000000			

7. References

1 A. Basheer, M. Mishima and I. Marek, *Org. Lett.*, 2011, **13**, 4076–4079.

2 F. Soddu, I. Mahdi, M. C. Cabua, F. Secci, P. Natho, R. Tassoni, P. Dambruoso, E. Mesto, M. Colella and R. Luisi, *Org. Lett.*, 2025, **27**, 5754–5759.

3 P. Natho, M. Colella, A. Vicenti, G. Romanazzi, F. Ullah, N. S. Sheikh, A. J. P. White, F. Pasca and R. Luisi, *Angew. Chem. Int. Ed.*, 2025, **64**, e202424346.

4 X. Cheng, L. Zhu, M. Lin, J. Chen and X. Huang, *Chem. Comm.*, 2017, **53**, 3745–3748.

5 D. J. Konowalchuk and D. G. Hall, *Angew. Chem. Int. Ed.,* 2023, **62**, e202313503

6 APEX 2, version 2010.3-0, Bruker (2010), Bruker AXS Inc., Madison, Wisconsin, USA

7 SAINT, version V7.60A, Bruker (2009), Bruker AXS Inc., Madison, Wisconsin, USA

8 SADABS, version 2008/1, Bruker (2008), Bruker AXS Inc., Madison, Wisconsin, USA

9 Sheldrick, G.M. (2008) XPREP Version 2008/2. Bruker AXS Inc., Madison

10 L. Palatinus, G. Chapuis *J. Appl. Cryst.* **2007**, 40, 786-790

11 R.I. Cooper, A. L. Thompson, D. J. Watkin *J. Appl. Crystallogr.* **2010**, 43,1100-1107

12 P. W. Betteridge, J. R. Carruthers, R. I. Cooper, K. Prout, & D. J. Watkin, *J. Appl. Cryst.* (**2003**), 36, 1487

13 Frisch, M., et al., *01; Gaussian, Inc*. Wallingford, CT, 2009

14 G. A. DiLabio and A. Otero-de-la-Roza, 2016, pp. 1–97.

15 Gray, M., *A Quantum-Mechanical Investigation of Noncovalent Interactions: Validation and Application of Density Functional Theory and Symmetry-Adapted Perturbation Theory*. 2025, The Ohio State University.

16 H. Jin, P. Li, P. Cui, J. Shi, W. Zhou, X. Yu, W. Song and C. Cao, *Nat. Commun.*, 2022, **13**, 723.

17 S. Mkrtchyan, V. B. Purohit, S. Sarfaraz, M. Yar, K. Ayub and V. O. Iaroshenko, *ACS Sustain. Chem. Eng.*, 2023, **11**, 8406–8412.

18 S. Mkrtchyan, M. Shkoor, S. Sarfaraz, K. Ayub and V. O. Iaroshenko, *Org. Biomol. Chem.*, 2023, **21**, 6549–6555.

19 S. Bibi, S. Sarfaraz, M. Yar, M. I. Zaman, A. Niaz, A. Khan, M. A. Hashmi and K. Ayub, *J. Mol. Liq.*, 2022, **366**, 120144.

20 A. Mukhtar, S. Sarfaraz and K. Ayub, *RSC Adv.*, 2022, **12**, 24397–24411.

21 S. Sarfaraz, M. Yar, A. Ali Khan, R. Ahmad and K. Ayub, *J. Mol. Liq.*, 2022, **352**, 118652.

22 Y. S. S. Al-Faiyz, S. Sarfaraz, M. Yar, S. Munsif, A. A. Khan, B. Amin, N. S. Sheikh and K. Ayub, *Nanomaterials*, 2023, **13**, 251.

23 H. Ullah, A.-H. A. Shah, S. Bilal and K. Ayub, *The Journal of Physical Chemistry C*, 2013, **117**, 23701–23711.

24 N. V. Bondarev, K. P. Katin, V. B. Merinov, A. I. Kochaev, S. Kaya and M. M. Maslov, *physica status solidi (RRL) – Rapid Research Letters*, 2022, **16**, 2100191.

25 V. S. Sastri and J. R. Perumareddi, *Corrosion*, 1997, **53**, 617–622.

26 M. Hagar, H. A. Ahmed, G. Aljohani and O. A. Alhaddad, *Int. J. Mol. Sci.*, 2020, **21**, 3922.

27 M. Noreen, N. Rasool, Y. Gull, M. Zubair, T. Mahmood, K. Ayub, F.-H. Nasim, A. Yaqoob, M. Zia-Ul-Haq and V. De Feo, *Molecules*, 2015, **20**, 19914–19928.

28 M. Özcan, İ. Dehri and M. Erbil, *Appl. Surf. Sci.*, 2004, **236**, 155–164.

8. NMR Spectra

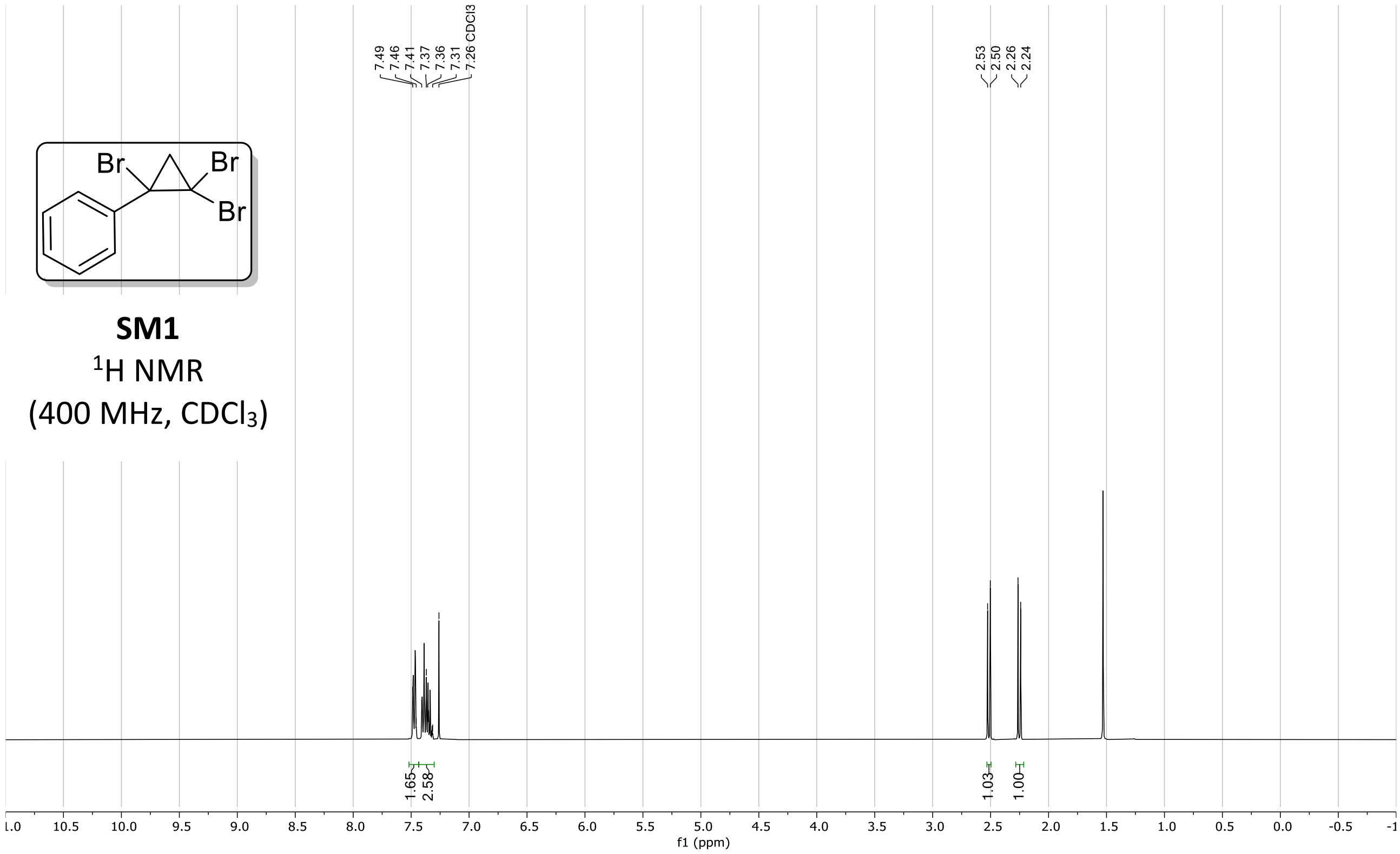

SM1

^{1}H NMR

(400 MHz, $CDCl_3$)

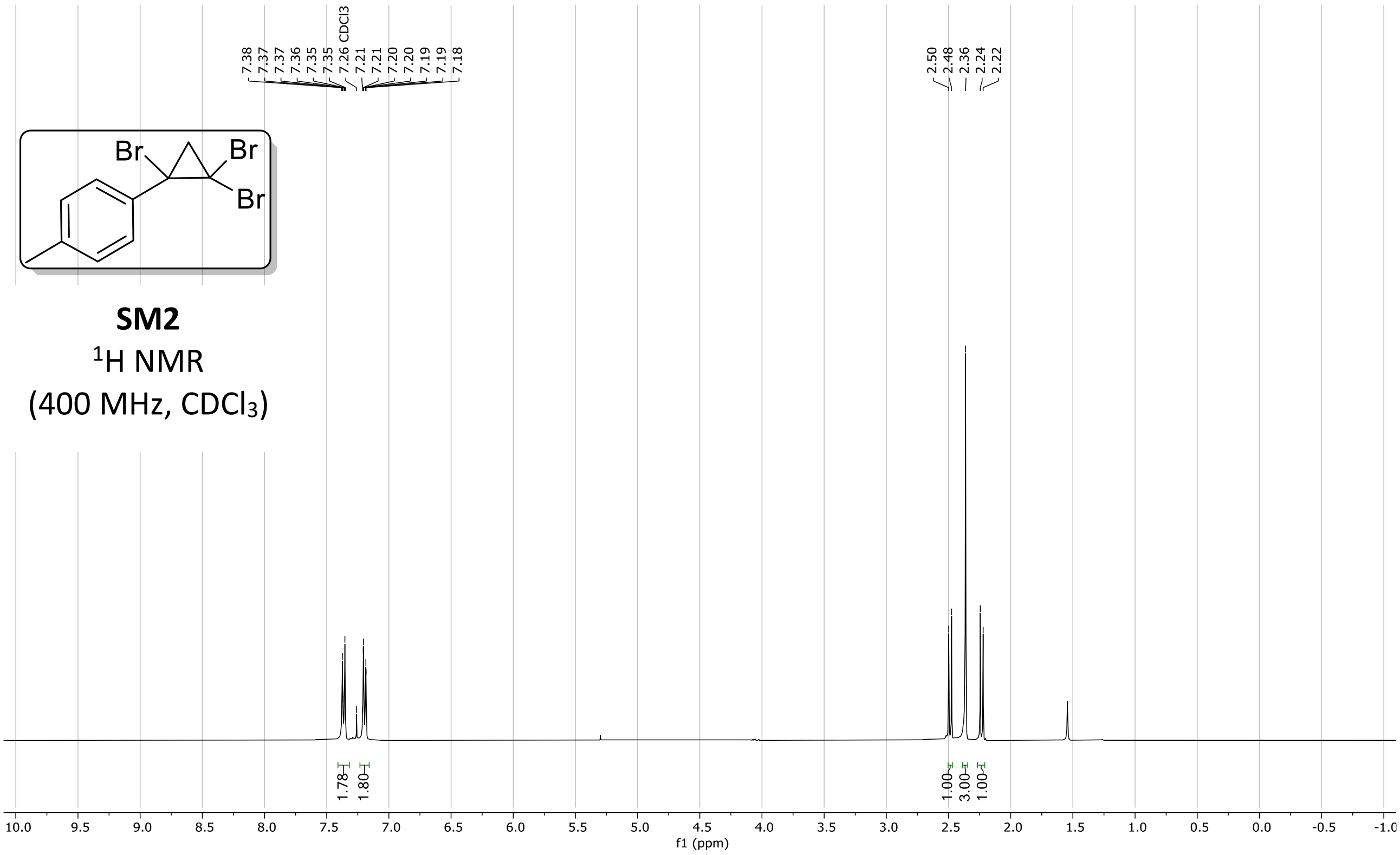

SM2

^{1}H NMR

(400 MHz, $CDCl_3$)

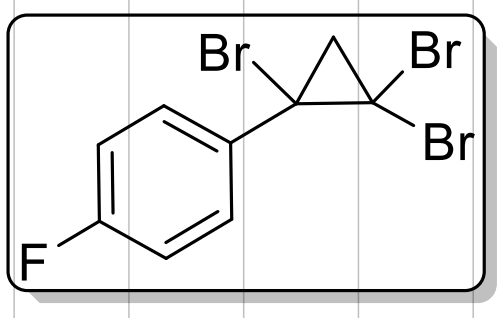

SM3

^{1}H NMR

(400 MHz, $CDCl_3$)

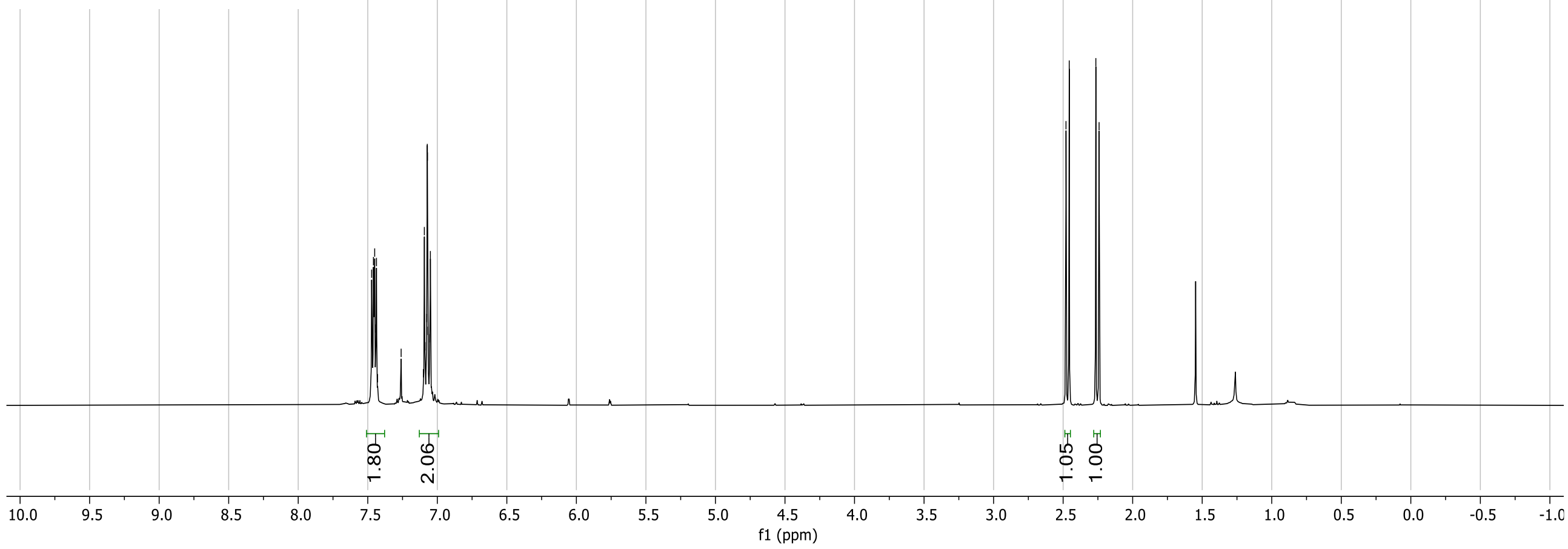

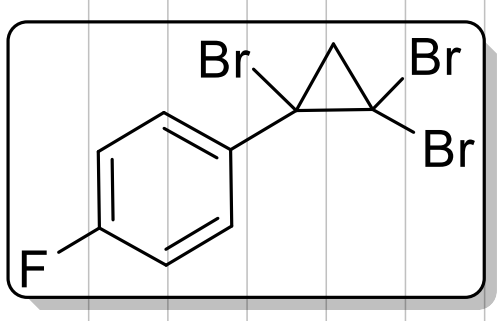

SM3

^{19}F NMR

(377 MHz, $CDCl_3$)

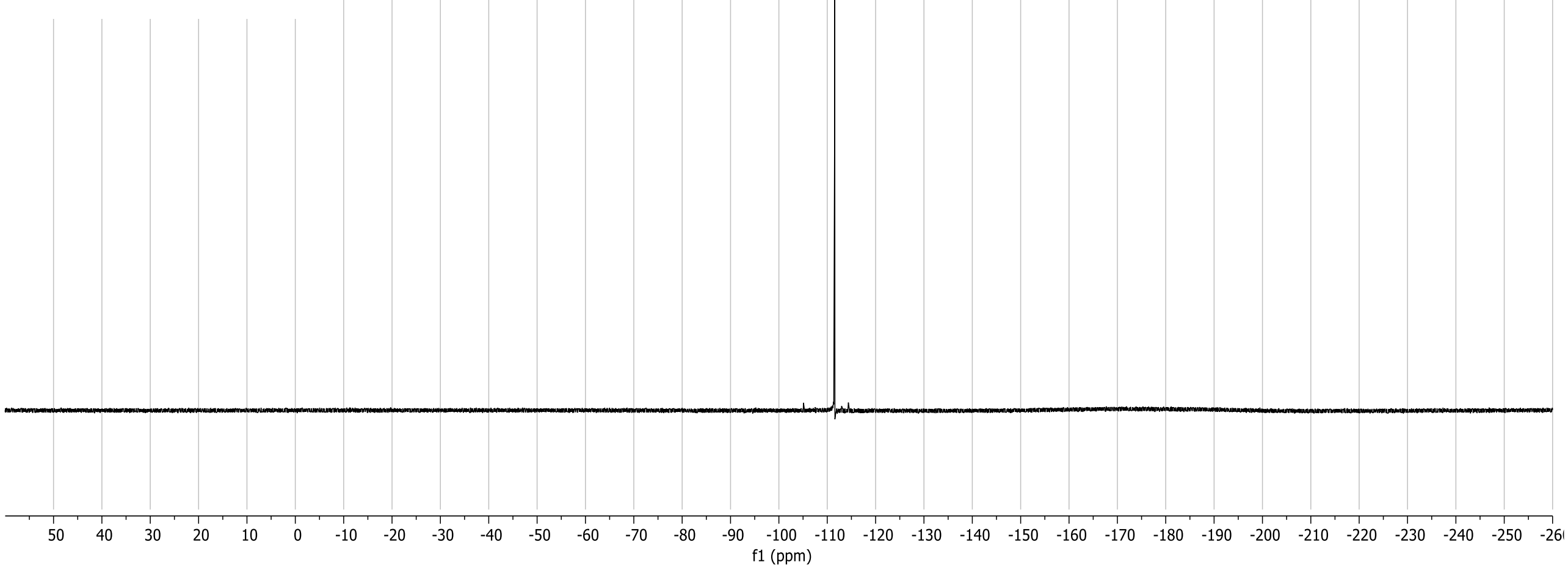

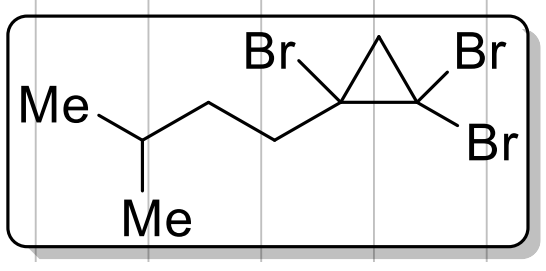

SM4
^{1}H NMR
(400 MHz, $CDCl_3$)

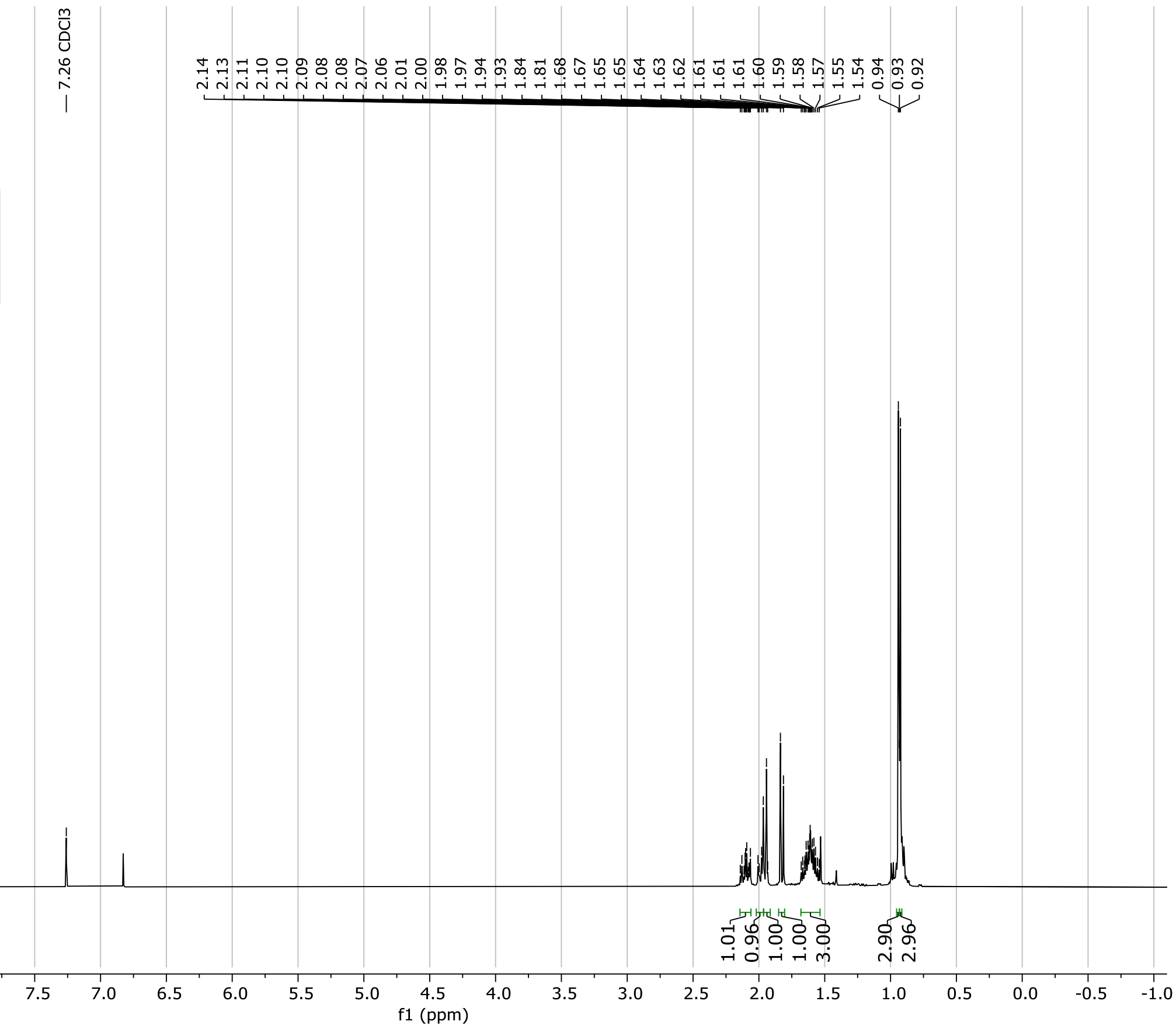

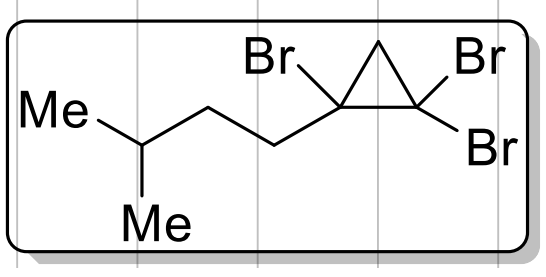

SM4
$^{13}C\{^{1}H\}$ NMR
(101 MHz, $CDCl_3$)

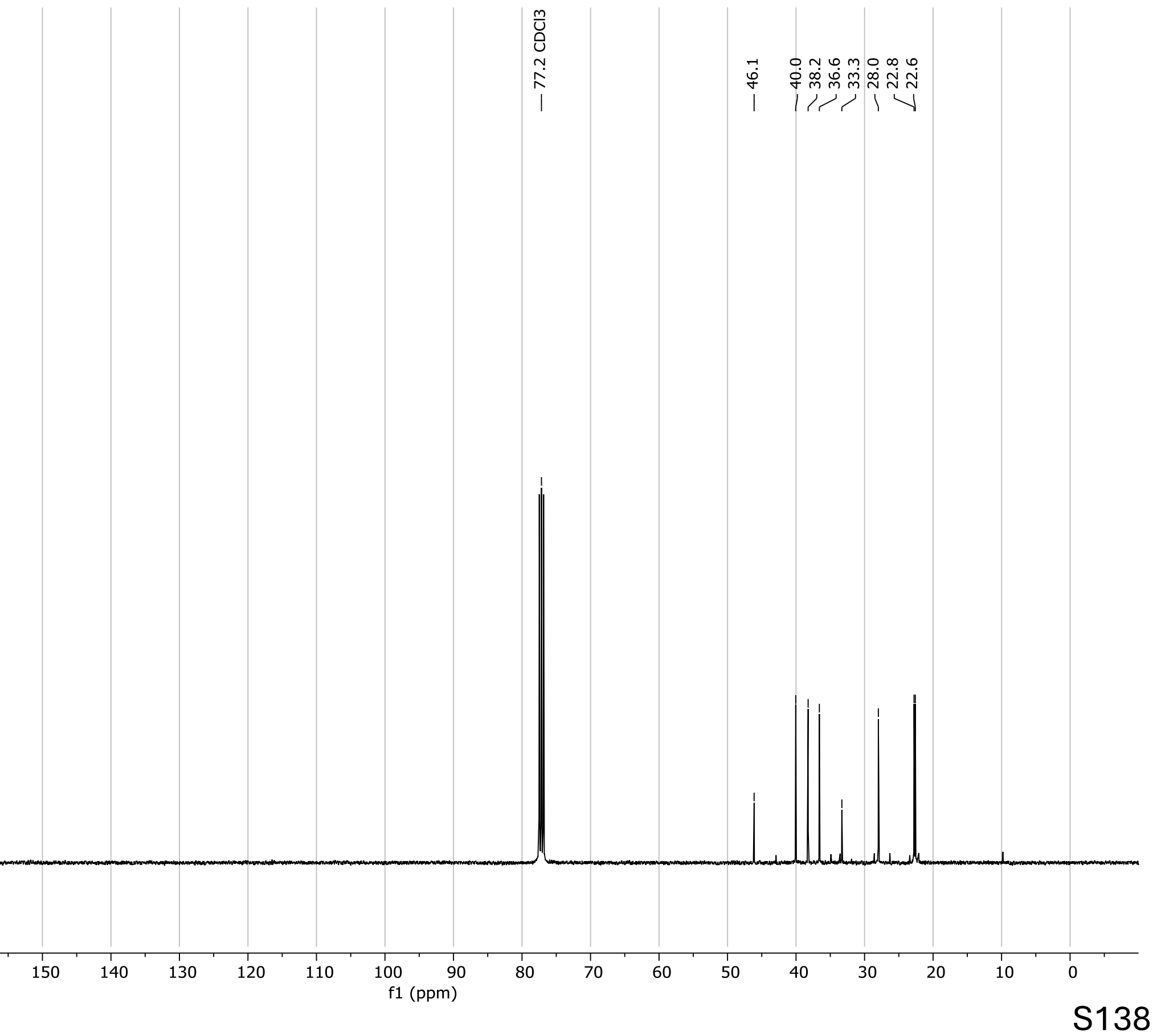

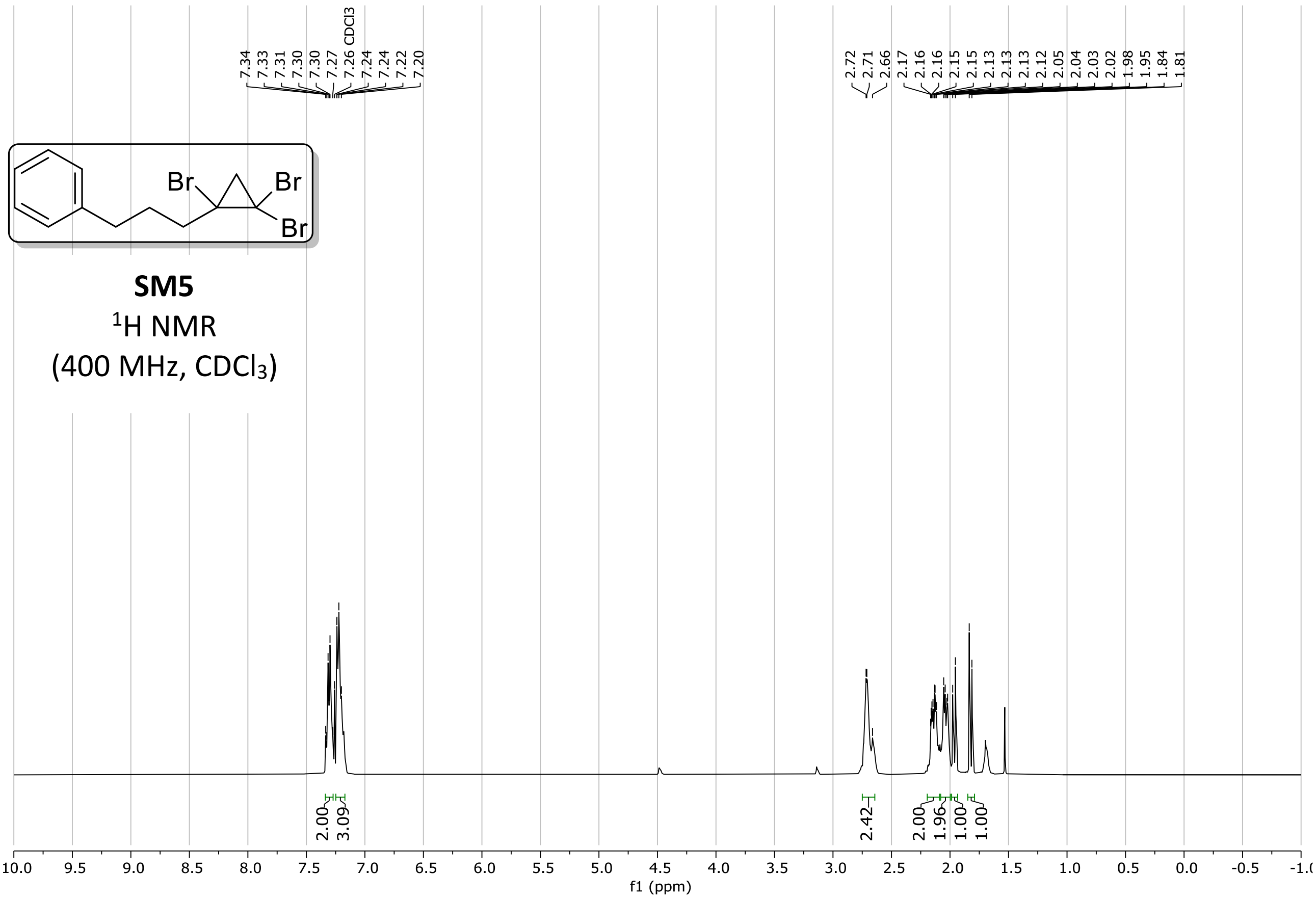

SM5
^{1}H NMR
(400 MHz, $CDCl_3$)

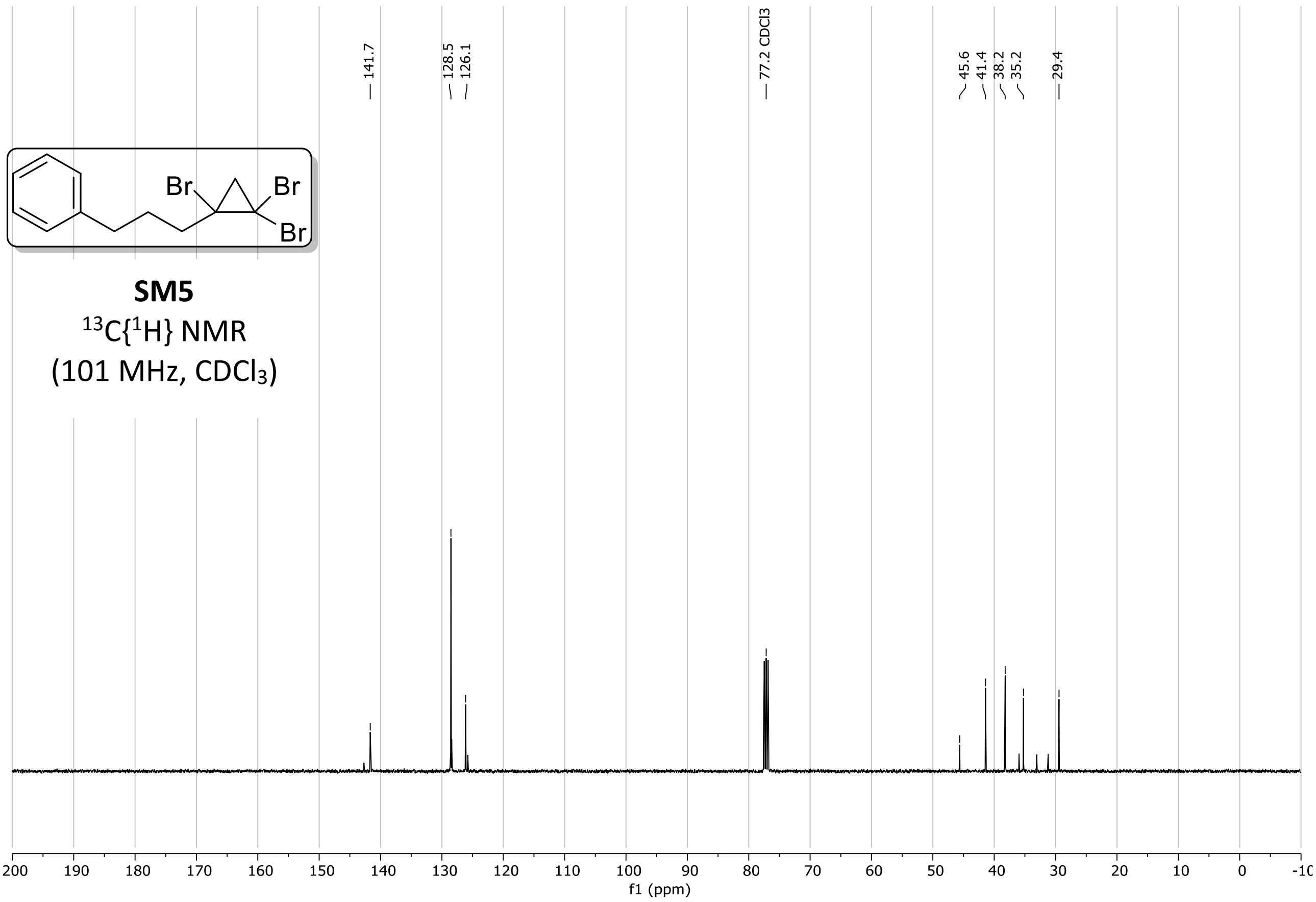

SM5
$^{13}C\{^{1}H\}$ NMR
(101 MHz, $CDCl_3$)

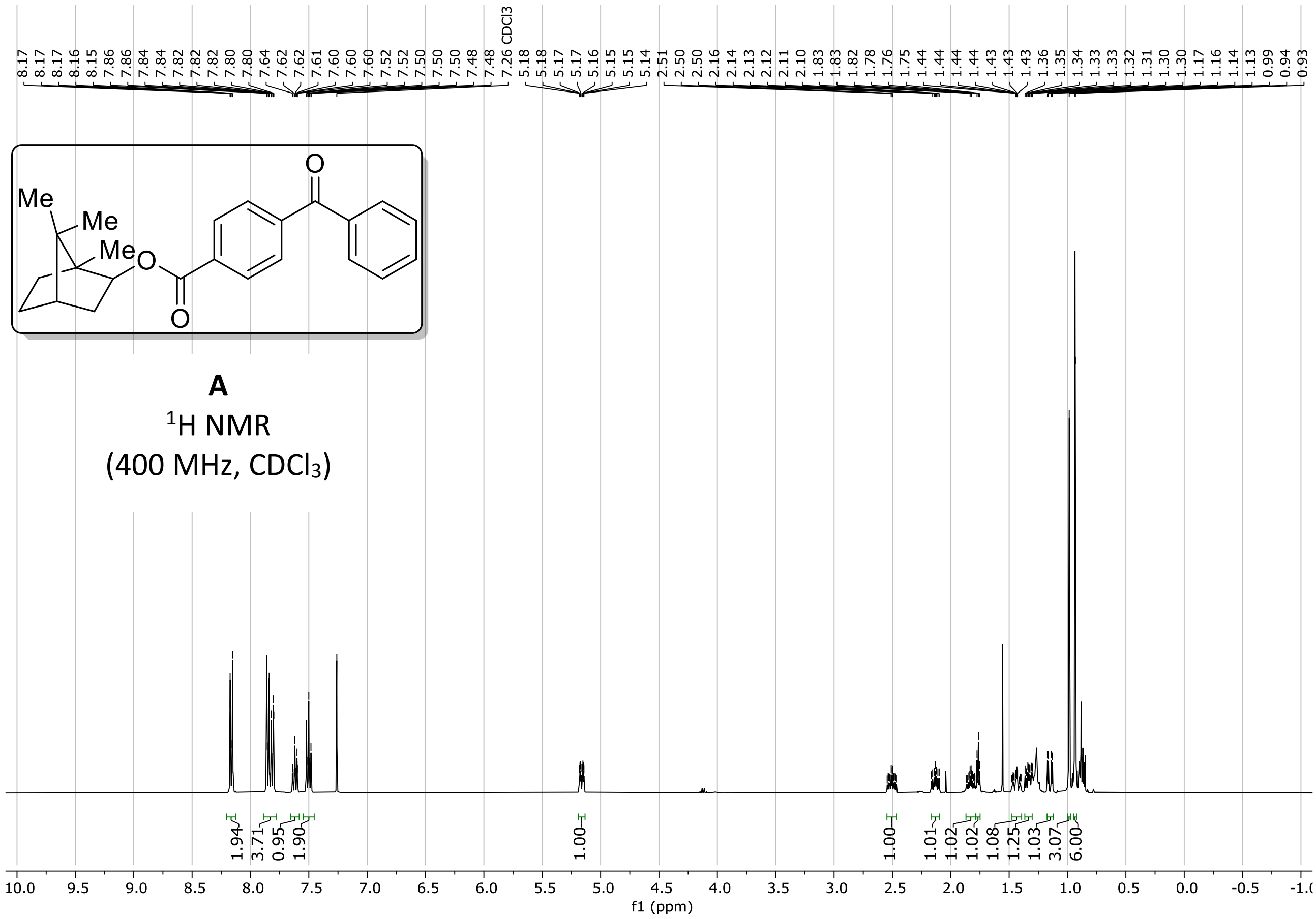

Me
Me
Me
O
O
O
A
1H NMR
(400 MHz, CDCl3)
CDCl3
1.94
3.71
0.95
1.90
1.00
1.00
1.01
1.02
1.02
1.08
1.25
1.03
3.07
6.00
f1 (ppm)

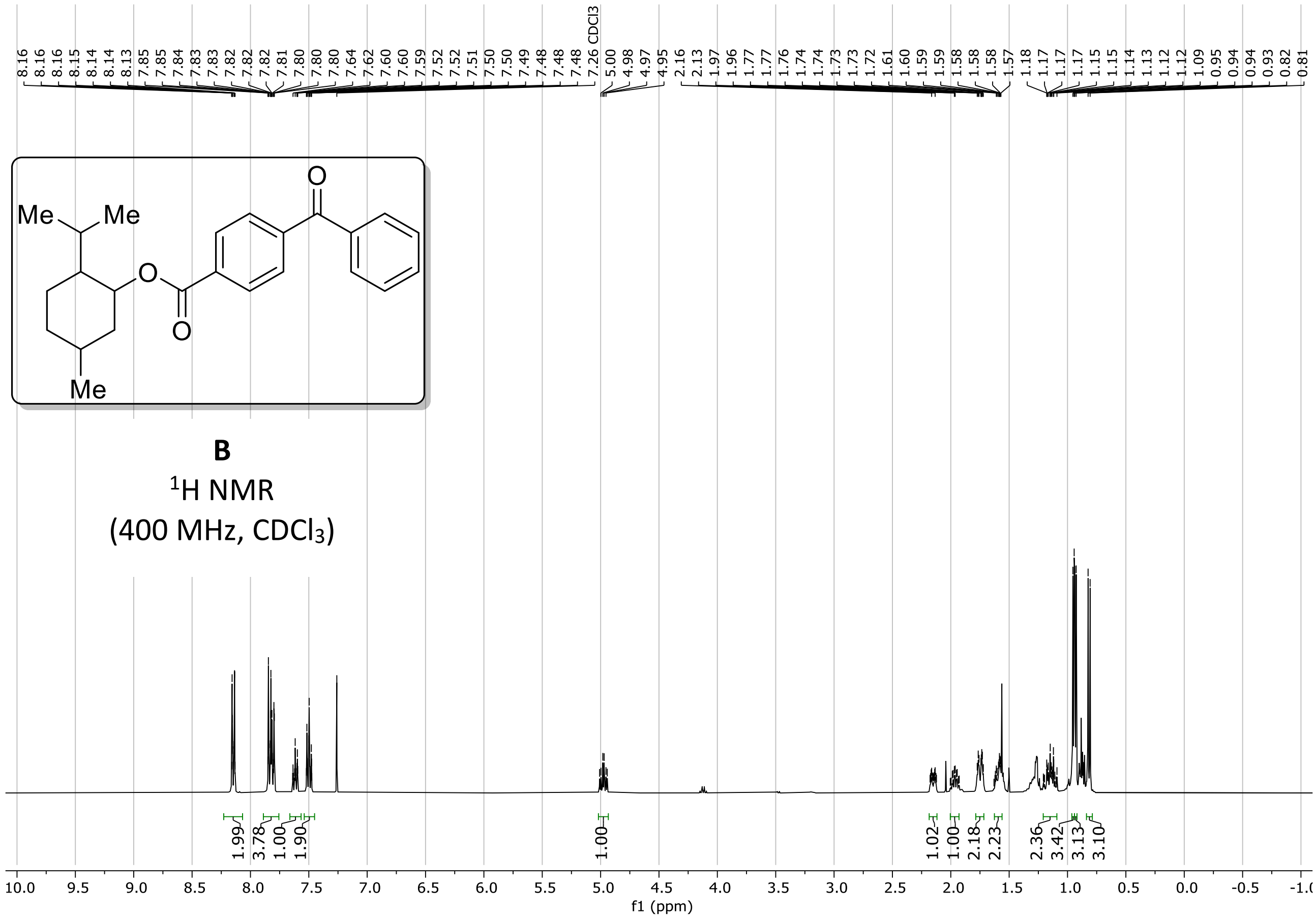

B

^{1}H NMR

(400 MHz, $CDCl_3$)

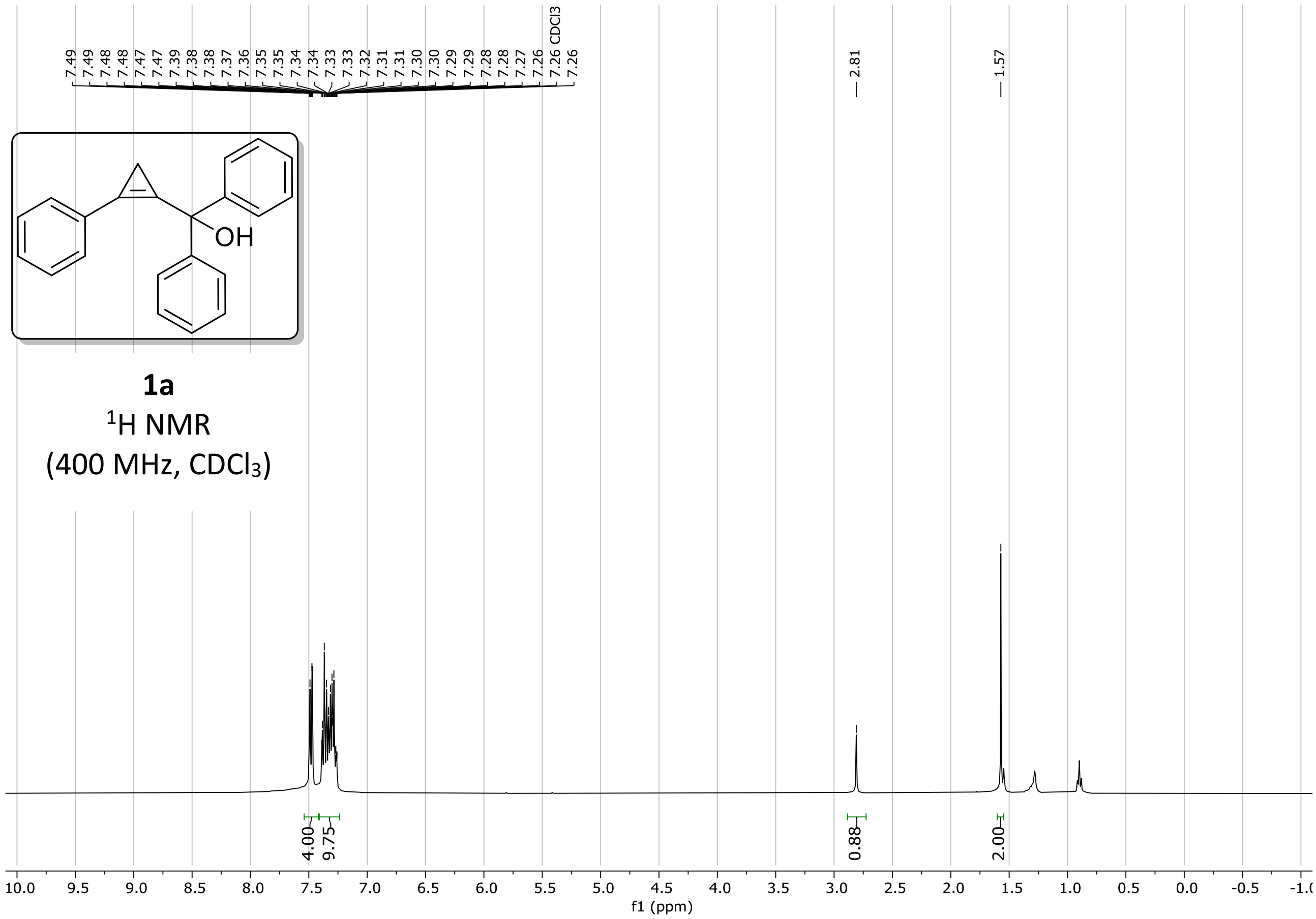
7.49 7.49 7.48 7.48 7.47 7.47 7.39 7.38 7.38 7.37 7.36 7.35 7.35 7.34 7.33 7.33 7.32 7.31 7.31 7.30 7.30 7.29 7.29 7.28 7.28 7.27 7.26 7.26 CDCl3 7.26
2.81
1.57
OH
1a
1H NMR
(400 MHz, CDCl3)
4.00
9.75
0.88
2.00
10.0 9.5 9.0 8.5 8.0 7.5 7.0 6.5 6.0 5.5 5.0 4.5 4.0 3.5 3.0 2.5 2.0 1.5 1.0 0.5 0.0 -0.5
f1 (ppm)

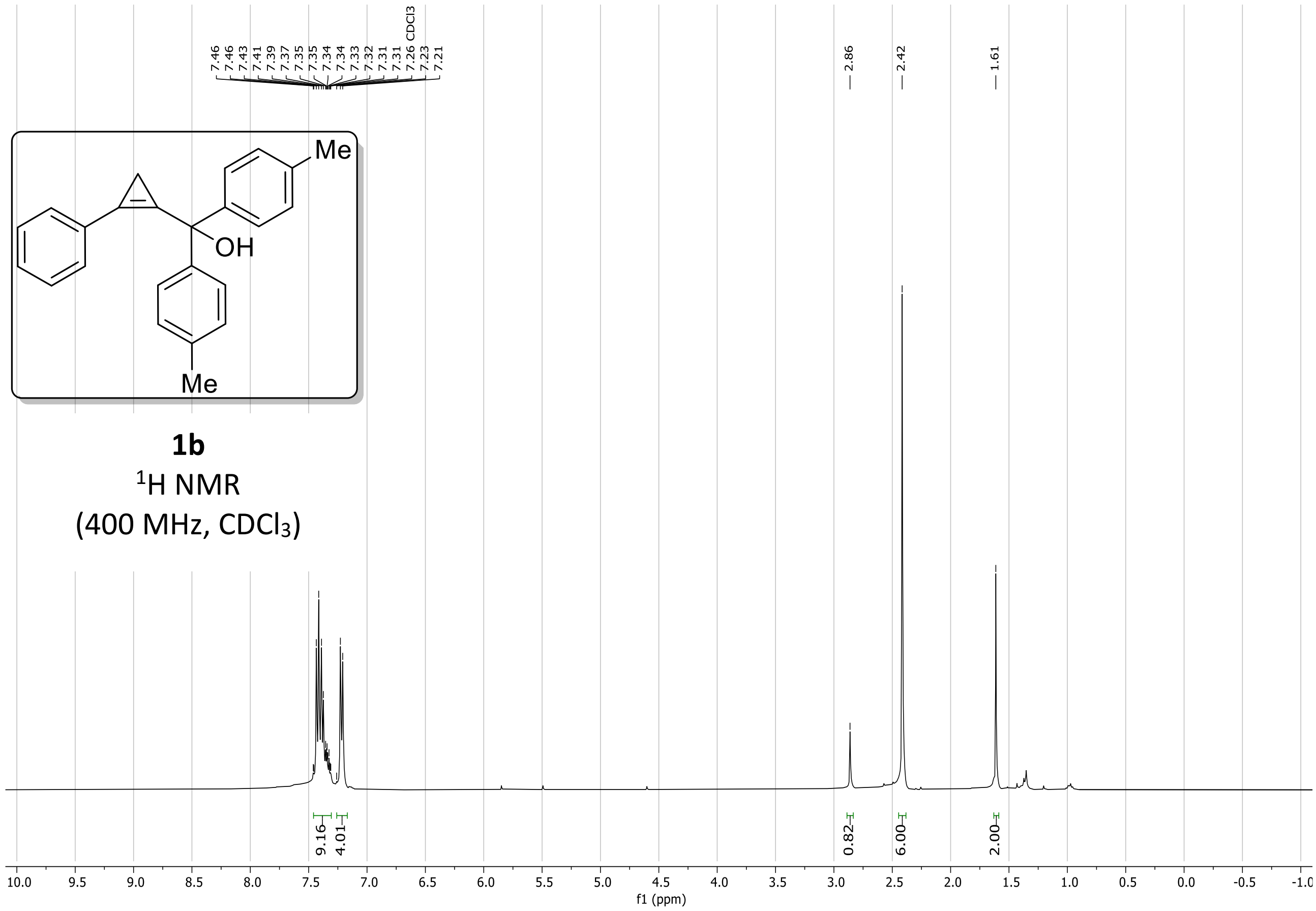
7.46 7.46 7.43 7.41 7.39 7.37 7.35 7.35 7.34 7.34 7.33 7.32 7.31 7.31 7.26 CDCl3 7.23 7.21
2.86
2.42
1.61
Me
OH
Me
1b
^{1}H NMR
(400 MHz, $CDCl_3$)
9.16
4.01
0.82
6.00
2.00
10.0 9.5 9.0 8.5 8.0 7.5 7.0 6.5 6.0 5.5 5.0 4.5 4.0 3.5 3.0 2.5 2.0 1.5 1.0 0.5 0.0 -0.5 -1.0
f1 (ppm)

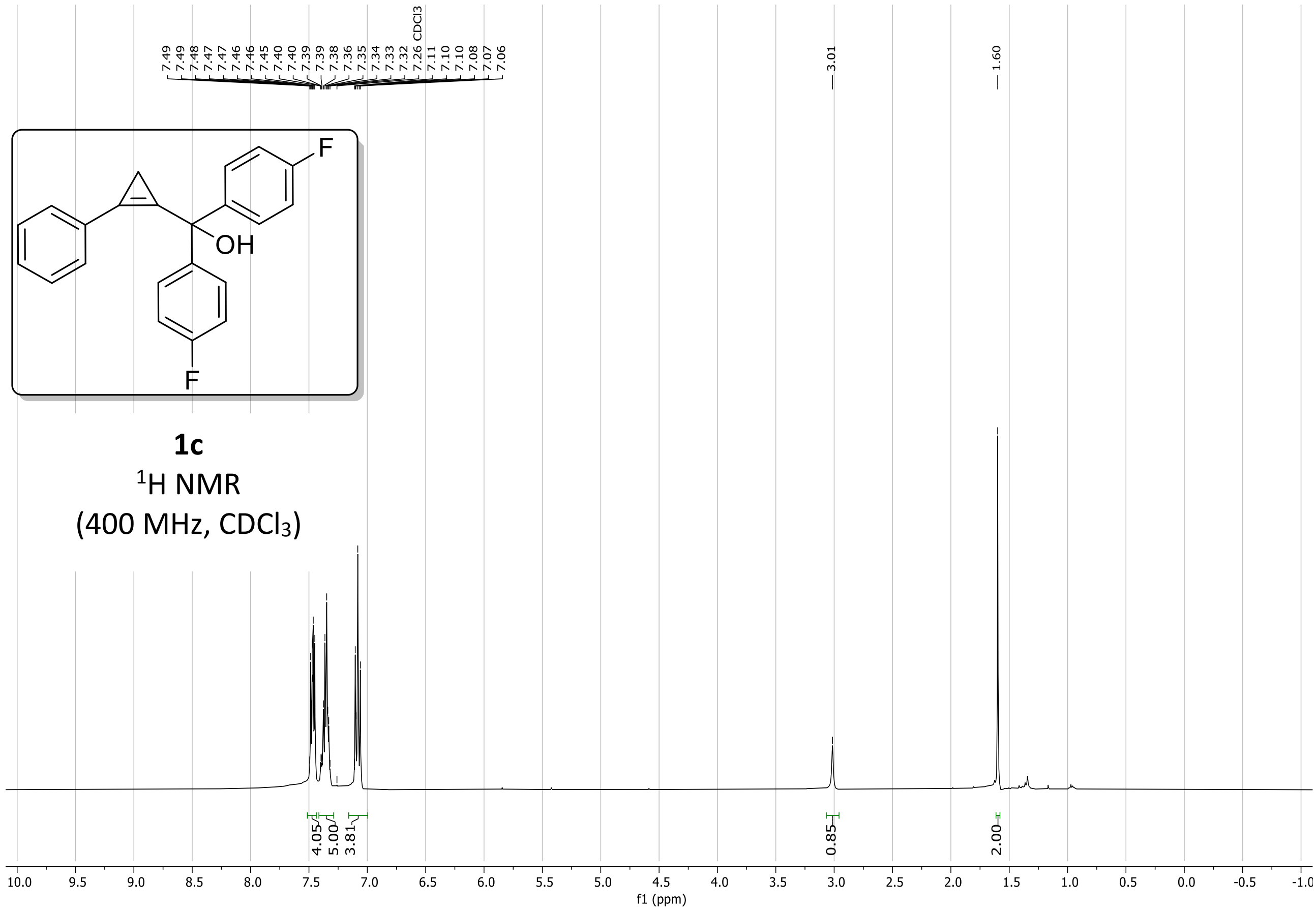
7.49
7.49
7.48
7.47
7.47
7.46
7.46
7.45
7.40
7.40
7.39
7.39
7.38
7.36
7.35
7.34
7.33
7.32
7.26 CDCl3
7.11
7.10
7.10
7.08
7.07
7.06
3.01
1.60
F
OH
F
1c
^{1}H NMR
(400 MHz, $CDCl_3$)
4.05
5.00
3.81
0.85
2.00
f1 (ppm)

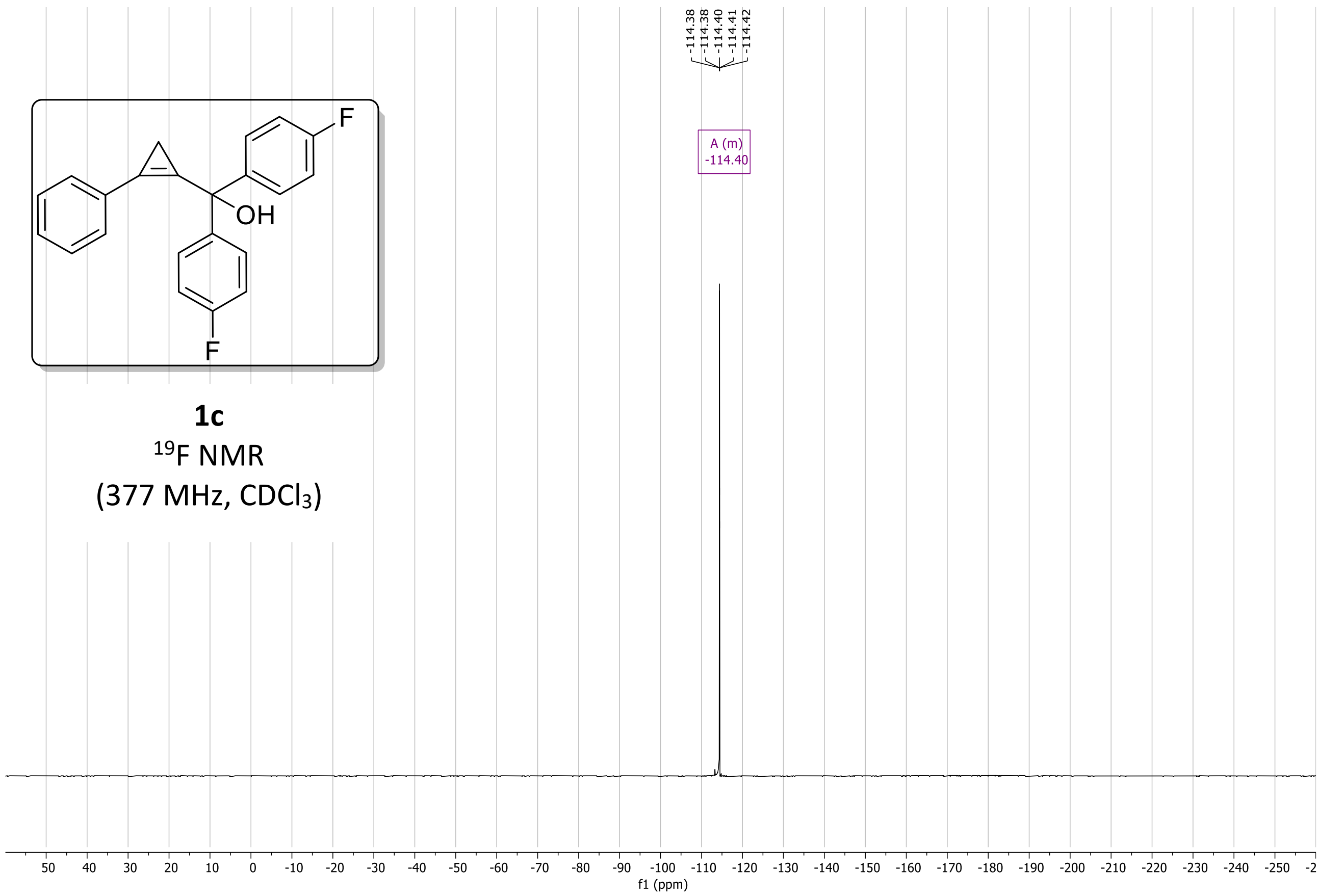
-114.38
-114.38
-114.40
-114.41
-114.42
A (m)
-114.40
F
OH
F
1c
^{19}F NMR
(377 MHz, $CDCl_3$)
f1 (ppm)

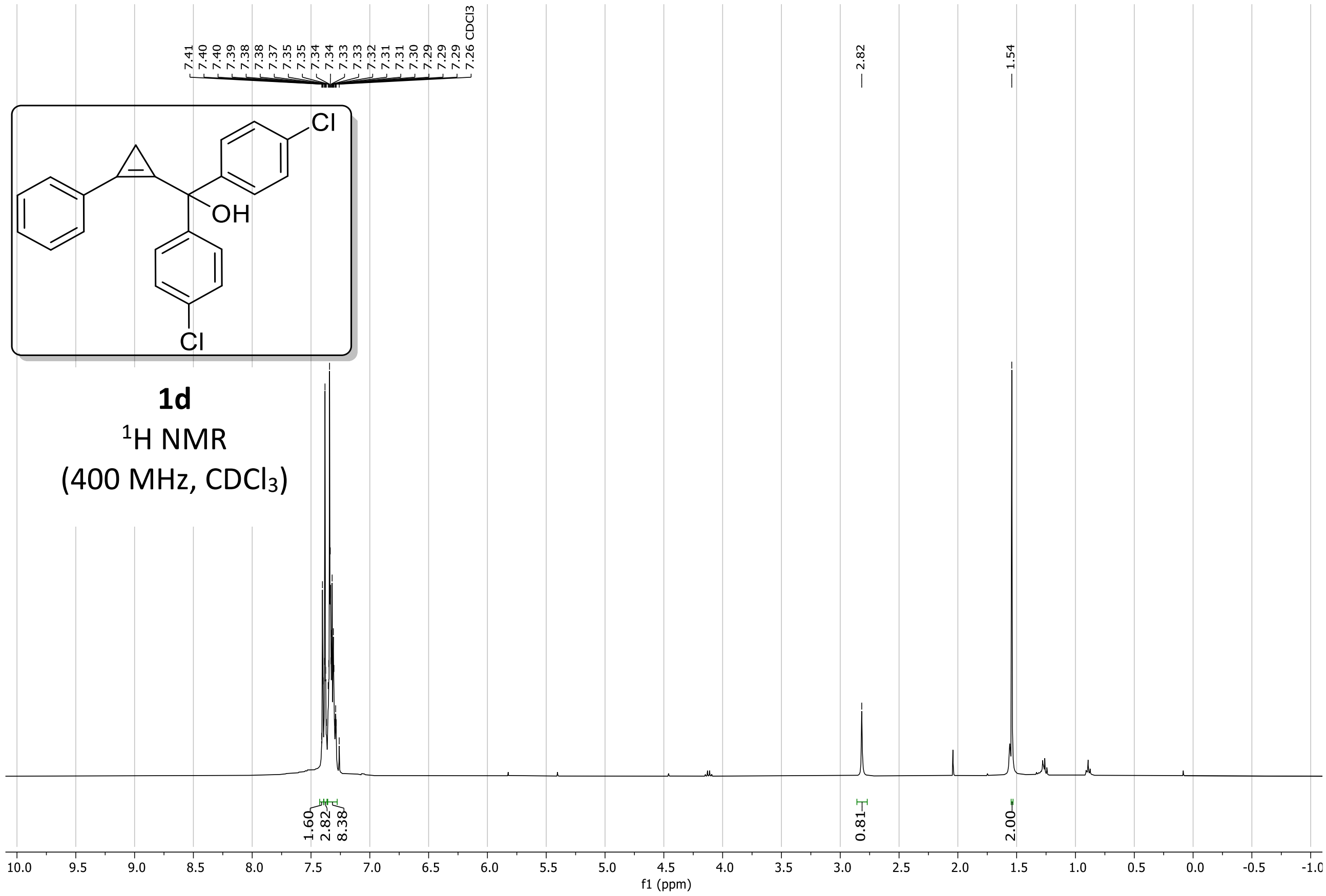

1d

^{1}H NMR

(400 MHz, $CDCl_3$)

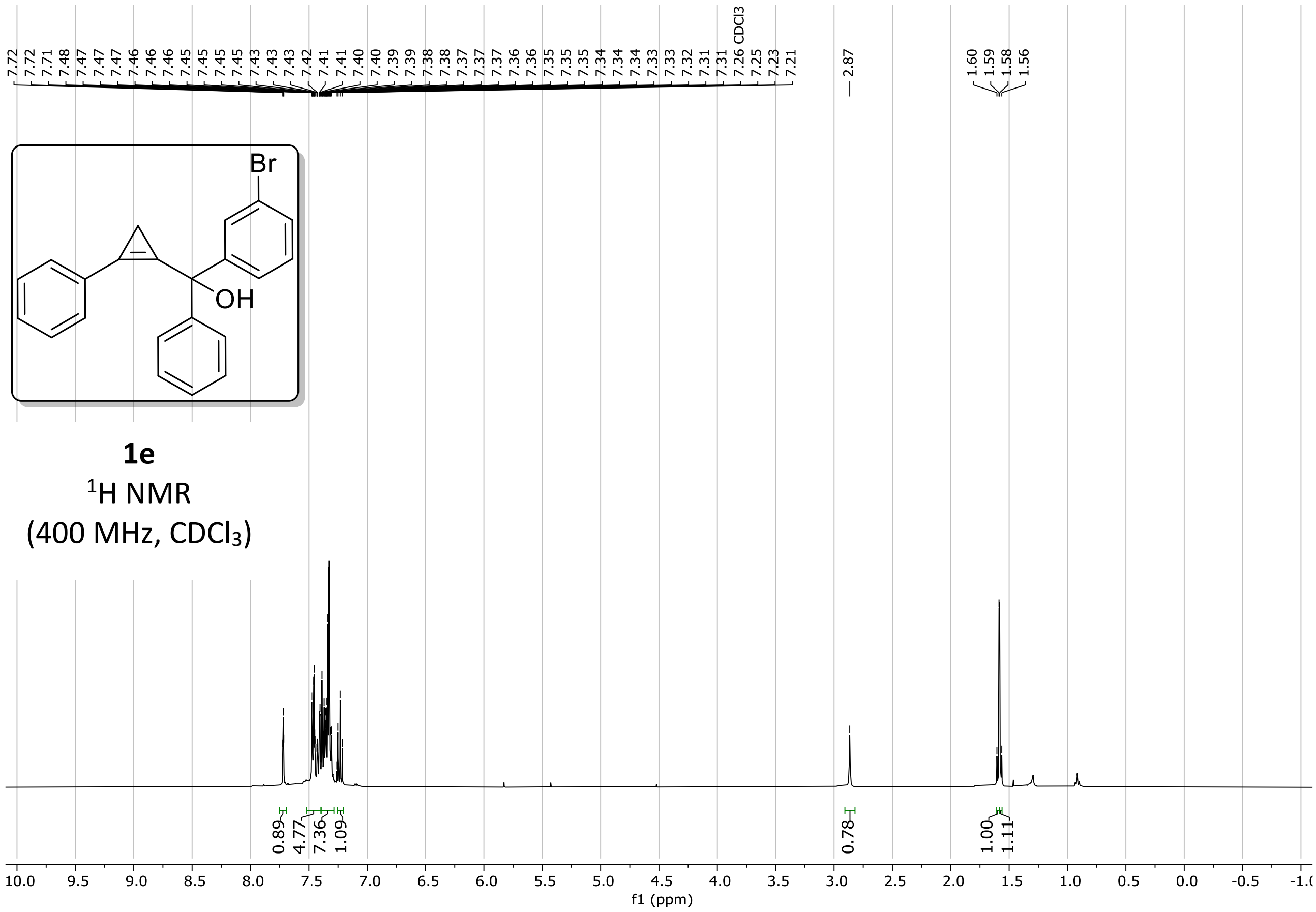
7.72
7.72
7.71
7.48
7.47
7.47
7.47
7.46
7.46
7.45
7.45
7.45
7.45
7.43
7.43
7.42
7.41
7.41
7.40
7.40
7.39
7.39
7.38
7.38
7.37
7.37
7.37
7.36
7.36
7.35
7.35
7.35
7.34
7.34
7.33
7.33
7.32
7.31
7.31
7.26 CDCl3
7.25
7.23
7.21
2.87
1.60
1.59
1.58
1.56
Br
OH
1e
1H NMR
(400 MHz, CDCl3)
0.89
4.77
7.36
1.09
0.78
1.00
1.11
f1 (ppm)

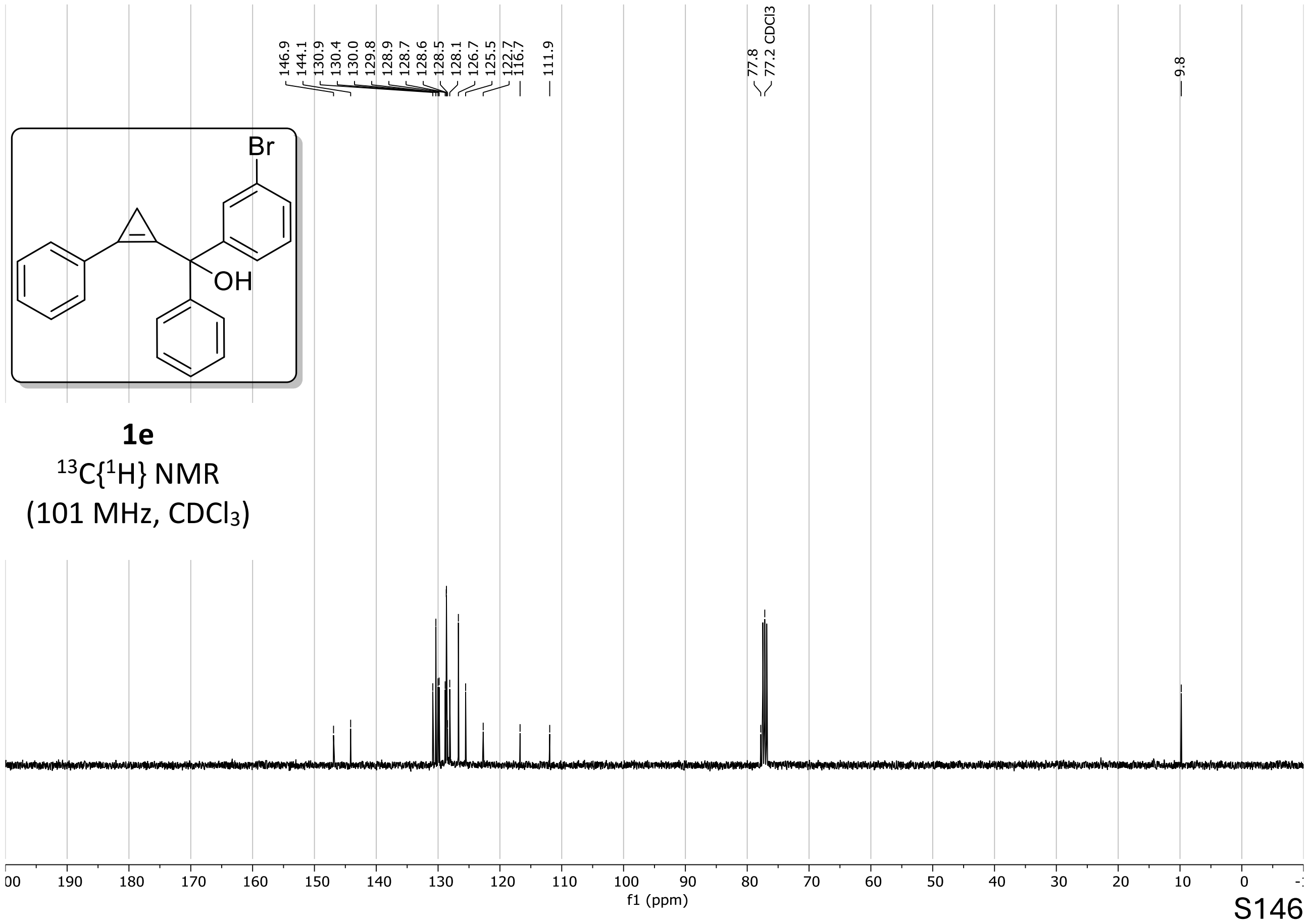
146.9
144.1
130.9
130.4
130.0
129.8
128.9
128.7
128.6
128.5
128.1
126.7
125.5
122.7
116.7
111.9
77.8
77.2 CDCl3
9.8
Br
OH
1e
13C{1H} NMR
(101 MHz, CDCl3)
f1 (ppm)

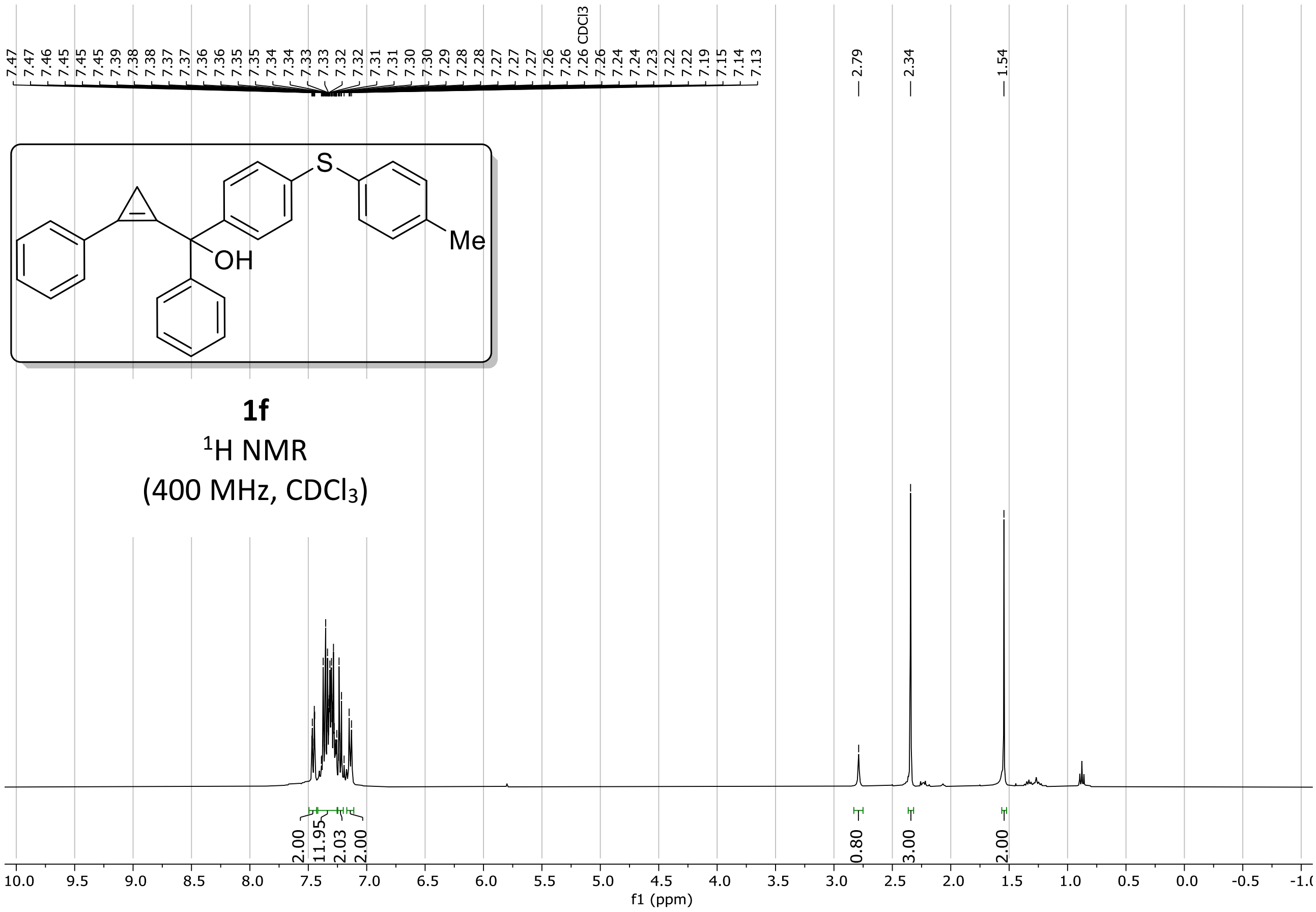

1f
^{1}H NMR
(400 MHz, $CDCl_3$)

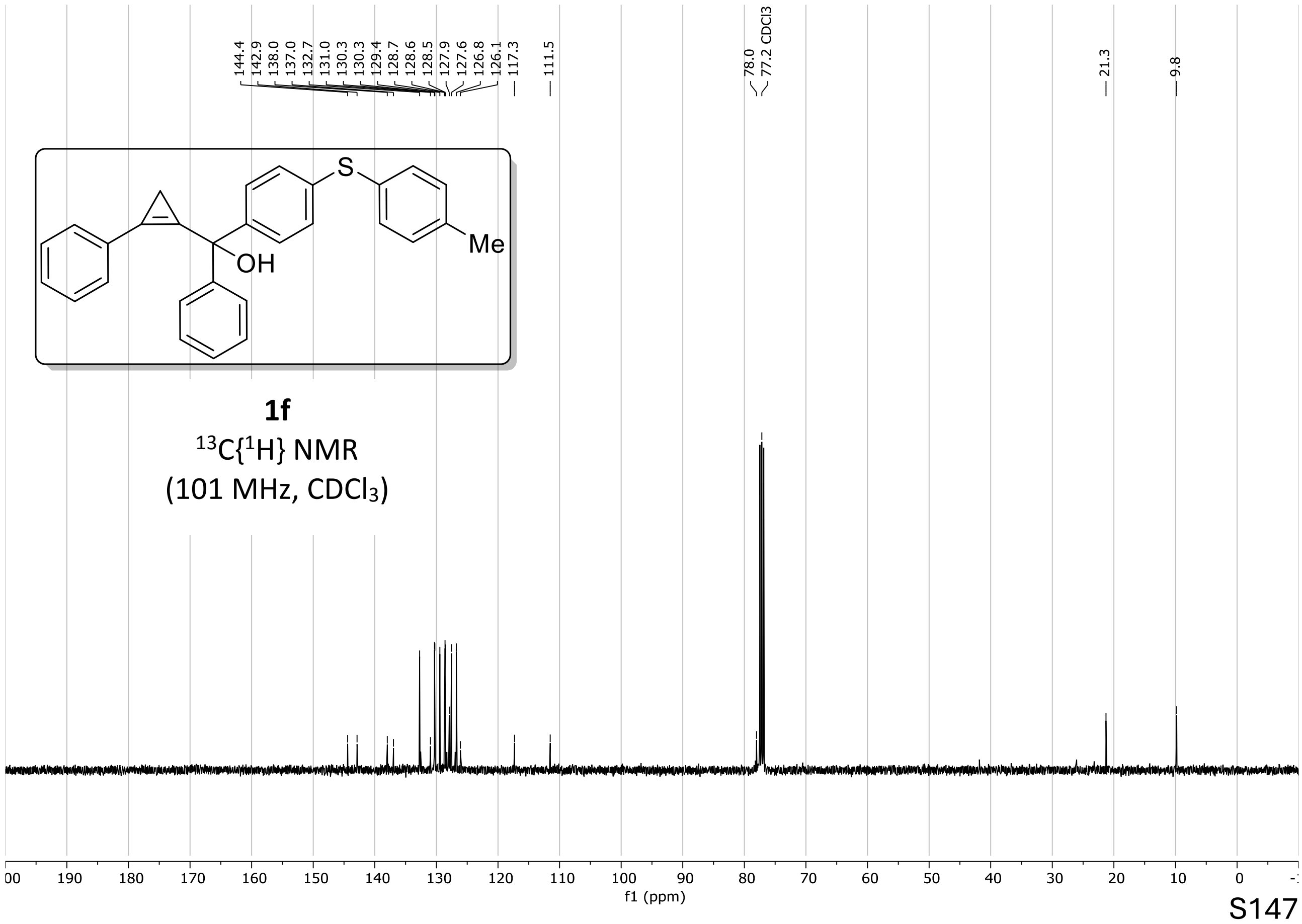

1f
$^{13}C\{^{1}H\}$ NMR
(101 MHz, $CDCl_3$)

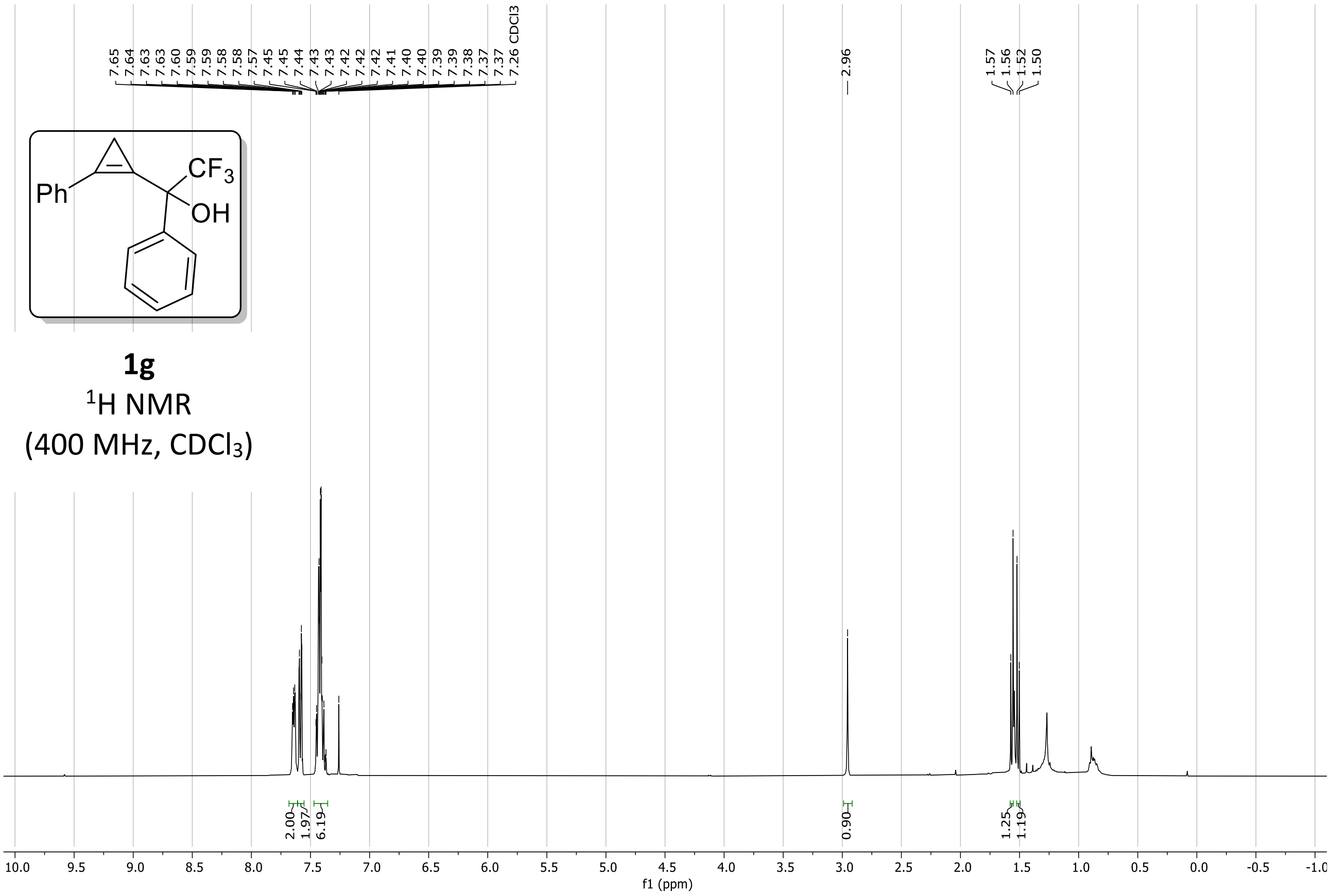

1g
^{1}H NMR
(400 MHz, $CDCl_3$)

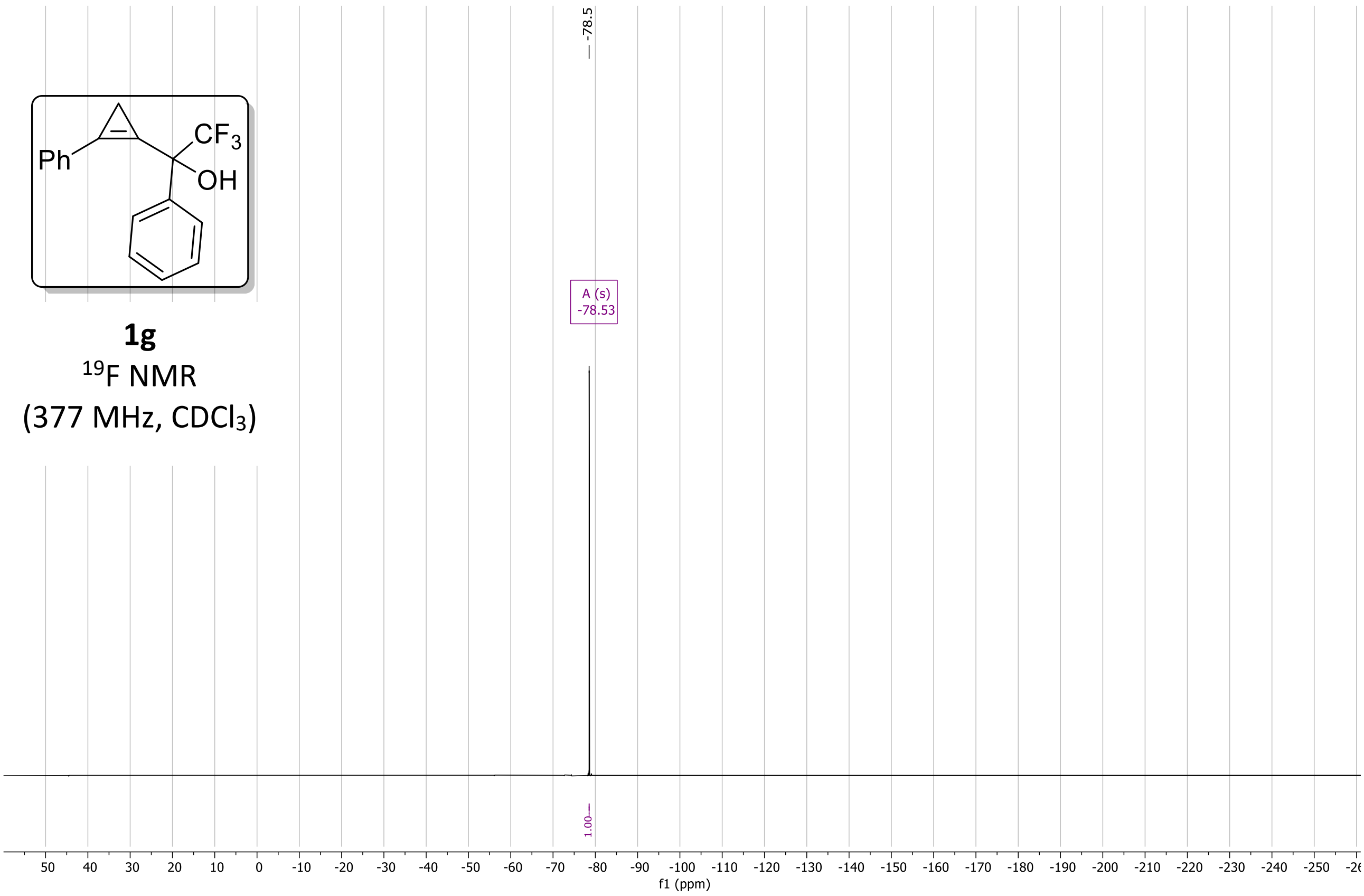

1g
^{19}F NMR
(377 MHz, $CDCl_3$)

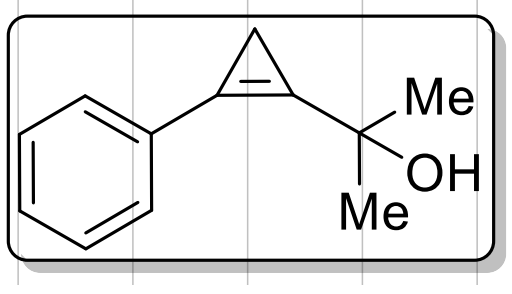

1h
^{1}H NMR
(400 MHz, $CDCl_3$)

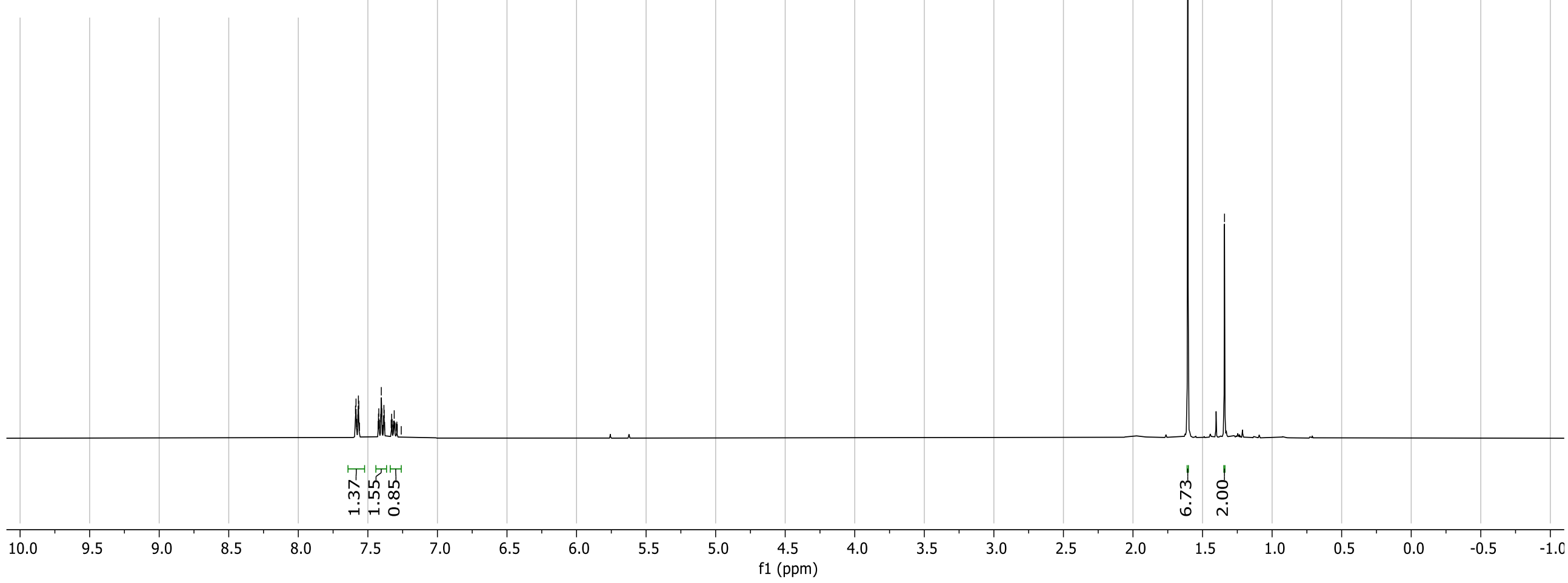

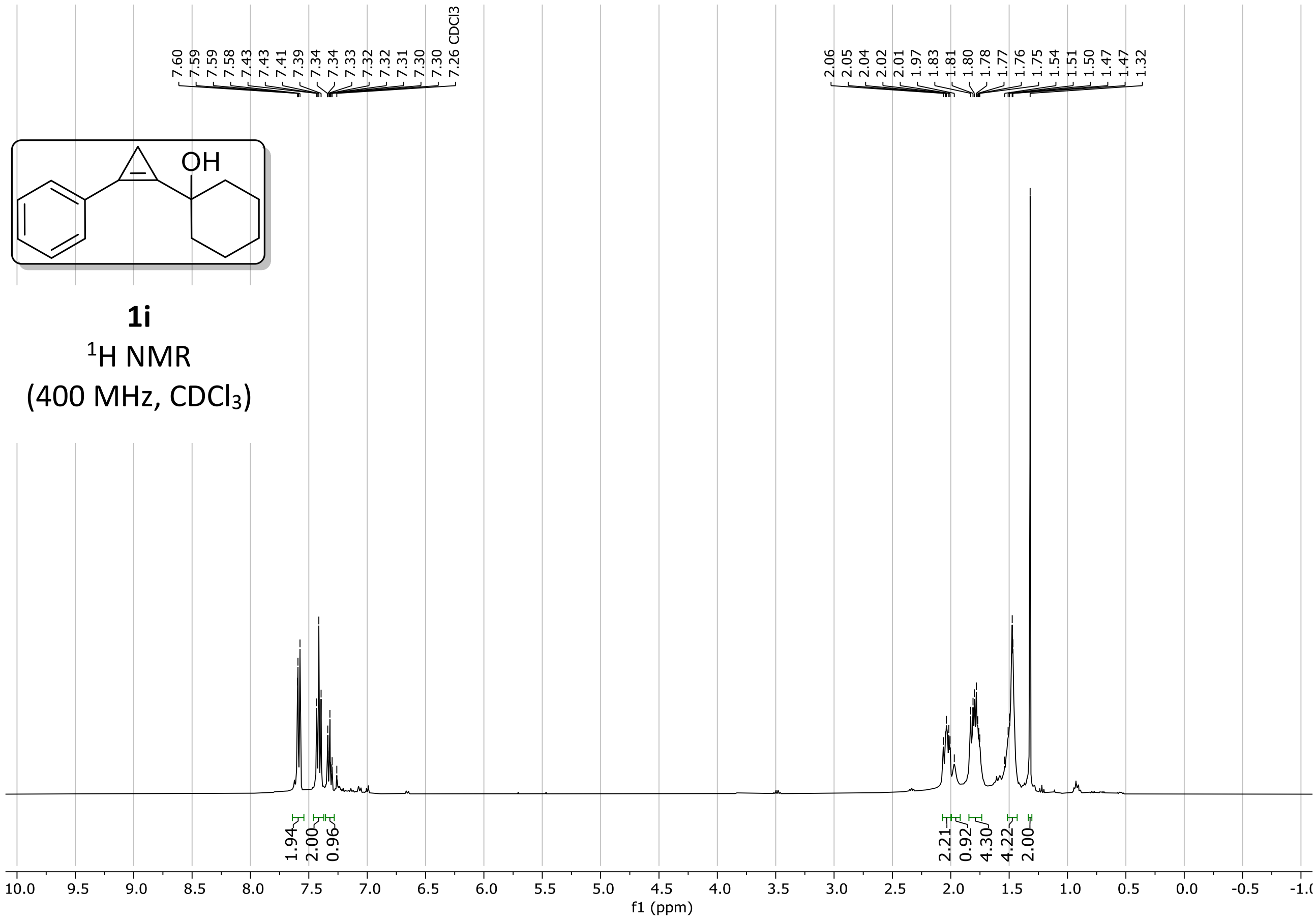

1i

^1H NMR

(400 MHz, $CDCl_3$)

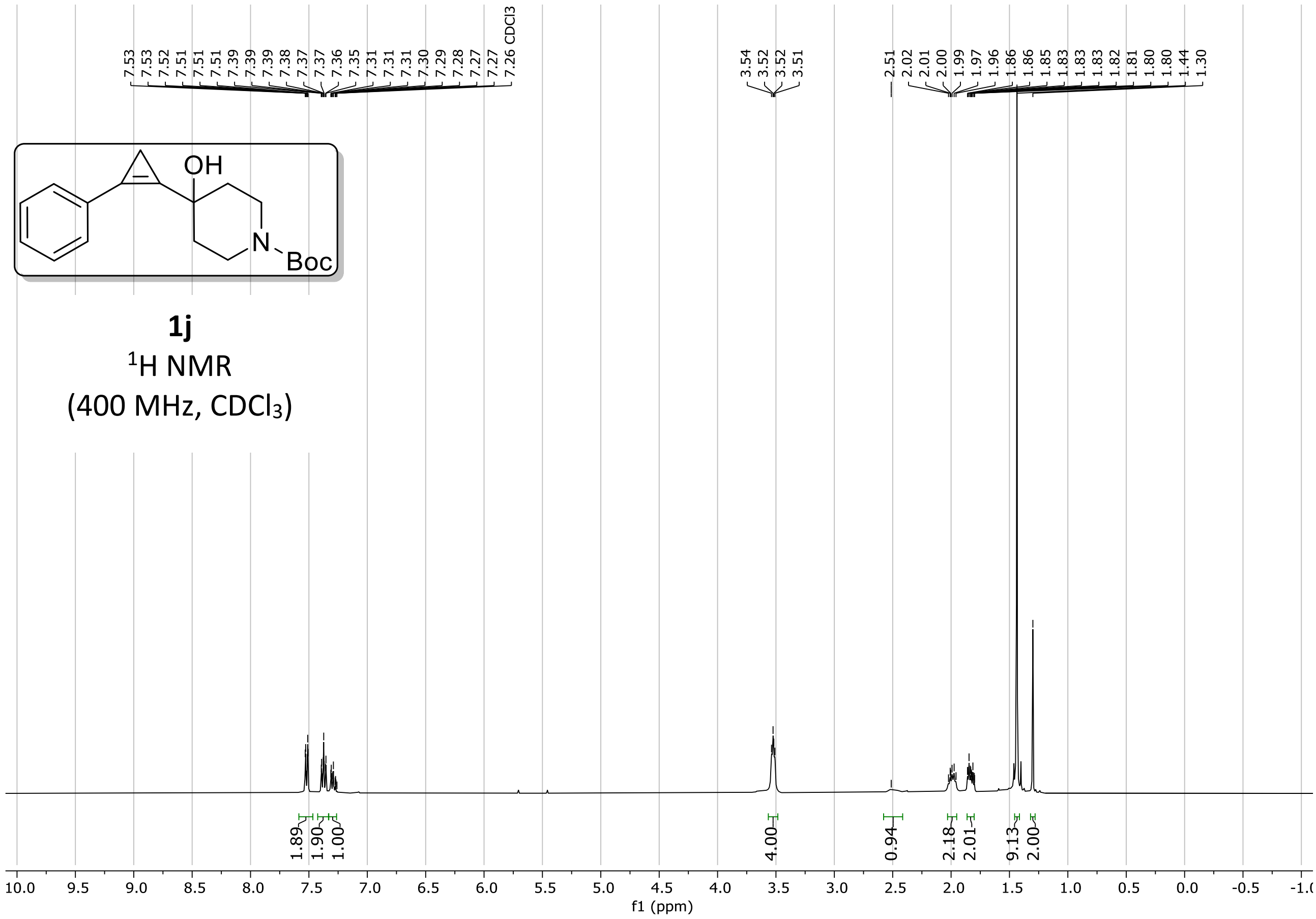

1j
^{1}H NMR
(400 MHz, $CDCl_3$)

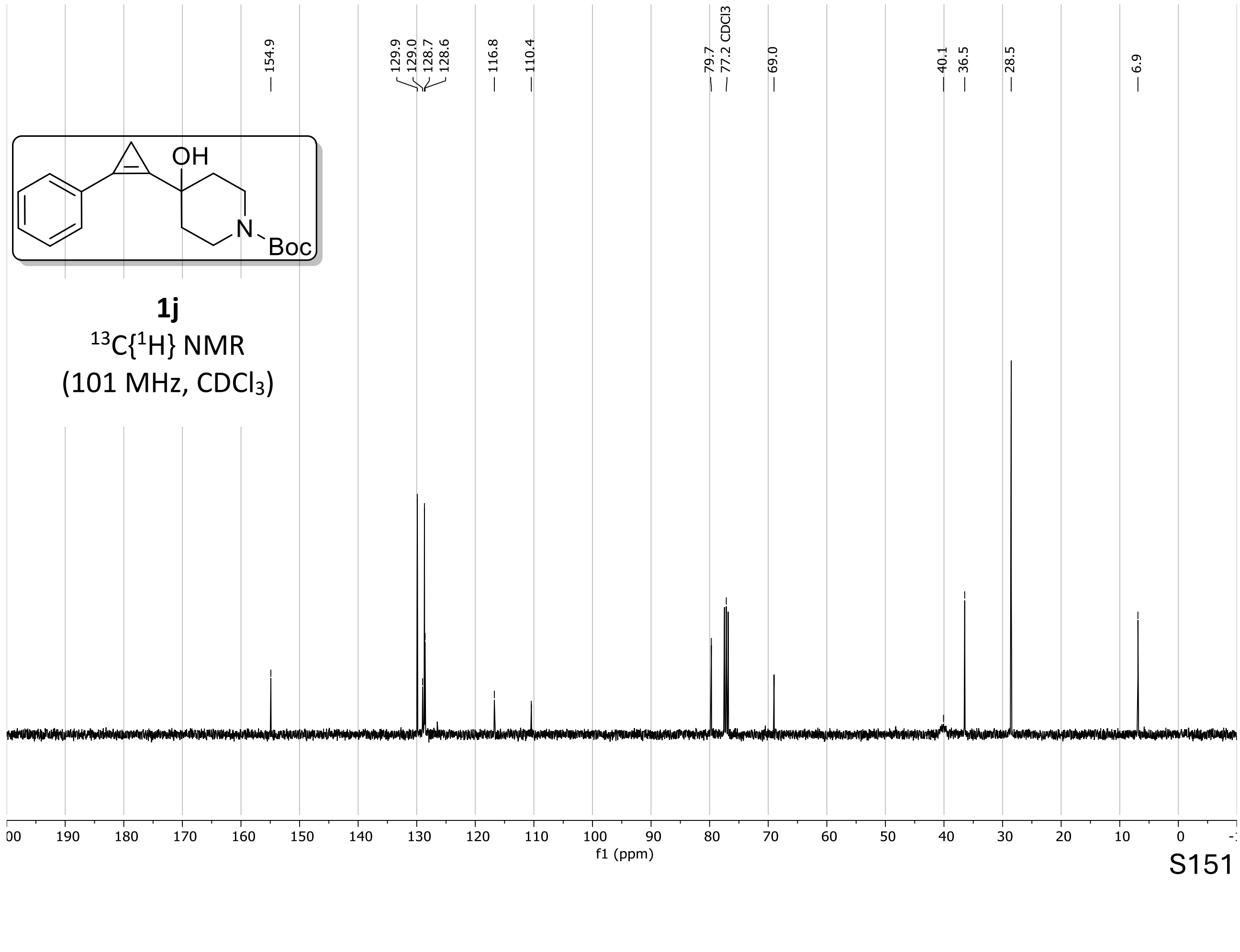

1j
$^{13}C\{^1H\}$ NMR
(101 MHz, $CDCl_3$)

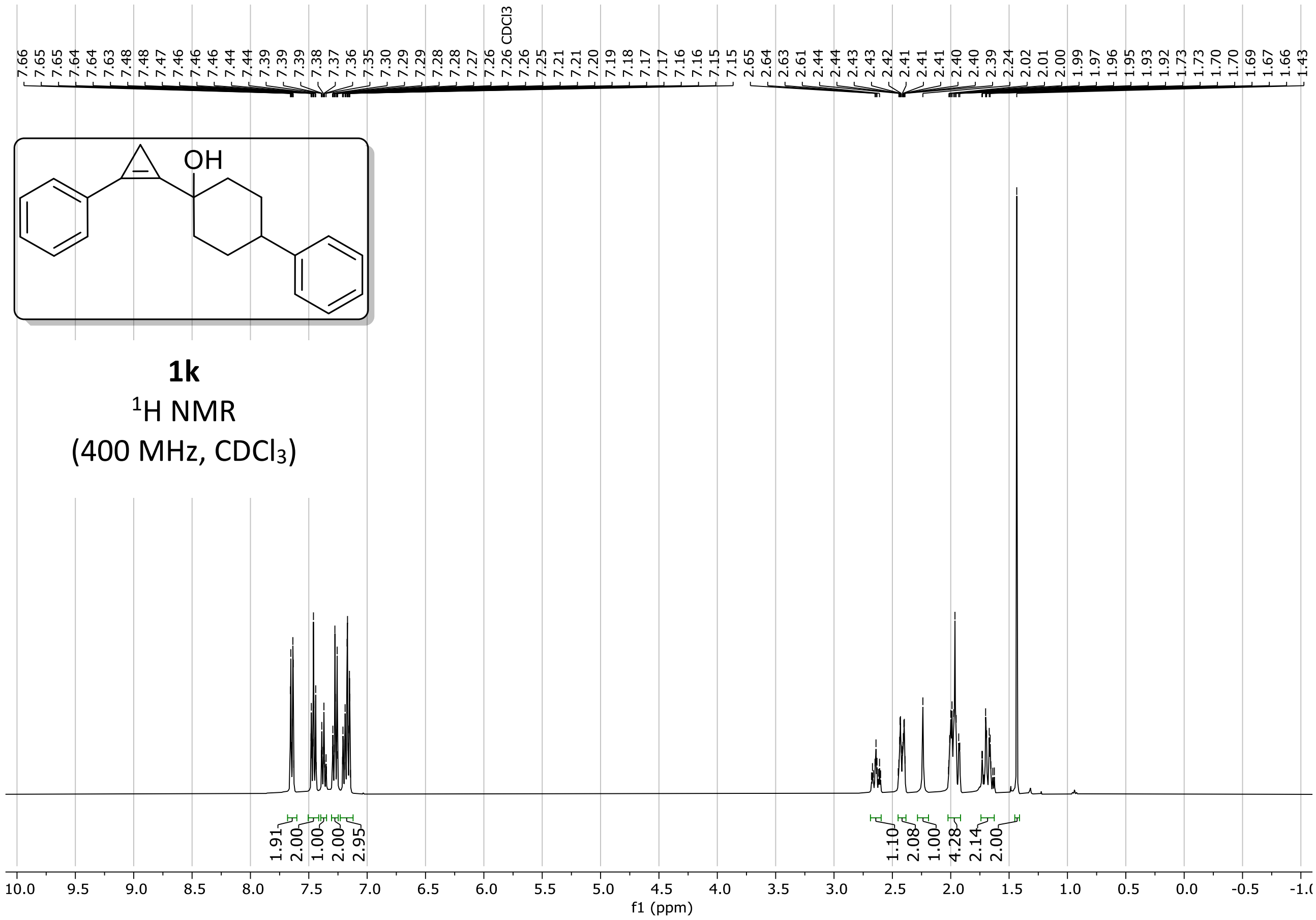
7.66 7.65 7.65 7.64 7.64 7.63 7.48 7.48 7.47 7.46 7.46 7.46 7.44 7.44 7.39 7.39 7.39 7.38 7.37 7.36 7.35 7.30 7.29 7.29 7.28 7.28 7.27 7.26 CDCl3 7.26 7.26 7.25 7.21 7.21 7.20 7.19 7.18 7.17 7.17 7.16 7.16 7.15 7.15
2.65 2.64 2.63 2.61 2.61 2.44 2.44 2.43 2.43 2.42 2.41 2.41 2.41 2.40 2.40 2.39 2.24 2.02 2.01 2.00 1.99 1.97 1.96 1.95 1.93 1.92 1.73 1.73 1.70 1.69 1.67 1.66 1.43
OH
1k
1H NMR
(400 MHz, CDCl3)
1.91 2.00 1.00 2.00 2.95
1.10 2.08 1.00 4.28 2.14 2.00
f1 (ppm)

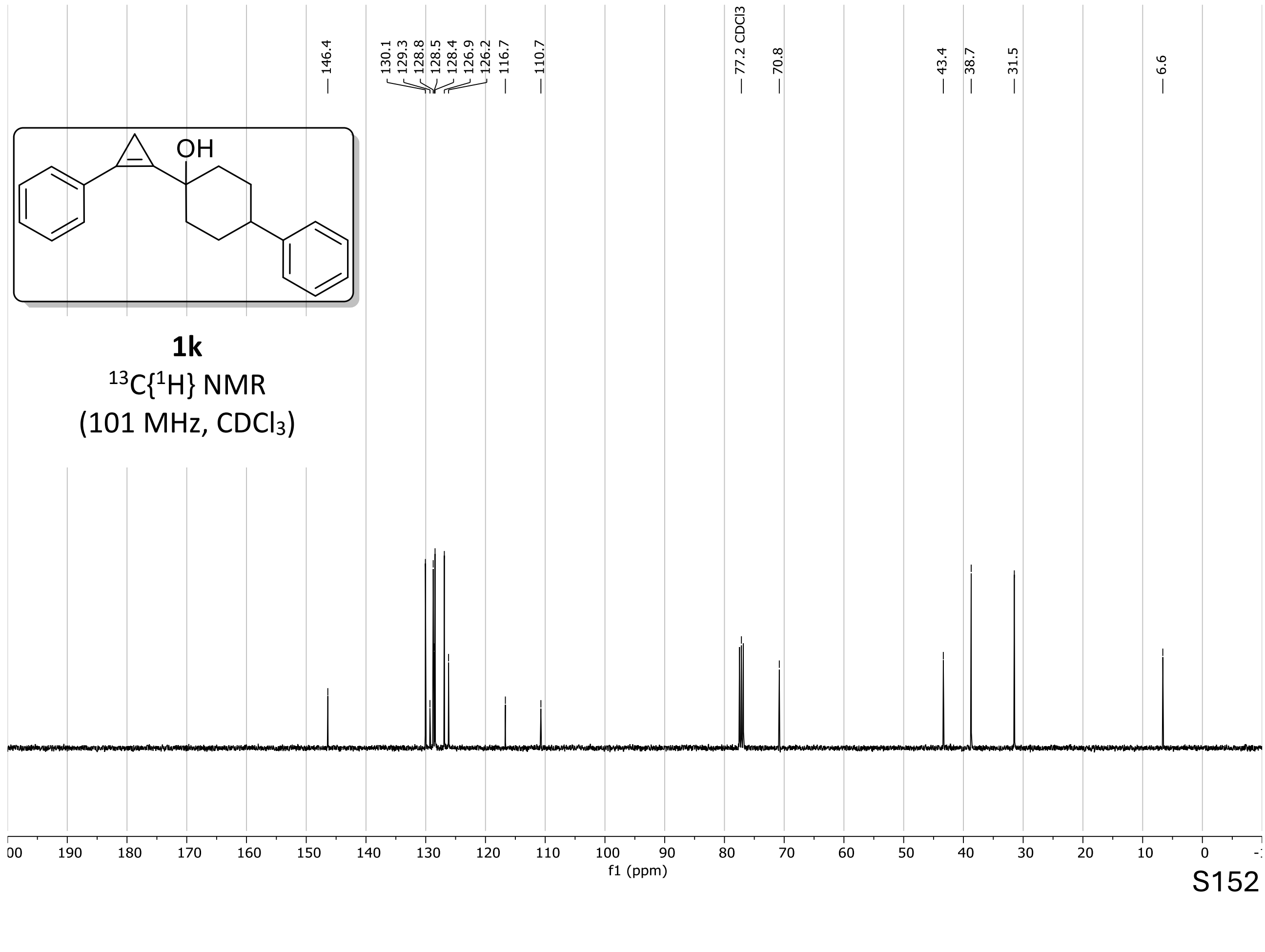
146.4
130.1 129.3 128.8 128.5 128.4 126.9 126.2
116.7
110.7
77.2 CDCl3
70.8
43.4
38.7
31.5
6.6
OH
1k
13C{1H} NMR
(101 MHz, CDCl3)
f1 (ppm)

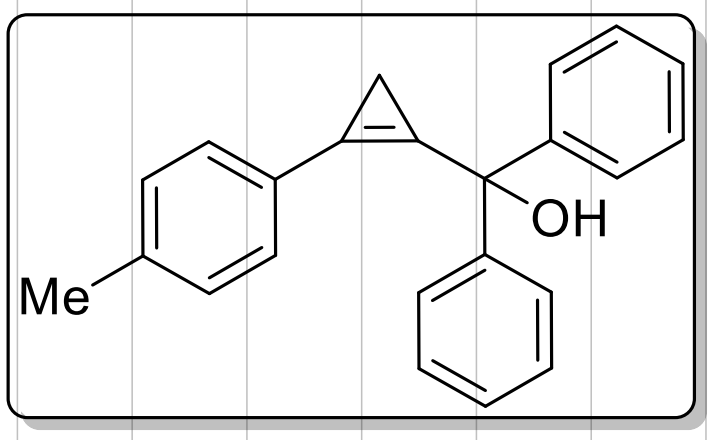

1l

^{1}H NMR

(400 MHz, $CDCl_3$)

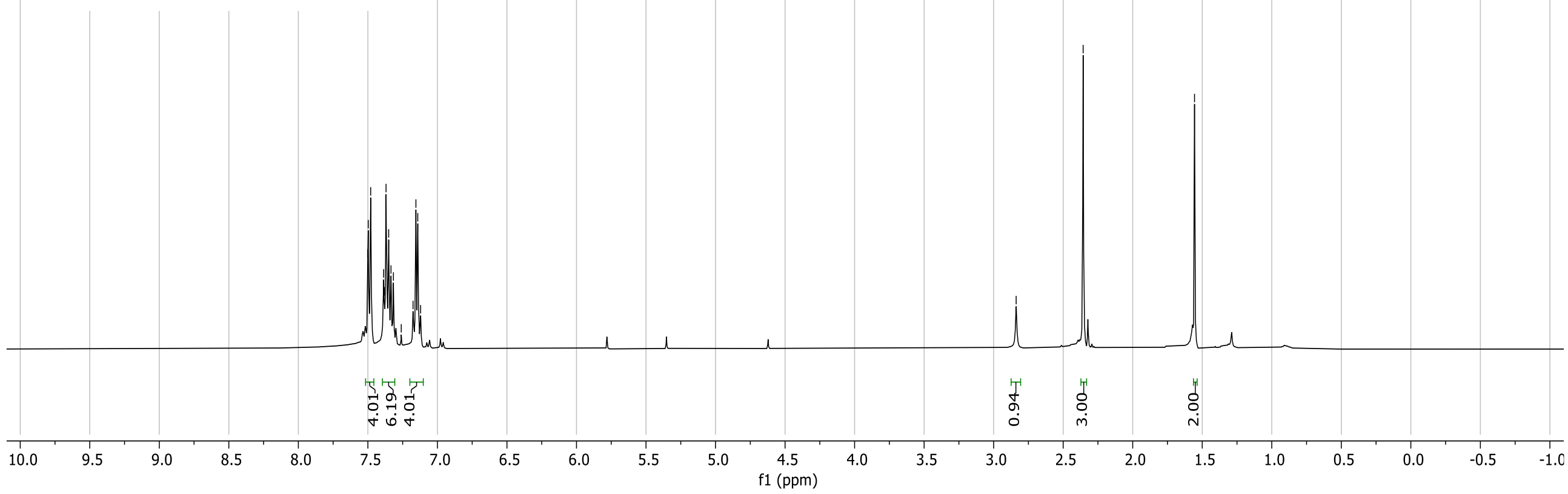

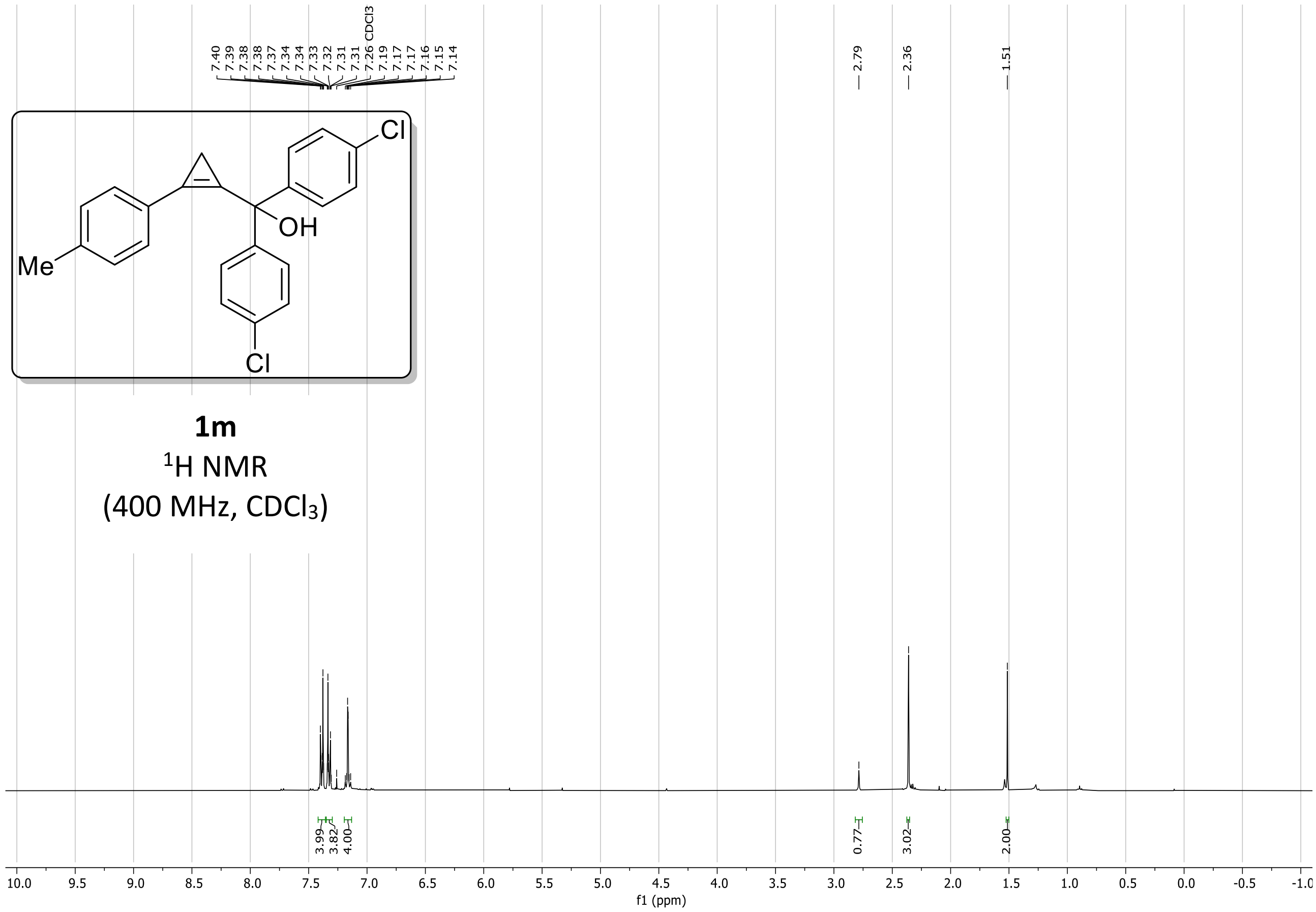

1m

^{1}H NMR

(400 MHz, $CDCl_3$)

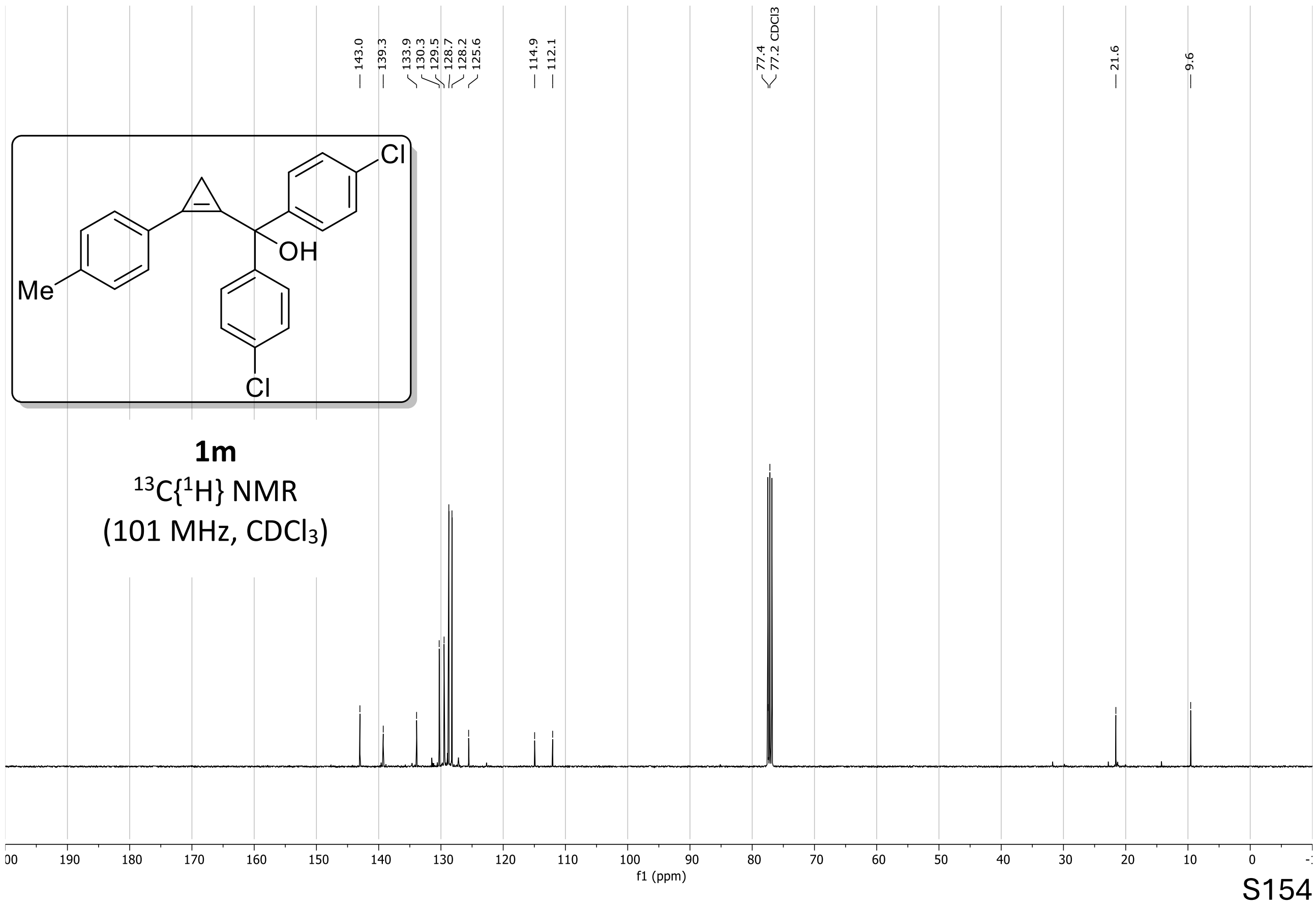

1m

$^{13}C\{^{1}H\}$ NMR

(101 MHz, $CDCl_3$)

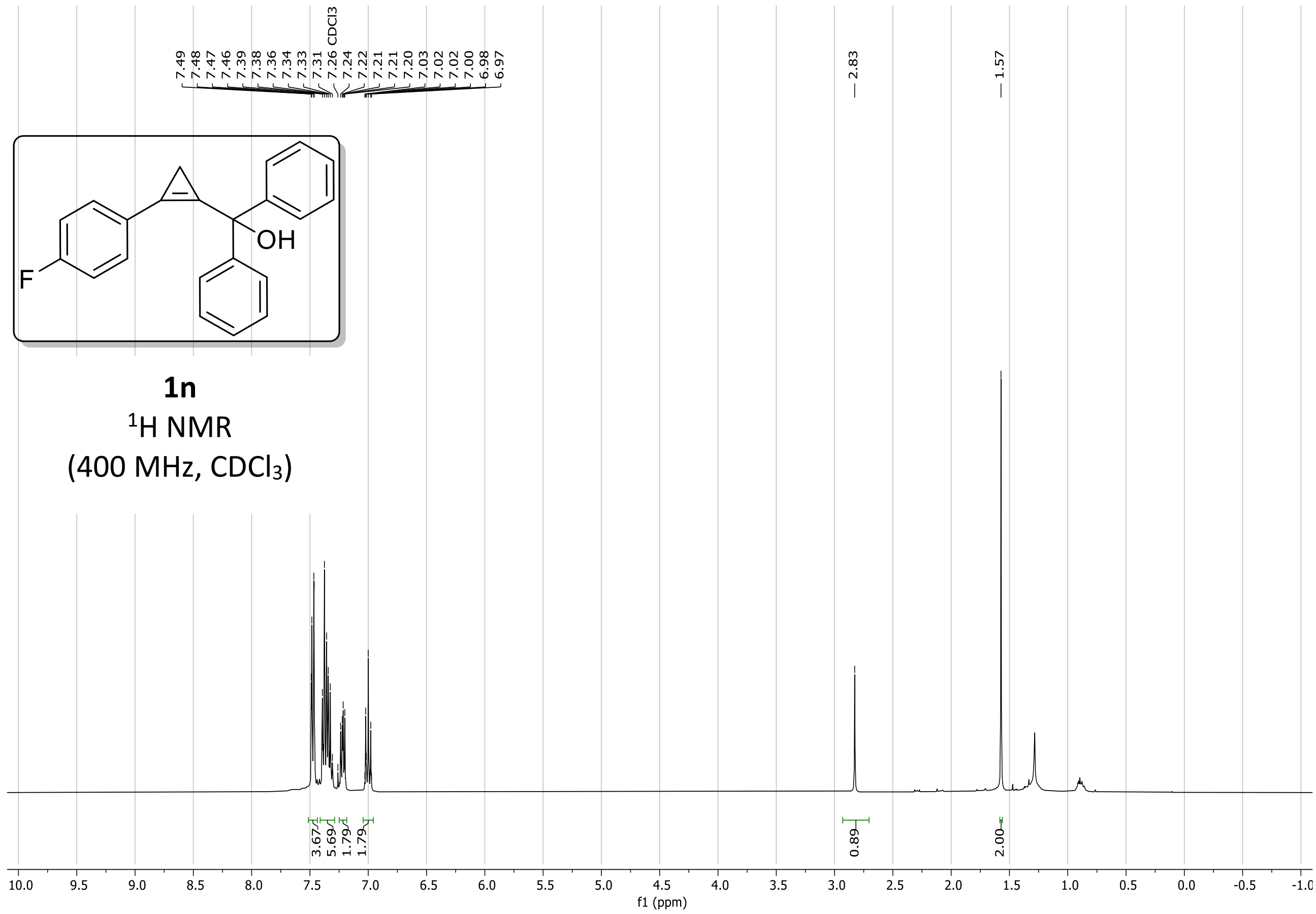

1n

^{1}H NMR

(400 MHz, $CDCl_3$)

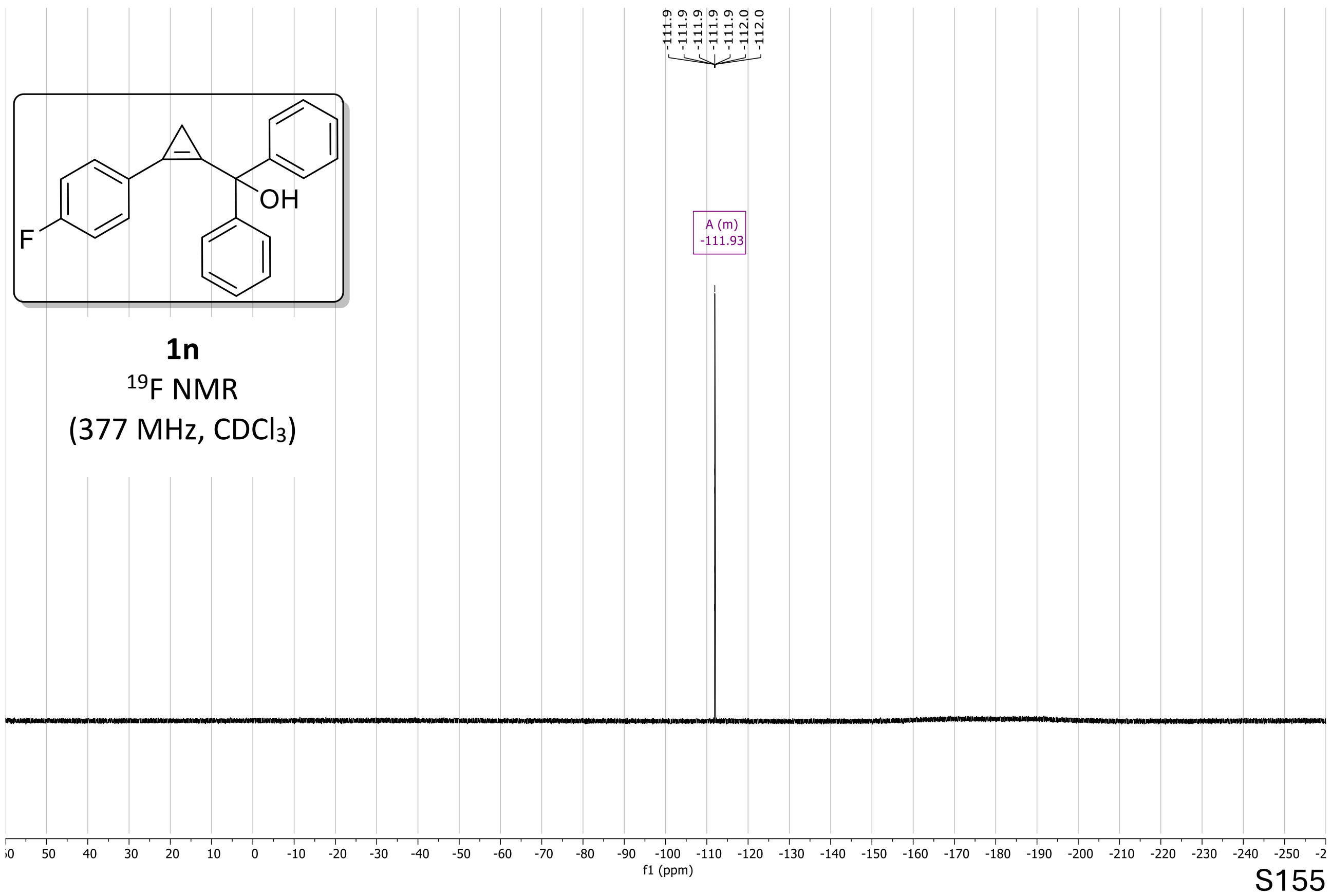

1n

^{19}F NMR

(377 MHz, $CDCl_3$)

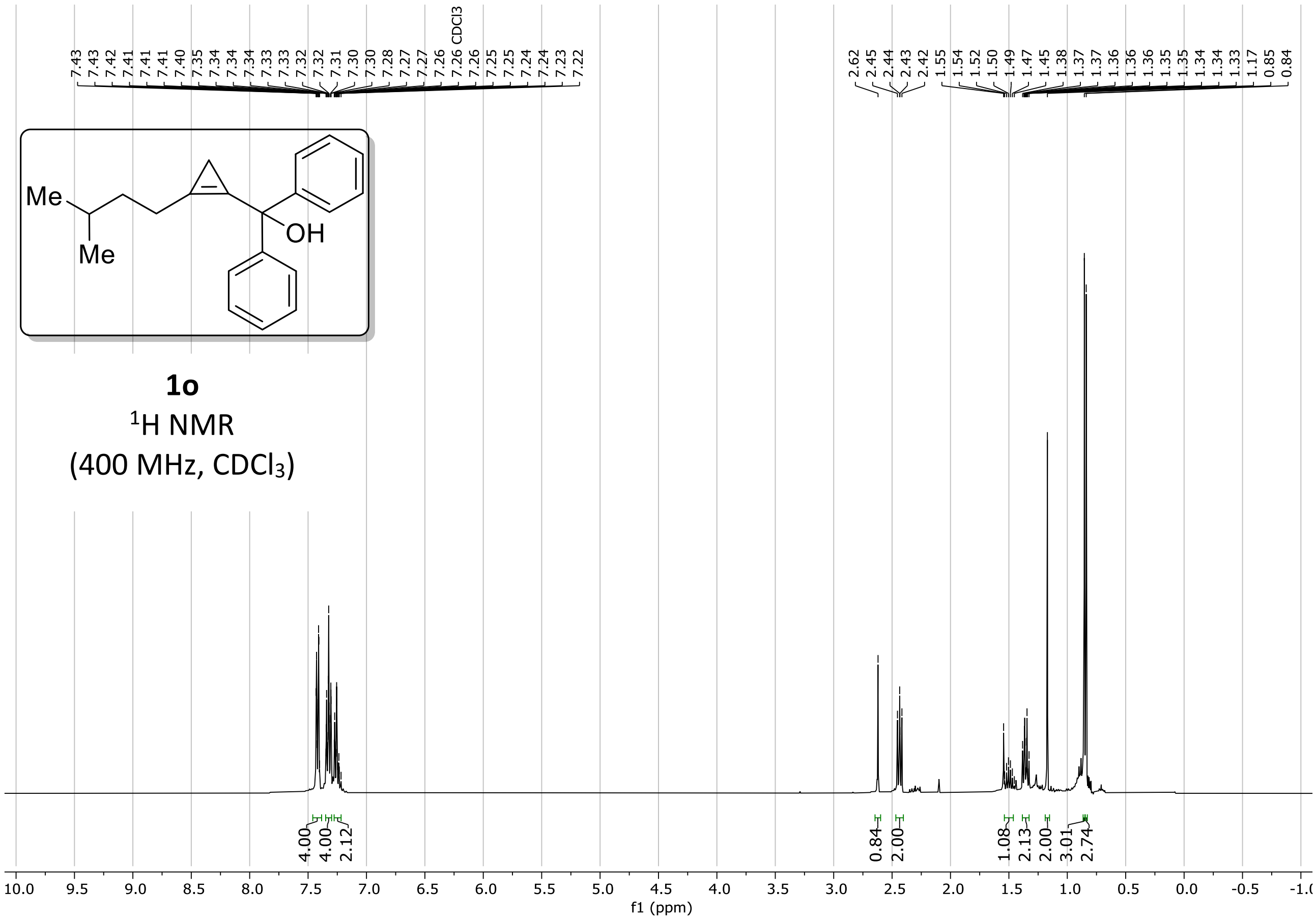

1o
^{1}H NMR
(400 MHz, $CDCl_3$)

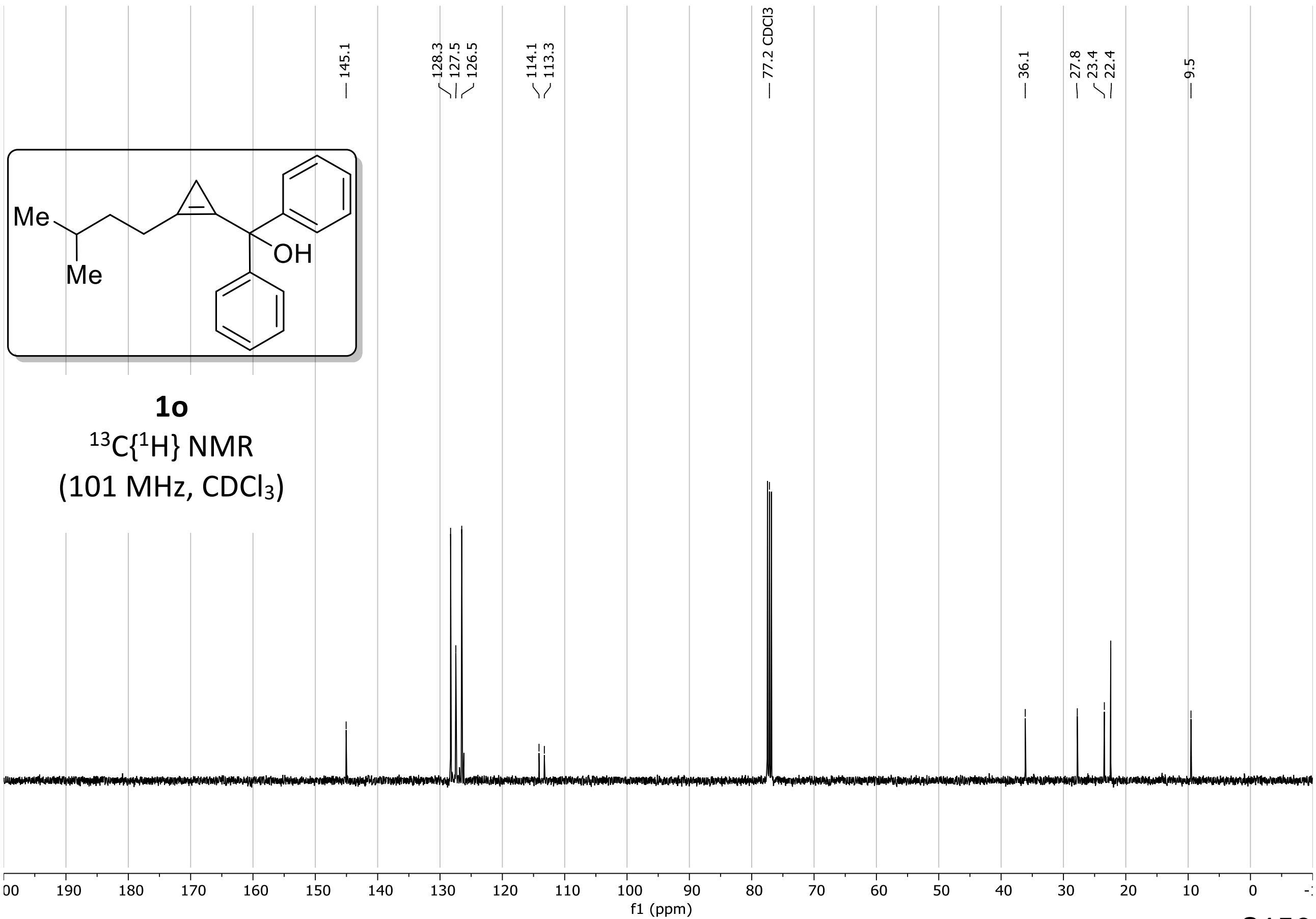

1o
$^{13}C\{^1H\}$ NMR
(101 MHz, $CDCl_3$)

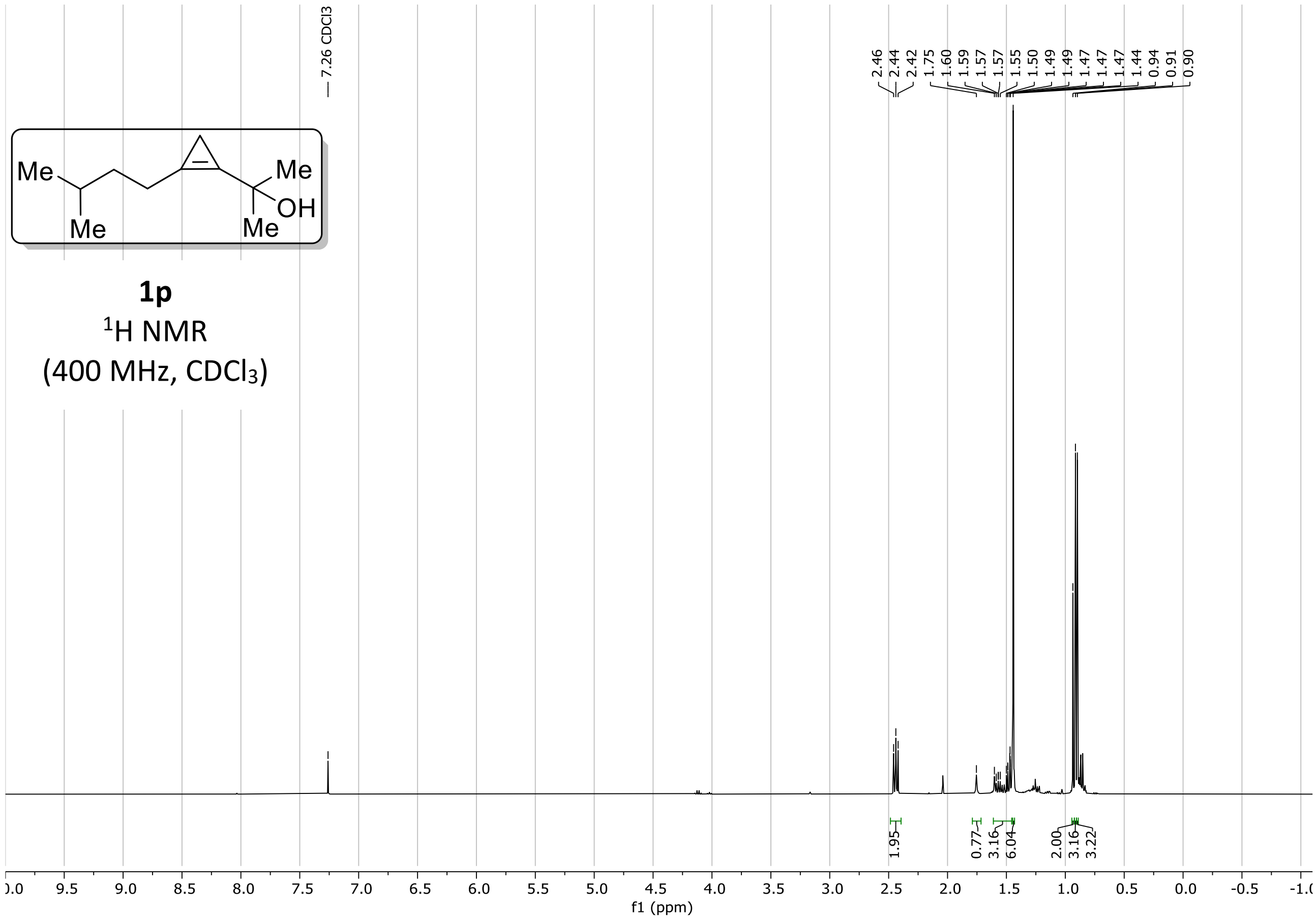
Me
Me
Me
OH
Me
1p
^{1}H NMR
(400 MHz, $CDCl_3$)
7.26 CDCl3
2.46
2.44
2.42
1.75
1.60
1.59
1.57
1.57
1.55
1.50
1.49
1.49
1.47
1.47
1.47
1.44
0.94
0.91
0.90
1.95
0.77
3.16
6.04
2.00
3.16
3.22
f1 (ppm)

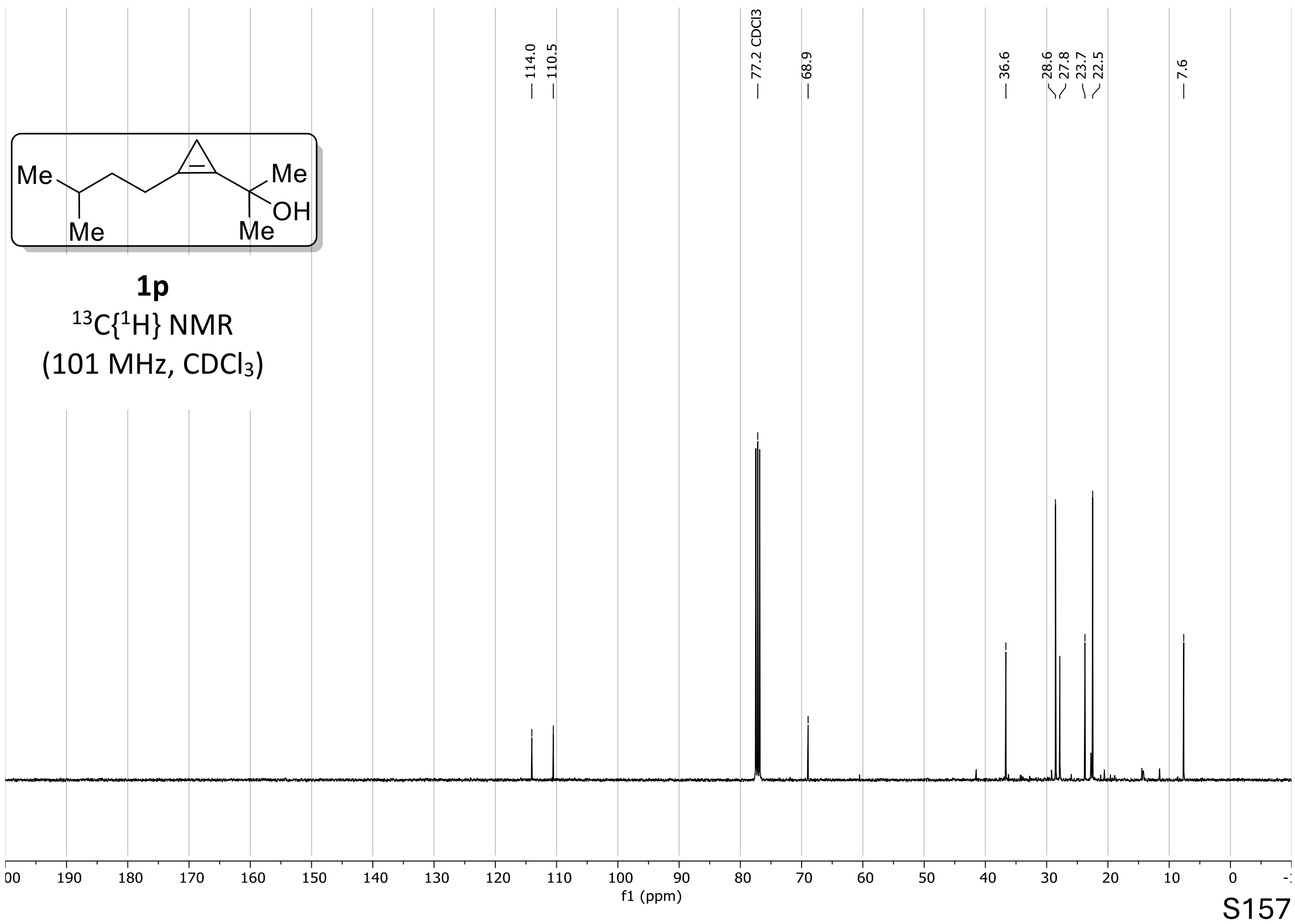
Me
Me
Me
OH
Me
1p
$^{13}C\{^1H\}$ NMR
(101 MHz, $CDCl_3$)
114.0
110.5
77.2 CDCl3
68.9
36.6
28.6
27.8
23.7
22.5
7.6
f1 (ppm)

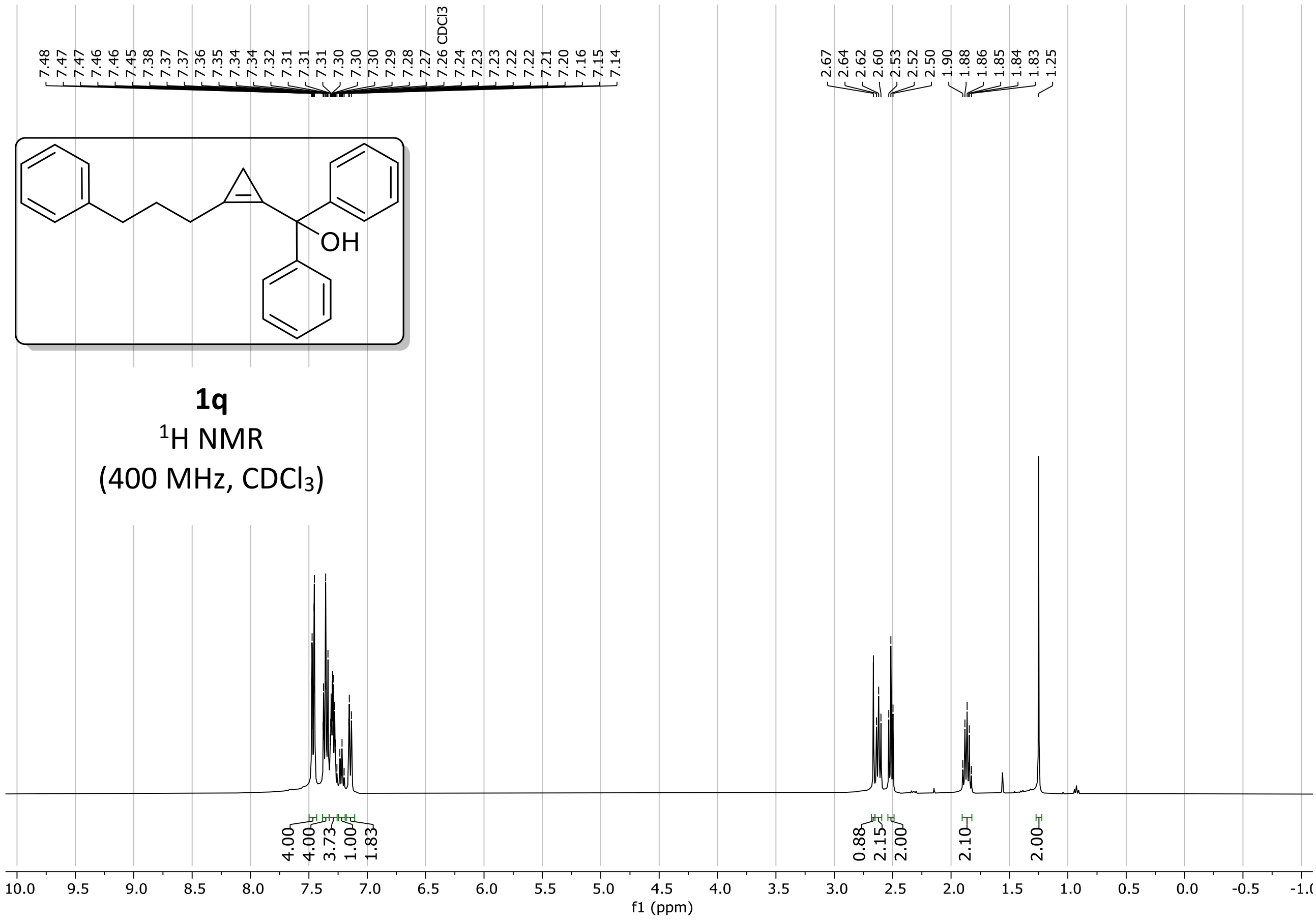
7.48 7.47 7.47 7.46 7.46 7.45 7.38 7.37 7.37 7.36 7.35 7.34 7.34 7.32 7.31 7.31 7.31 7.30 7.30 7.30 7.29 7.28 7.27 7.26 CDCl3 7.24 7.23 7.23 7.22 7.22 7.22 7.21 7.20 7.16 7.15 7.14
2.67 2.64 2.62 2.60 2.53 2.52 2.50 1.90 1.88 1.86 1.85 1.84 1.83 1.25
OH
1q
1H NMR
(400 MHz, CDCl3)
4.00 4.00 3.73 1.00 1.83
0.88 2.15 2.00
2.10
2.00
f1 (ppm)

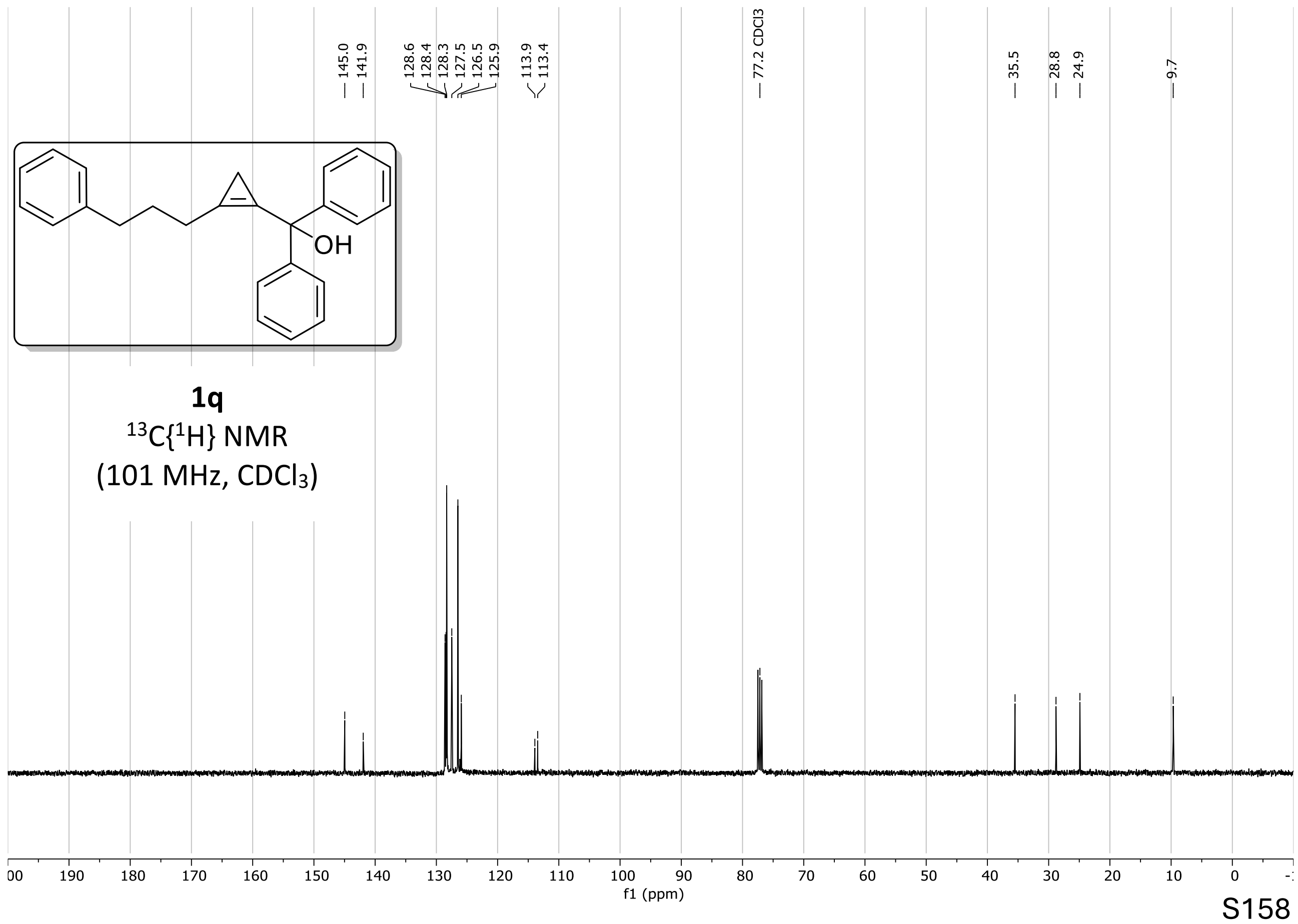
145.0 141.9
128.6 128.4 128.3 127.5 126.5 125.9
113.9 113.4
77.2 CDCl3
35.5 28.8 24.9
9.7
OH
1q
13C{1H} NMR
(101 MHz, CDCl3)
f1 (ppm)

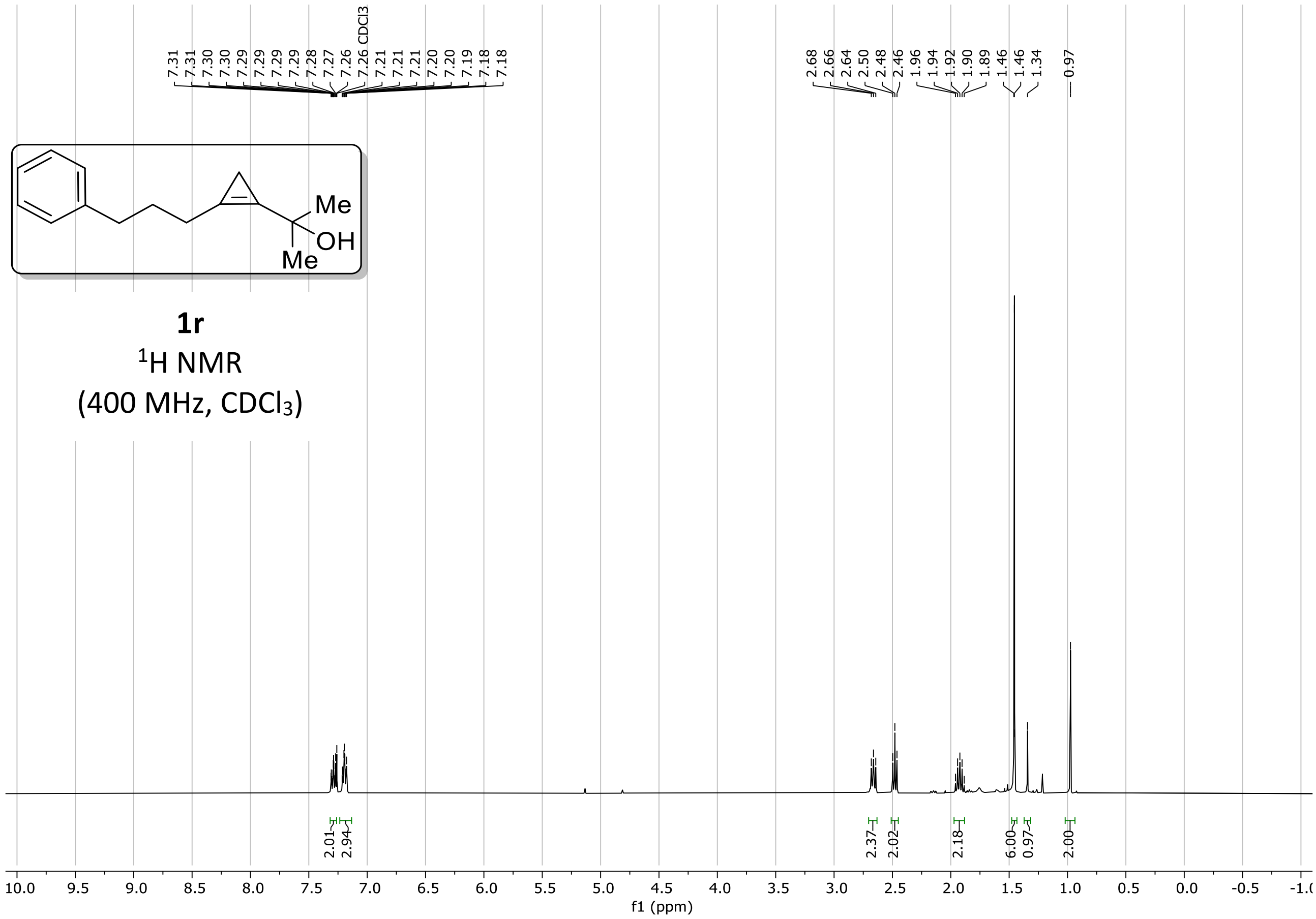
7.31 7.31 7.30 7.29 7.29 7.29 7.28 7.27 7.26 7.26 CDCl3 7.21 7.21 7.21 7.21 7.20 7.20 7.19 7.18 7.18
2.68 2.66 2.64 2.50 2.48 2.46 1.96 1.94 1.92 1.90 1.89 1.46 1.46 1.34
0.97
Me
OH
Me
1r
1H NMR
(400 MHz, CDCl3)
2.01 2.94 2.37 2.02 2.18 6.00 0.97 2.00
f1 (ppm)

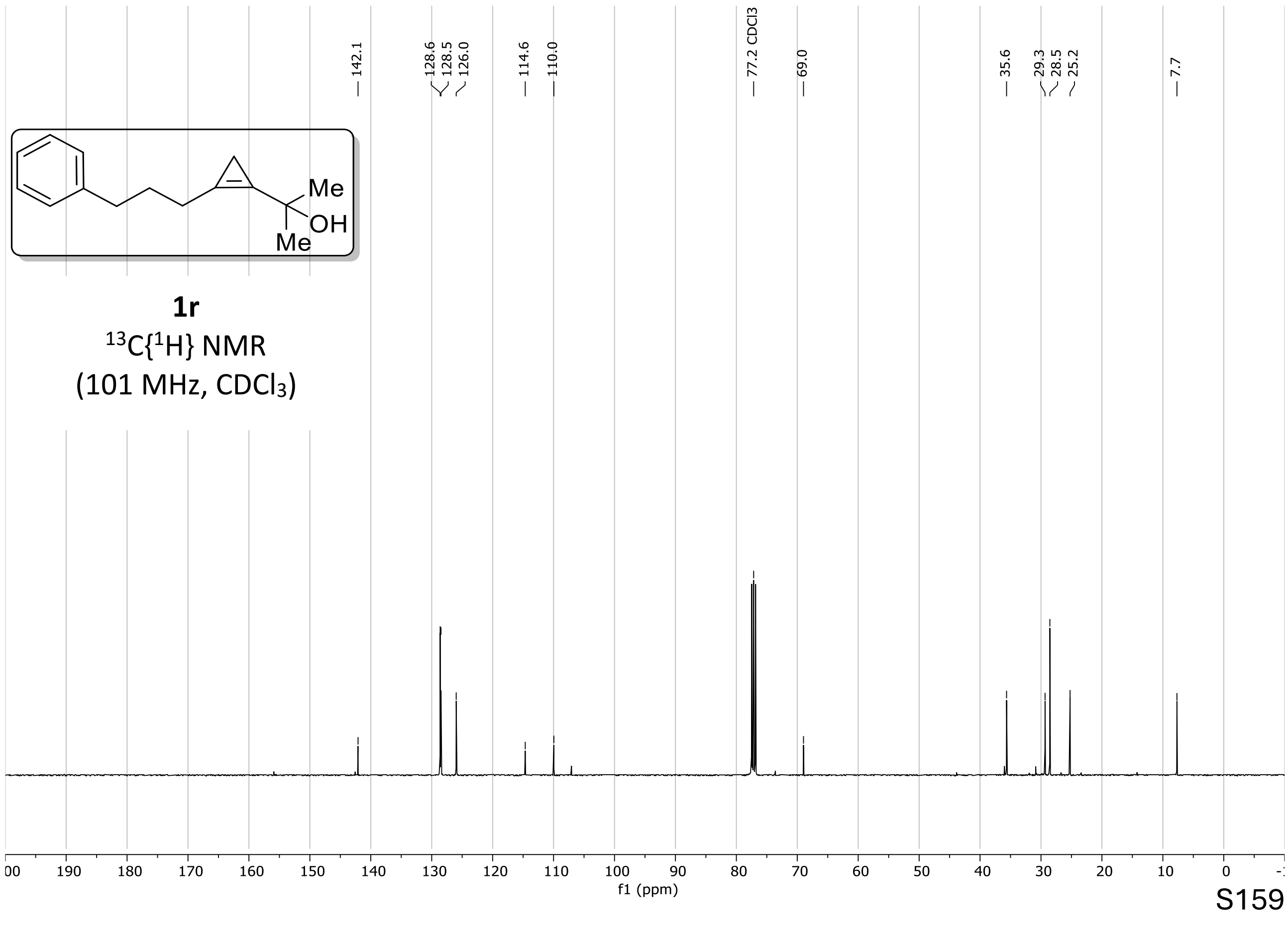
142.1
128.6 128.5 126.0
114.6 110.0
77.2 CDCl3
69.0
35.6
29.3 28.5 25.2
7.7
Me
OH
Me
1r
13C{1H} NMR
(101 MHz, CDCl3)
f1 (ppm)

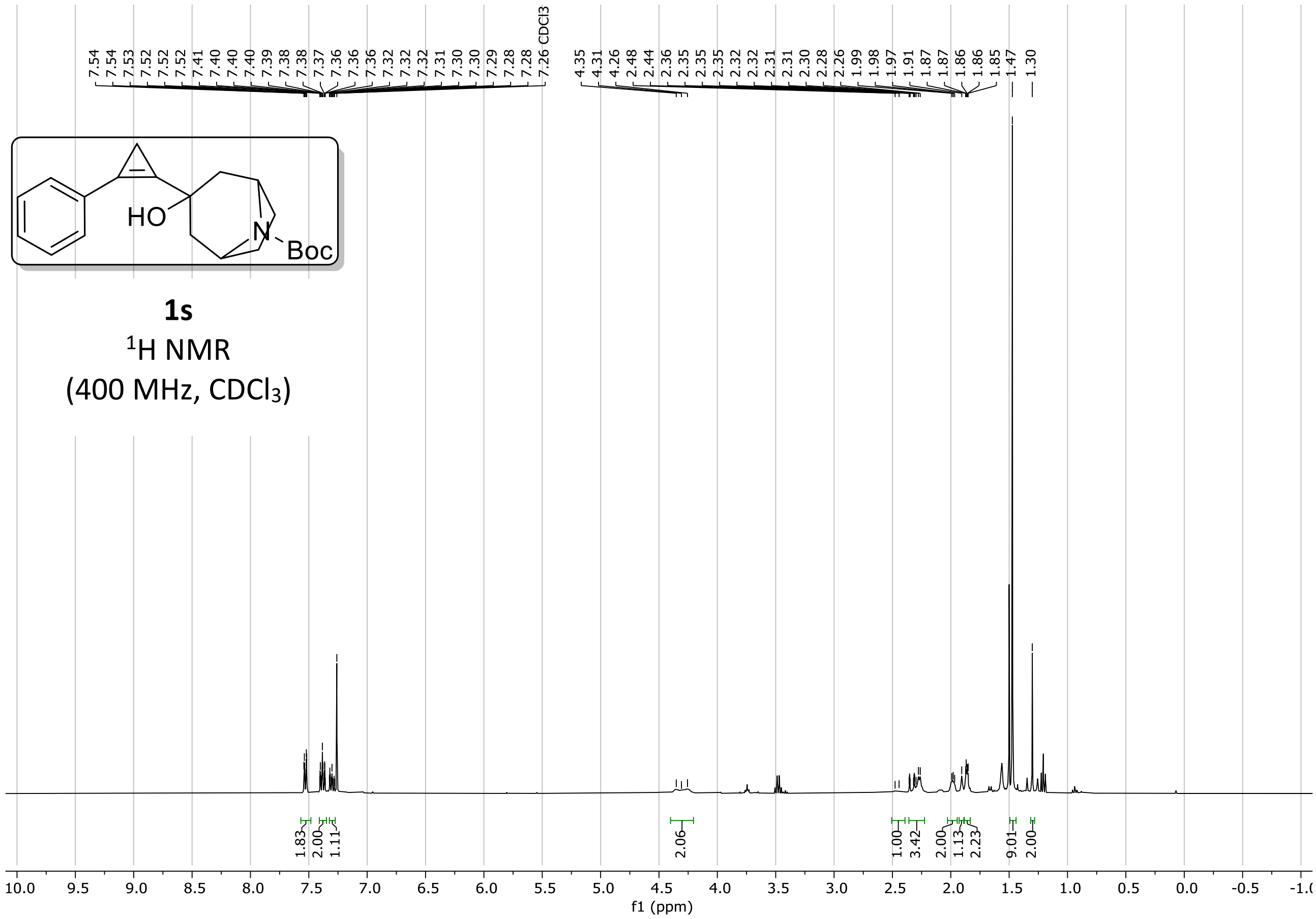
1s
1H NMR
(400 MHz, CDCl3)
HO
N
Boc
f1 (ppm)

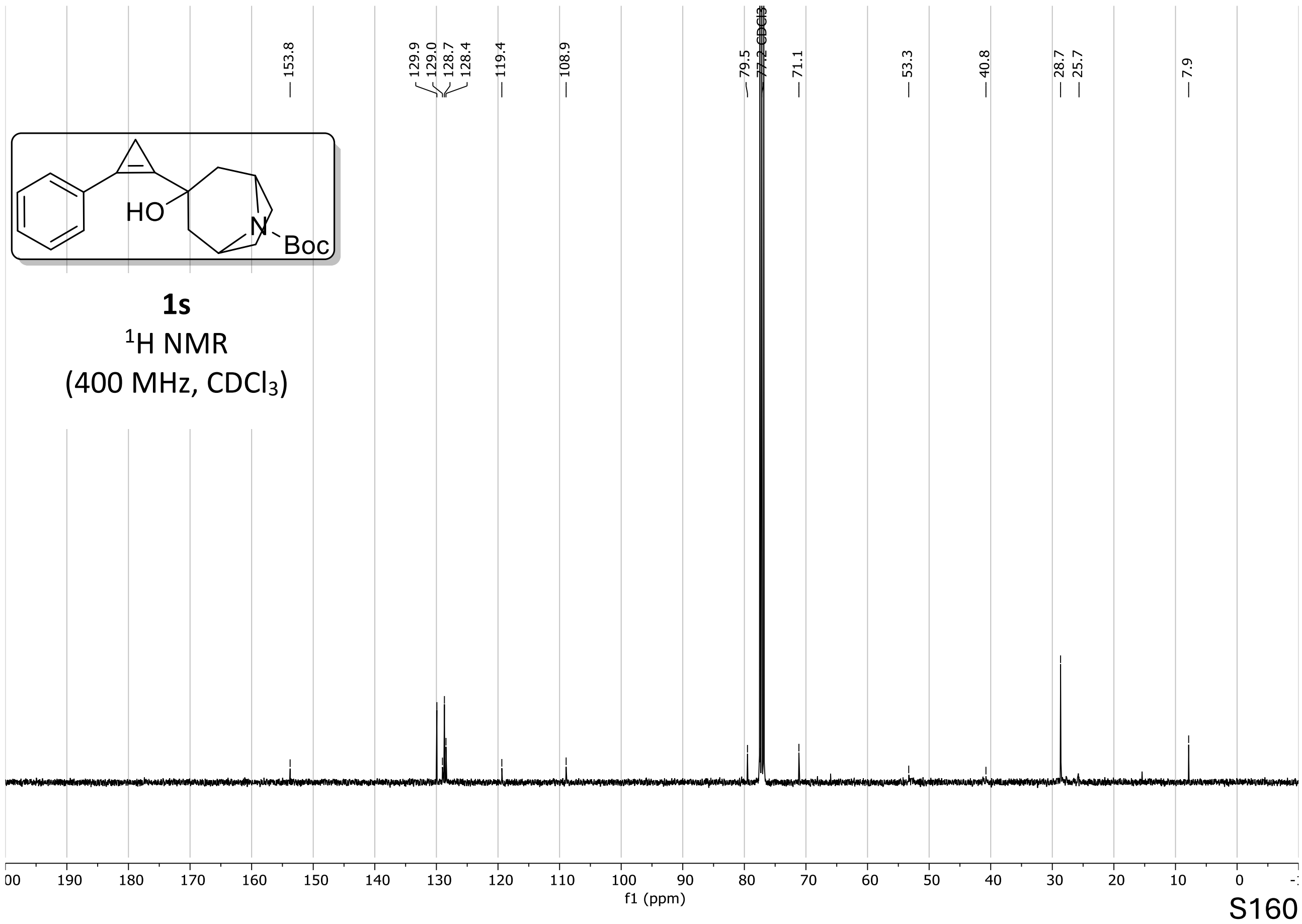
1s
1H NMR
(400 MHz, CDCl3)
HO
N
Boc
f1 (ppm)

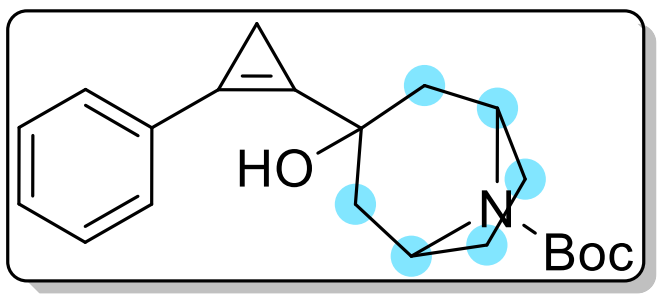

1s - HSQC ($CDCl_3$)
^{1}H NMR (400 MHz)
^{13}C{^{1}H} NMR (101 MHz)

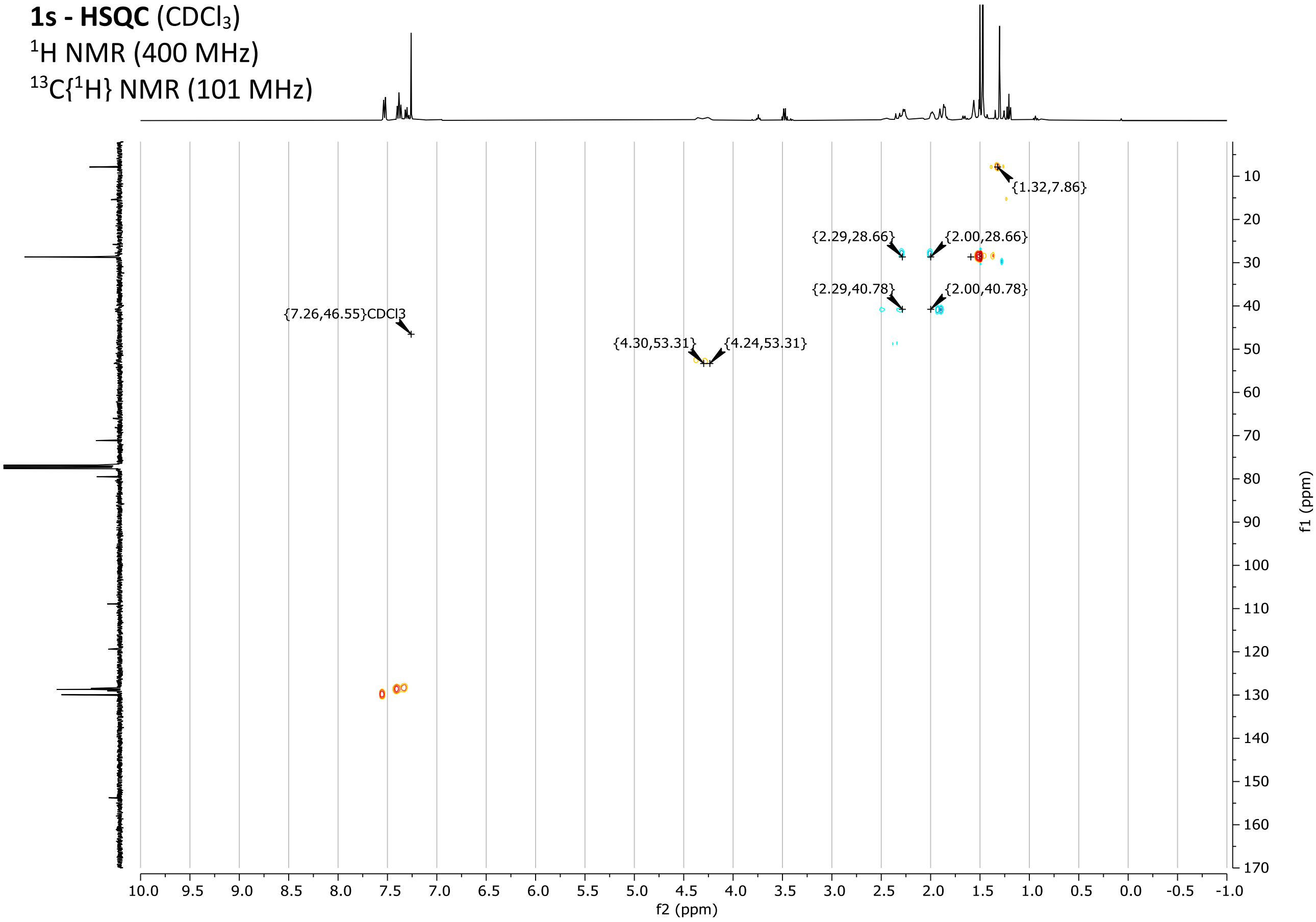

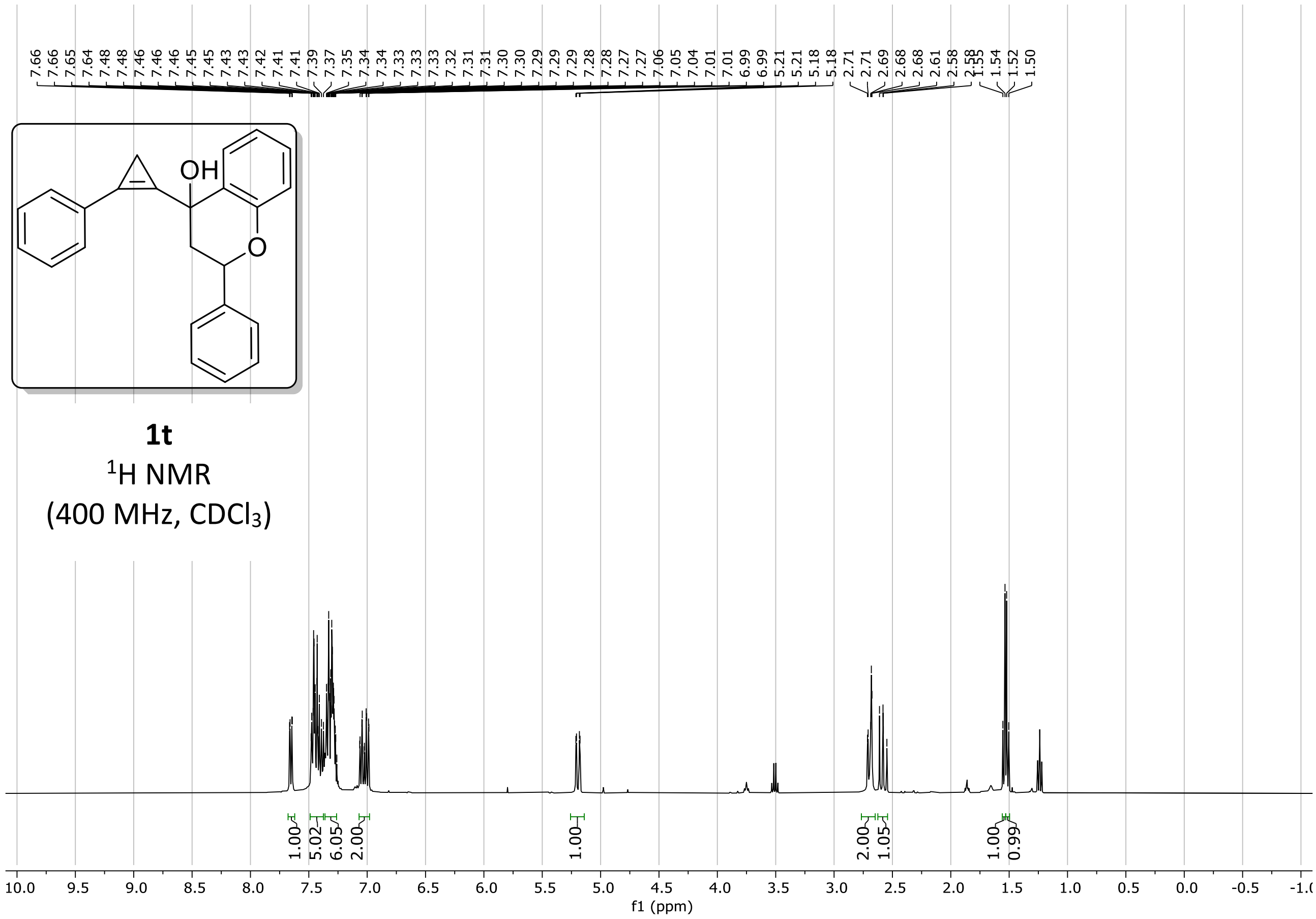
7.66
7.66
7.65
7.64
7.48
7.48
7.46
7.46
7.46
7.45
7.45
7.43
7.43
7.42
7.41
7.41
7.39
7.37
7.35
7.34
7.34
7.33
7.33
7.33
7.32
7.31
7.31
7.30
7.30
7.29
7.29
7.29
7.28
7.28
7.27
7.27
7.06
7.05
7.04
7.01
7.01
6.99
6.99
5.21
5.21
5.18
2.71
2.71
2.69
2.68
2.68
2.61
2.58
2.58
1.55
1.54
1.52
1.50
OH
O
1t
^{1}H NMR
(400 MHz, $CDCl_3$)
1.00
5.02
6.05
2.00
1.00
2.00
1.05
1.00
0.99
f1 (ppm)

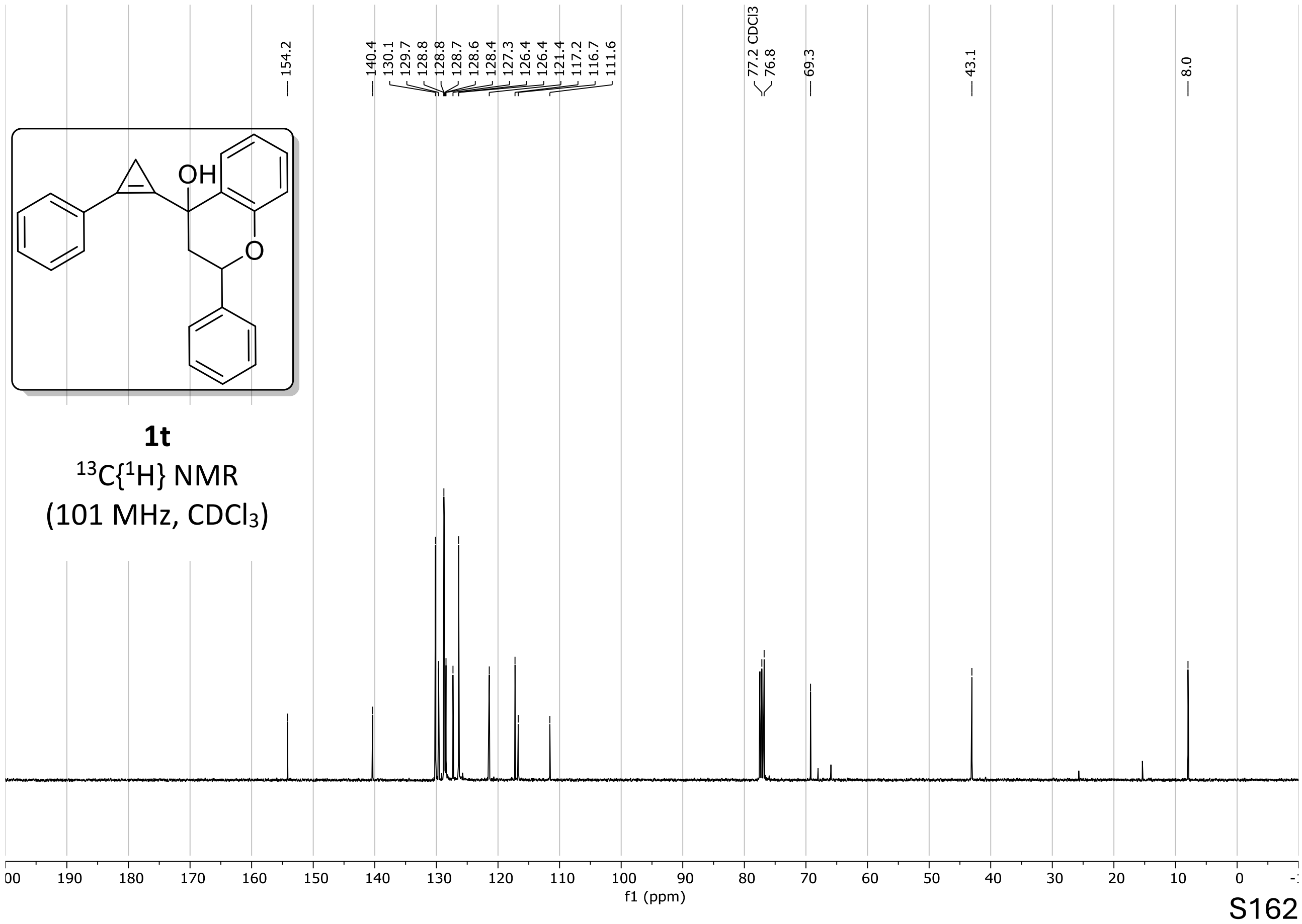
154.2
140.4
130.1
129.7
128.8
128.8
128.7
128.6
128.4
127.3
126.4
126.4
121.4
117.2
116.7
111.6
77.2 CDCl3
76.8
69.3
43.1
8.0
OH
O
1t
$^{13}C\{^1H\}$ NMR
(101 MHz, $CDCl_3$)
f1 (ppm)

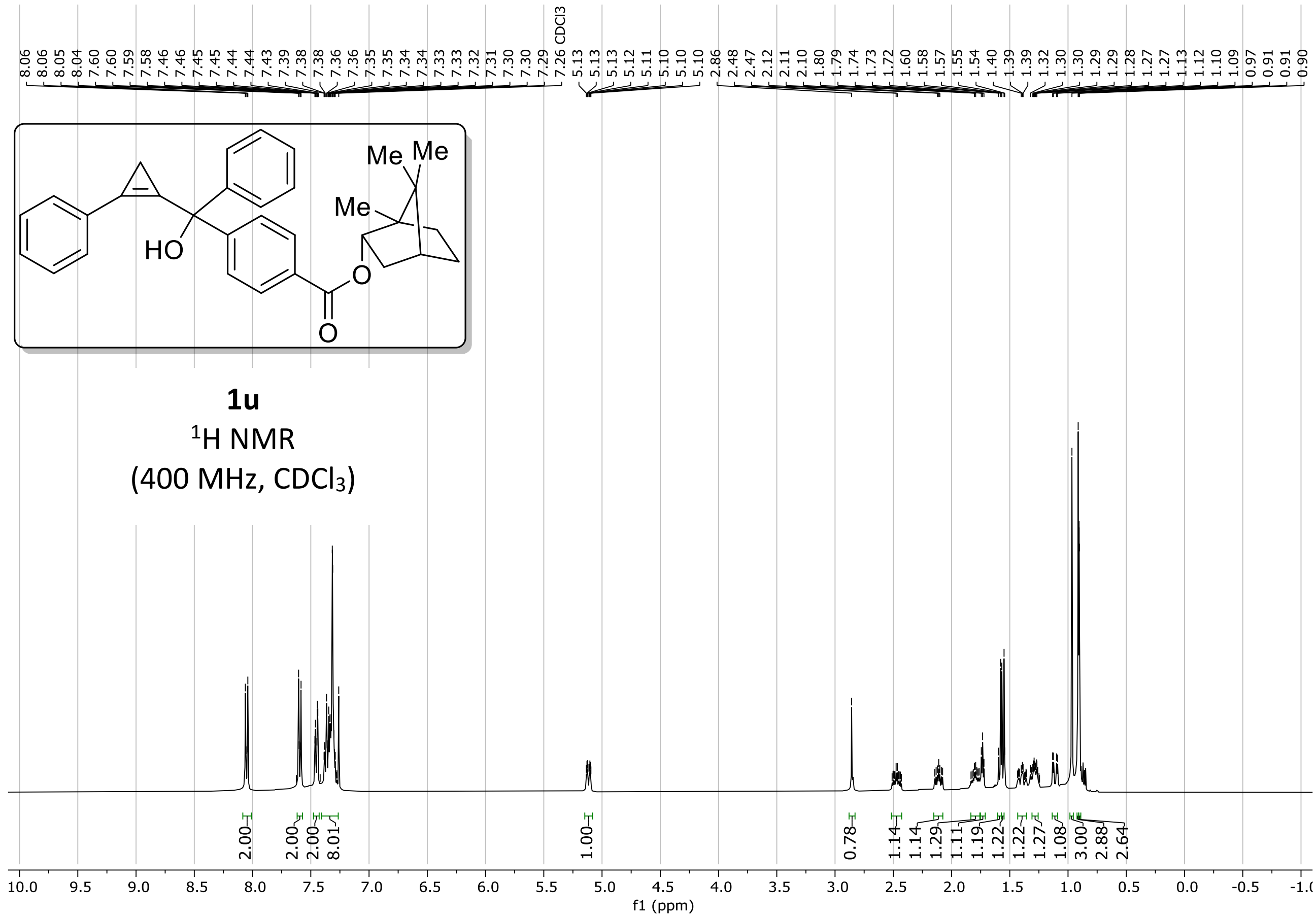
8.06 8.06 8.05 8.04 7.60 7.60 7.59 7.58 7.46 7.46 7.45 7.45 7.44 7.44 7.43 7.39 7.38 7.38 7.36 7.36 7.35 7.35 7.34 7.34 7.33 7.33 7.32 7.31 7.30 7.30 7.29 7.26 CDCl3 5.13 5.13 5.13 5.12 5.11 5.10 5.10 5.10 2.86 2.48 2.47 2.12 2.11 2.10 1.80 1.79 1.74 1.73 1.72 1.60 1.58 1.57 1.55 1.54 1.40 1.39 1.39 1.32 1.30 1.30 1.29 1.29 1.28 1.27 1.27 1.13 1.12 1.10 1.09 0.97 0.91 0.91 0.90
Me
Me
Me
HO
O
O
1u
1H NMR
(400 MHz, CDCl3)
2.00 2.00 2.00 8.01 1.00 0.78 1.14 1.14 1.29 1.11 1.19 1.22 1.22 1.27 1.08 3.00 2.88 2.64
f1 (ppm)

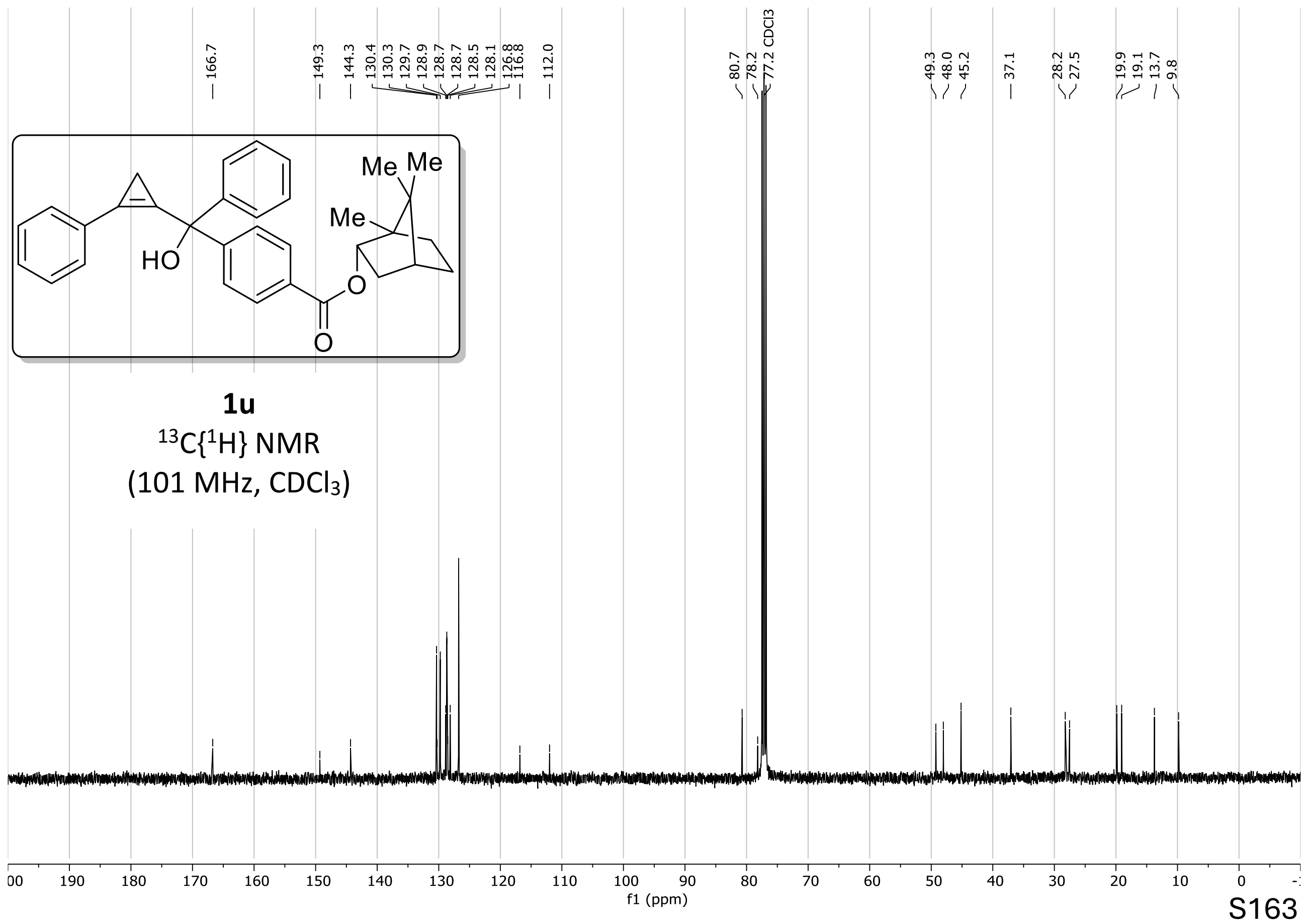
166.7 149.3 144.3 130.4 130.3 129.7 128.9 128.7 128.7 128.5 128.1 126.8 116.8 112.0 80.7 78.2 77.2 CDCl3 49.3 48.0 45.2 37.1 28.2 27.5 19.9 19.1 13.7 9.8
Me
Me
Me
HO
O
O
1u
13C{1H} NMR
(101 MHz, CDCl3)
f1 (ppm)

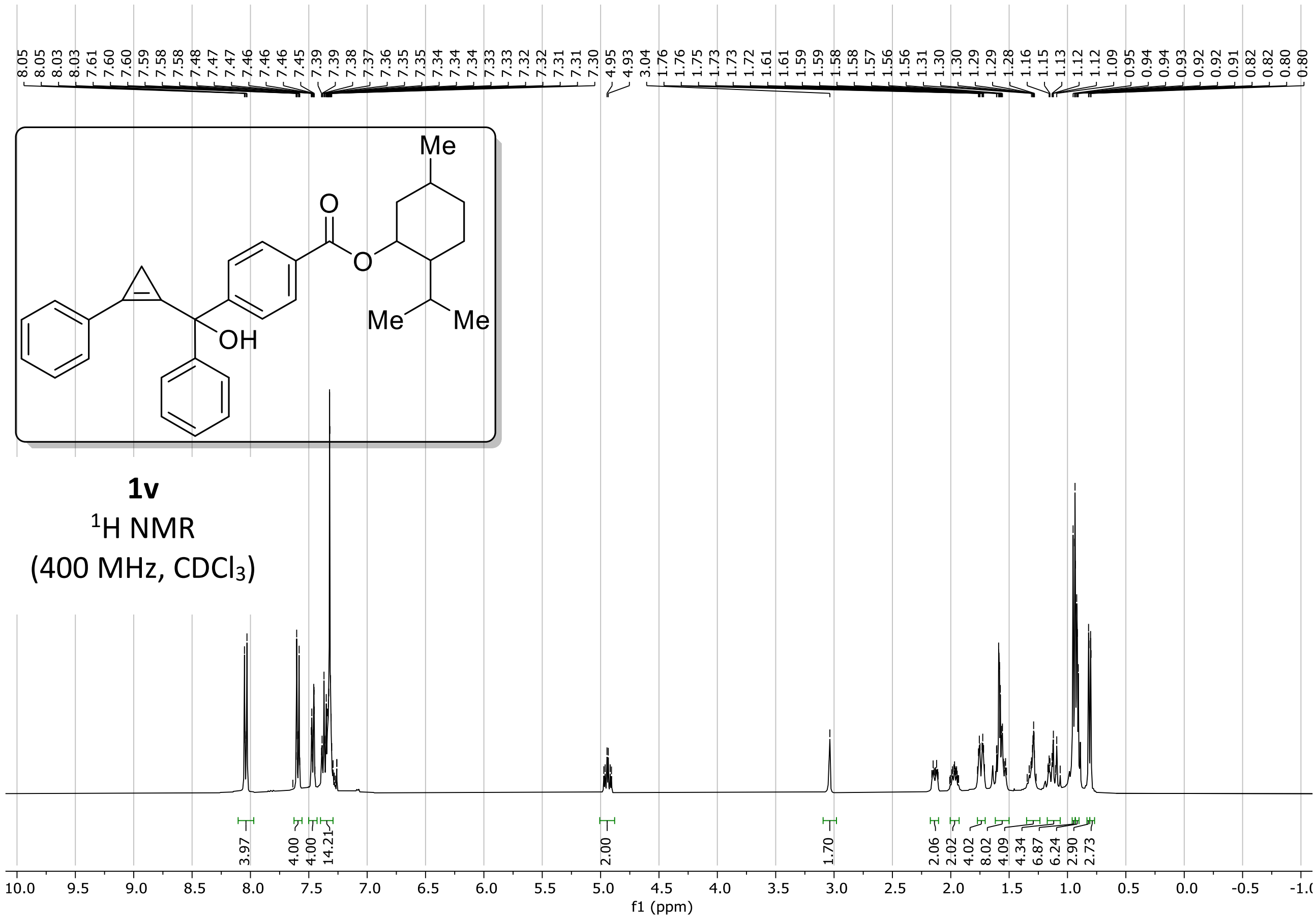
8.05 8.05 8.03 8.03 7.61 7.60 7.59 7.58 7.58 7.48 7.47 7.46 7.46 7.45 7.39 7.39 7.38 7.37 7.36 7.35 7.35 7.34 7.34 7.34 7.33 7.33 7.32 7.32 7.31 7.31 7.30 4.95 4.93 3.04 1.76 1.76 1.75 1.73 1.73 1.72 1.61 1.61 1.59 1.59 1.58 1.58 1.57 1.56 1.31 1.30 1.30 1.29 1.29 1.28 1.16 1.15 1.13 1.12 1.12 1.09 0.95 0.94 0.94 0.93 0.92 0.92 0.91 0.82 0.80 0.80
Me
O
O
OH
Me
Me
1v
^{1}H NMR
(400 MHz, CDCl$_3$)
3.97 4.00 4.00 14.21 2.00 1.70 2.06 2.02 4.02 8.02 4.09 4.34 6.87 6.24 2.90 2.73
10.0 9.5 9.0 8.5 8.0 7.5 7.0 6.5 6.0 5.5 5.0 4.5 4.0 3.5 3.0 2.5 2.0 1.5 1.0 0.5 0.0 -0.5 -1.0
f1 (ppm)

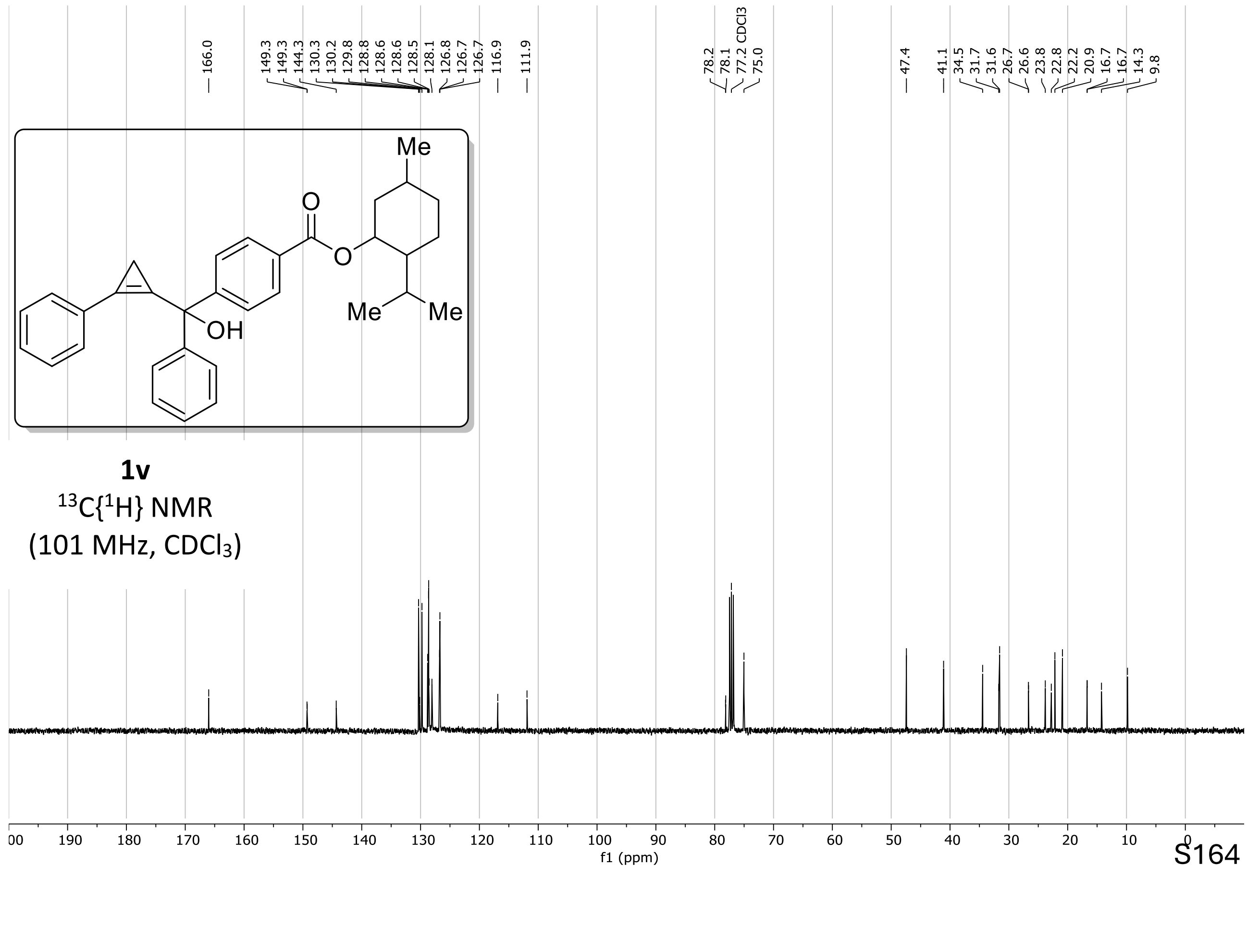
166.0 149.3 149.3 144.3 130.3 130.2 129.8 128.8 128.6 128.6 128.5 128.1 126.8 126.7 116.9 111.9 78.2 78.1 77.2 CDCl3 75.0 47.4 41.1 34.5 31.7 31.6 26.7 26.6 23.8 22.8 22.2 20.9 16.7 16.7 14.3 9.8
Me
O
O
OH
Me
Me
1v
^{13}C{^{1}H} NMR
(101 MHz, CDCl$_3$)
00 190 180 170 160 150 140 130 120 110 100 90 80 70 60 50 40 30 20 10 0
f1 (ppm)

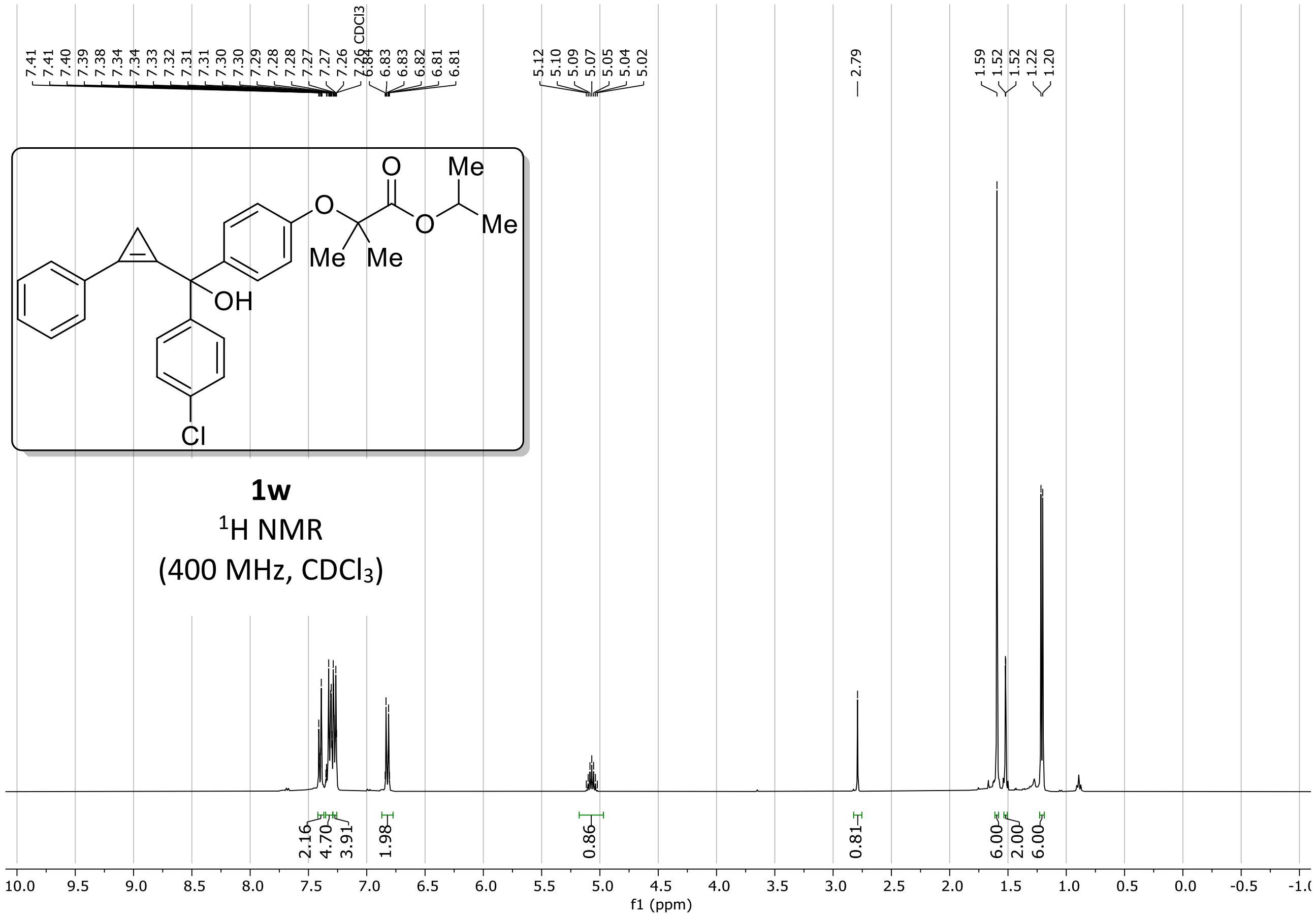
7.41 7.41 7.40 7.39 7.38 7.34 7.34 7.33 7.32 7.31 7.31 7.30 7.30 7.29 7.28 7.28 7.27 7.27 7.26 7.26 CDCl3 6.84 6.83 6.83 6.82 6.81 6.81
5.12 5.10 5.09 5.07 5.05 5.04 5.02
2.79
1.59 1.52 1.52 1.22 1.20
O
Me
O
O
Me
Me
Me
OH
Cl
1w
1H NMR
(400 MHz, CDCl3)
2.16 4.70 3.91 1.98 0.86 0.81 6.00 2.00 6.00
10.0 9.5 9.0 8.5 8.0 7.5 7.0 6.5 6.0 5.5 5.0 4.5 4.0 3.5 3.0 2.5 2.0 1.5 1.0 0.5 0.0 -0.5 -1.0
f1 (ppm)

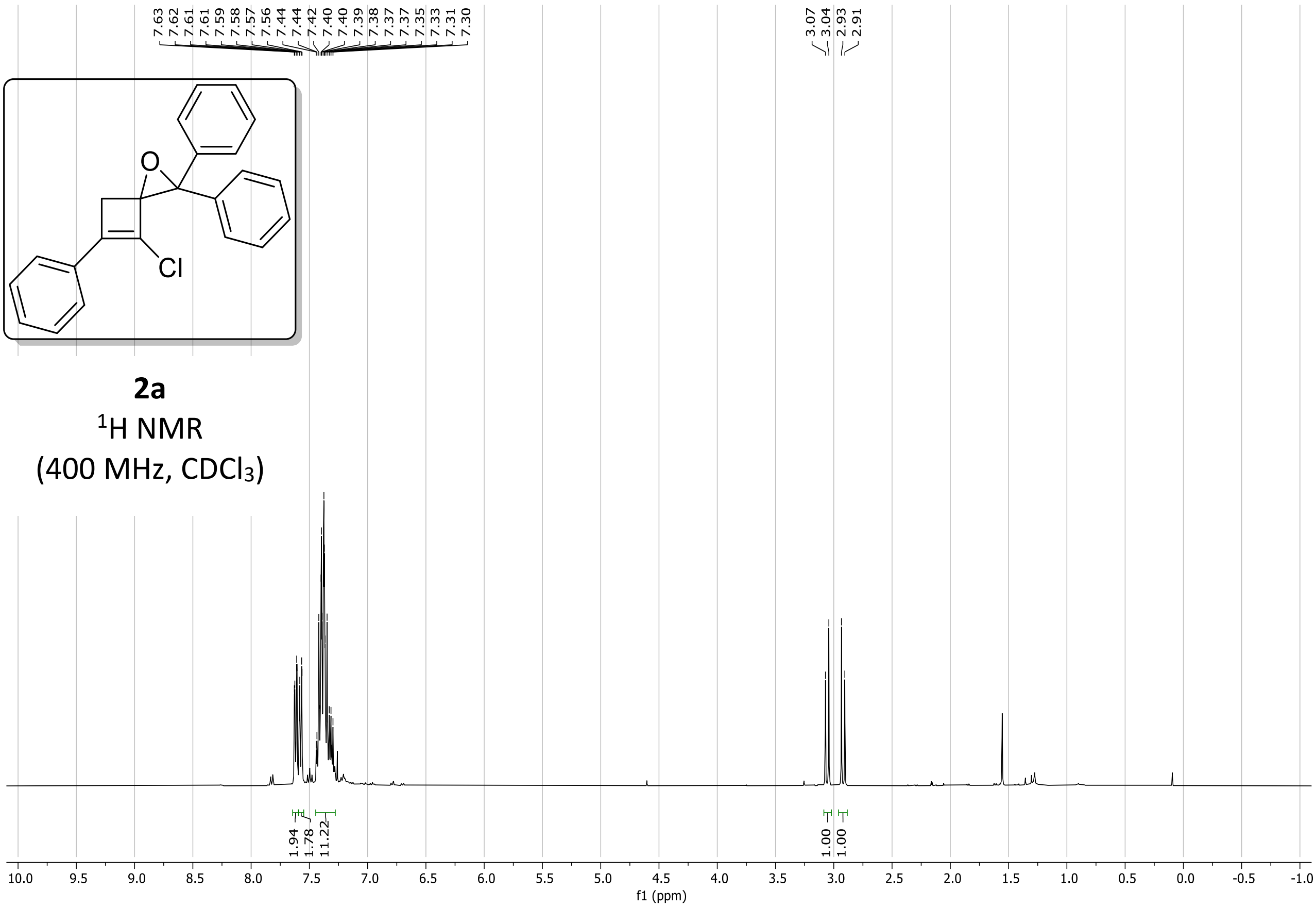

2a
^{1}H NMR
(400 MHz, $CDCl_3$)

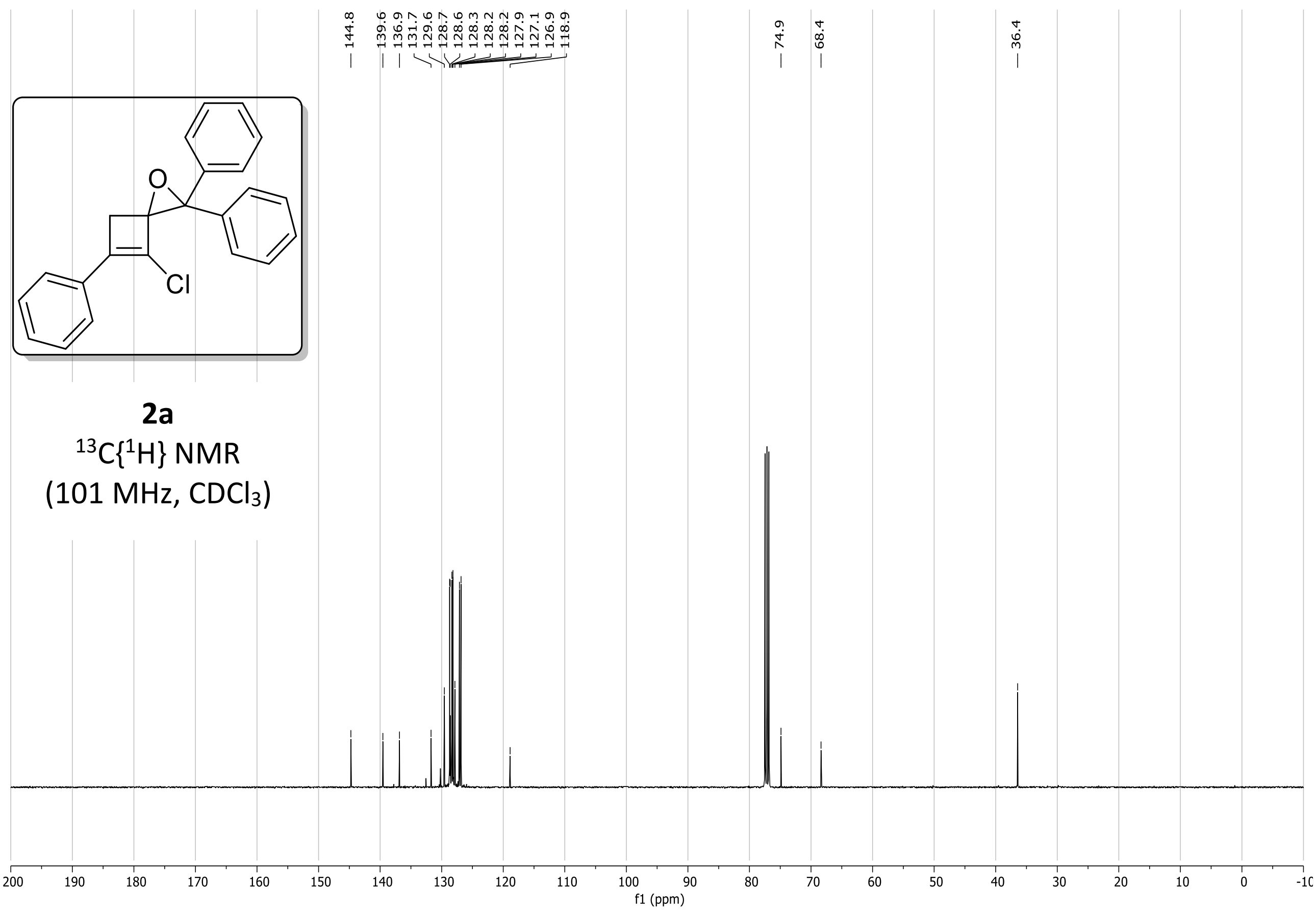

2a
$^{13}C\{^1H\}$ NMR
(101 MHz, $CDCl_3$)

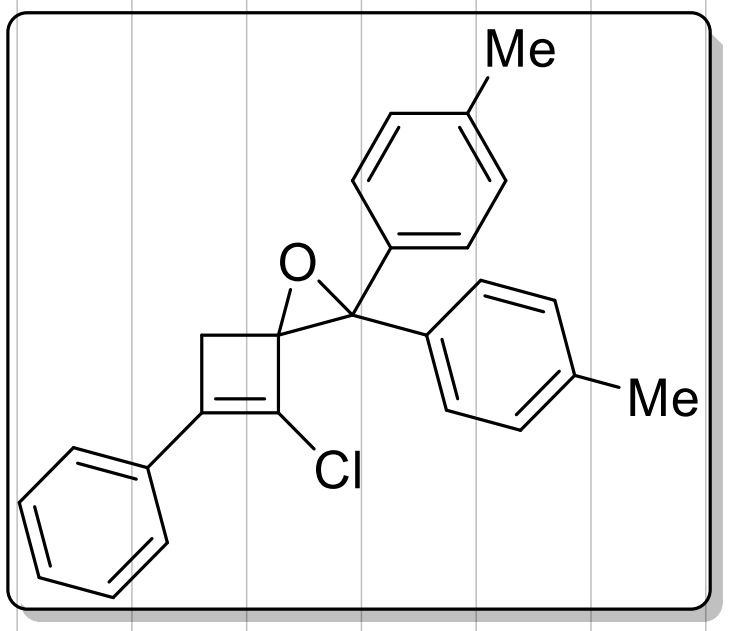

2b
^1H NMR
(400 MHz, $CDCl_3$)

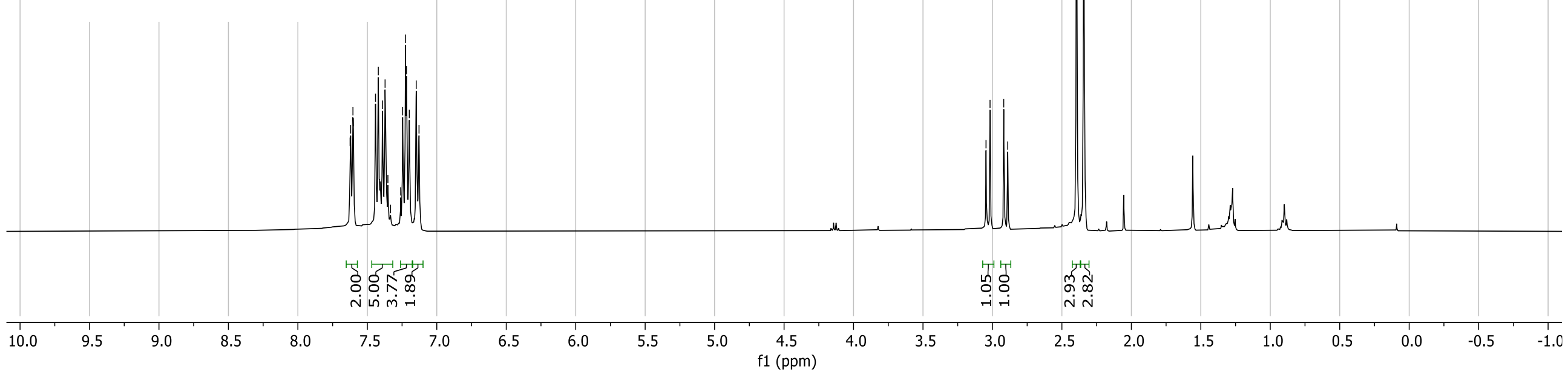

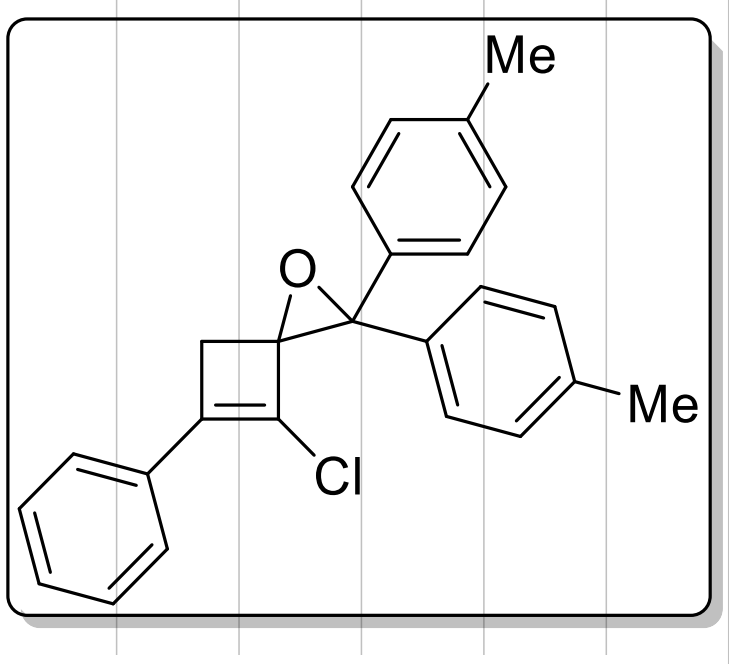

2b
$^{13}C\{^1H\}$ NMR
(101 MHz, $CDCl_3$)

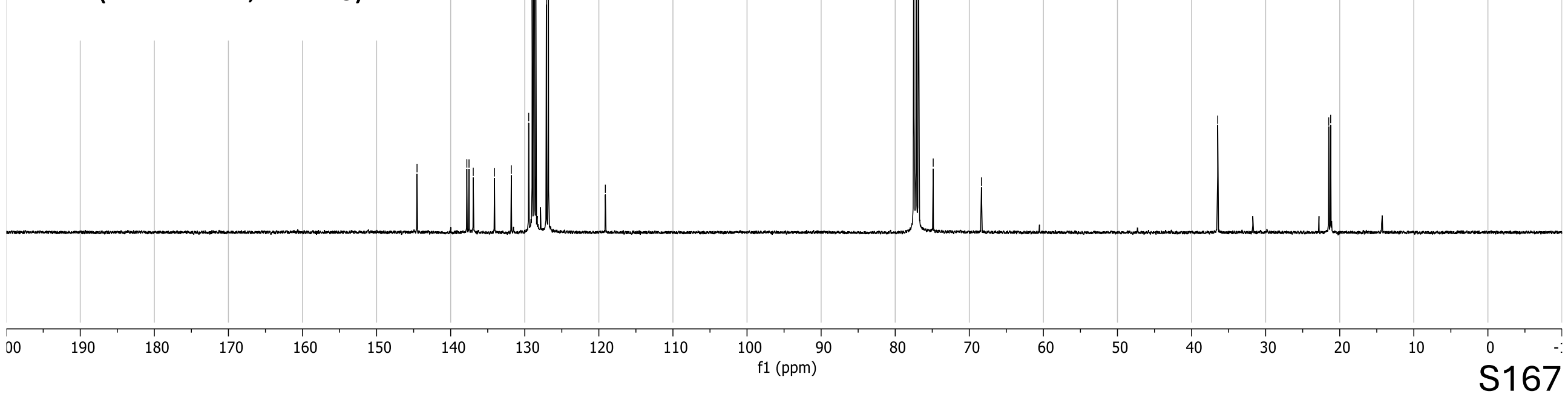

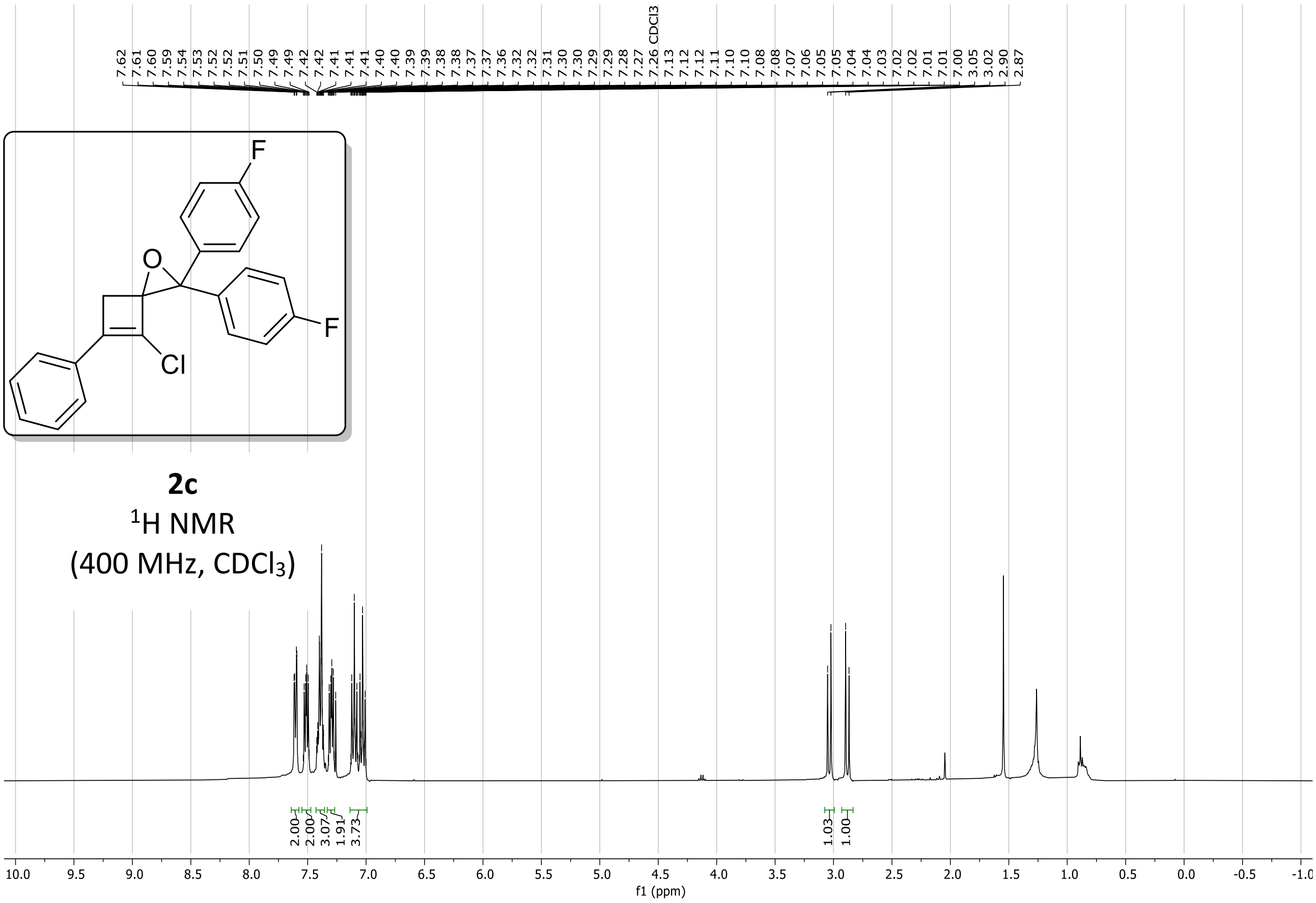

2c

^{1}H NMR

(400 MHz, $CDCl_3$)

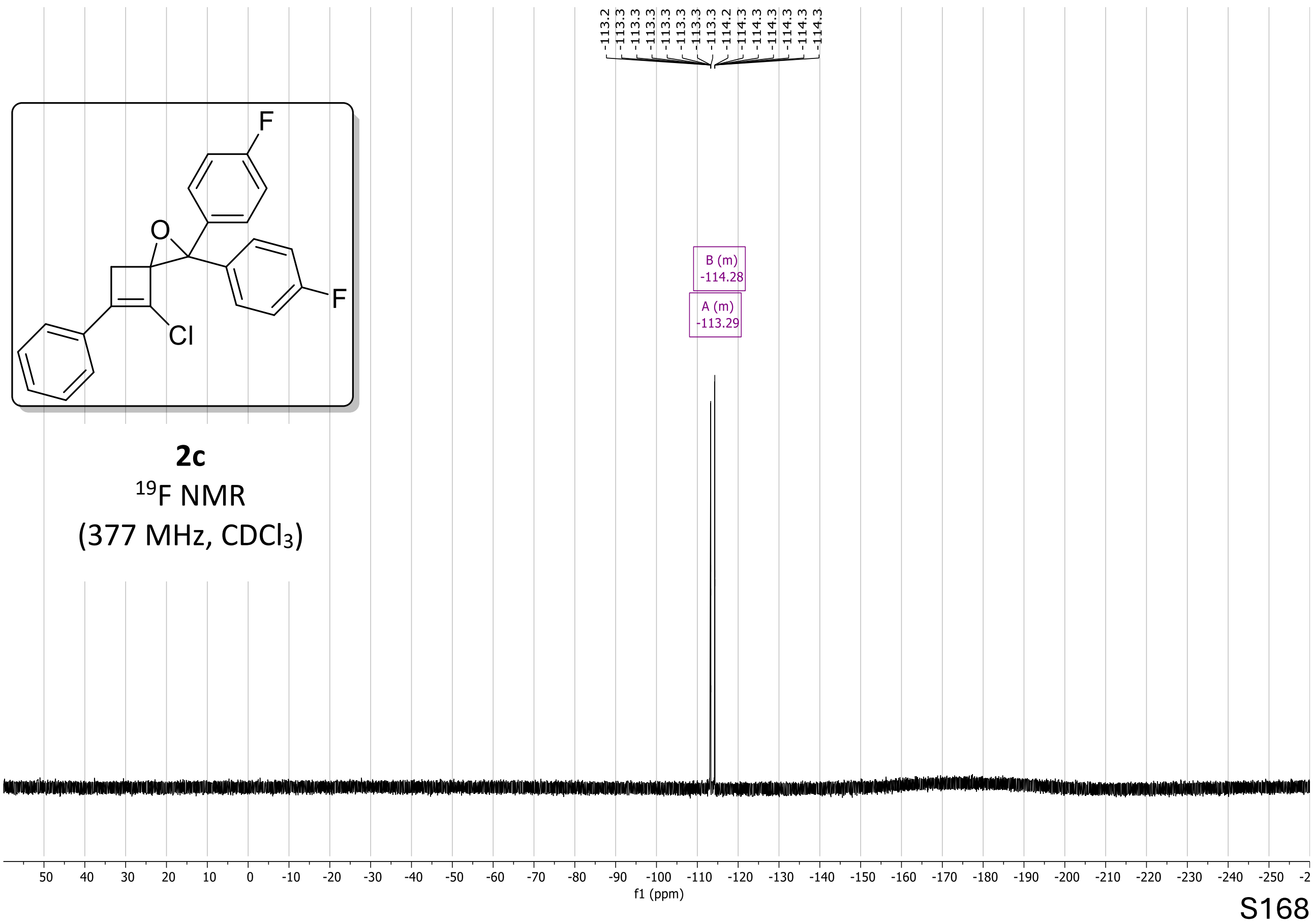

2c

^{19}F NMR

(377 MHz, $CDCl_3$)

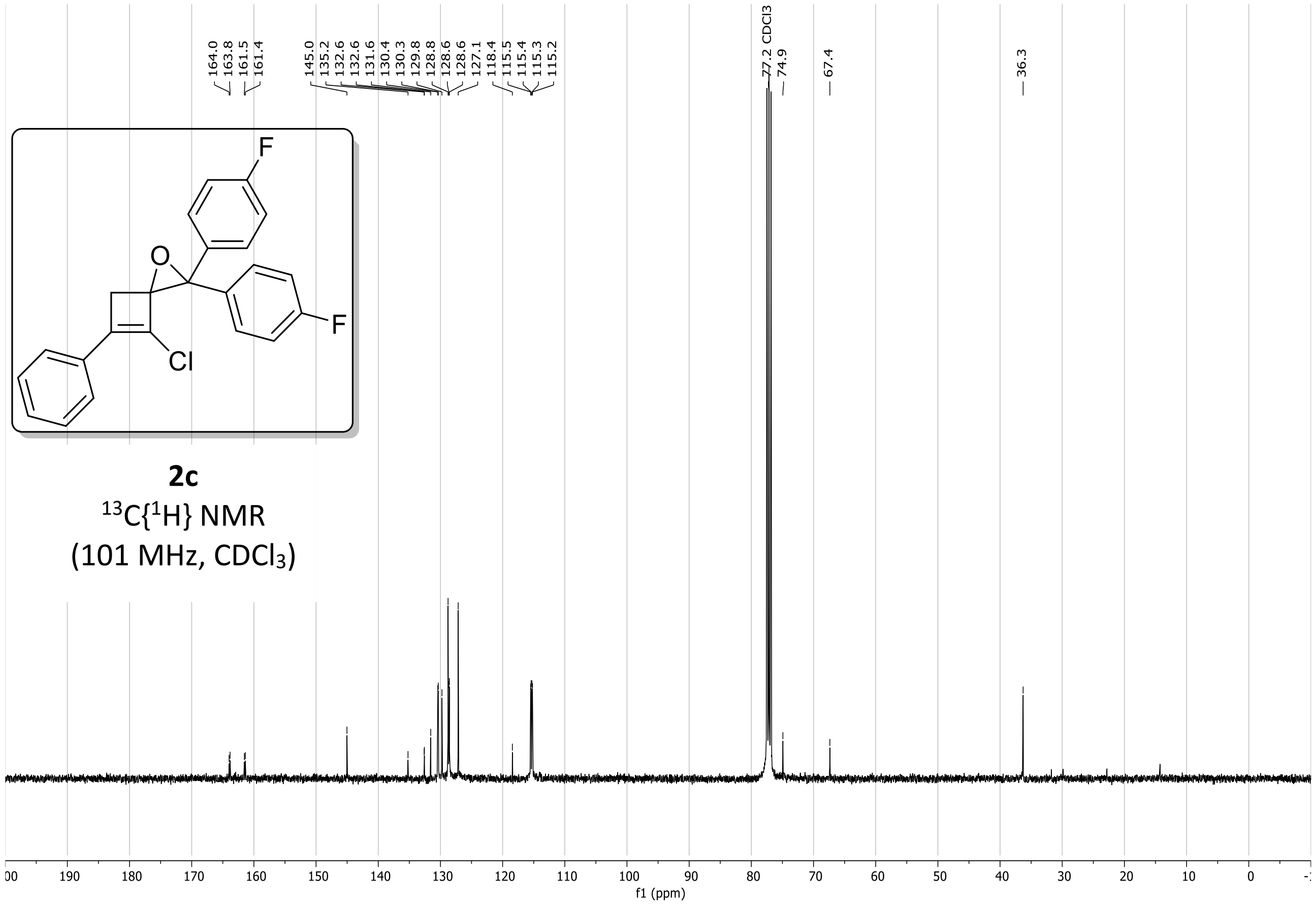

2c
$^{13}C\{^{1}H\}$ NMR
(101 MHz, $CDCl_3$)

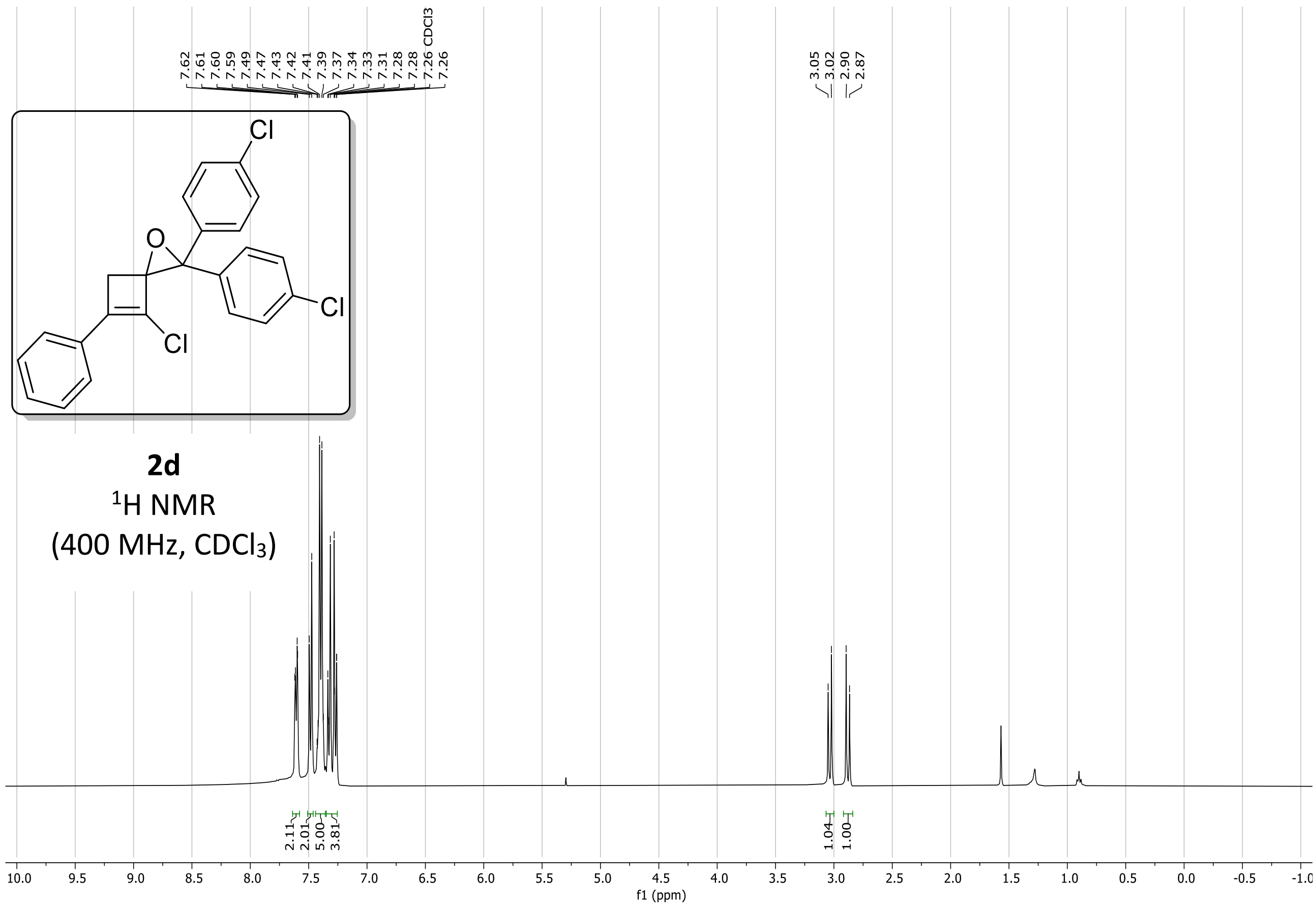

2d
^{1}H NMR
(400 MHz, $CDCl_3$)

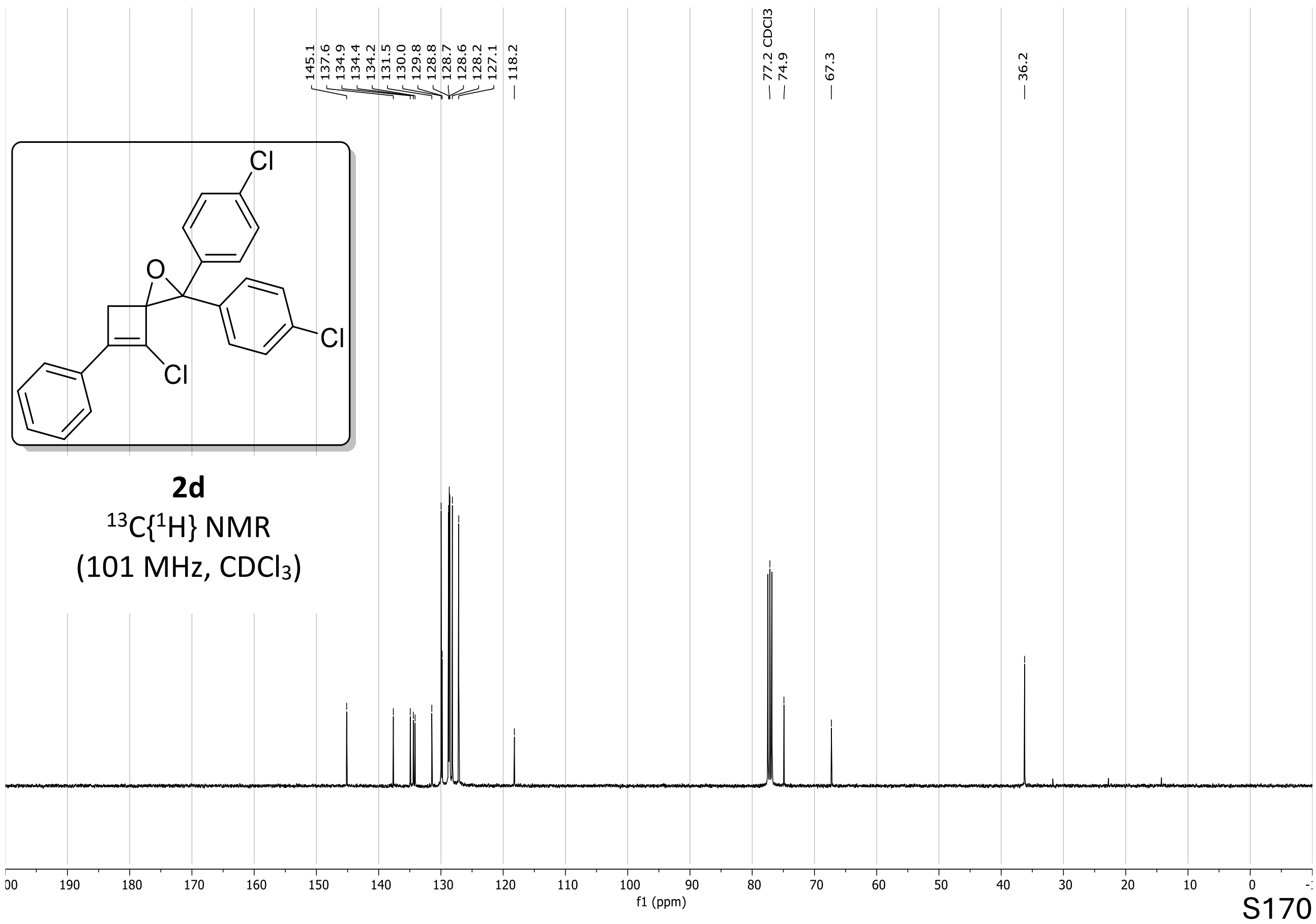

2d
$^{13}C\{^1H\}$ NMR
(101 MHz, $CDCl_3$)

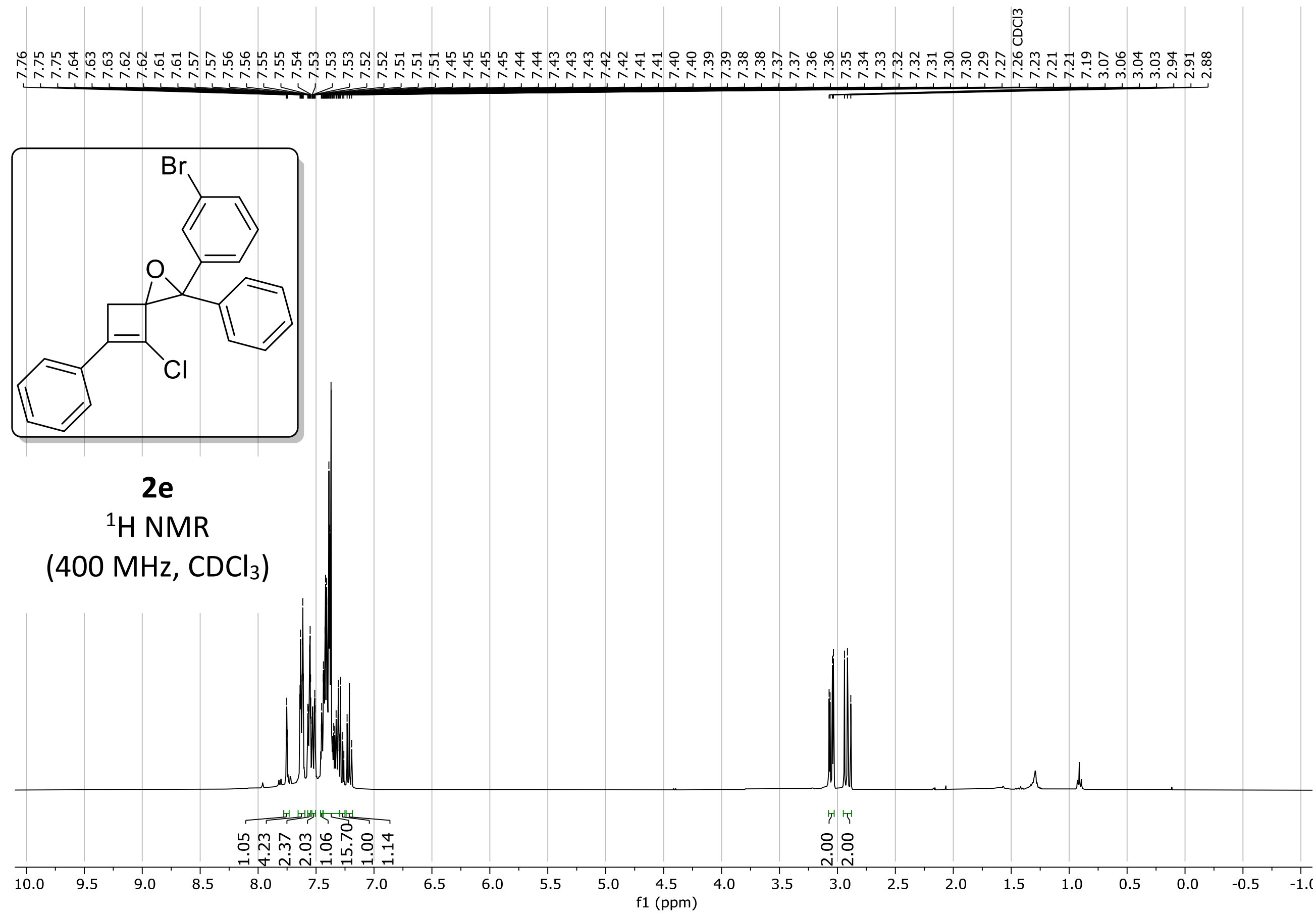
Br
O
Cl
2e
1H NMR
(400 MHz, CDCl3)
f1 (ppm)

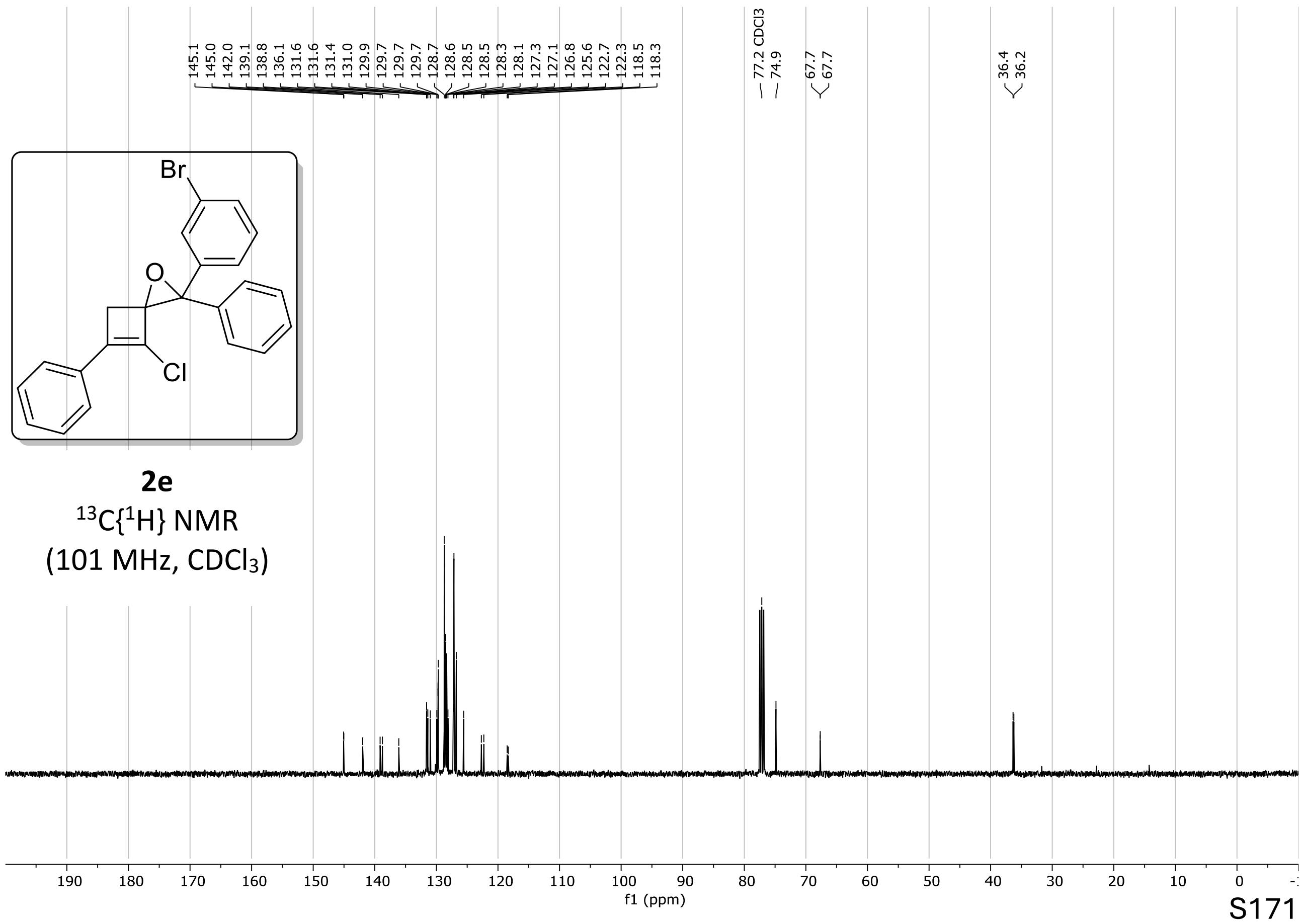
Br
O
Cl
2e
13C{1H} NMR
(101 MHz, CDCl3)
f1 (ppm)

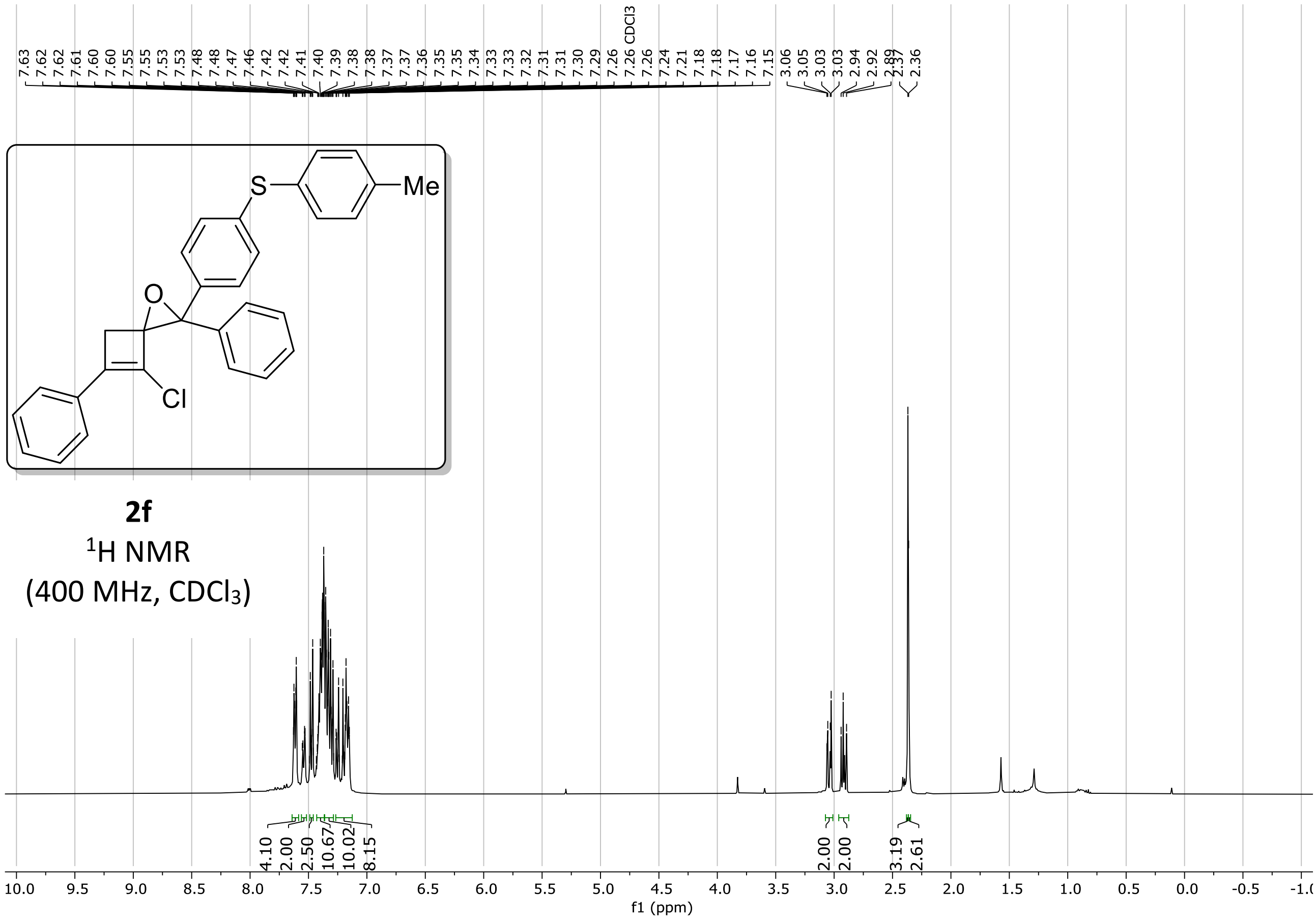

2f

^{1}H NMR

(400 MHz, $CDCl_3$)

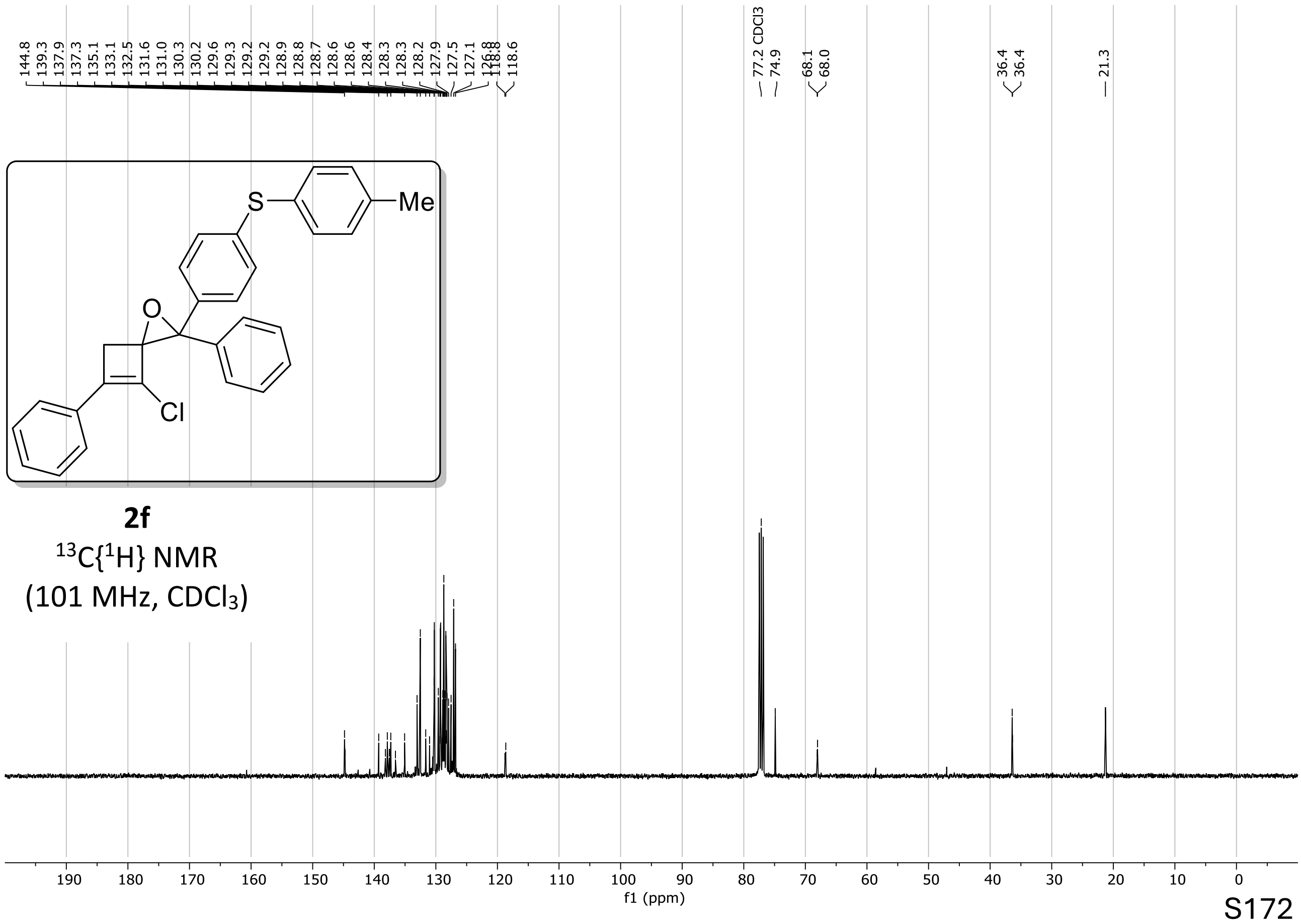

2f

$^{13}C\{^{1}H\}$ NMR

(101 MHz, $CDCl_3$)

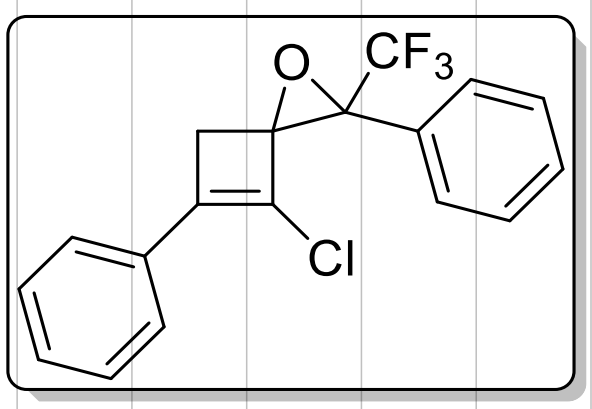

2g

^{1}H NMR

(400 MHz, $CDCl_3$)

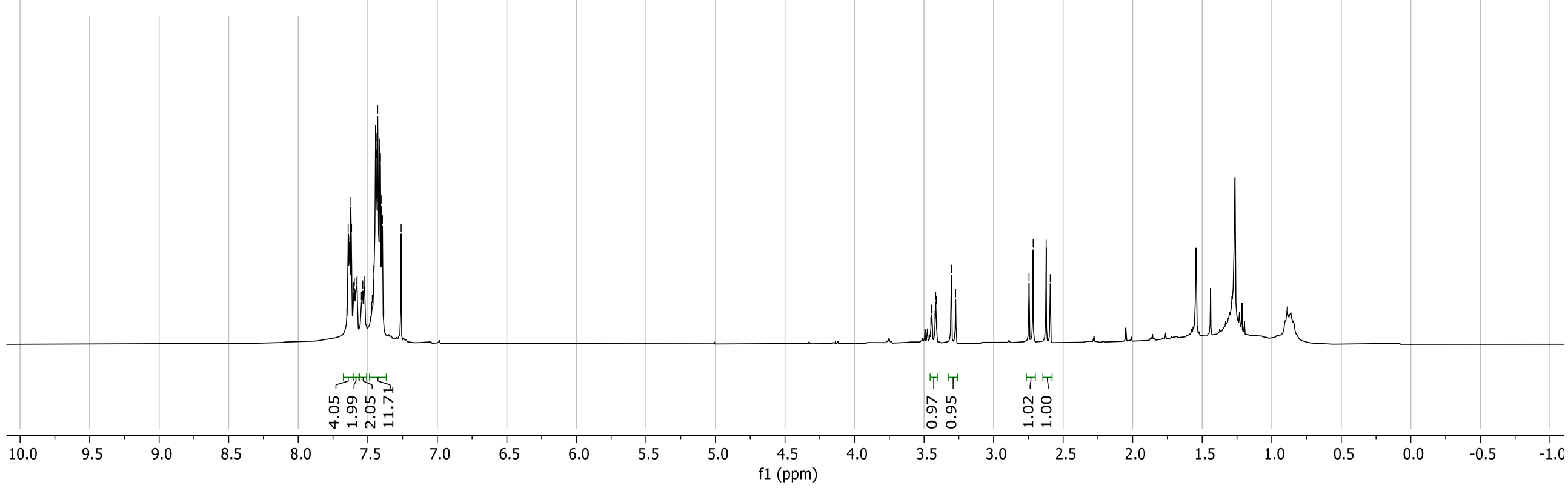

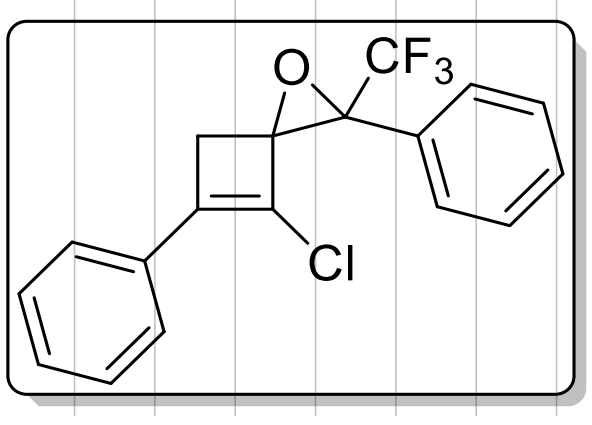

2g

^{19}F NMR

(377 MHz, $CDCl_3$)

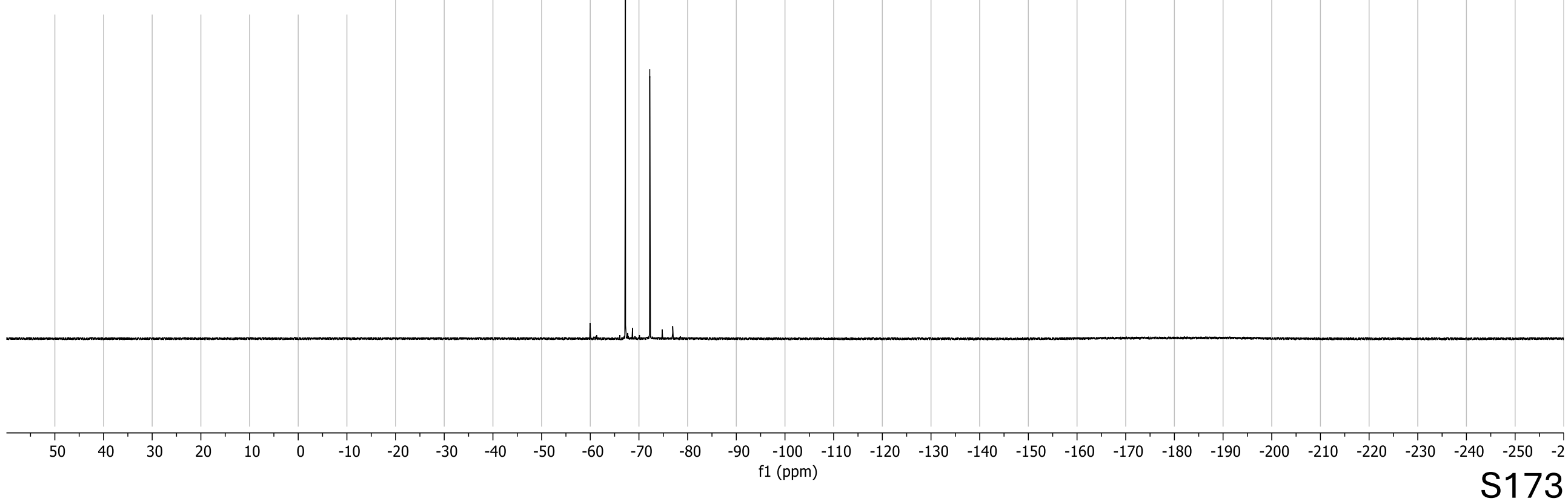

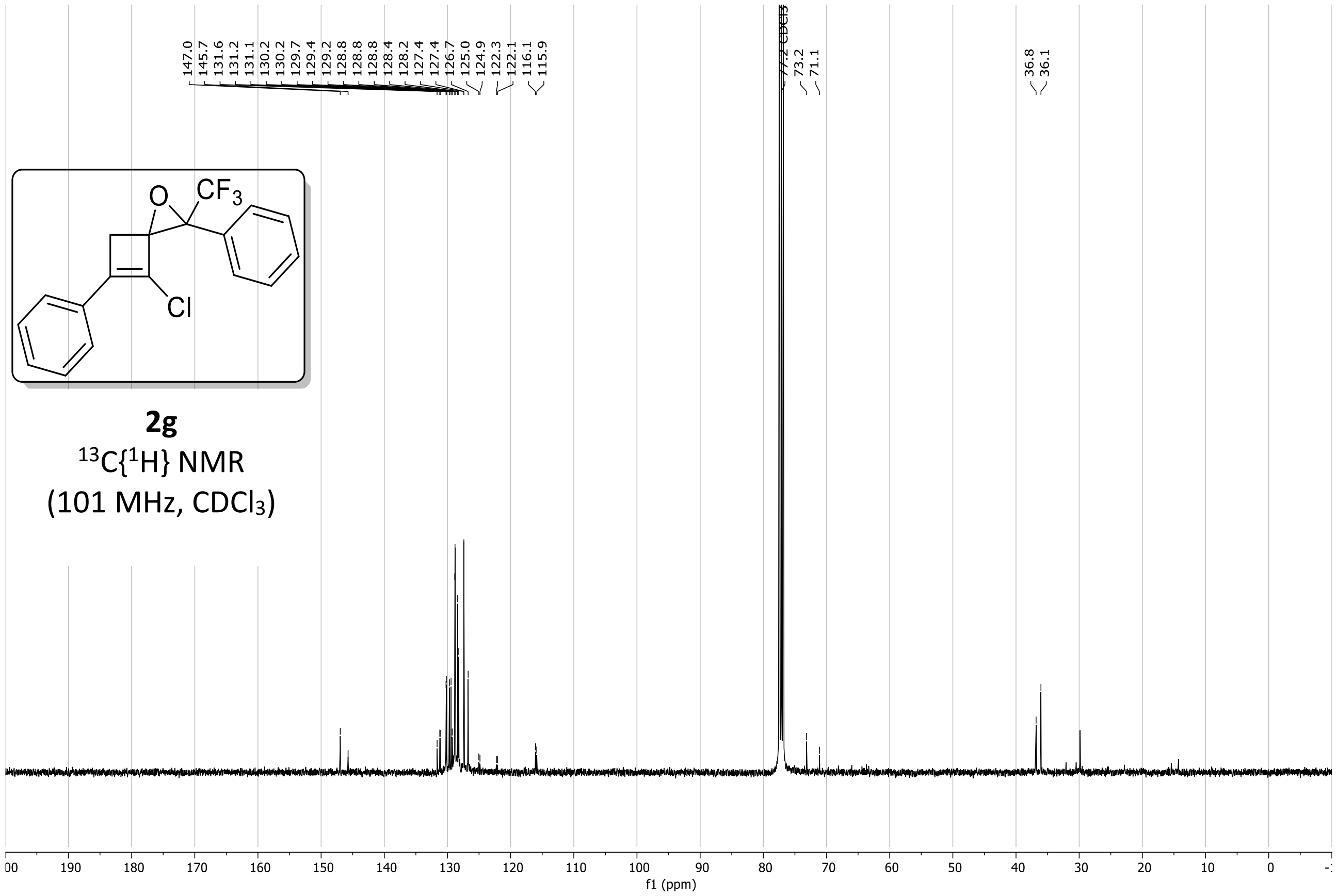
147.0
145.7
131.6
131.2
131.1
130.2
130.2
129.7
129.4
129.2
128.8
128.8
128.8
128.4
128.2
127.4
127.4
126.7
125.0
124.9
122.3
122.1
116.1
115.9
77.2 CDCl3
73.2
71.1
36.8
36.1
O
CF3
Cl
2g
13C{1H} NMR
(101 MHz, CDCl3)
190
180
170
160
150
140
130
120
110
100
90
80
70
60
50
40
30
20
10
0
f1 (ppm)

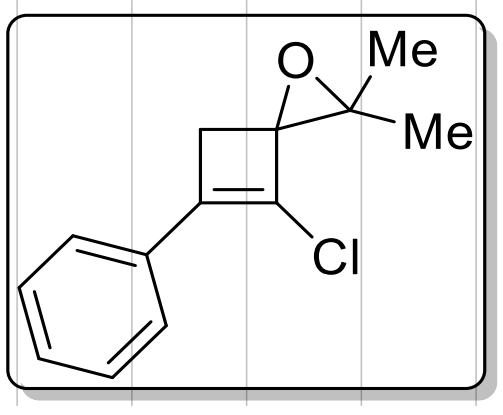

2h
^{1}H NMR
(400 MHz, $CDCl_3$)

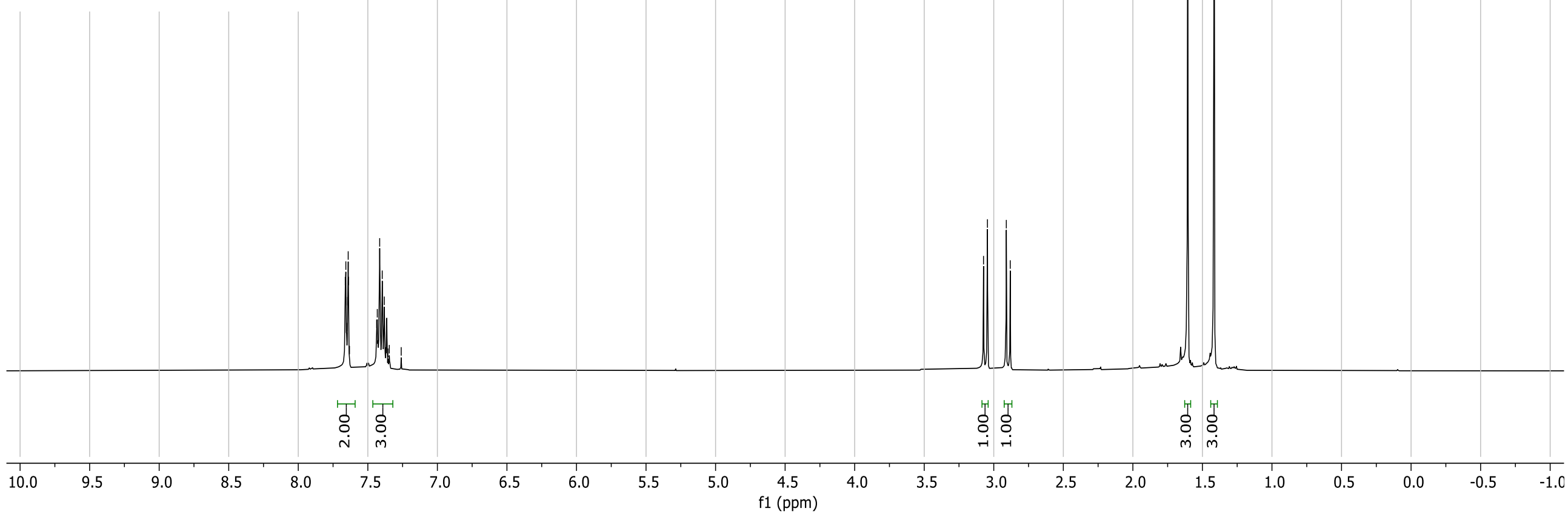

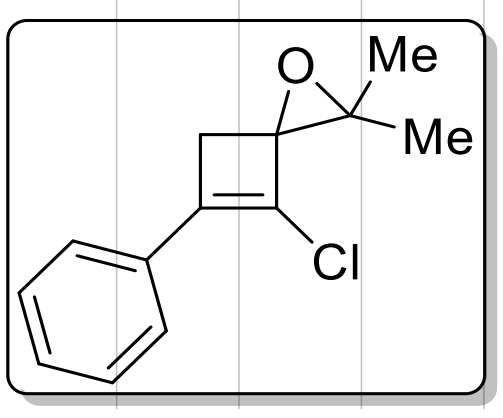

2h
$^{13}C\{^{1}H\}$ NMR
(101 MHz, $CDCl_3$)

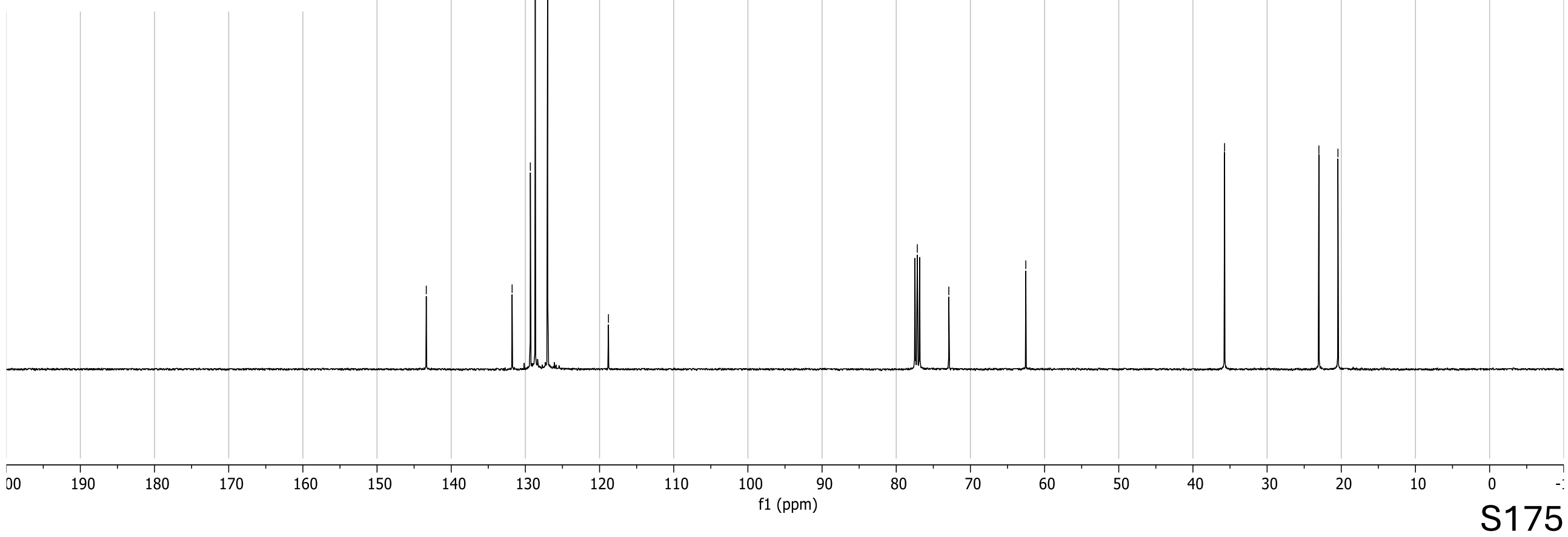

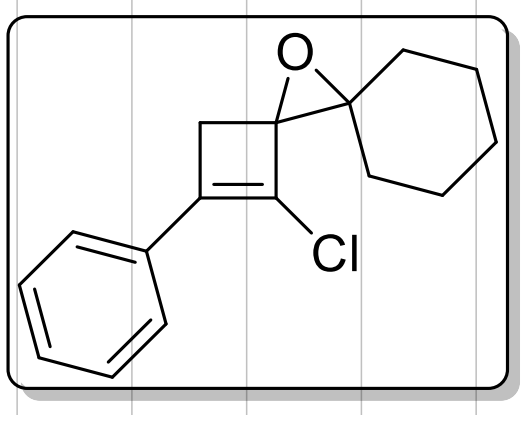

2i

^{1}H NMR

(400 MHz, $CDCl_3$)

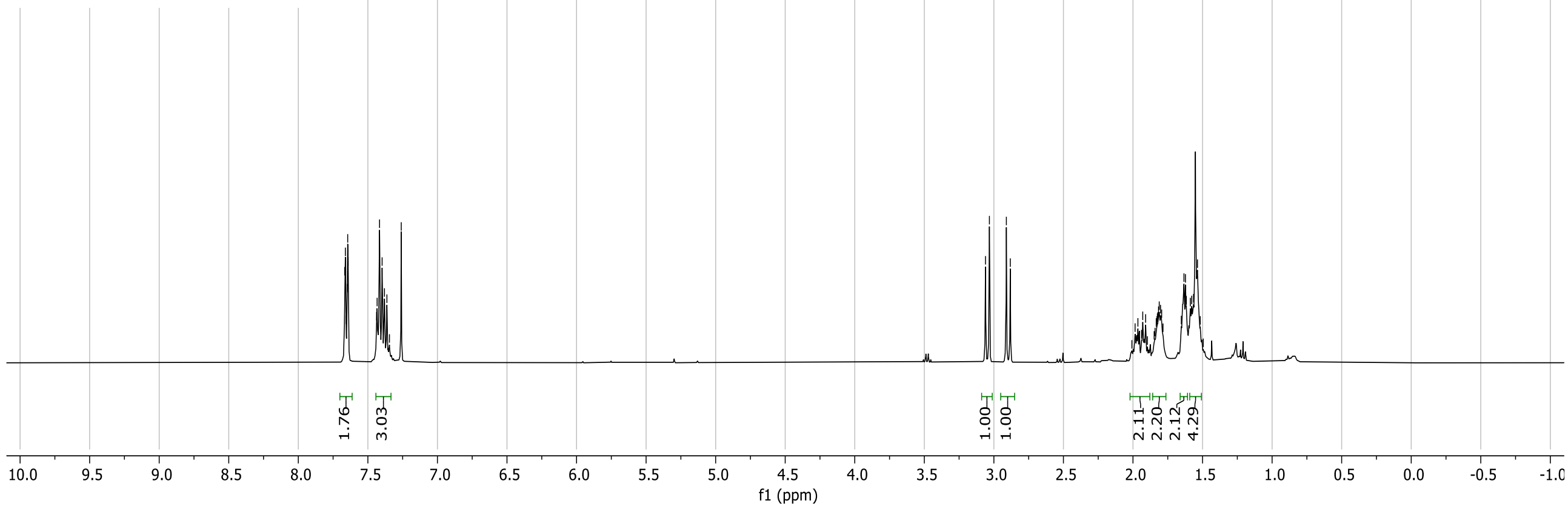

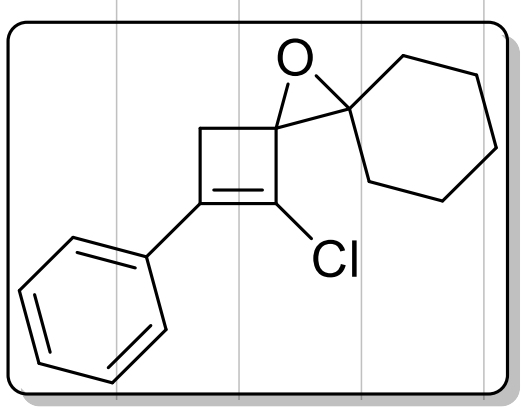

2i

$^{13}C\{^{1}H\}$ NMR

(101 MHz, $CDCl_3$)

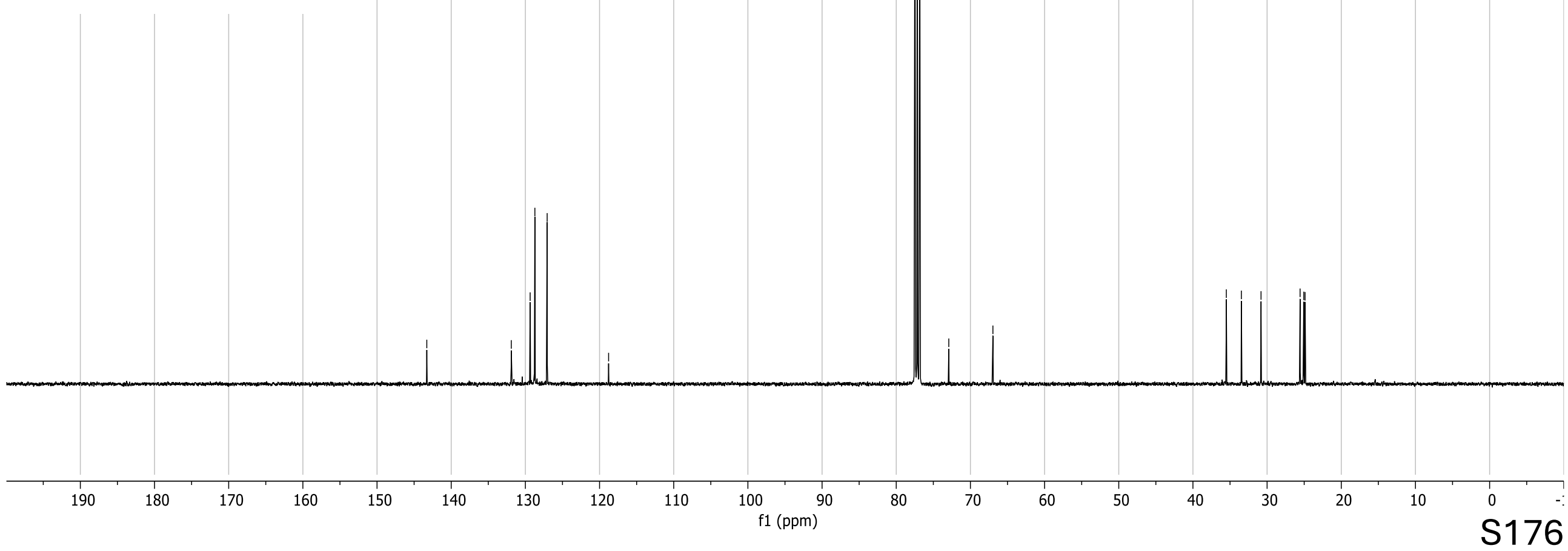

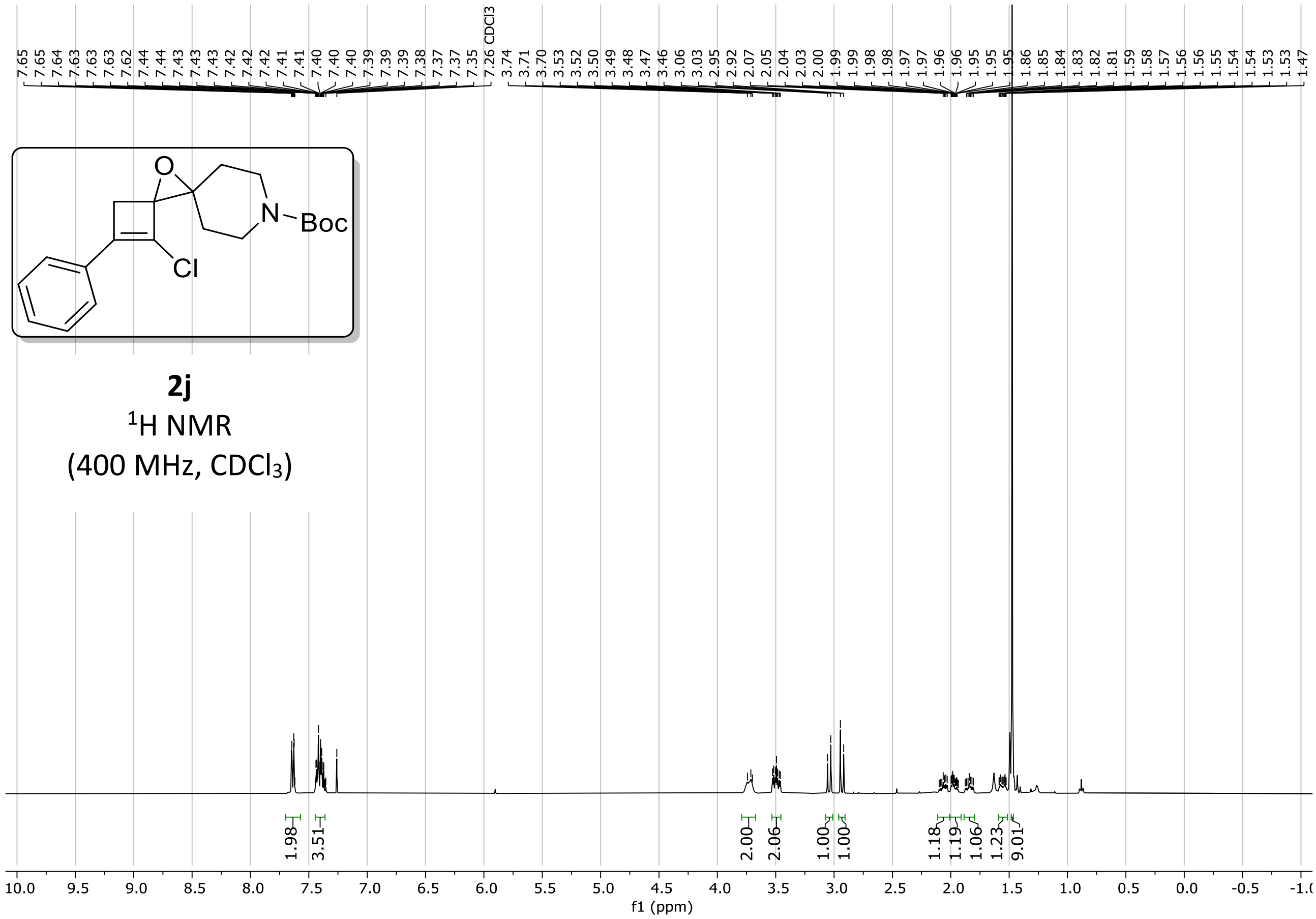

2j

^{1}H NMR

(400 MHz, $CDCl_3$)

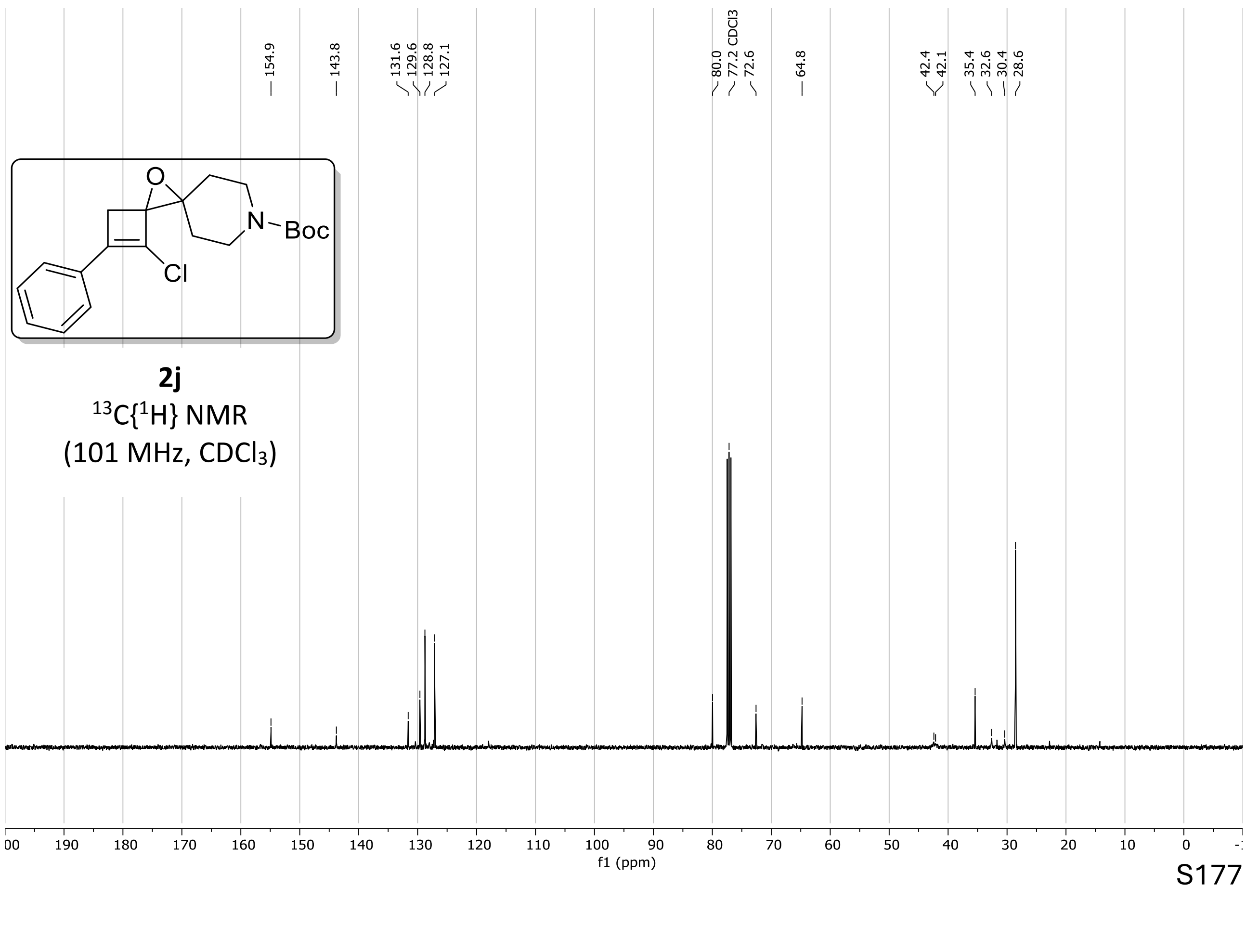

2j

$^{13}C\{^{1}H\}$ NMR

(101 MHz, $CDCl_3$)

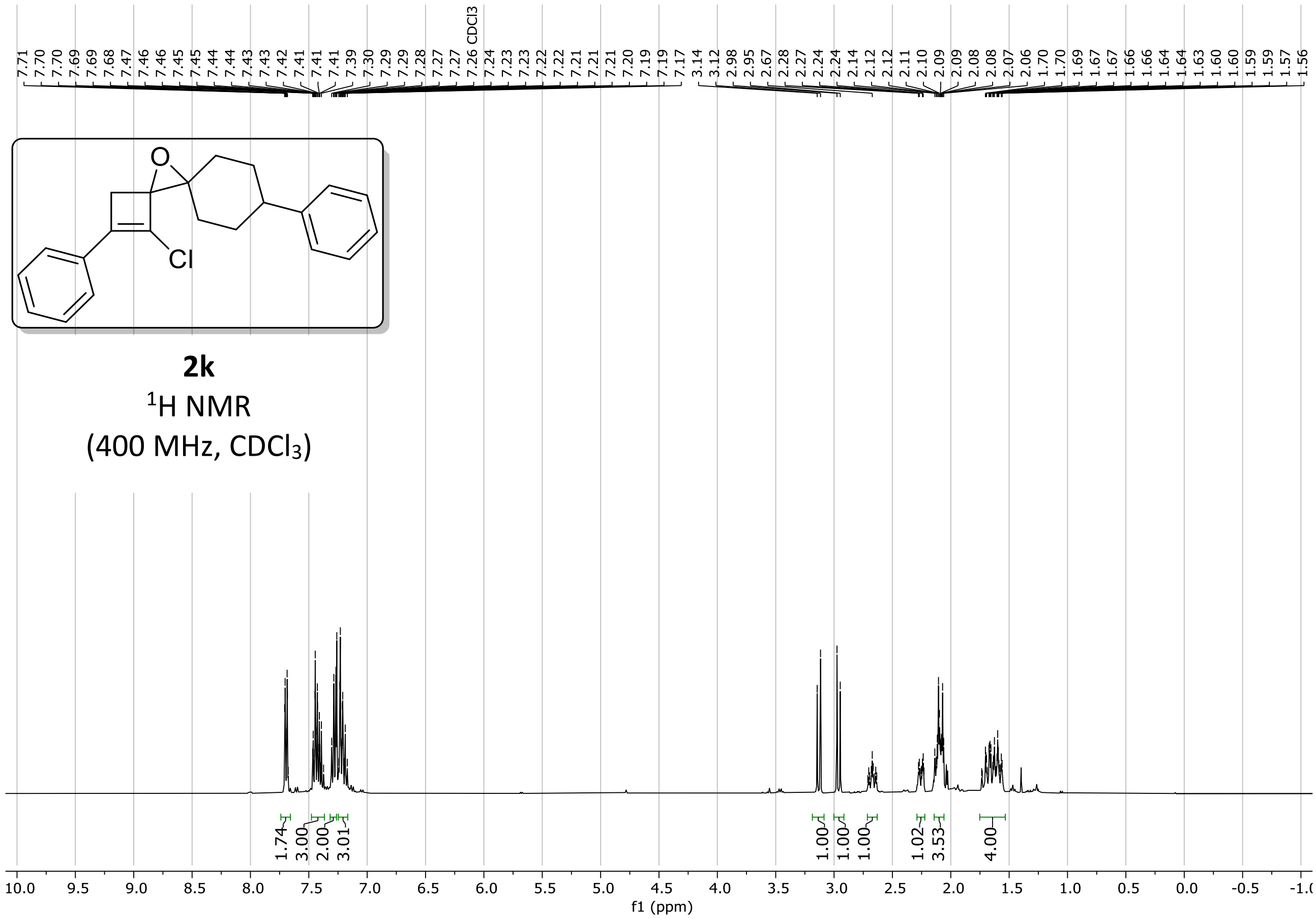

2k
^{1}H NMR
(400 MHz, $CDCl_3$)

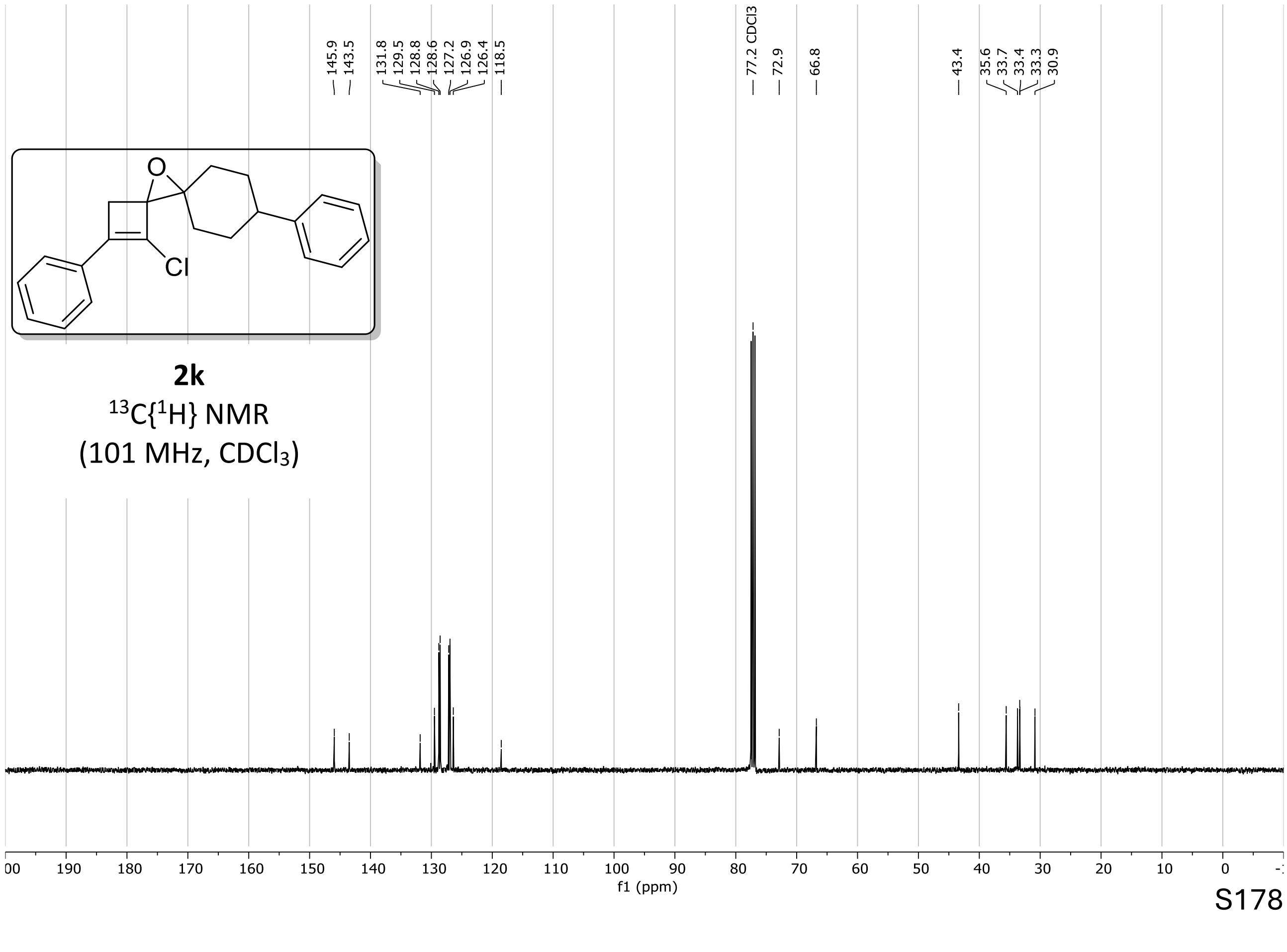

2k
$^{13}C\{^{1}H\}$ NMR
(101 MHz, $CDCl_3$)

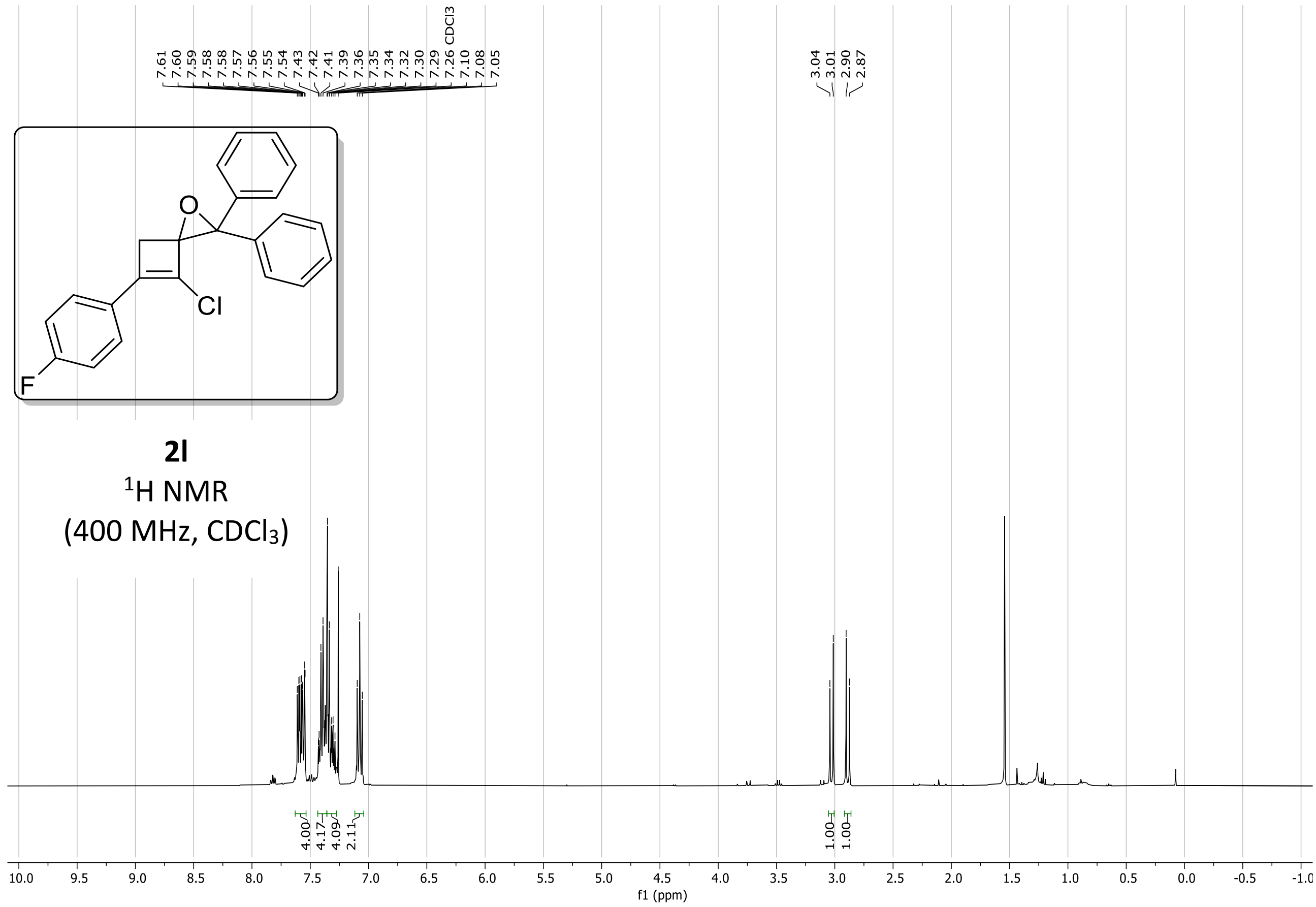

2l
^{1}H NMR
(400 MHz, $CDCl_3$)

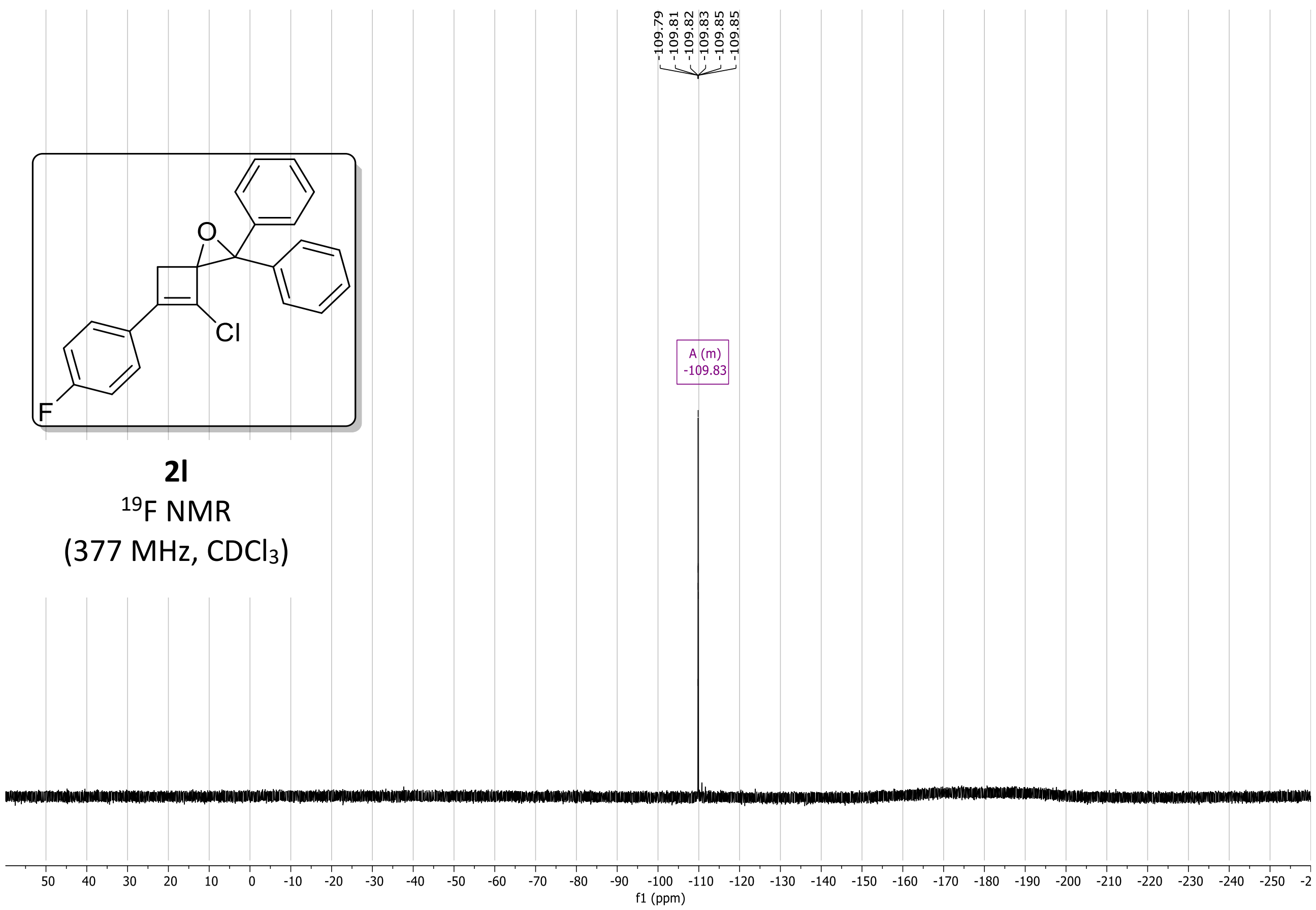

2l
^{19}F NMR
(377 MHz, $CDCl_3$)

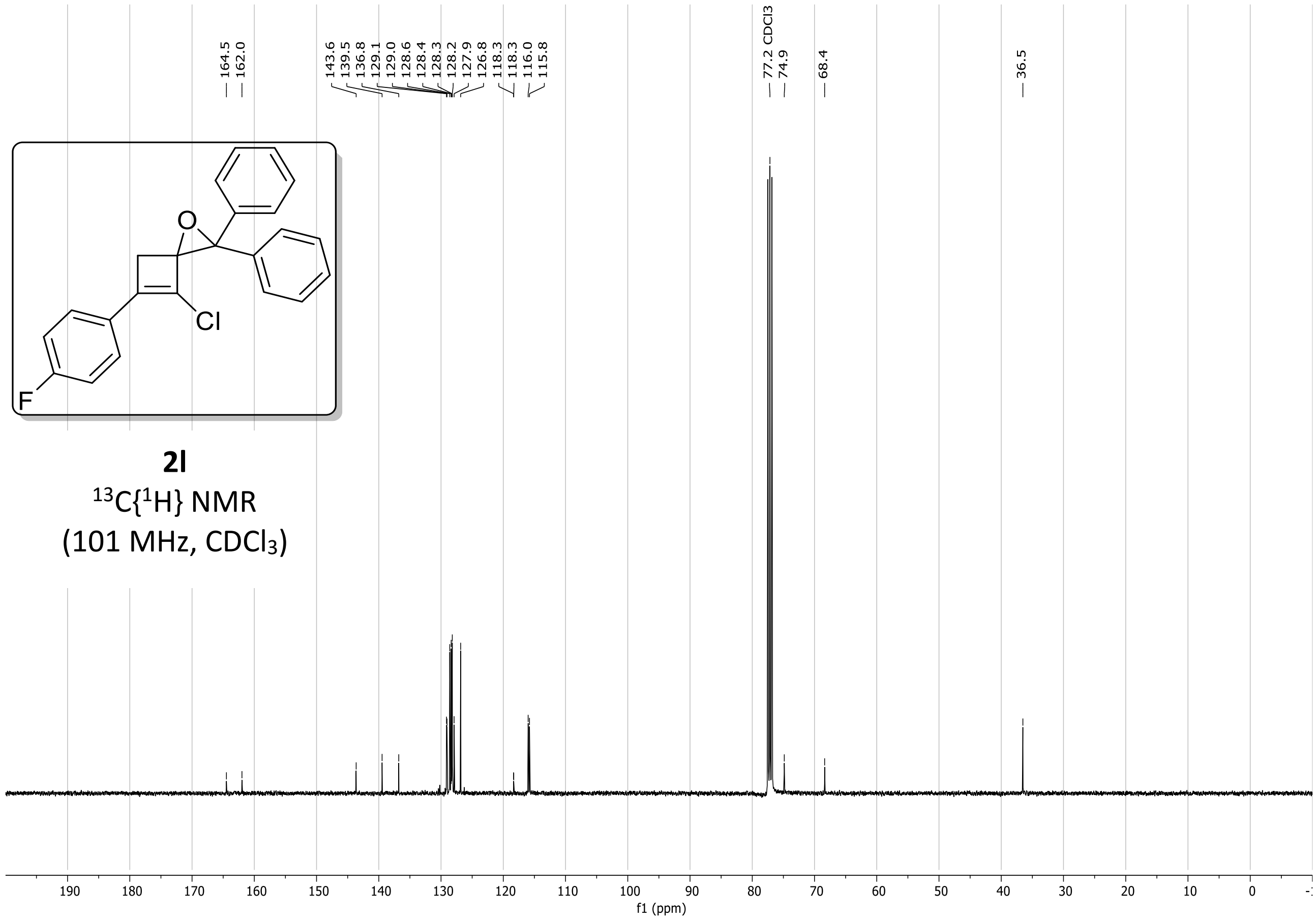

2l

$^{13}C\{^{1}H\}$ NMR

(101 MHz, $CDCl_3$)

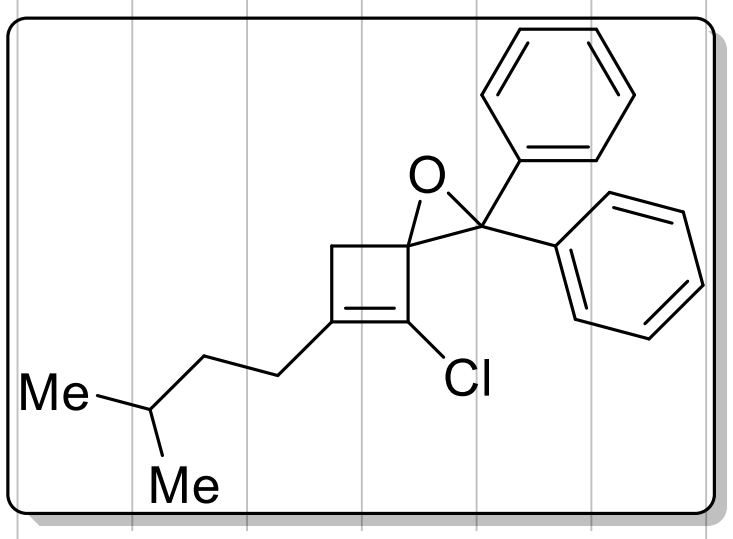

2m
^{1}H NMR
(400 MHz, $CDCl_3$)

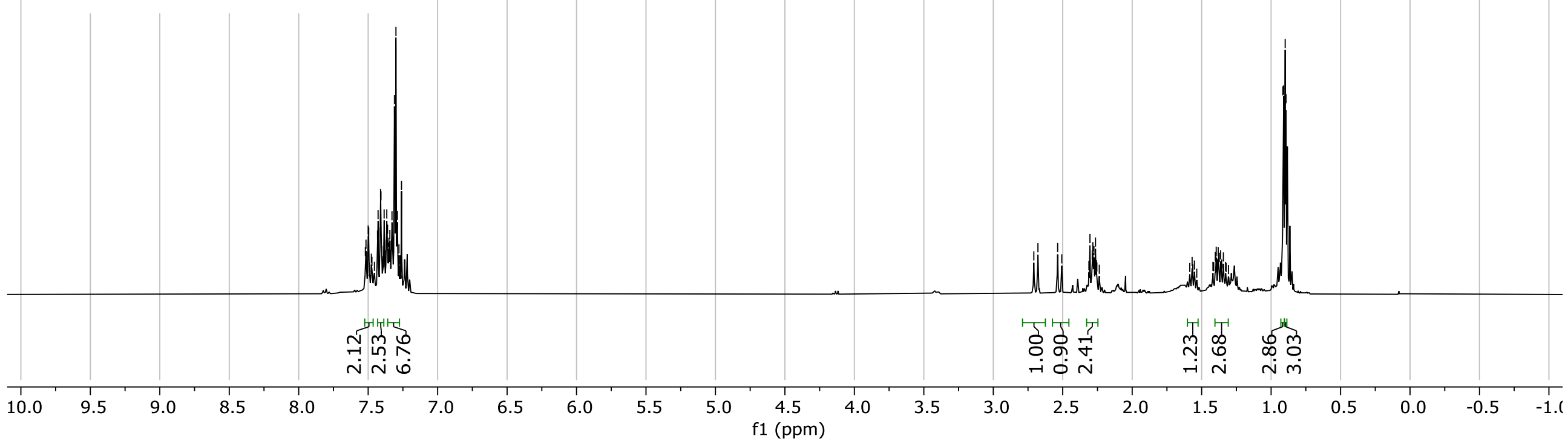

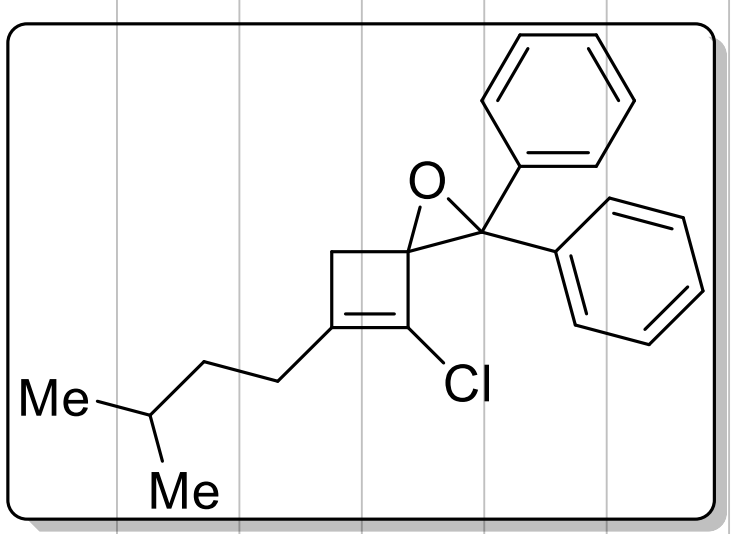

2m
$^{13}C\{^{1}H\}$ NMR
(101 MHz, $CDCl_3$)

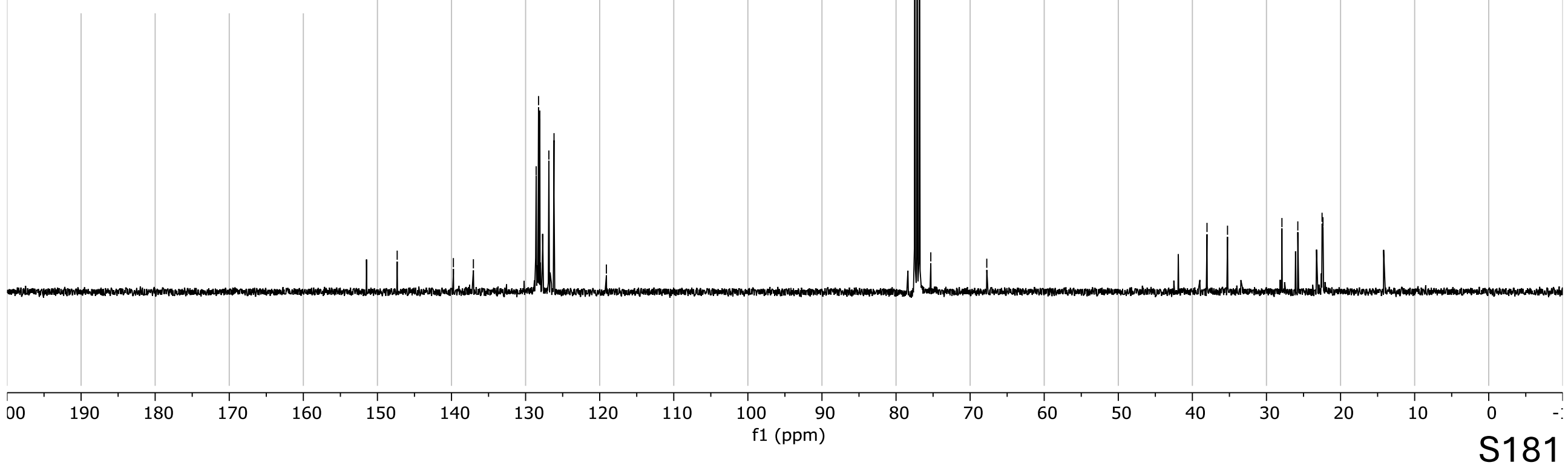

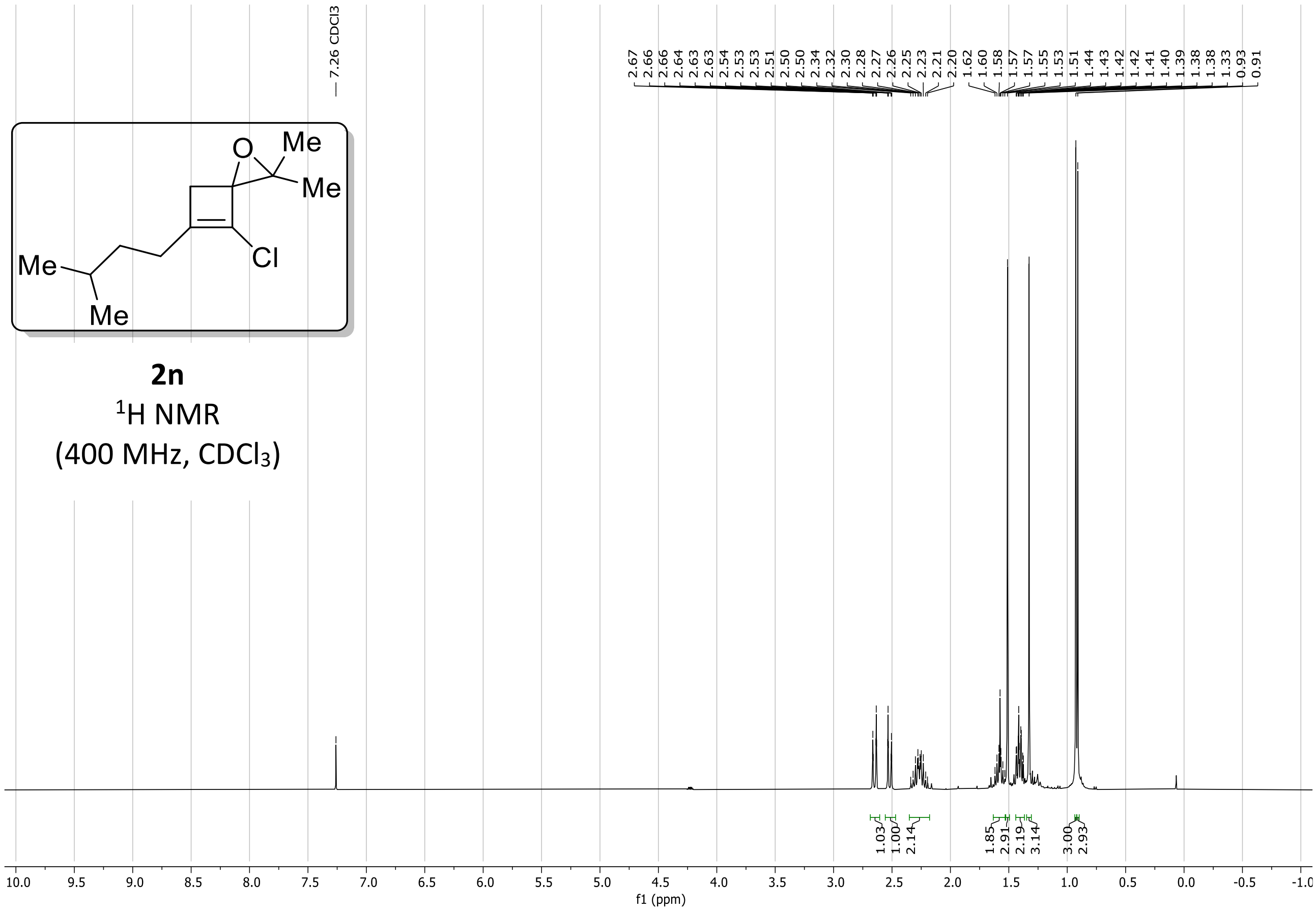

2n

^{1}H NMR

(400 MHz, $CDCl_3$)

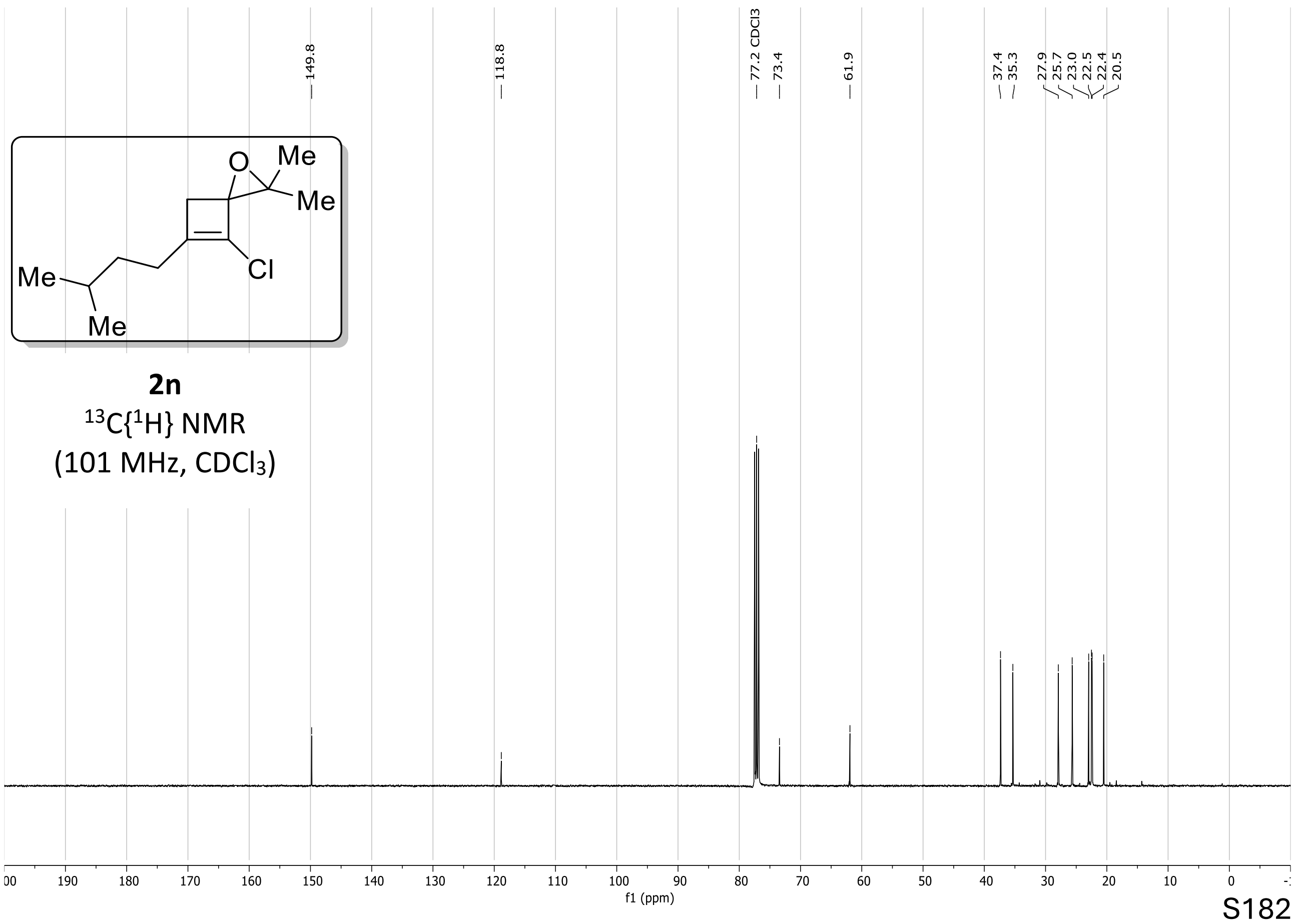

2n

$^{13}C\{^{1}H\}$ NMR

(101 MHz, $CDCl_3$)

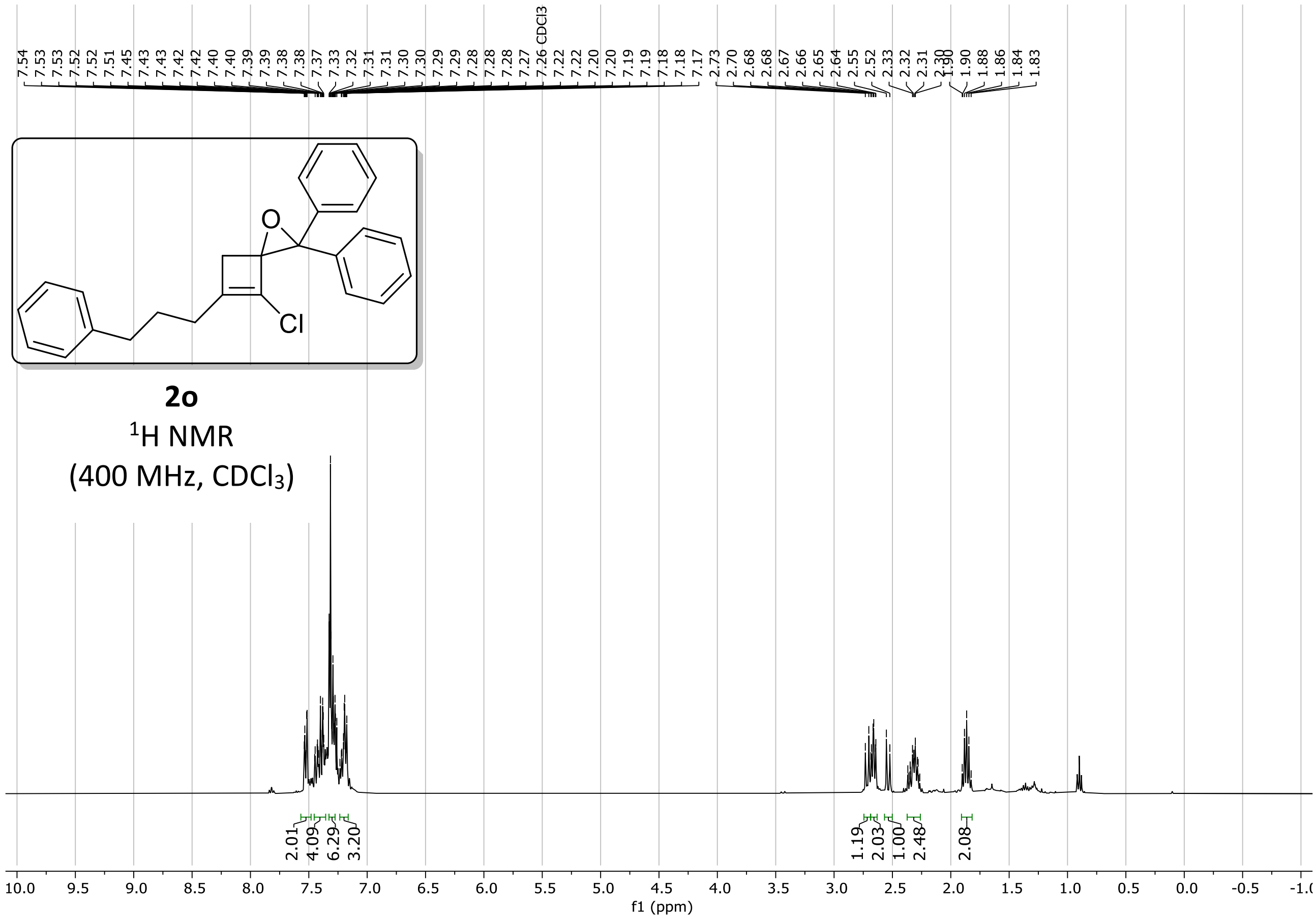
2o
1H NMR
(400 MHz, CDCl3)
7.54 7.53 7.53 7.52 7.52 7.51 7.45 7.43 7.43 7.43 7.42 7.42 7.40 7.40 7.39 7.39 7.38 7.38 7.37 7.33 7.32 7.31 7.31 7.30 7.30 7.29 7.29 7.28 7.28 7.28 7.27 7.26 CDCl3 7.22 7.22 7.22 7.20 7.20 7.19 7.19 7.18 7.18 7.17
2.73 2.70 2.68 2.68 2.67 2.66 2.65 2.64 2.55 2.52 2.33 2.32 2.31 2.30 1.90 1.88 1.86 1.84 1.83
2.01 4.09 6.29 3.20 1.19 2.03 1.00 2.48 2.08
f1 (ppm)
O
Cl

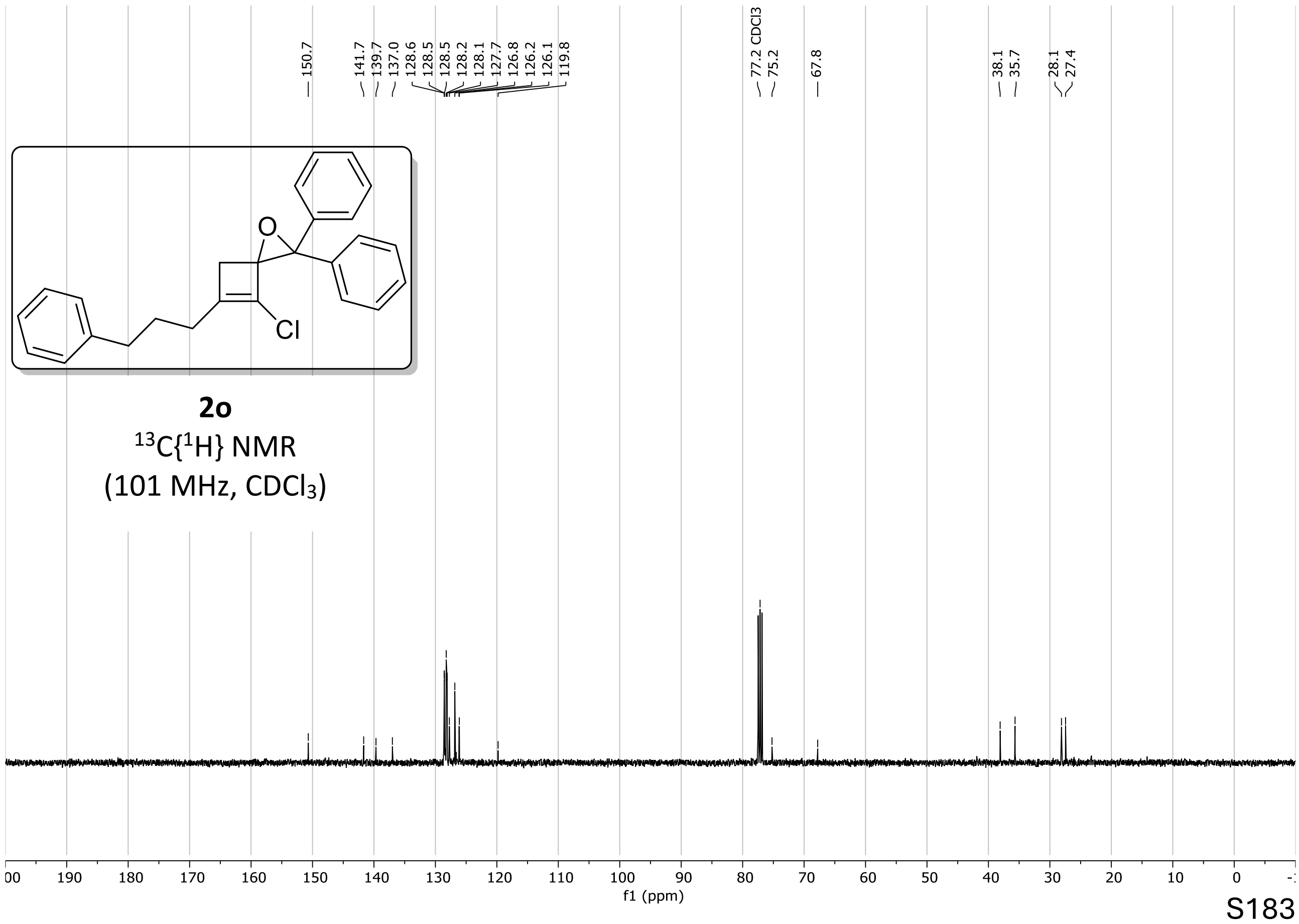
2o
13C{1H} NMR
(101 MHz, CDCl3)
150.7 141.7 139.7 137.0 128.6 128.5 128.5 128.2 128.1 127.7 126.8 126.2 126.1 119.8
77.2 CDCl3 75.2 67.8
38.1 35.7 28.1 27.4
f1 (ppm)
O
Cl

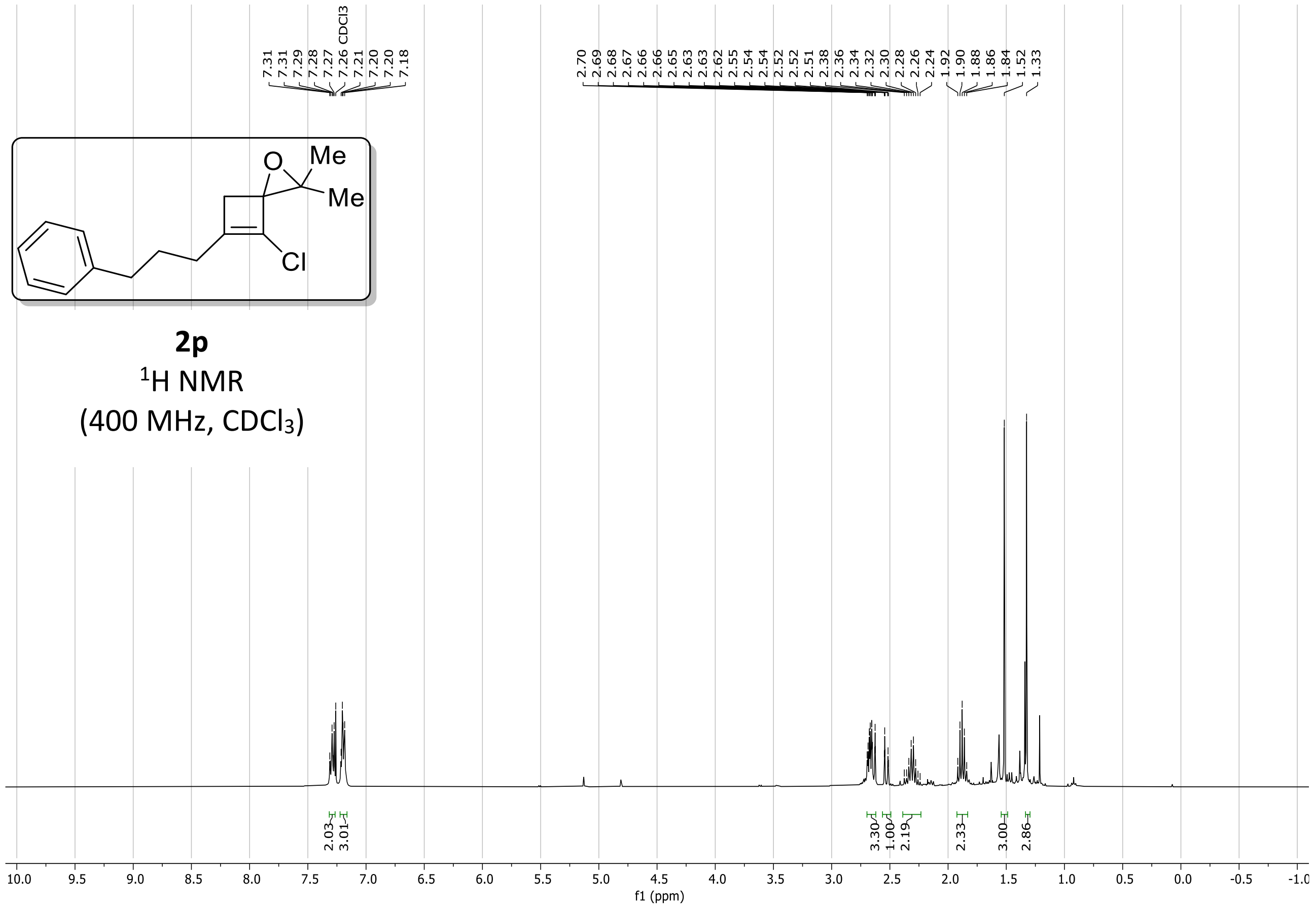

2p
^1H NMR
(400 MHz, $CDCl_3$)

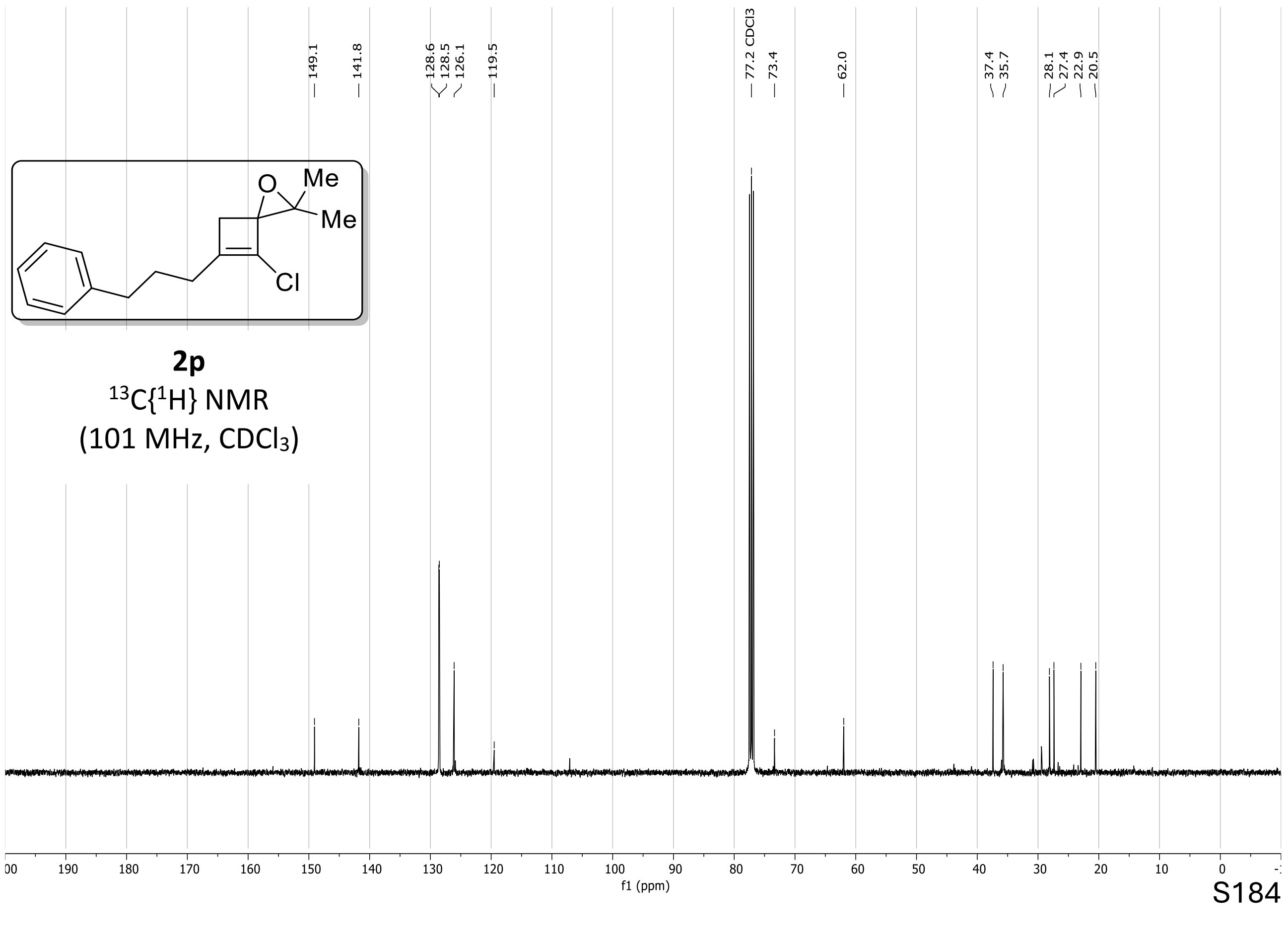

2p
$^{13}C\{^1H\}$ NMR
(101 MHz, $CDCl_3$)

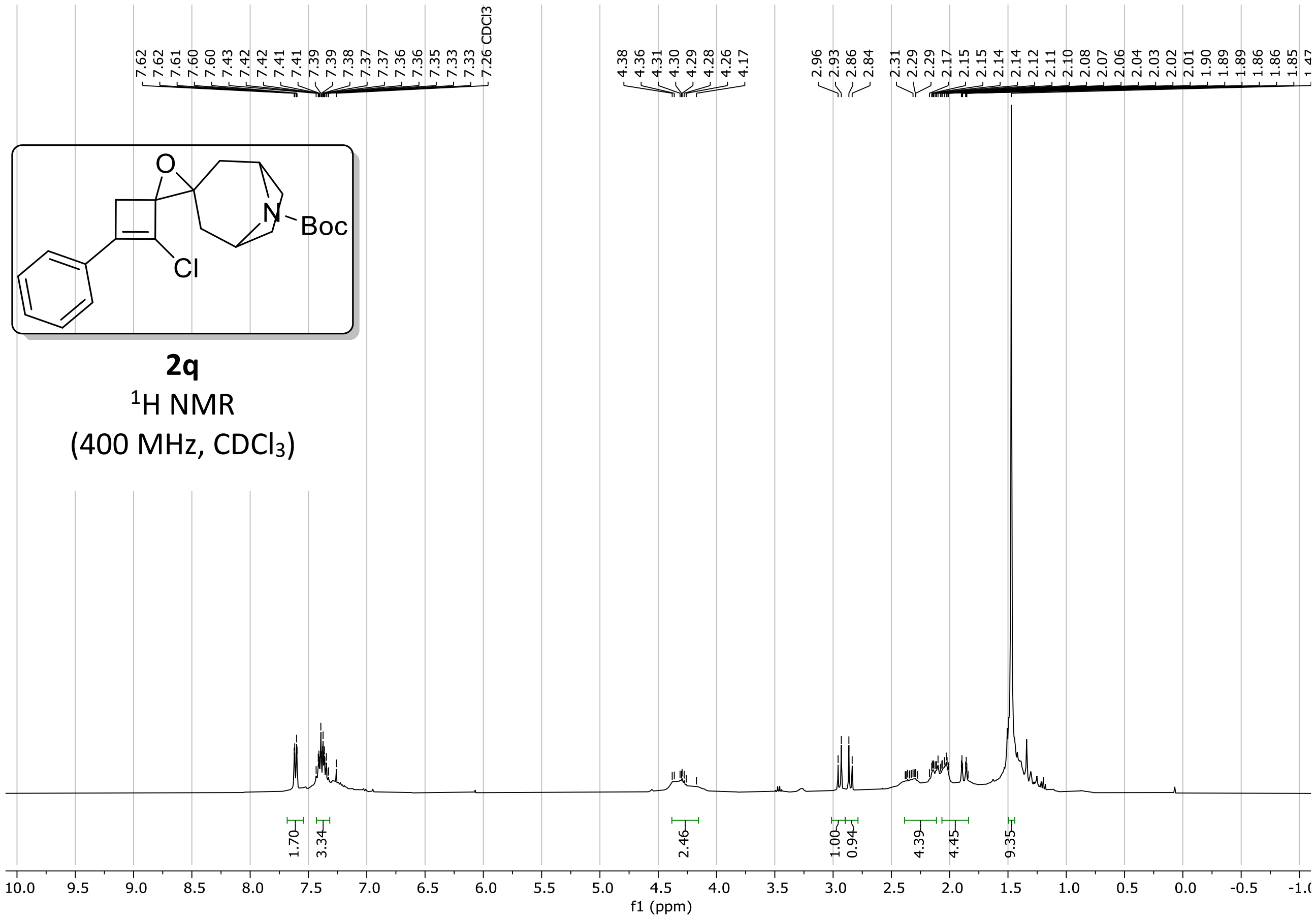

7.62 7.62 7.61 7.60 7.43 7.42 7.42 7.41 7.41 7.39 7.39 7.38 7.37 7.37 7.36 7.36 7.35 7.33 7.33 7.26 CDCl3
4.38 4.36 4.31 4.30 4.29 4.28 4.26 4.17
2.96 2.93 2.86 2.84
2.31 2.29 2.29 2.17 2.15 2.15 2.14 2.14 2.12 2.11 2.10 2.08 2.07 2.06 2.04 2.03 2.02 2.01 1.90 1.89 1.89 1.86 1.86 1.85
O
N
Boc
Cl
2q
^{1}H NMR
(400 MHz, $CDCl_3$)
1.70 3.34 2.46 1.00 0.94 4.39 4.45 9.35
10.0 9.5 9.0 8.5 8.0 7.5 7.0 6.5 6.0 5.5 5.0 4.5 4.0 3.5 3.0 2.5 2.0 1.5 1.0 0.5 0.0 -0.5
f1 (ppm)

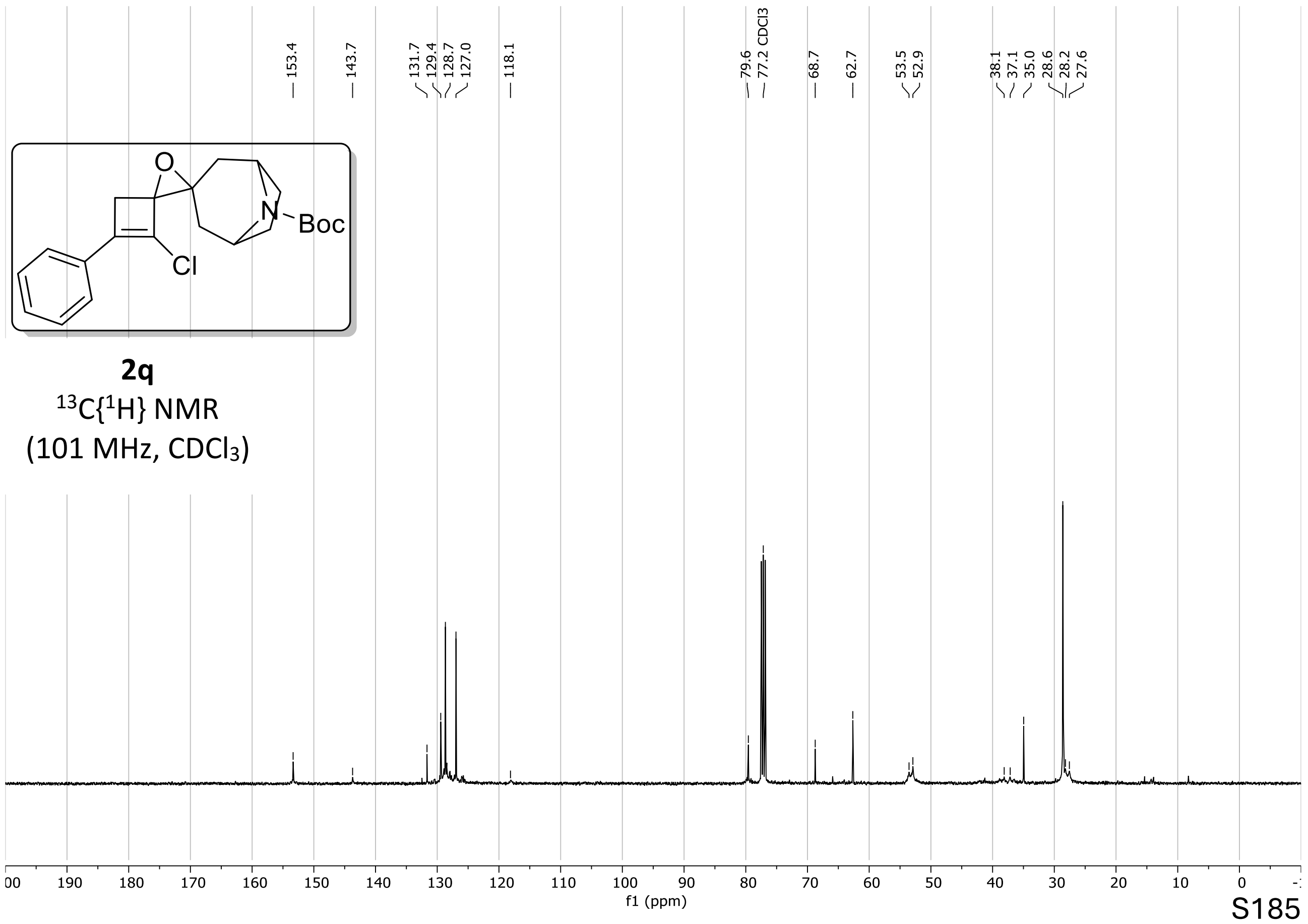

153.4
143.7
131.7 129.4 128.7 127.0
118.1
79.6 77.2 CDCl3
68.7
62.7
53.5 52.9
38.1 37.1 35.0 28.6 28.2 27.6
O
N
Boc
Cl
2q
$^{13}C\{^{1}H\}$ NMR
(101 MHz, $CDCl_3$)
190 180 170 160 150 140 130 120 110 100 90 80 70 60 50 40 30 20 10 0
f1 (ppm)

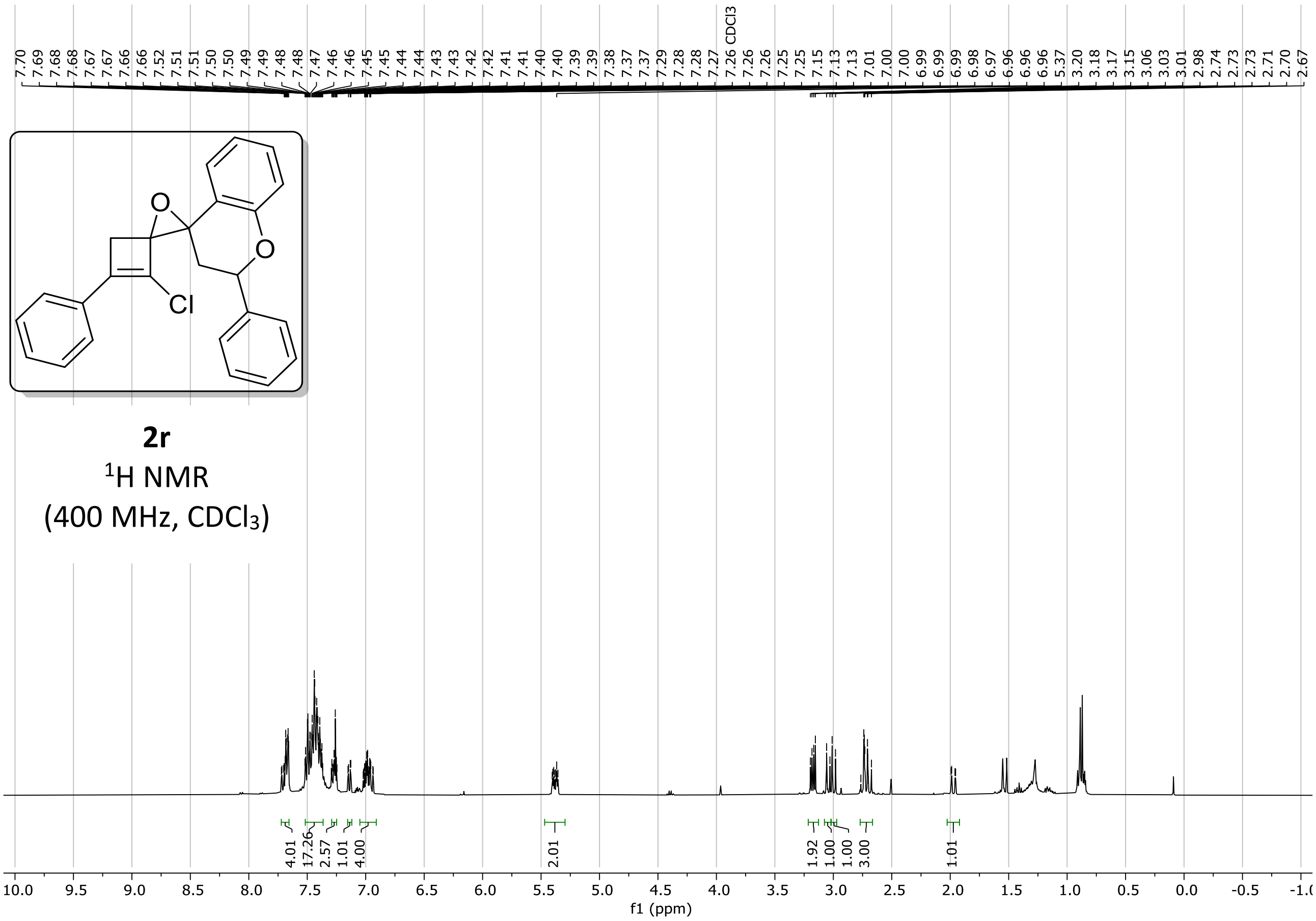

2r
^{1}H NMR
(400 MHz, $CDCl_3$)

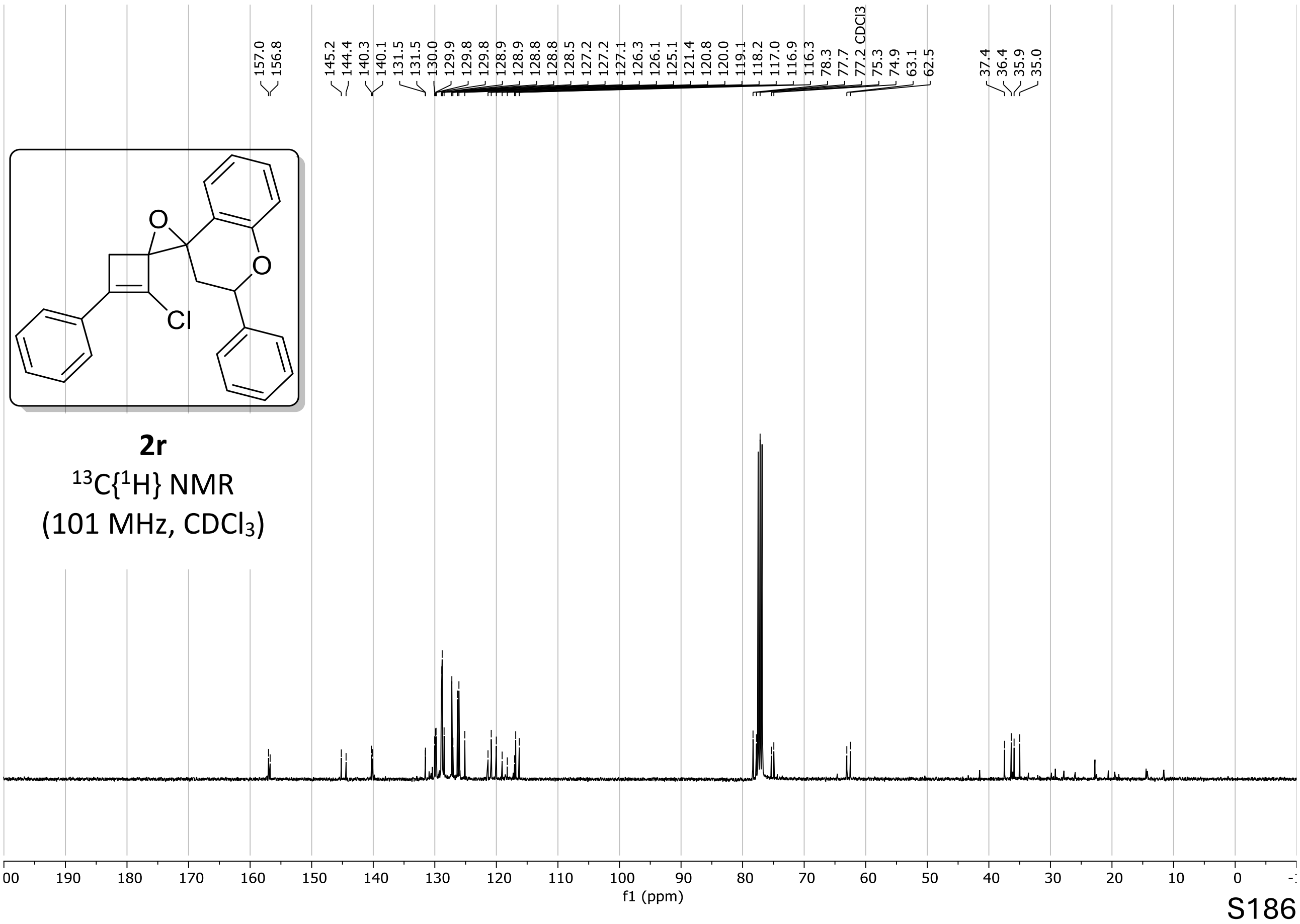

2r
$^{13}C\{^{1}H\}$ NMR
(101 MHz, $CDCl_3$)

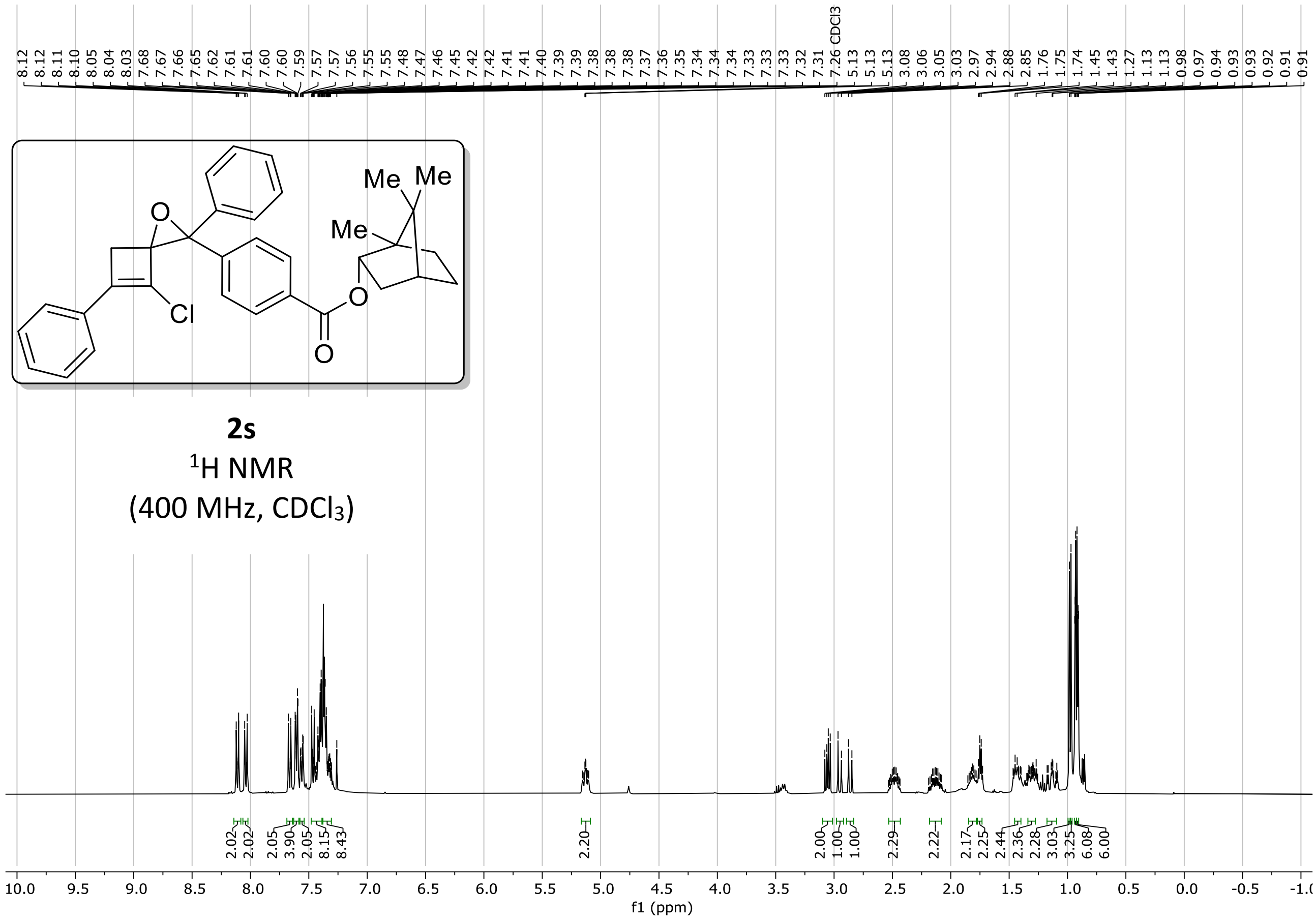

2s
^{1}H NMR
(400 MHz, $CDCl_3$)

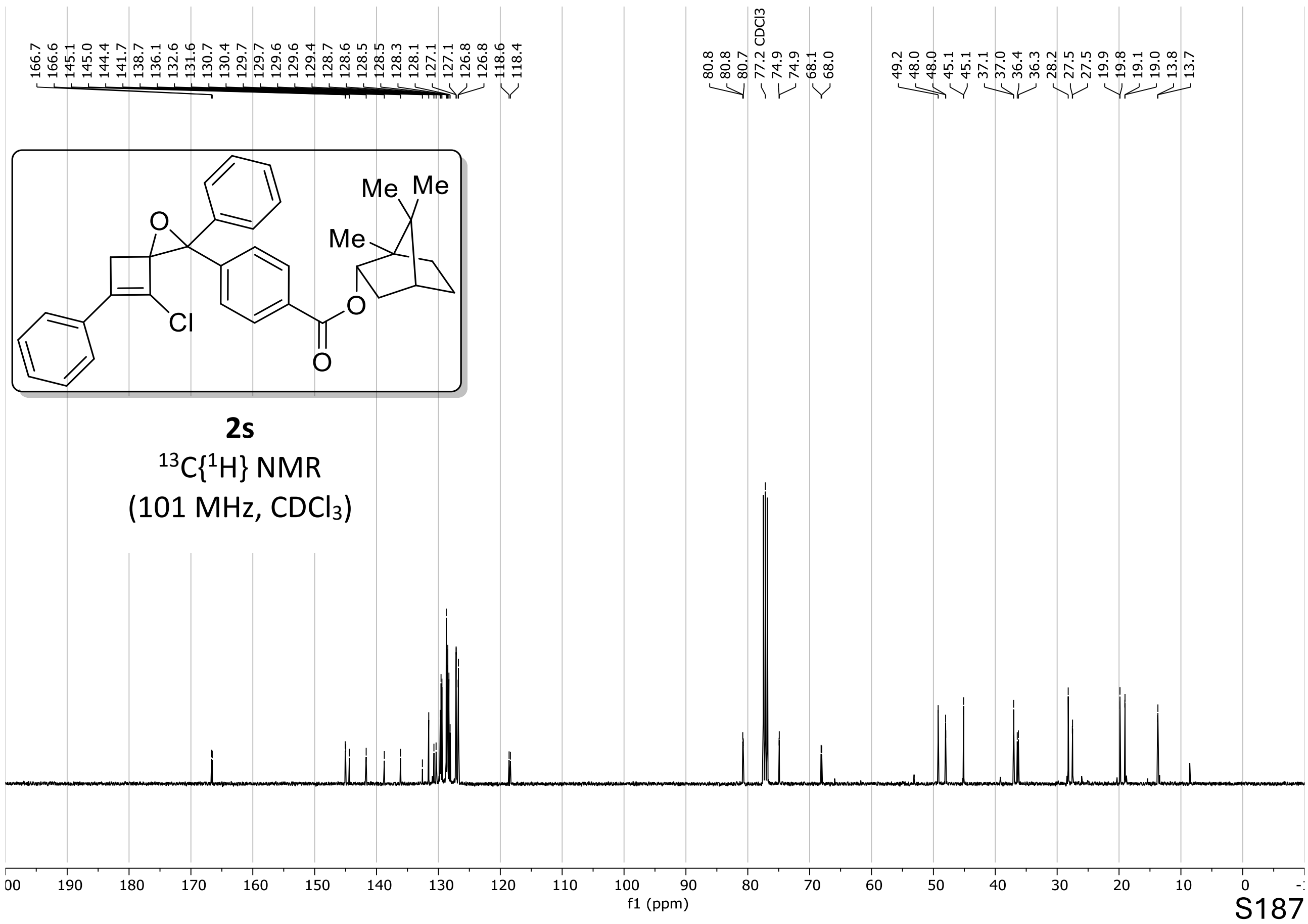

2s
$^{13}C\{^{1}H\}$ NMR
(101 MHz, $CDCl_3$)

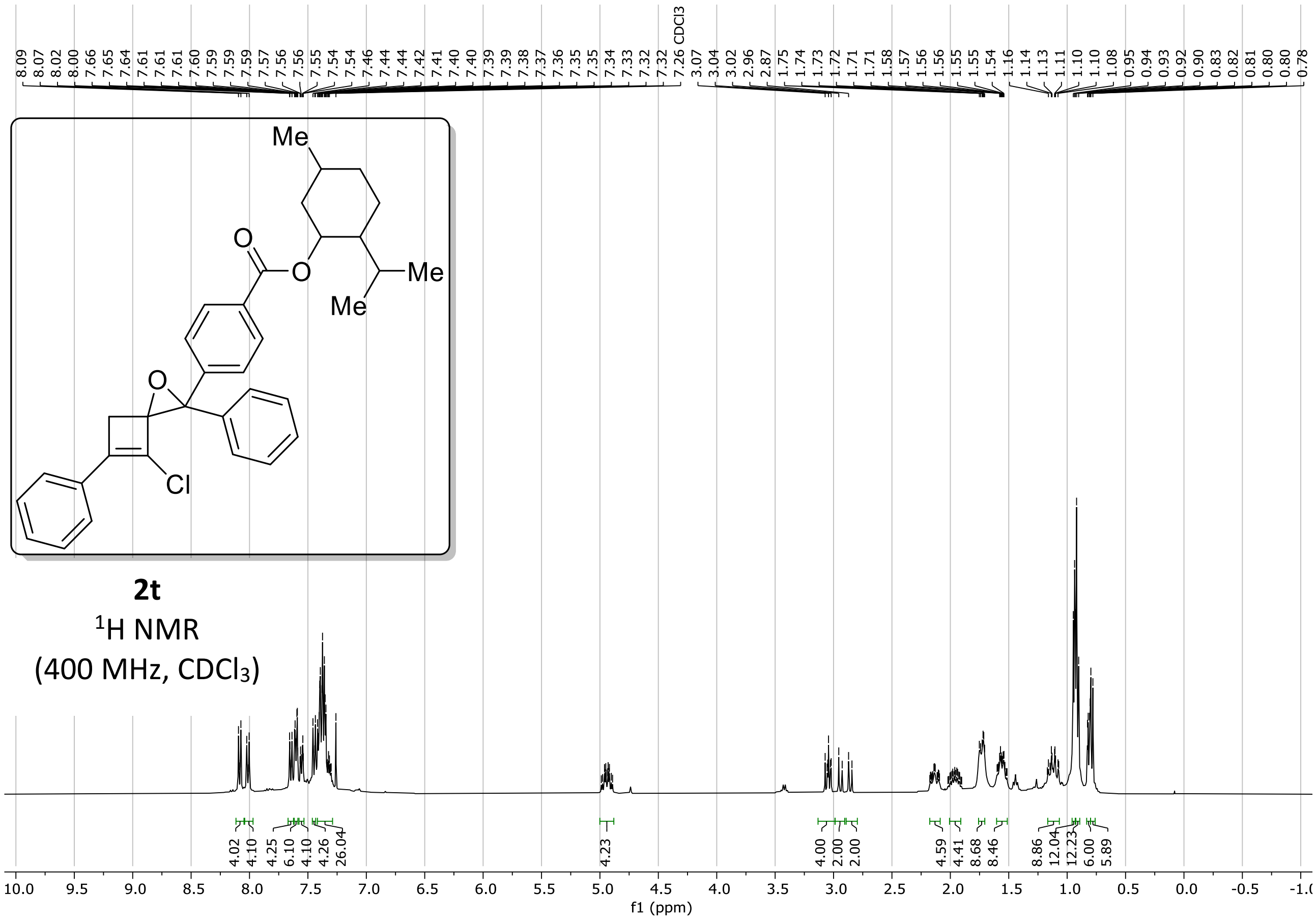

2t

^{1}H NMR

(400 MHz, $CDCl_3$)

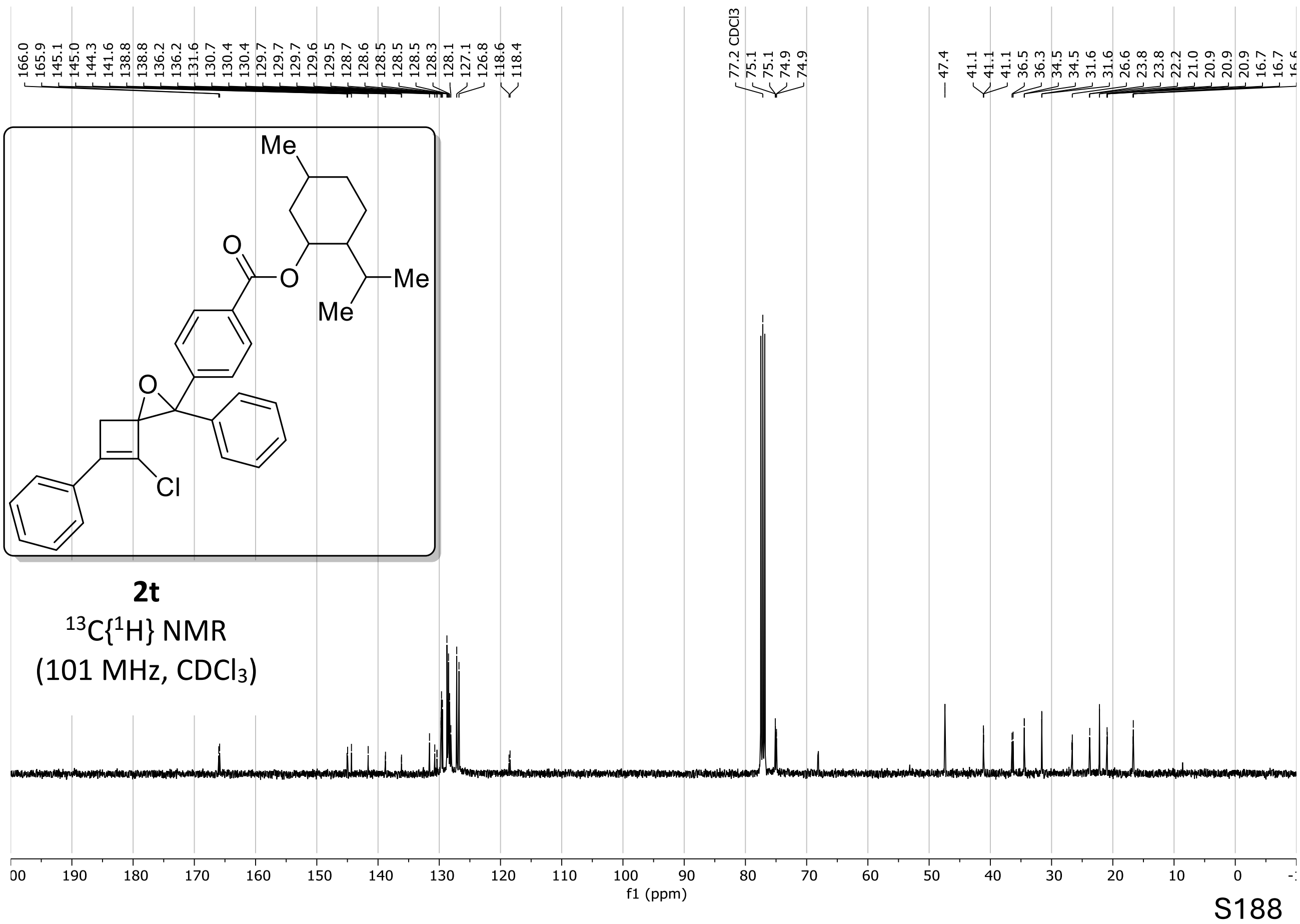

2t

$^{13}C\{^{1}H\}$ NMR

(101 MHz, $CDCl_3$)

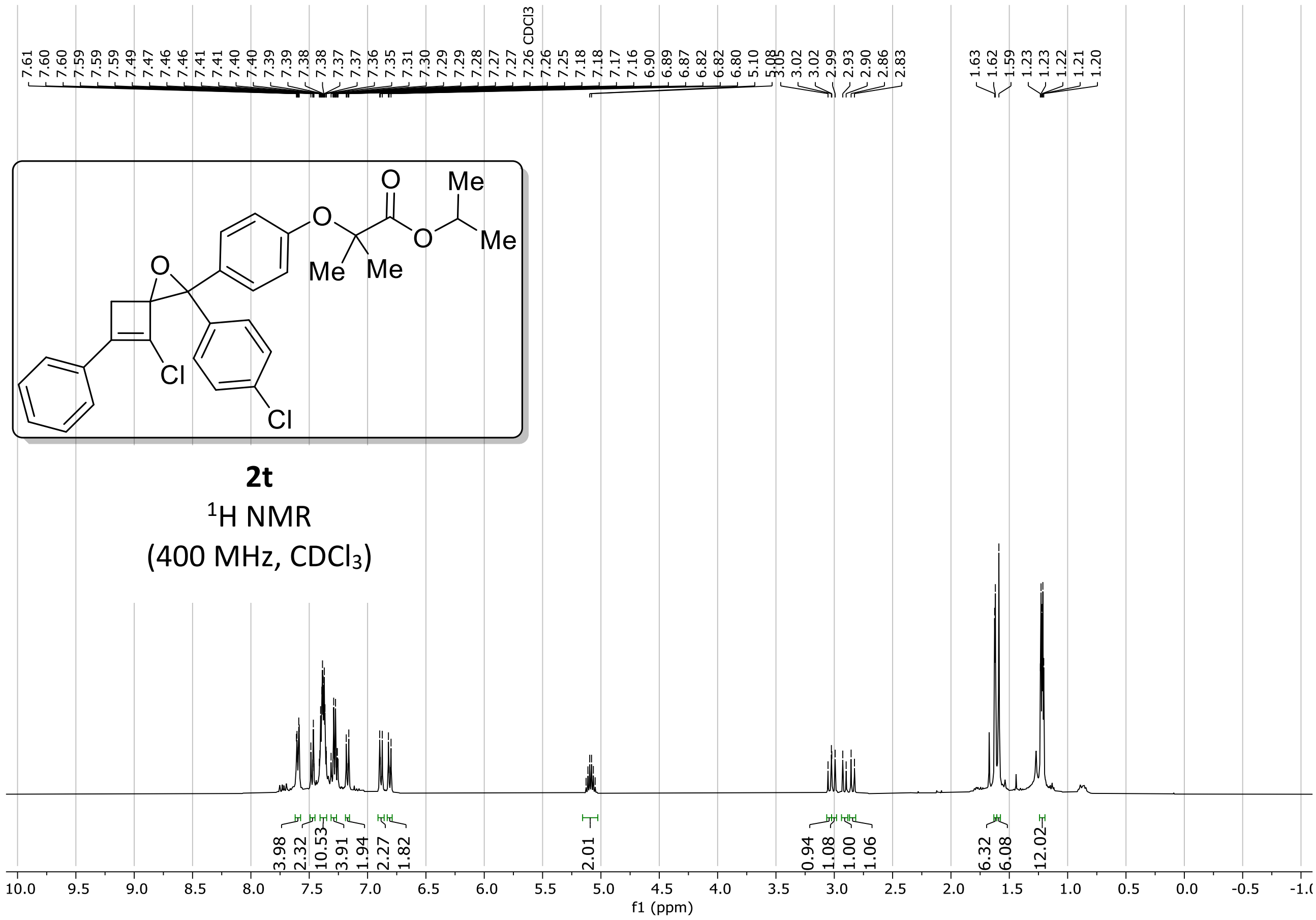

2t
^{1}H NMR
(400 MHz, $CDCl_3$)

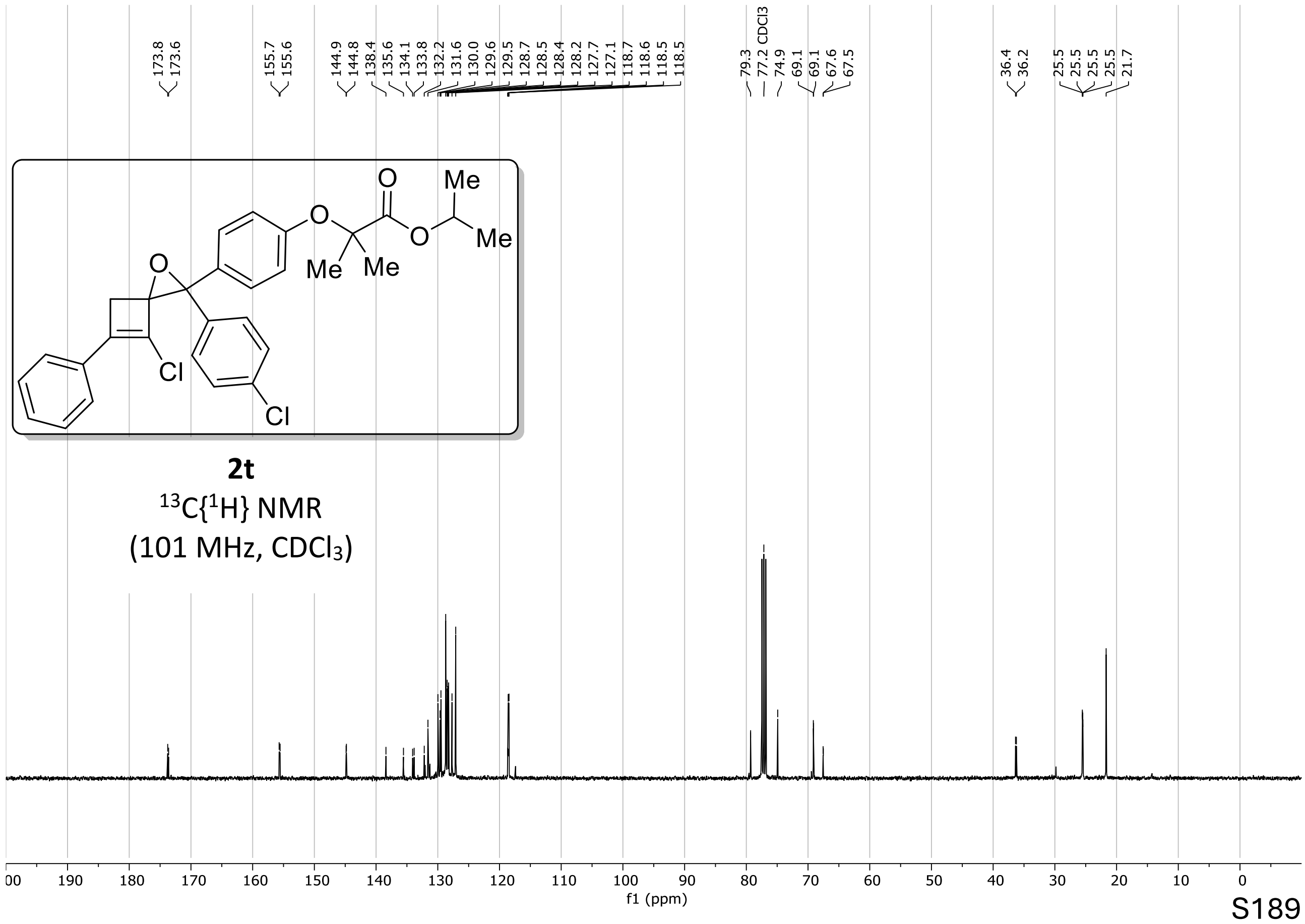

2t
$^{13}C\{^{1}H\}$ NMR
(101 MHz, $CDCl_3$)

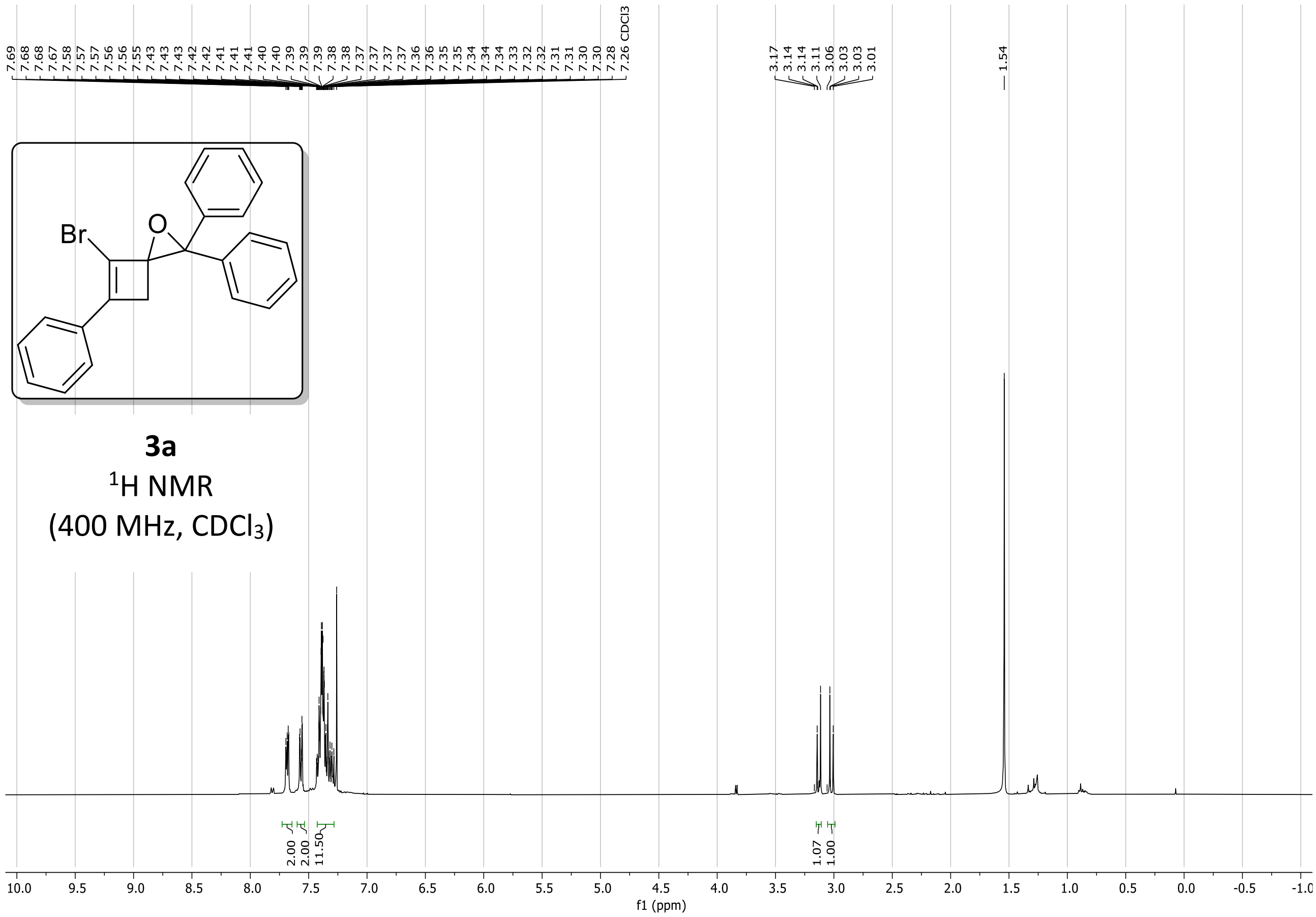

7.69 7.68 7.67 7.67 7.58 7.57 7.57 7.56 7.56 7.55 7.43 7.43 7.43 7.42 7.42 7.41 7.41 7.41 7.40 7.40 7.39 7.39 7.38 7.38 7.37 7.37 7.37 7.37 7.36 7.35 7.35 7.34 7.34 7.33 7.33 7.32 7.32 7.31 7.31 7.30 7.30 7.28 7.26 CDCl3
3.17 3.14 3.14 3.11 3.06 3.03 3.03 3.01
1.54
Br
O
3a
¹H NMR
(400 MHz, CDCl3)
2.00 2.00 11.50
1.07 1.00
10.0 9.5 9.0 8.5 8.0 7.5 7.0 6.5 6.0 5.5 5.0 4.5 4.0 3.5 3.0 2.5 2.0 1.5 1.0 0.5 0.0 -0.5 -1.0
f1 (ppm)

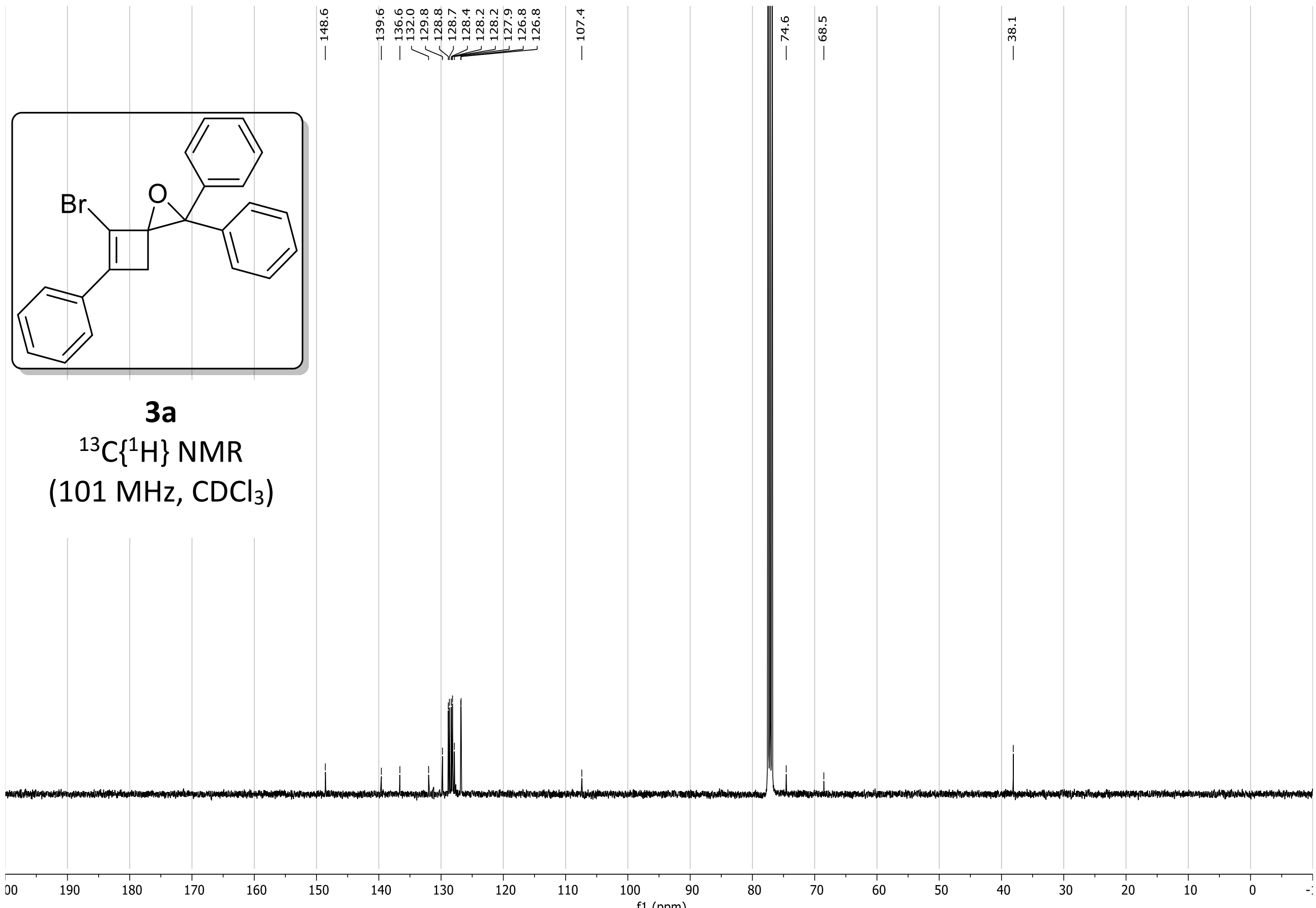

148.6
139.6 136.6 132.0 129.8 128.8 128.7 128.4 128.2 128.2 127.9 126.8 126.8
107.4
74.6
68.5
38.1
Br
O
3a
¹³C{¹H} NMR
(101 MHz, CDCl3)
190 180 170 160 150 140 130 120 110 100 90 80 70 60 50 40 30 20 10 0
f1 (ppm)

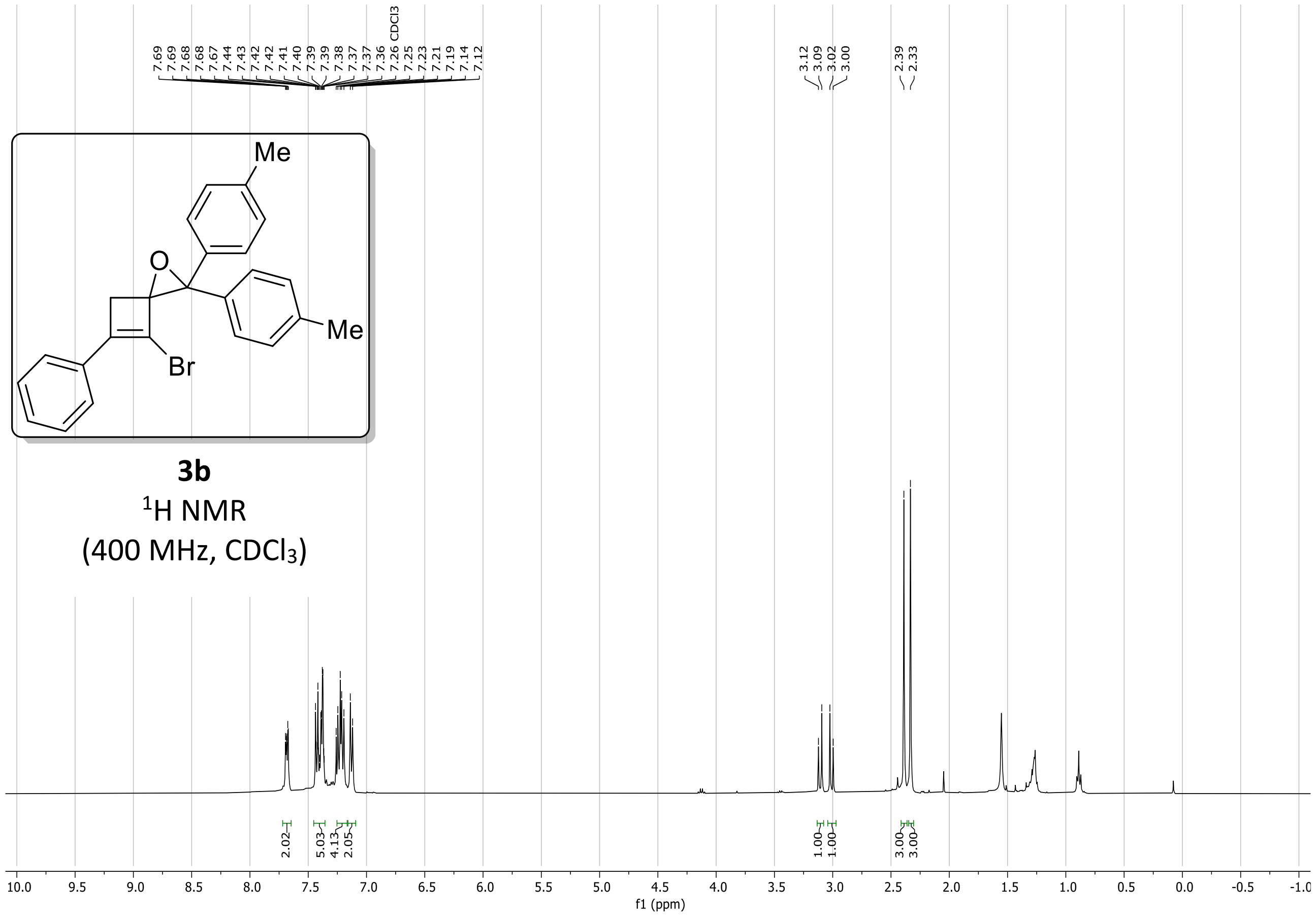
7.69 7.69 7.68 7.68 7.67 7.44 7.43 7.42 7.42 7.41 7.40 7.39 7.39 7.38 7.37 7.37 7.36 7.26 CDCl3 7.25 7.23 7.21 7.19 7.14 7.12
3.12 3.09 3.02 3.00
2.39 2.33
Me
O
Me
Br
3b
^{1}H NMR
(400 MHz, $CDCl_3$)
2.02 5.03 4.13 2.05 1.00 1.00 3.00 3.00
f1 (ppm)

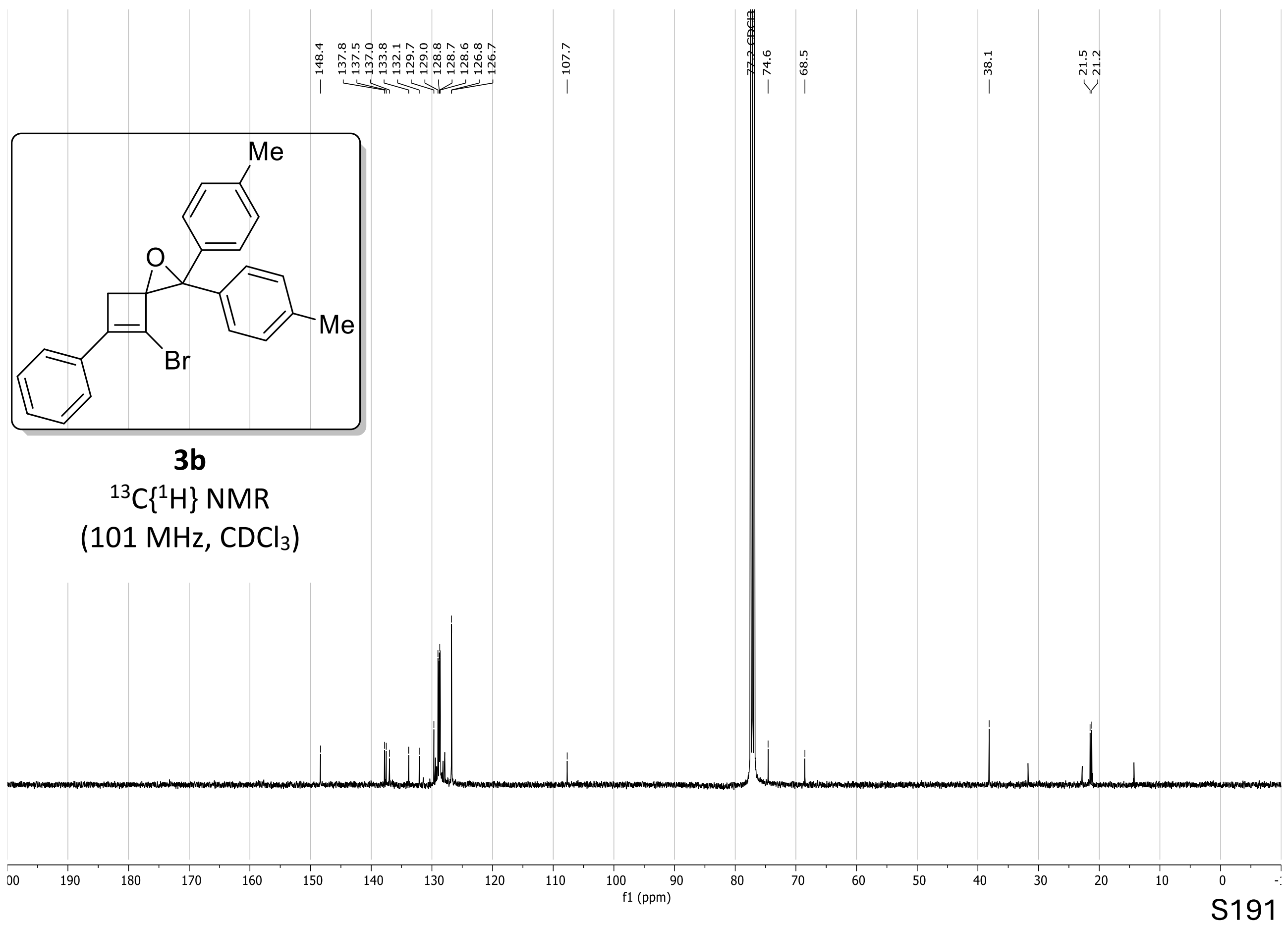
148.4
137.8 137.5 137.0 133.8 132.1 129.7 129.0 128.8 128.7 128.6 126.8 126.7
107.7
77.2 CDCl3
74.6
68.5
38.1
21.5 21.2
Me
O
Me
Br
3b
$^{13}C\{^{1}H\}$ NMR
(101 MHz, $CDCl_3$)
f1 (ppm)

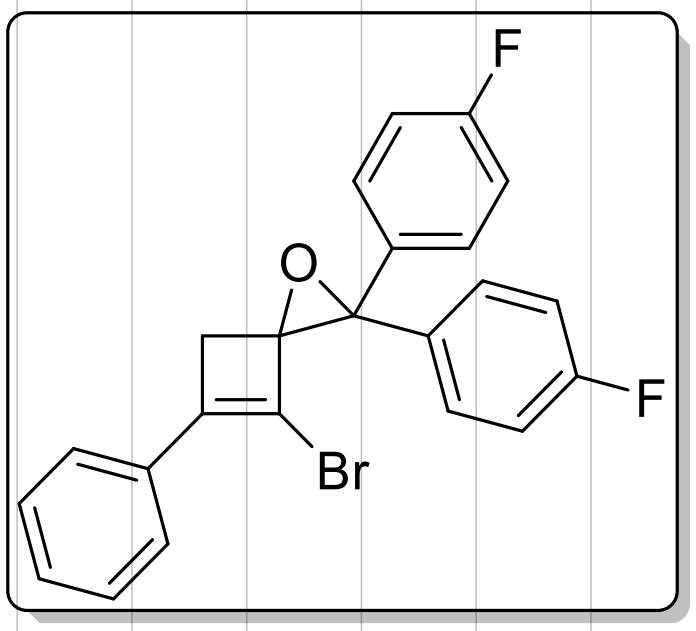

3c
^{1}H NMR
(400 MHz, $CDCl_3$)

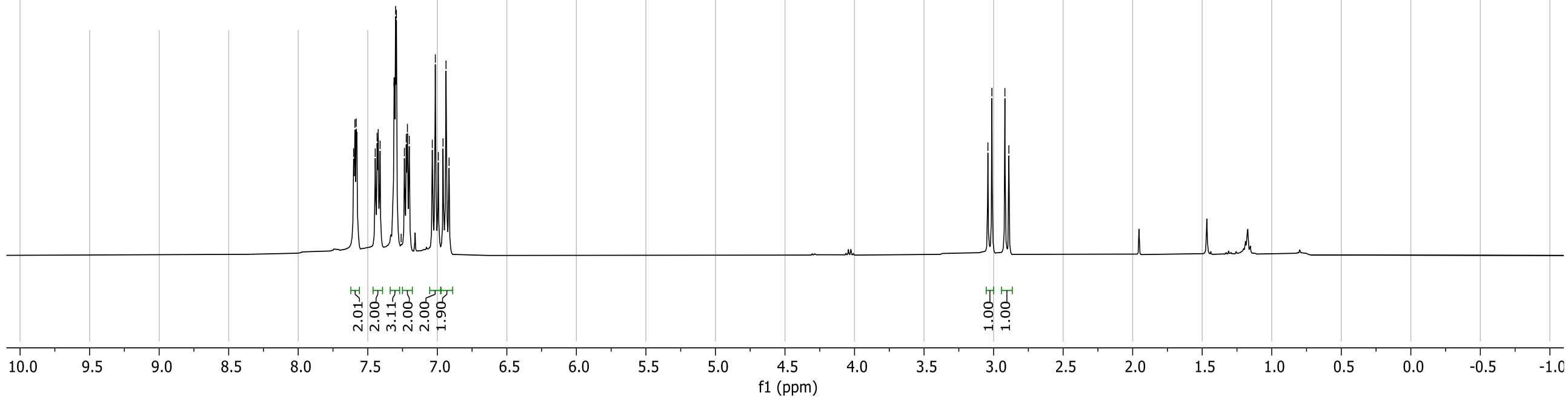

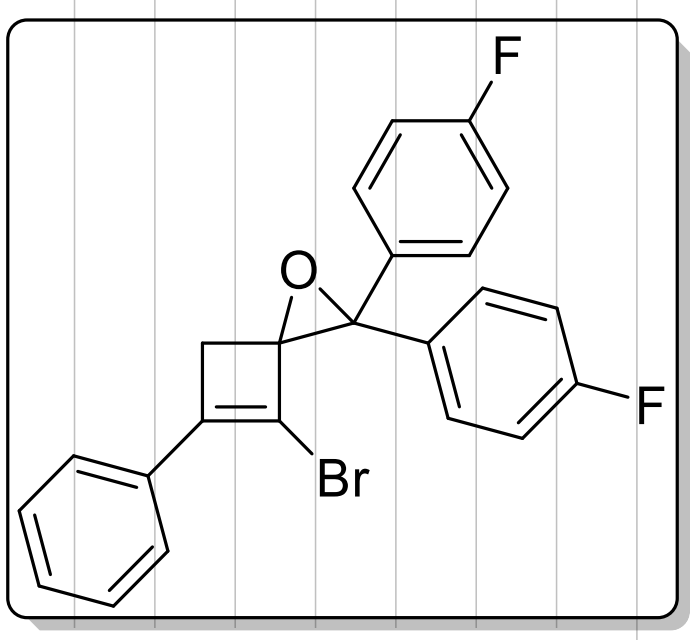

3c
^{19}F NMR
(377 MHz, $CDCl_3$)

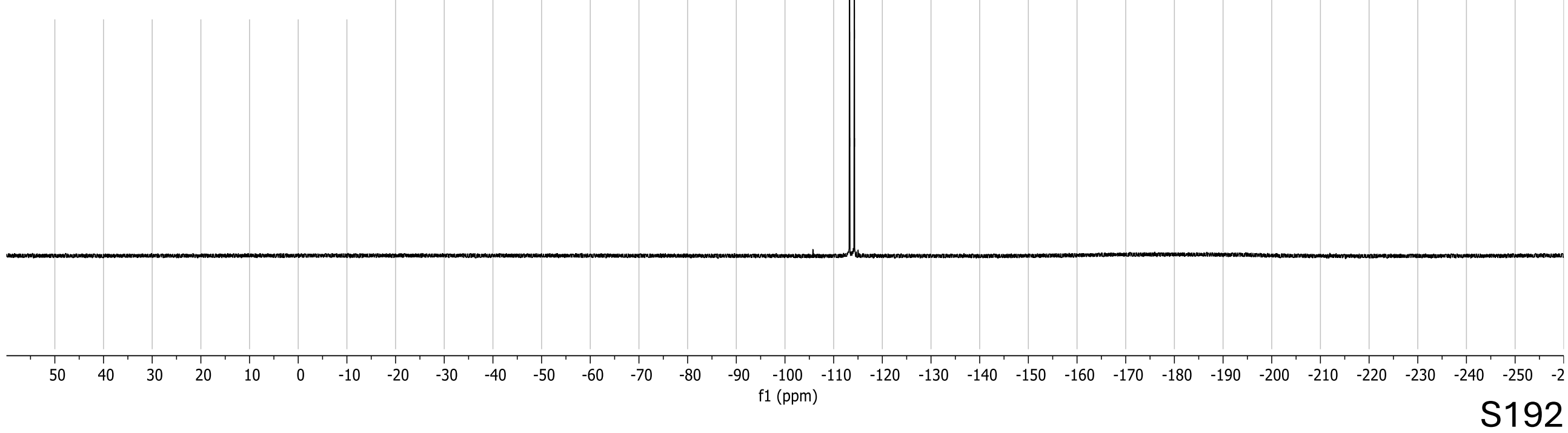

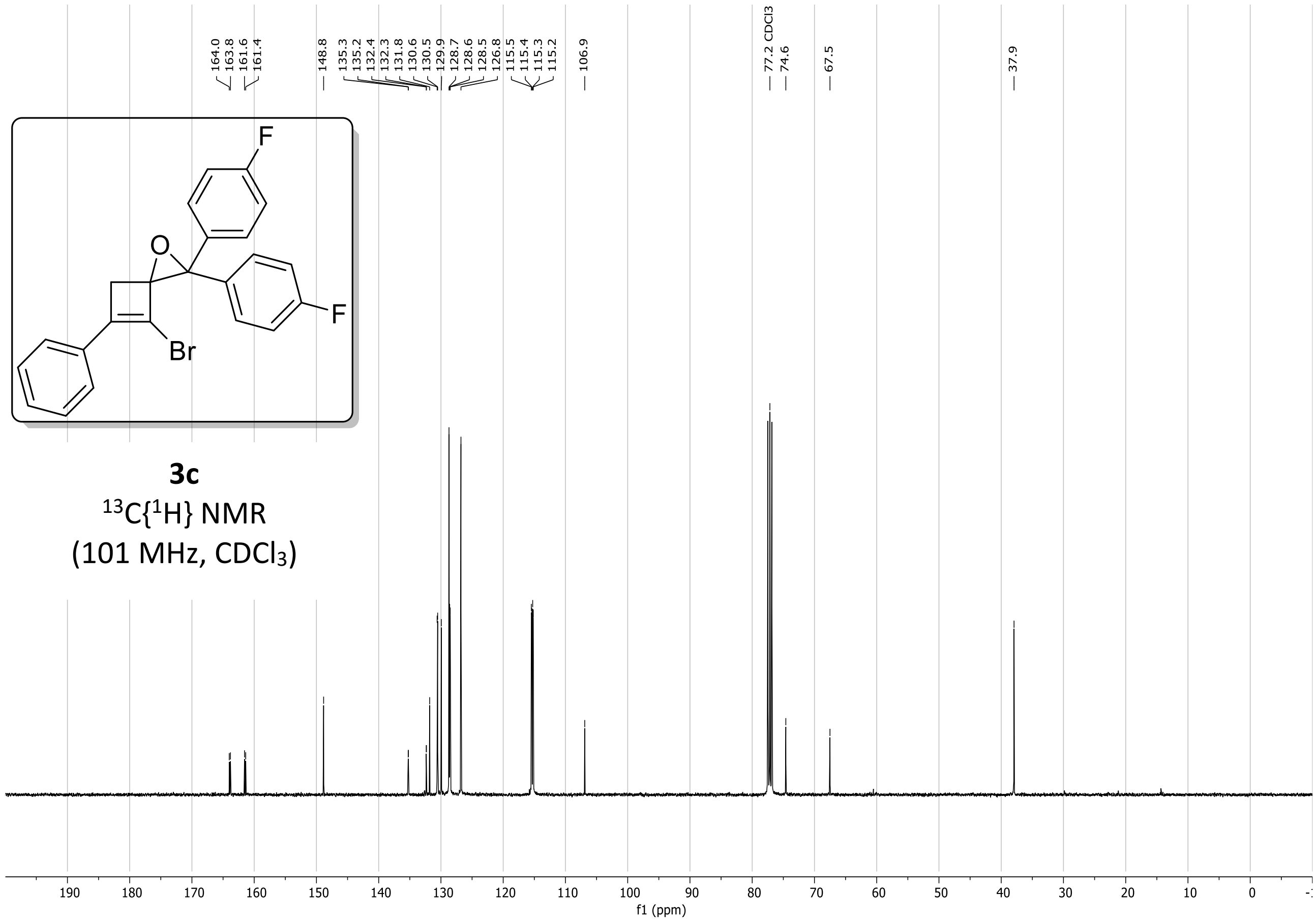

3c

$^{13}C\{^{1}H\}$ NMR

(101 MHz, $CDCl_3$)

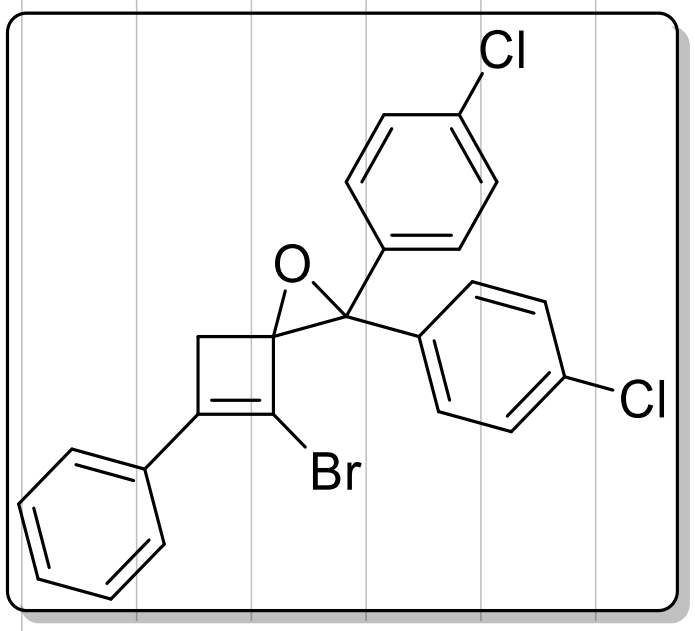

3d
^{1}H NMR
(400 MHz, $CDCl_3$)

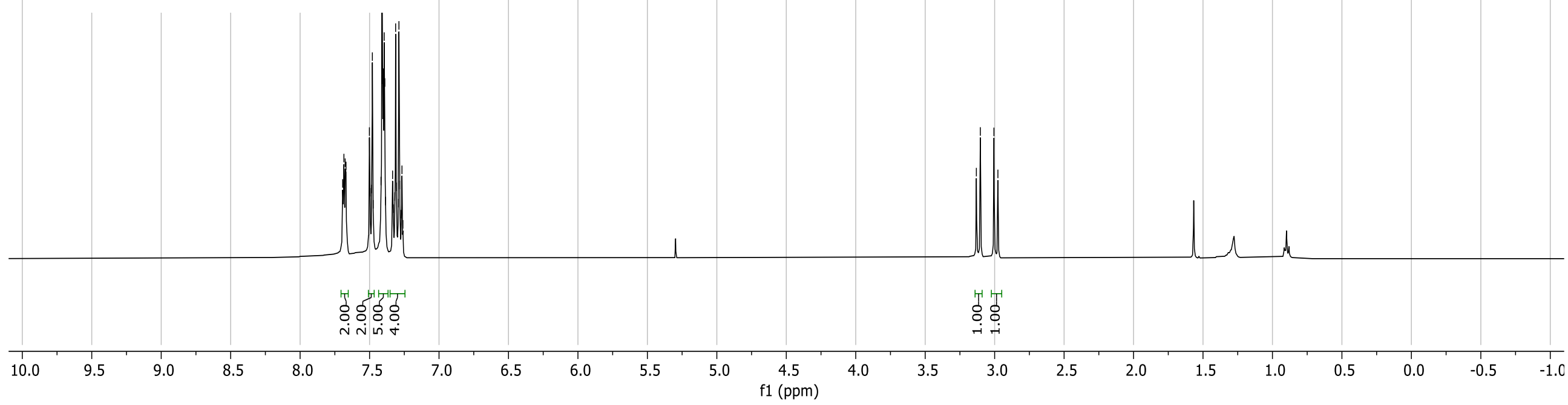

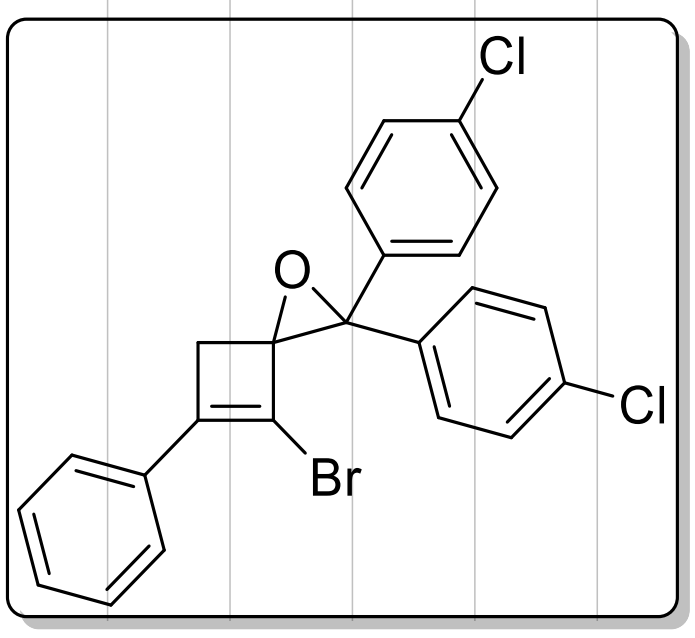

3d
^{13}C{^{1}H} NMR
(101 MHz, $CDCl_3$)

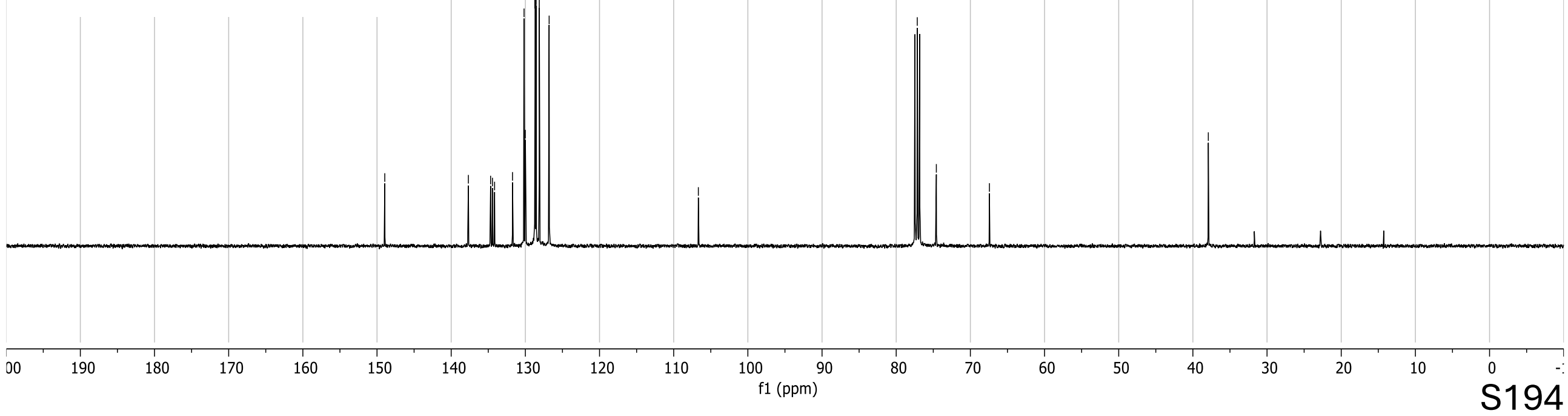

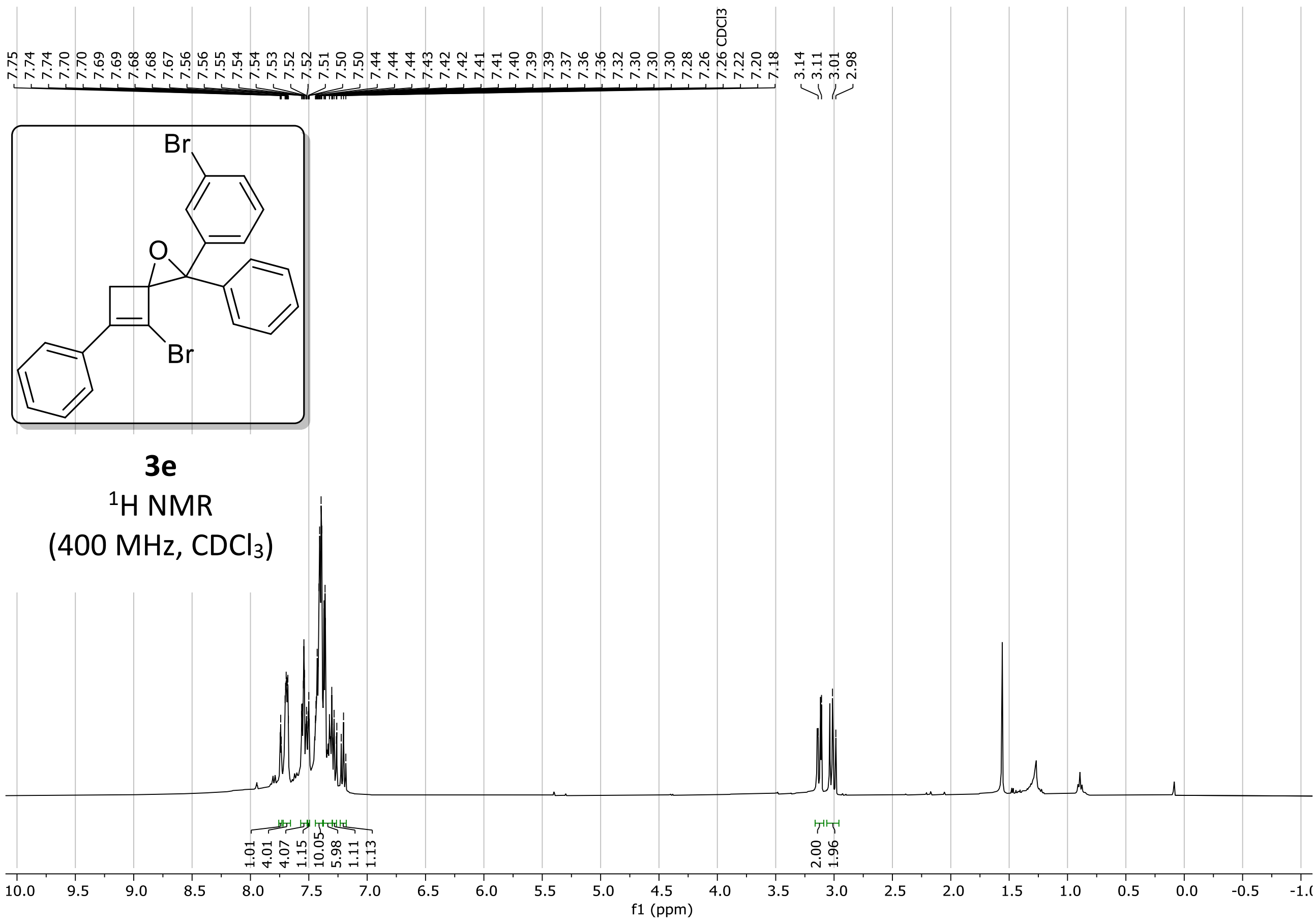

3e

^{1}H NMR

(400 MHz, $CDCl_3$)

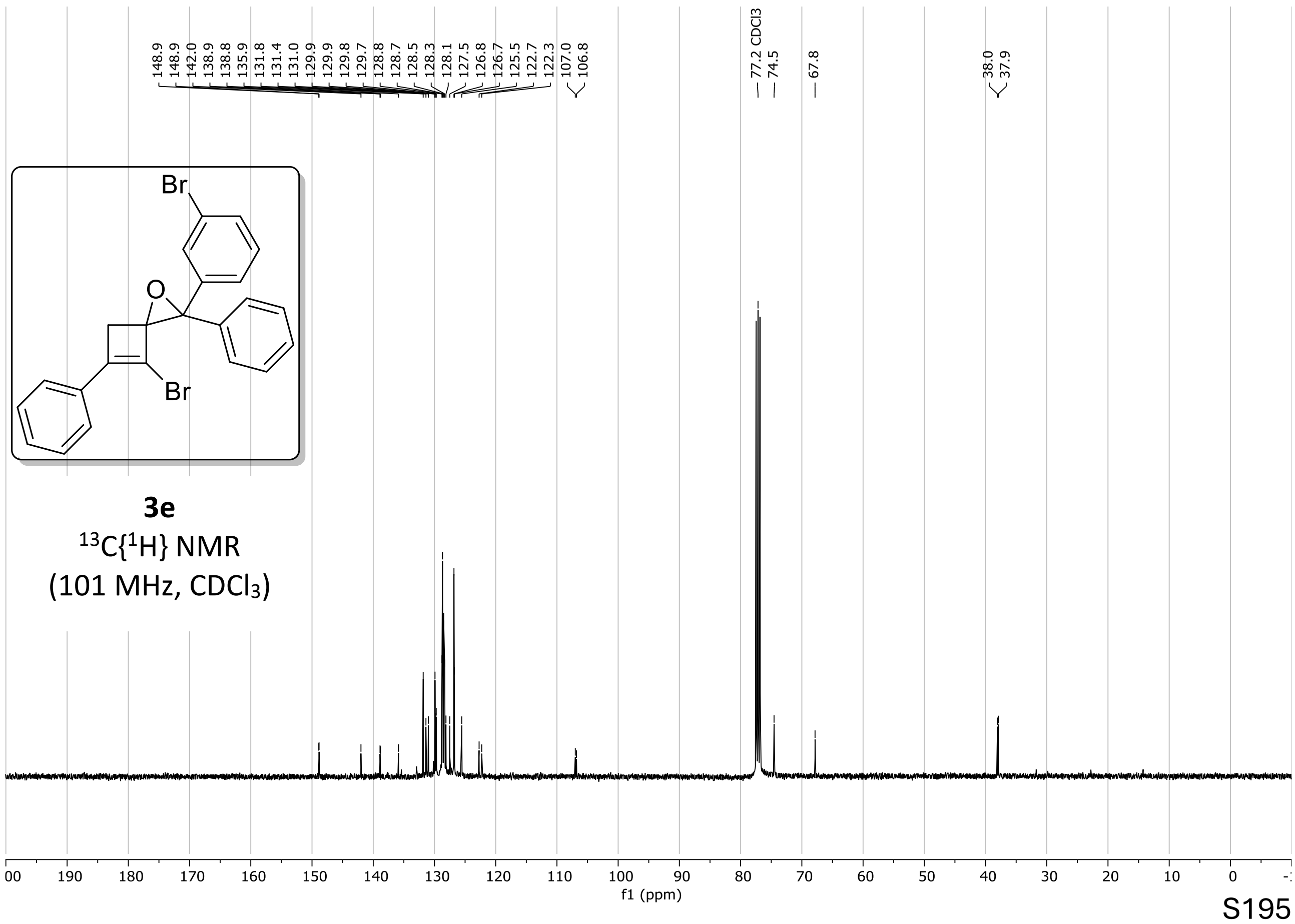

3e

$^{13}C\{^{1}H\}$ NMR

(101 MHz, $CDCl_3$)

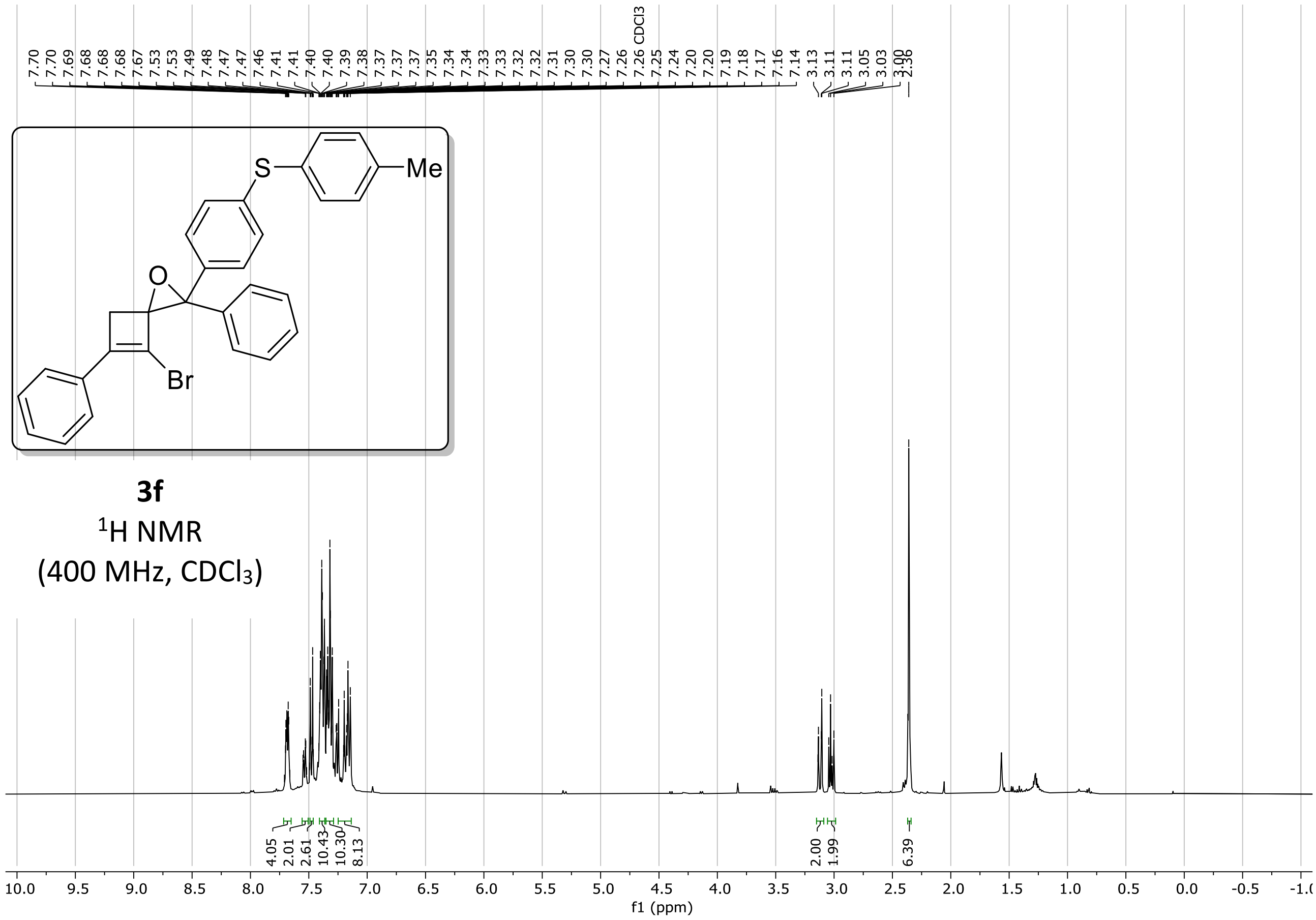
7.70 7.70 7.69 7.68 7.68 7.68 7.67 7.53 7.53 7.49 7.48 7.47 7.47 7.46 7.41 7.41 7.40 7.40 7.39 7.38 7.37 7.37 7.37 7.35 7.34 7.34 7.33 7.33 7.32 7.32 7.31 7.30 7.30 7.27 7.26 7.26 CDCl3 7.25 7.24 7.20 7.20 7.19 7.18 7.17 7.16 7.14 3.13 3.11 3.11 3.05 3.03 3.00 2.38
S
Me
O
Br
3f
^{1}H NMR
(400 MHz, CDCl$_3$)
4.05 2.01 2.61 10.43 10.30 8.13
2.00 1.99
6.39
f1 (ppm)

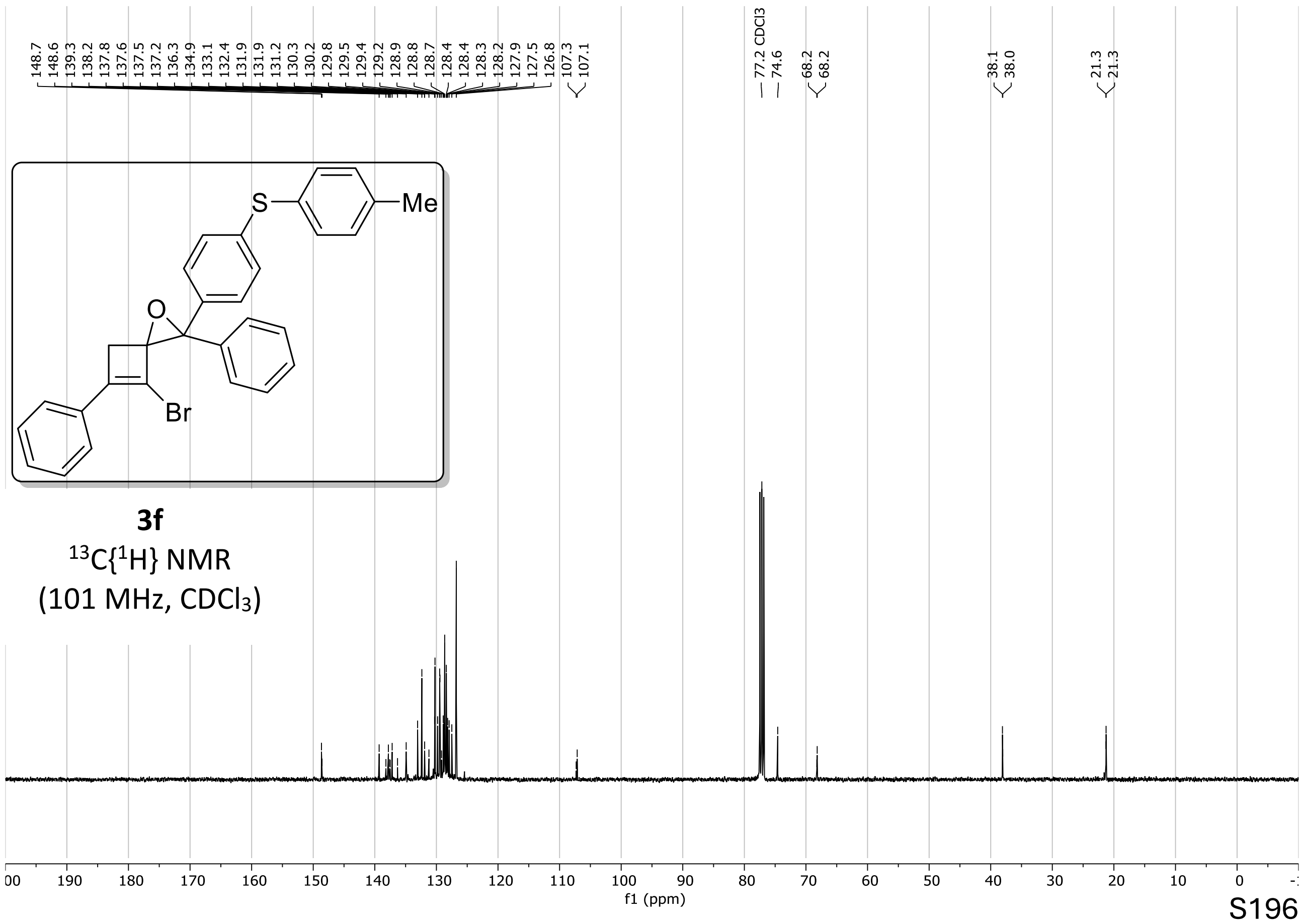
148.7 148.6 139.3 138.2 137.8 137.6 137.5 137.2 136.3 134.9 133.1 132.4 131.9 131.9 131.2 130.3 130.2 129.8 129.5 129.4 129.2 128.9 128.8 128.7 128.4 128.4 128.3 128.2 127.9 127.5 126.8 107.3 107.1
77.2 CDCl3 74.6
68.2 68.2
38.1 38.0
21.3 21.3
S
Me
O
Br
3f
^{13}C{^{1}H} NMR
(101 MHz, CDCl$_3$)
f1 (ppm)

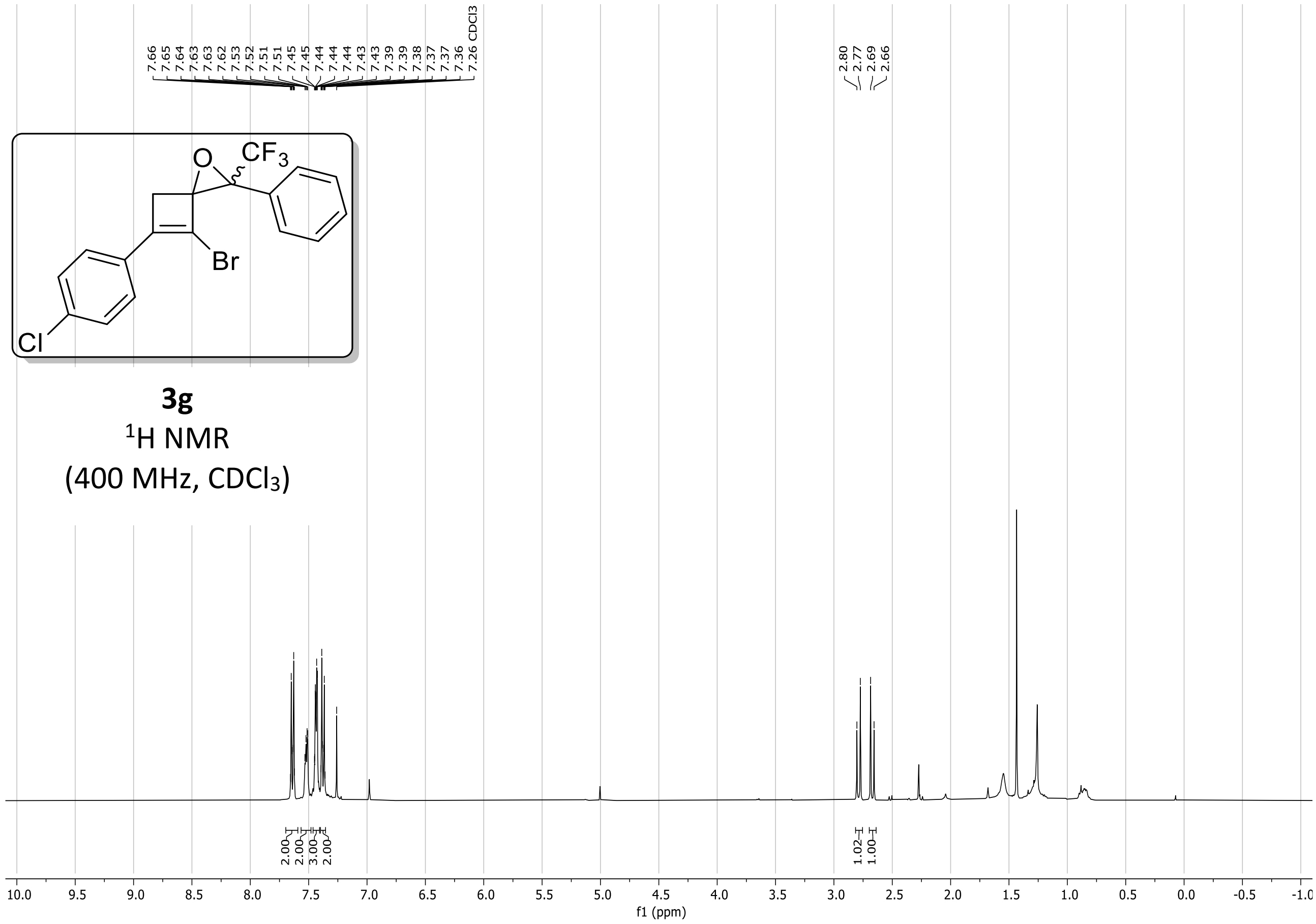
7.66
7.65
7.64
7.63
7.63
7.62
7.53
7.52
7.51
7.51
7.45
7.45
7.44
7.44
7.44
7.43
7.43
7.39
7.39
7.38
7.37
7.37
7.36
7.26 CDCl3
2.80
2.77
2.69
2.66
O
CF3
Br
Cl
3g
1H NMR
(400 MHz, CDCl3)
2.00
2.00
3.00
2.00
1.02
1.00
f1 (ppm)

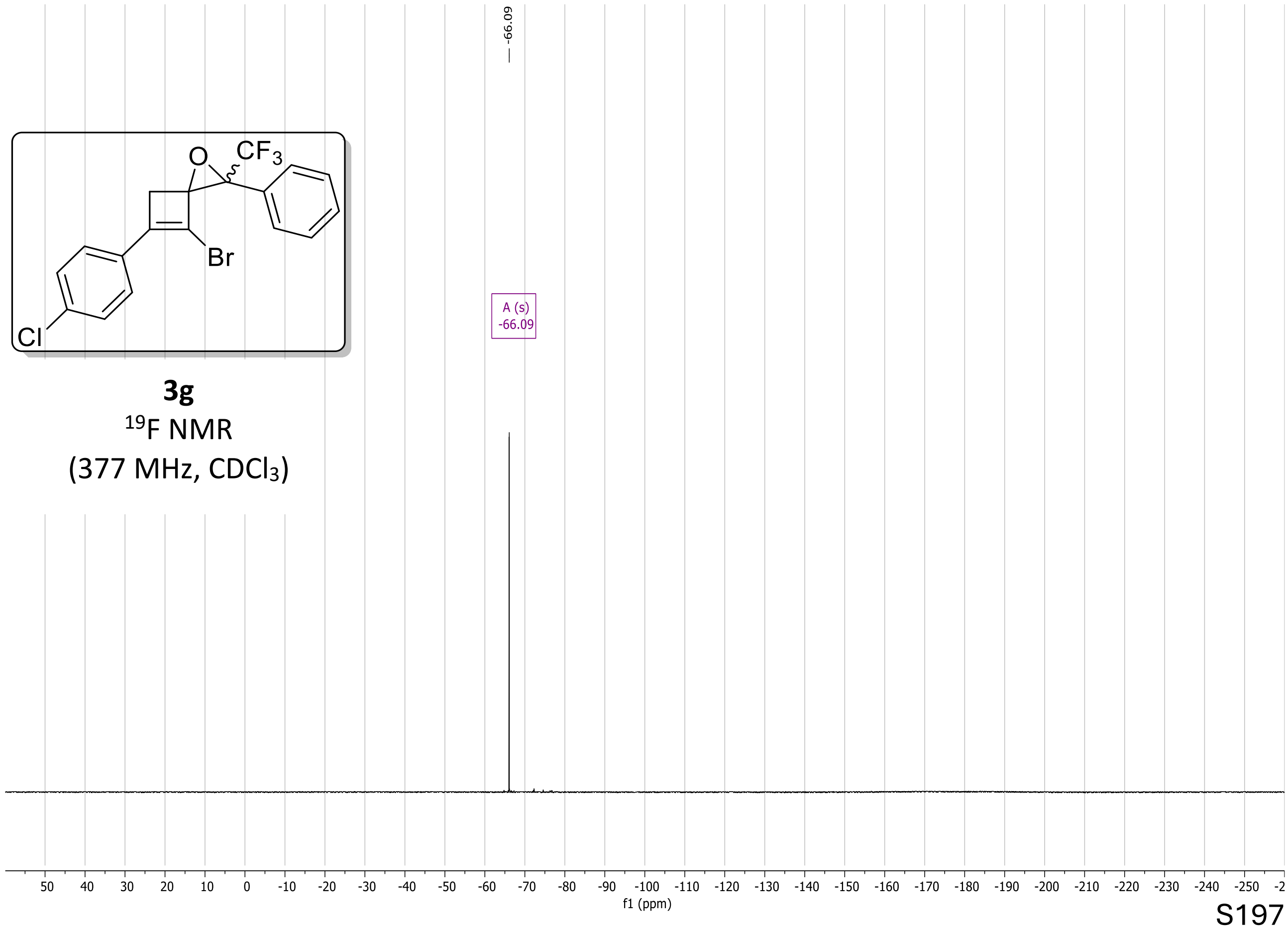
-66.09
O
CF3
Br
Cl
3g
19F NMR
(377 MHz, CDCl3)
A (s)
-66.09
f1 (ppm)

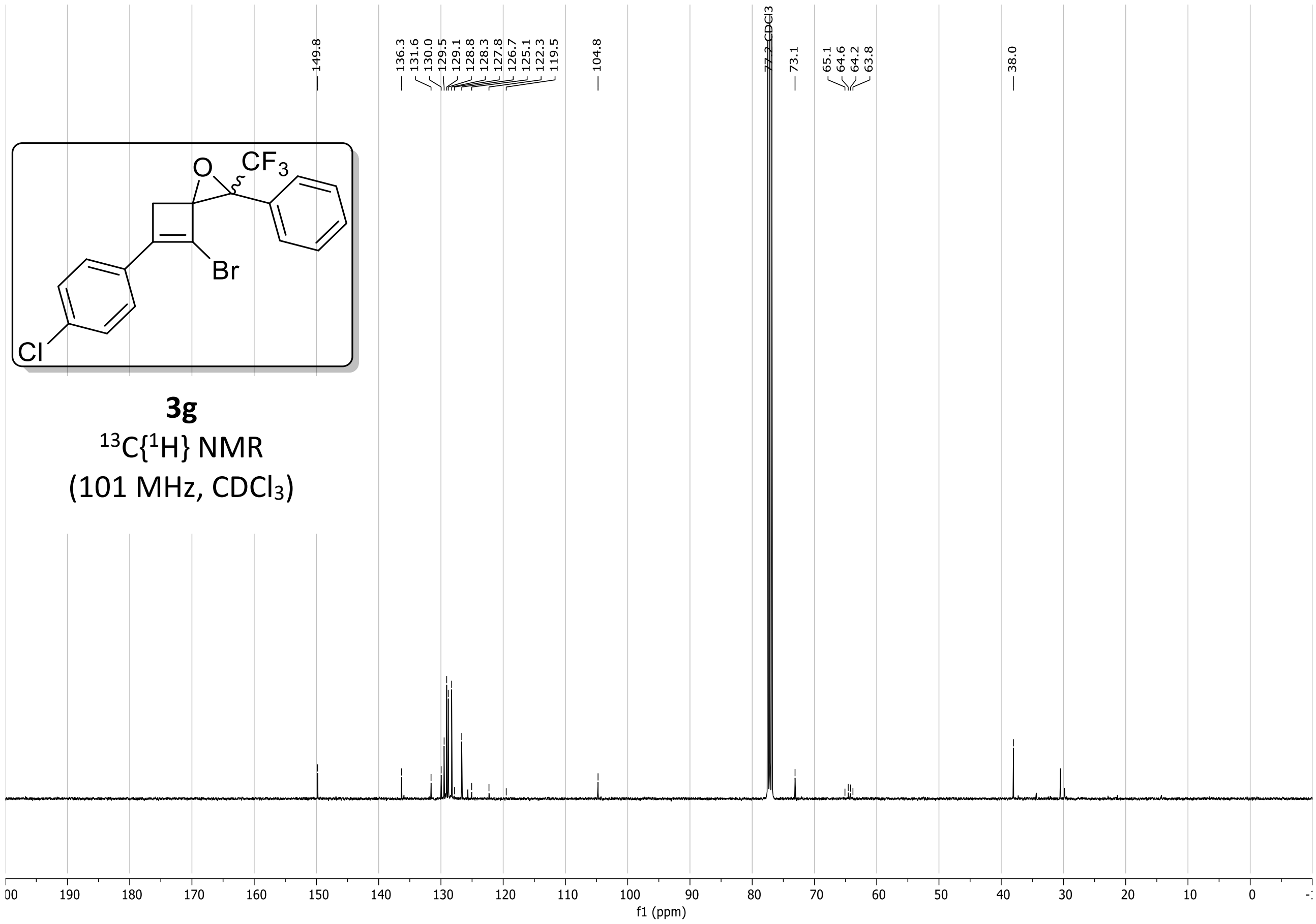
CF3
O
Br
Cl
3g
13C{1H} NMR
(101 MHz, CDCl3)
149.8
136.3
131.6
130.0
129.5
129.1
128.8
128.3
127.8
126.7
125.1
122.3
119.5
104.8
77.2 CDCl3
73.1
65.1
64.6
64.2
63.8
38.0
f1 (ppm)

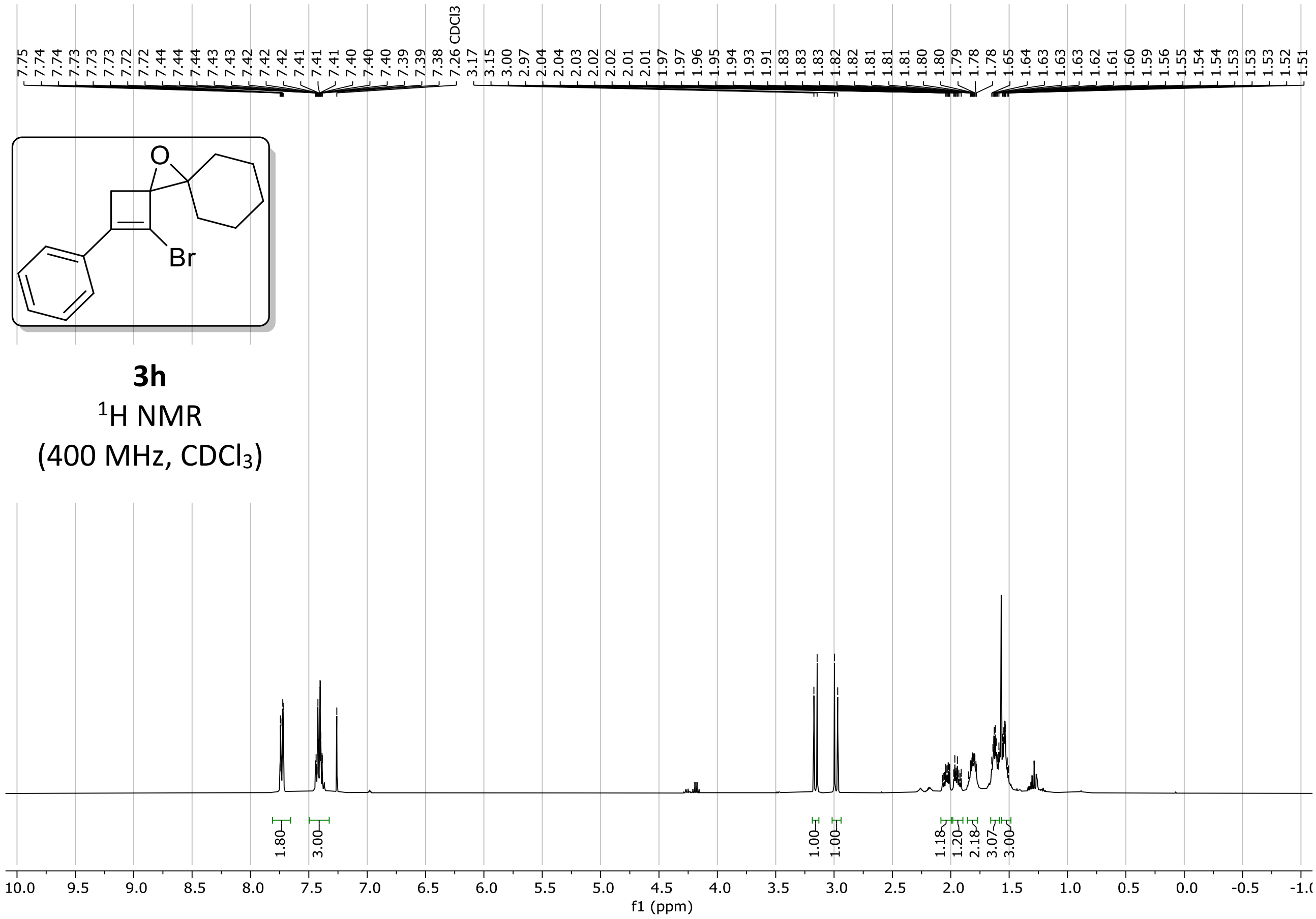

3h
^{1}H NMR
(400 MHz, $CDCl_3$)

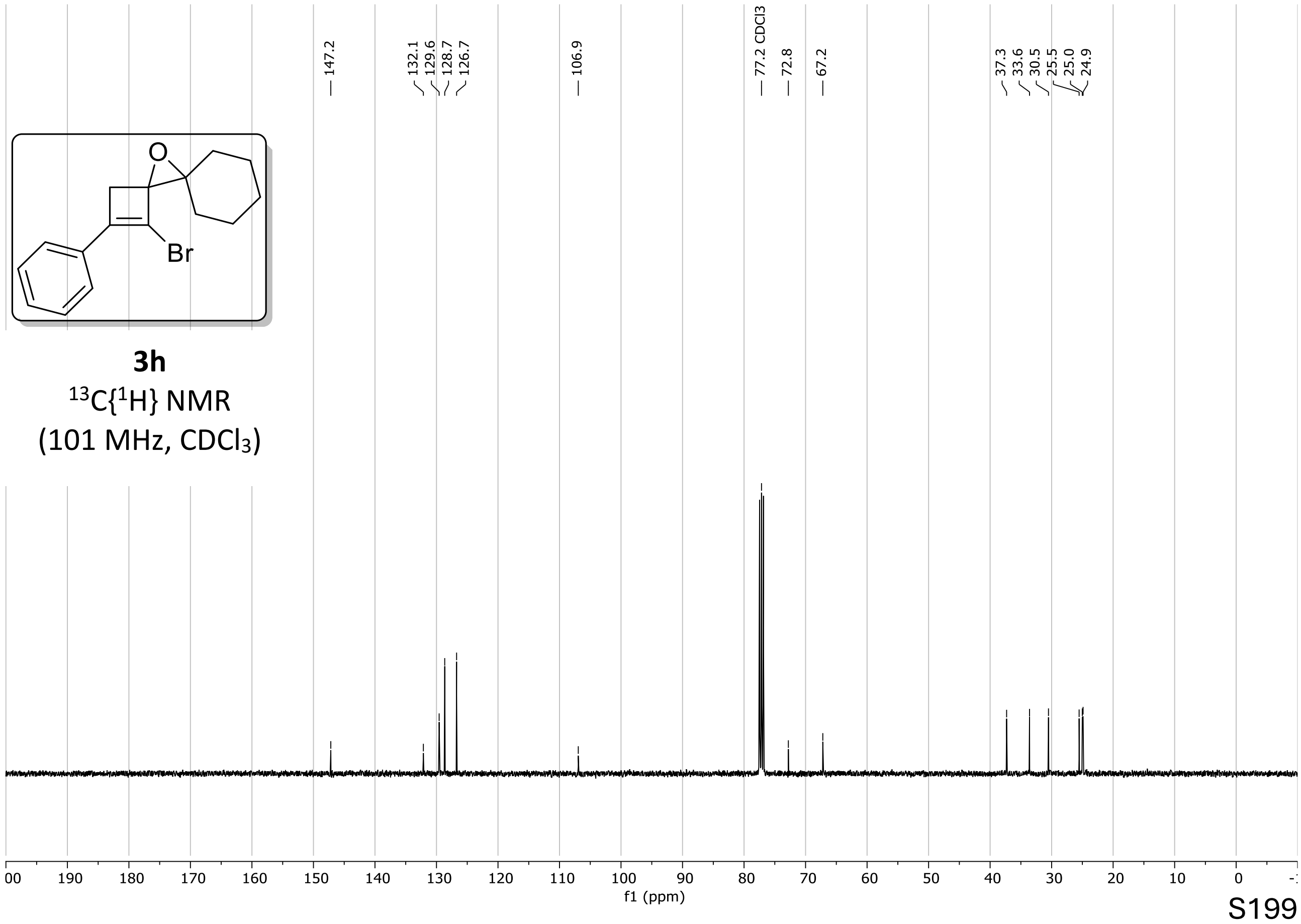

3h
$^{13}C\{^{1}H\}$ NMR
(101 MHz, $CDCl_3$)

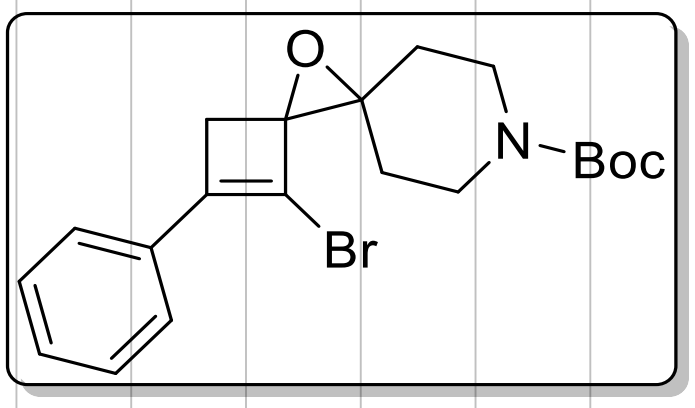

3i

^{1}H NMR

(400 MHz, $CDCl_3$)

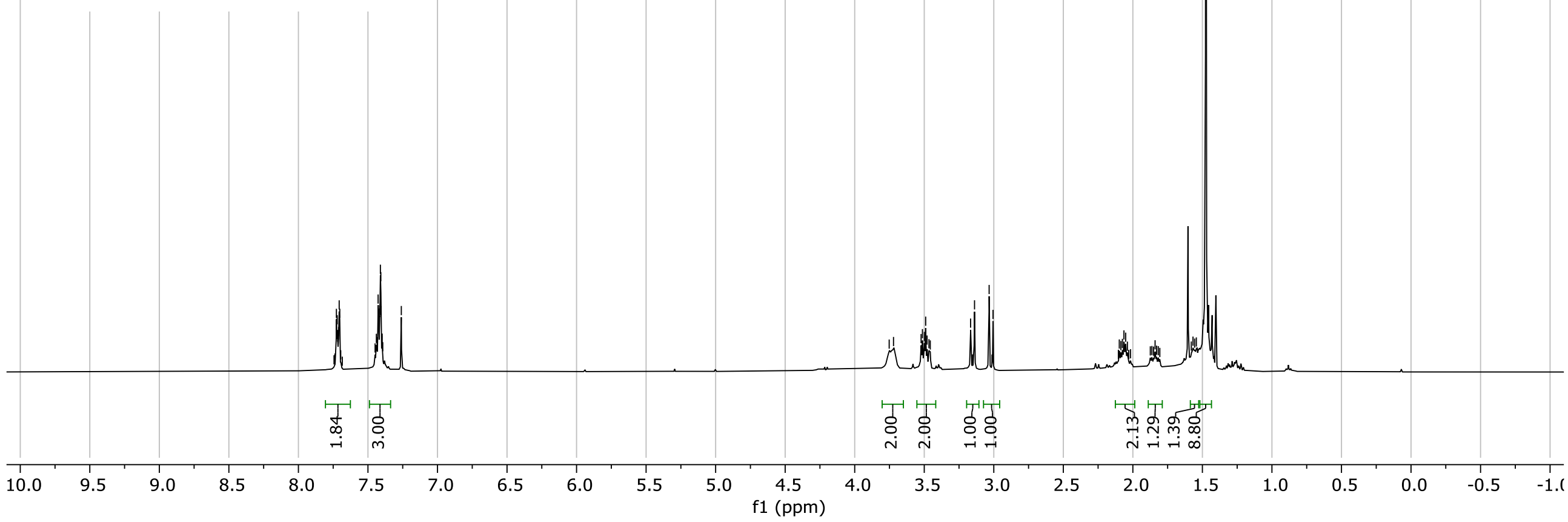

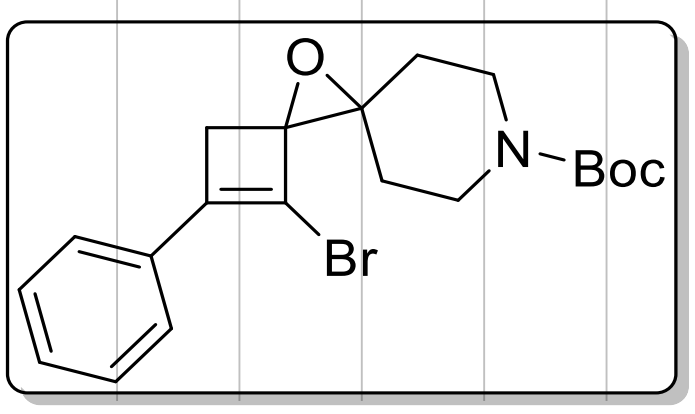

3i

$^{13}C\{^1H\}$ NMR

(101 MHz, $CDCl_3$)

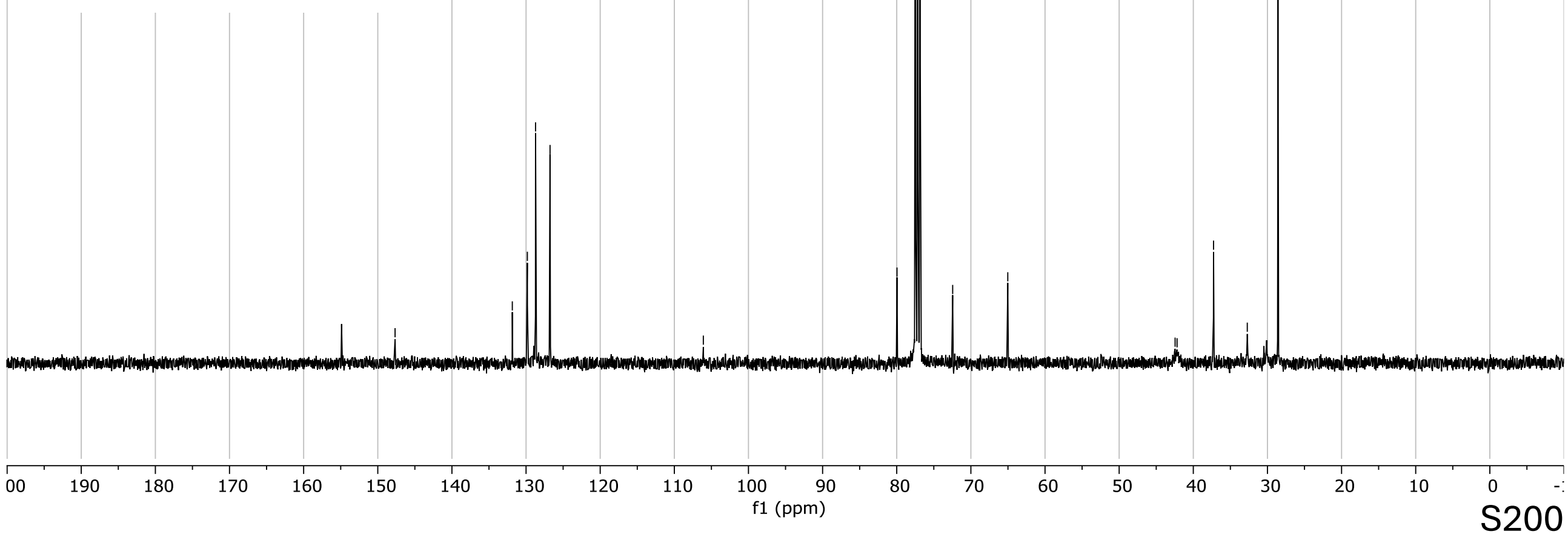

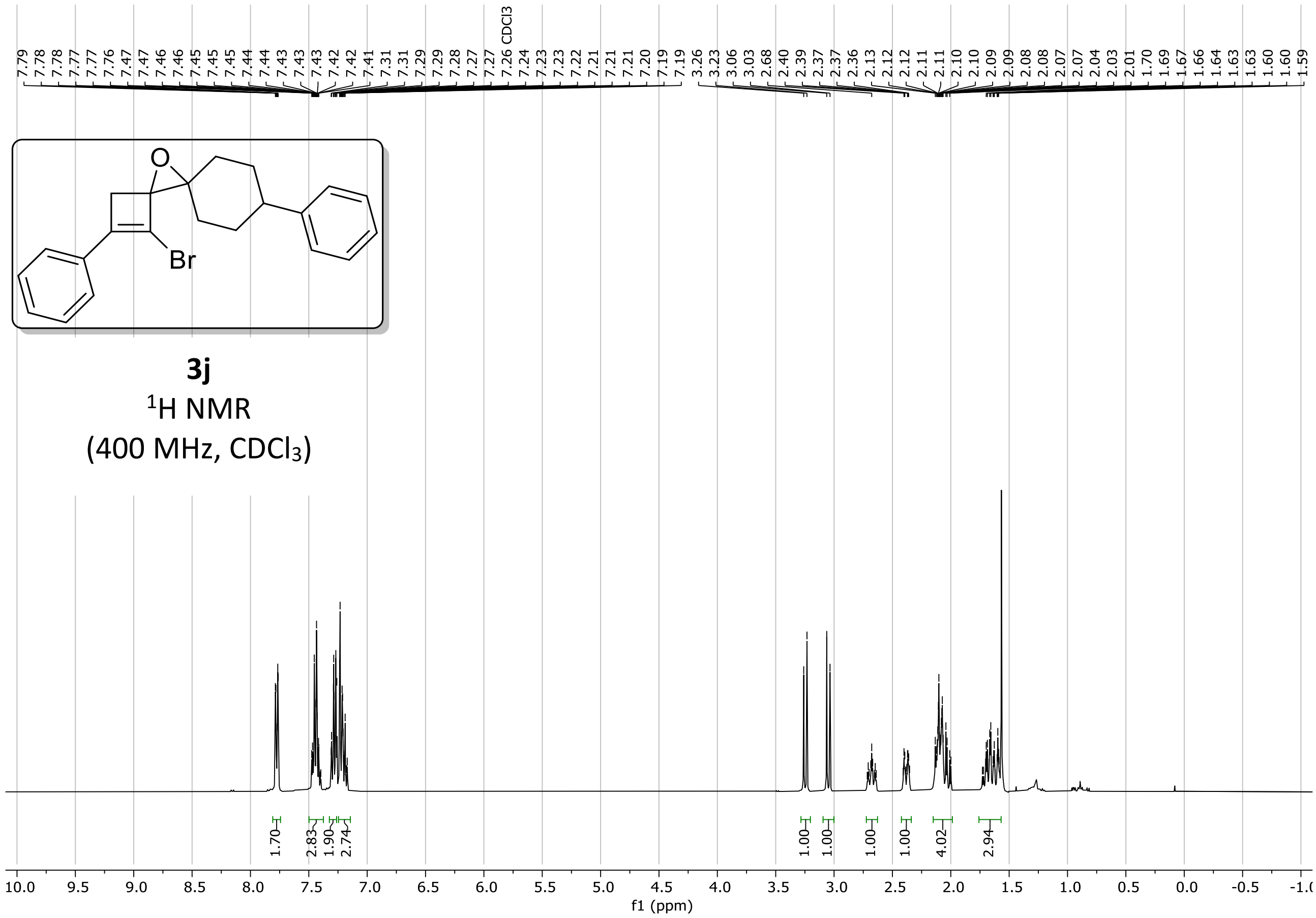

3j
^{1}H NMR
(400 MHz, $CDCl_3$)

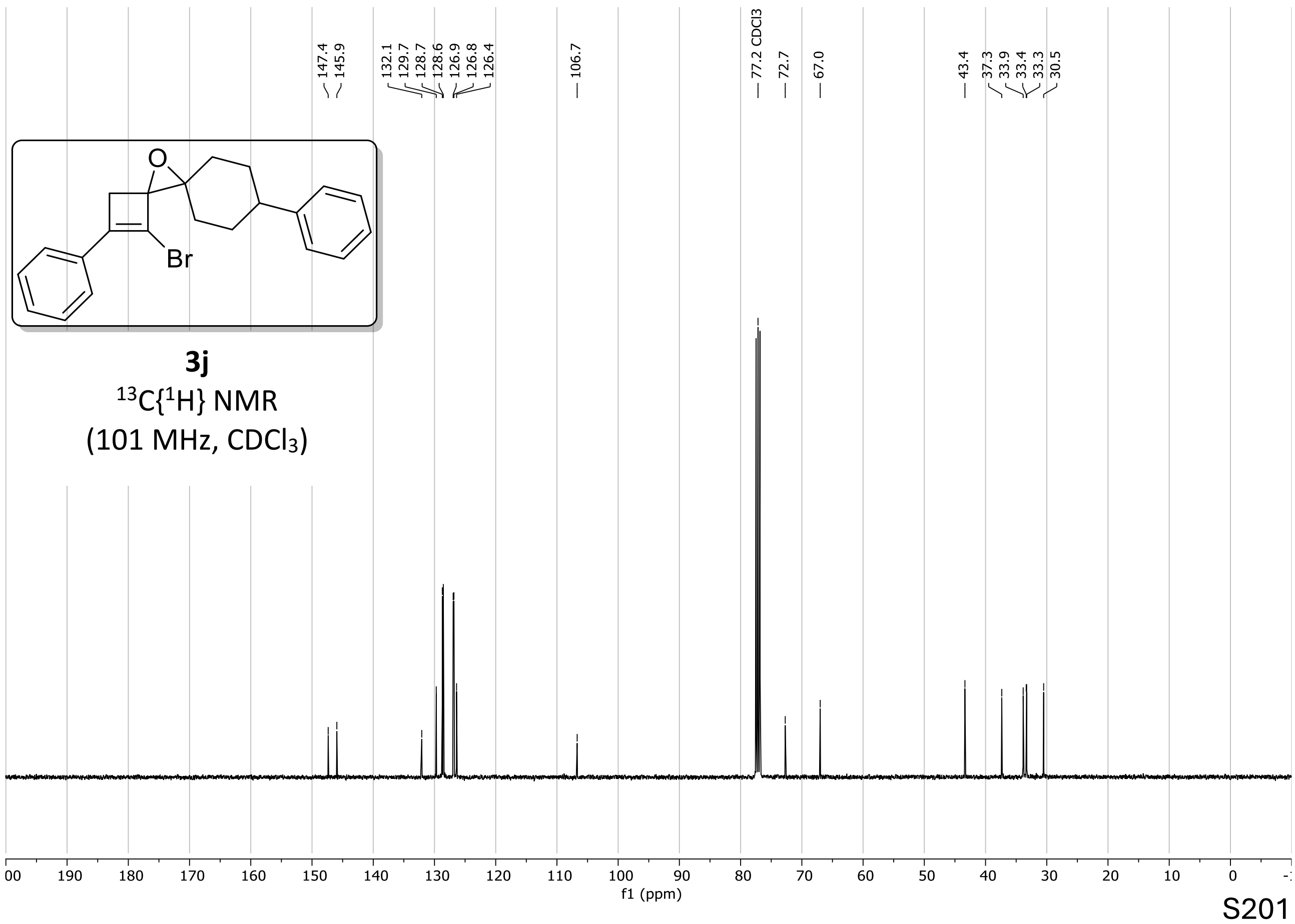

3j
$^{13}C\{^{1}H\}$ NMR
(101 MHz, $CDCl_3$)

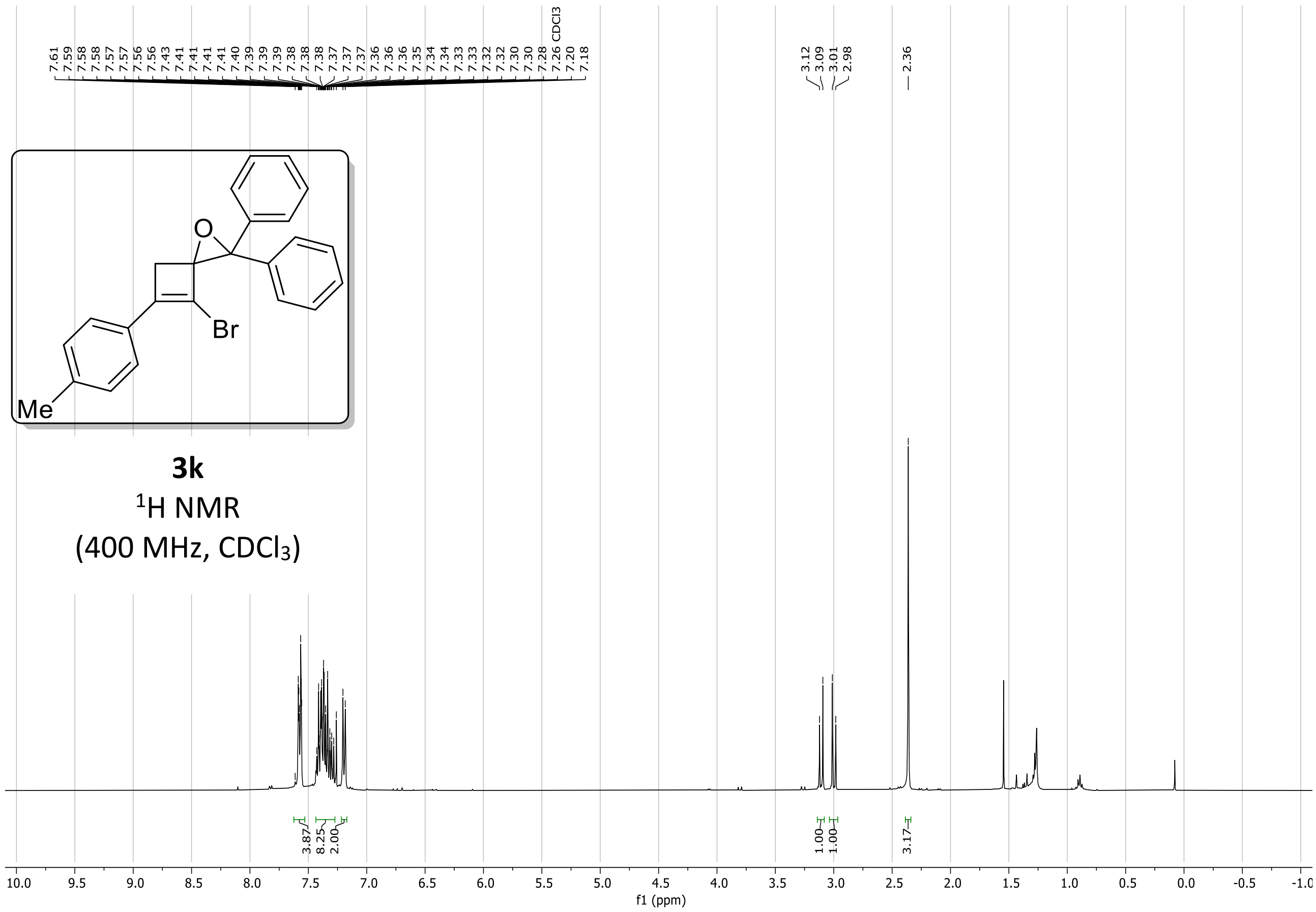

3k
^{1}H NMR
(400 MHz, $CDCl_3$)

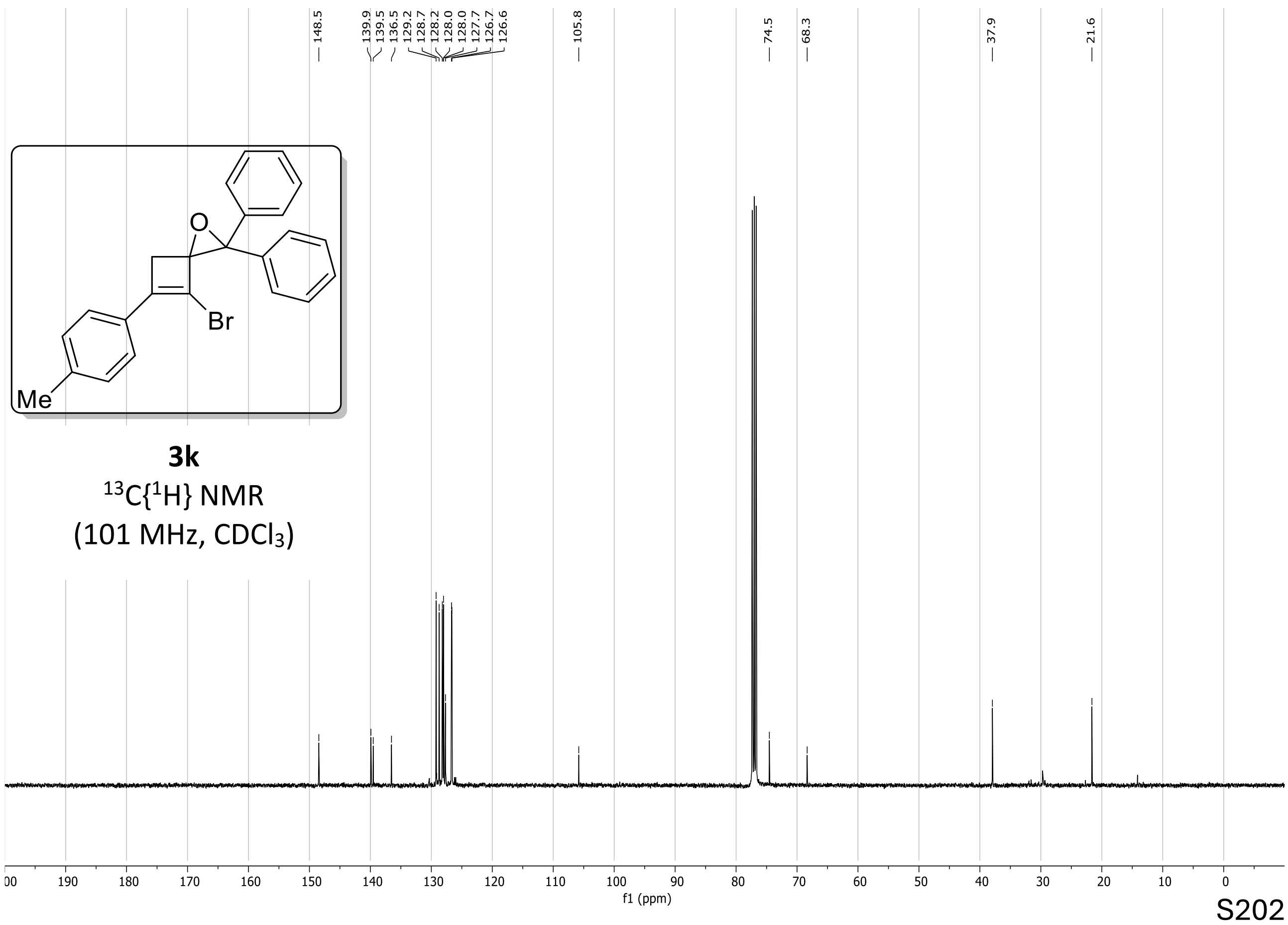

3k
$^{13}C\{^1H\}$ NMR
(101 MHz, $CDCl_3$)

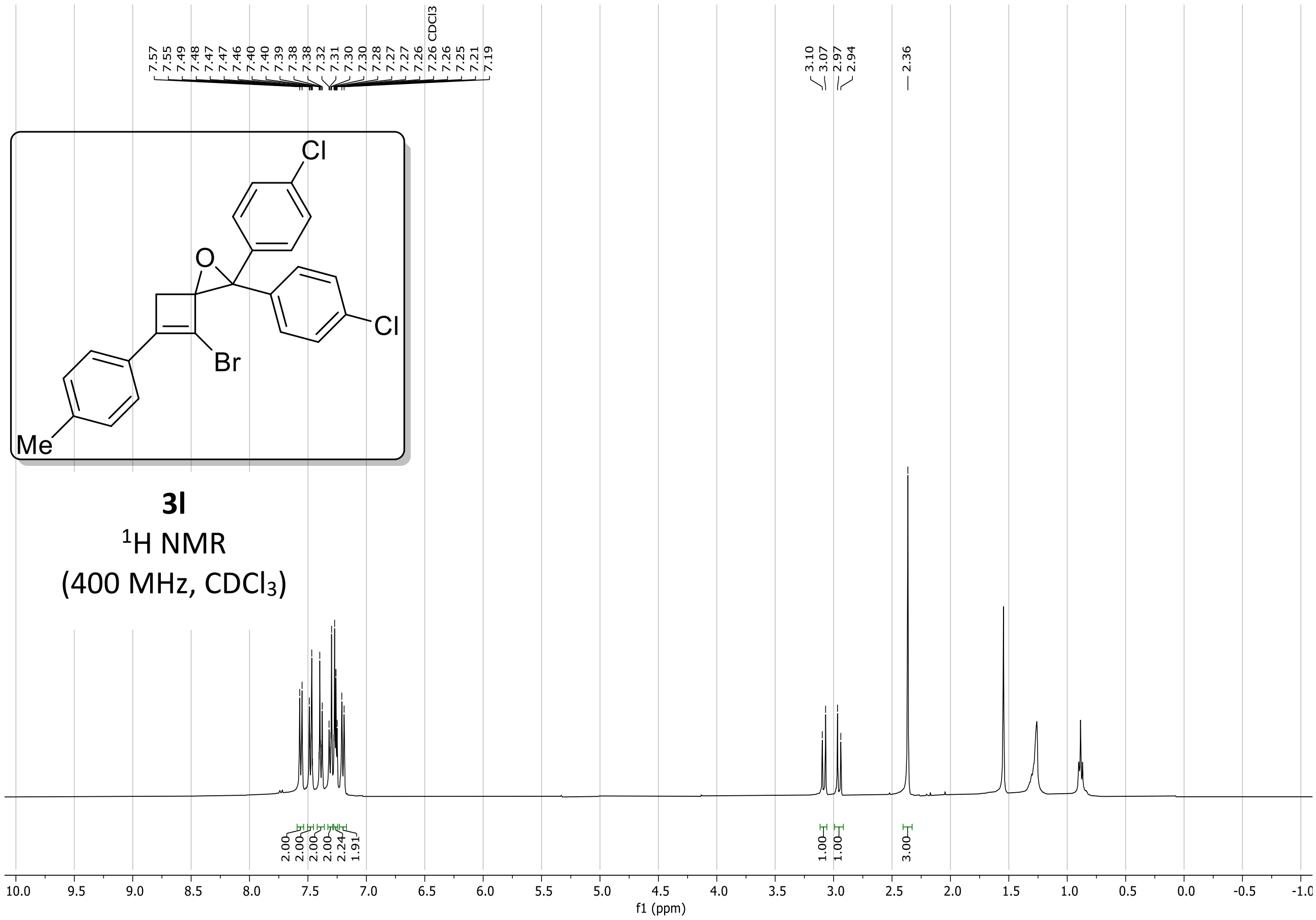

3l
^{1}H NMR
(400 MHz, $CDCl_3$)

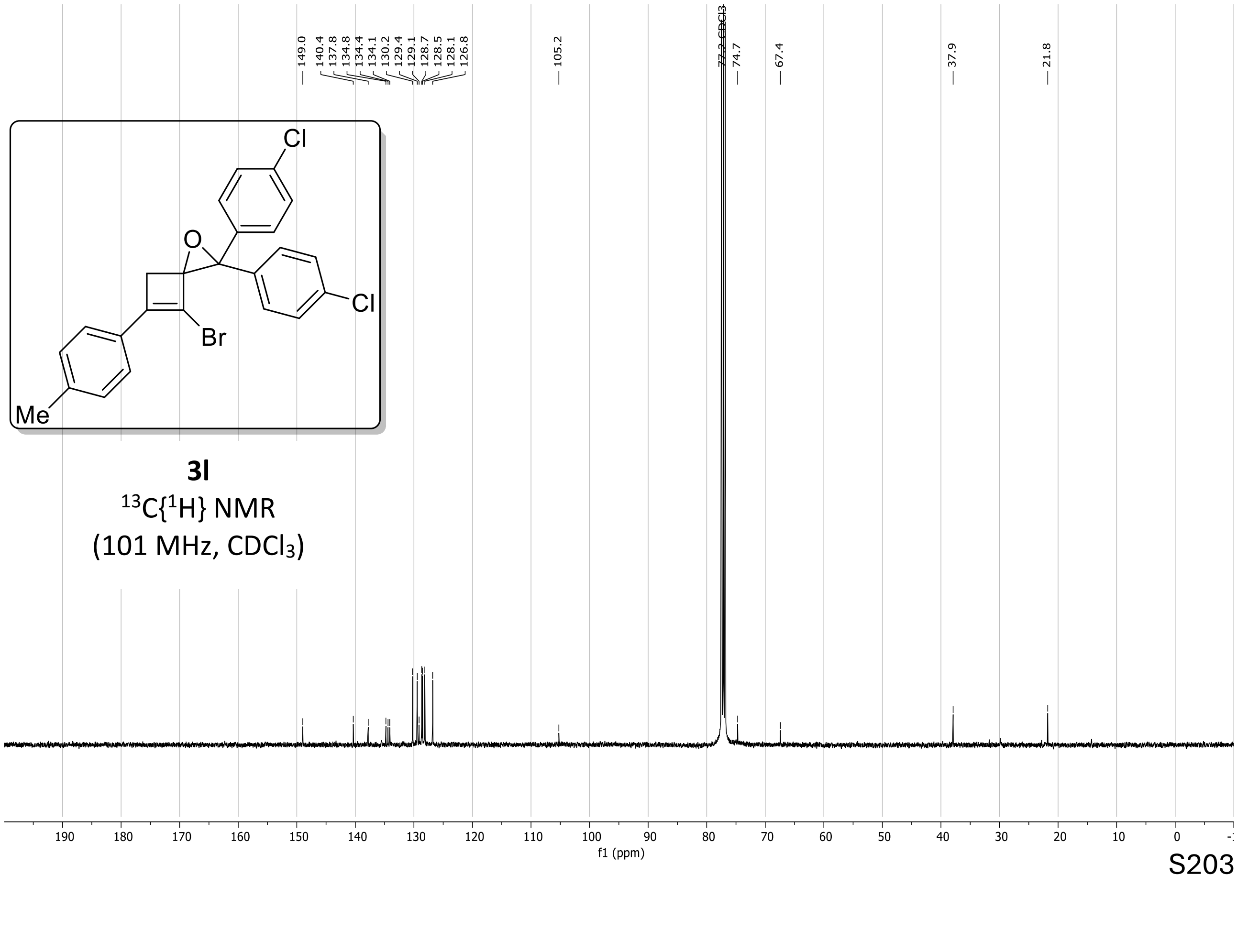

3l
$^{13}C\{^{1}H\}$ NMR
(101 MHz, $CDCl_3$)

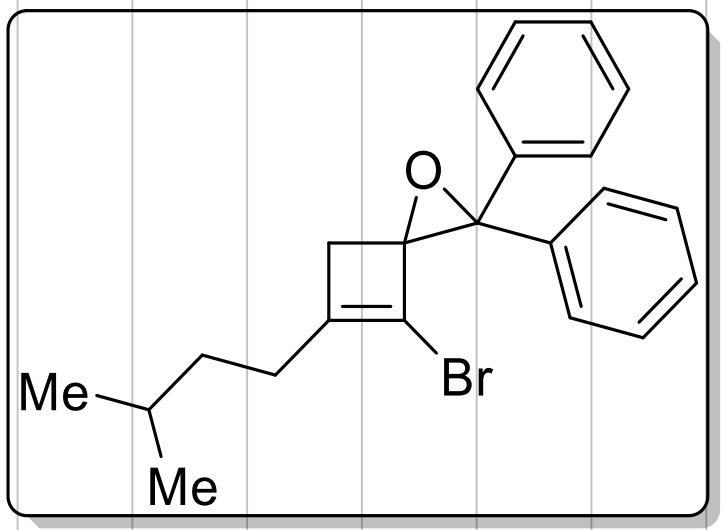

3m
^{1}H NMR
(400 MHz, $CDCl_3$)

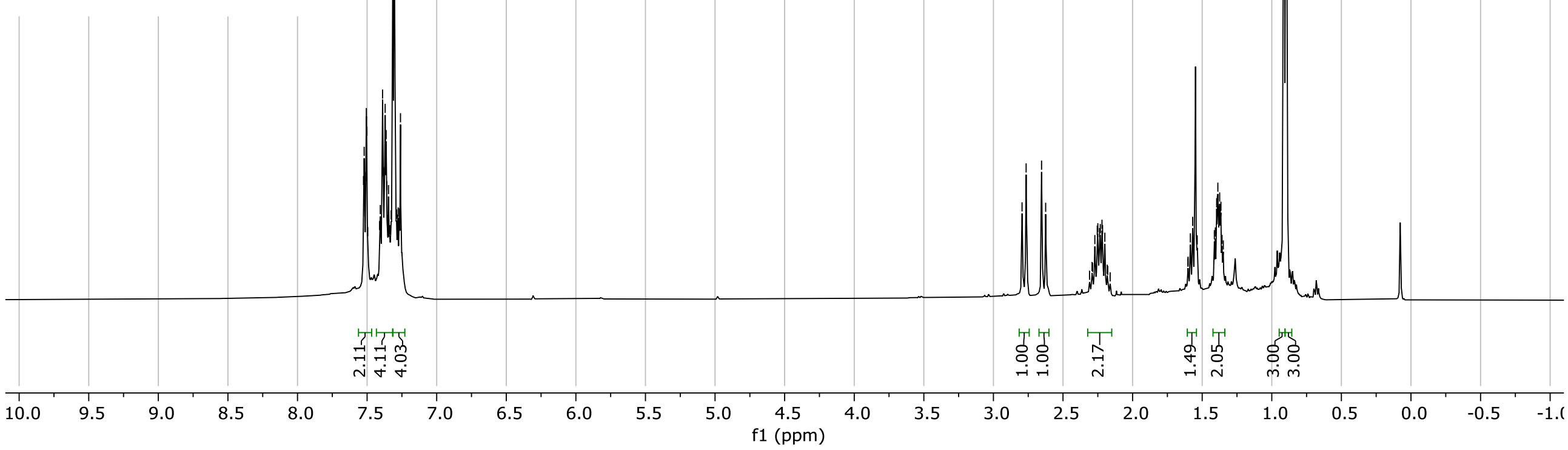

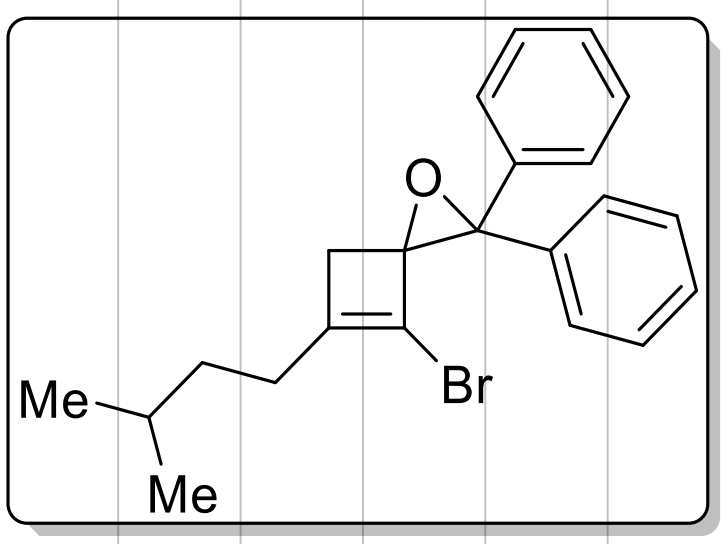

3m
$^{13}C\{^1H\}$ NMR
(101 MHz, $CDCl_3$)

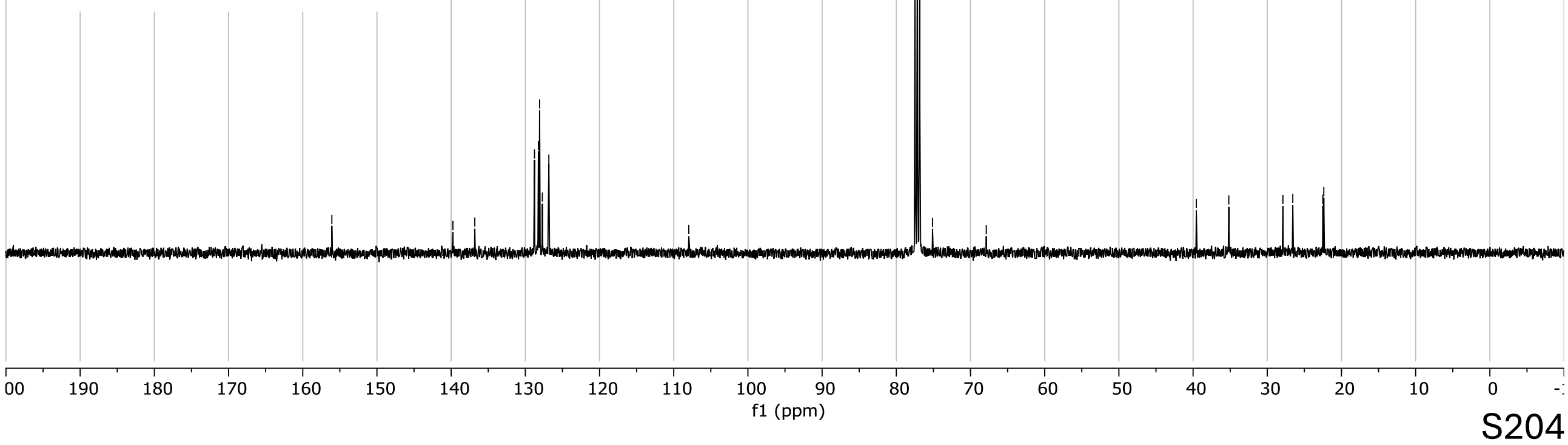

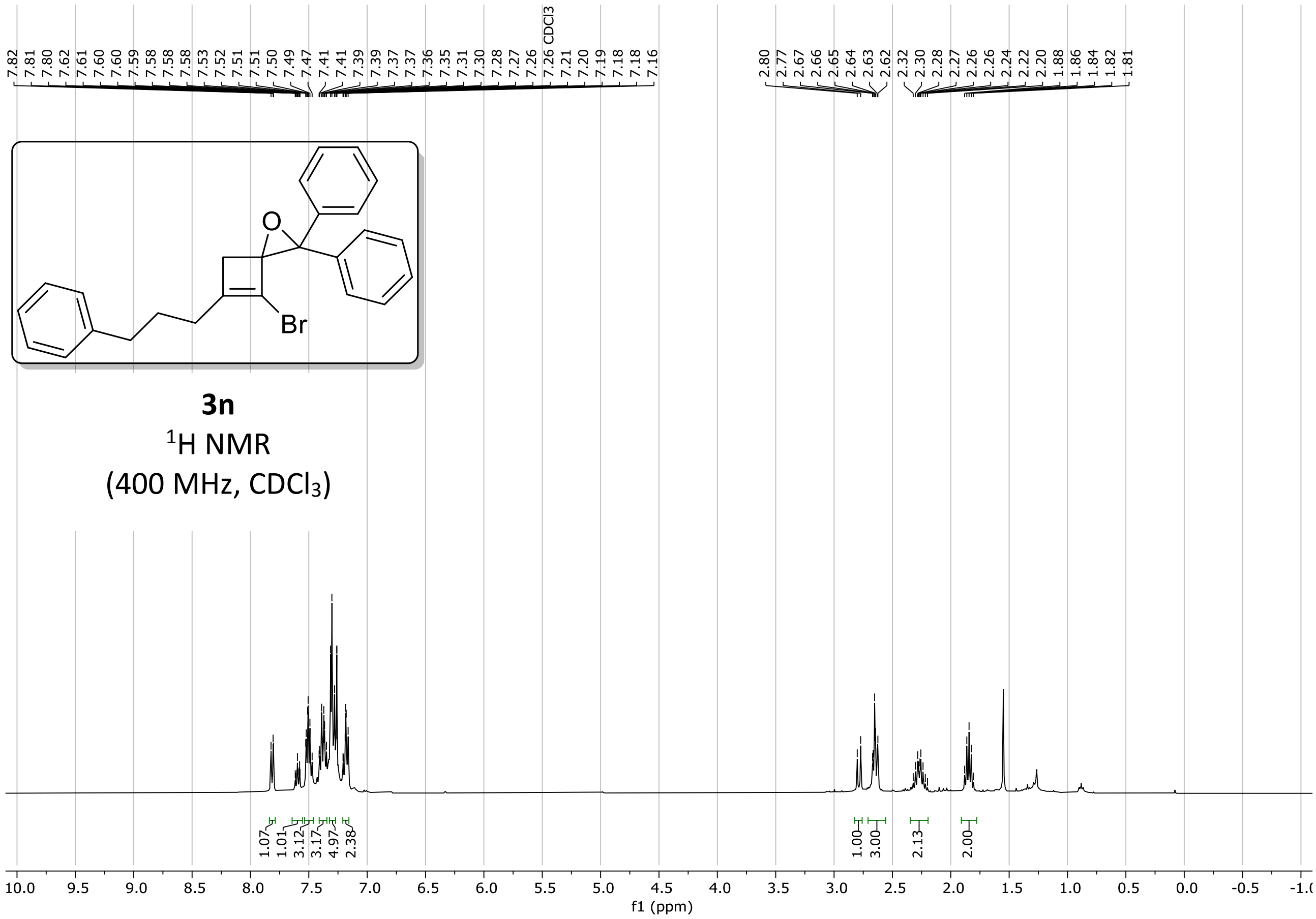
7.82 7.81 7.80 7.62 7.61 7.60 7.60 7.59 7.58 7.58 7.58 7.53 7.52 7.51 7.51 7.50 7.49 7.47 7.41 7.41 7.39 7.39 7.37 7.37 7.36 7.35 7.31 7.30 7.28 7.27 7.26 7.26 CDCl3 7.21 7.20 7.19 7.18 7.18 7.16
2.80 2.77 2.67 2.66 2.65 2.64 2.63 2.62 2.32 2.30 2.28 2.27 2.26 2.26 2.24 2.22 2.20 1.88 1.86 1.84 1.82 1.81
O
Br
3n
^{1}H NMR
(400 MHz, CDCl$_3$)
1.07 1.01 3.12 3.17 4.97 2.38
1.00 3.00 2.13 2.00
10.0 9.5 9.0 8.5 8.0 7.5 7.0 6.5 6.0 5.5 5.0 4.5 4.0 3.5 3.0 2.5 2.0 1.5 1.0 0.5 0.0 -0.5
f1 (ppm)

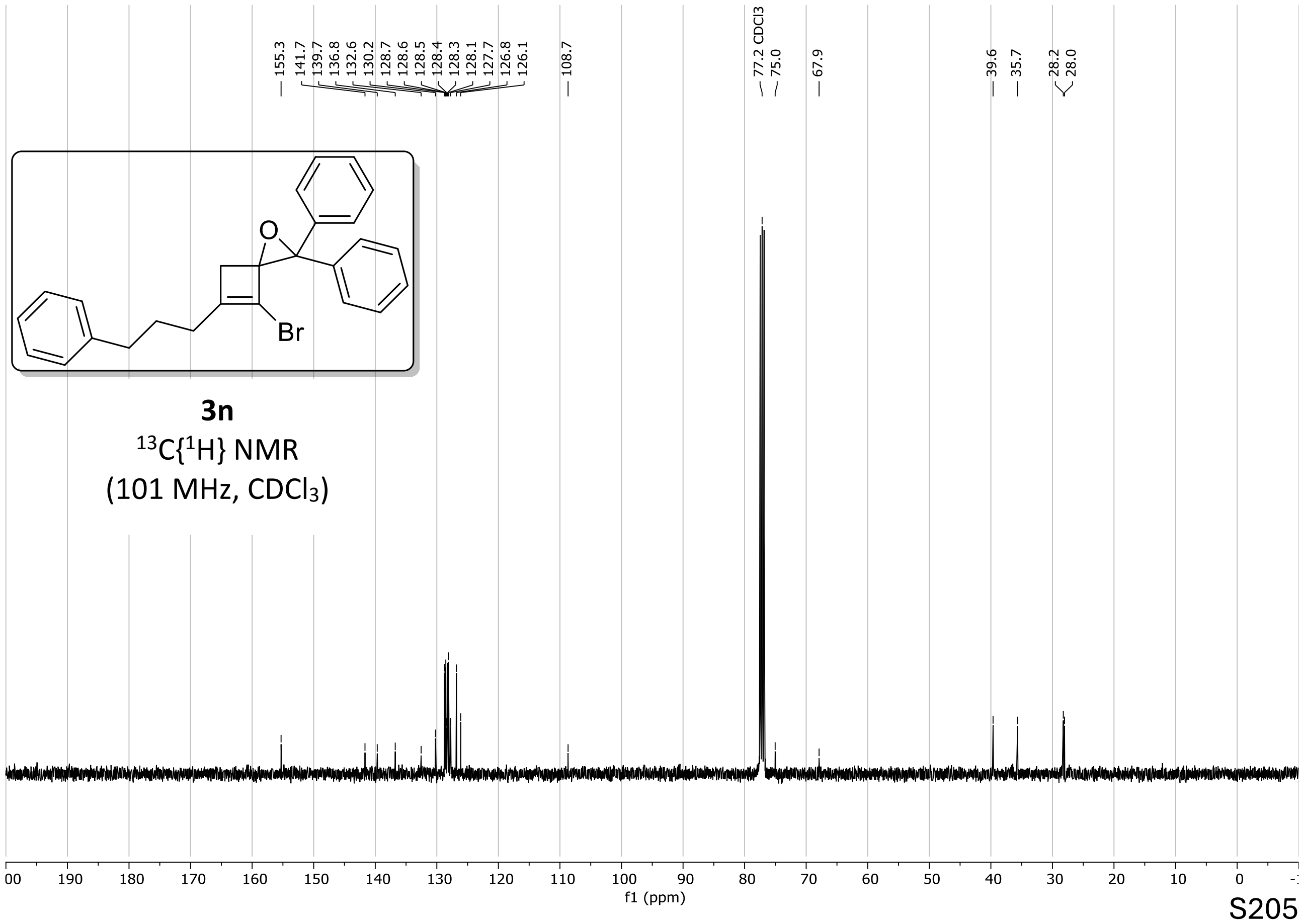
155.3 141.7 139.7 136.8 132.6 130.2 128.7 128.6 128.5 128.4 128.3 128.1 127.7 126.8 126.1 108.7
77.2 CDCl3 75.0 67.9
39.6 35.7 28.2 28.0
O
Br
3n
^{13}C{^{1}H} NMR
(101 MHz, CDCl$_3$)
190 180 170 160 150 140 130 120 110 100 90 80 70 60 50 40 30 20 10 0
f1 (ppm)

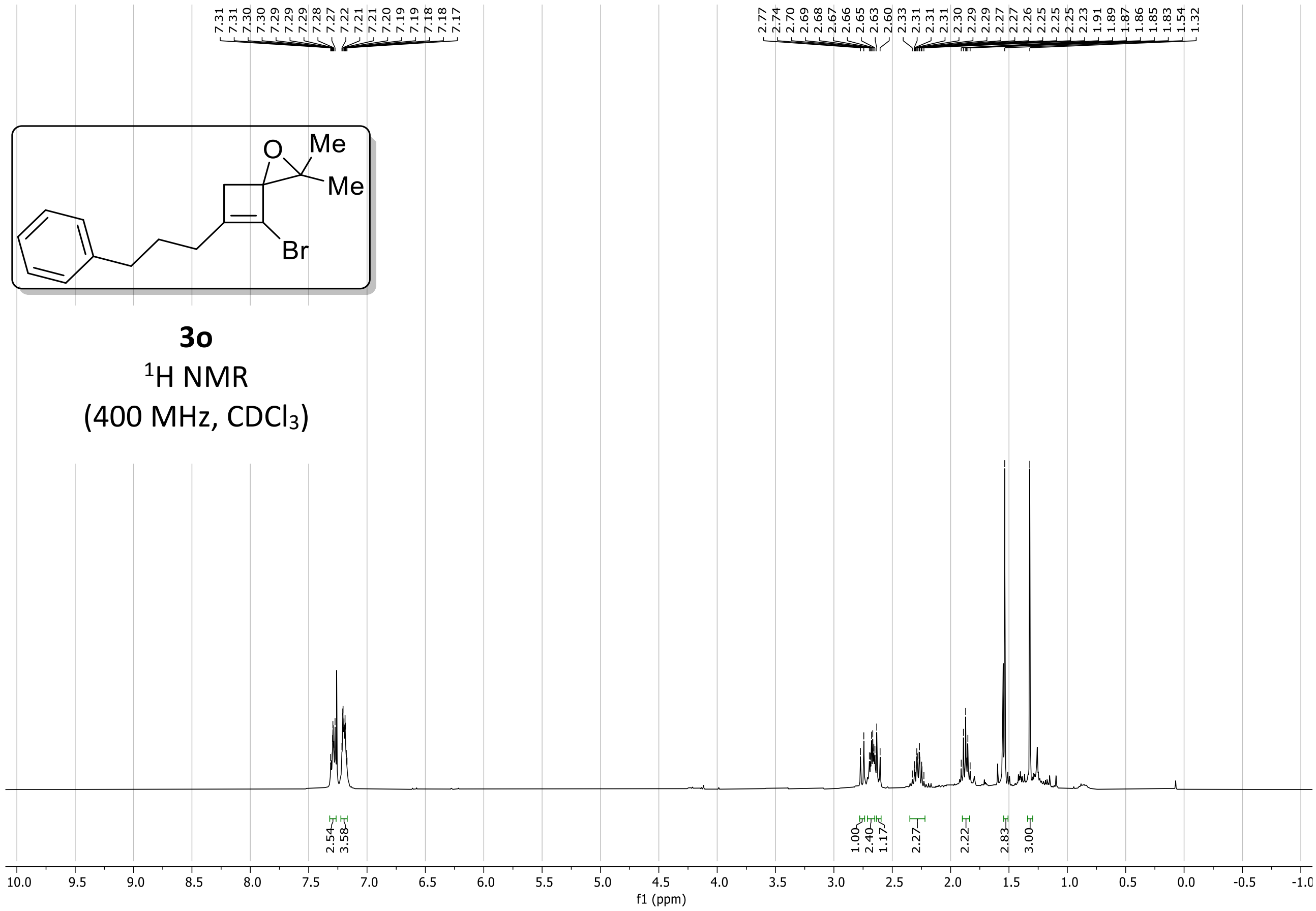

3o

^1H NMR

(400 MHz, $CDCl_3$)

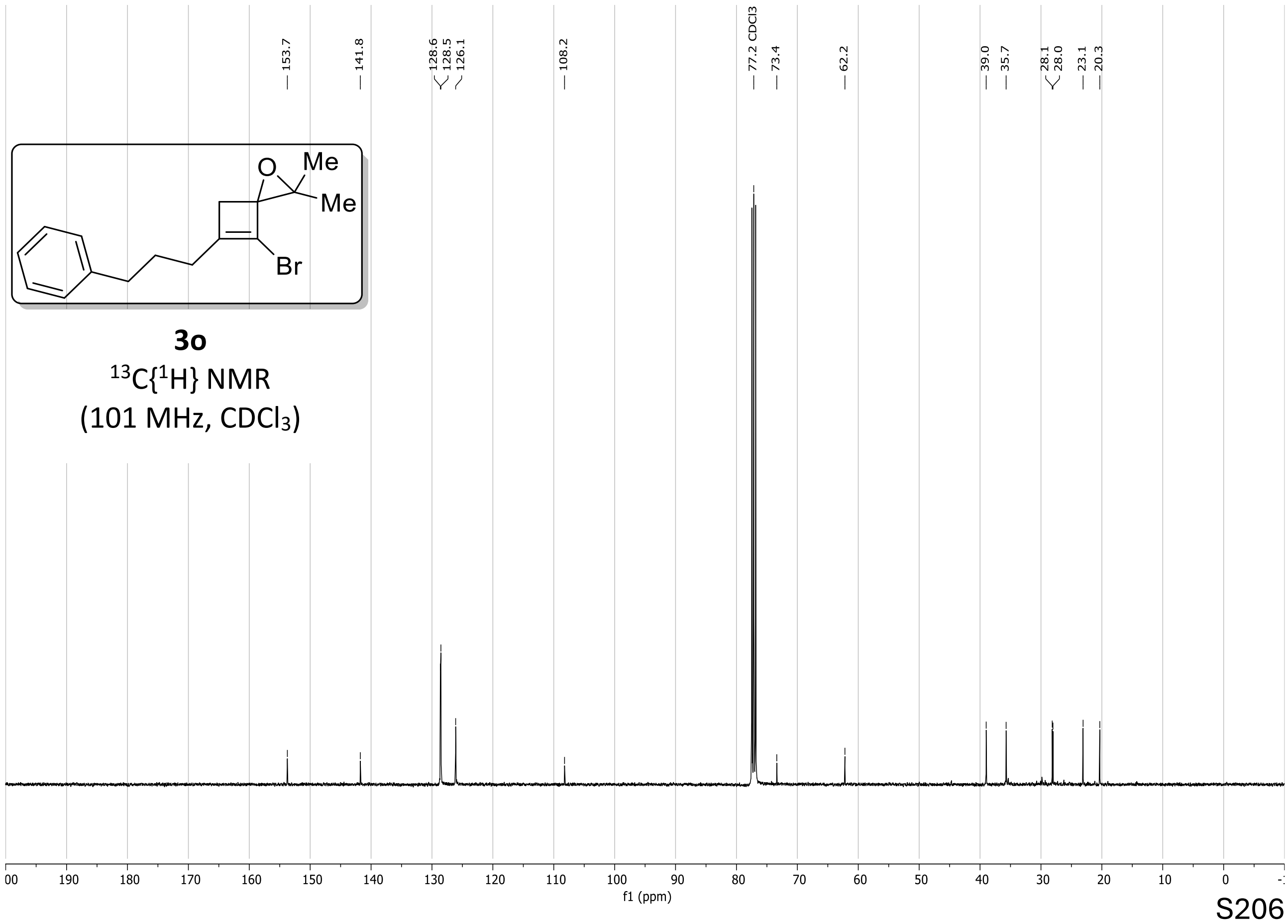

3o

$^{13}C\{^1H\}$ NMR

(101 MHz, $CDCl_3$)

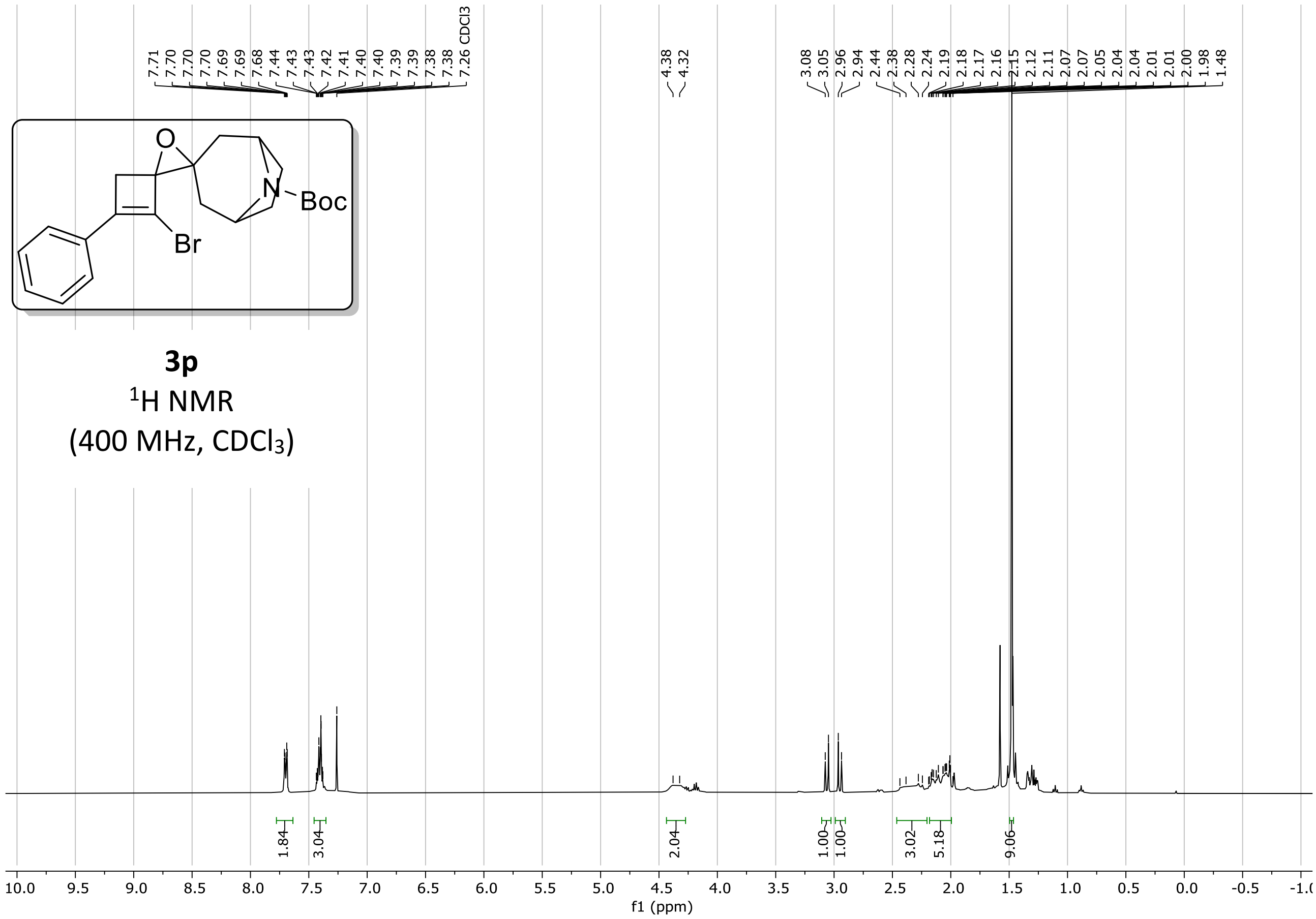

3p

^{1}H NMR

(400 MHz, $CDCl_3$)

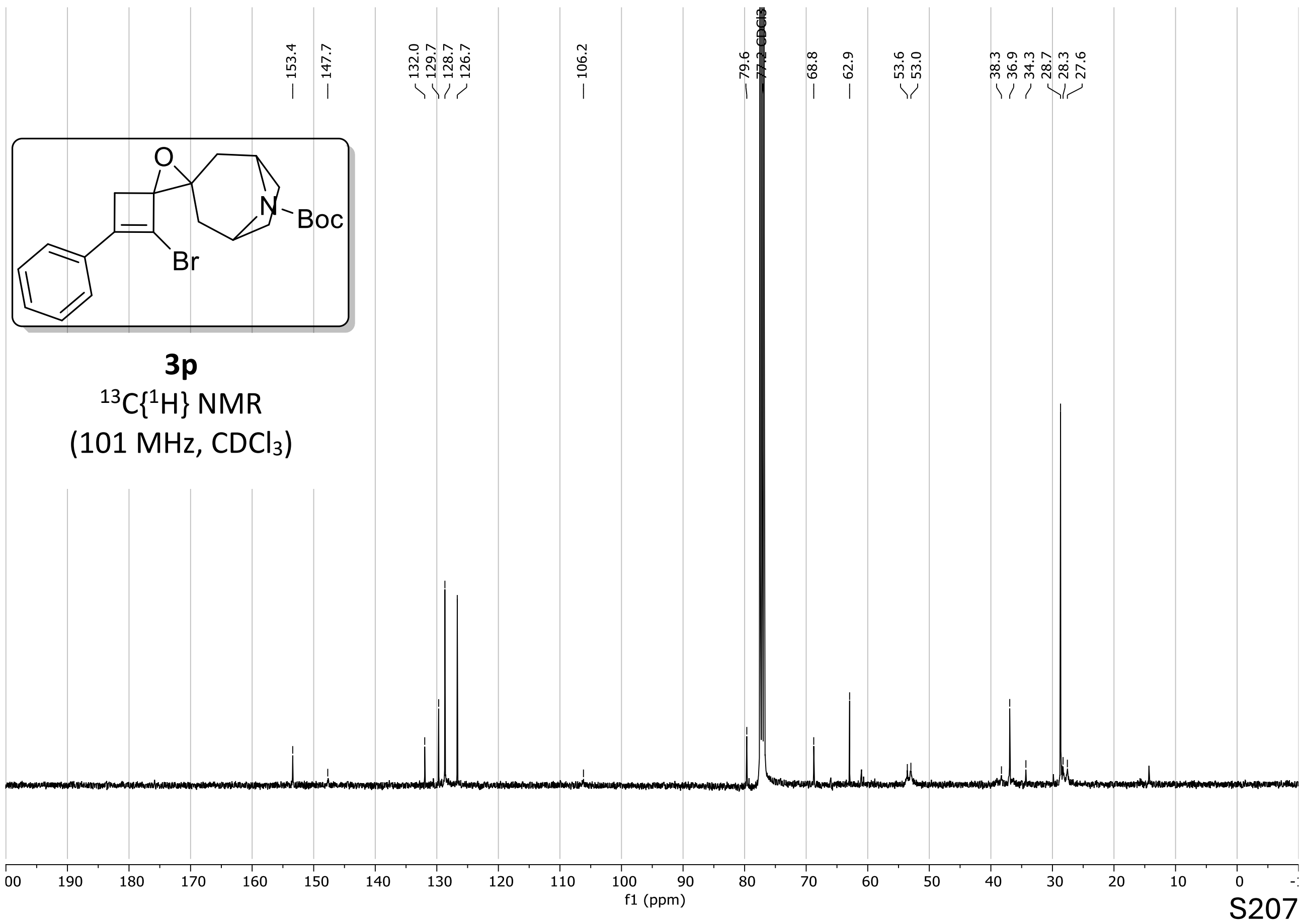

3p

$^{13}C\{^{1}H\}$ NMR

(101 MHz, $CDCl_3$)

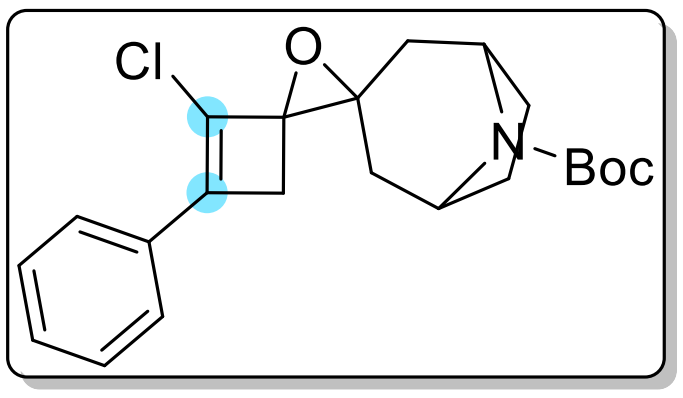

3p - HMBC ($CDCl_3$)
^1H NMR (400 MHz)
$^{13}C\{^1H\}$ NMR (101 MHz)

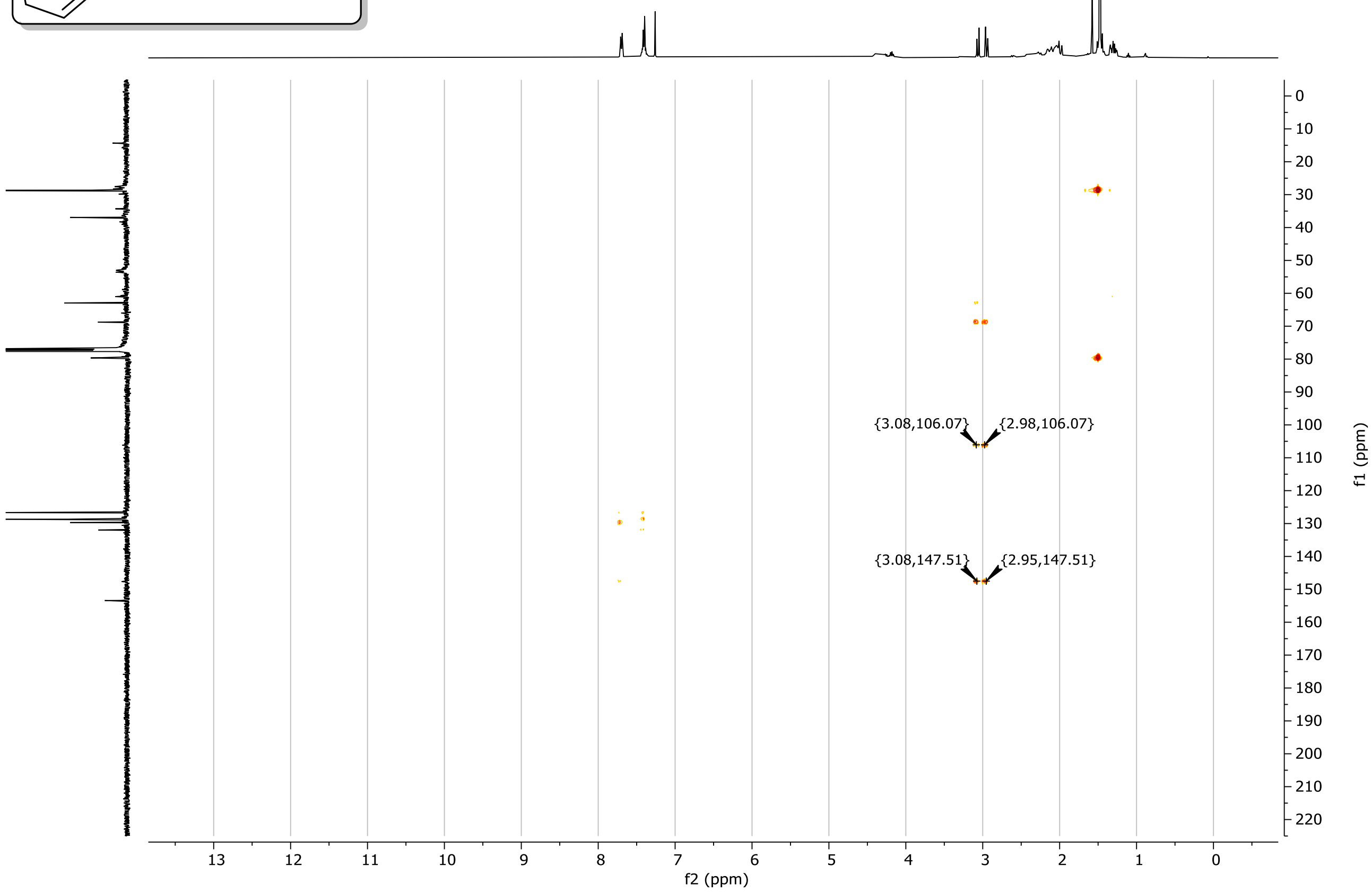

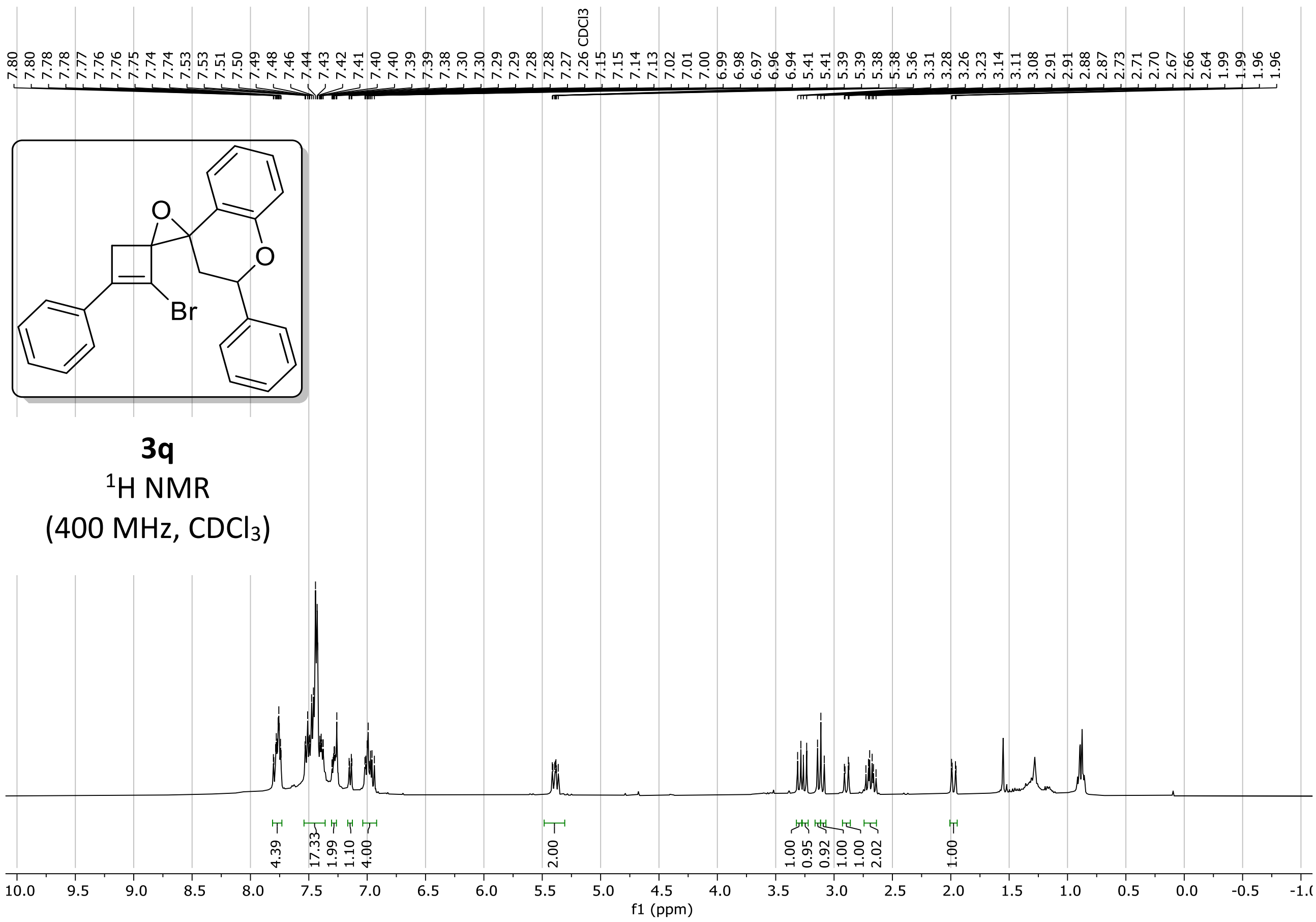

3q

^{1}H NMR

(400 MHz, $CDCl_3$)

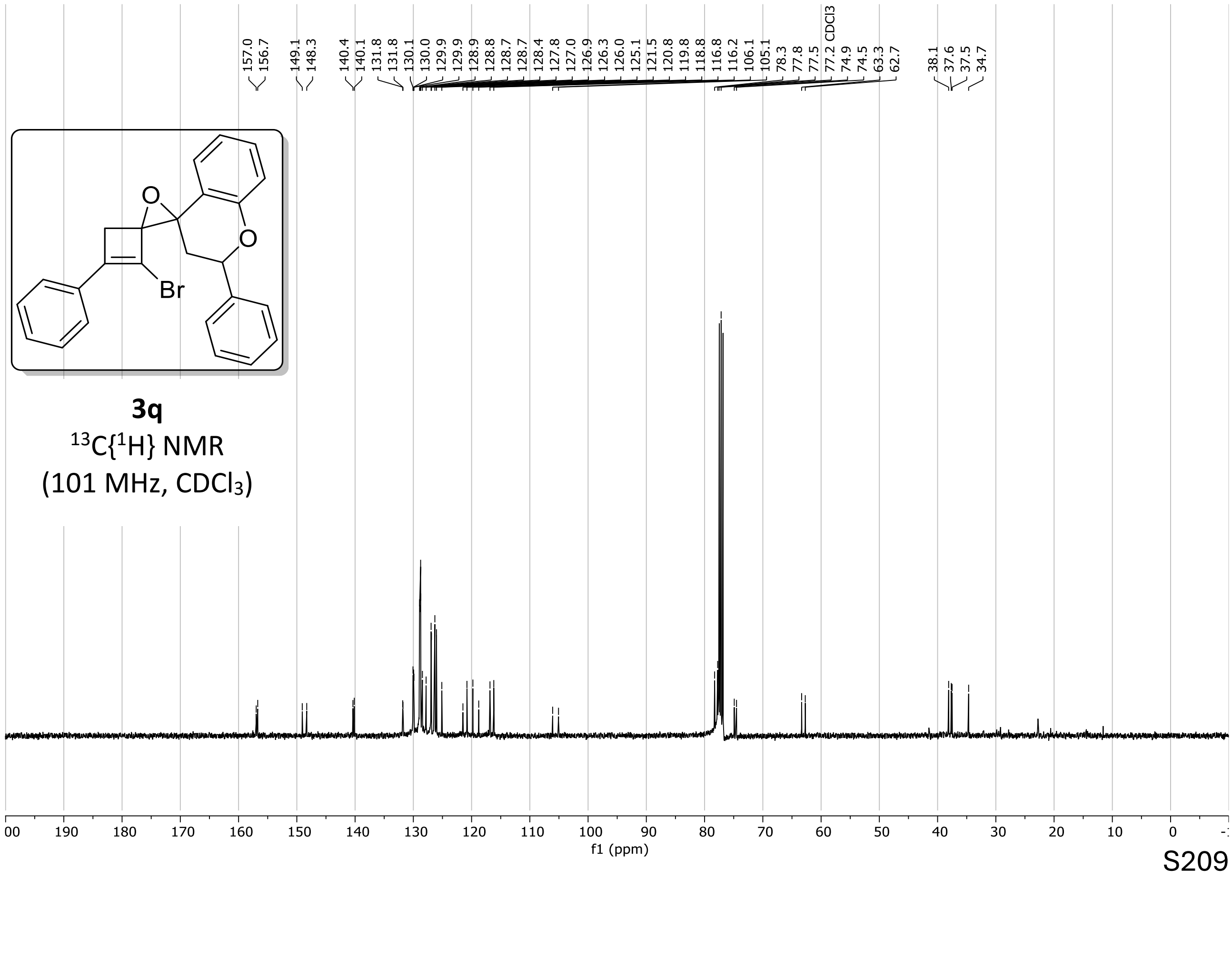

3q

^{13}C{^{1}H} NMR

(101 MHz, $CDCl_3$)

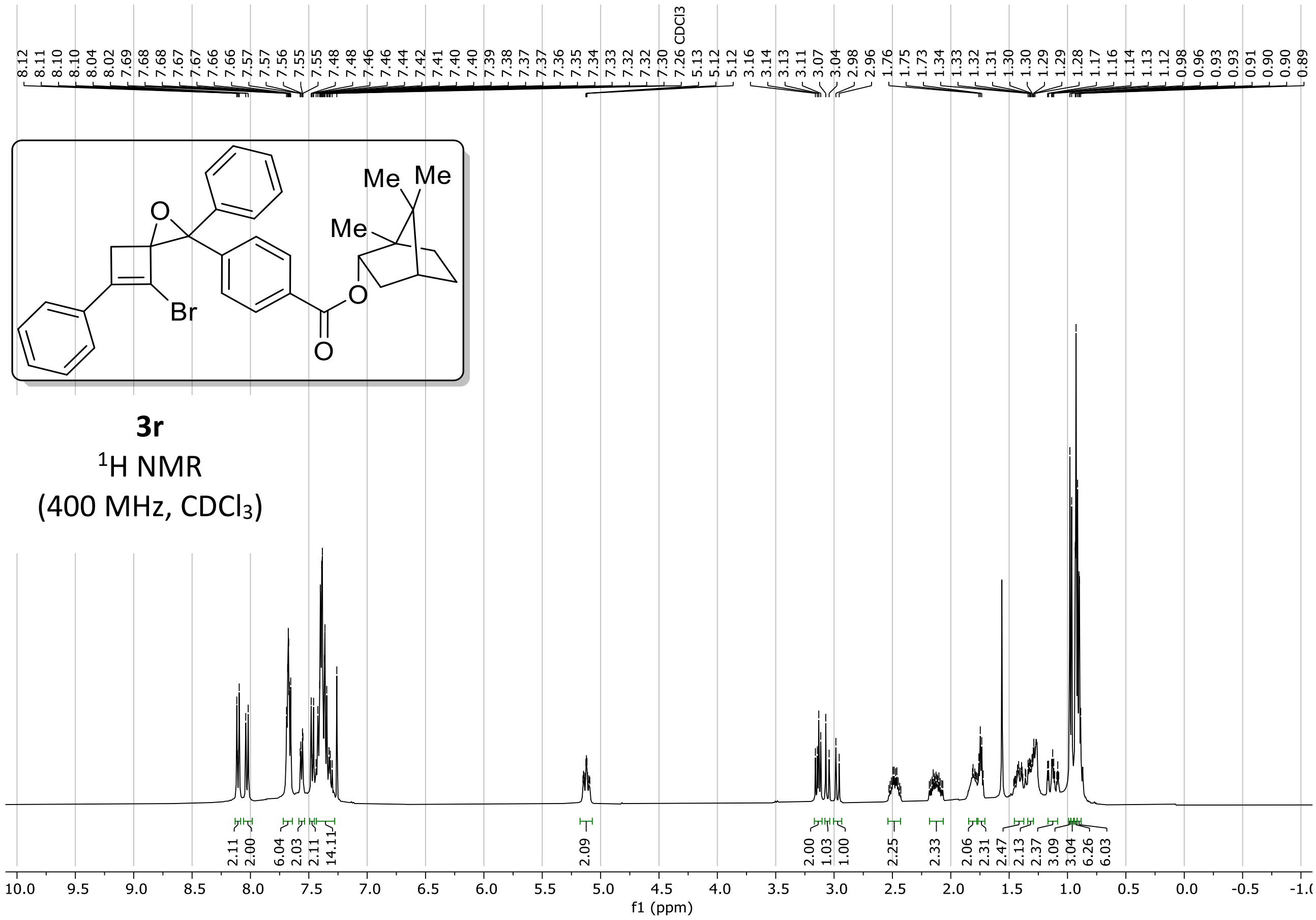
8.12 8.11 8.10 8.10 8.04 8.02 7.69 7.68 7.68 7.67 7.67 7.66 7.66 7.57 7.57 7.56 7.55 7.55 7.48 7.48 7.46 7.46 7.44 7.42 7.41 7.40 7.40 7.39 7.38 7.37 7.37 7.36 7.35 7.34 7.33 7.32 7.32 7.30 7.26 CDCl3 5.13 5.12 5.12 3.16 3.14 3.13 3.11 3.07 3.04 2.98 2.96 1.76 1.75 1.73 1.34 1.33 1.32 1.31 1.30 1.30 1.29 1.29 1.28 1.17 1.16 1.14 1.13 1.12 0.98 0.96 0.93 0.93 0.91 0.90 0.89
Me
Me
Me
O
O
O
Br
3r
1H NMR
(400 MHz, CDCl3)
2.11 2.00 6.04 2.03 2.11 14.11 2.09 2.00 1.03 1.00 2.25 2.33 2.06 2.31 2.47 2.13 2.37 3.09 3.04 6.26 6.03
10.0 9.5 9.0 8.5 8.0 7.5 7.0 6.5 6.0 5.5 5.0 4.5 4.0 3.5 3.0 2.5 2.0 1.5 1.0 0.5 0.0 -0.5
f1 (ppm)

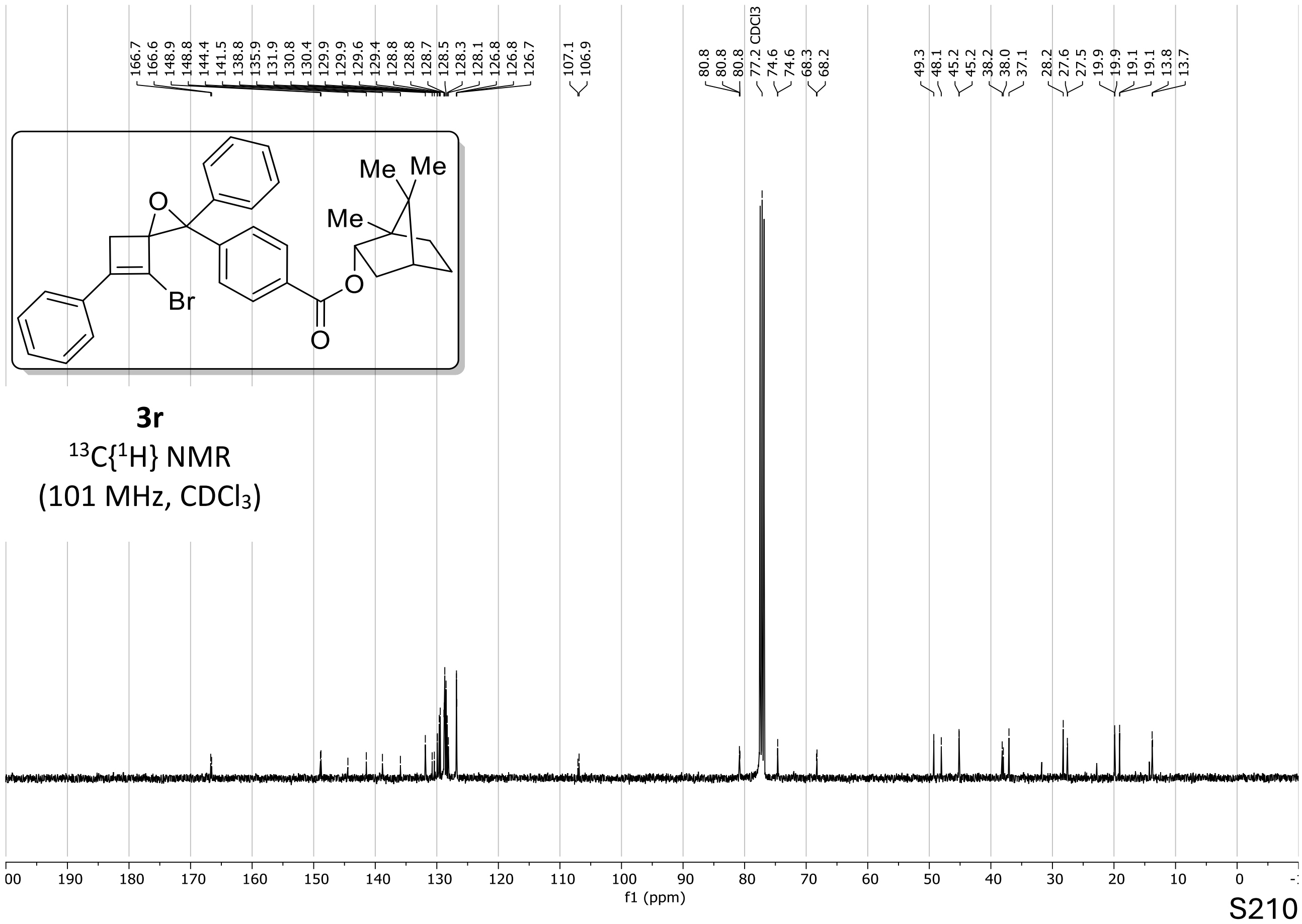
166.7 166.6 148.9 148.8 144.4 141.5 138.8 135.9 131.9 130.8 130.4 129.9 129.9 129.6 129.4 128.8 128.8 128.7 128.5 128.3 128.1 126.8 126.8 126.7 107.1 106.9 80.8 80.8 80.8 77.2 CDCl3 74.6 74.6 68.3 68.2 49.3 48.1 45.2 45.2 38.2 38.0 37.1 28.2 27.6 27.5 19.9 19.1 19.1 13.8 13.7
Me
Me
Me
O
O
O
Br
3r
13C{1H} NMR
(101 MHz, CDCl3)
190 180 170 160 150 140 130 120 110 100 90 80 70 60 50 40 30 20 10 0
f1 (ppm)

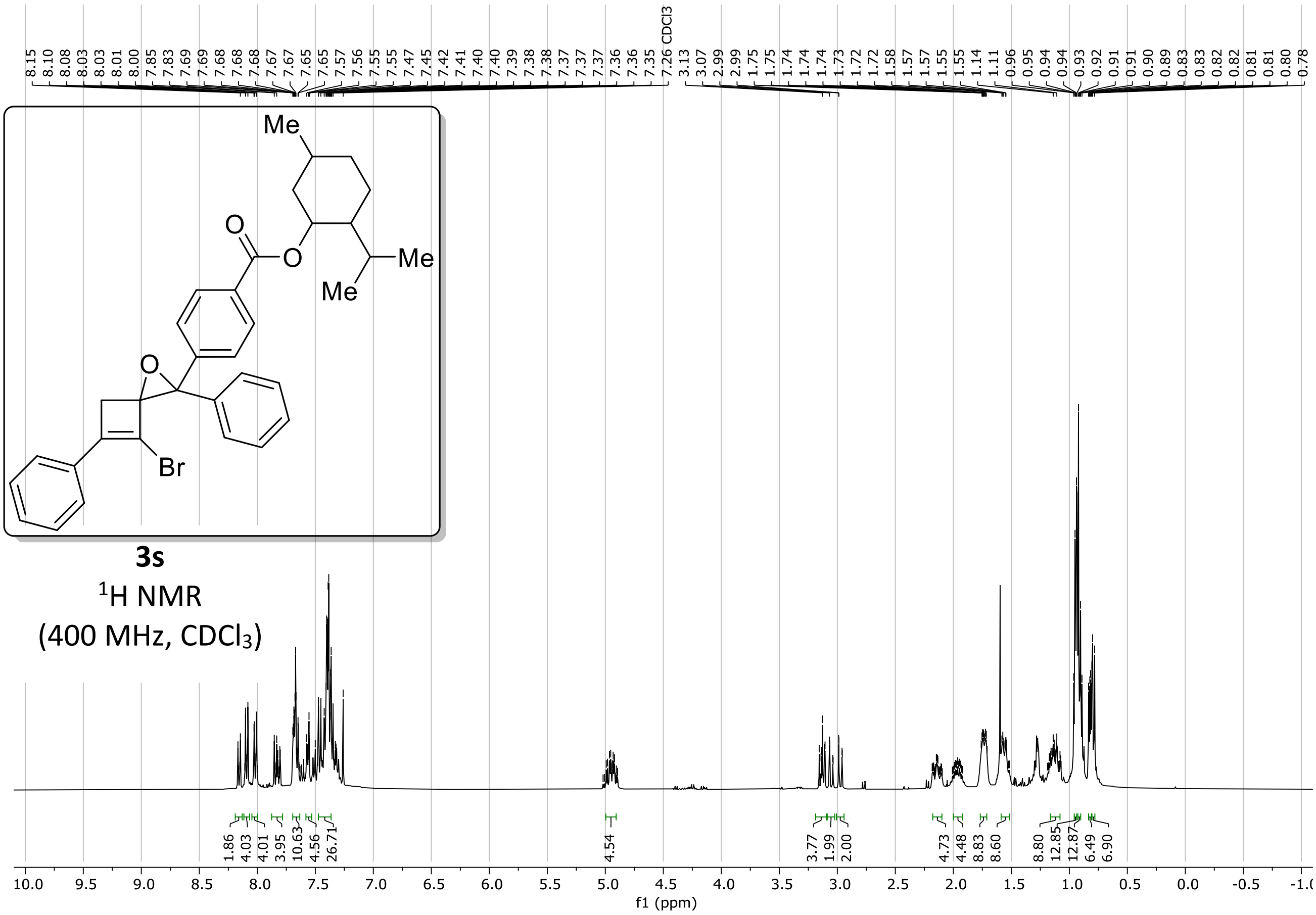

3s

^{1}H NMR

(400 MHz, $CDCl_3$)

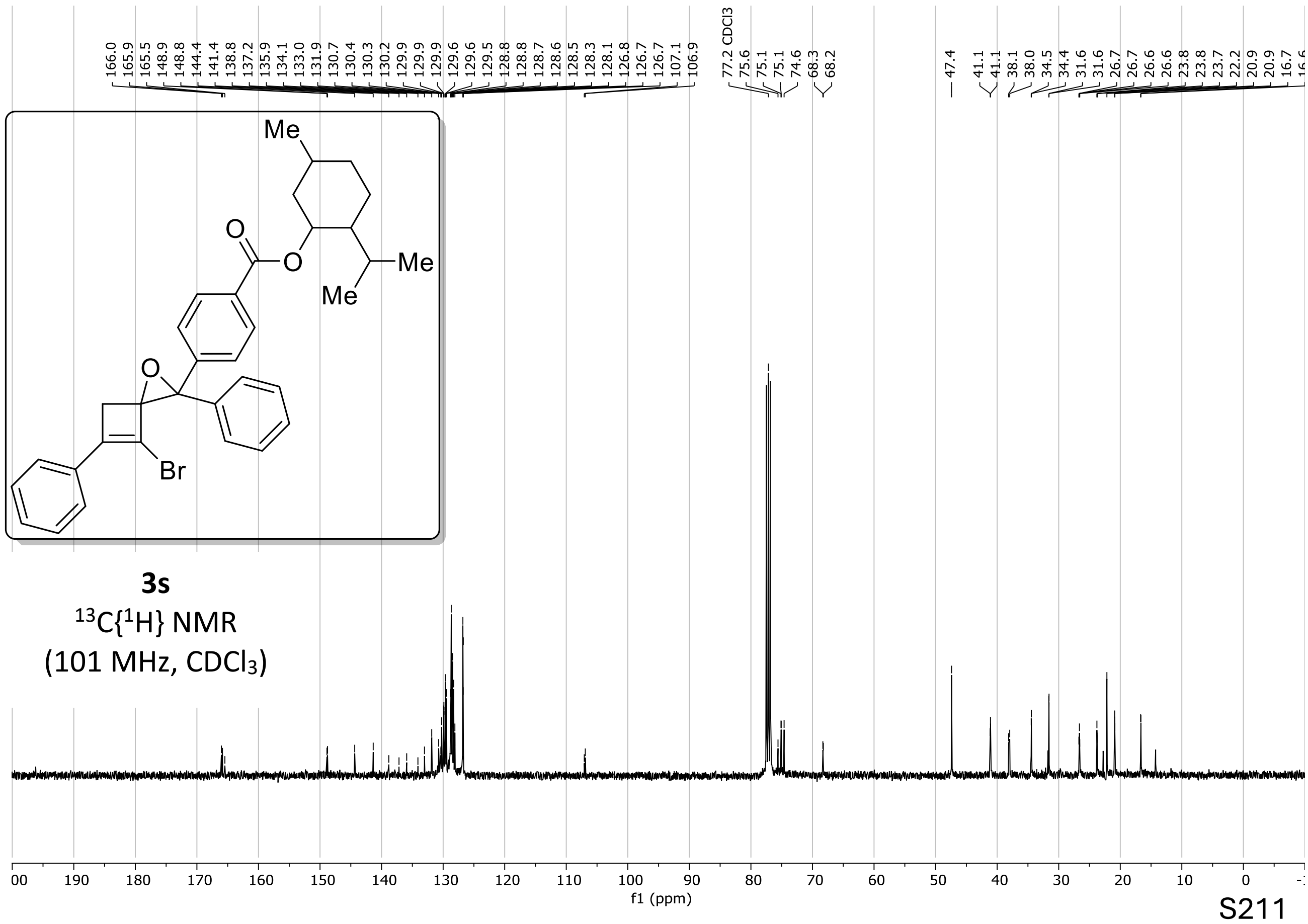

3s

$^{13}C\{^{1}H\}$ NMR

(101 MHz, $CDCl_3$)

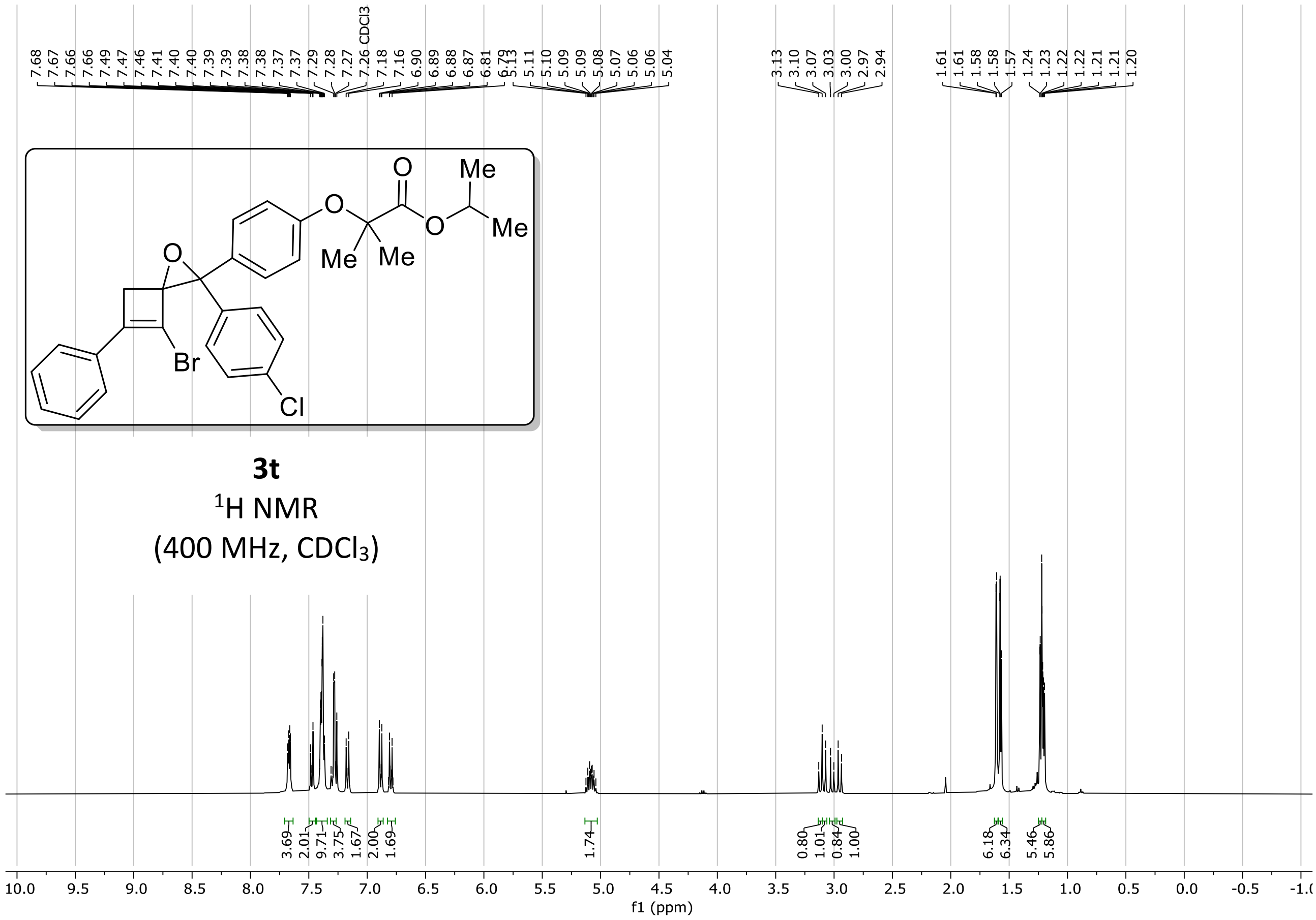

3t
^{1}H NMR
(400 MHz, $CDCl_3$)

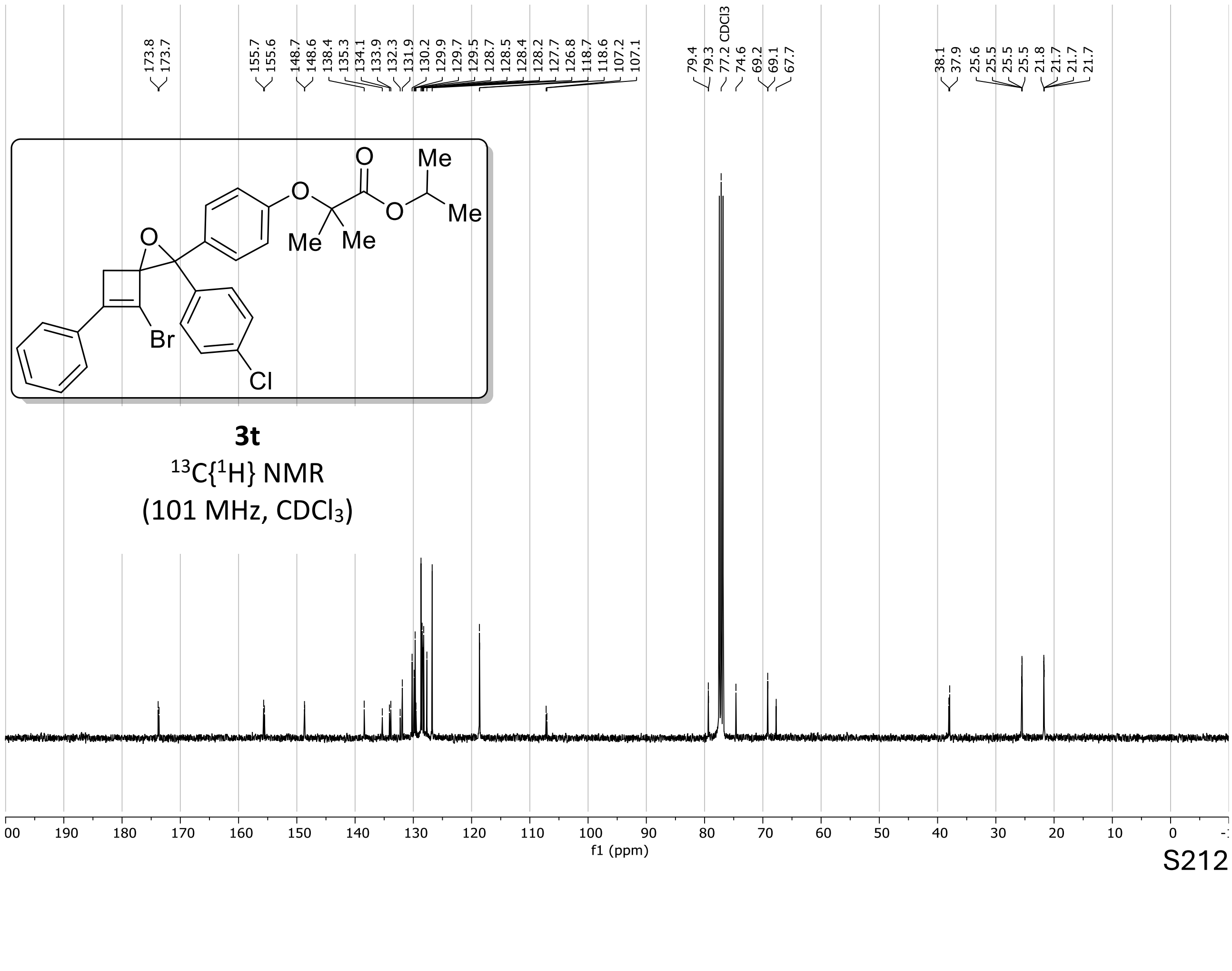

3t
$^{13}C\{^{1}H\}$ NMR
(101 MHz, $CDCl_3$)

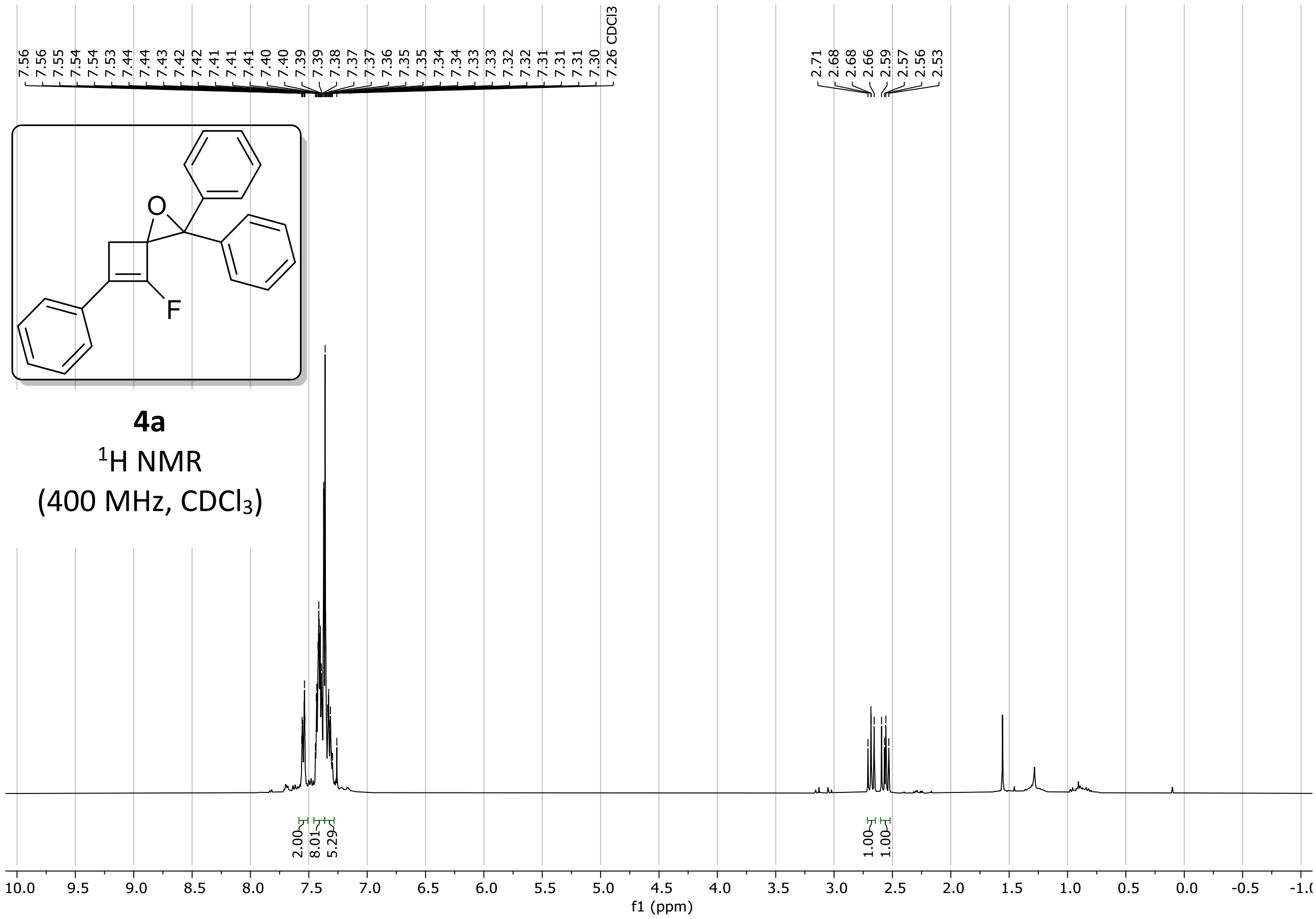

4a

^{1}H NMR

(400 MHz, $CDCl_3$)

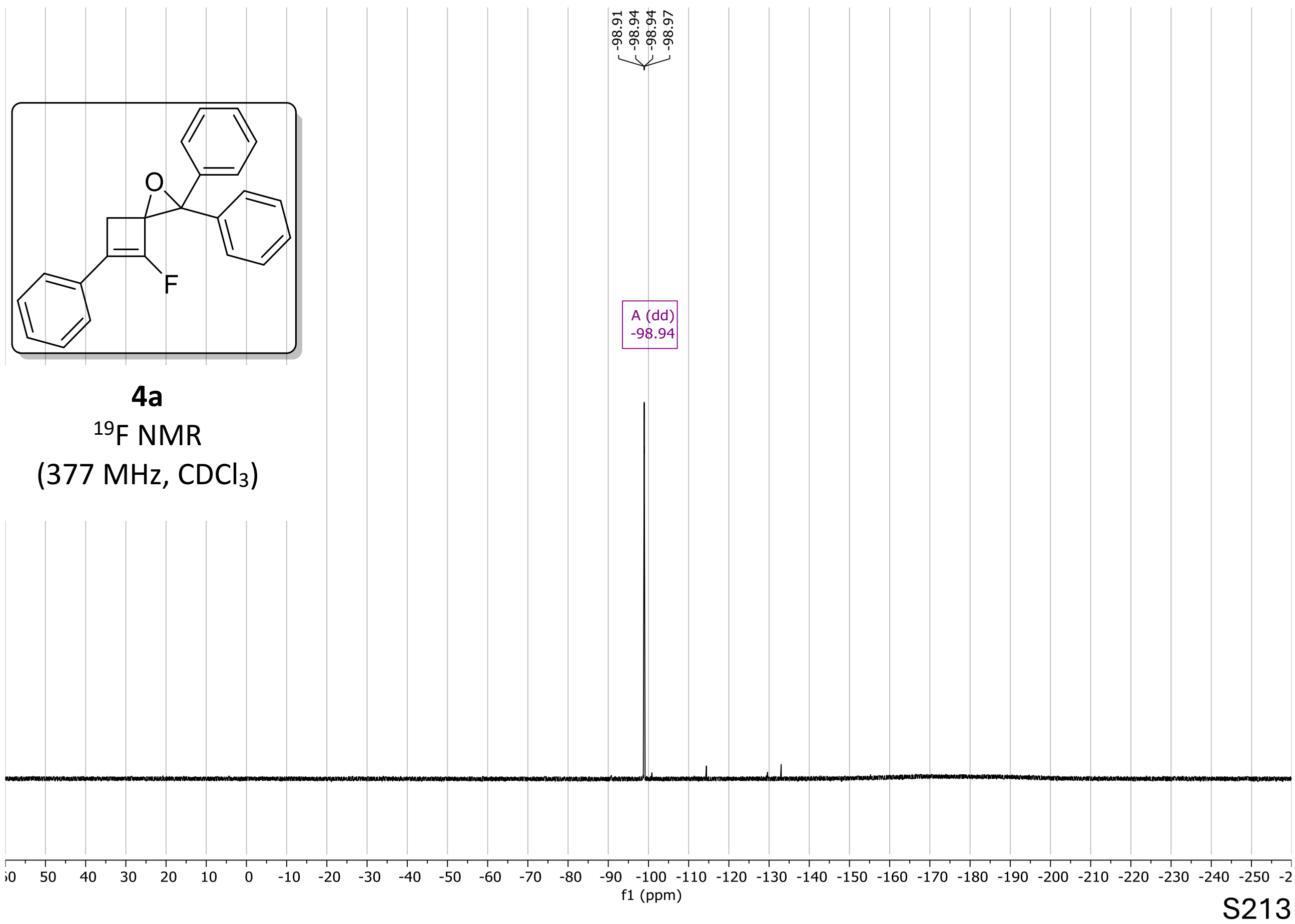

4a

^{19}F NMR

(377 MHz, $CDCl_3$)

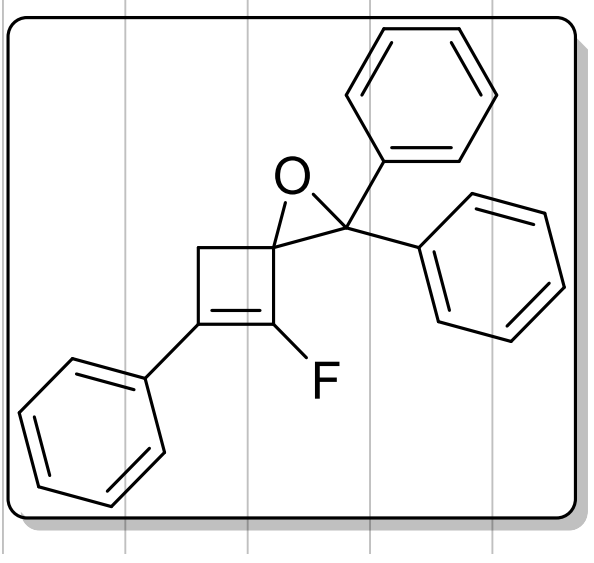

4a
$^{13}C\{^{1}H\}$ NMR
(101 MHz, $CDCl_3$)

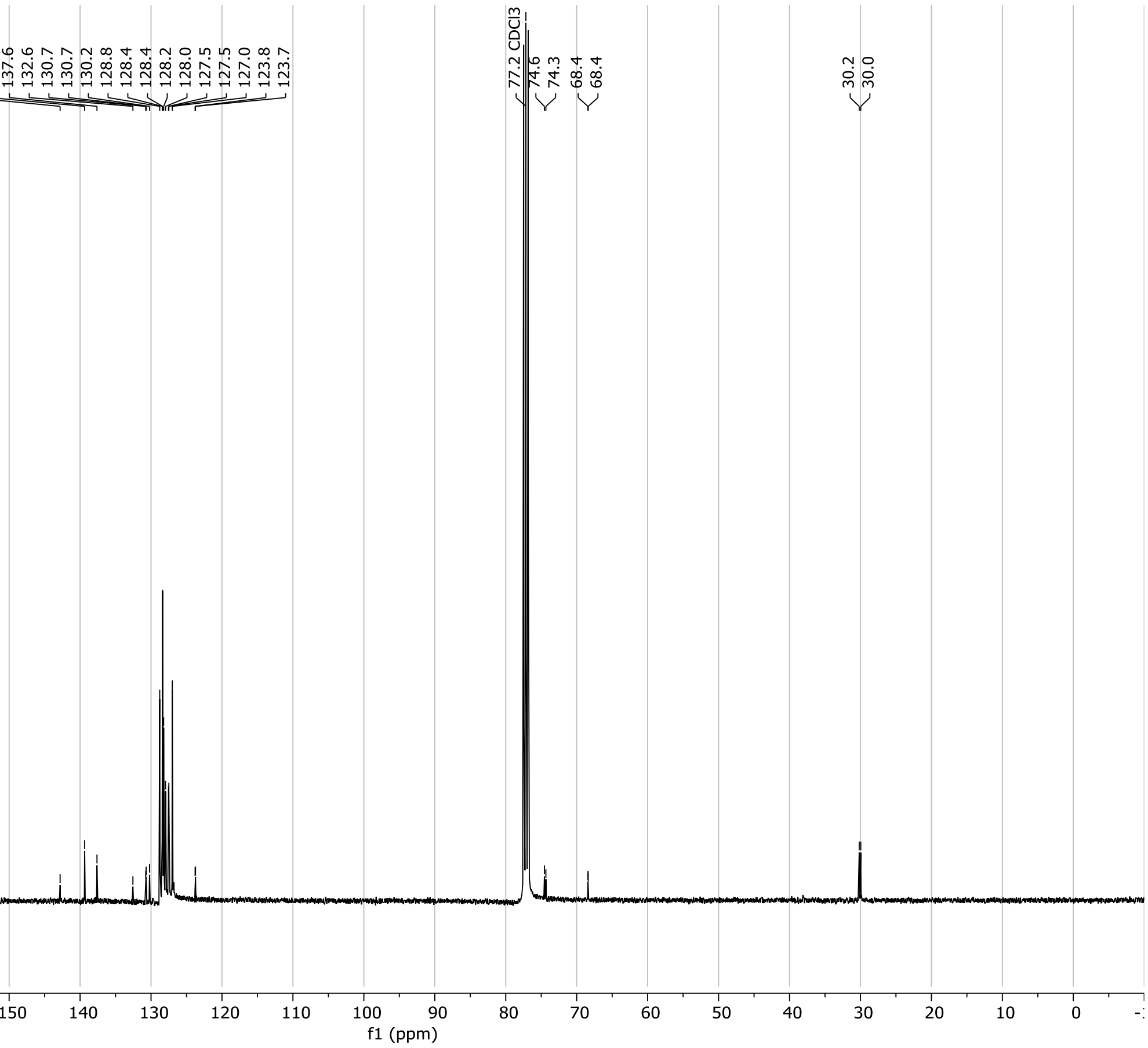

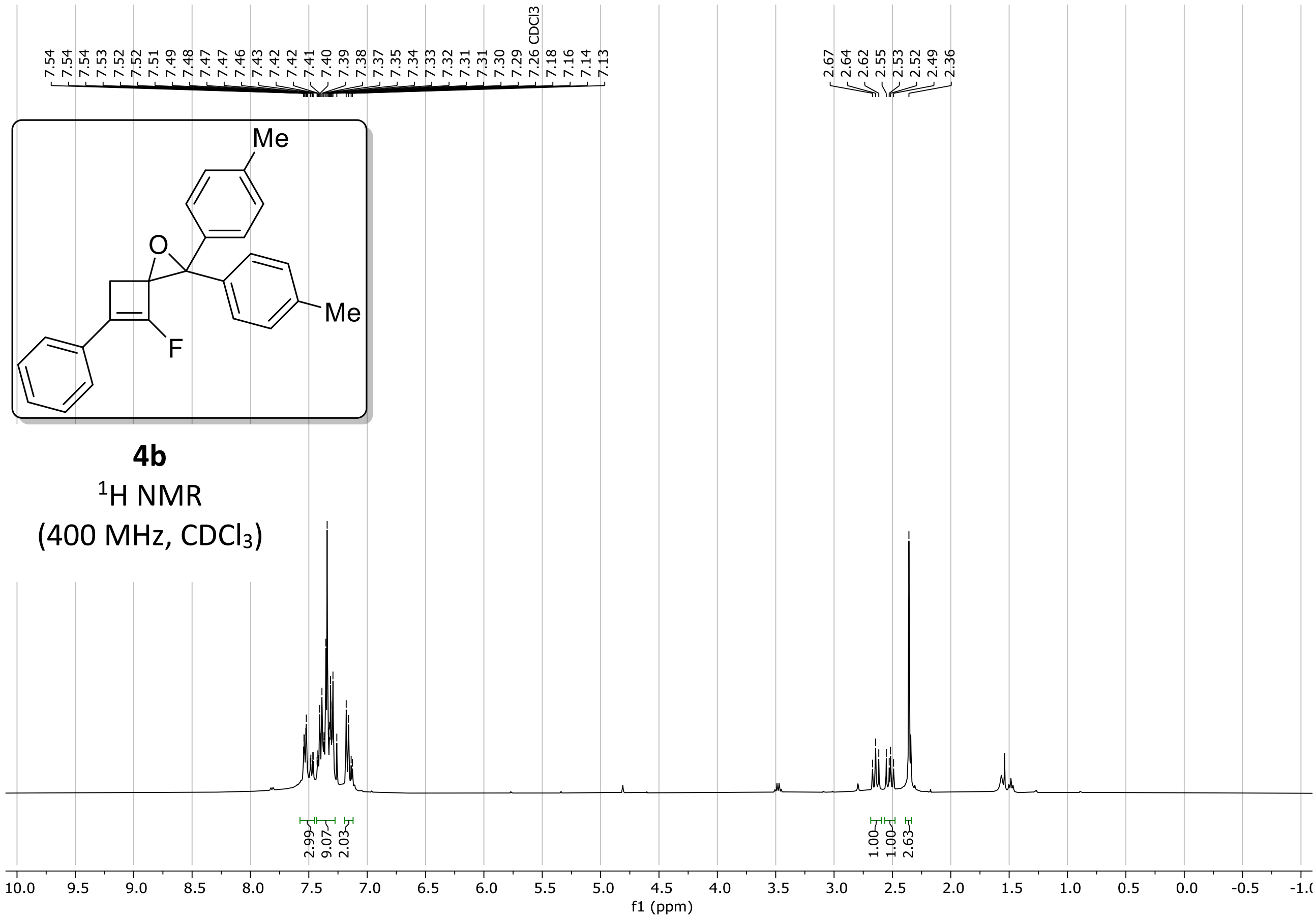

4b
^{1}H NMR
(400 MHz, $CDCl_3$)

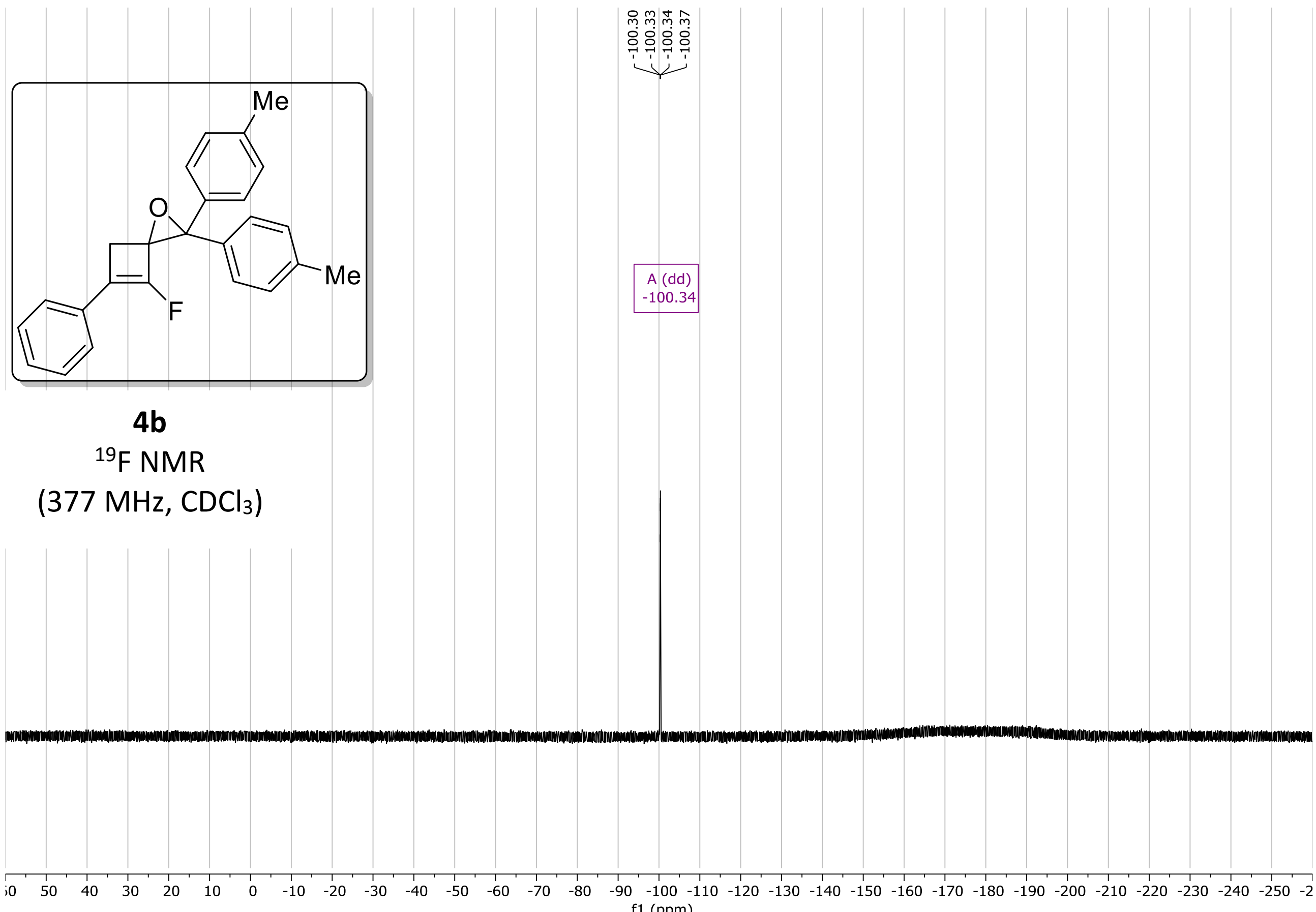

4b
^{19}F NMR
(377 MHz, $CDCl_3$)

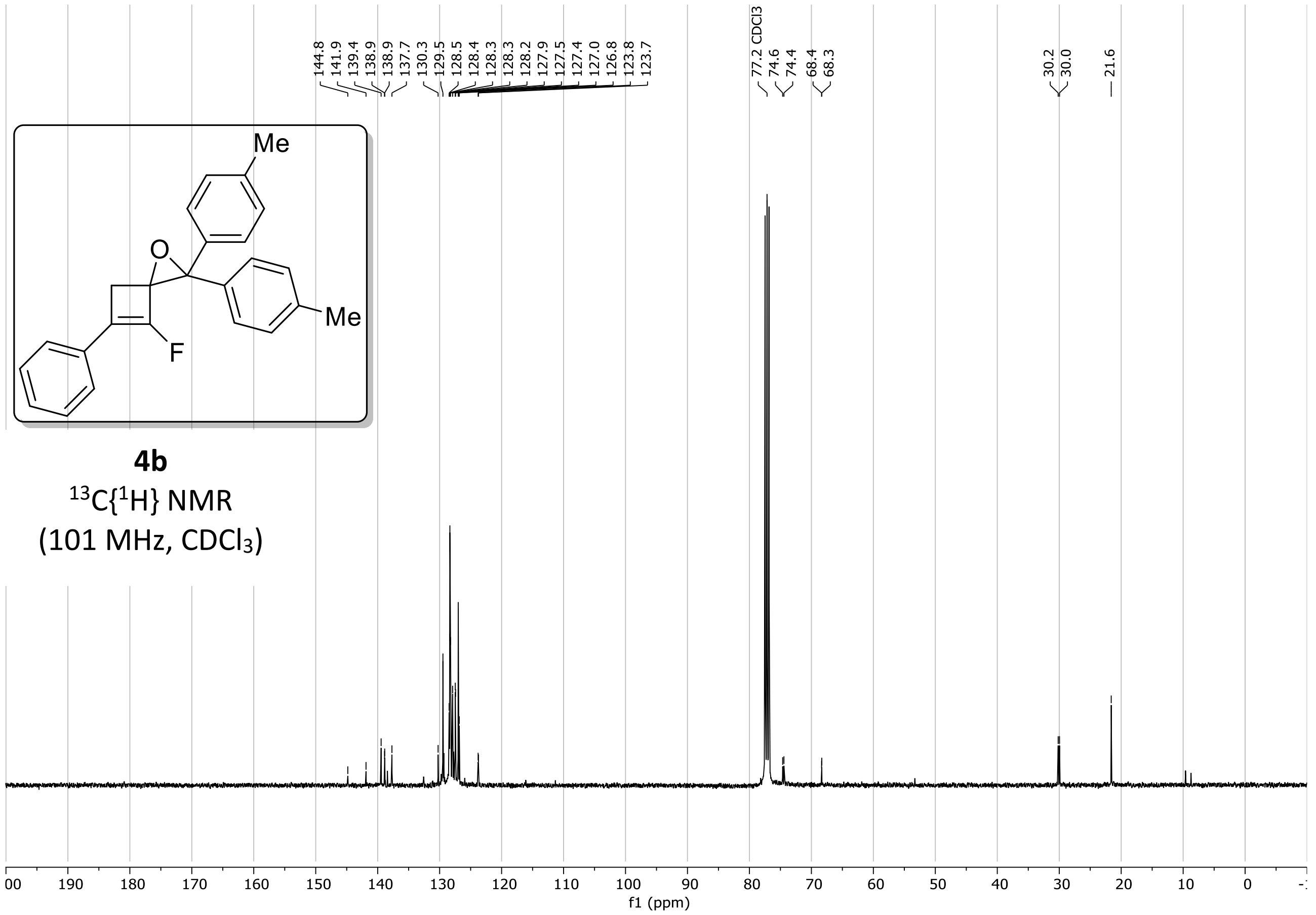
144.8
141.9
139.4
138.9
138.9
137.7
130.3
129.5
128.5
128.4
128.3
128.3
128.2
127.9
127.5
127.4
127.0
126.8
123.8
123.7
77.2 CDCl3
74.6
74.4
68.4
68.3
30.2
30.0
21.6
Me
O
Me
F
4b
13C{1H} NMR
(101 MHz, CDCl3)
00
190
180
170
160
150
140
130
120
110
100
90
80
70
60
50
40
30
20
10
0
f1 (ppm)

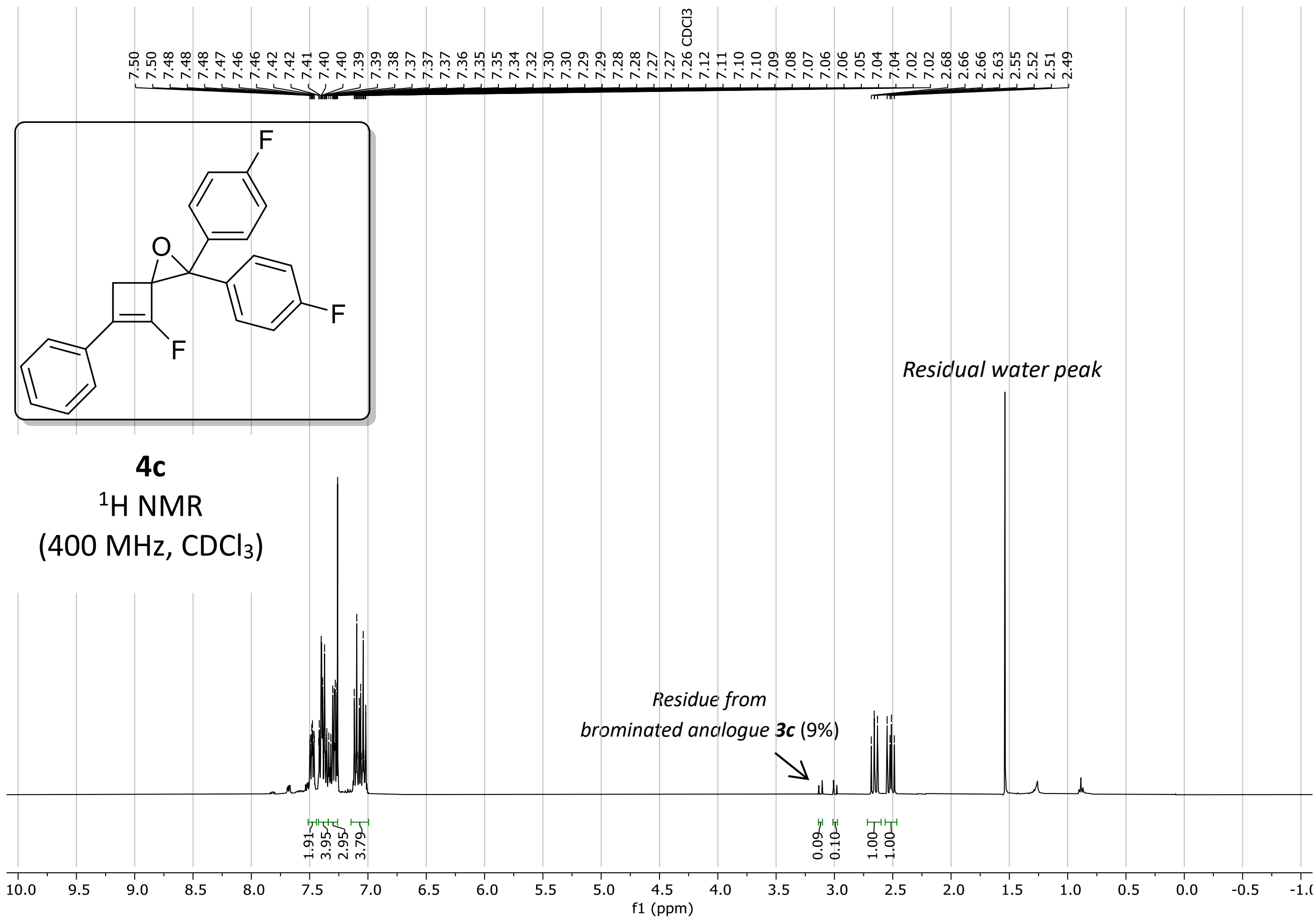
7.50 7.50 7.48 7.48 7.48 7.47 7.46 7.42 7.42 7.41 7.40 7.40 7.40 7.39 7.39 7.38 7.37 7.37 7.36 7.35 7.34 7.32 7.30 7.30 7.29 7.29 7.28 7.28 7.27 7.26 CDCl3 7.12 7.11 7.10 7.10 7.09 7.08 7.07 7.06 7.06 7.05 7.04 7.04 7.02 7.02 2.68 2.66 2.66 2.63 2.55 2.52 2.51 2.49
F
O
F
F
4c
^{1}H NMR
(400 MHz, $CDCl_3$)
Residual water peak
Residue from
brominated analogue 3c (9%)
1.91 3.95 2.95 3.79 0.09 0.10 1.00 1.00
f1 (ppm)

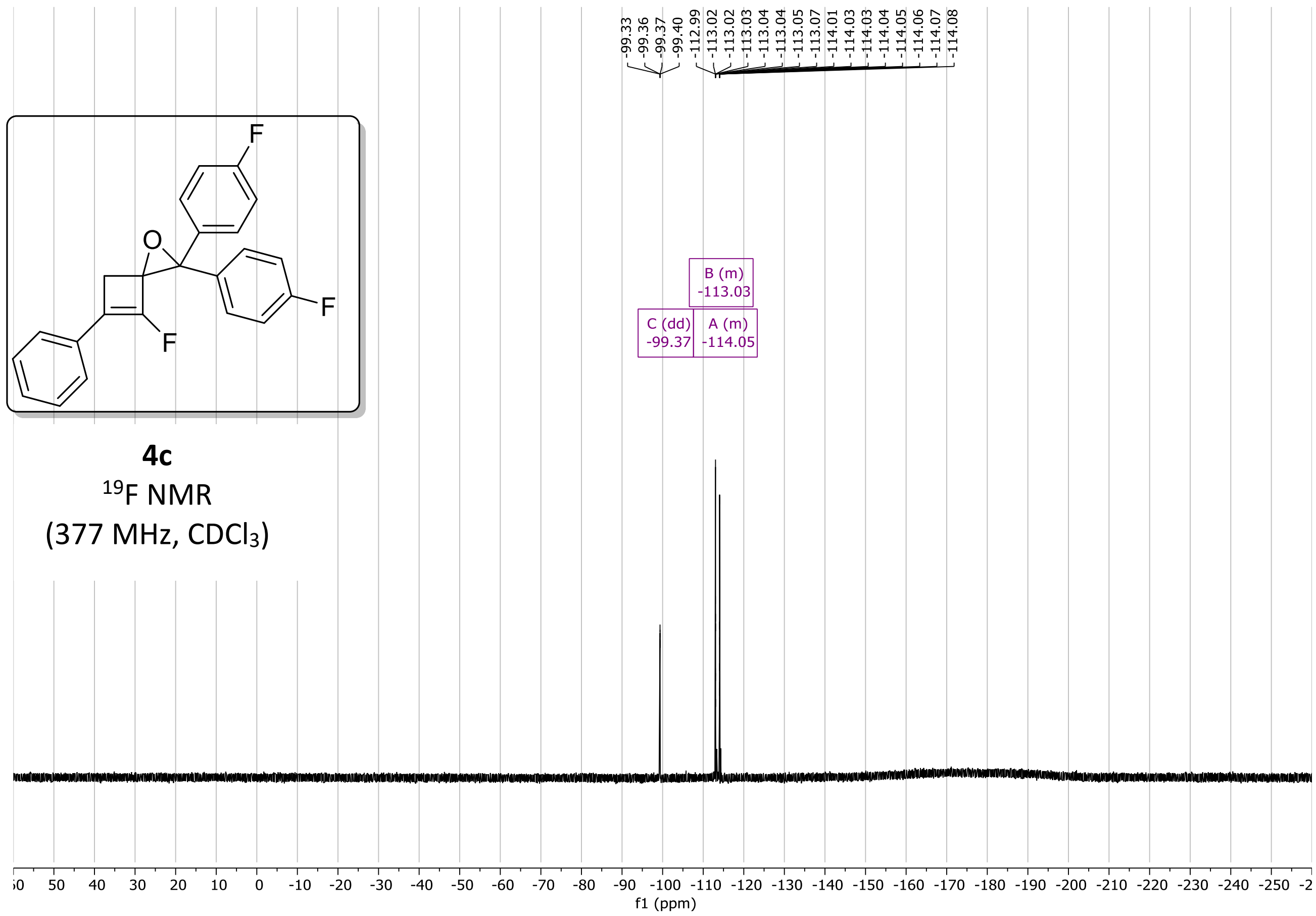
-99.33 -99.36 -99.37 -99.40 -112.99 -113.02 -113.02 -113.03 -113.04 -113.05 -113.07 -114.01 -114.03 -114.03 -114.04 -114.05 -114.06 -114.07 -114.08
F
O
F
F
4c
^{19}F NMR
(377 MHz, $CDCl_3$)
B (m)
-113.03
C (dd)
-99.37
A (m)
-114.05
f1 (ppm)

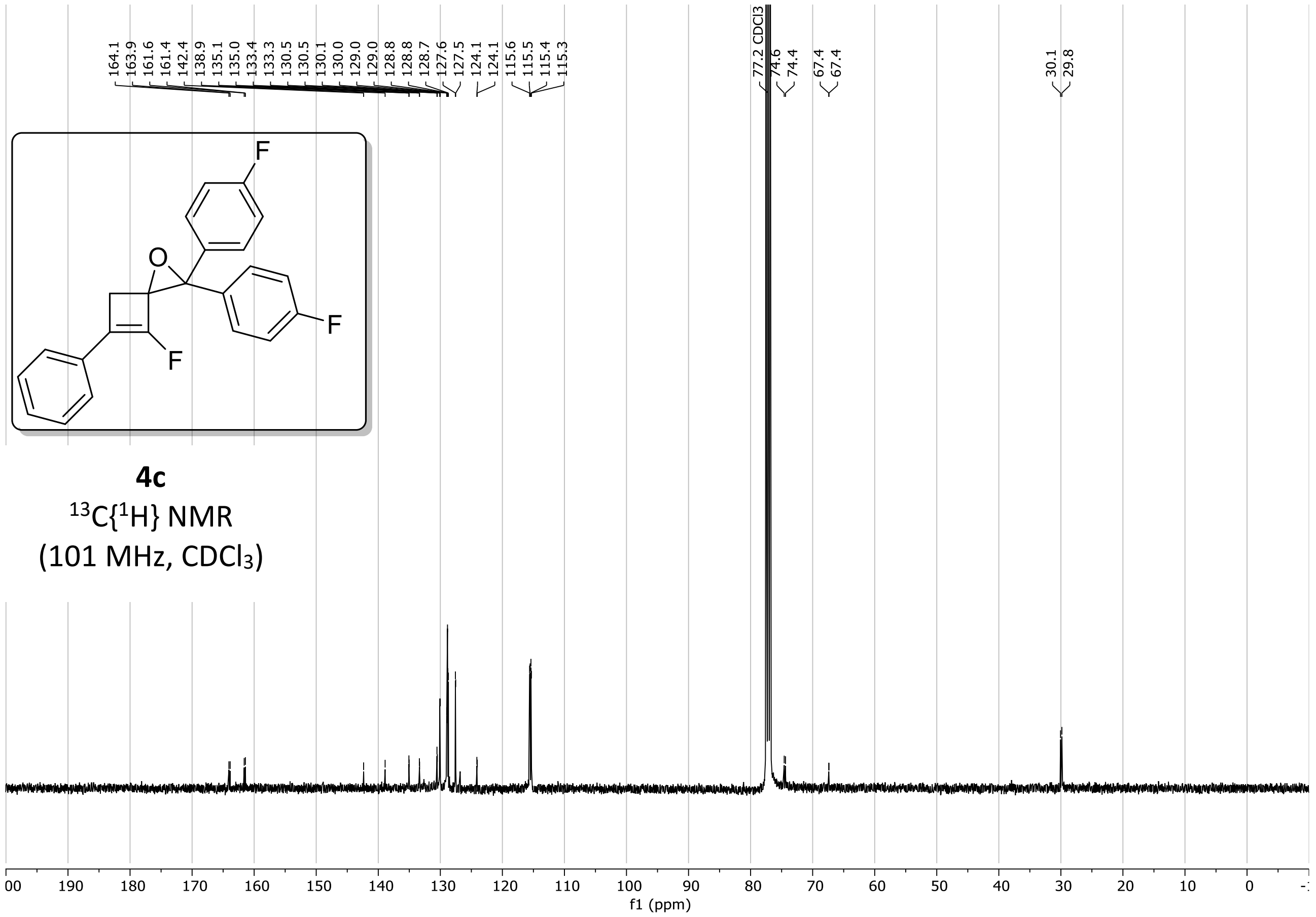

4c
$^{13}C\{^{1}H\}$ NMR
(101 MHz, $CDCl_3$)

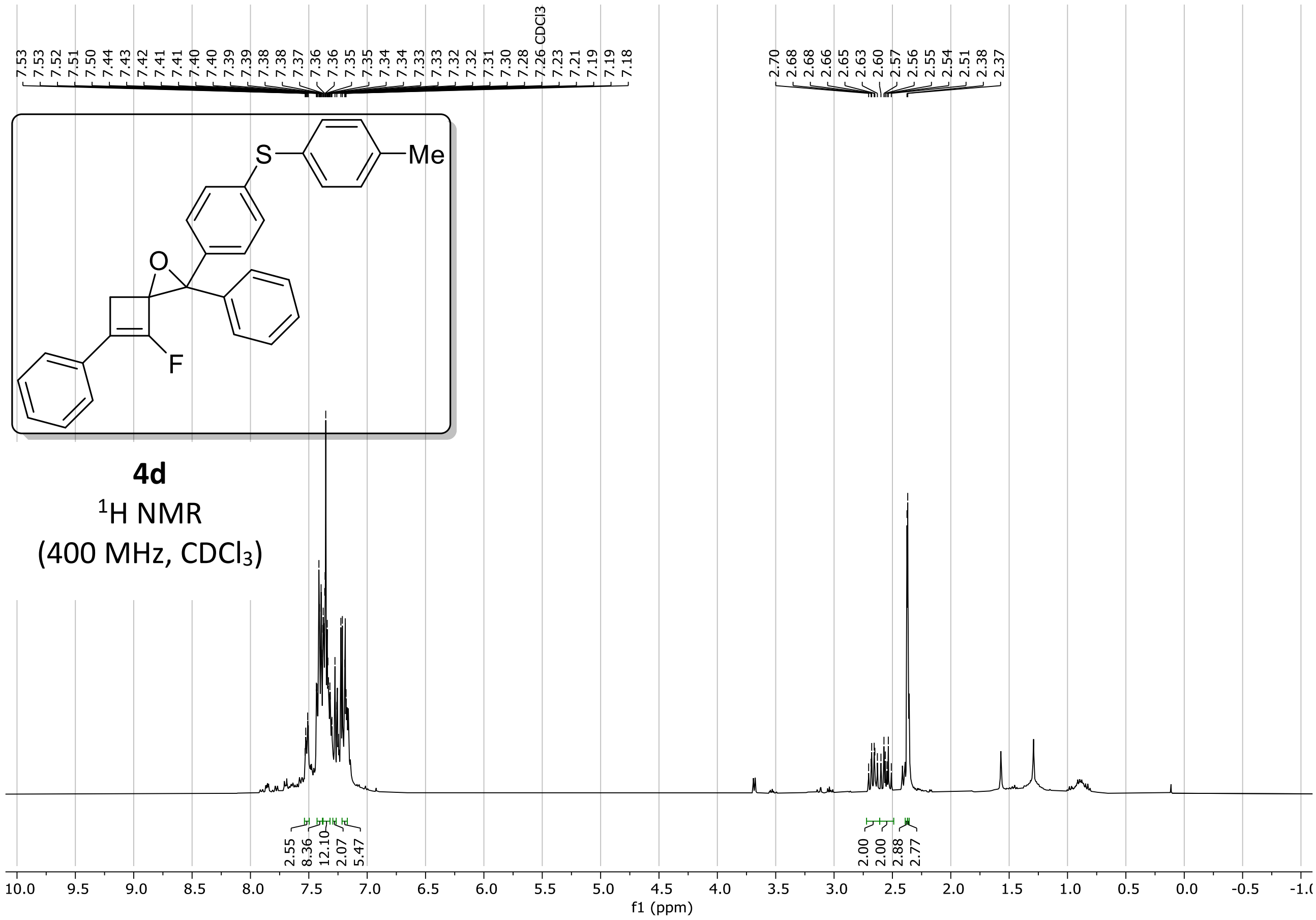
7.53 7.53 7.52 7.51 7.50 7.44 7.43 7.42 7.41 7.41 7.40 7.40 7.39 7.39 7.38 7.38 7.37 7.36 7.36 7.35 7.35 7.34 7.34 7.33 7.33 7.32 7.32 7.31 7.30 7.28 7.26 CDCl3 7.23 7.21 7.19 7.18
2.70 2.68 2.68 2.66 2.65 2.63 2.60 2.57 2.56 2.55 2.54 2.51 2.38 2.37
S
Me
O
F
4d
^{1}H NMR
(400 MHz, $CDCl_3$)
2.55 8.36 12.10 2.07 5.47
2.00 2.00 2.88 2.77
f1 (ppm)

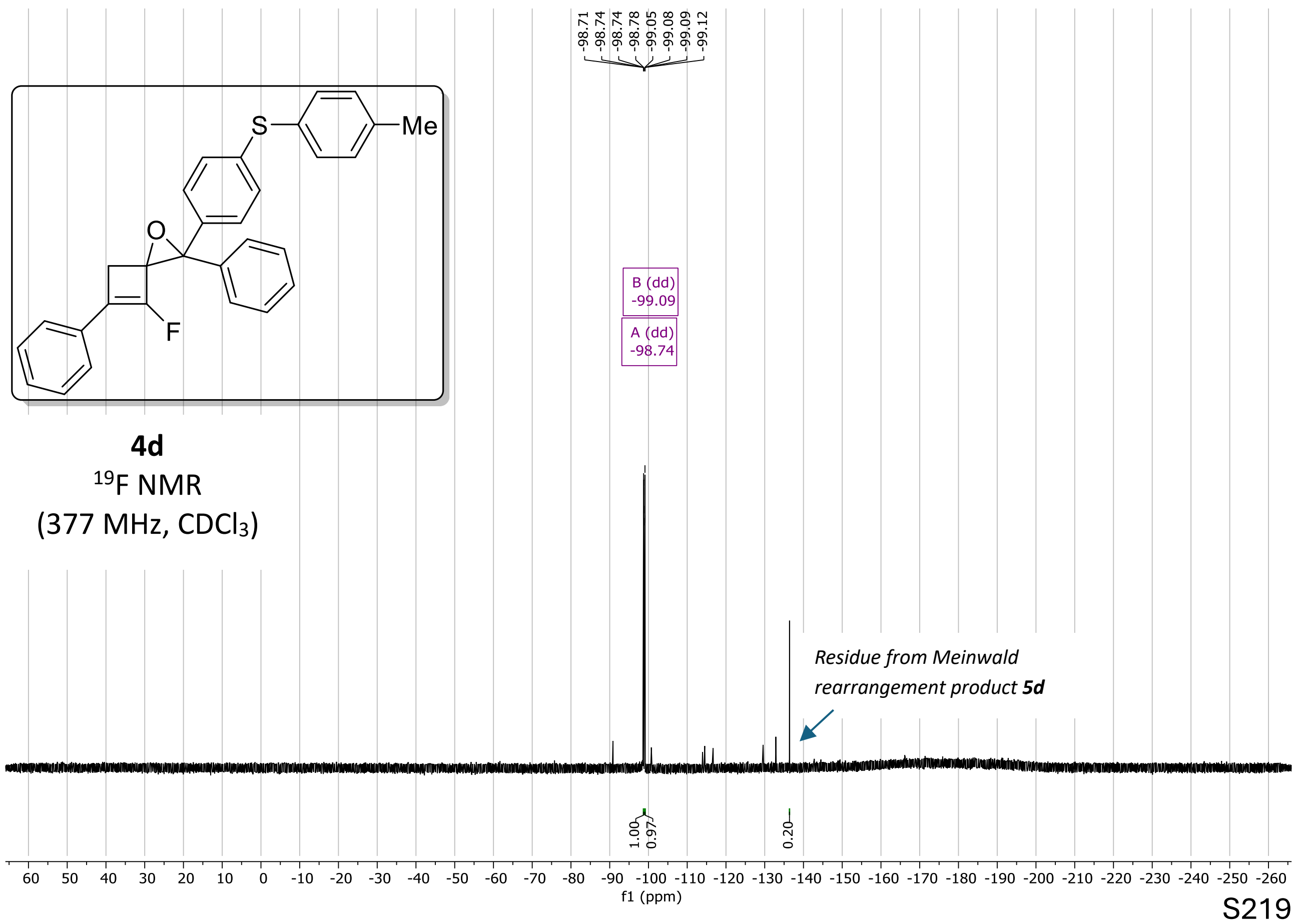
-98.71 -98.74 -98.74 -98.78 -99.05 -99.08 -99.09 -99.12
S
Me
O
F
B (dd)
-99.09
A (dd)
-98.74
4d
^{19}F NMR
(377 MHz, $CDCl_3$)
*Residue from Meinwald rearrangement product **5d***
1.00 0.97
0.20
f1 (ppm)

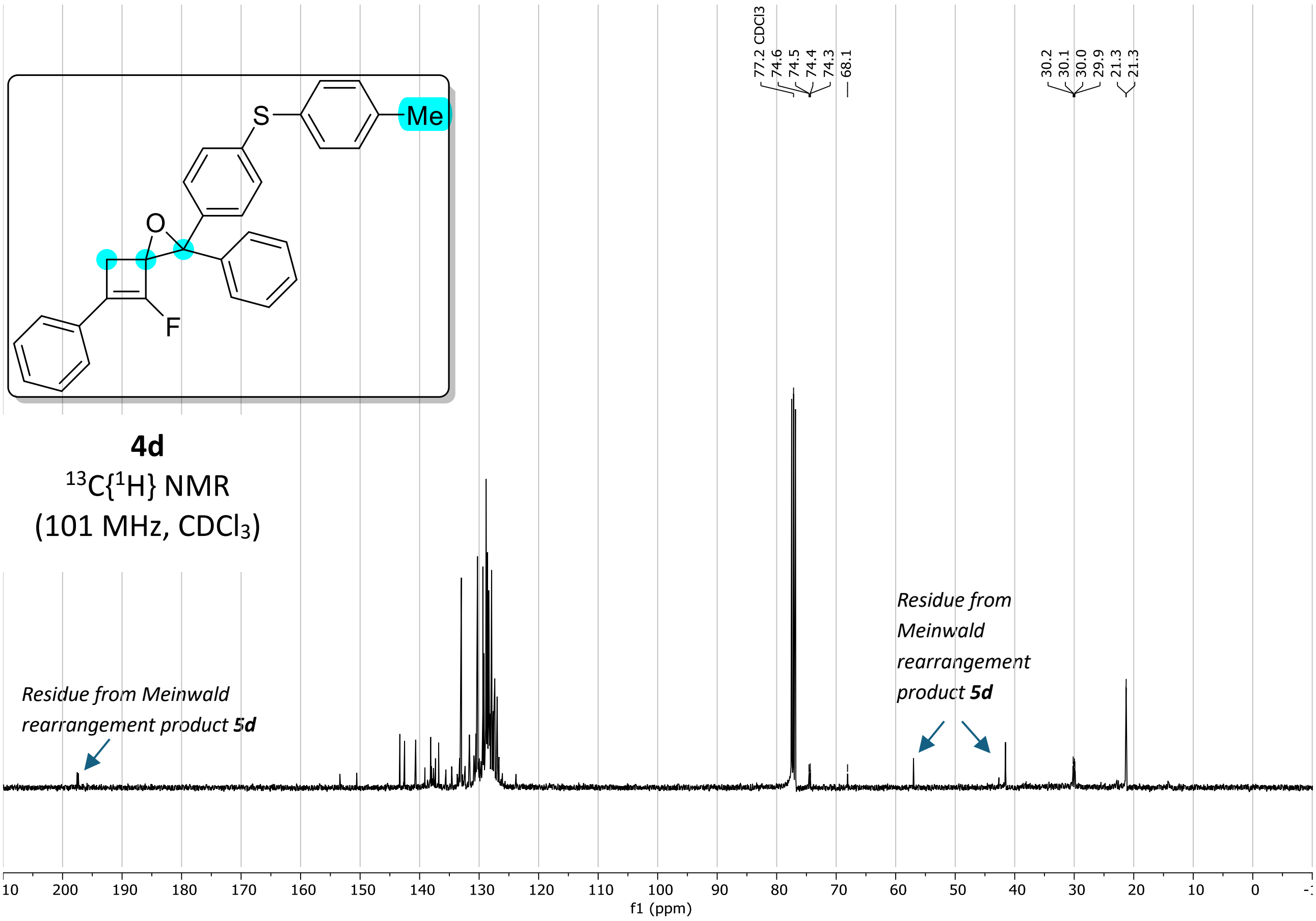

S
Me
O
F
4d
$^{13}C\{^{1}H\}$ NMR
(101 MHz, $CDCl_3$)
77.2 CDCl3
74.6
74.5
74.4
74.3
68.1
30.2
30.1
30.0
29.9
21.3
21.3
Residue from Meinwald rearrangement product **5d**
Residue from Meinwald rearrangement product **5d**
10 200 190 180 170 160 150 140 130 120 110 100 90 80 70 60 50 40 30 20 10 0
f1 (ppm)

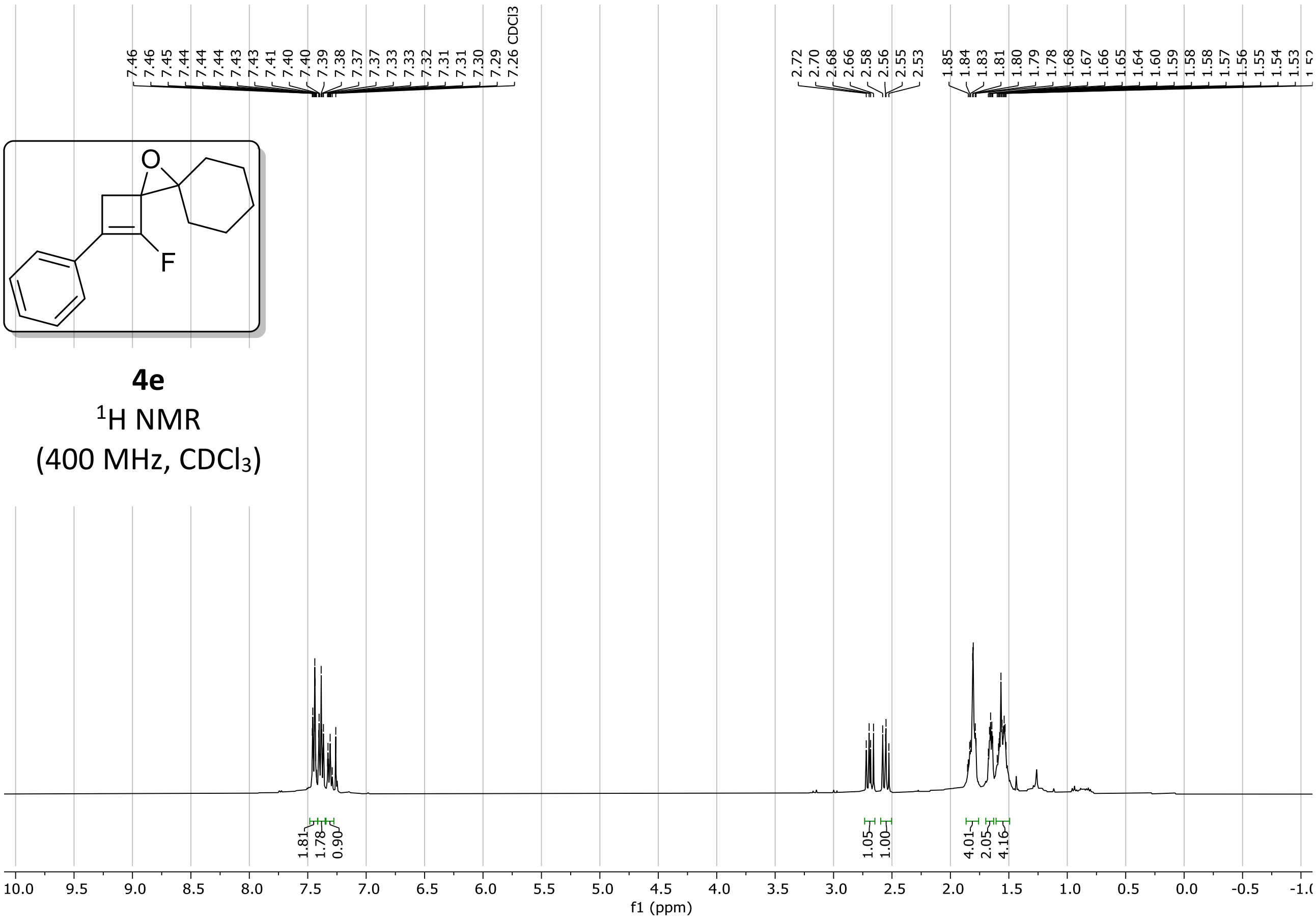

4e
^{1}H NMR
(400 MHz, $CDCl_3$)

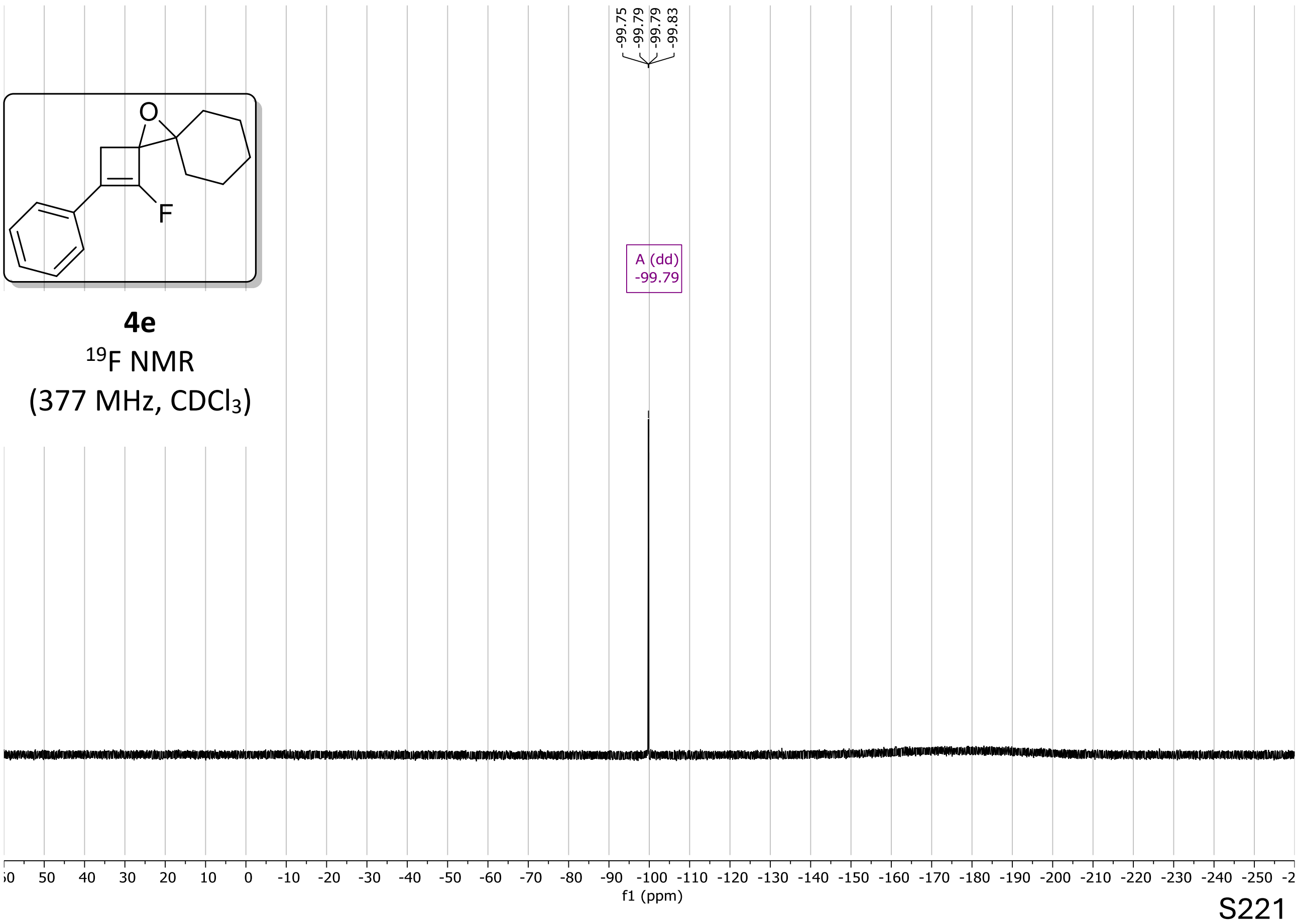

4e
^{19}F NMR
(377 MHz, $CDCl_3$)

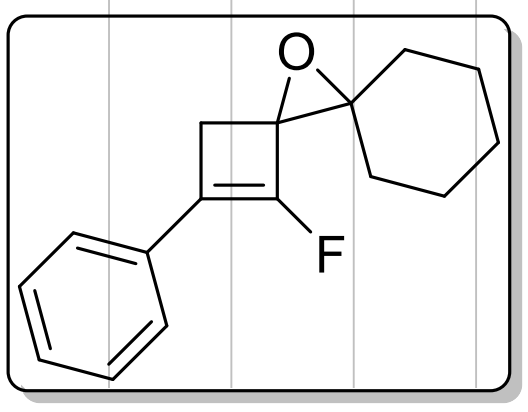

4e

$^{13}C\{^{1}H\}$ NMR

(101 MHz, $CDCl_3$)

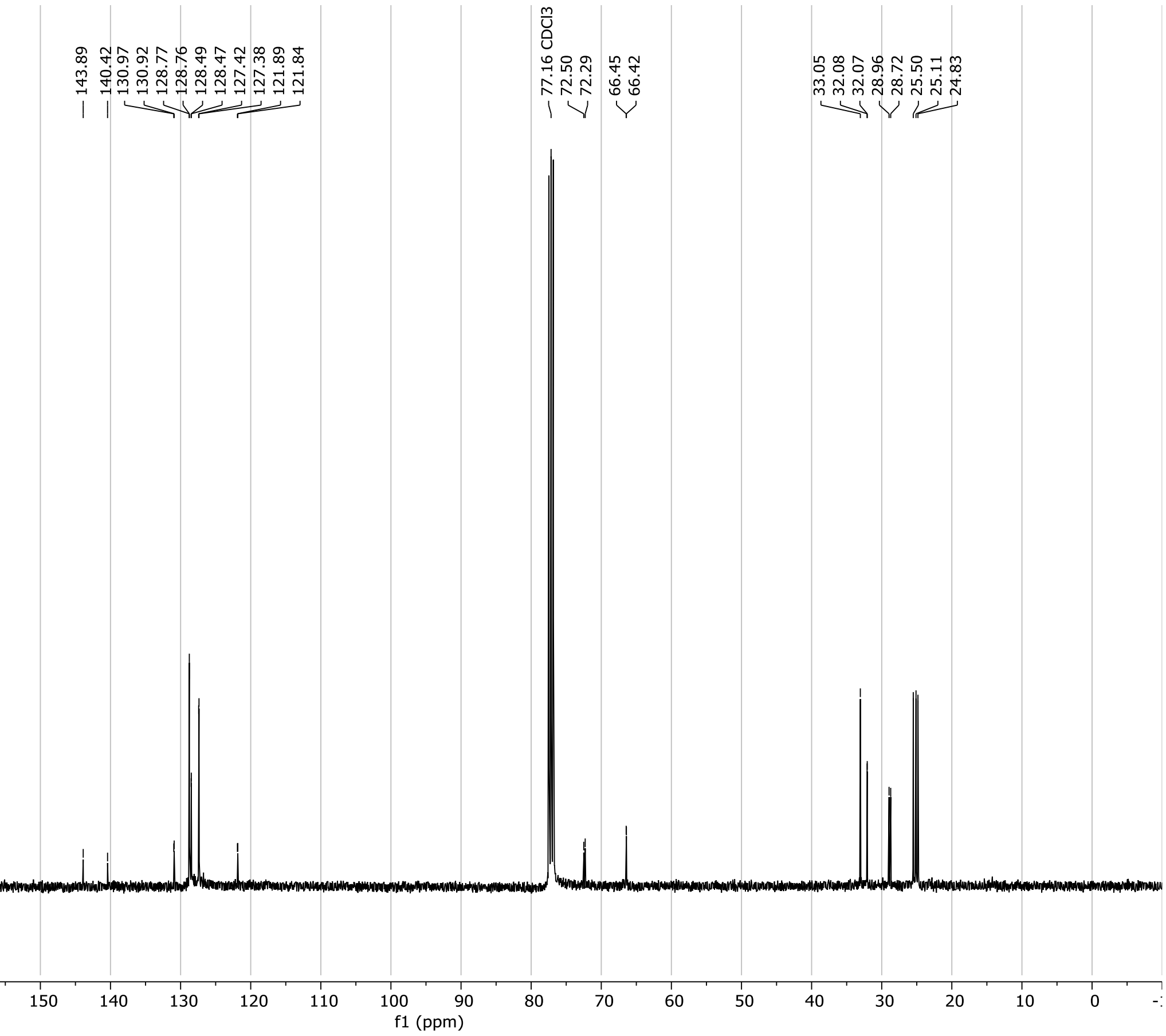

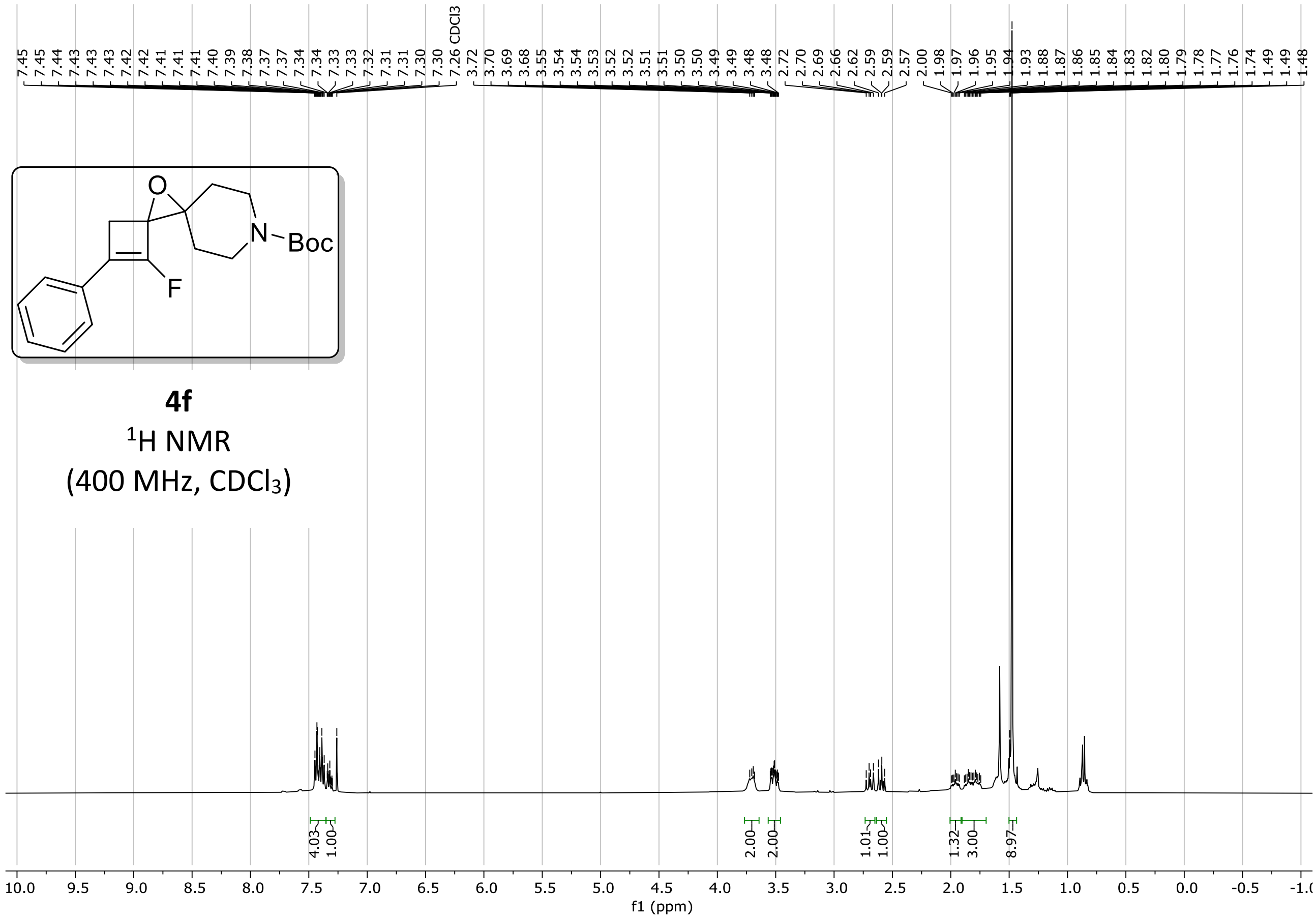

4f

^{1}H NMR

(400 MHz, $CDCl_3$)

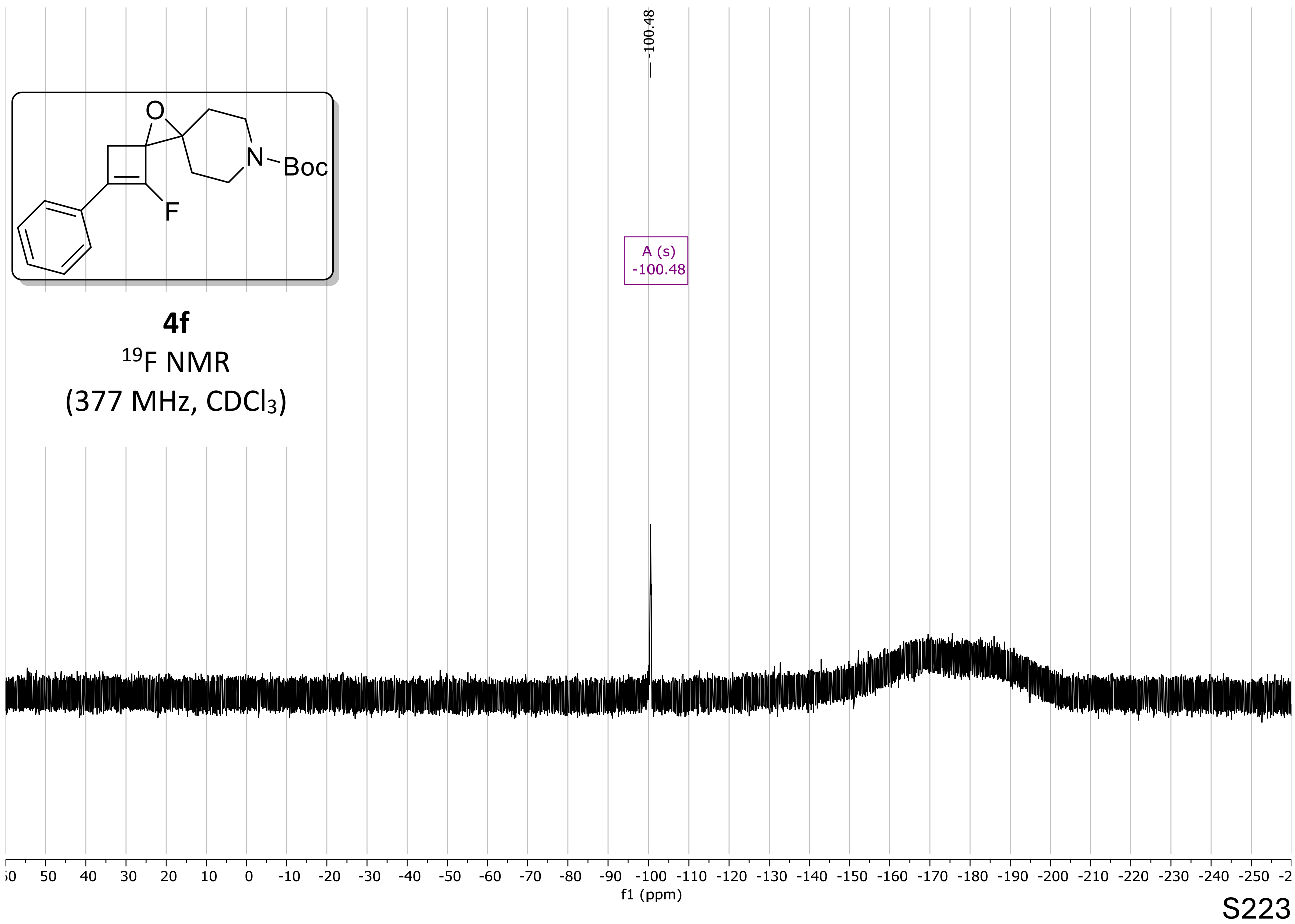

4f

^{19}F NMR

(377 MHz, $CDCl_3$)

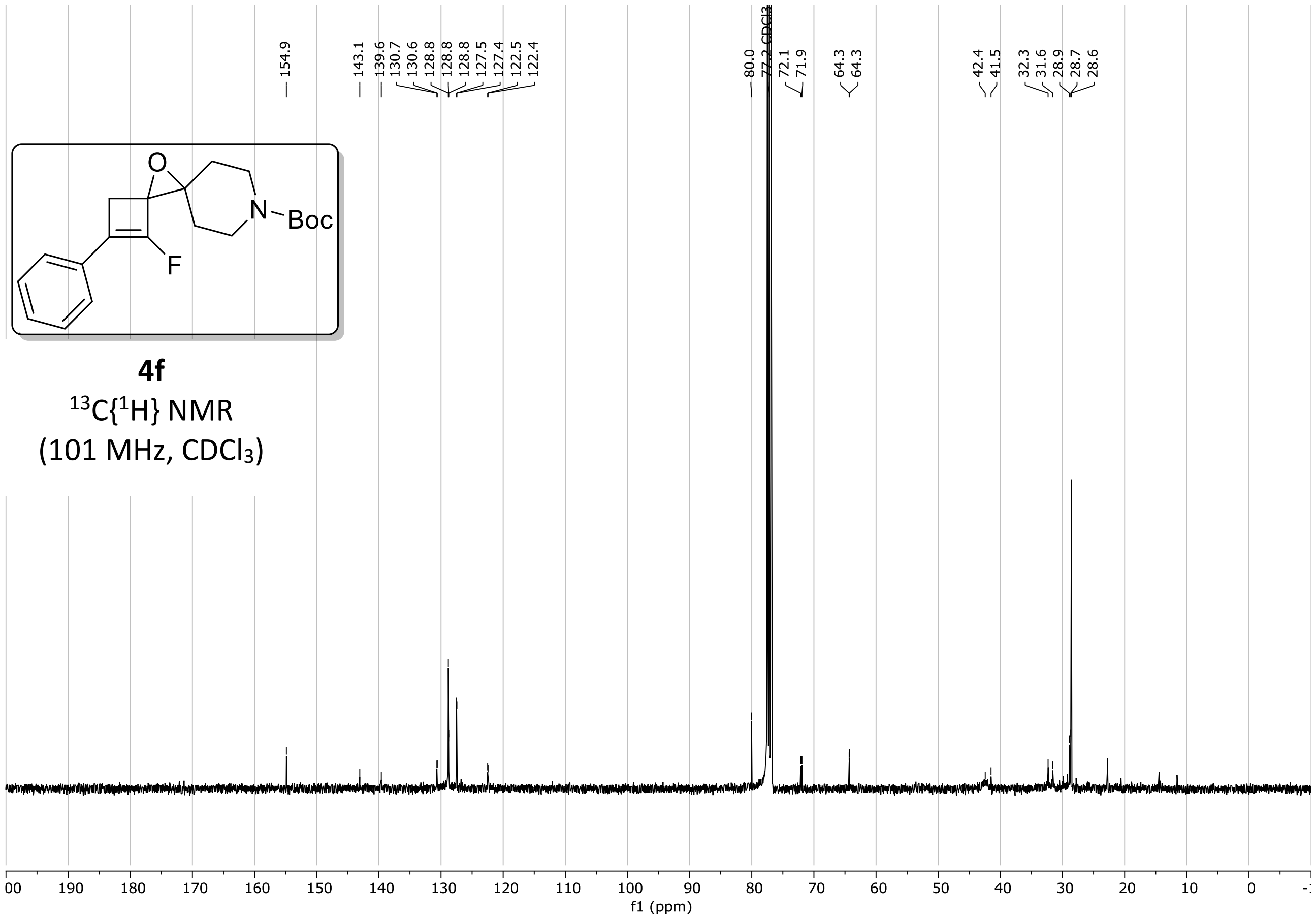
O
N
Boc
F
4f
$^{13}C\{^1H\}$ NMR
(101 MHz, $CDCl_3$)
154.9
143.1
139.6
130.7
130.6
128.8
128.8
128.8
127.5
127.4
122.5
122.4
80.0
77.2 CDCl3
72.1
71.9
64.3
64.3
42.4
41.5
32.3
31.6
28.9
28.7
28.6
f1 (ppm)

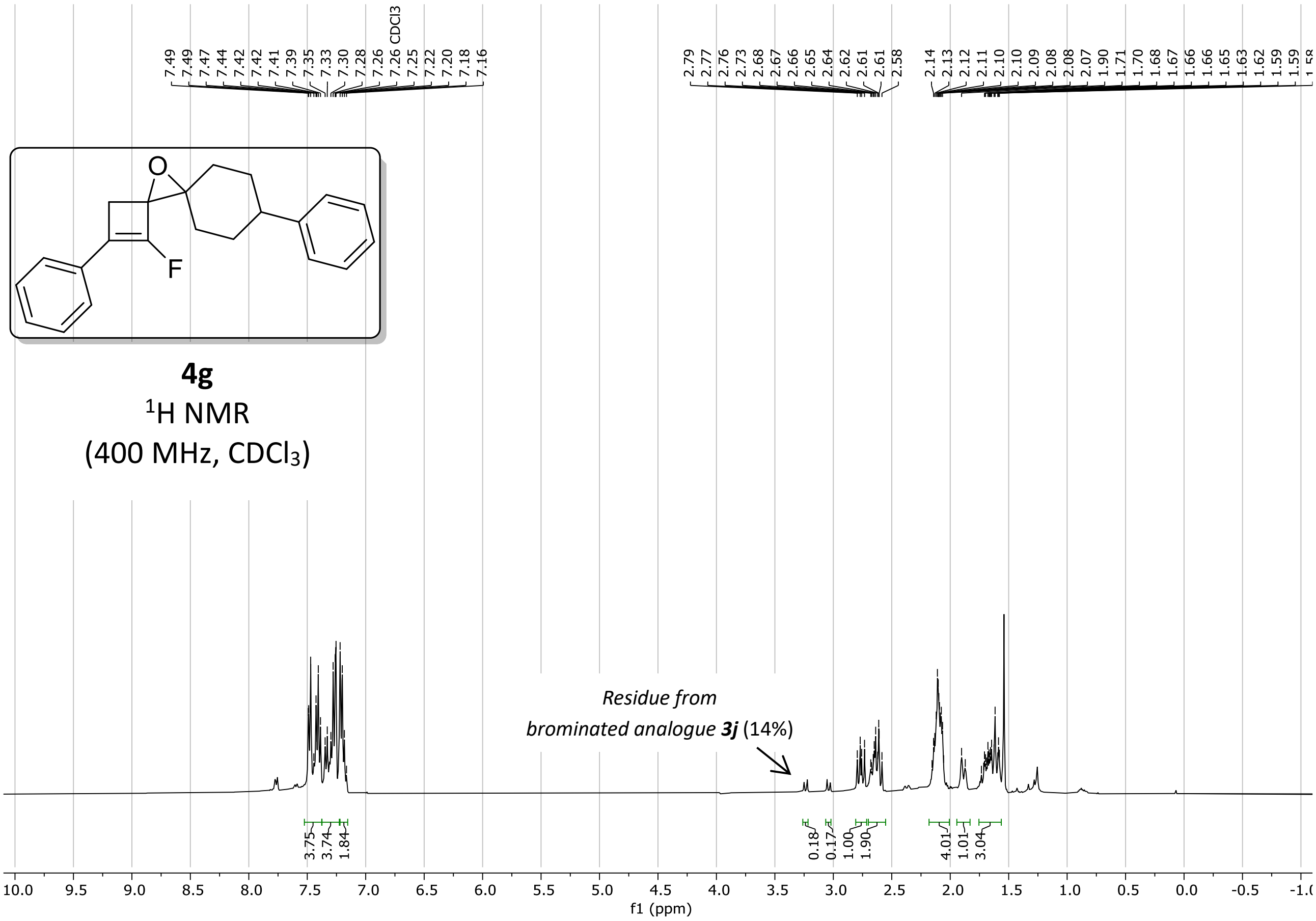

CDCl3
O
F
4g
¹H NMR
(400 MHz, CDCl₃)
Residue from
brominated analogue 3j (14%)
f1 (ppm)

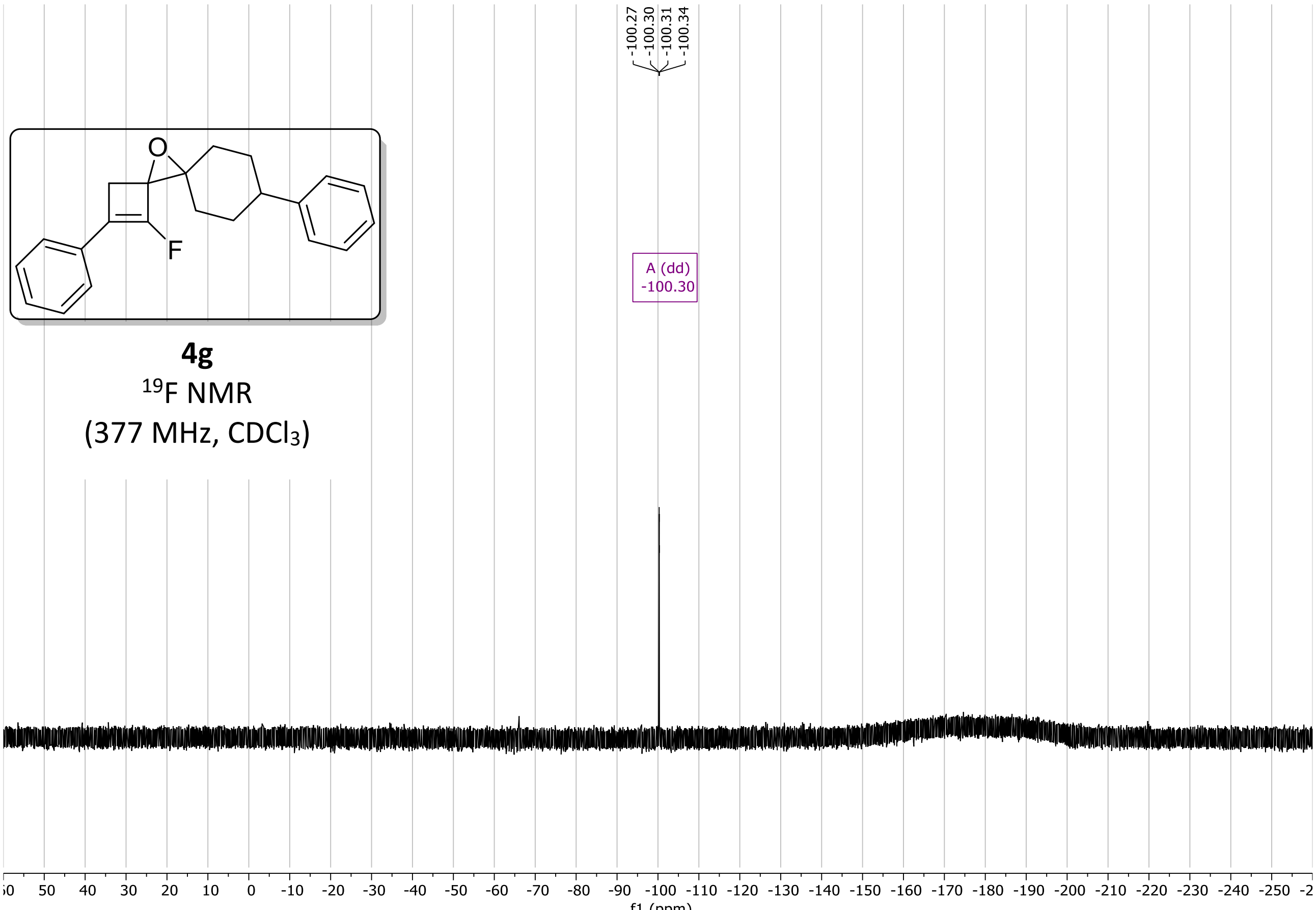

-100.27
-100.30
-100.31
-100.34
O
F
A (dd)
-100.30
4g
¹⁹F NMR
(377 MHz, CDCl₃)
f1 (ppm)

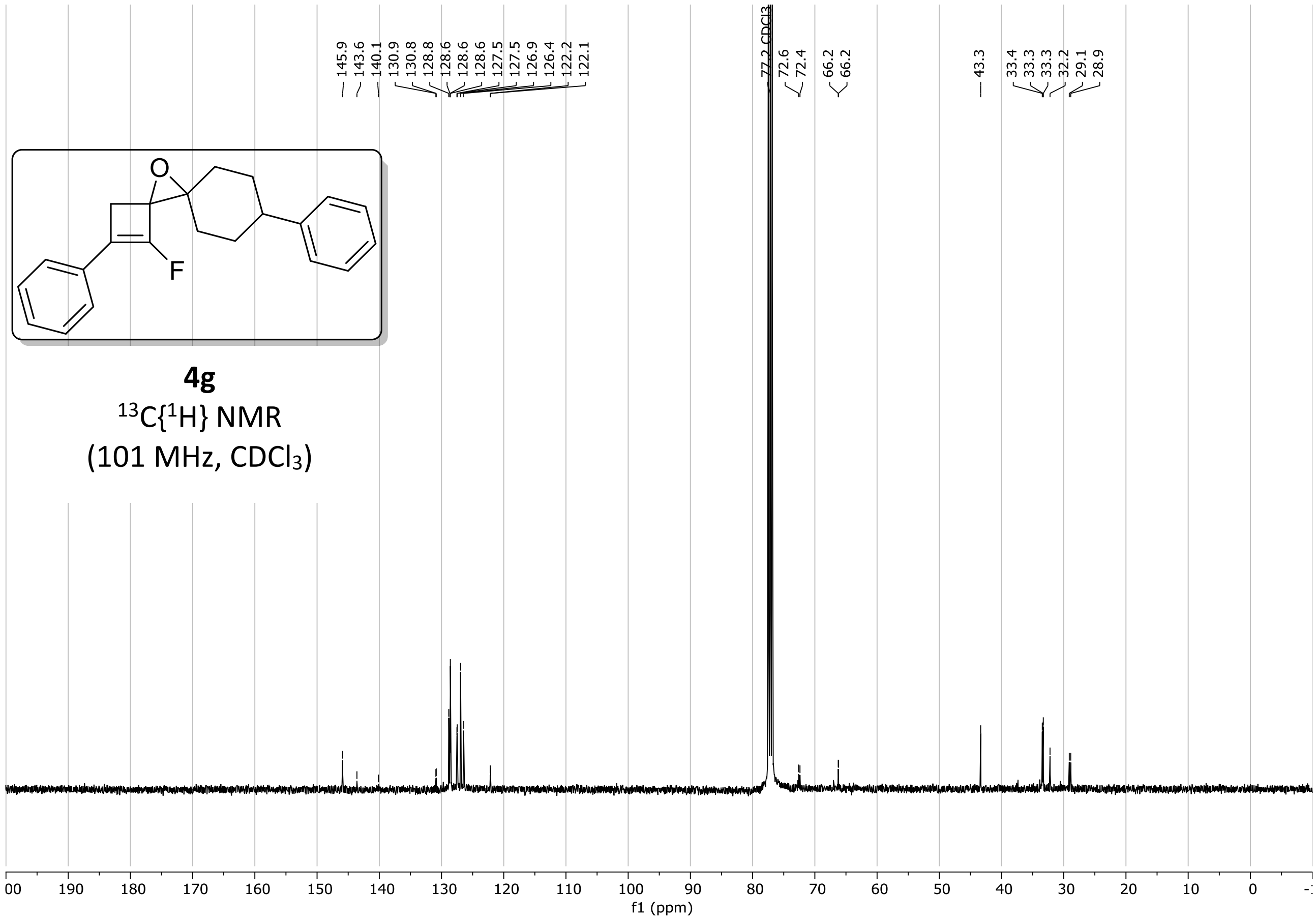

4g

$^{13}C\{^1H\}$ NMR

(101 MHz, $CDCl_3$)

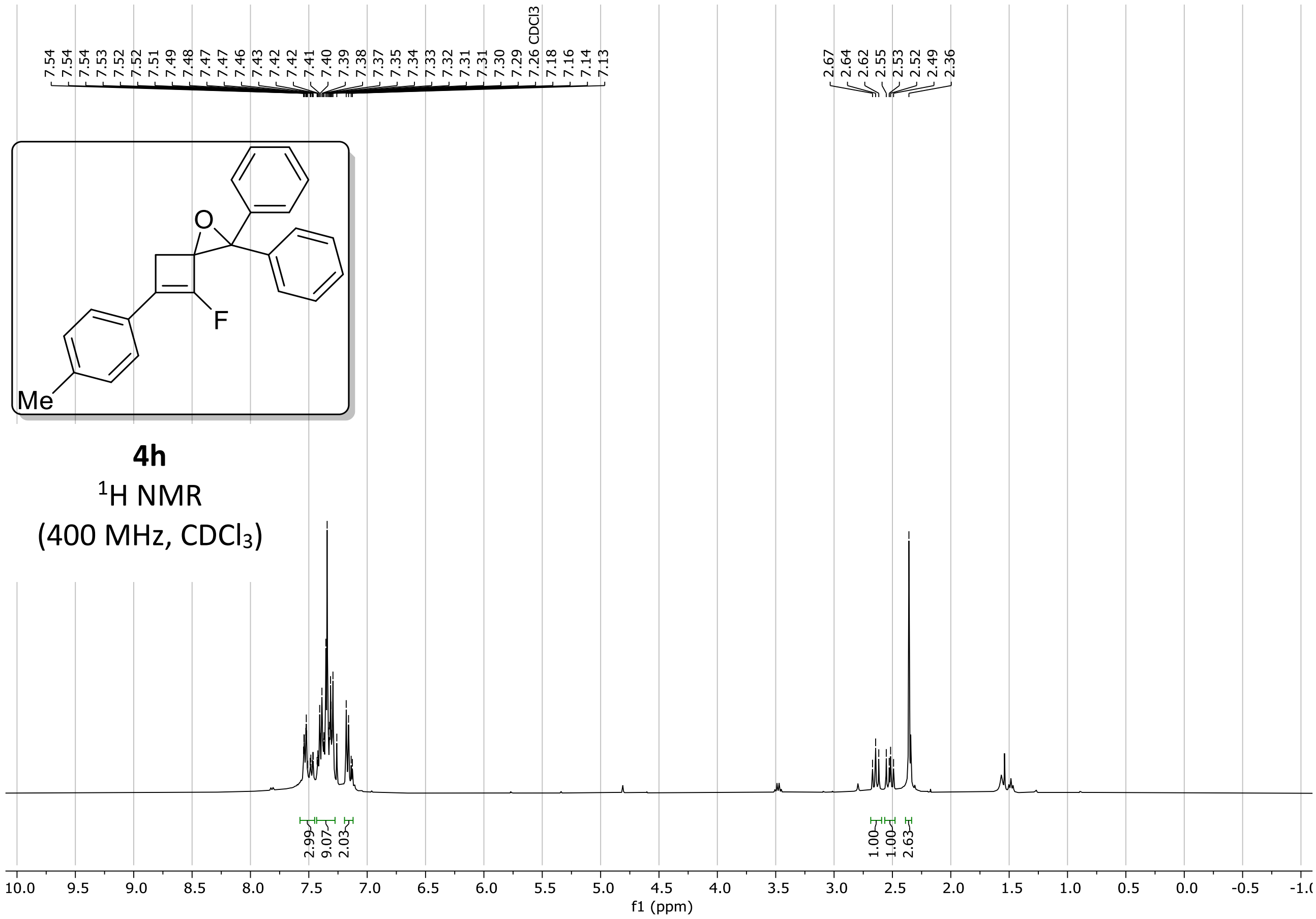

4h
^{1}H NMR
(400 MHz, $CDCl_3$)

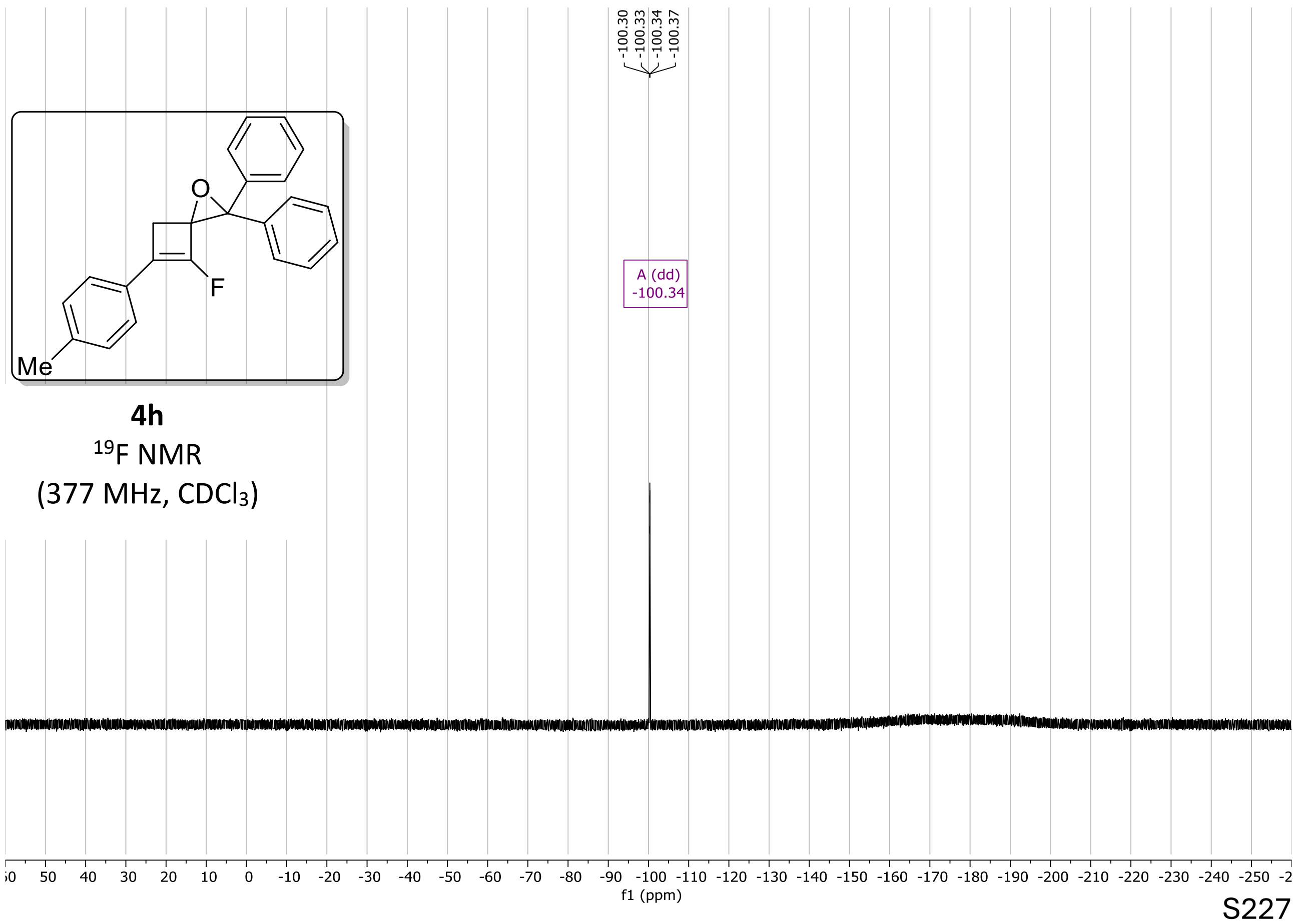

4h
^{19}F NMR
(377 MHz, $CDCl_3$)

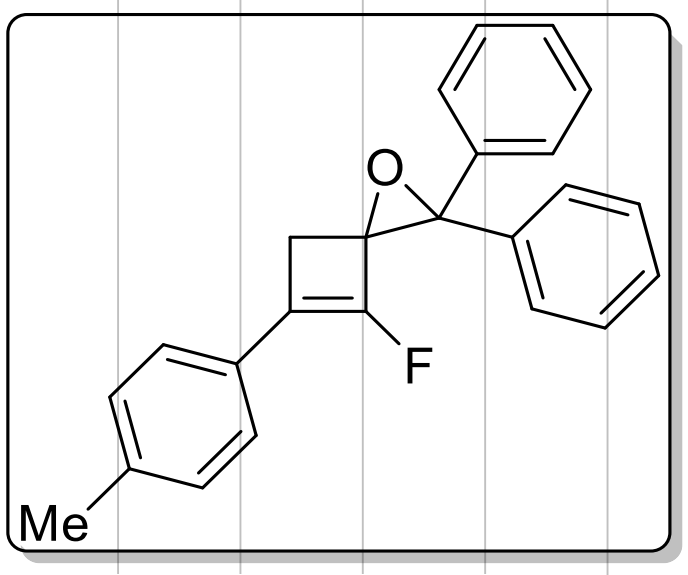

4h

$^{13}C\{^1H\}$ NMR

(101 MHz, $CDCl_3$)

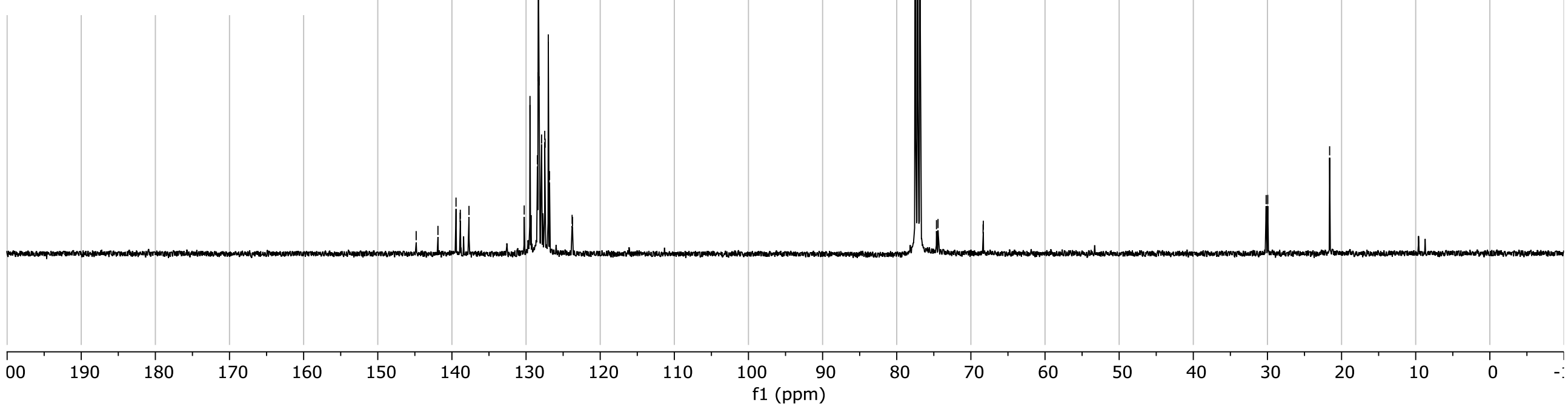

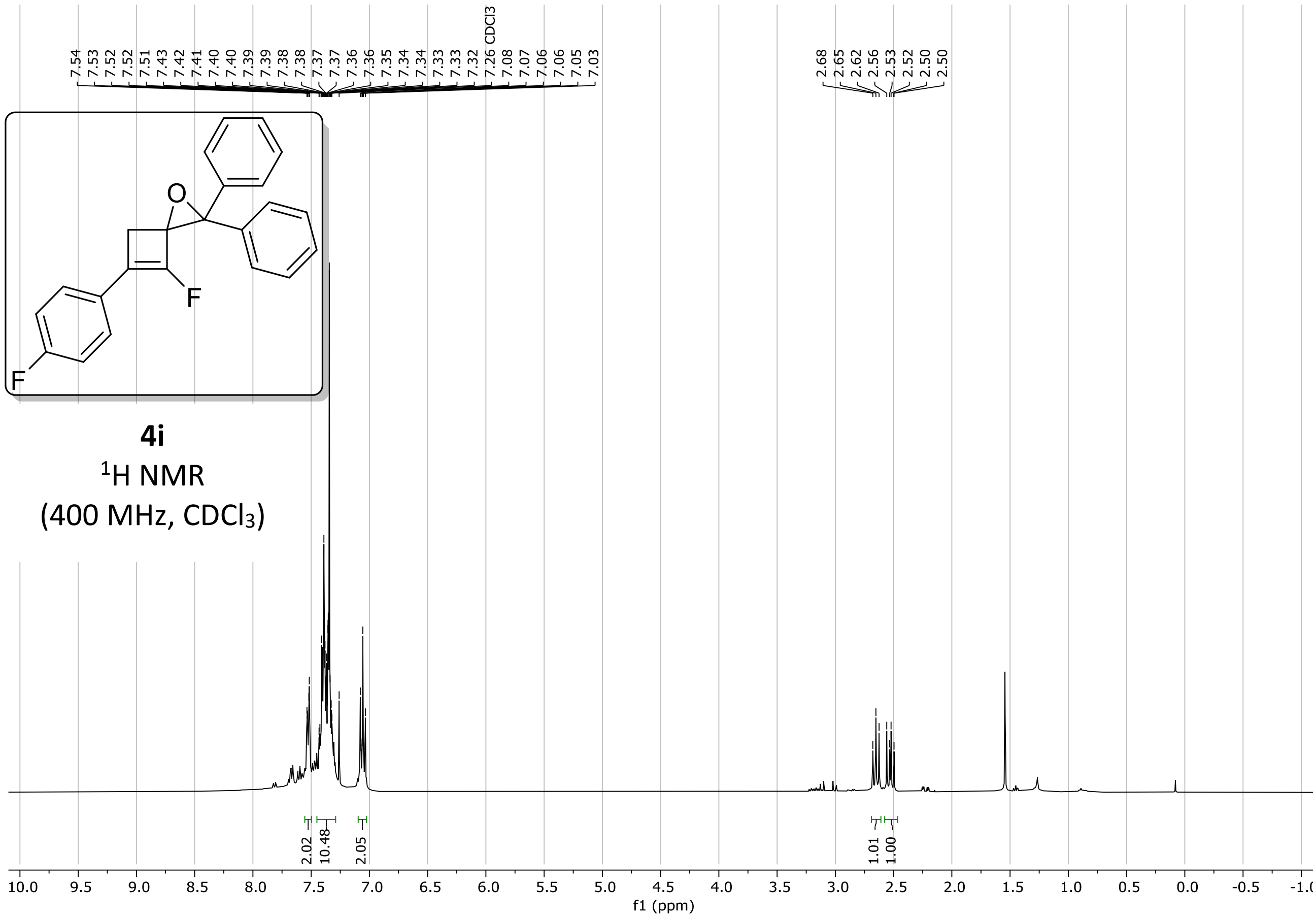

4i
^{1}H NMR
(400 MHz, $CDCl_3$)

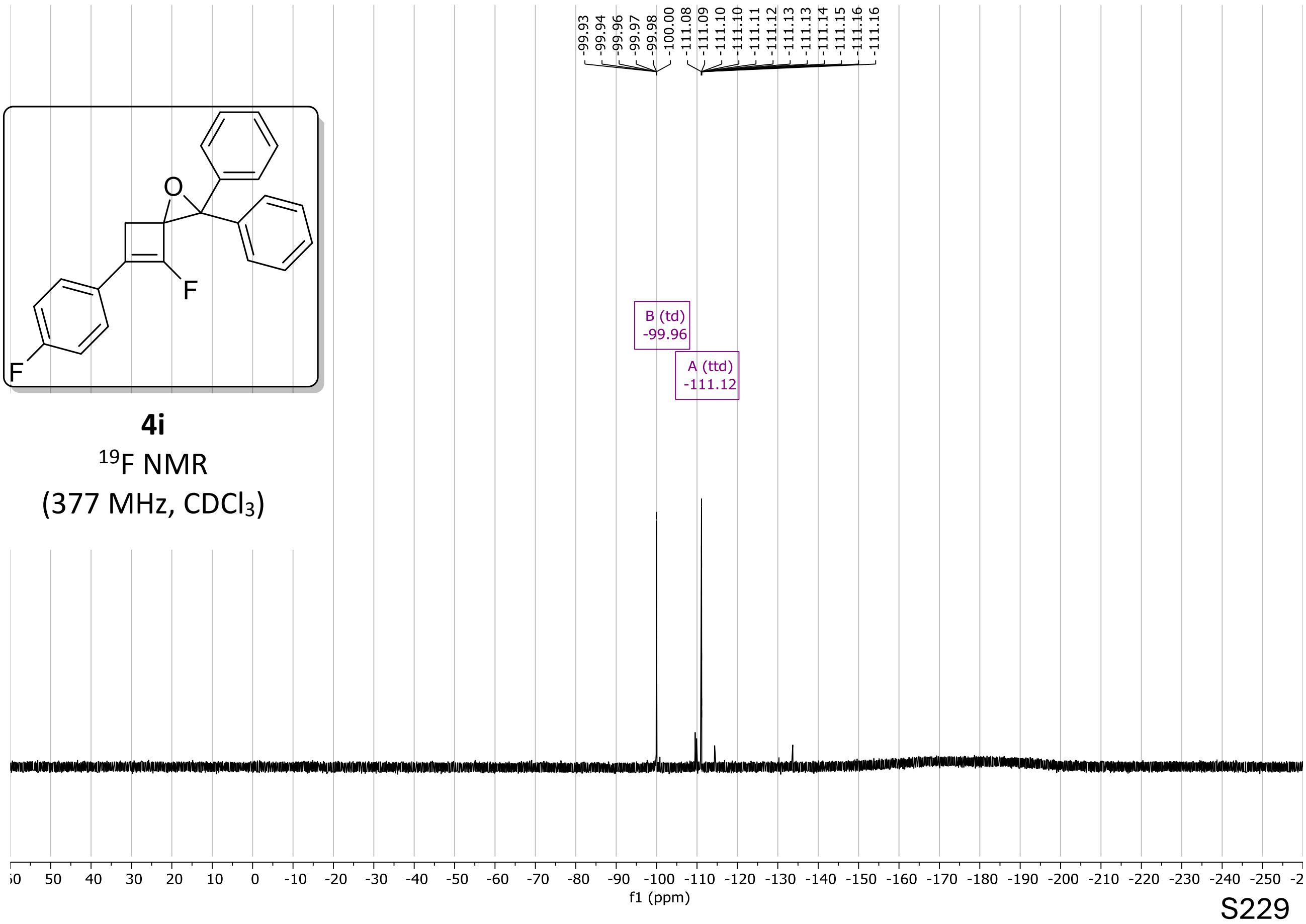

4i
^{19}F NMR
(377 MHz, $CDCl_3$)

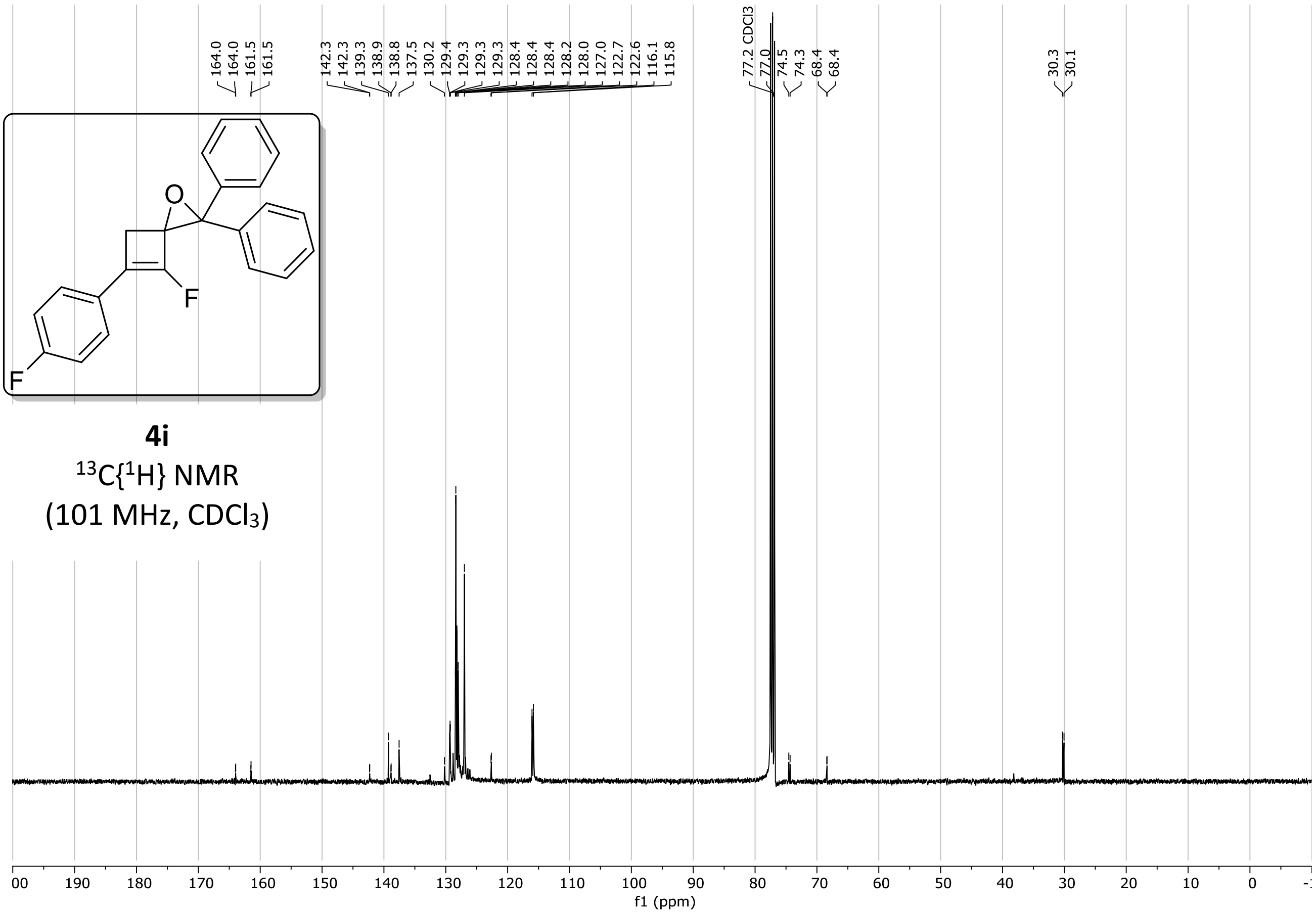

4i
$^{13}C\{^1H\}$ NMR
(101 MHz, $CDCl_3$)
164.0
164.0
161.5
161.5
142.3
142.3
139.3
138.9
138.8
137.5
130.2
129.4
129.3
129.3
129.3
128.4
128.4
128.4
128.2
128.0
127.0
122.7
122.6
116.1
115.8
77.2 CDCl3
77.0
74.5
74.3
68.4
68.4
30.3
30.1
f1 (ppm)

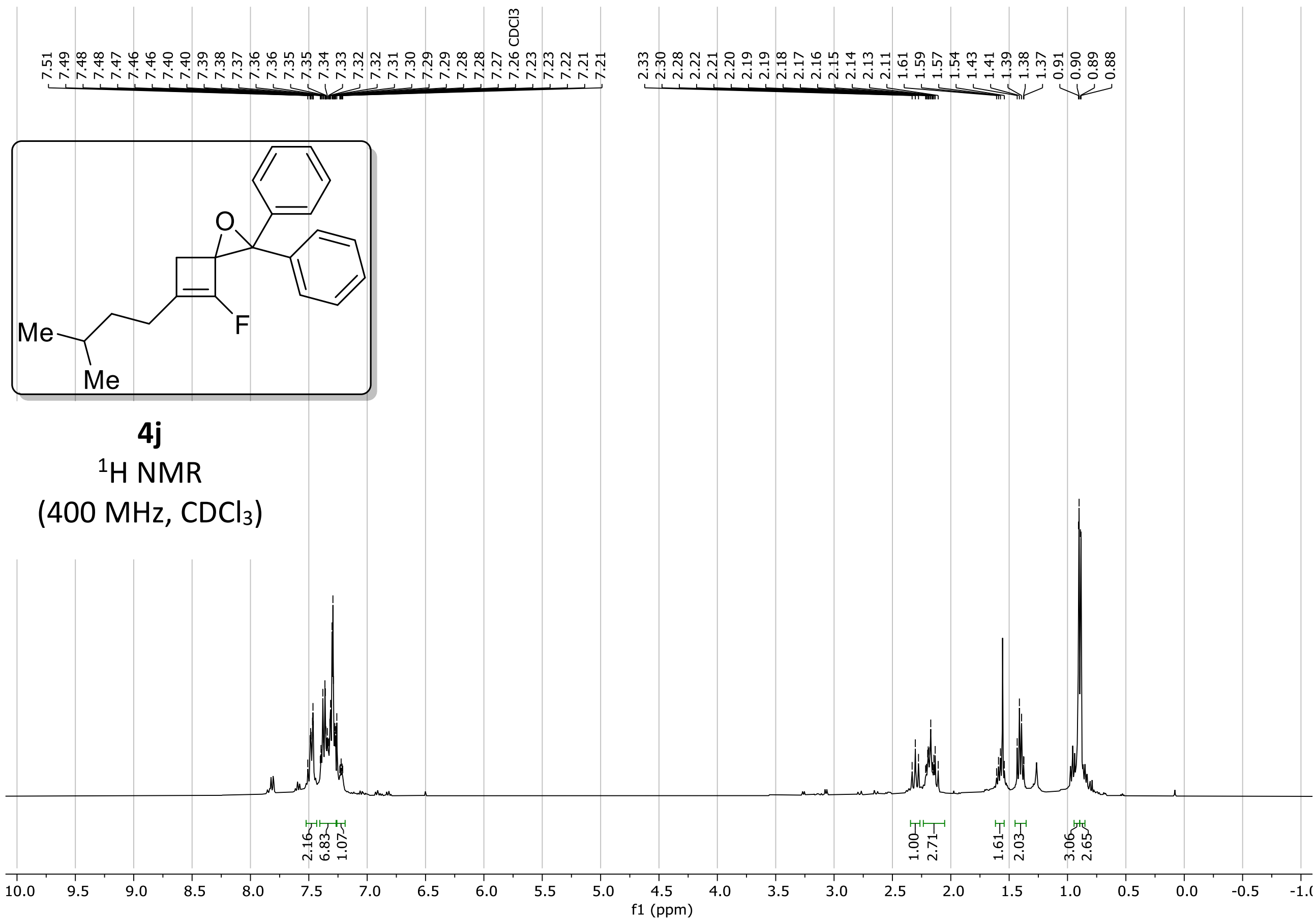
7.51 7.49 7.48 7.48 7.47 7.46 7.46 7.40 7.40 7.39 7.38 7.37 7.36 7.36 7.35 7.35 7.34 7.33 7.32 7.32 7.31 7.30 7.29 7.29 7.28 7.28 7.27 7.26 CDCl3 7.23 7.23 7.22 7.21 7.21
2.33 2.30 2.28 2.22 2.21 2.20 2.19 2.19 2.18 2.17 2.16 2.15 2.14 2.13 2.11 1.61 1.59 1.57 1.54 1.43 1.41 1.39 1.38 1.37 0.91 0.90 0.89 0.88
Me
Me
O
F
4j
^{1}H NMR
(400 MHz, CDCl$_3$)
2.16 6.83 1.07 1.00 2.71 1.61 2.03 3.06 2.65
f1 (ppm)

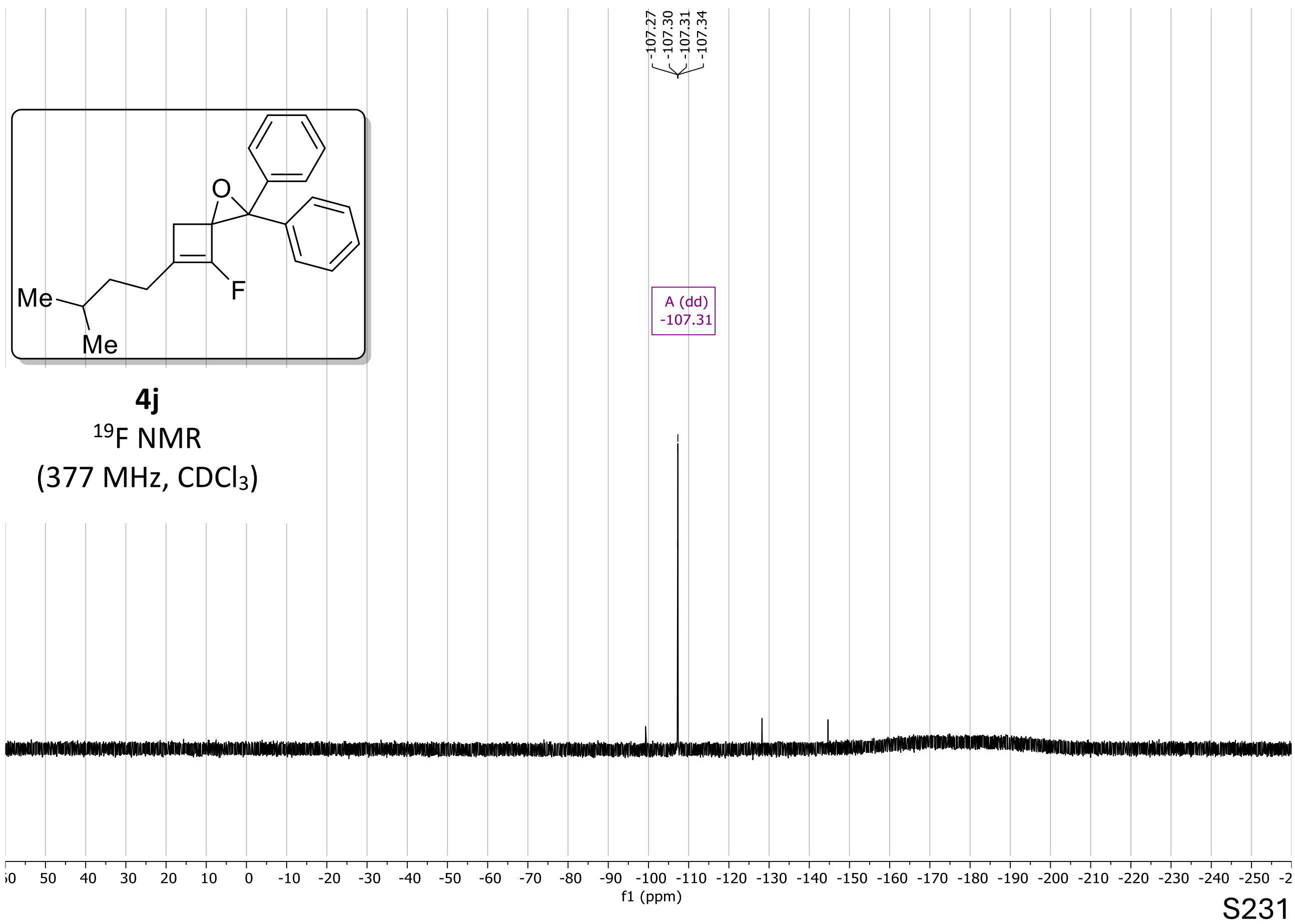
-107.27 -107.30 -107.31 -107.34
A (dd)
-107.31
Me
Me
O
F
4j
^{19}F NMR
(377 MHz, CDCl$_3$)
f1 (ppm)

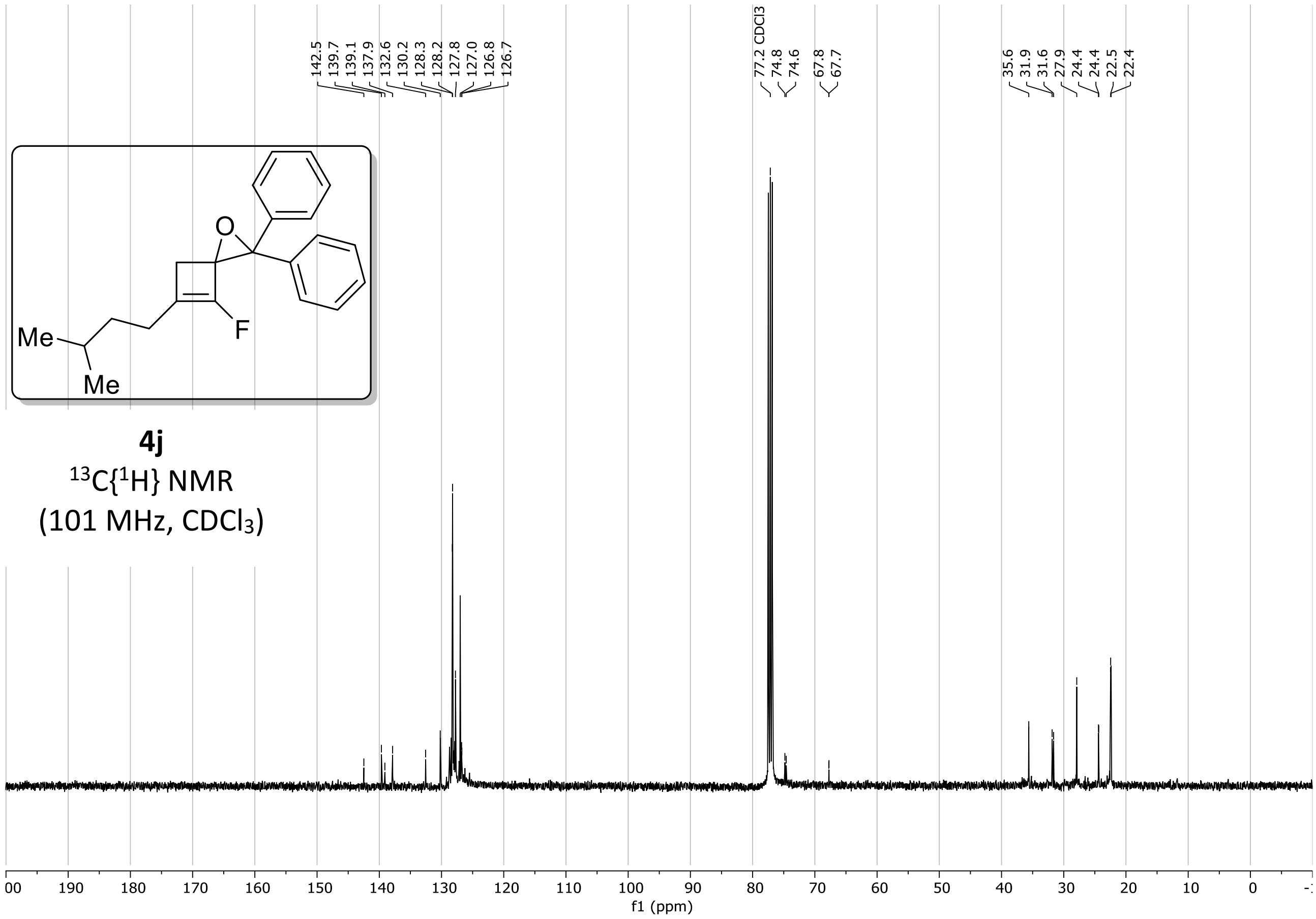

4j

$^{13}C\{^{1}H\}$ NMR

(101 MHz, $CDCl_3$)

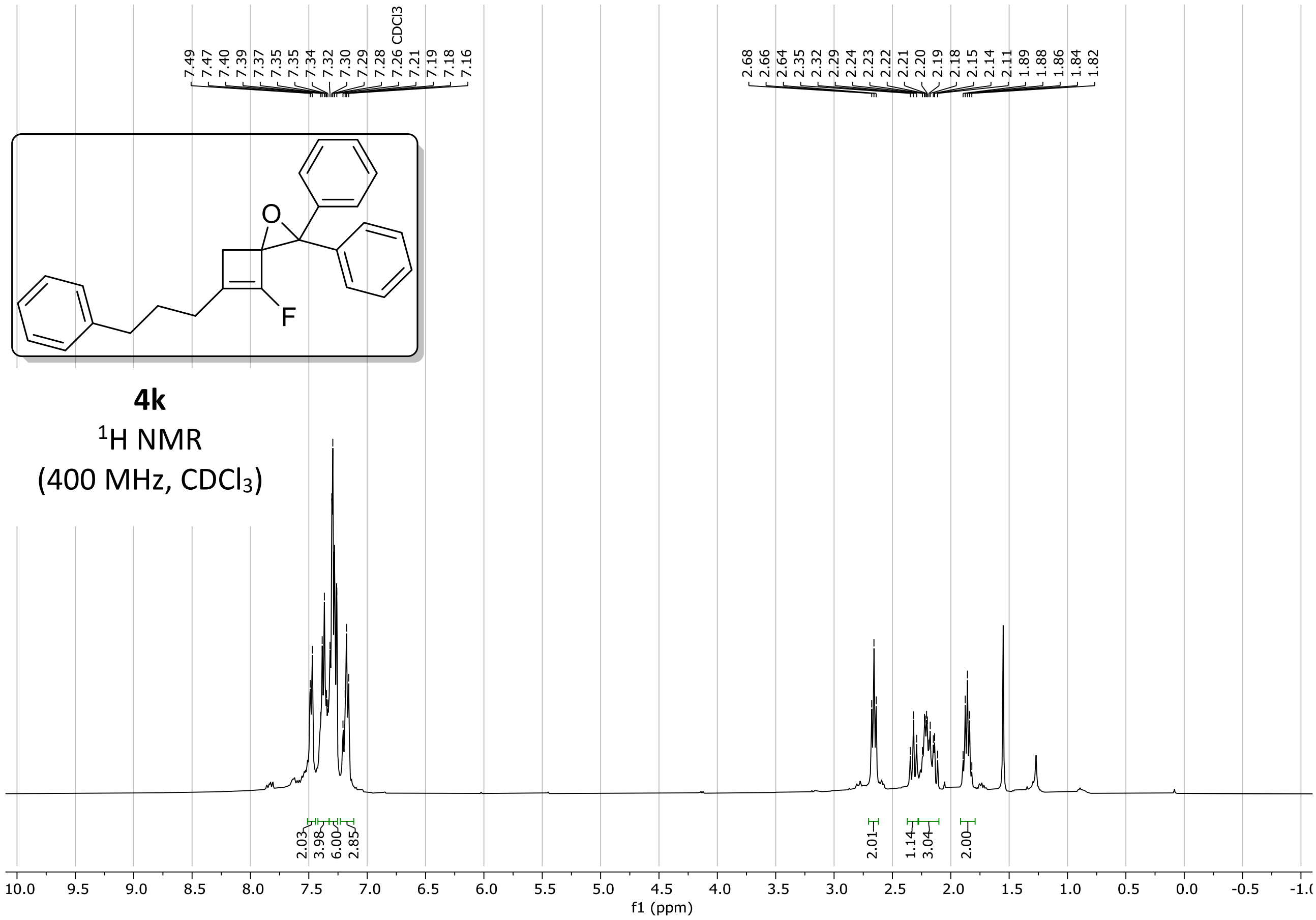

4k
^{1}H NMR
(400 MHz, $CDCl_3$)

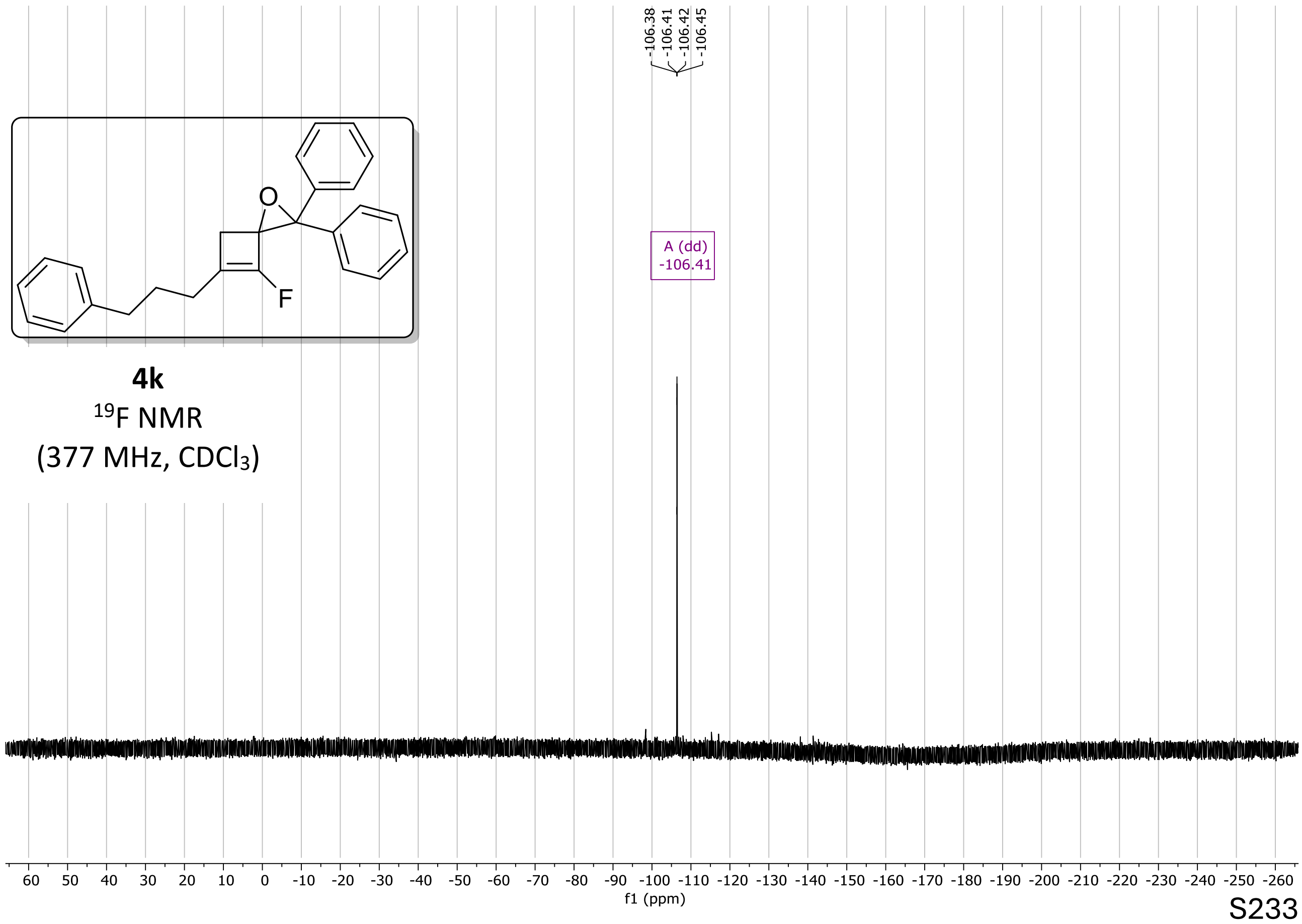

4k
^{19}F NMR
(377 MHz, $CDCl_3$)

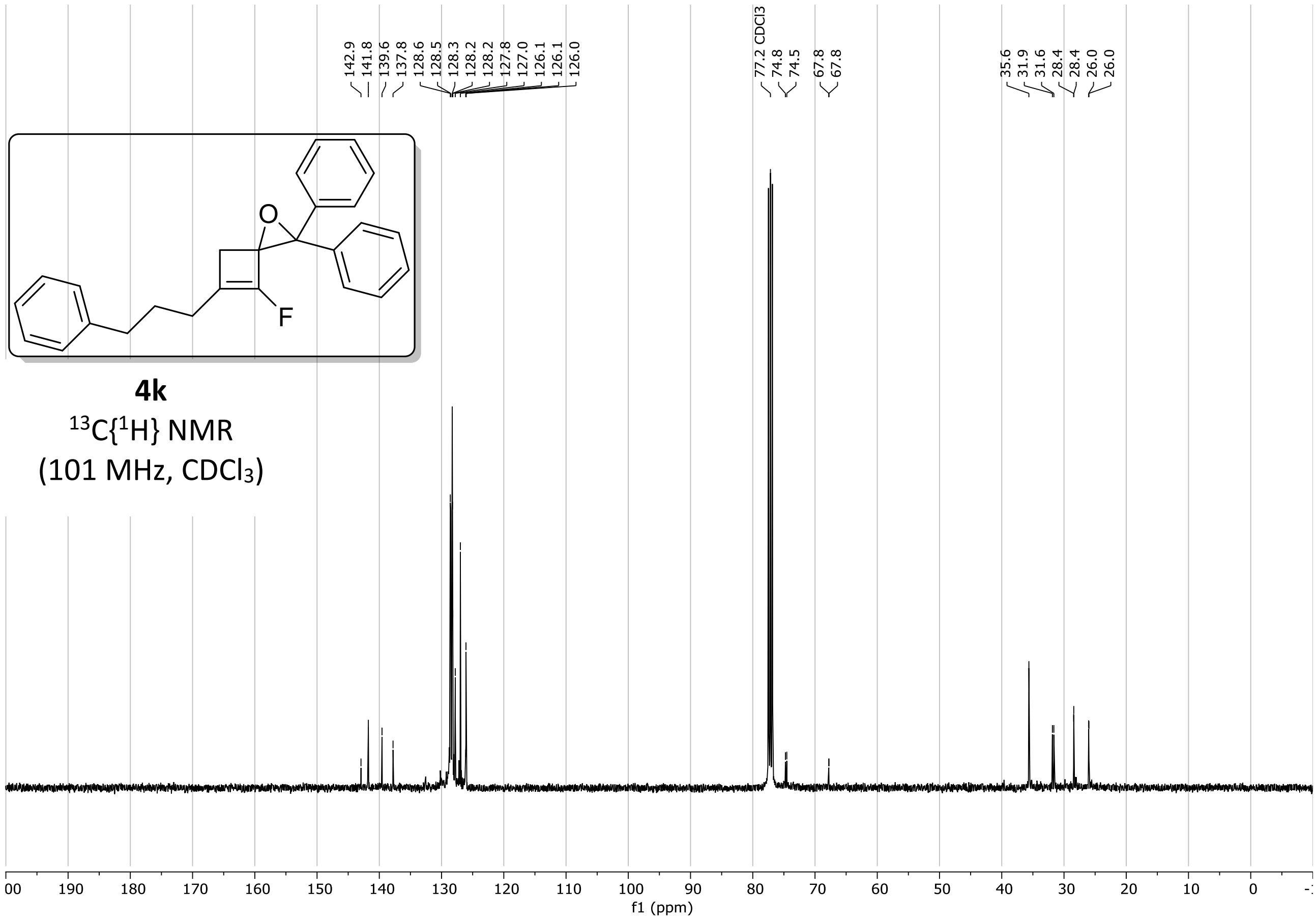
4k
13C{1H} NMR
(101 MHz, CDCl3)
142.9
141.8
139.6
137.8
128.6
128.5
128.3
128.2
128.2
127.8
127.0
126.1
126.1
126.0
77.2 CDCl3
74.8
74.5
67.8
67.8
35.6
31.9
31.6
28.4
28.4
26.0
26.0
f1 (ppm)

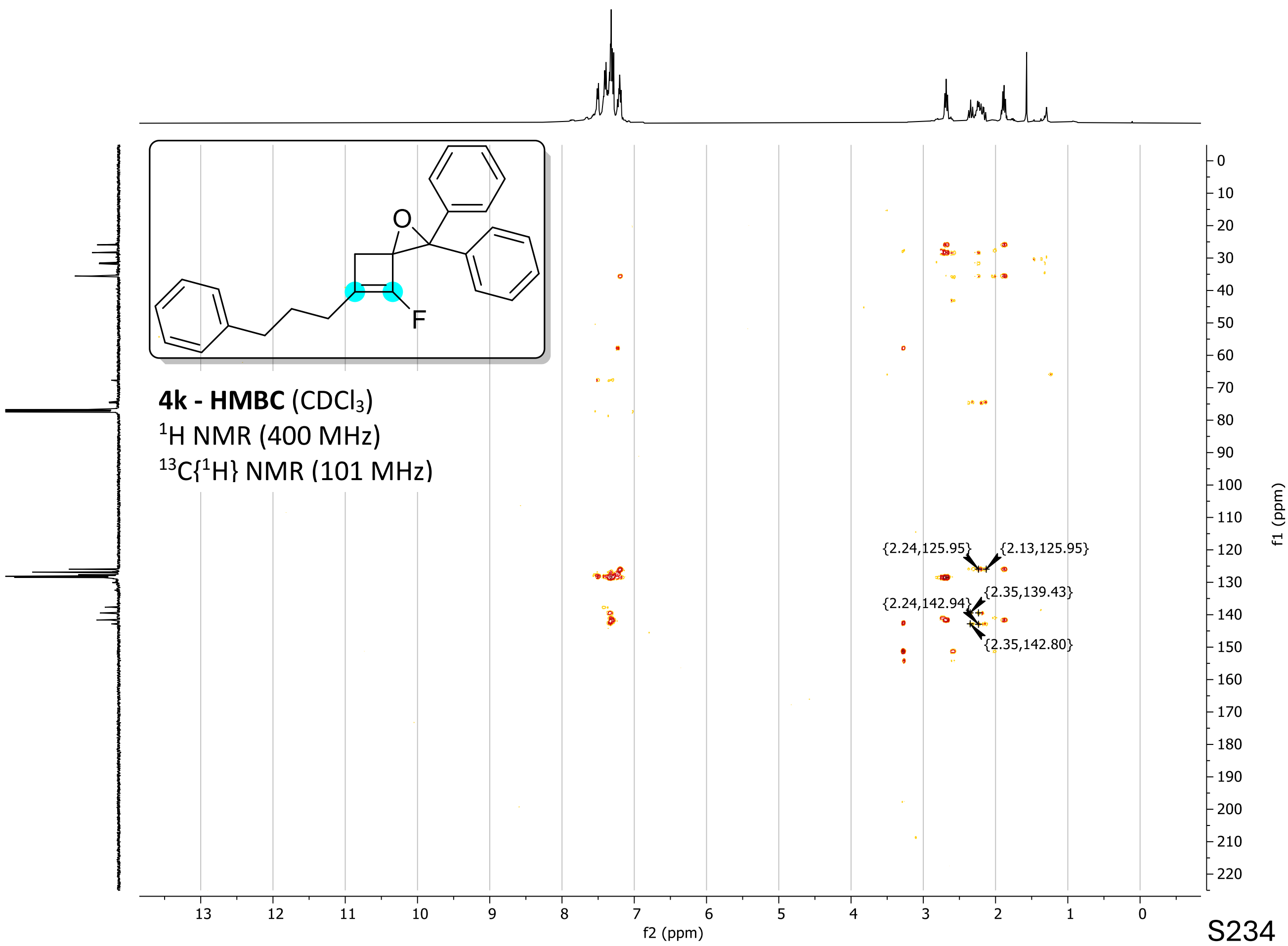
4k - HMBC (CDCl3)
1H NMR (400 MHz)
13C{1H} NMR (101 MHz)
{2.24,125.95}
{2.13,125.95}
{2.35,139.43}
{2.24,142.94}
{2.35,142.80}
f2 (ppm)
f1 (ppm)

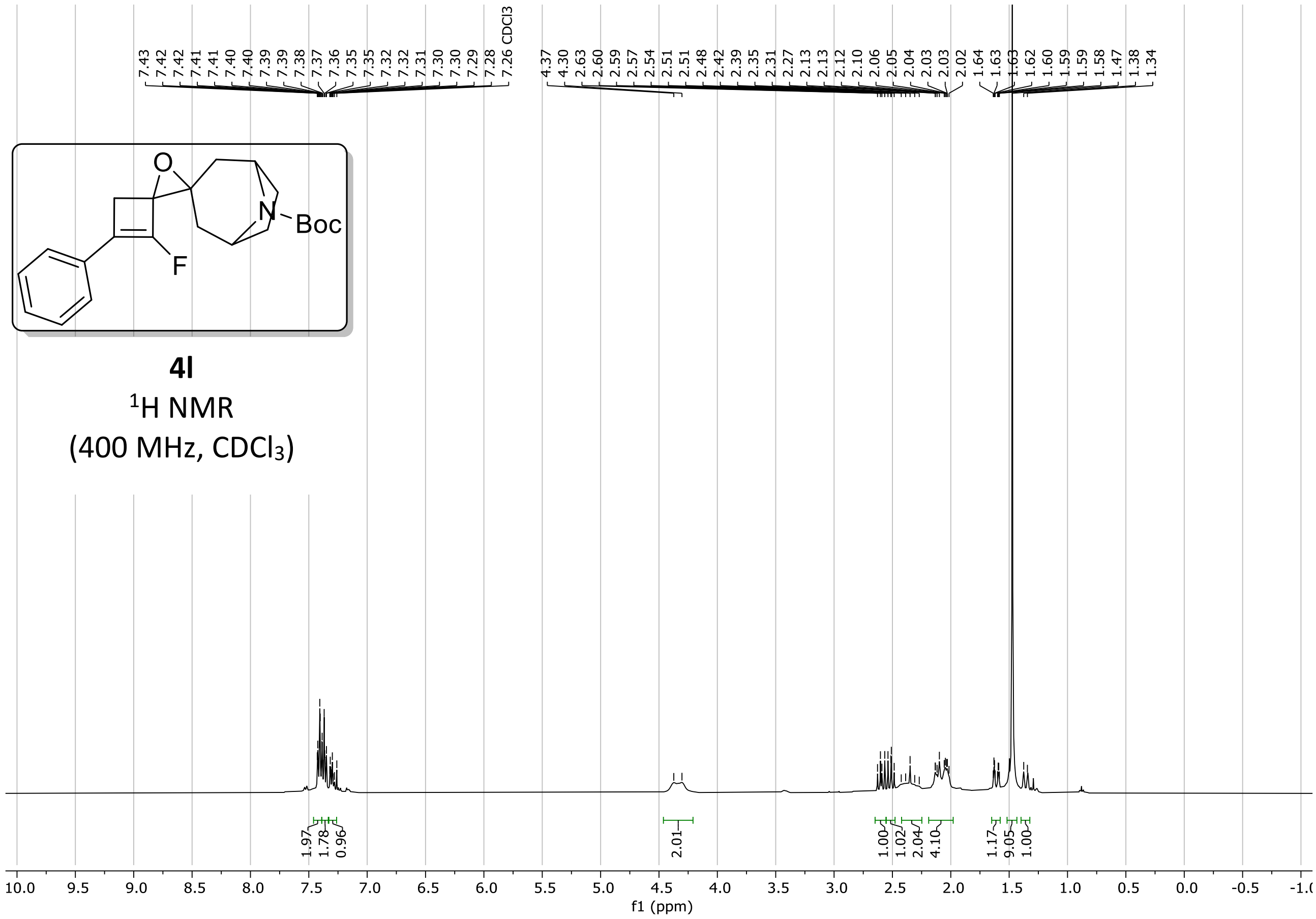

4l

^{1}H NMR

(400 MHz, $CDCl_3$)

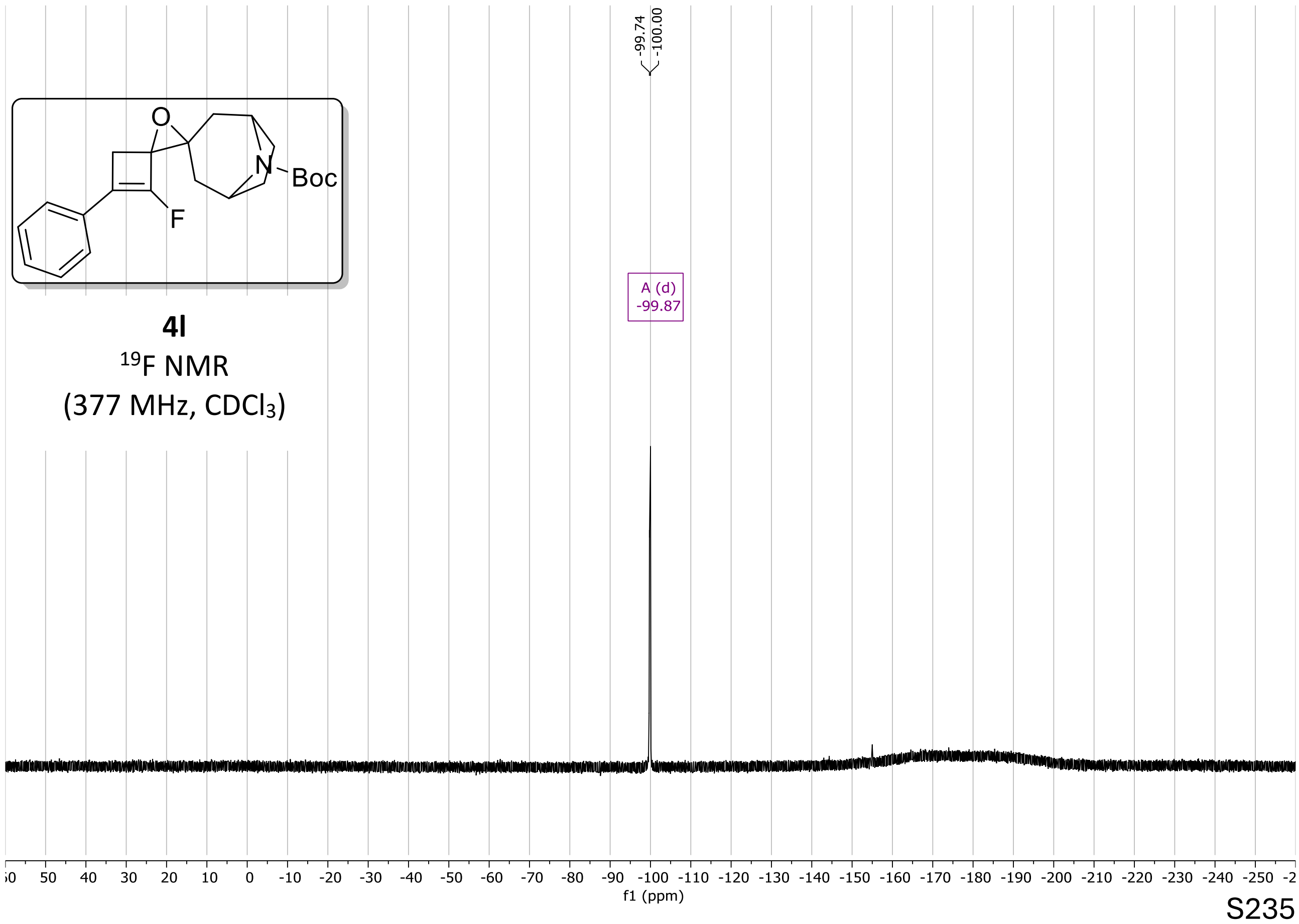

4l

^{19}F NMR

(377 MHz, $CDCl_3$)

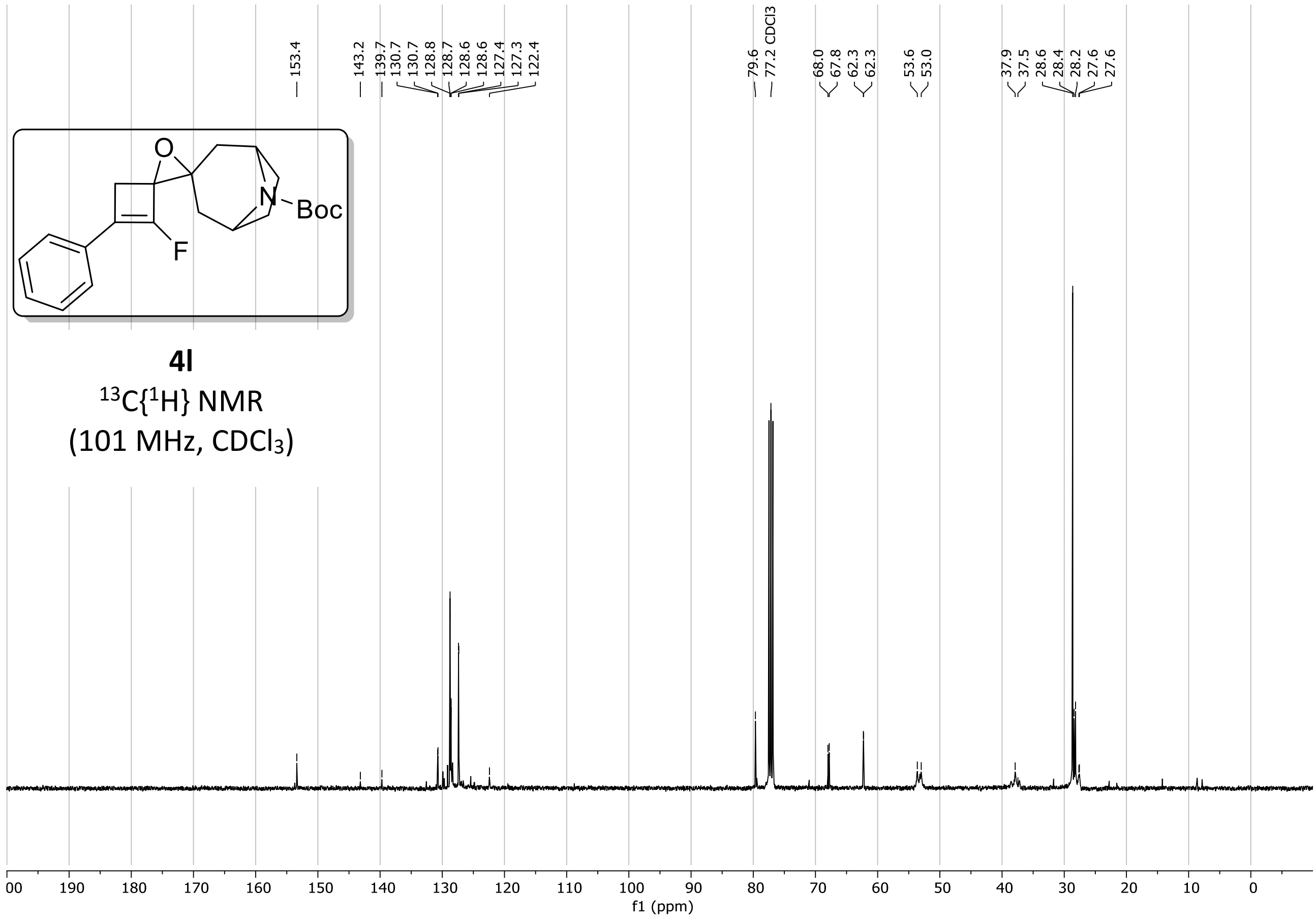

4l

$^{13}C\{^{1}H\}$ NMR

(101 MHz, $CDCl_3$)

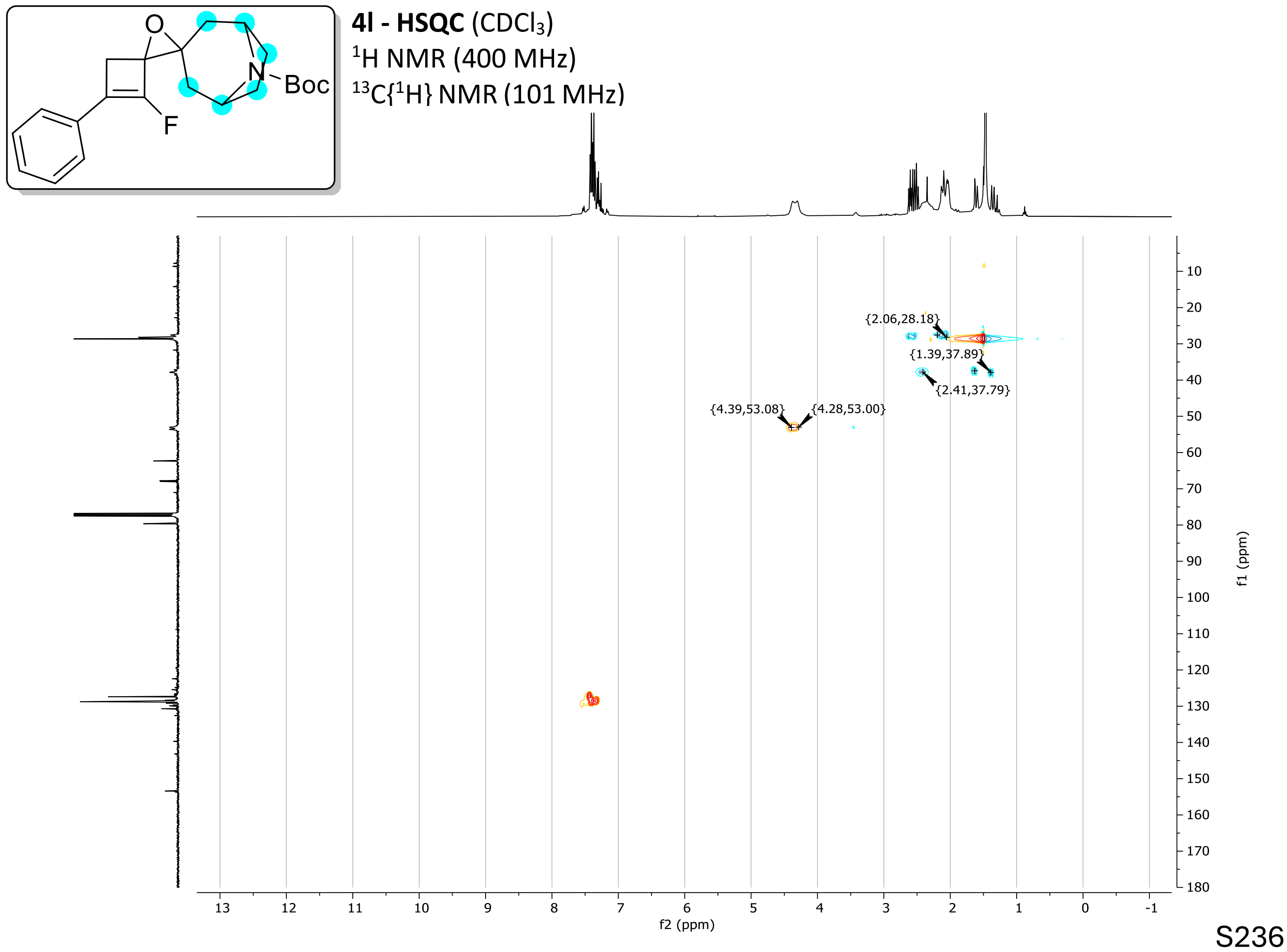

4l - HSQC ($CDCl_3$)

^{1}H NMR (400 MHz)

$^{13}C\{^{1}H\}$ NMR (101 MHz)

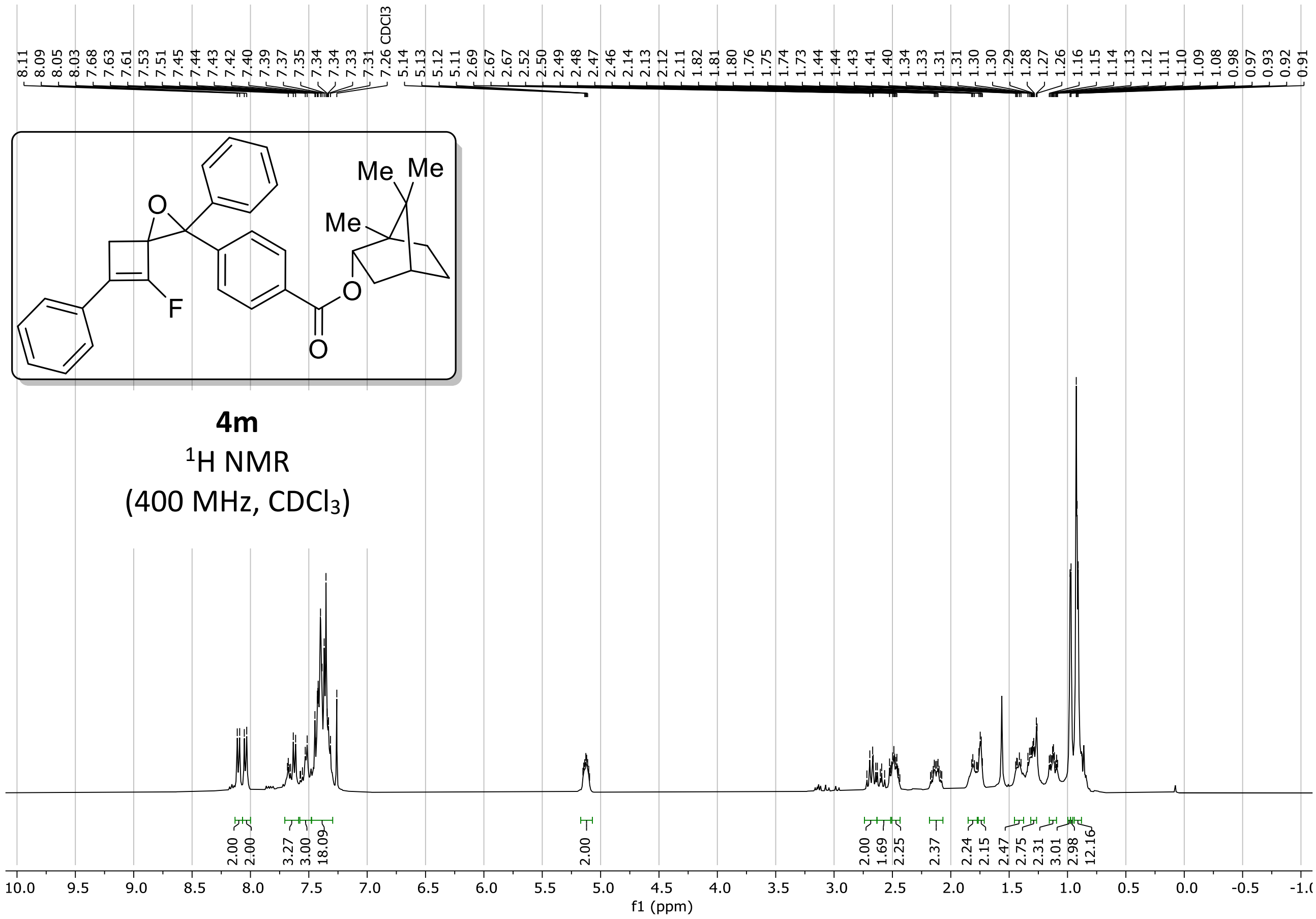
8.11
8.09
8.05
8.03
7.68
7.63
7.61
7.53
7.51
7.45
7.44
7.43
7.42
7.40
7.39
7.37
7.35
7.34
7.34
7.33
7.31
7.26 CDCl3
5.14
5.13
5.12
5.11
2.69
2.67
2.67
2.52
2.50
2.49
2.48
2.47
2.46
2.14
2.13
2.12
2.11
1.82
1.81
1.80
1.76
1.75
1.74
1.73
1.44
1.44
1.43
1.41
1.40
1.34
1.33
1.31
1.31
1.30
1.29
1.28
1.27
1.26
1.16
1.15
1.14
1.13
1.12
1.11
1.10
1.09
1.08
0.98
0.97
0.93
0.92
0.91
Me
Me
Me
O
F
O
O
4m
^{1}H NMR
(400 MHz, $CDCl_3$)
2.00
2.00
3.27
3.00
18.09
2.00
2.00
1.69
2.25
2.37
2.24
2.15
2.47
2.75
2.31
3.01
2.98
12.16
10.0 9.5 9.0 8.5 8.0 7.5 7.0 6.5 6.0 5.5 5.0 4.5 4.0 3.5 3.0 2.5 2.0 1.5 1.0 0.5 0.0 -0.5
f1 (ppm)

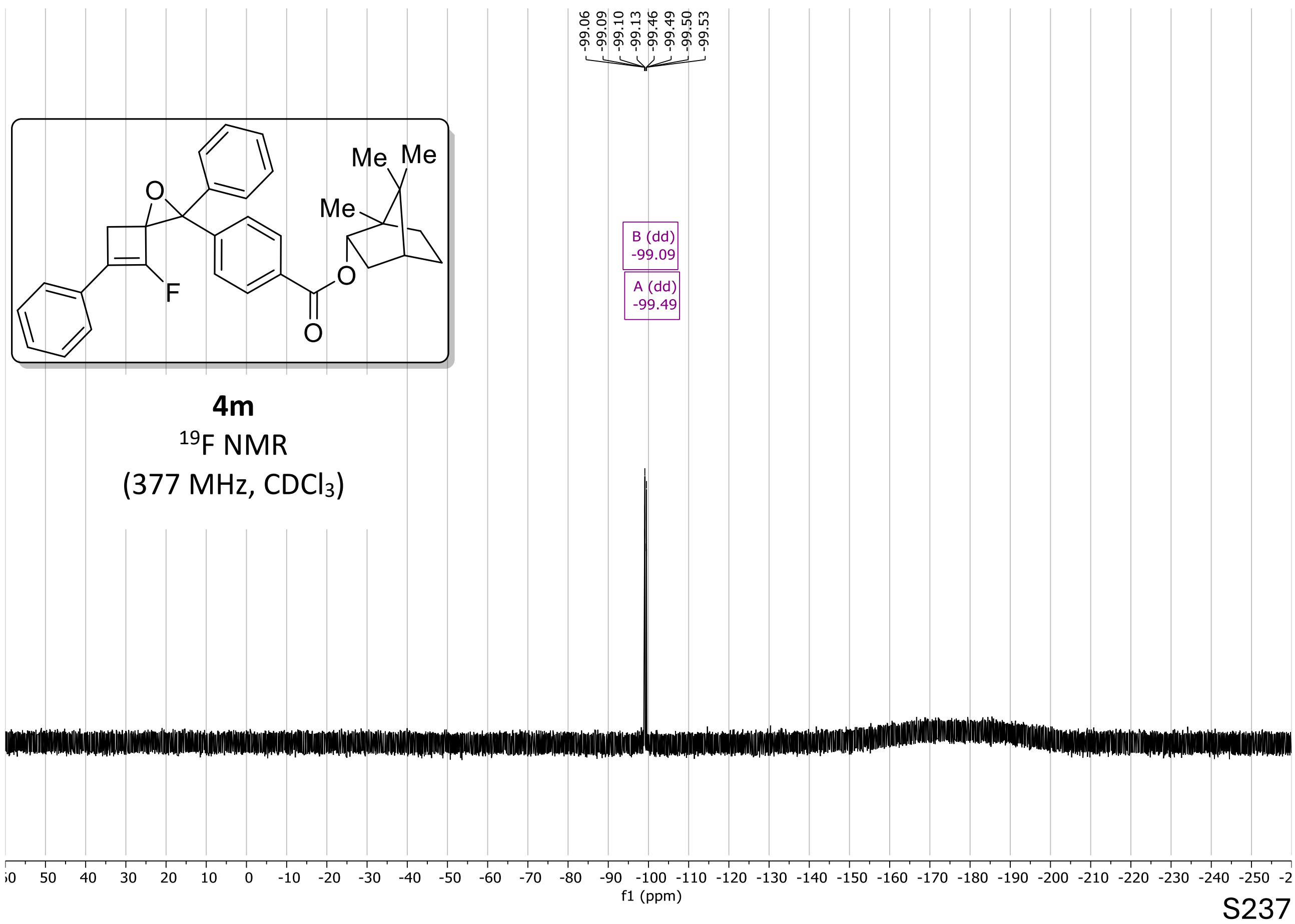
-99.06
-99.09
-99.10
-99.13
-99.46
-99.49
-99.50
-99.53
B (dd)
-99.09
A (dd)
-99.49
Me
Me
Me
O
F
O
O
4m
^{19}F NMR
(377 MHz, $CDCl_3$)
50 40 30 20 10 0 -10 -20 -30 -40 -50 -60 -70 -80 -90 -100 -110 -120 -130 -140 -150 -160 -170 -180 -190 -200 -210 -220 -230 -240 -250
f1 (ppm)

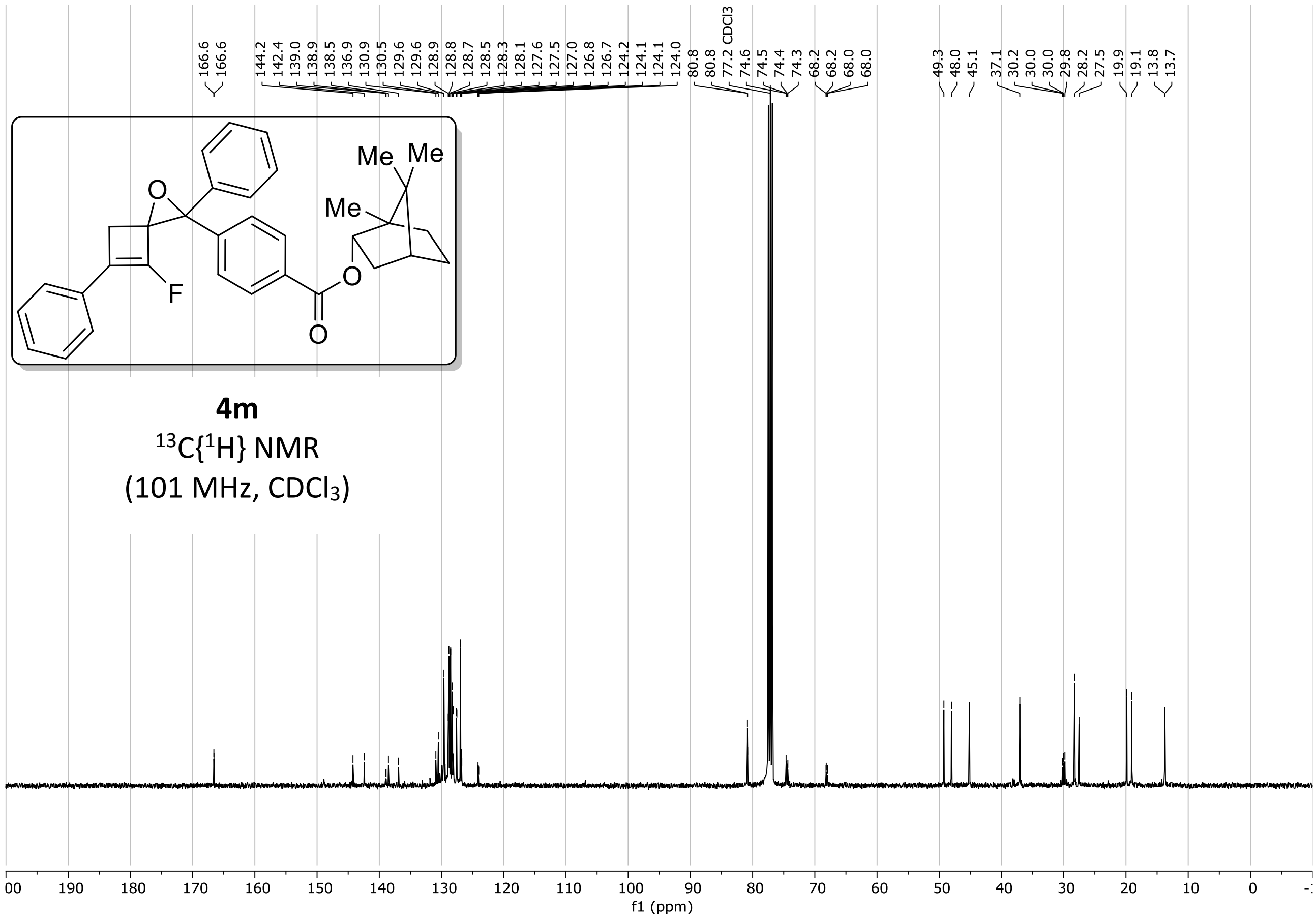

4m

$^{13}C\{^{1}H\}$ NMR

(101 MHz, $CDCl_3$)

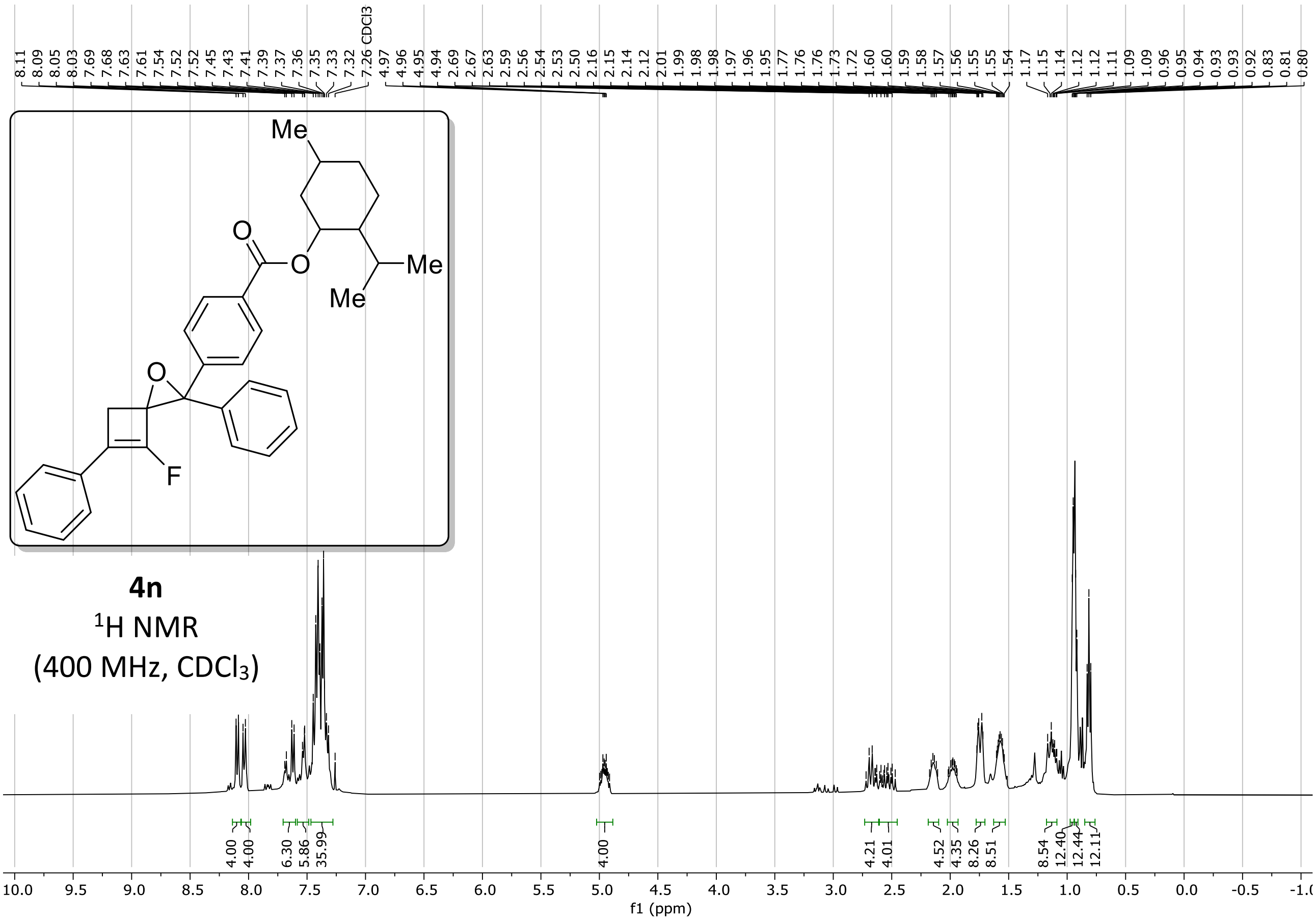
4n
^{1}H NMR
(400 MHz, $CDCl_3$)
Me
O
Me
F
f1 (ppm)

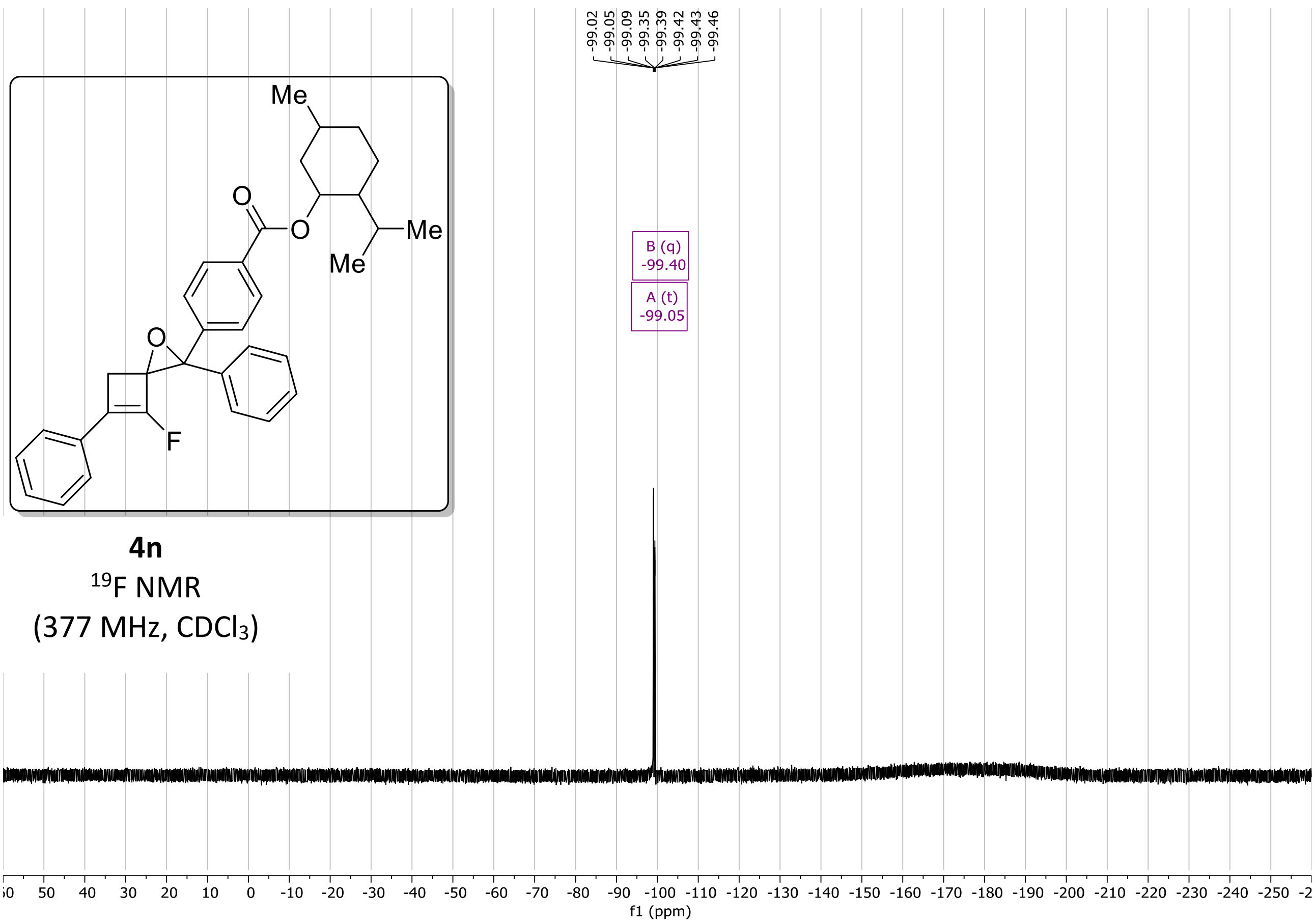
4n
^{19}F NMR
(377 MHz, $CDCl_3$)
B (q)
-99.40
A (t)
-99.05
f1 (ppm)

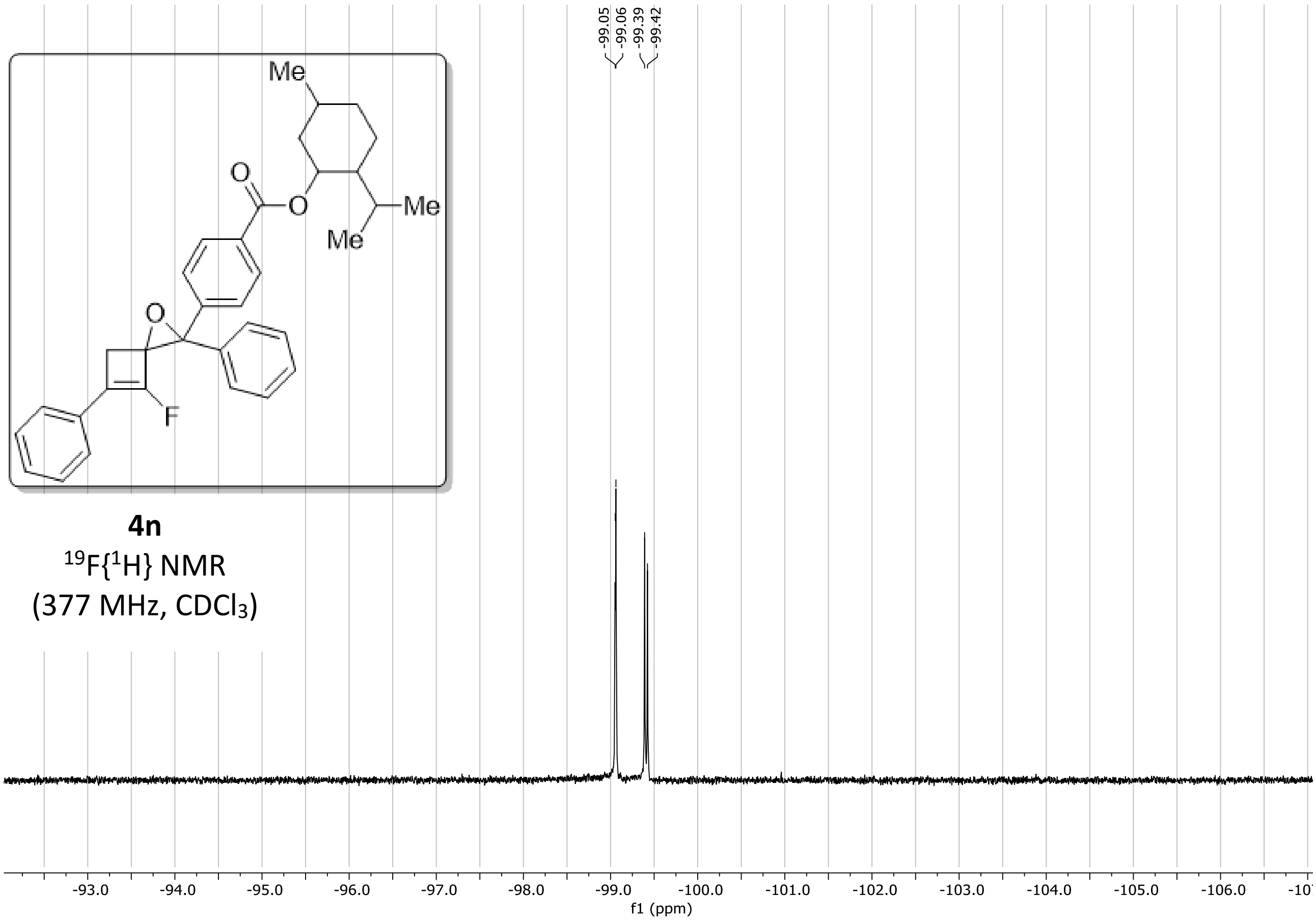

4n
$^{19}F\{^1H\}$ NMR
(377 MHz, $CDCl_3$)

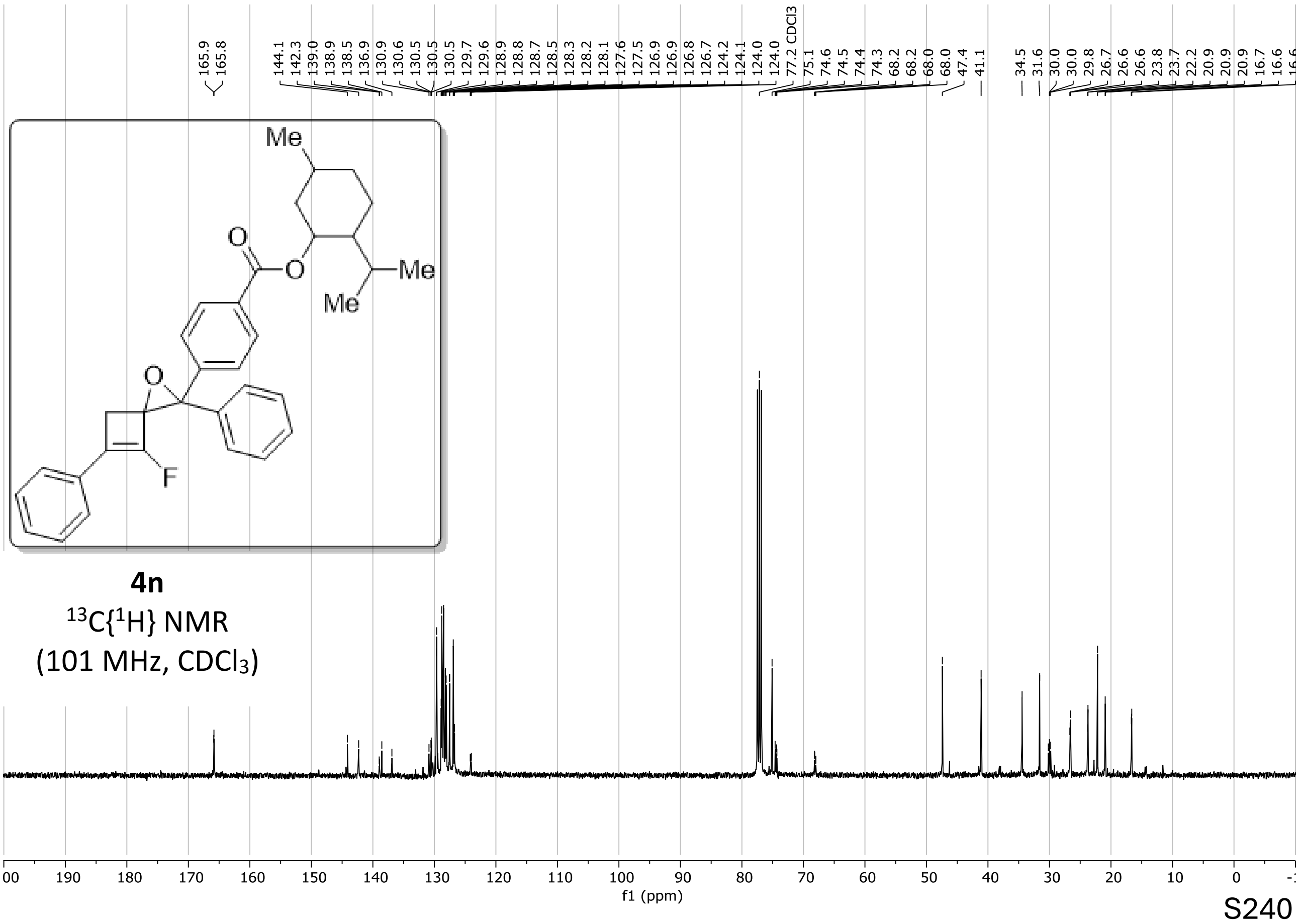

4n
$^{13}C\{^1H\}$ NMR
(101 MHz, $CDCl_3$)

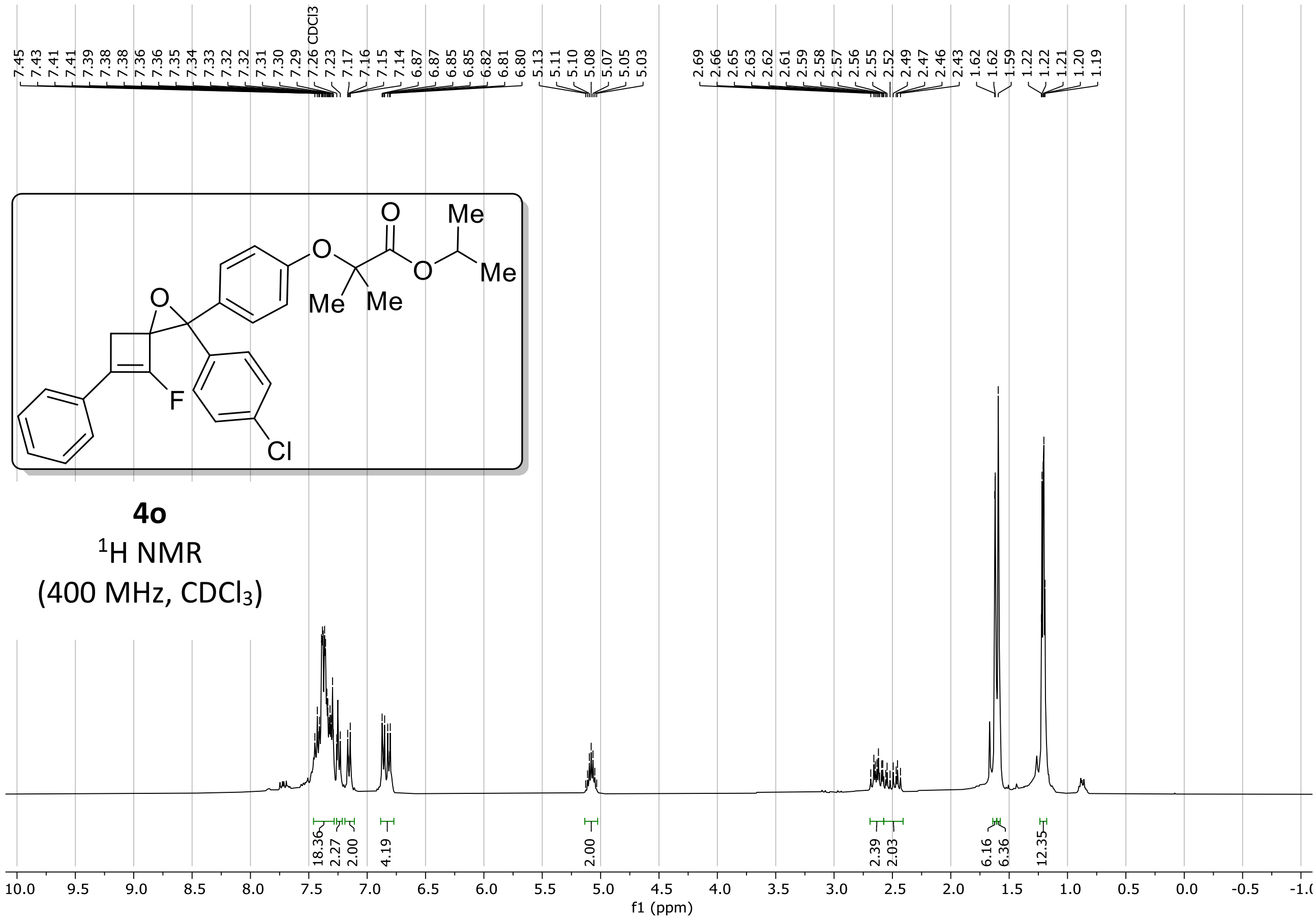
7.45 7.43 7.41 7.39 7.38 7.38 7.36 7.36 7.35 7.34 7.33 7.32 7.32 7.31 7.30 7.29 7.26 CDCl3 7.23 7.17 7.16 7.15 7.14 6.87 6.87 6.85 6.85 6.82 6.81 6.80 5.13 5.11 5.10 5.08 5.07 5.05 5.03 2.69 2.66 2.65 2.63 2.62 2.61 2.59 2.58 2.57 2.56 2.55 2.52 2.49 2.47 2.46 2.43 1.62 1.62 1.59 1.22 1.22 1.21 1.20 1.19
O
Me
O
O
Me
Me Me
O
F
Cl
4o
^{1}H NMR
(400 MHz, CDCl$_3$)
18.36 2.27 2.00 4.19 2.00 2.39 2.03 6.16 6.36 12.35
f1 (ppm)

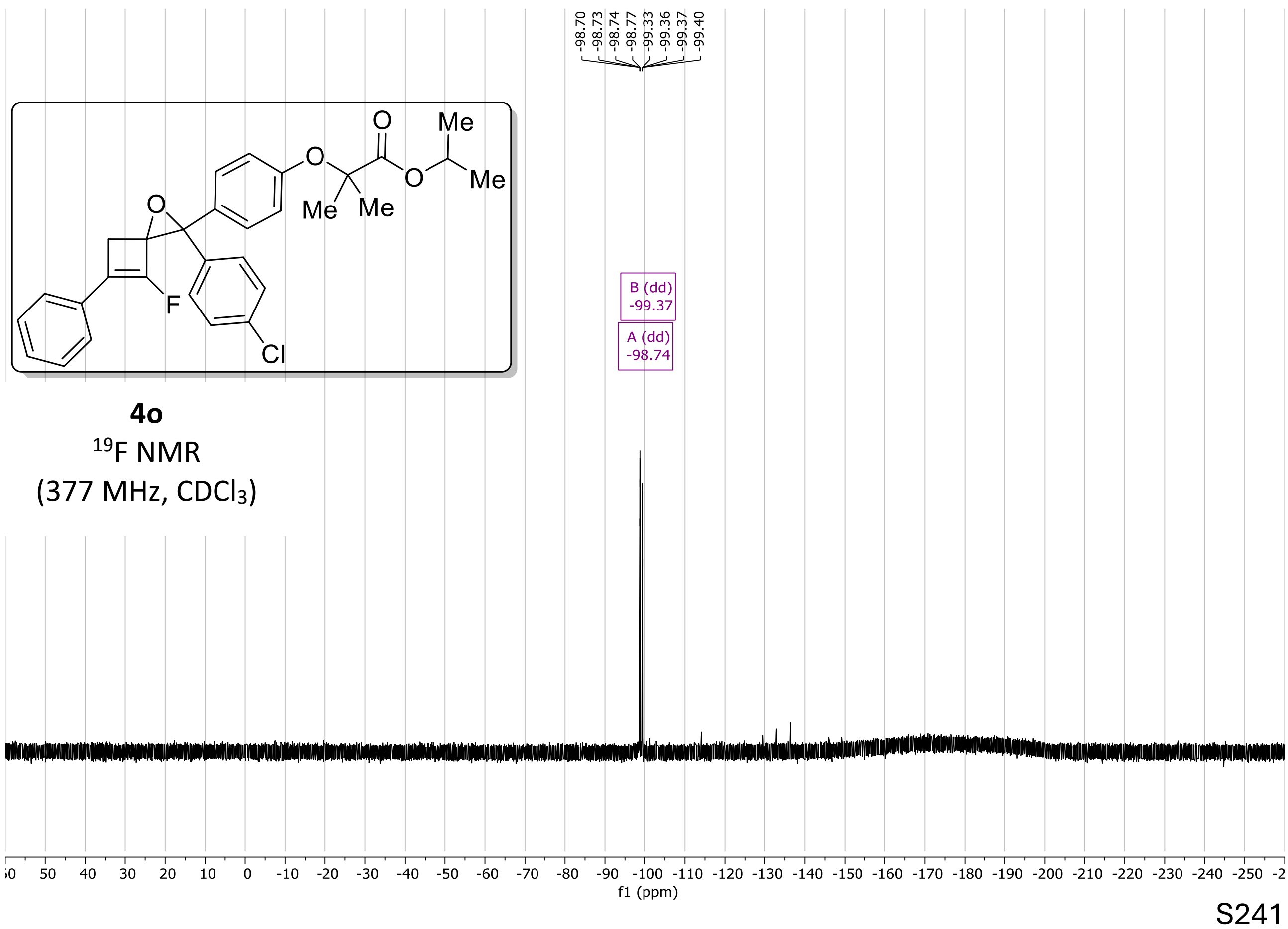
-98.70 -98.73 -98.74 -98.77 -99.33 -99.36 -99.37 -99.40
O
Me
O
O
Me
Me Me
O
F
Cl
B (dd)
-99.37
A (dd)
-98.74
4o
^{19}F NMR
(377 MHz, CDCl$_3$)
f1 (ppm)

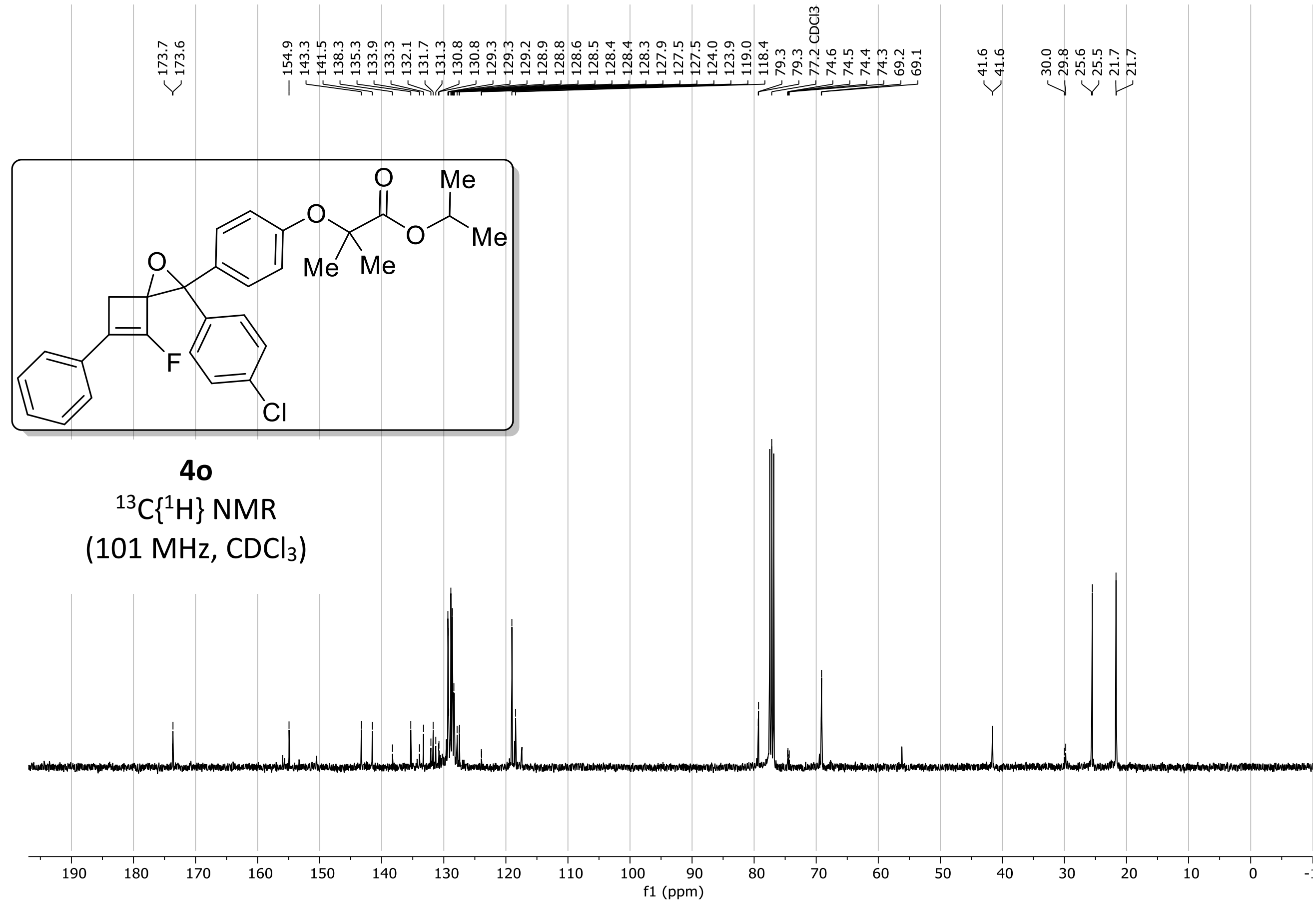

4o

$^{13}C\{^{1}H\}$ NMR

(101 MHz, $CDCl_3$)

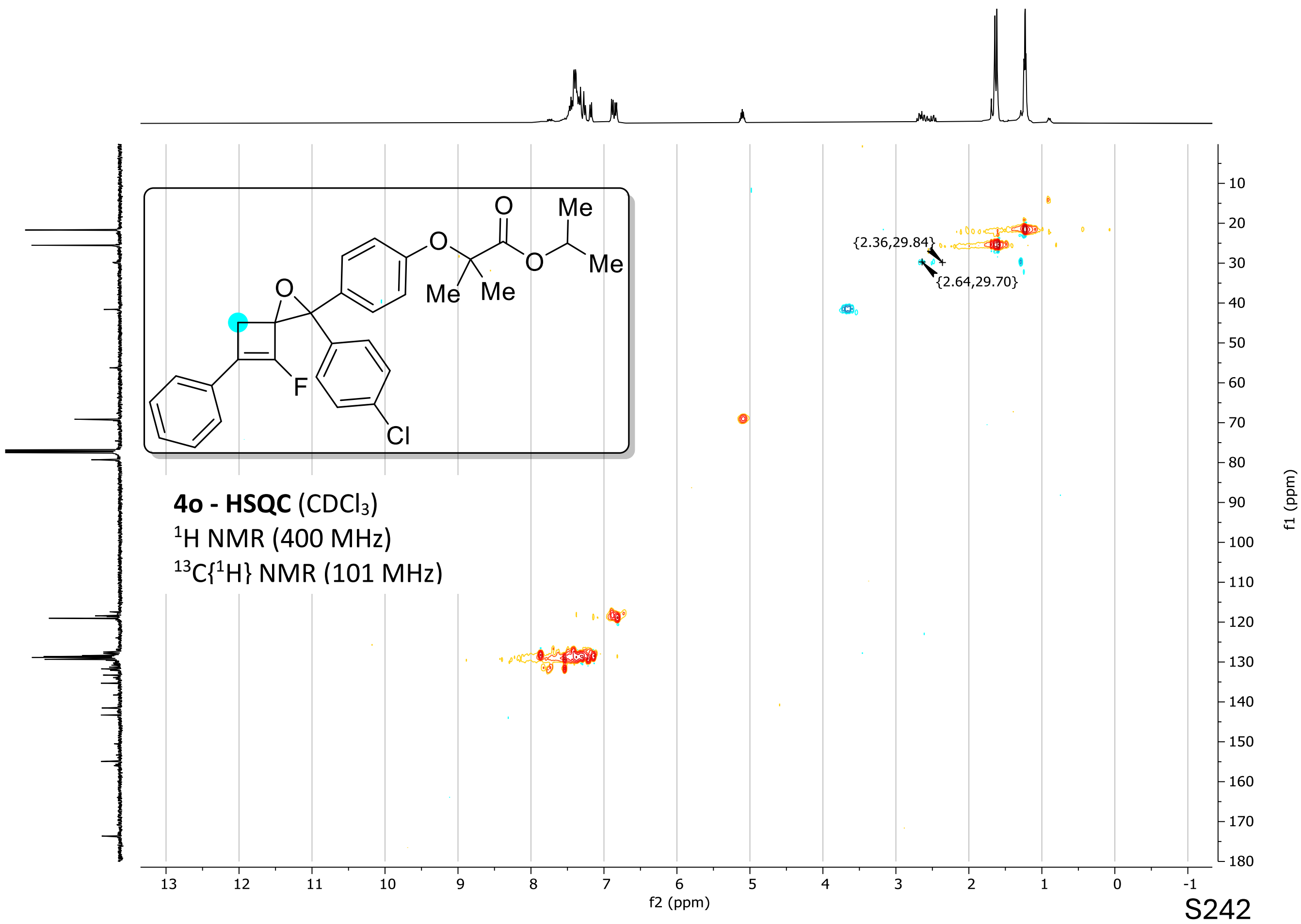

4o - HSQC ($CDCl_3$)

^{1}H NMR (400 MHz)

$^{13}C\{^{1}H\}$ NMR (101 MHz)

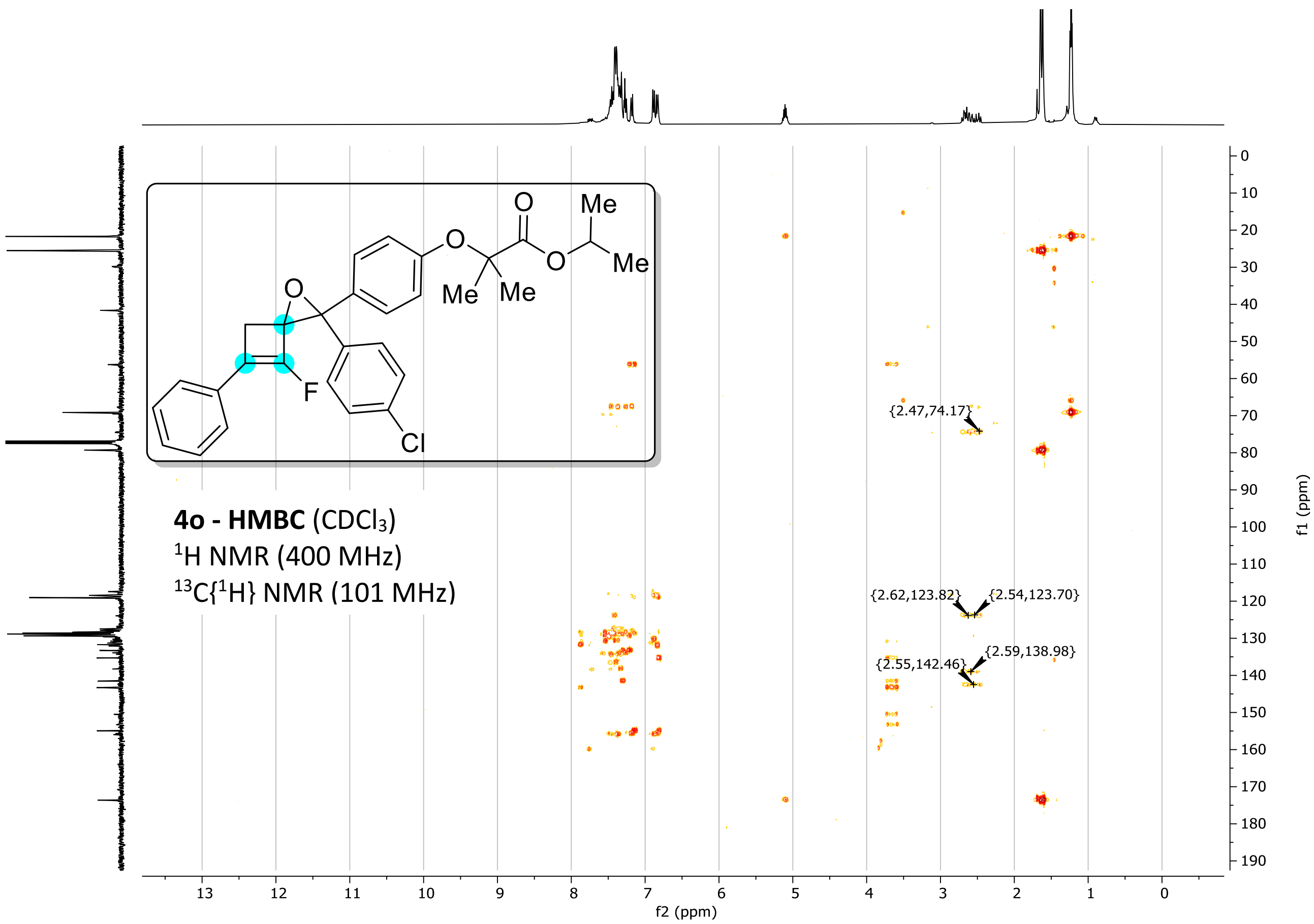

O
Me
O
O
Me
Me
Me
O
F
Cl
4o - HMBC (CDCl3)
1H NMR (400 MHz)
13C{1H} NMR (101 MHz)
{2.47,74.17}
{2.62,123.82}
{2.54,123.70}
{2.59,138.98}
{2.55,142.46}
f2 (ppm)
f1 (ppm)

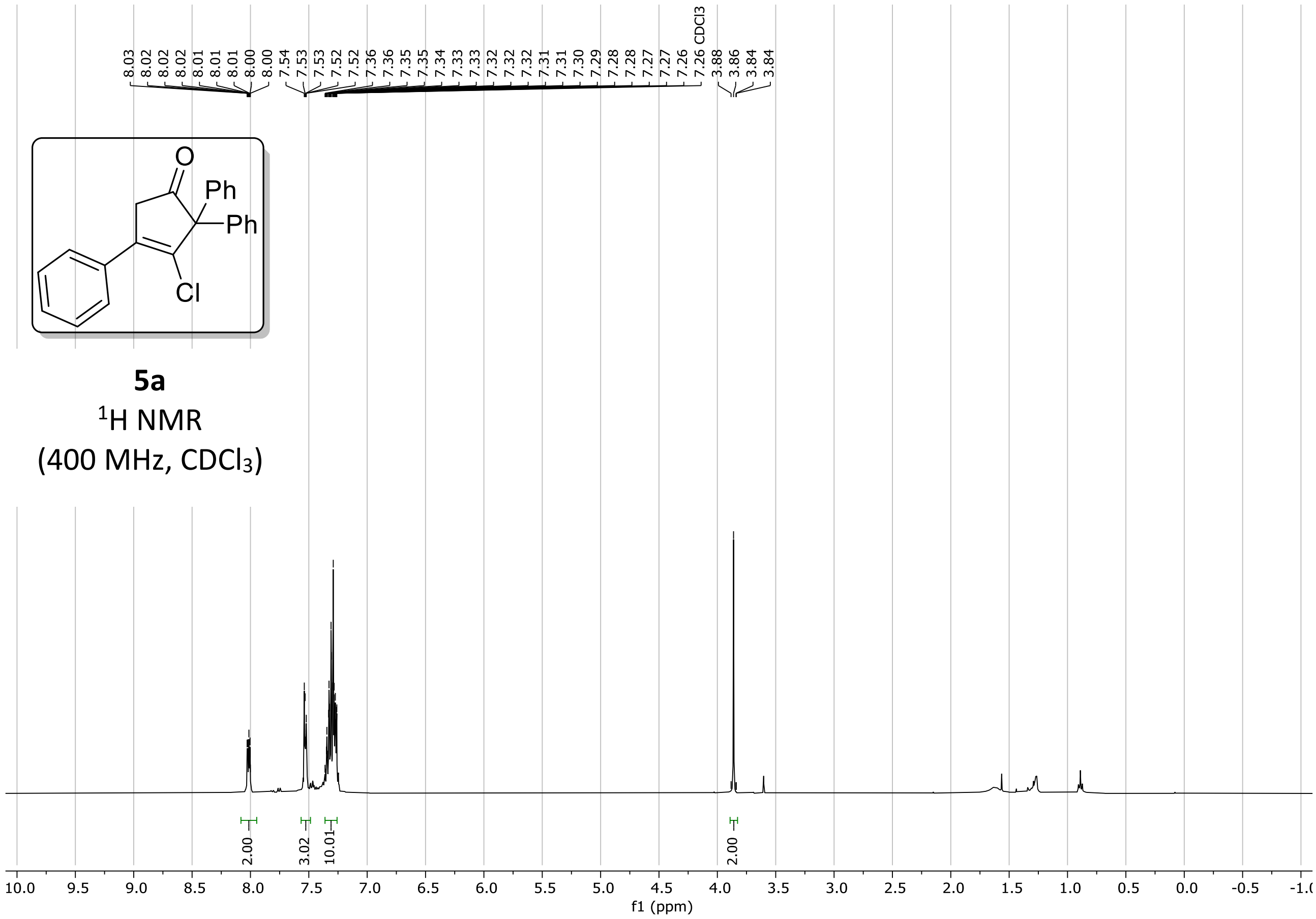

5a
^{1}H NMR
(400 MHz, $CDCl_3$)

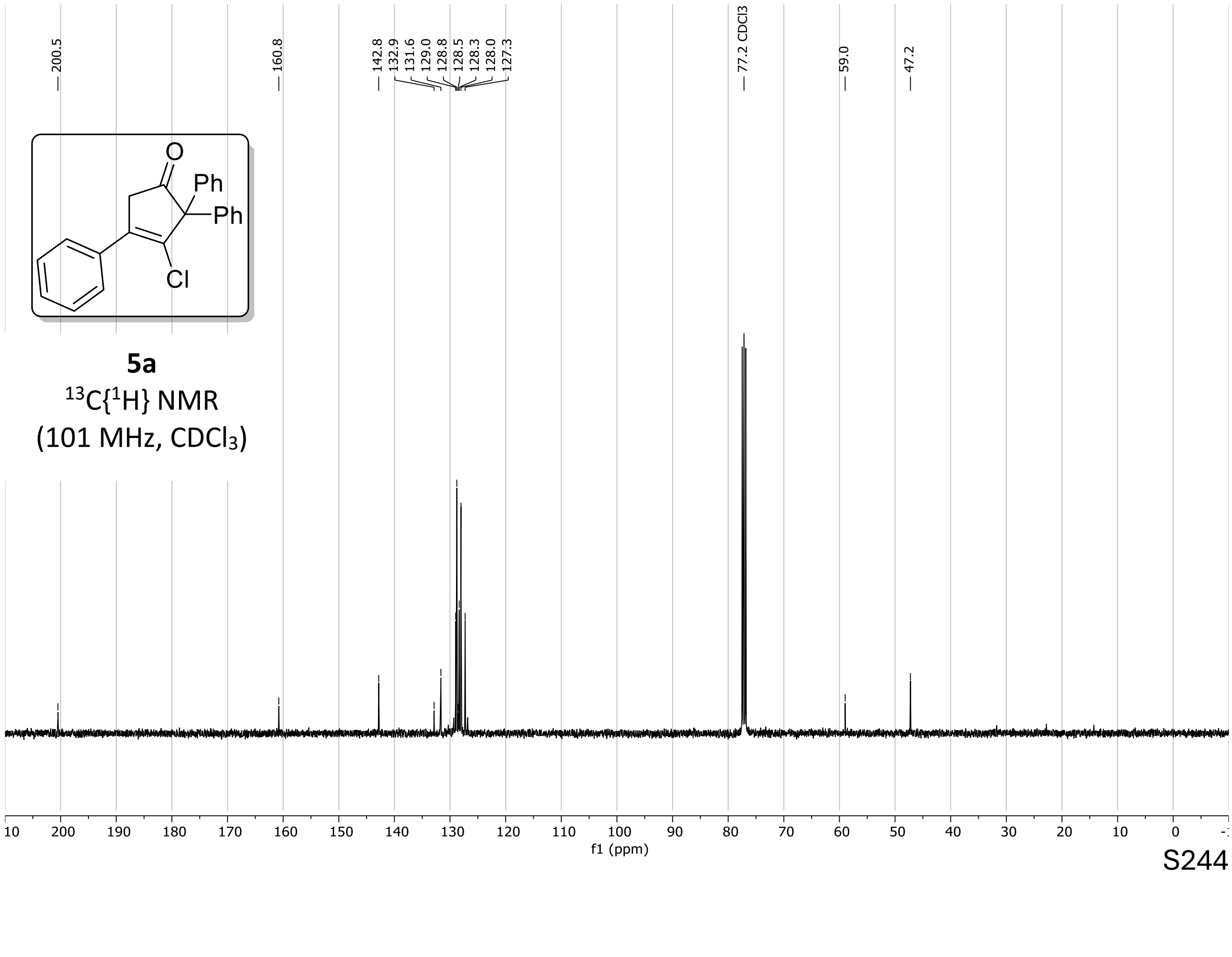

5a
$^{13}C\{^{1}H\}$ NMR
(101 MHz, $CDCl_3$)

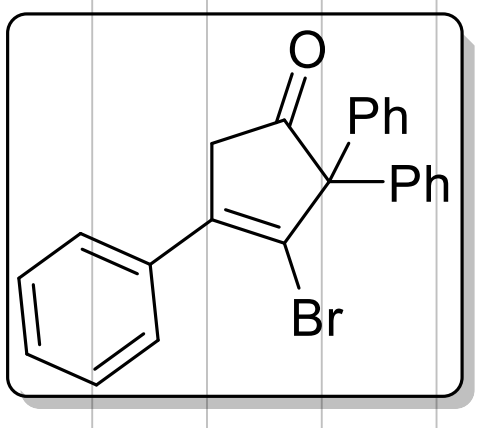

5b
^{1}H NMR
(400 MHz, $CDCl_3$)

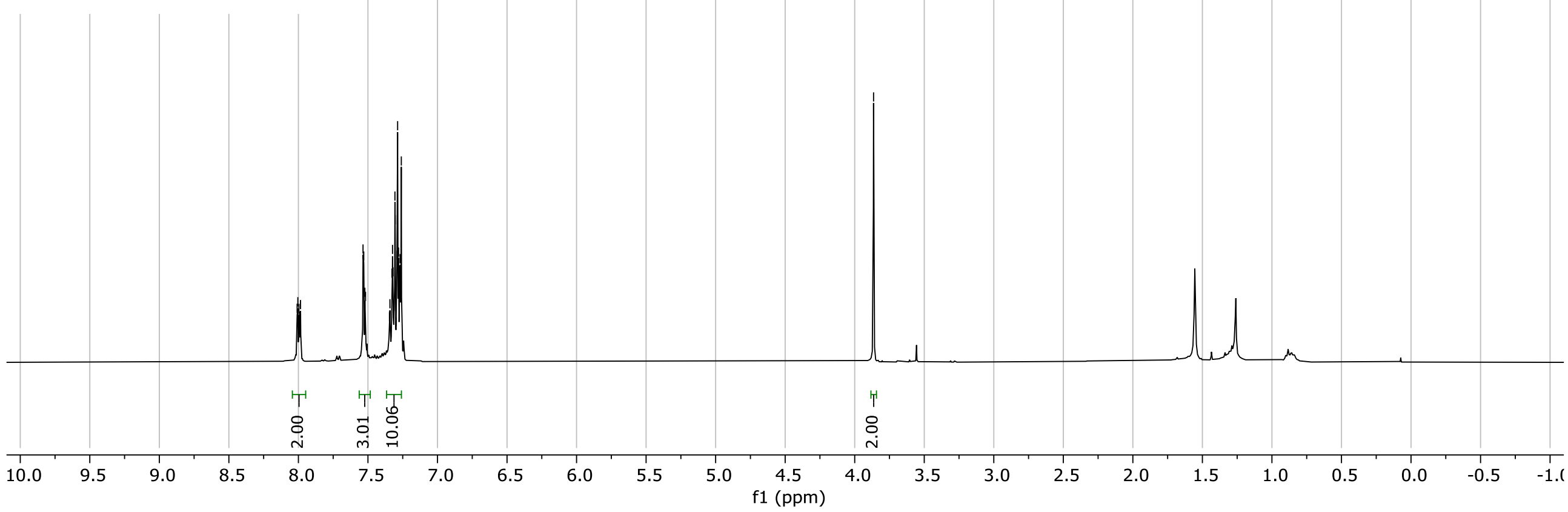

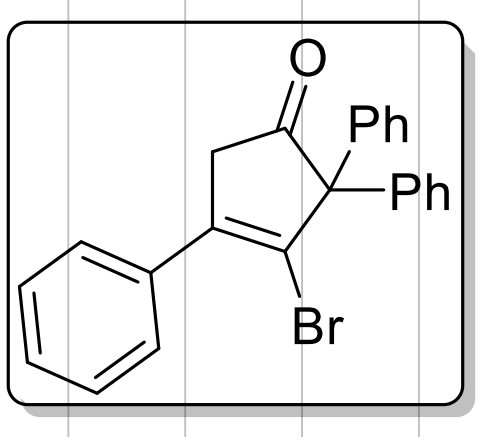

5b
^{13}C{^{1}H} NMR
(101 MHz, $CDCl_3$)

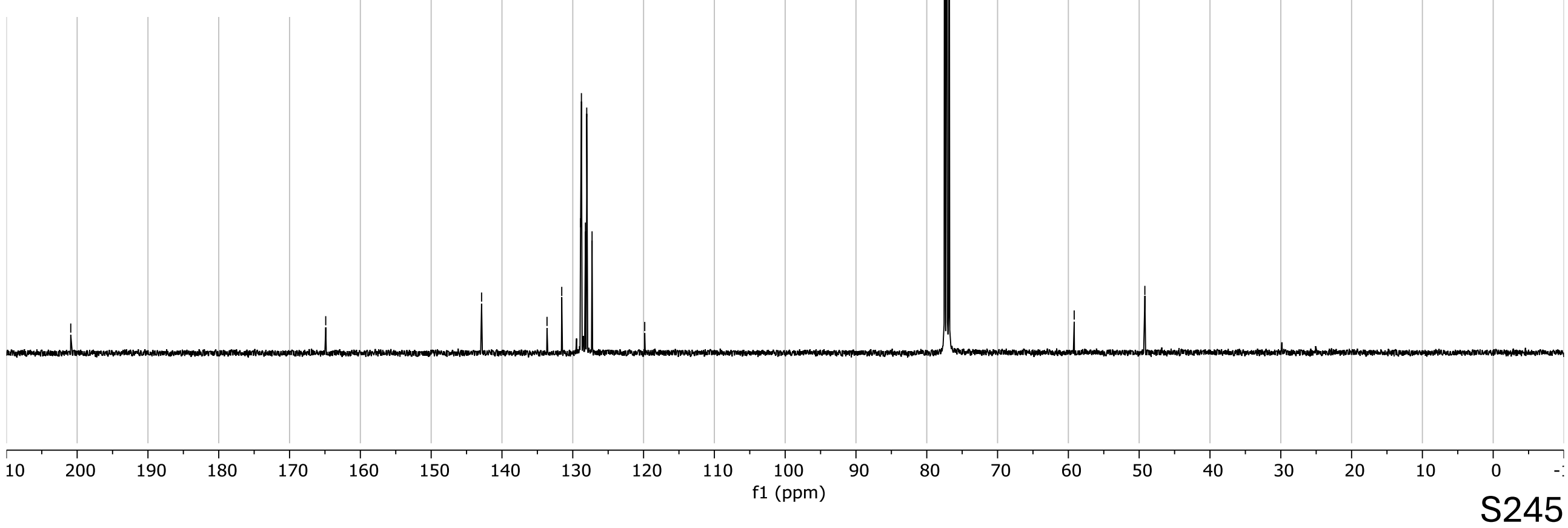

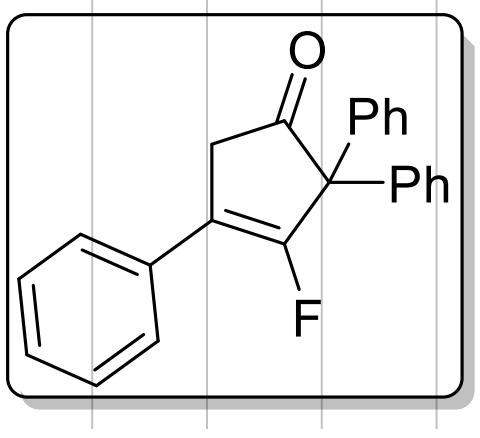

5c
^{1}H NMR
(400 MHz, $CDCl_3$)

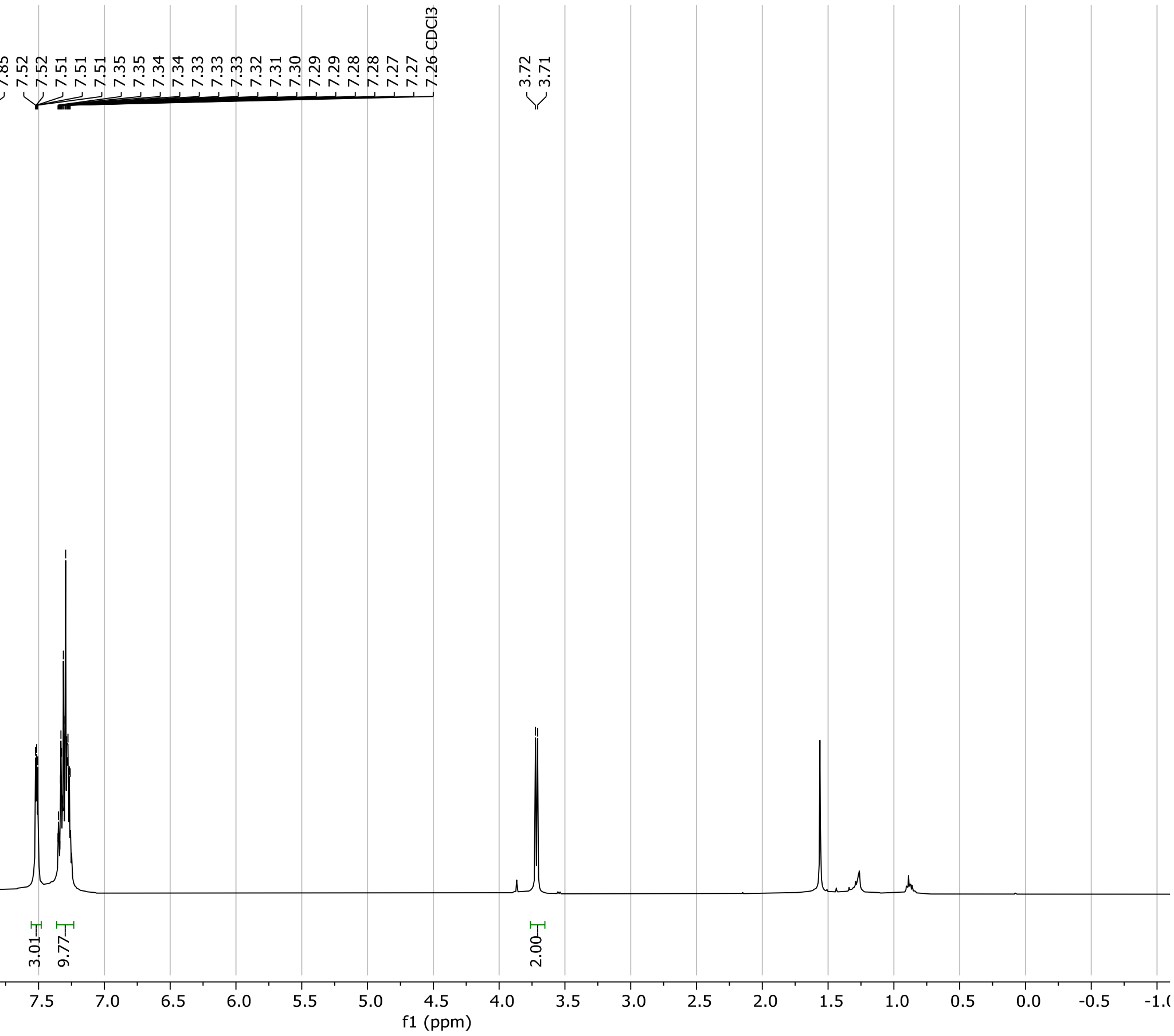

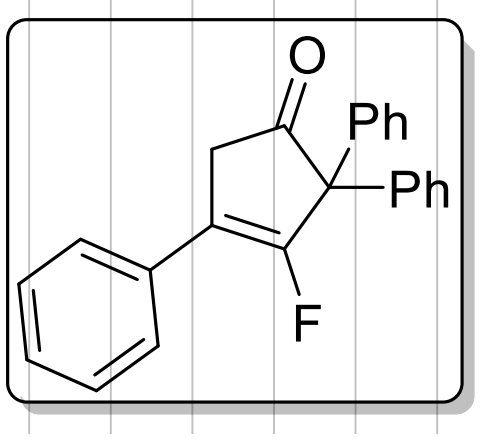

5c
^{19}F NMR
(377 MHz, $CDCl_3$)

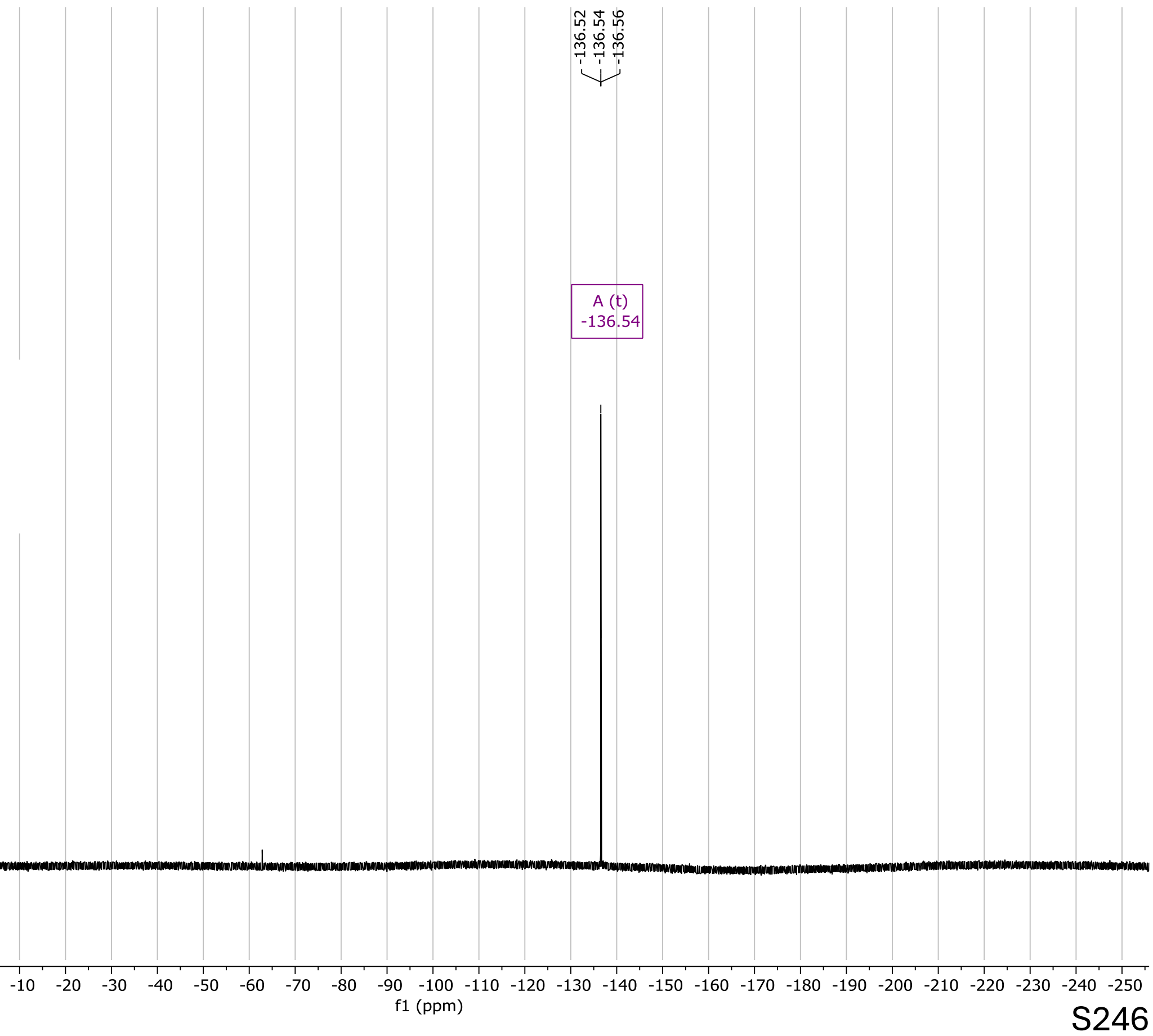

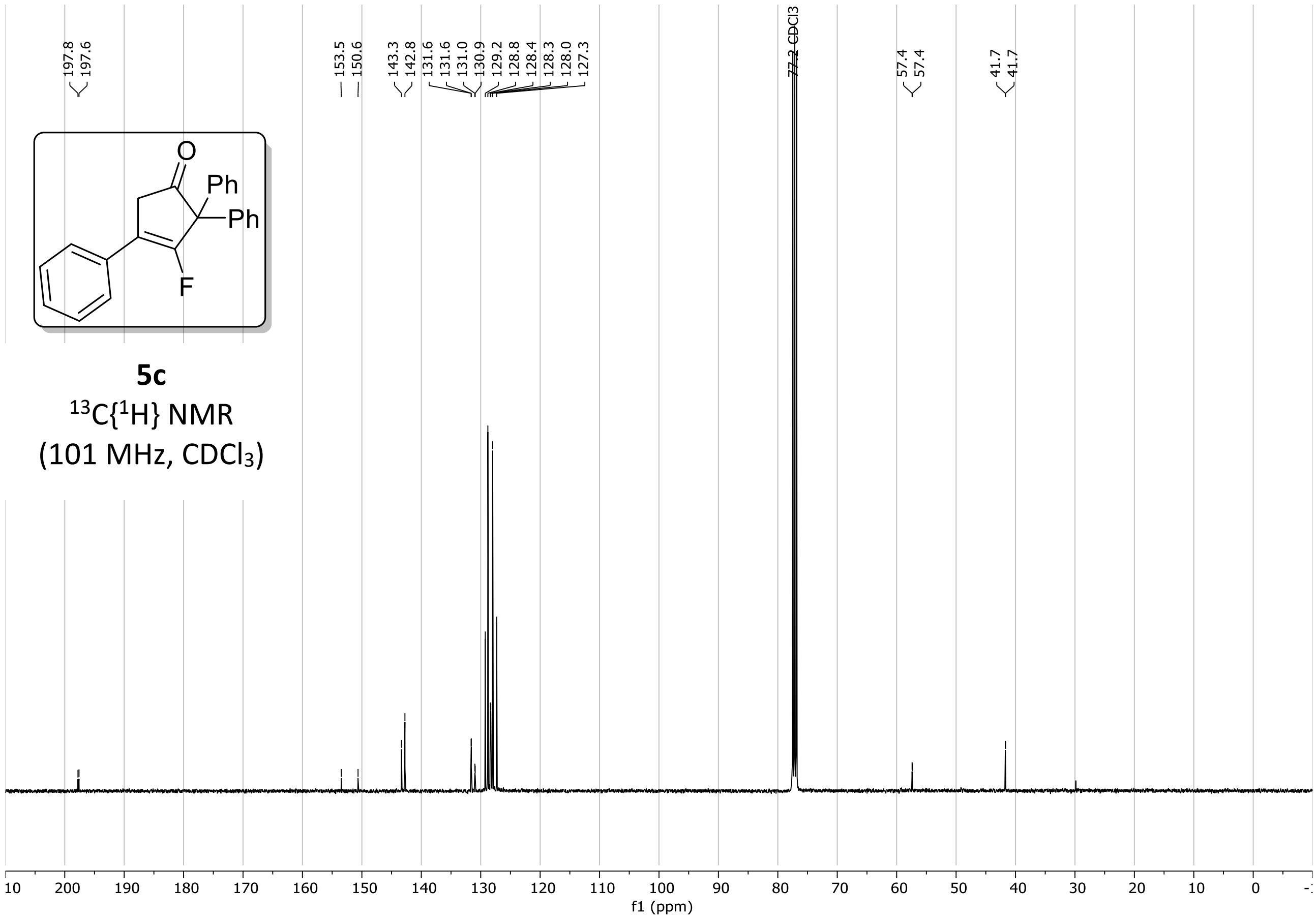

5c

$^{13}C\{^{1}H\}$ NMR

(101 MHz, $CDCl_3$)

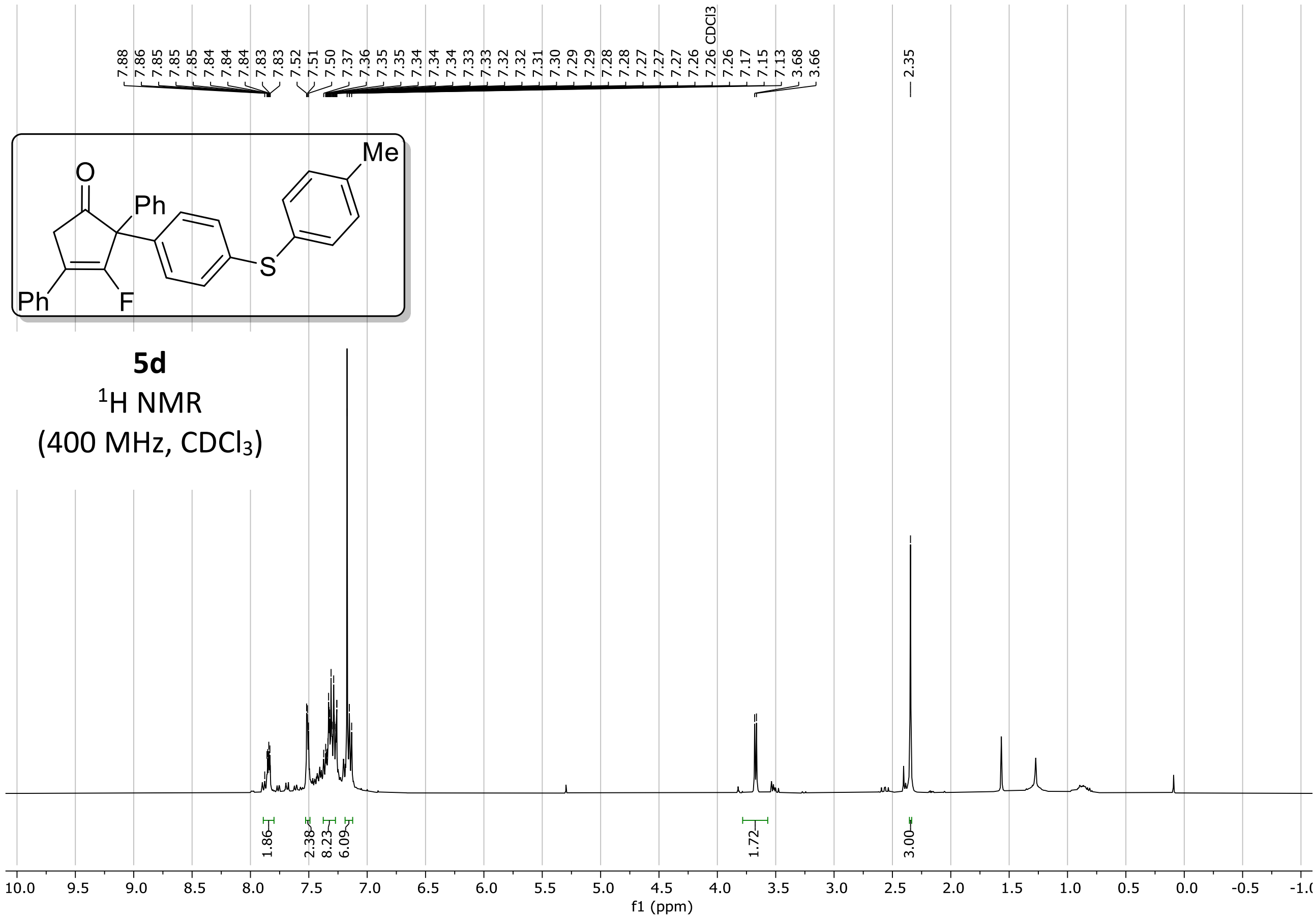

5d
^{1}H NMR
(400 MHz, $CDCl_3$)

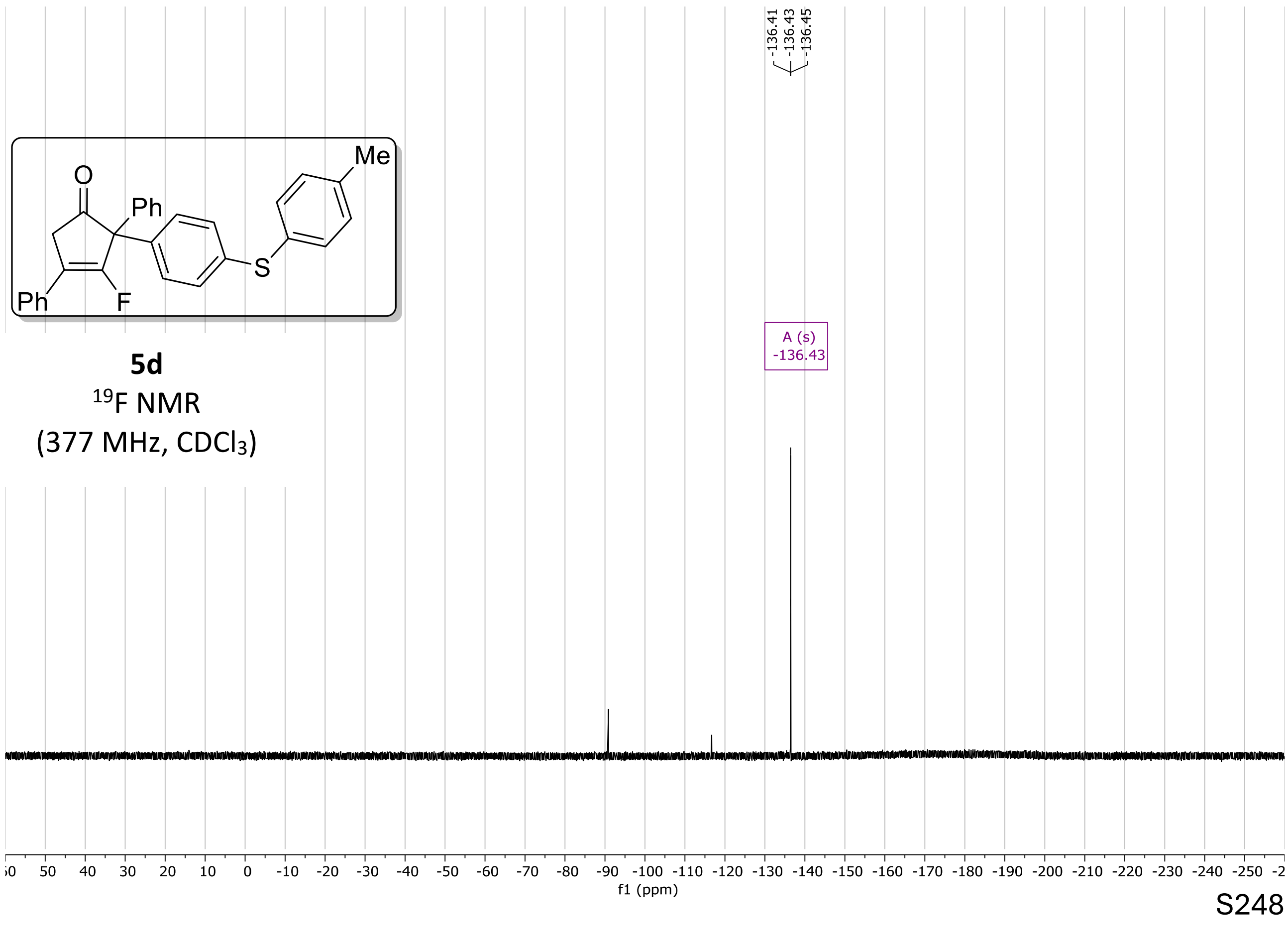

5d
^{19}F NMR
(377 MHz, $CDCl_3$)

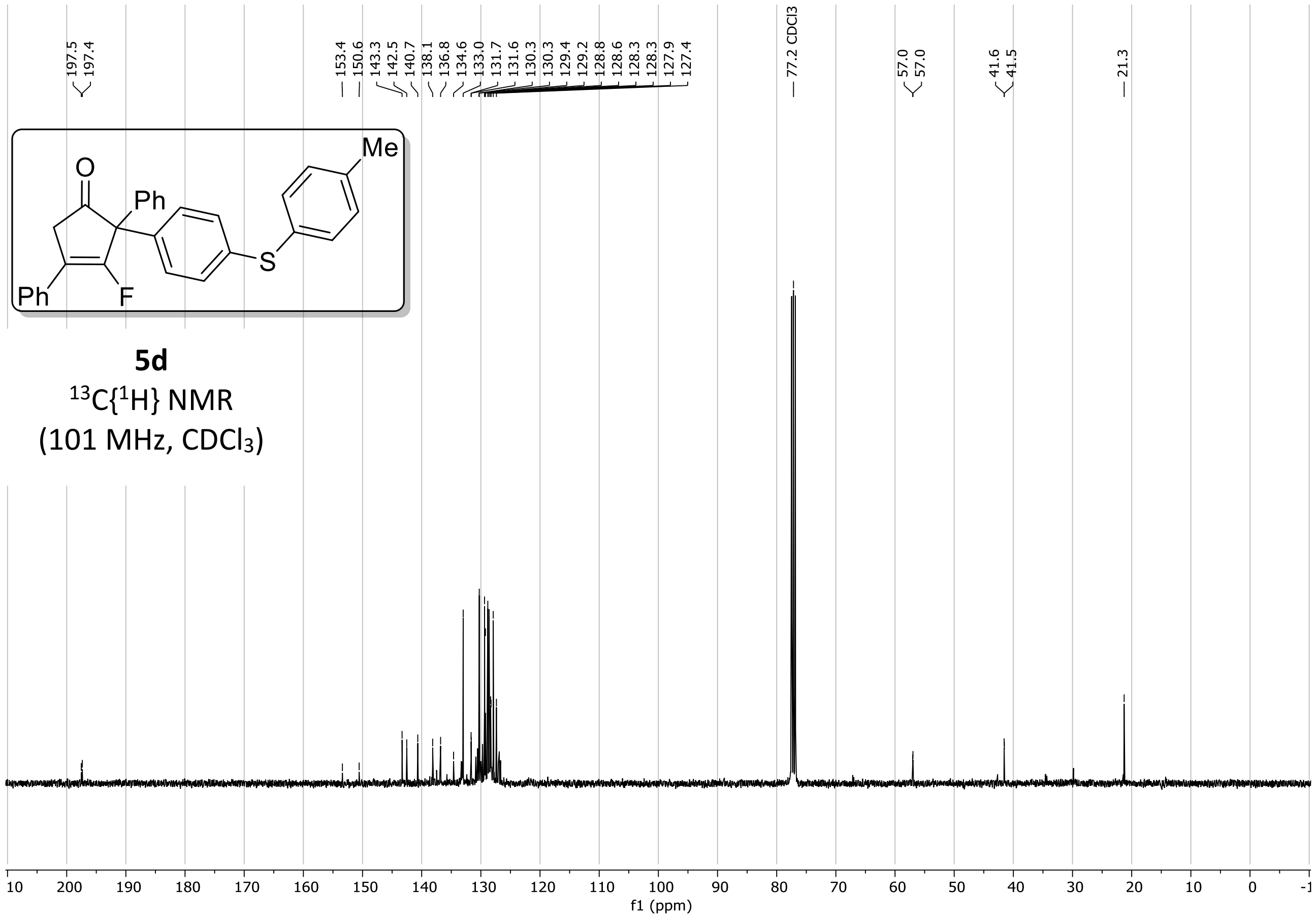

5d
$^{13}C\{^1H\}$ NMR
(101 MHz, $CDCl_3$)

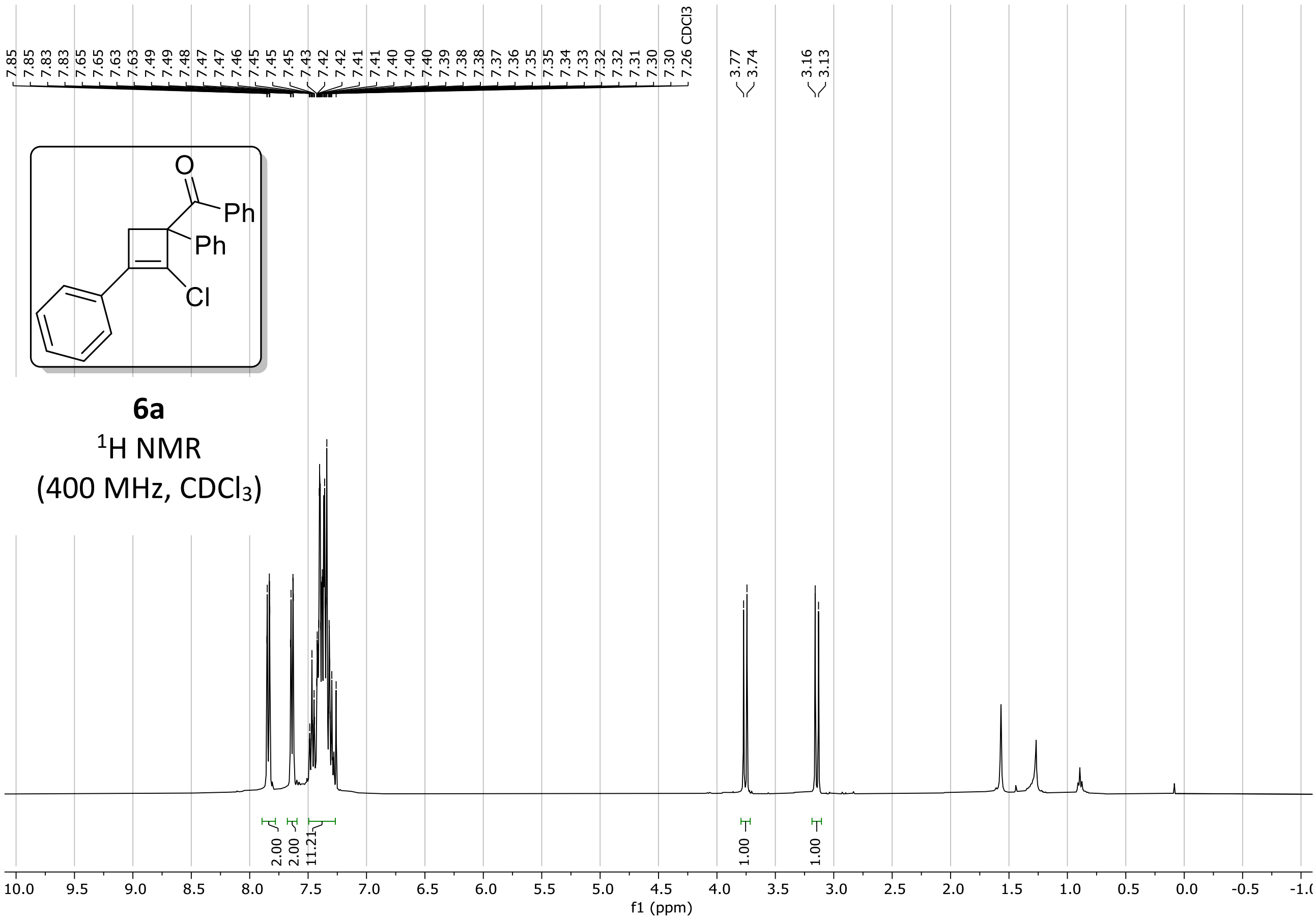
7.85 7.85 7.83 7.83 7.65 7.65 7.63 7.63 7.49 7.49 7.48 7.47 7.47 7.46 7.45 7.45 7.45 7.43 7.42 7.42 7.41 7.41 7.40 7.40 7.40 7.39 7.38 7.38 7.37 7.36 7.35 7.35 7.34 7.33 7.32 7.32 7.31 7.30 7.30 7.26 CDCl3
3.77 3.74
3.16 3.13
O
Ph
Ph
Cl
6a
1H NMR
(400 MHz, CDCl3)
2.00 2.00 11.21
1.00
1.00
10.0 9.5 9.0 8.5 8.0 7.5 7.0 6.5 6.0 5.5 5.0 4.5 4.0 3.5 3.0 2.5 2.0 1.5 1.0 0.5 0.0 -0.5
f1 (ppm)

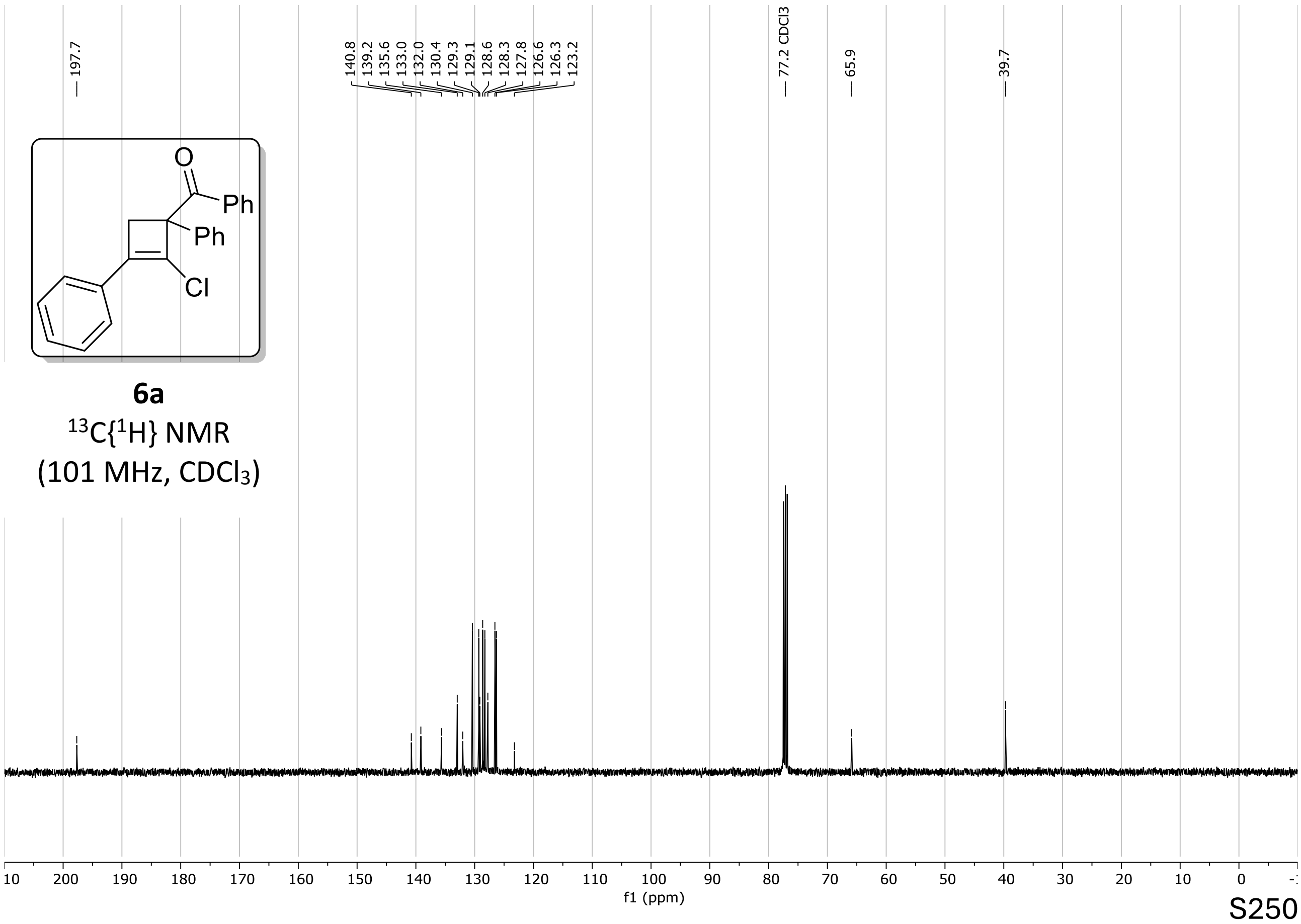
197.7
140.8 139.2 135.6 133.0 132.0 130.4 129.3 129.1 128.6 128.3 127.8 126.6 126.3 123.2
77.2 CDCl3
65.9
39.7
O
Ph
Ph
Cl
6a
13C{1H} NMR
(101 MHz, CDCl3)
200 190 180 170 160 150 140 130 120 110 100 90 80 70 60 50 40 30 20 10 0
f1 (ppm)

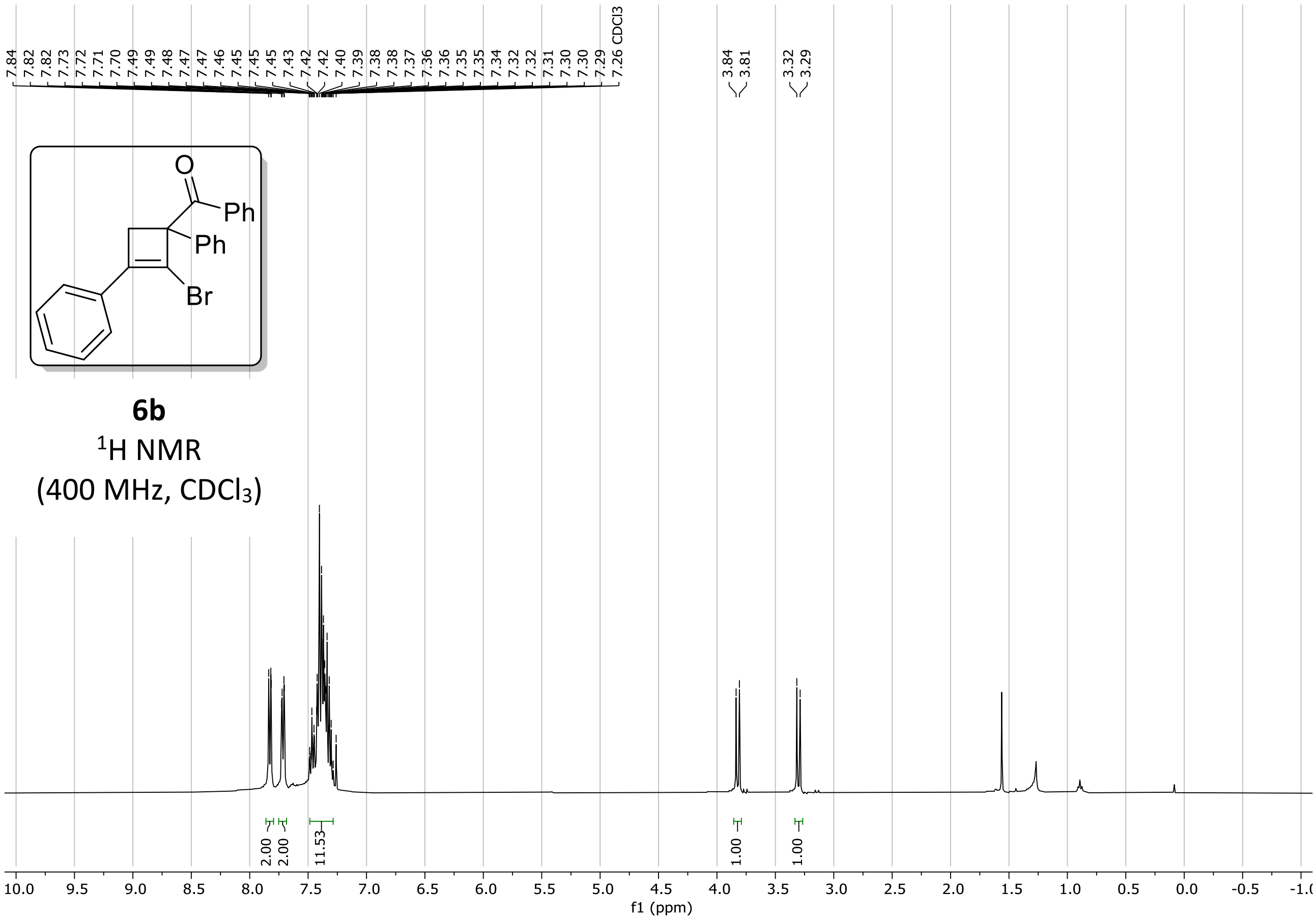

6b

^1H NMR

(400 MHz, $CDCl_3$)

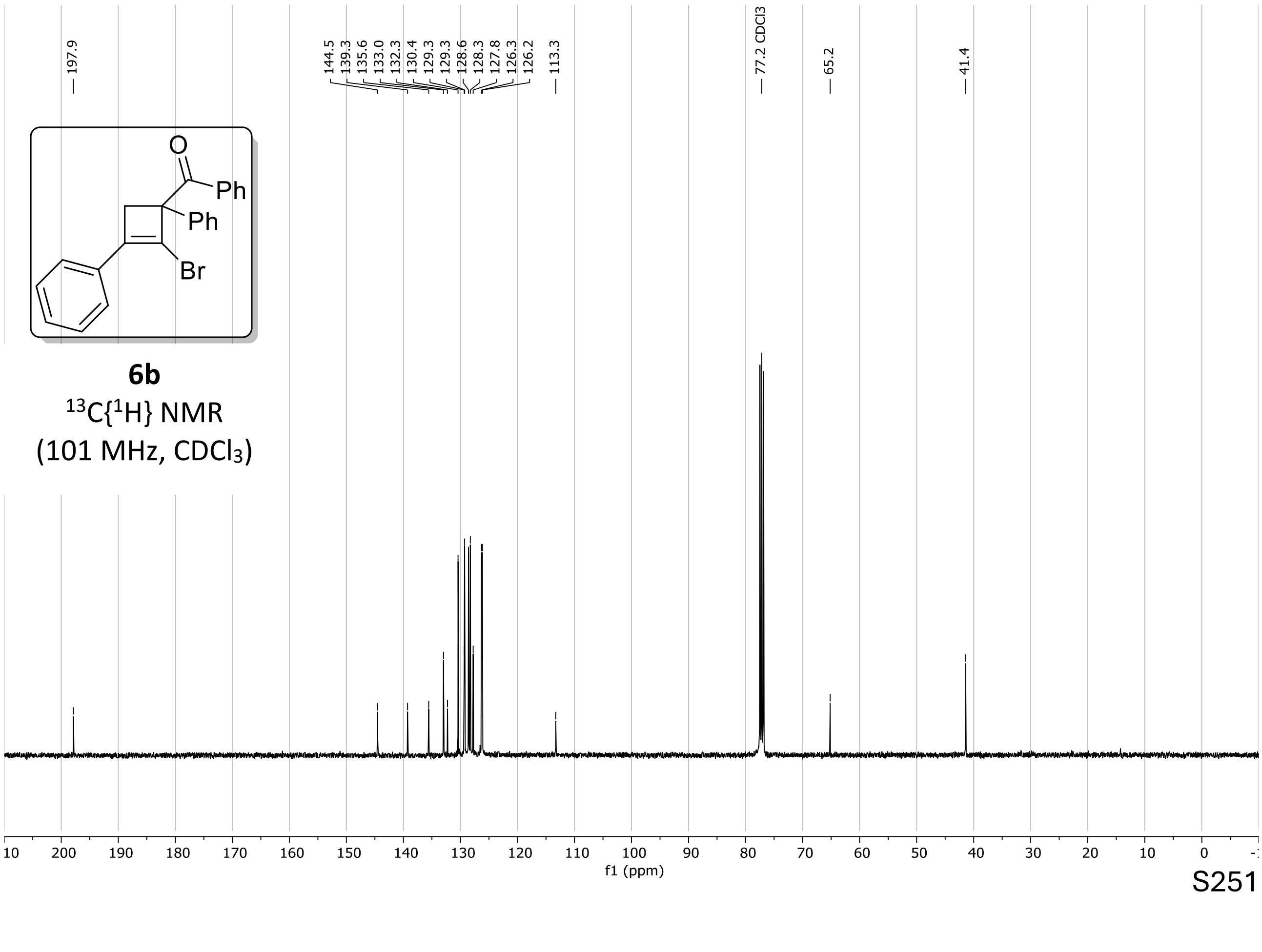

6b

$^{13}C\{^1H\}$ NMR

(101 MHz, $CDCl_3$)

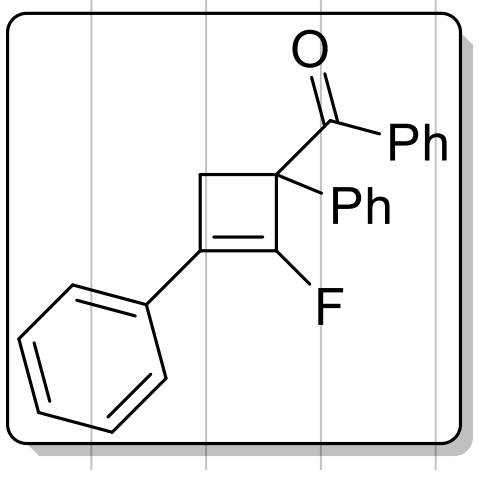

6c

^{1}H NMR

(400 MHz, $CDCl_3$)

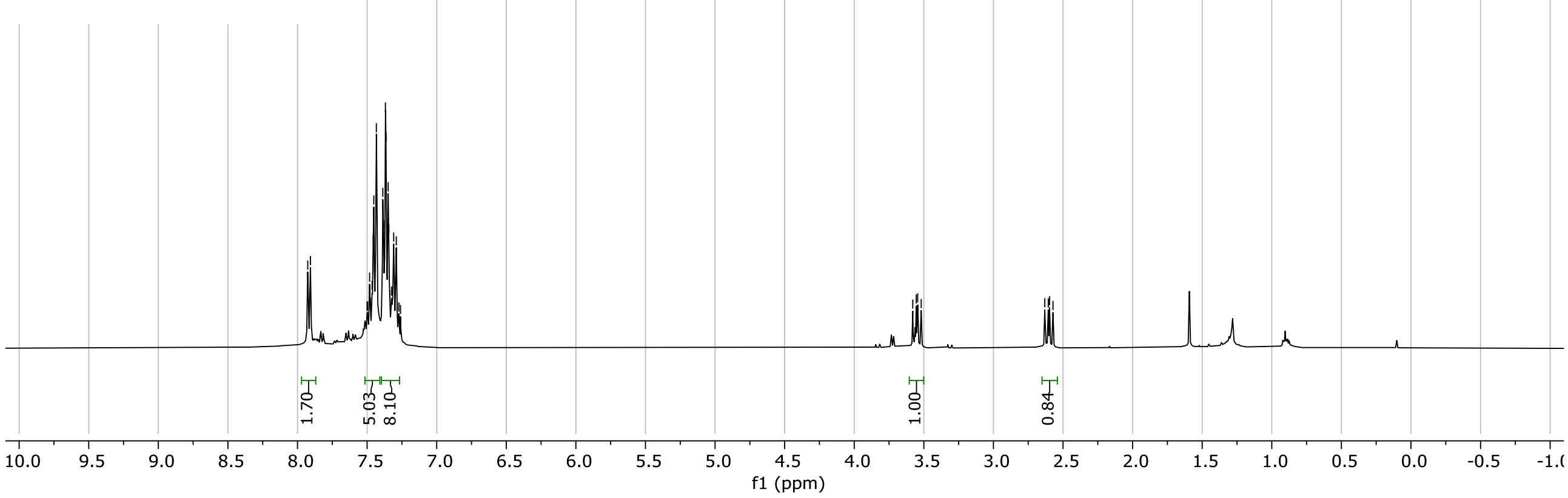

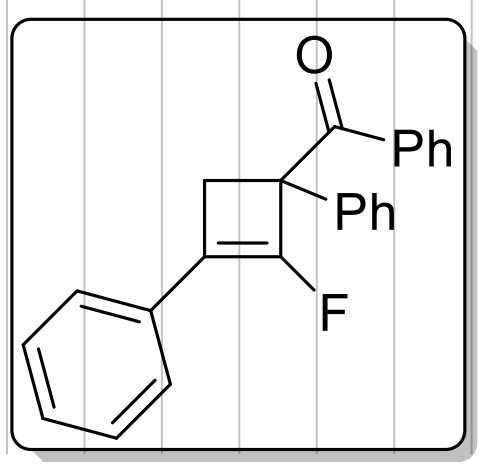

6c

^{19}F NMR

(377 MHz, $CDCl_3$)

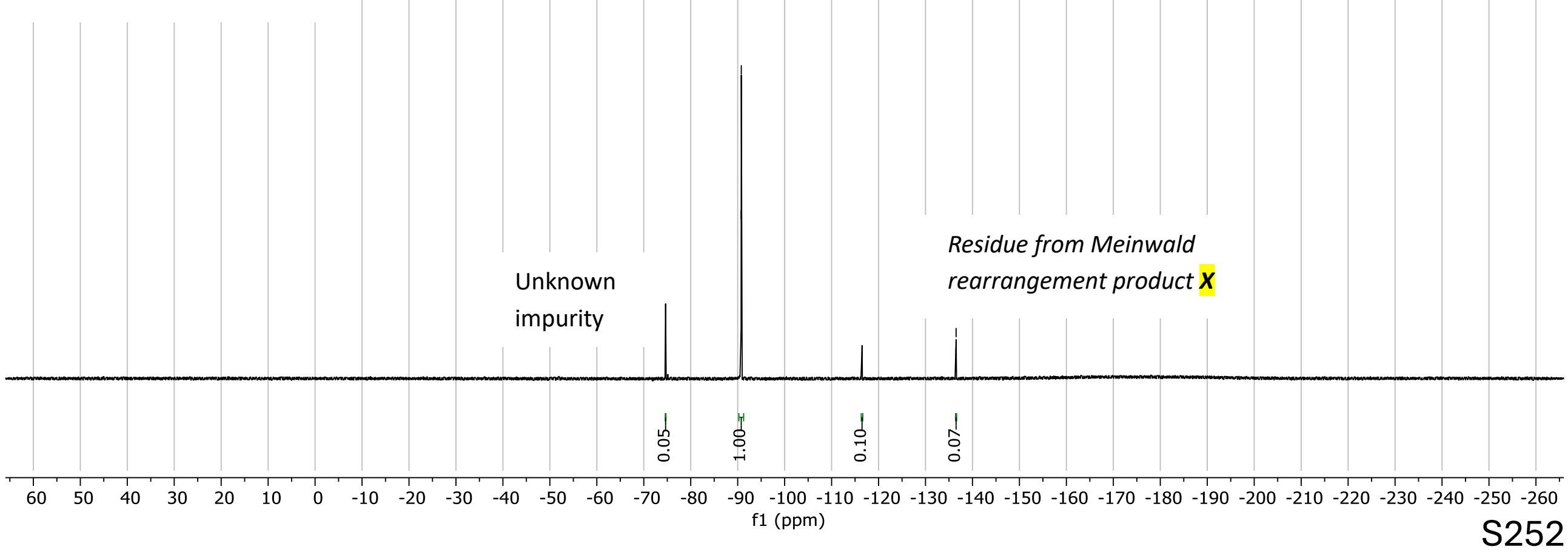

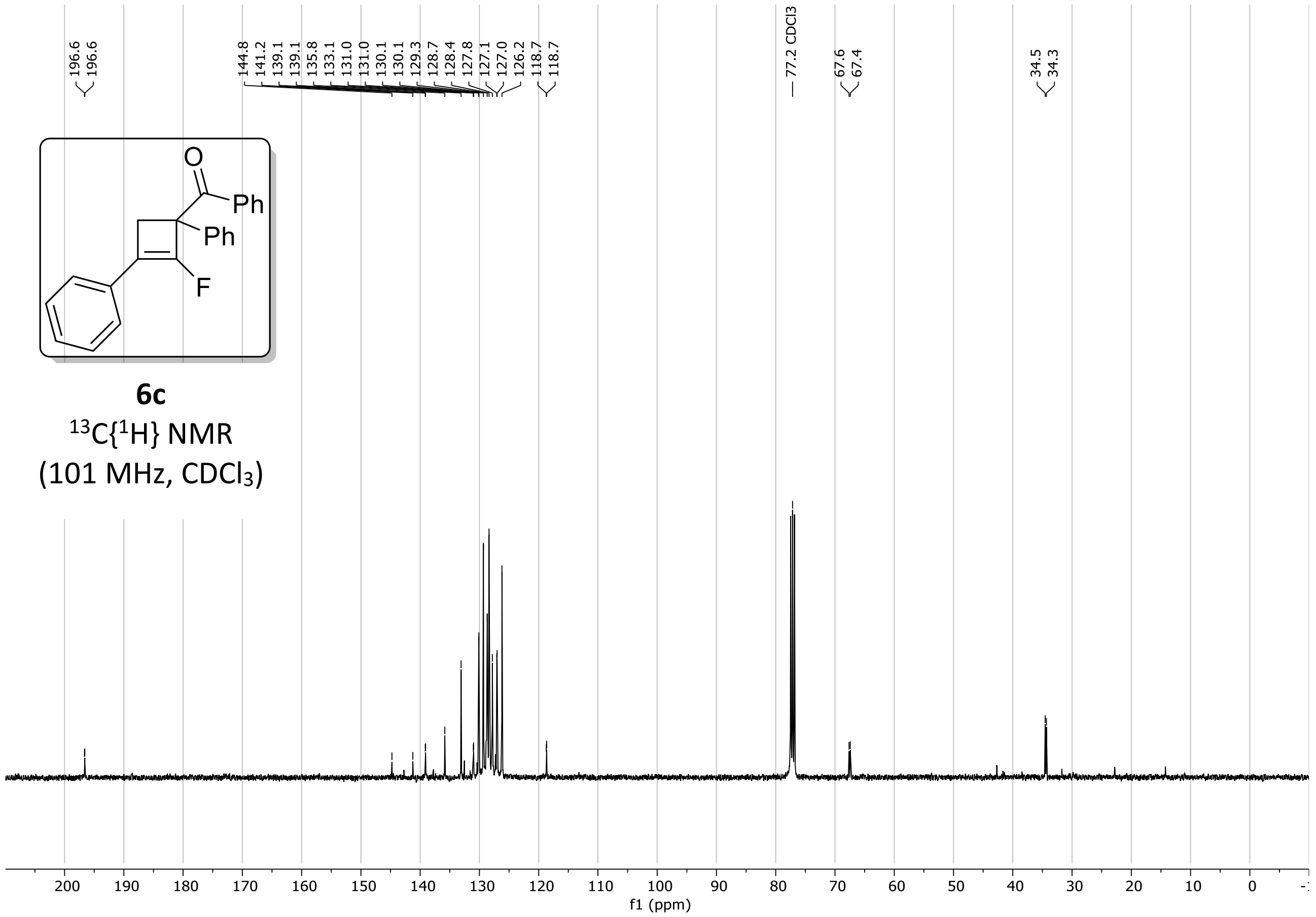
196.6
196.6
144.8
141.2
139.1
139.1
135.8
133.1
131.0
131.0
130.1
130.1
129.3
128.7
128.4
127.8
127.1
127.0
126.2
118.7
118.7
77.2 CDCl3
67.6
67.4
34.5
34.3
O
Ph
Ph
F
6c
13C{1H} NMR
(101 MHz, CDCl3)
200
190
180
170
160
150
140
130
120
110
100
90
80
70
60
50
40
30
20
10
0
f1 (ppm)

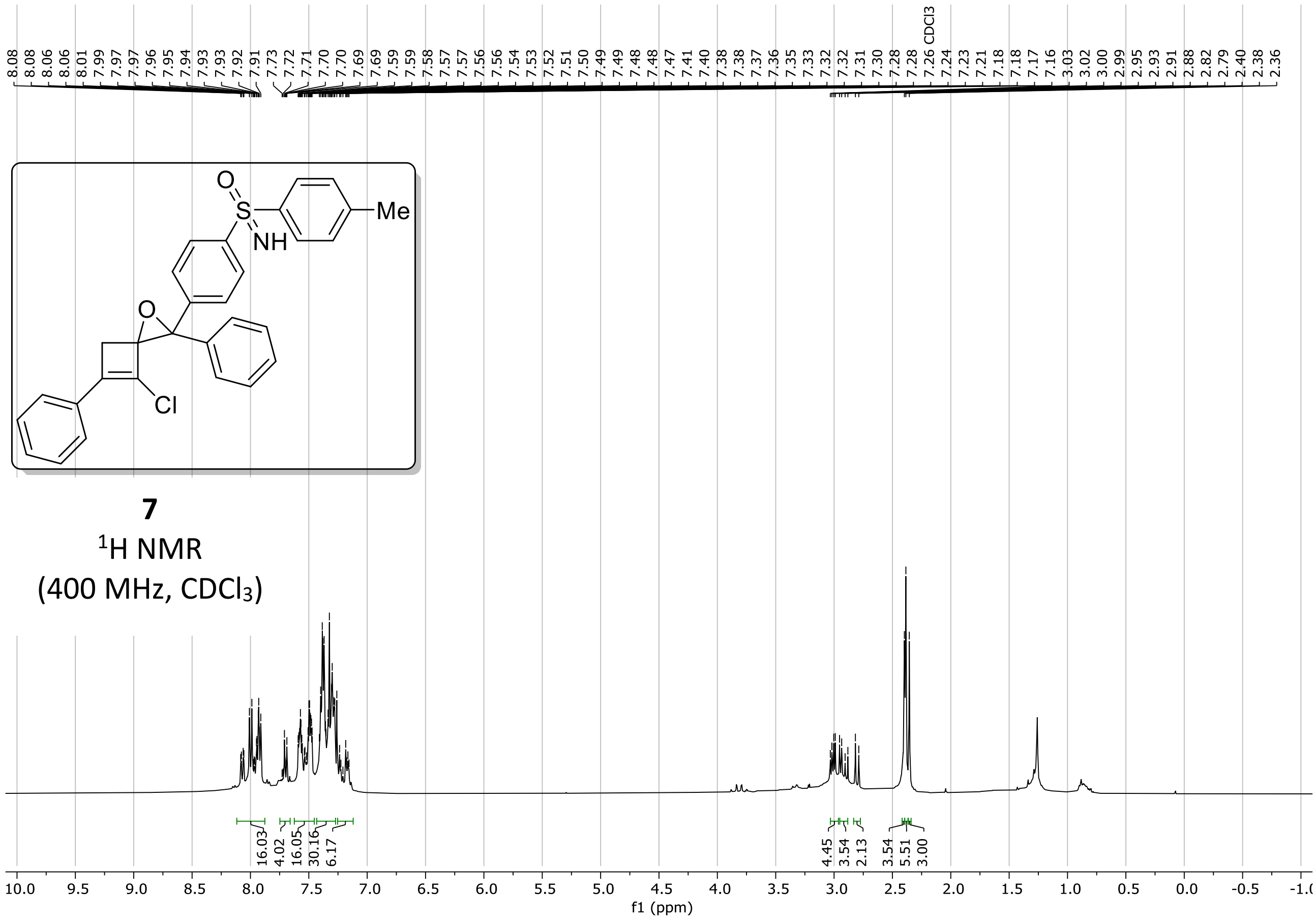

7
^{1}H NMR
(400 MHz, $CDCl_3$)

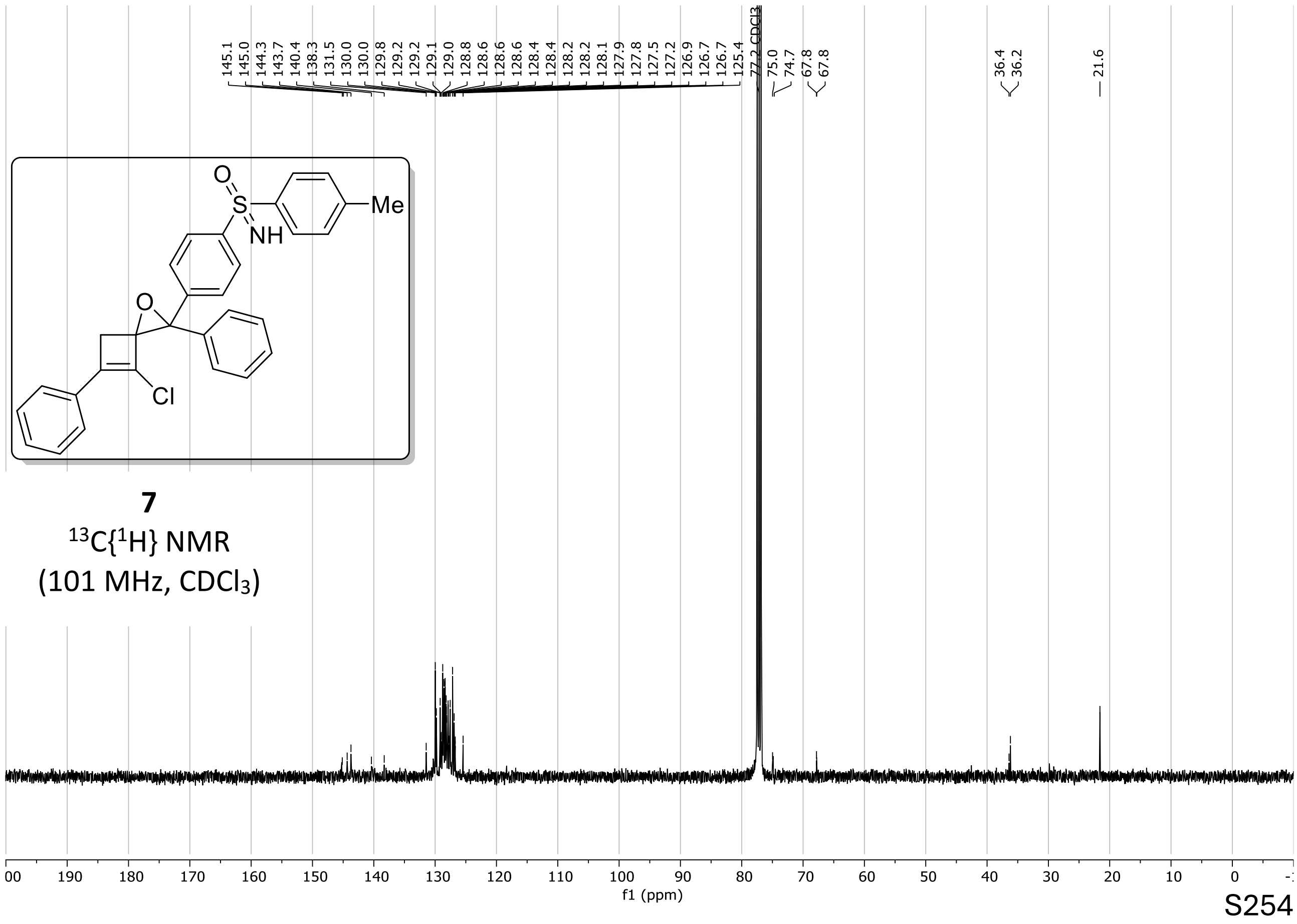

7
^{13}C{^{1}H} NMR
(101 MHz, $CDCl_3$)

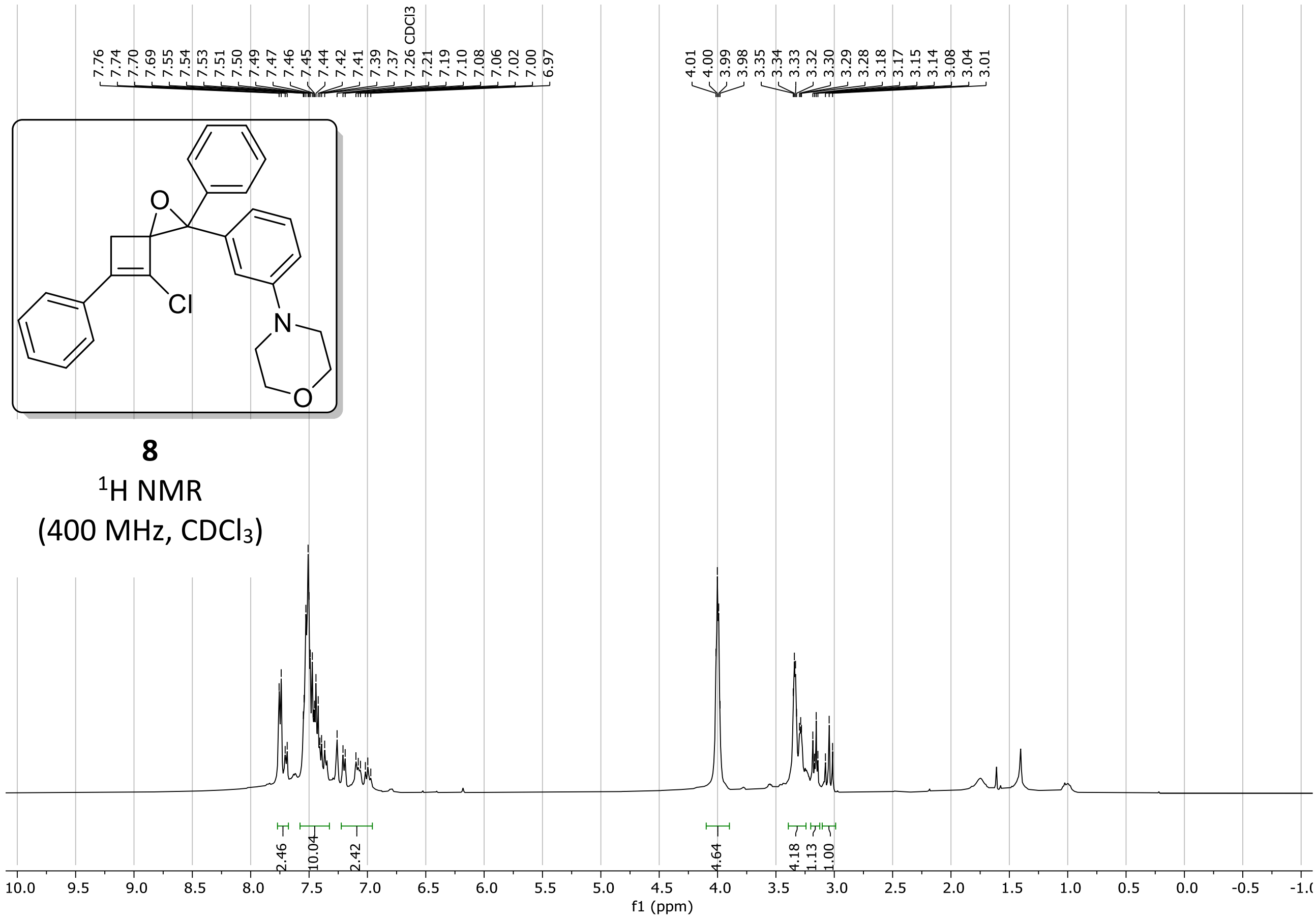
7.76 7.74 7.70 7.69 7.55 7.54 7.53 7.51 7.50 7.49 7.47 7.46 7.45 7.44 7.42 7.41 7.39 7.37 7.26 CDCl3 7.21 7.19 7.10 7.08 7.06 7.02 7.00 6.97
4.01 4.00 3.99 3.98 3.35 3.34 3.33 3.32 3.30 3.29 3.28 3.18 3.17 3.15 3.14 3.08 3.04 3.01
O
Cl
N
O
8
1H NMR
(400 MHz, CDCl3)
2.46 10.04 2.42 4.64 4.18 1.13 1.00
10.0 9.5 9.0 8.5 8.0 7.5 7.0 6.5 6.0 5.5 5.0 4.5 4.0 3.5 3.0 2.5 2.0 1.5 1.0 0.5 0.0 -0.5
f1 (ppm)

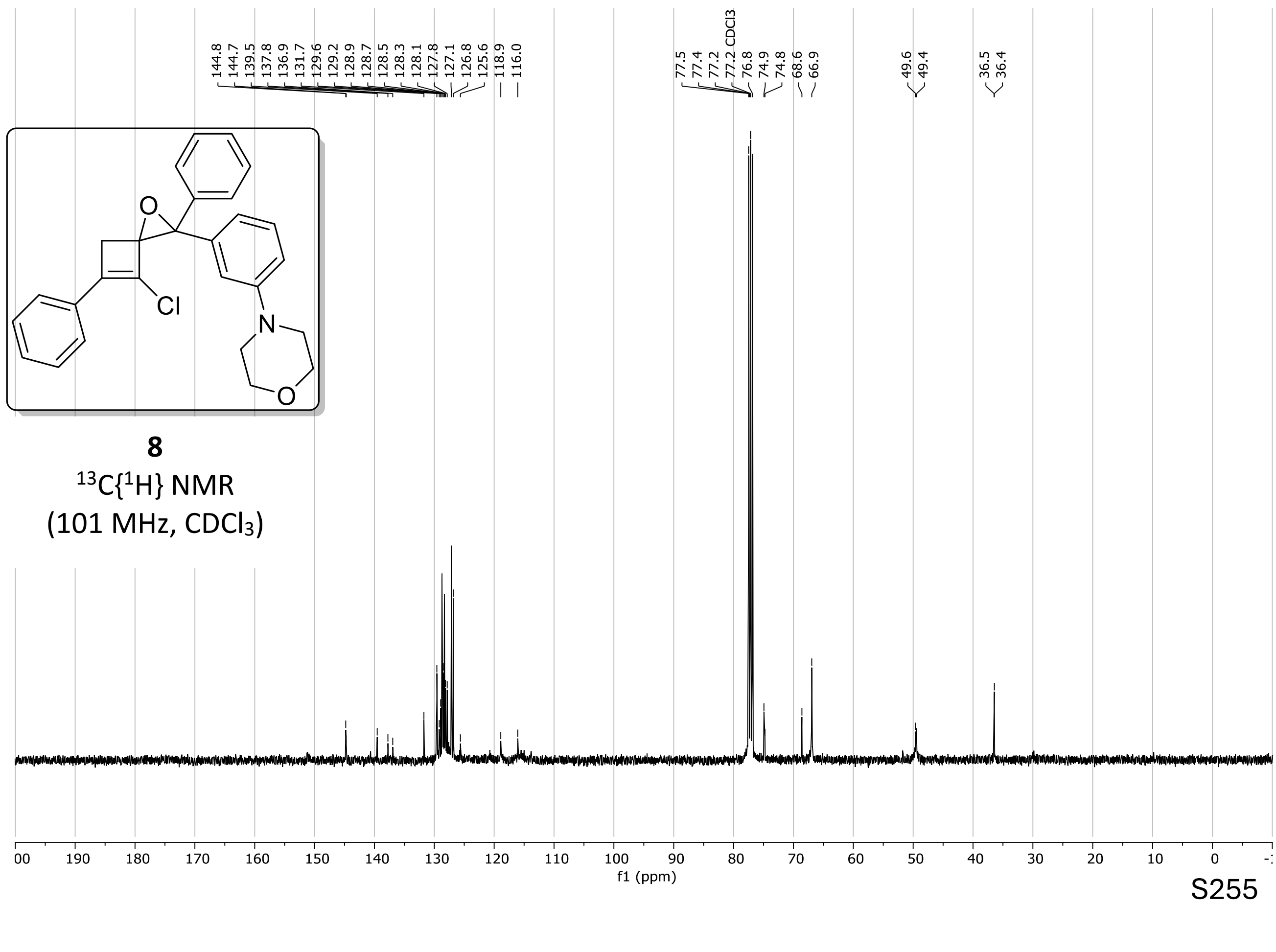
144.8 144.7 139.5 137.8 136.9 131.7 129.6 129.2 128.9 128.7 128.5 128.3 128.1 127.8 127.1 126.8 125.6 118.9 116.0
77.5 77.4 77.2 CDCl3 76.8 74.9 74.8 68.6 66.9
49.6 49.4
36.5 36.4
O
Cl
N
O
8
13C{1H} NMR
(101 MHz, CDCl3)
190 180 170 160 150 140 130 120 110 100 90 80 70 60 50 40 30 20 10 0
f1 (ppm)

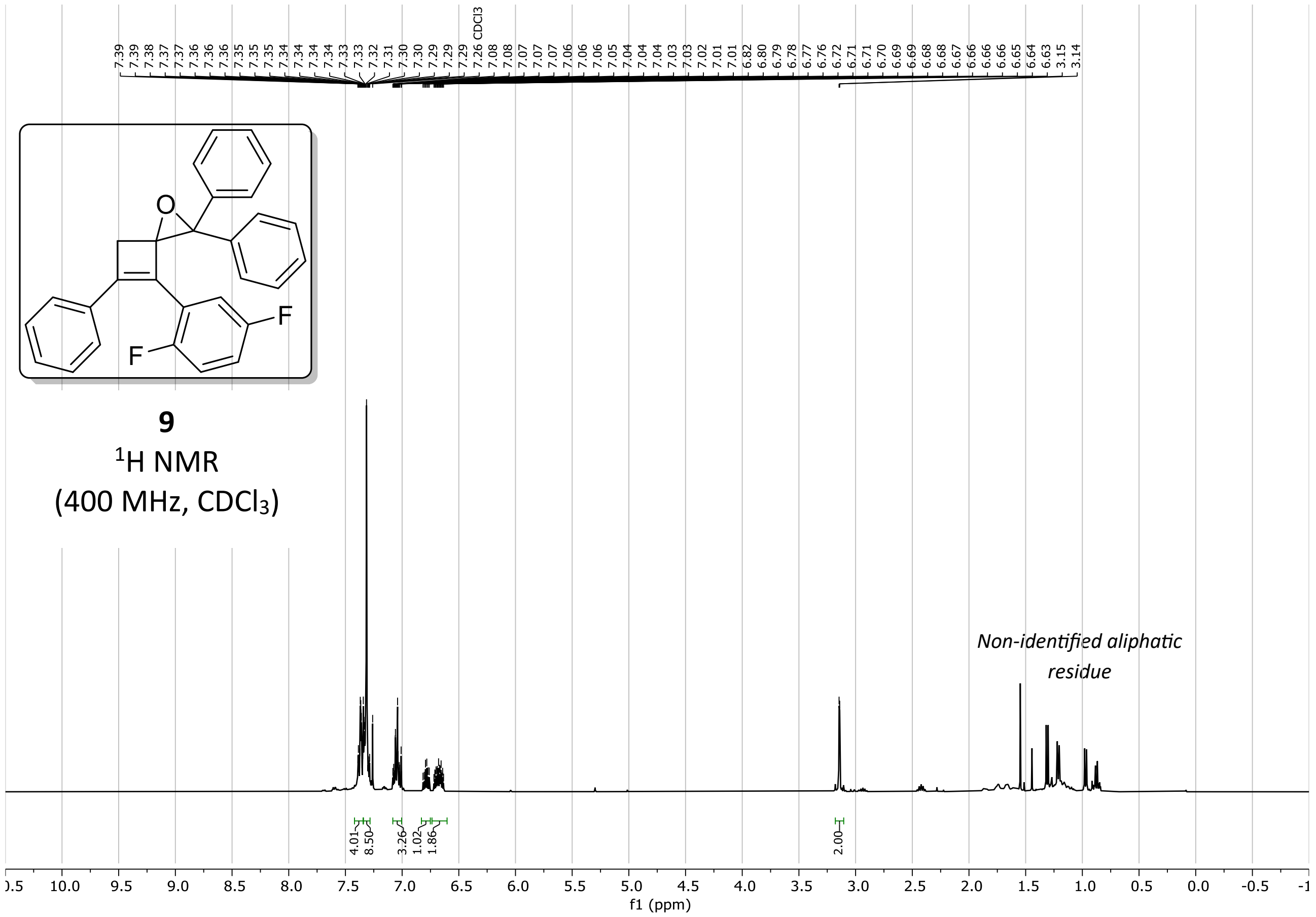

9
^{1}H NMR
(400 MHz, $CDCl_3$)

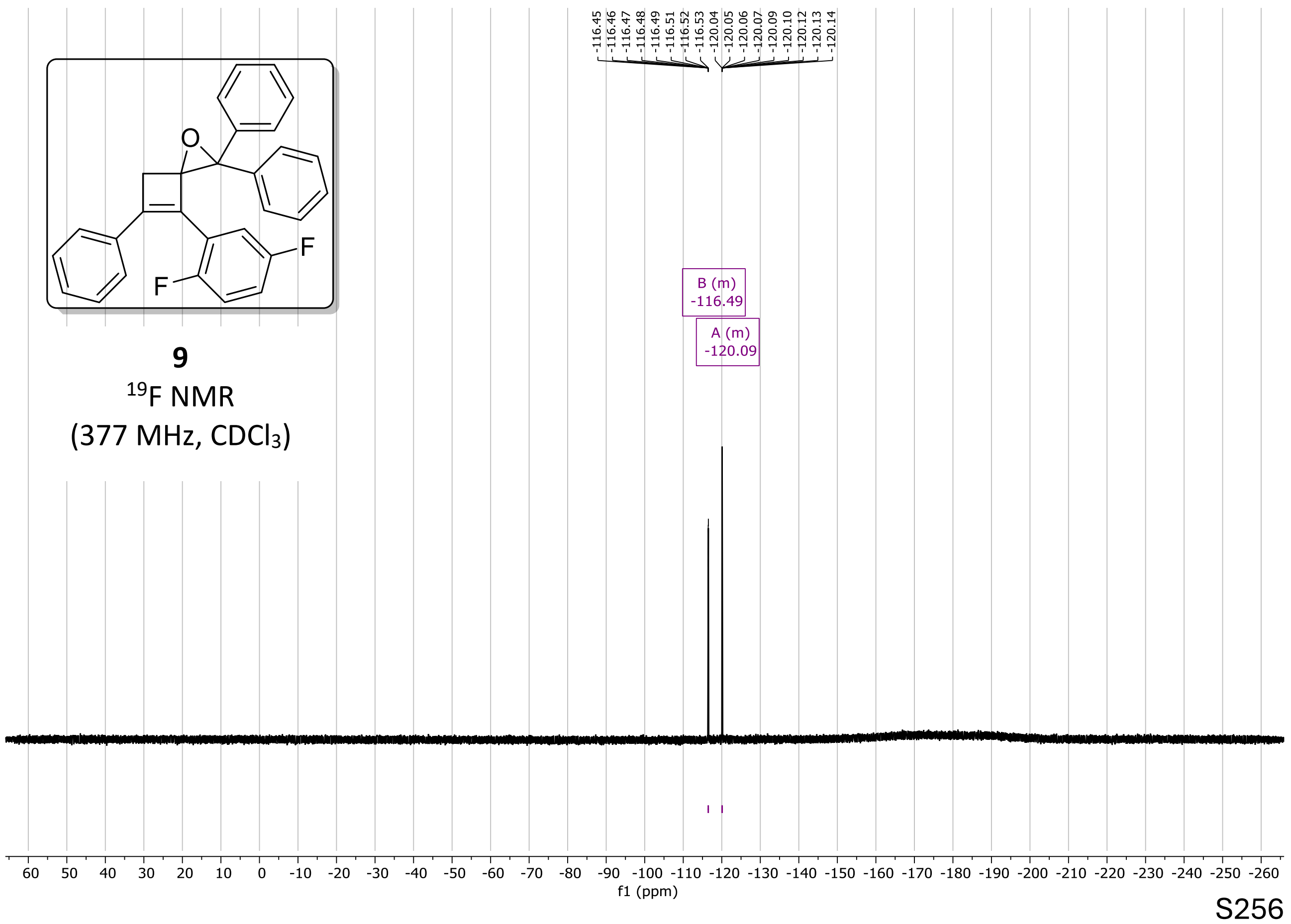

9
^{19}F NMR
(377 MHz, $CDCl_3$)

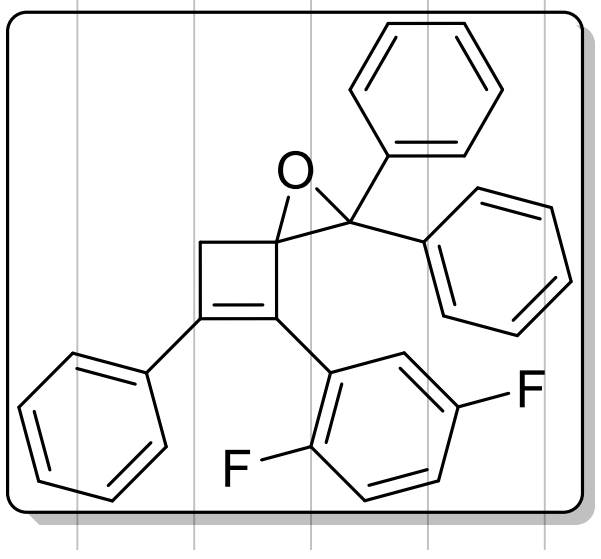

9

$^{13}C\{^{1}H\}$ NMR

(101 MHz, $CDCl_3$)

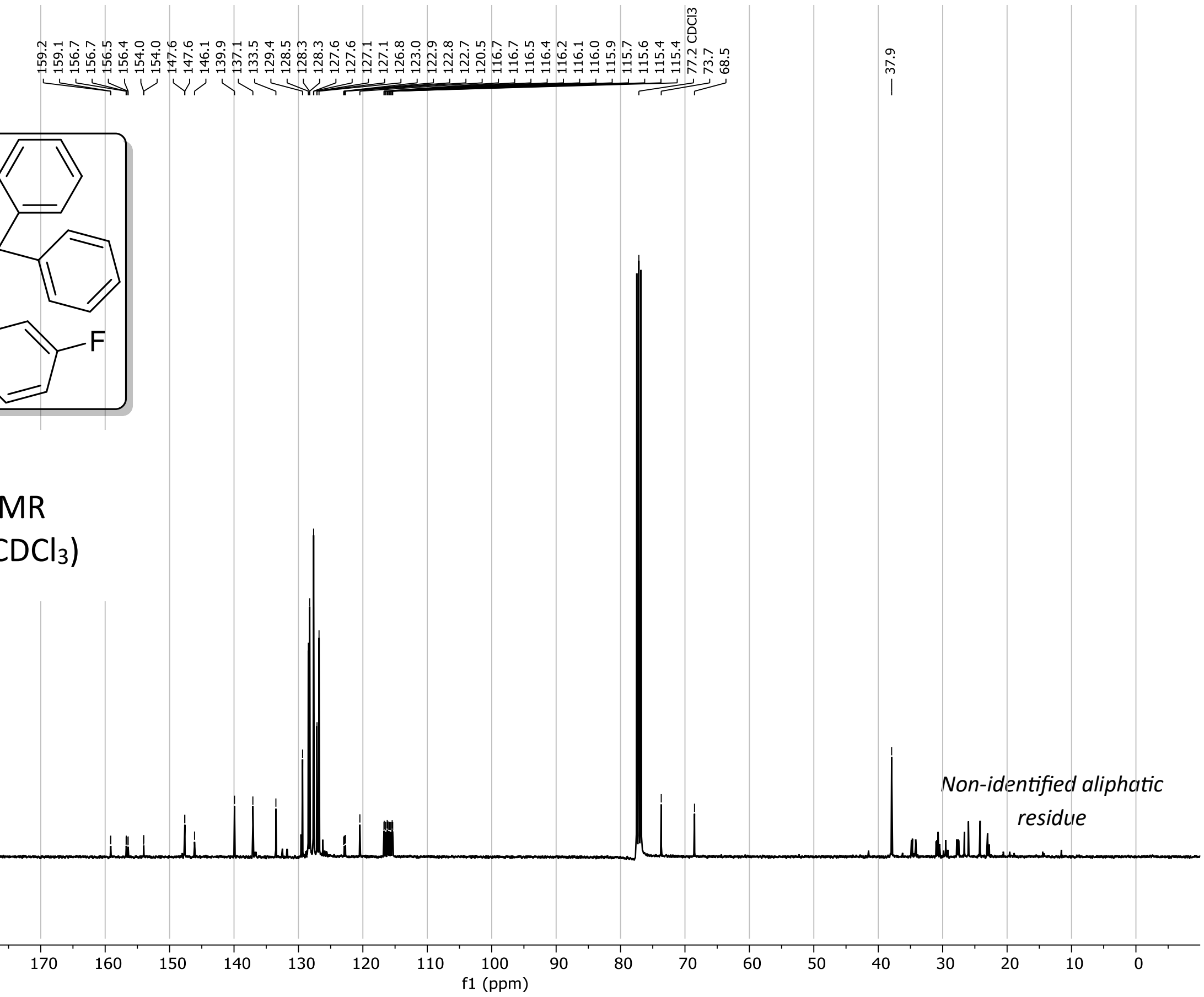

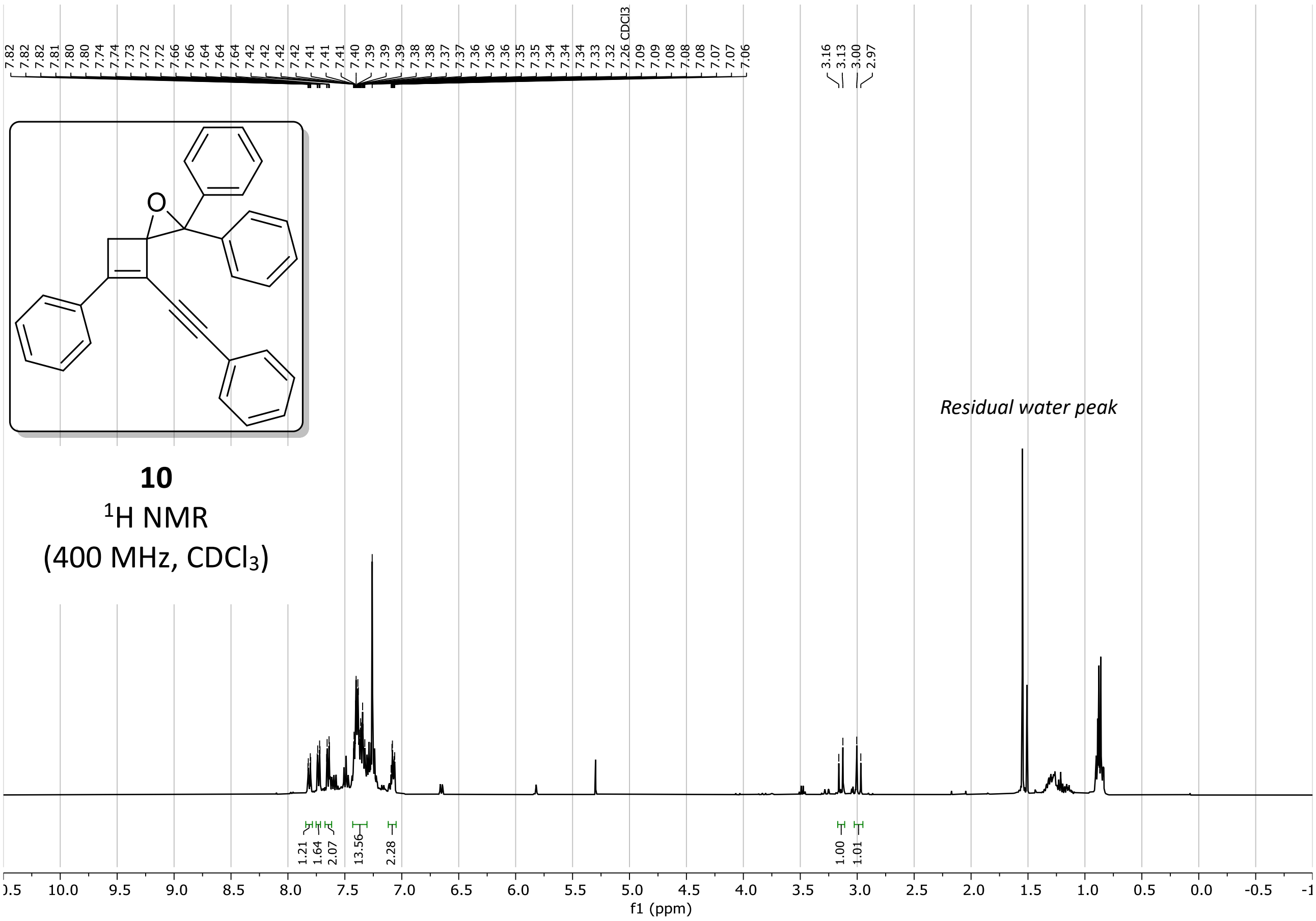

10
^{1}H NMR
(400 MHz, $CDCl_3$)

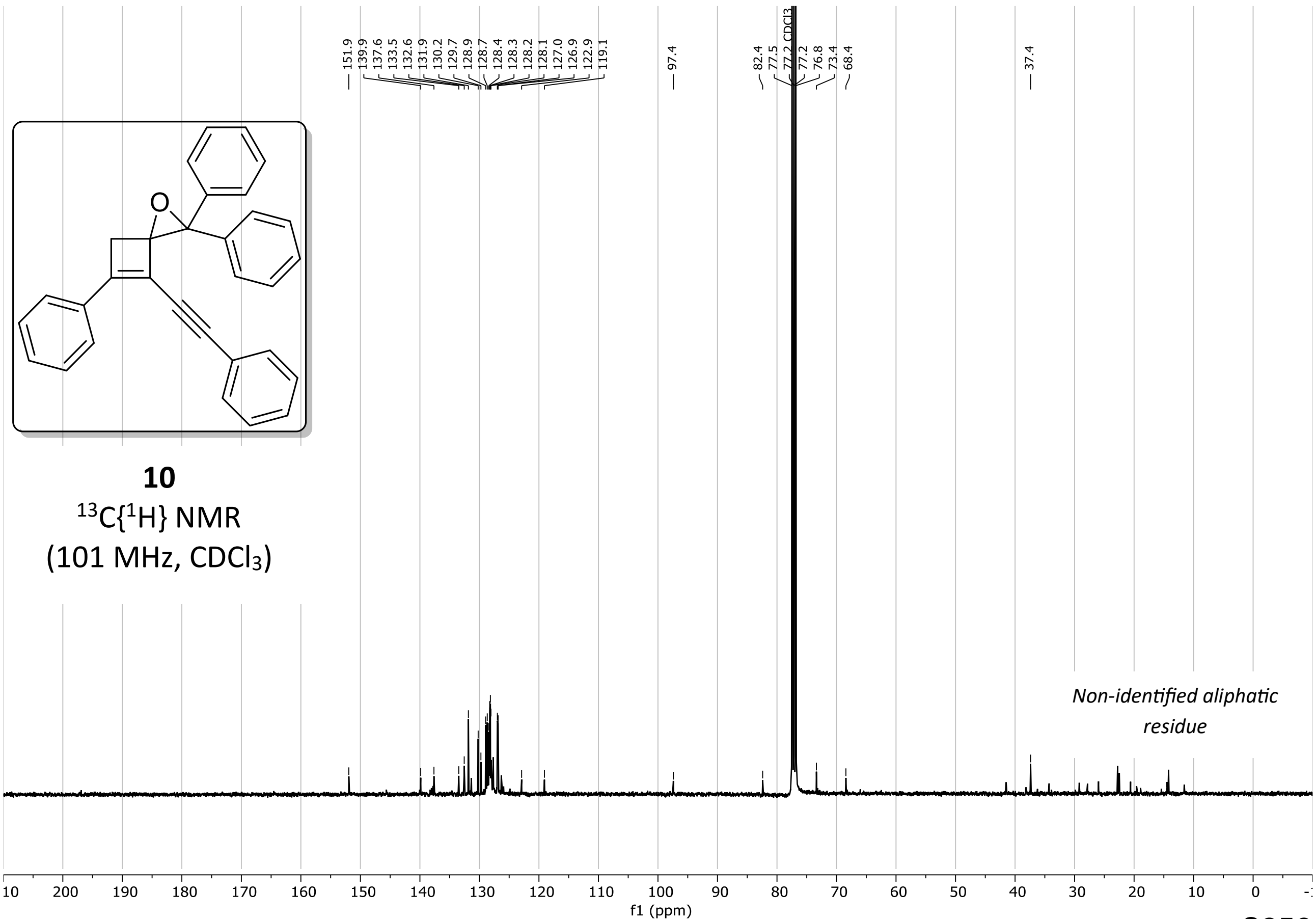

10
$^{13}C\{^{1}H\}$ NMR
(101 MHz, $CDCl_3$)
